# Chiral Dibenzopentalene‐Based Conjugated Nanohoops through Stereoselective Synthesis

**DOI:** 10.1002/anie.202016968

**Published:** 2021-03-23

**Authors:** Mathias Hermann, Daniel Wassy, Julia Kohn, Philipp Seitz, Martin U. Betschart, Stefan Grimme, Birgit Esser

**Affiliations:** ^1^ Institute for Organic Chemistry University of Freiburg Albertstr. 21 79104 Freiburg Germany; ^2^ Mulliken Center for Theoretical Chemistry University of Bonn Beringstr. 4 53115 Bonn Germany; ^3^ Institut für Pharmazeutische Wissenschaften University of Freiburg Albertstr. 25 79104 Freiburg Germany; ^4^ Freiburg Materials Research Center University of Freiburg Stefan-Meier-Str. 21 79104 Freiburg Germany; ^5^ Freiburg Center for Interactive Materials and Bioinspired Technologies University of Freiburg Georges-Köhler-Allee 105 79110 Freiburg Germany

**Keywords:** antiaromaticity, chiral macrocycles, chiral resolution, cycloparaphenylenes, fullerenes

## Abstract

Conjugated nanohoops allow to investigate the effect of radial conjugation and bending on the involved π‐systems. They can possess unexpected optoelectronic properties and their radially oriented π‐system makes them attractive for host–guest chemistry. Bending the π‐subsystems can lead to chiral hoops. Herein, we report the stereoselective synthesis of two enantiomers of chiral conjugated nanohoops by incorporating dibenzo[*a*,*e*]pentalenes (DBPs), which are generated in the last synthetic step from enantiomerically pure diketone precursors. Owing to its bent shape, this diketone unit was used as the only bent precursor and novel “corner unit” in the synthesis of the hoops. The [6]DBP[4]Ph‐hoops contain six antiaromatic DBP units and four bridging phenylene groups. The small HOMO–LUMO gap and ambipolar electrochemical character of the DBP units is reflected in the optoelectronic properties of the hoop. Electronic circular dichroism spectra and MD simulations showed that the chiral hoop did not racemize even when heated to 110 °C. Due to its large diameter, it was able to accommodate two C60 molecules, as binding studies indicate.

## Graphical abstract

Chiral, dibenzo[*a*,*e*]pentalene‐based conjugated nanohoops were synthesized in a stereoselective way from enantiomerically pure diketone precursors. The hoop shows ambipolar electrochemical behavior due to the antiaromaticity of the dibenzopentalene units and can accommodate two fullerene molecules. Circular dichroism measurements and molecular dynamics simulations indicated no racemization even at 110 °C.

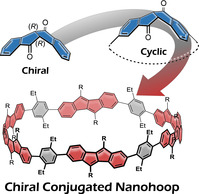

## Introduction

**Figure 1:**
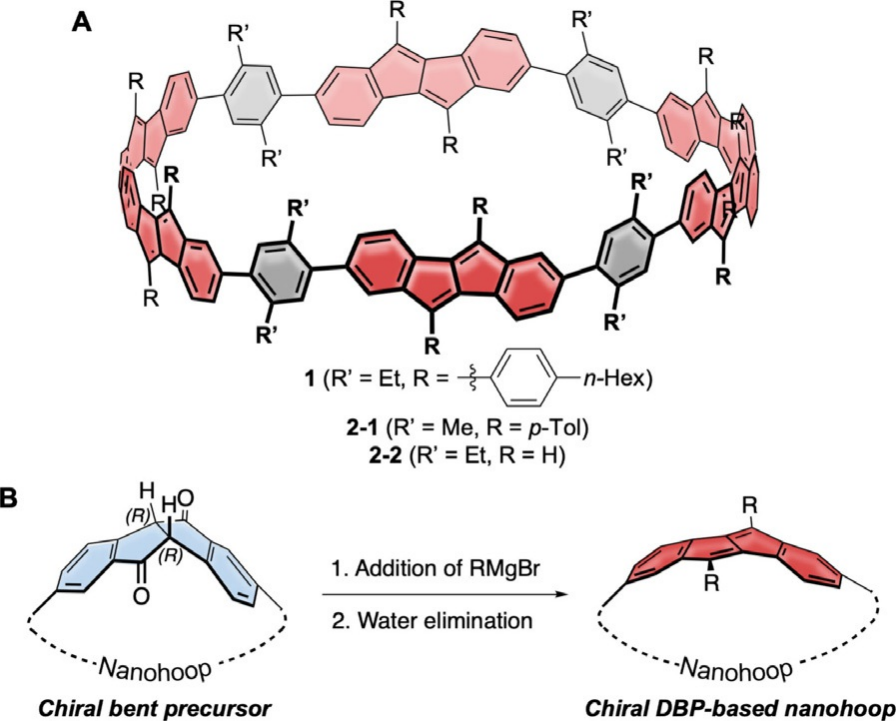
A) DBP‐based chiral nanohoop **1** synthesized herein (and derivatives **2‐1** and **2‐2** used for calculations); B) Synthetic strategy using chiral, enantiopure diketone units as precursors to bent DBP units.

Conjugated nanohoops have become a major research area in recent years.^[1]^ While hoop‐shaped molecules have fascinated chemists since the 1950s,[^1^, ^2^, ^3^] the interest in the field has rapidly increased since 2008, when the first [*n*]cycloparaphenylenes ([*n*]CPPs) were synthesized.[^4^, ^5^, ^6^] Much progress has been achieved since then.^[7]^ Not only have [*n*]CPPs of various sizes been synthesized[^8^, ^9^, ^10^, ^11^] and their properties extensively investigated, but also many derivatives containing aromatic units other than benzene.[^12^, ^13^, ^14^] Emerging applications for nanohoops were identified,^[15]^ and their use as segments of carbon nanotubes[^16^, ^17^] as well as in supramolecular chemistry[^18^, ^19^] is a rising field. The incorporation of π‐systems other than benzene allows to alter the optoelectronic and structural properties of nanohoops.^[12]^ This has been demonstrated using donor‐/acceptor‐aromatics or polycyclic aromatic hydrocarbons mostly composed of six‐membered rings.^[12]^ We herein present the dibenzo[*a*,*e*]pentalene (DBP)‐based chiral nanohoops **(+)‐1** and **(−)‐1** consisting of six DBP units and four phenylene rings (Figure 1 A), accessed in a stereoselective synthesis. The DBP units endow ambipolar electrochemical behavior to the hoop,^[20]^ they possess antiaromatic character,[^21^, ^22^] and their lack of a dividing mirror plane when bent makes nanohoop **1** chiral. After rubicene‐containing hoops reported by the Isobe group^[23]^ and [2]DBP[12]CPP nanohoops synthesized by us,^[24]^ this is the third synthetic report incorporating a non‐alternant hydrocarbon into a nanohoop and the second using an antiaromatic unit.^[25]^ The hoop incorporation allows to study the effect of radial conjugation and bending on the antiaromaticity of the DBP units. DBP possesses a small band gap due to an increased HOMO and decreased LUMO energy in comparison to an alternant hydrocarbon of similar size.^[1]^ This causes its ambipolar electrochemical character, making it attractive for, e.g., field‐effect transistors.[^26^, ^27^, ^28^]

Chiral conjugated nanohoops have been synthesized on few occasions by unsymmetrically incorporating polycyclic aromatic hydrocarbons, such as phenanthrene,^[29]^ anthracene,^[30]^ chrysene,[^31^, ^32^] anthanthrene,^[33]^ and rubicene,^[23]^ or through topological design.[^34^, ^35^, ^36^] In most of these cases the hoops were synthesized as racemic or diastereomeric mixtures, and pure stereoisomers were separated by chiral HPLC. This was possible since racemization barriers were high enough to slow down conformational isomerization at room temperature. One example has been reported using a chiral catalyst, which allowed obtaining an enantiomeric excess of a cyclophenylene,^[37]^ and another where an enantioenrichment was obtained for a cyclochrysenylene.^[31]^ We herein present a unique and novel synthetic strategy, which enabled the stereoselective synthesis of **(+)‐1** and **(−)‐1** as one of the first reports of a stereoselective nanohoop synthesis. This was possible by using a novel bent precursor for nanohoop synthesis, as shown in Figure 1 B, a bent and chiral diketone unit, which can be transformed into DBPs once incorporated into the hoop. In the synthesis of dibenzopentalenophanes, we had demonstrated before that this method is suitable to introduce strain and to strongly bend DBP units.^[38]^ Herein we used both enantiomers of this diketone, synthesized by racemic resolution, as the only bent precursor in the synthesis of conjugated nanohoops **(+)‐1** and **(−)‐1**. Bending a preferably planar π‐system into a hoop shape is one of the highest challenges in nanohoop synthesis.^[11]^ Several strategies leading to a bent array of six‐membered rings have been reported in the context of CPP syntheses.[^4^, ^5^, ^6^, ^39^, ^40^, ^41^] Our strategy reported herein adds to this pool and enables the stereoselective synthesis of chiral nanohoops containing antiaromatic and electrochemically ambipolar DBP units.

## Results and Discussion

### Racemic resolution of diketone 3

**Scheme 1:**
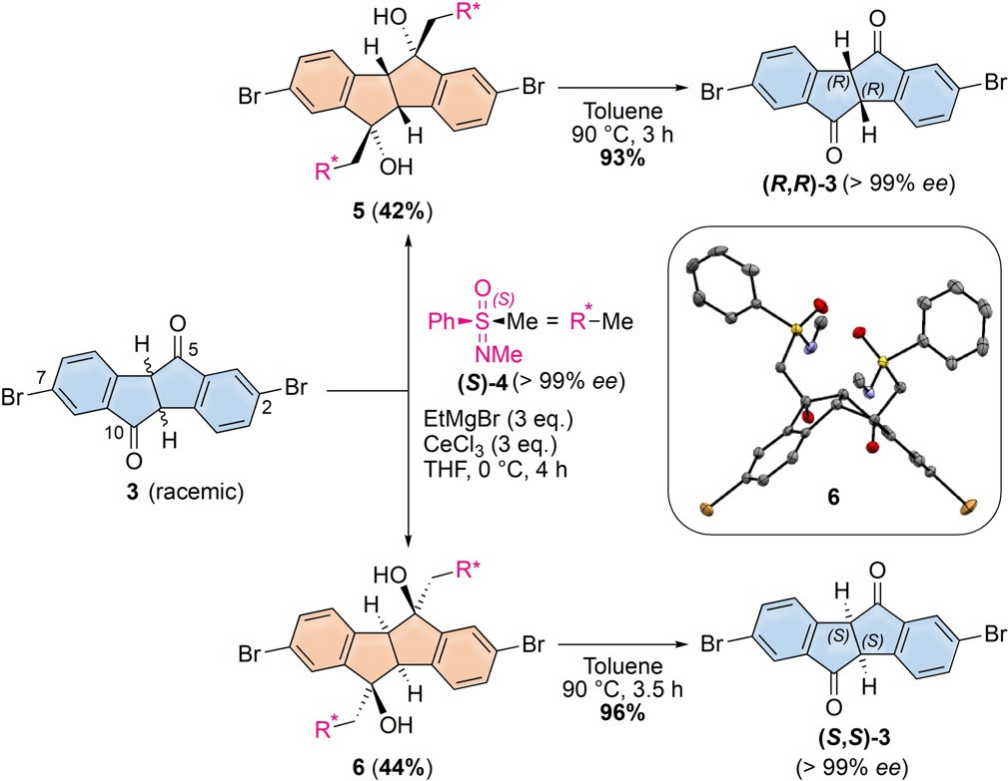
Racemic resolution of 2,7‐dibromo‐diketone **3** and molecular structure of bis‐adduct **6** in the solid state (displacement ellipsoids are shown at 50 % probability; hydrogen atoms and co‐crystallized chloroform molecule are omitted for clarity).

We recently reported on the synthesis of the DBP‐containing nanohoops [2]DBP[12]CPP,^[24]^ accessed using Itami's L‐shaped diphenylcyclohexane units^[42]^ as bent precursors to terphenyl units together with tetrafunctionalized DBPs. In order to synthesize a nanohoop that is chiral and contains a higher ratio of DBP units, we wanted to refrain from using bent terphenylene precursors and instead employ diketone **3**, which possesses a naturally bent structure (107.2° between two six‐membered rings)^[38]^ and is a chiral precursor to DBPs, making it ideally suited to access strained nanohoops. The 2,7‐bromo substituents in **3** would later allow to perform cross‐coupling reactions. Racemic **3** was synthesized in four steps from 2‐(4‐bromophenyl)acetic acid, as previously reported.^[38]^ In initial synthetic attempts to nanohoop **1** (as mixture of stereoisomers) using racemic **3** we faced two main difficulties: (a) Diastereomeric mixtures of intermediates were formed, containing several of the diketone units, which were difficult to purify and structurally characterize, and (b) macrocyclization proceeded with low yield, likely due to the required conformation being hard to achieve in some of the diastereomeric precursors. To avoid these issues we decided to enter the nanohoop synthesis with enantiomerically pure diketone **3** and therefore establish a method for its racemic resolution. While a separation of its enantiomers by analytical chiral HPLC was possible (see SI, Figure S88), the low solubility of **3** in common organic solvents restricted its resolution on a semi‐preparative HPLC scale to <50 mg. For the syntheses envisioned herein to obtain chiral DBP‐based nanohoops, larger amounts were required. The carbonyl functions in **3** provide a useful handle for its transformation into diastereomers, separable on a preparative scale. We initially tested the transformation of **3** into a bisketal using l‐(+)‐diethyl tartrate, its functionalization to a bisimine using (*S*)‐α‐methylbenzylamine or to a bishydrazone using (−)‐menthydrazide.^[43]^ However, in the first two cases functionalization was unsuccessful, and in the third case the diastereomeric hydrazones formed from **3** were inseparable by column chromatography or crystallization. With success, on the other hand, proceeded the sulfoximine‐mediated racemic resolution developed by Johnson,[^44^, ^45^] as shown in Scheme 1. Chiral sulfoximine **(*S*)‐4** was synthesized from thioanisol in three steps including racemic resolution of the intermediate sulfoximine before *N*‐methylation.[^46^, ^47^, ^48^]

After deprotonation with ethyl magnesium bromide, diastereoselective, cerium trichloride‐mediated addition^[49]^ of **(*S*)‐4** to **3** afforded diastereomers **5** and **6** in high yields, which were easily separable (Δ*R*_f_=0.4) on large scale using an automated column chromatography system. The addition of the sulfoximine selectively occurred from the convex side of diketone **3**. This can be seen in the molecular structure of **6** in the solid state, shown in Scheme 1, resolved by X‐ray diffraction on single crystals, grown from chloroform/*n*‐pentane by solvent layering. Thermolysis in toluene allowed removing the chiral auxiliary and furnished **(*R***,***R*** **)‐3** and **(*S***,***S*** **)‐3** in excellent yields and *ee*s. Their absolute configuration was determined through the molecular structures of **6** as well as **(*S***,***S*** **)‐3** (see SI) in the solid state. This method of racemic resolution allowed accessing both enantiomers of **3** in 1.2 g scale.

### Synthesis of DBP‐based nanohoops (+)‐1 and (−)‐1 and reference compound 7

**Scheme 2:**
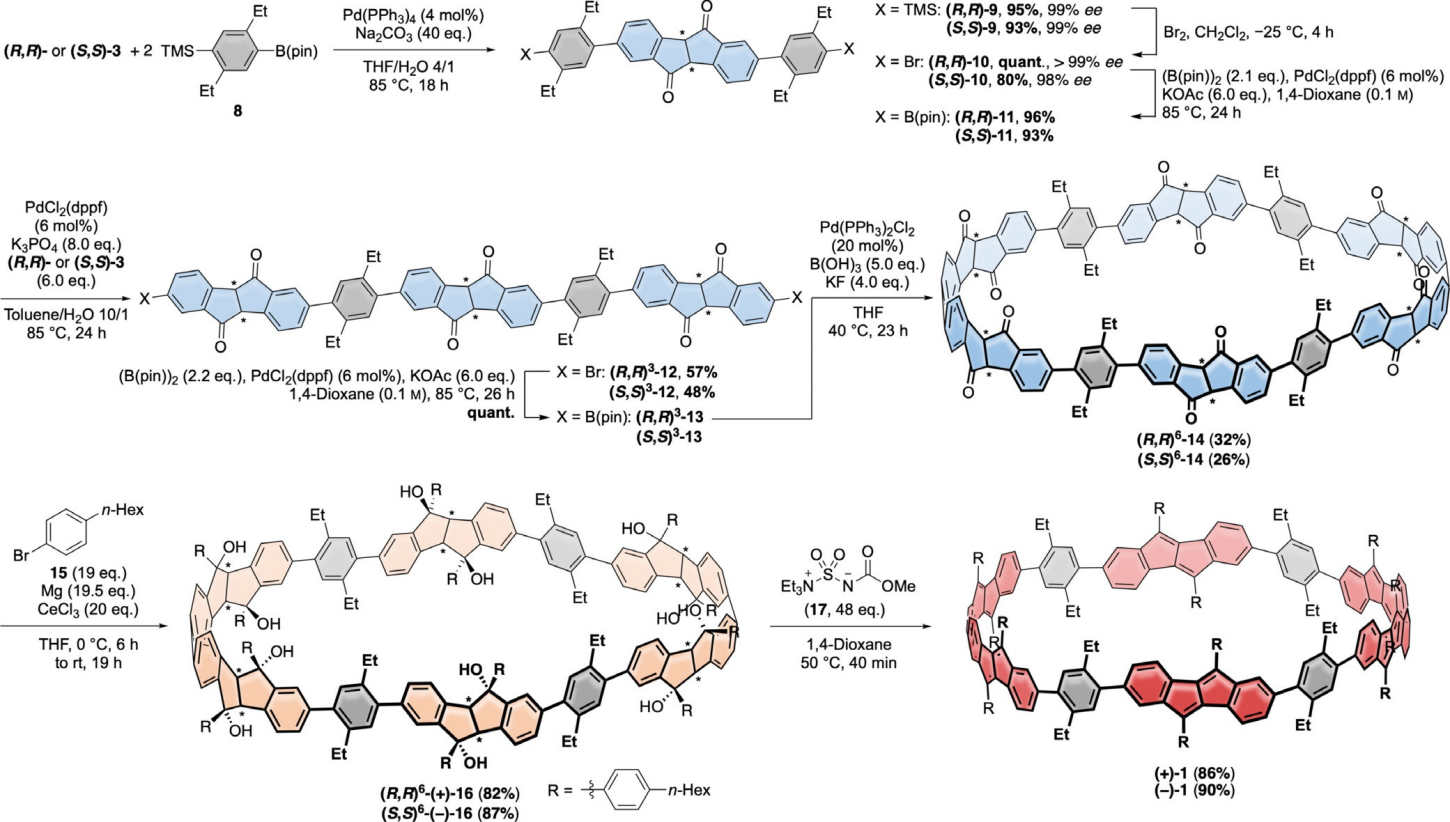
Stereoselective synthesis of the chiral, DBP‐based nanohoops **(+)‐1** and **(−)‐1** (the stereocenters assigned by (*R*) or (*S*) are indicated by an asterisk, for **14**, **16** and **1**, the structures of the (*R*,*R*)^6^/(+)‐isomers are shown).

With sufficiently large amounts of enantiomerically pure diketone **3** in hand, we embarked on the stereoselective nanohoop synthesis using both enantiomers **(*R***,***R*** **)‐3** and **(*S***,***S*** **)‐3** (Scheme 2). From the molecular design we incorporated four diethyl‐substituted phenylene groups in total into the hoop as spacers between DBP units, since preliminary investigations had shown that a linear trimer of **3** (in analogy to **12** without the two bridging arylene units) was too insoluble for further functionalization. Suzuki–Miyaura‐coupling of boronic ester **8** (for synthesis see SI) with **(*R***,***R*** **)‐3** or **(*S***,***S*** **)‐3** afforded **(*R***,***R*** **)‐** and **(*S***,***S*** **)‐9** in excellent yields of 95 % and 93 %, respectively, which were then transformed into dibromides **(*R***,***R*** **)‐** and **(*S***,***S*** **)‐10**. The following Miyaura‐borylation furnished **(*R***,***R*** **)‐** and **(*S***,***S*** **)‐11** in excellent yields of 96 % and 93 %, respectively. **(*R***,***R*** **)‐** and **(*S***,***S*** **)‐11** were then cross‐coupled with an excess of **(*R***,***R*** **)‐** and **(*S***,***S*** **)‐3**, respectively, to give **(*R***,***R*** **)^3^‐** and **(*S***,***S*** **)^3^**–**12** in good yields of 57 % respective 48 % (the superscript ^3^ indicates the presence of three diketone units with the respective (*R*,*R*)‐ or (*S*,*S*)‐configuration). The surplus of **(*R***,***R*** **)‐** or and **(*S***,***S*** **)‐3** employed in this step could be recovered during purification of the product. Quantitative Miyaura‐borylation of **(*R***,***R*** **)^3^‐** and **(*S***,***S*** **)^3^‐12** led to **(*R***,***R*** **)^3^‐** and **(*S***,***S*** **)^3^‐13**, which were the compounds required to perform the macrocyclization. Due to its success in the synthesis of strained [*n*]CPPs, we chose Jasti's oxidative homocoupling of boronic esters as the hoop‐forming reaction.^[50]^ Subjecting **(*R***,***R*** **)^3^‐** and **(*S***,***S*** **)^3^‐13** to these conditions furnished the dimeric hoops **(*R***,***R*** **)^6^‐** and **(*S***,***S*** **)^6^‐14** in respectable yields of 32 % and 26 %, respectively, which could be purified by column chromatography. Interestingly, the smaller hoops, resulting from an intramolecular cyclization of **(*R***,***R*** **)^3^‐** and **(*S***,***S*** **)^3^‐13**, were not detected. This must have been due to the conformation of the open precursors **(*R***,***R*** **)^3^‐** and **(*S***,***S*** **)^3^‐13**, which can take on a U‐shape, ideally suited for a cyclodimerization (for calculated structure of **(*R***,***R*** **)^3^‐13** see SI, Figure S124), but not an intramolecular reaction. Larger oligomers were also formed (as indicated by analytical GPC, see SI, Figure S83), but could not be isolated in pure form. In the last two steps we converted all six diketone units in **14** into DBPs. 4‐(*n*‐Hexyl)phenyl substituents to the DBPs were chosen to impart sufficient solubility to both **16** and **1**. Cerium trichloride‐mediated addition[^38^, ^49^] of the Grignard‐reagent of **15** (for synthesis see SI) afforded dodecaols **(*R***,***R*** **)^6^‐(+)‐** and **(*S***,***S*** **)^6^‐(−)‐16** in high yields of 82 % and 87 %, respectively. This corresponds to a yield of 98 % respective 99 % for the individual addition to each of the twelve carbonyl groups. The last critical step was the 12‐fold water elimination to furnish conjugated hoops **1**. We had previously optimized this step in the synthesis of strained DBP‐phanes and found Burgess's reagent (**17**) to be the mild method of choice.^[38]^ The same procedure worked cleanly for **(*R***,***R*** **)^6^‐(+)‐** and **(*S***,***S*** **)^6^‐(−)‐16** and gave DBP‐based chiral hoops **(+)‐1** and **(−)‐1** in high yields of 86 % and 90 %, respectively. As for the previous step, this corresponds to impressive yields of 99 % for each individual water elimination step. The identity and purity of **(+)‐1** and **(−)‐1** as well as cyclic precursors **(*R***,***R*** **)^6^‐** and **(*S***,***S*** **)^6^‐14** and **(*R***,***R*** **)^6^‐(+)‐** and **(*S***,***S*** **)^6^‐(−)‐16** was confirmed by 1D‐ and 2D‐NMR spectroscopy (^1^H, ^13^C, COSY, HSQC, for **(+)‐1** also ROESY) and HRMS.

**Scheme 3:**
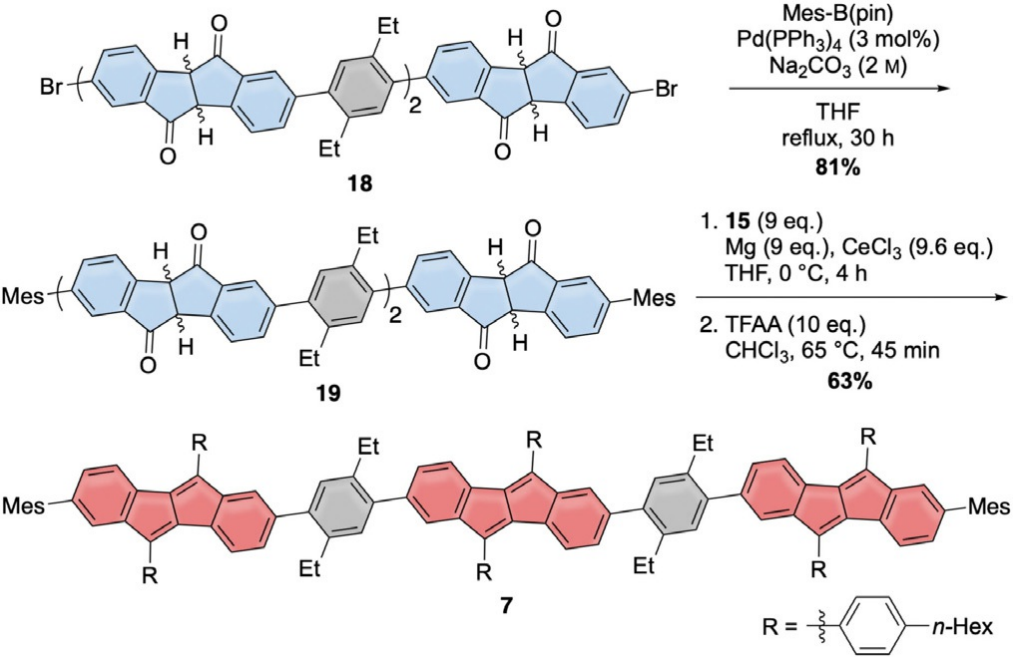
Synthesis of reference compound **7**.

For comparison of the optoelectronic properties we synthesized **7**, representing a planar reference compound for nanohoop **1** (Scheme 3). Its synthesis started from **18**, which was obtained in analogy to **12** (Scheme 2), but using racemic **3** as starting material (see SI for details). Mesityl groups were attached to the two outer diketone rings in **18** using a Suzuki–Miyaura‐coupling reaction to furnish **19**. Cerium trichloride‐mediated addition of the Grignard‐reagent of **15** to the diketone units in **19** followed by acid‐catalyzed hexafold water elimination afforded reference compound **7** in good yield of 63 %.

### Molecular structure of DBP‐based nanohoops (+)‐1 and (−)‐1

**Figure 2:**
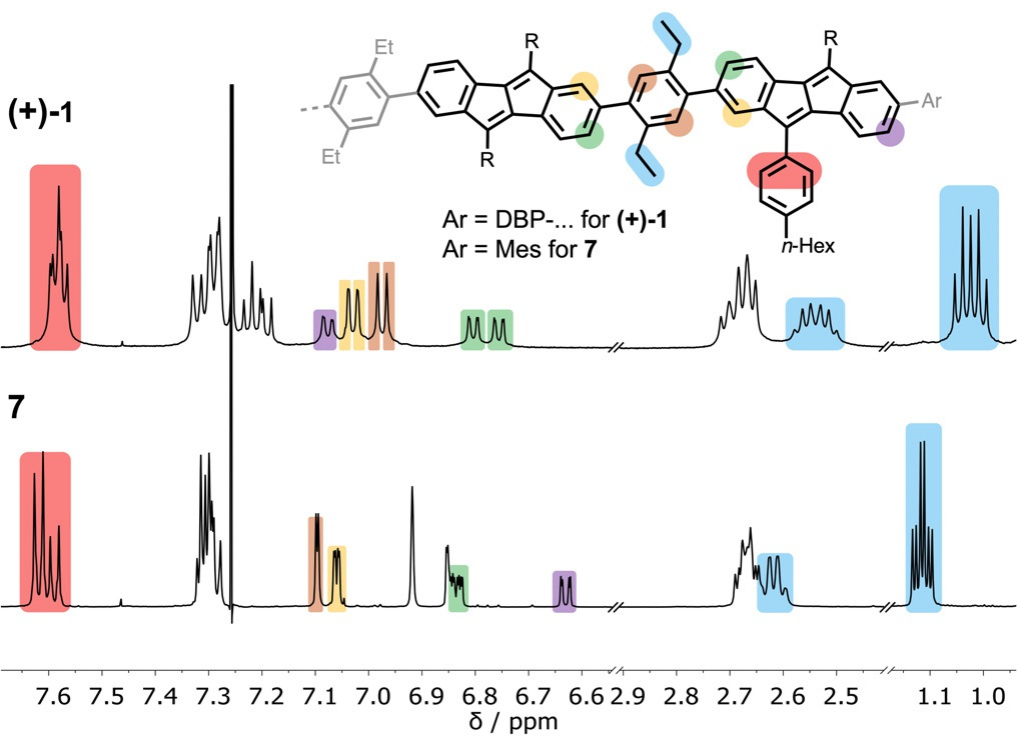
Selected regions of the ^1^H NMR spectra of hoop **(+)‐1** and reference compound **7** (CDCl_3_, 500 MHz, 300 K).

Due to their mirror‐symmetric structure as enantiomers, the NMR spectra of hoops **(+)‐1** and **(−)‐1** were identical (see SI). Comparison of the ^1^H NMR spectra of nanohoop **(+)‐1** with planar reference compound **7** confirmed the high symmetry and more rigid structure of the hoop (Figure 2, some protons were assigned for comparison, for full assignment see SI). The doublets of doublets (highlighted in purple) for the DBP units, located next to the mesityl groups in **7** and next to a neighboring DBP unit in hoop **(+)‐1**, were shifted downfield by about 0.5 ppm in the hoop compared to planar **7**. The DBP protons highlighted in green were more differentiated in hoop **(+)‐1** compared to reference compound **7** due to the higher rigidity of the hoop, the same held for the DBP protons highlighted in yellow. A stronger splitting was also observed for the ethyl protons (highlighted in blue) in **(+)‐1** compared to **7**. A VT‐NMR experiment did not reveal significant changes in signal width or splitting with increasing temperature (see SI).

To evaluate the structural properties of **1** by DFT calculations all alkyl groups were replaced by methyl groups, furnishing **2‐1** (its stereochemistry corresponding to the *D*_2_‐symmetric‐enantiomer drawn in Figure 1 or **1 a** in Figure 4). The geometry of **2‐1** was initially assessed using molecular dynamics (MD) simulations (with the OPLS3 Force Field as implemented in Schrodinger 2017), followed by geometry optimization at the PBEh‐3c^[51]^‐level of theory (see SI for details).

**Figure 3:**
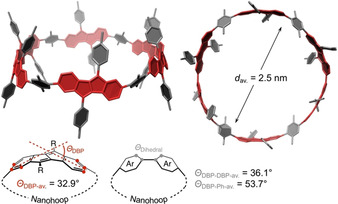
Calculated structure of **2‐1** (PBEh‐3c, H‐atoms are omitted for clarity) and geometrical parameters.

The calculated structure of **2‐1** shows its near *D*_2_ symmetry and circular geometry (Figure 3). Its diameter averages to 2.5 nm, which makes [6]DBP[4]CPP‐hoop **2‐1** slightly larger than the [2]DBP[12]CPP‐hoop recently reported by our group with 2.1 nm diameter.^[24]^ The DBP units are bent by *Θ*_DBP_=32.9° on average, which is slightly less than in the [2]DBP[12]CPP‐hoop mentioned above and significantly less than the bends of up to *Θ*_DBP_=91.9° reported by our group for DBP‐phanes.^[38]^ In comparison, the four phenylene units in **2‐1** experience a bend of only 7.1°, which is lower than that in [18]CPP of 8.0° with similar diameter.^[52]^ While the phenylene units are rotated more strongly out of the hoop shape relative to the DBP units (dihedral angle *Θ*_DBP‐Ph‐av._=53.7°), the dihedral angles between two DBP units are comparably small (*Θ*_DBP‐DBP‐av._=36.1°). The calculated strain energy of **2‐1** amounts to 44.8 kcal mol^−1^ (see SI for homodesmotic equation used). This is larger than in the slightly smaller [2]DBP[12]CPP‐hoop (36.5 kcal mol^−1^) reported by us^[24]^ or in [18]CPP of similar size (31.7 kcal mol^−1^).^[52]^ The reason may lie in the larger dihedral angles between the DBP and phenylene units in **2‐1** of 54° due to the *ortho*‐substituents on the latter compared to large [*n*]CPPs, where the dihedral angles amount to 36° on average. This implies a lower conjugation and hence lower conjugative stabilization in **2‐1**, responsible for its relatively high strain energy.

### Stereoisomerism in DBP‐based nanohoops (+)‐1 and (−)‐1

**Figure 4:**
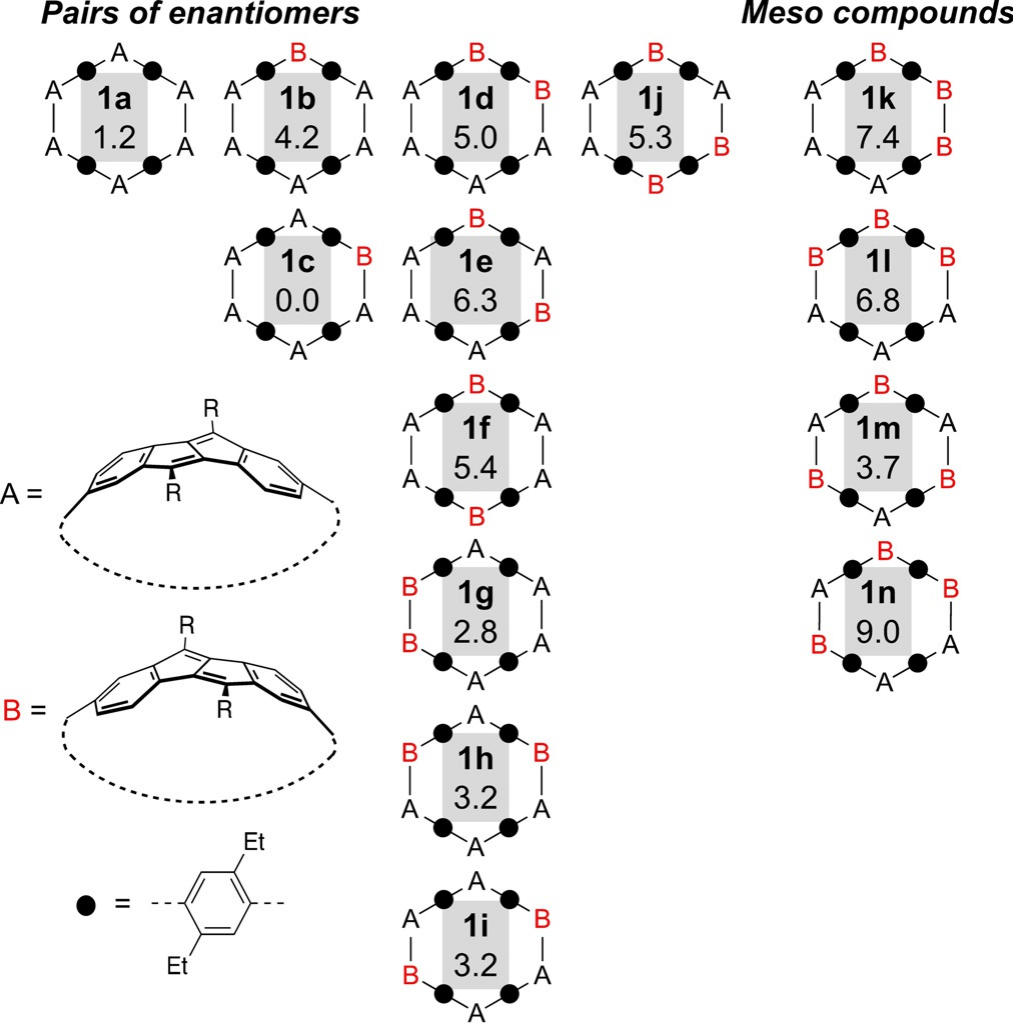
14 possible diastereomers **1 a**–**1 n** of hoop **1** with relative energies in kcal mol^−1^ (B97‐3c//GFN2‐xTB; for the pairs of enantiomers, one enantiomer each is shown).

We next assessed the stereoisomerism in **1**. In theory, 14 diastereomers **1 a**–**1 n** are possible by rotation of one or several DBP units through the hoop (A or B conformation, Figure 4), of which ten are pairs of enantiomers and four are meso compounds. In the most symmetric (*D*_2_‐symmetric) enantiomer **1 a**, all DBP units are oriented the same way (A conformation). Its enantiomer (all DBPs in B conformation) would be 1 a*. 1 a is also the conformation we depicted in Figure 1 and Scheme 2 for simplicity.

DFT calculations provided insight into the relative energies and interconversion barriers of the 14 diastereomers **1 a**–**1 n** shown in Figure 4. The structures of **1 a**–**1 n** were optimized using the semiempirical extended tight‐binding model GFN2‐xTB^[53]^ with tight convergence criteria and applying the implicit solvation model GBSA^[54]^ with toluene solvent. This method is particularly well‐suited to explore the conformational space of molecular systems. B97‐3c^[55]^ DFT single‐point energies were computed on all optimized structures, applying the implicit solvent model D‐COSMO‐RS^[56]^ with toluene solvent. With 9.0 kcal mol^−1^ (maximum energy for **1 n**) the energy range is substantial, and **1 a** as the *D*_2_‐symmetrical isomer as well as **1 c** with one DBP unit rotated relative to the others are the most stable diastereomers with relative energies of 1.2 and 0.0 kcal mol^−1^, respectively. Energetic barriers for the rotation of the two symmetrically inequivalent DBP units were estimated at the B97‐3c(toluene)//GFN2‐xTB(gas phase) level of theory using a simplified molecular structure of the hoop, namely **2‐2** (Figure 1). In **2‐2** the 4‐(*n*‐hexyl)‐phenyl substituents R on the DBP units were replaced by H atoms to allow for a transition state calculation, since the actual hoop was too large for such a calculation. The calculated values for one DBP rotation amounted to 8.9 and 10.8 kcal mol^−1^, and they thus mostly reflect the dihedral strain occurring through rotation of the DBP units with respect to the neighboring aryl or DBP rings. Using these estimated values in two possible pathways for complete racemization of hoop **2‐2 a** to its enantiomer 2‐2 a* (see SI, Figure S132), in which all six DBP units have to rotate, provided estimated values of 16 or 18 kcal mol^−1^ for the simplified molecular structure of the hoop **2‐2**. In the actual hoop **1**, including all hexylphenyl substituents R on the DBP units, both energetic as well as entropic contributions would significantly increase the rotational (free energy) barriers for the DBP units. Interestingly, a molecular dynamics simulation of the full system **1 a** at 400 K at the GFN2‐xTB/GBSA(toluene) level of theory showed that while the hoop is conformationally highly flexible and its shape changes from circular to oval, no rotation of a DBP unit through the hoop occurred. This can be seen in the animation, provided as additional material online. In comparison, the same calculation with highest energy conformer **1 n** (relative energy 9 kcal mol^−1^) led to almost a complete rotation of one DBP unit to furnish a lower energy conformer (see animation as additional material).

**Figure 5:**
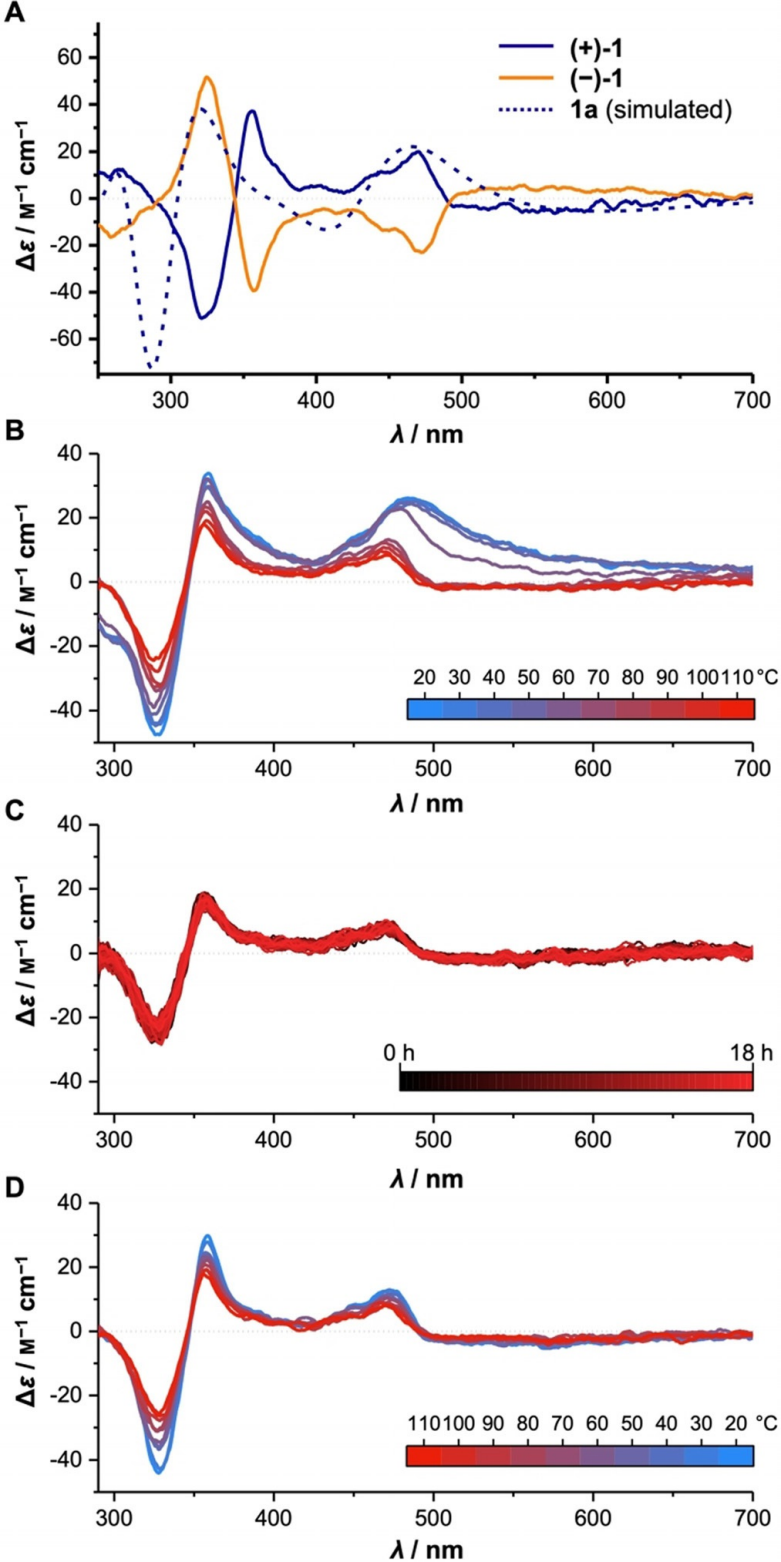
A) Electronic circular dichroism (ECD) spectra of **(+)‐1** and **(−)‐1** (CHCl_3_, 20 °C) with spectrum simulated for enantiomer **1 a** (sTDA‐xTB level out of 100 snapshots of a GFN2‐xTB/GBSA(toluene) MD‐simulation of 100 ps length); B–D) ECD‐spectra of **(+)‐1** (3.34×10^−5^ m in PhCl) during (B) heating at a rate of 2 °C min^−1^, (C) holding the temperature at 110 °C and (D) cooling at a rate of 2 °C min^−1^.

**Figure 6:**
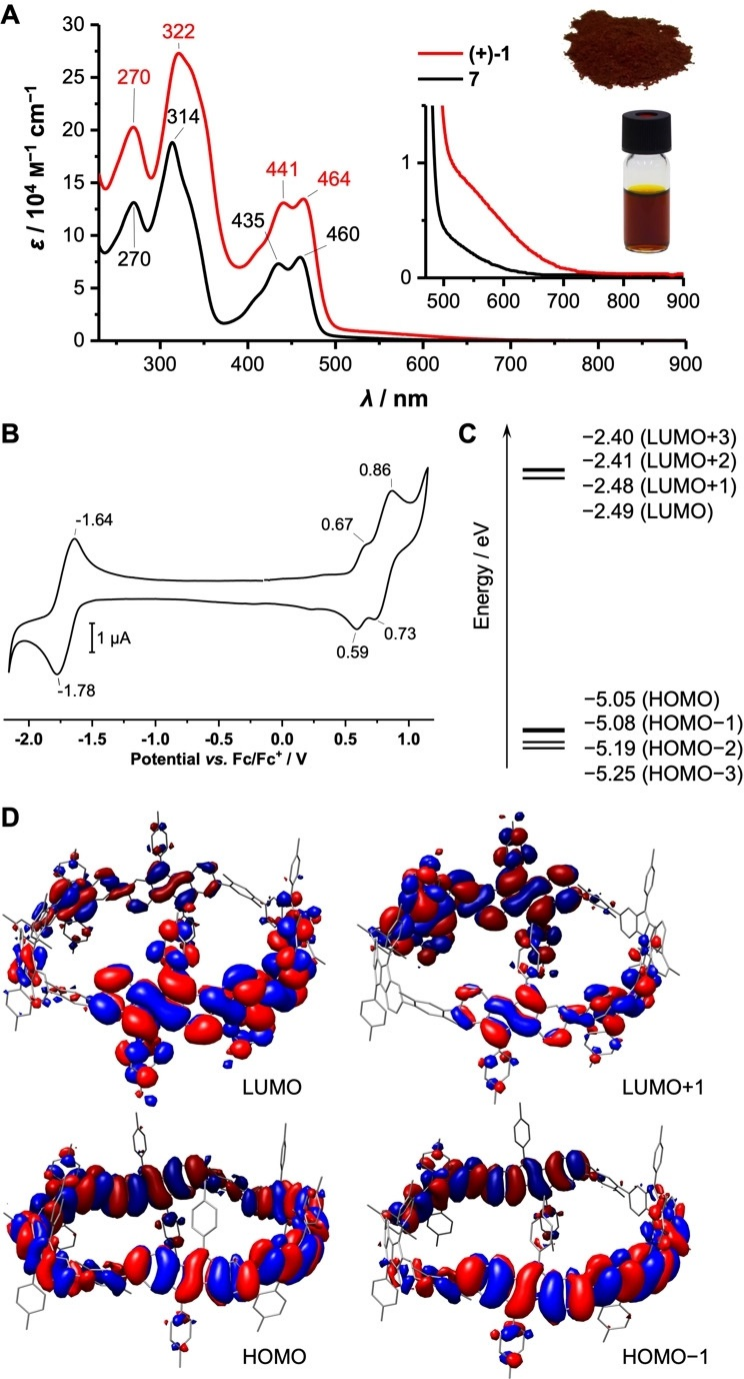
A) UV/Vis absorption spectra of **(+)‐1** and reference compound **7** (CH_2_Cl_2_) and photos of **(+)‐1** as powder and in solution; B) cyclic voltammogram of **(+)‐1** (0.16 mm in CH_2_Cl_2_ with 0.1 m *n*‐Bu_4_NPF_6_, scan rate 0.1 V s^−1^, glassy carbon electrode); C) Selected orbital energies and D) plots for **2** (B3LYP‐D3/def2‐TZVP//PBEh‐3c).

We next turned to optical rotation and electronic circular dichroism (ECD) measurements to assess the chirality of the synthesized hoops **(+)‐1** or **(−)‐1**. The ECD spectra for **(+)‐1** or **(−)‐1** possess a mirror image relationship, confirming their enantiomeric relationship (Figure 5 A). The ECD spectrum of **(+)‐1** shows a positive Cotton effect between 500–350 nm followed by a negative Cotton effect from 350–300 (Figure 5 A). In addition, a negative Cotton effect appears between 700–500 nm, corresponding to the HOMO–LUMO transition of the DBP units, which is symmetry‐forbidden and therefore weak (see also below, discussion of the UV/Vis spectra). The signals correspond well to the UV/Vis absorption spectrum of **(+)‐1** shown in Figure 6 A (an overlay of both spectra can be found in the SI, Figure S111). The ECD spectrum of **(−)‐1** shows the opposite signs for the Cotton effects at the same wavelengths as in **(+)‐1** (Figure 5 A), confirming its mirror‐symmetric structure. To potentially assign the ECD‐spectrum of **(+)‐1** to one or several of the stereoisomers **1 a**–**1 n** (see Figure 4), we simulated ECD spectra for all 14 stereoisomers (see SI, Figure S115). Chiral hoops **1 a**–**1 j** showed Cotton effects of the same signs at similar wavelengths, only with different intensities, depending on the number of DBP units in each orientation (A or B in Figure 4). **1 a** with all DBP units oriented the same way had the highest intensities, followed by **1 b** and **1 c** with one unit rotated, and **1 d**–**1 i** as well as **1 j** had the lowest calculated intensities of the CD‐spectroscopic bands. Exemplarily, the simulated spectrum for **1 a** is shown in Figure 5 A. It corresponds well in shape to the experimental ECD‐spectrum of **(+)‐1**. Measurement of specific optical rotation for the DBP hoops gave [α]25D values of opposite sign with +5.4° (*c=*0.44, CHCl_3_) for **(+)‐1** and −2.4° (*c=*0.42, CHCl_3_) for **(−)‐1**. These rotation values are low and at the instrumental detection limit (see SI), but their opposite sign confirms the mirror image geometry for both hoops.

ECD‐spectra were also measured for linear hexaones **(*R***,***R*** **)^3^‐12** and **(*S***,***S*** **)^3^‐12**, cyclic dodecaones **(*R***,***R*** **)^6^‐14** and **(*S***,***S*** **)^6^‐14** and cyclic dodecaols **(*R***,***R*** **)^6^‐(+)‐16** and **(*S***,***S*** **)^6^‐(−)‐16**, displaying strong Cotton effects and mirror image relationships for each of the respective pairs of enantiomers. These can be found in the SI. **(*R***,***R*** **)^6^‐(+)‐16** and **(*S***,***S*** **)^6^‐(−)‐16** provided specific optical rotation values of [α]25D =+82.6° (*c=*1.03, CHCl_3_) and −76.8° (*c=*0.98, CHCl_3_), respectively. These are significantly larger than in **(+)‐1** or **(−)‐1** likely due to the presence of discrete stereocenters in **16**. However, a quantitative evaluation of optical rotation values in different molecules is not possible.

We performed temperature‐dependent ECD spectra to gain insight into the conformational behavior of hoop **(+)‐1** (Figure 5 B–D). We chose chlorobenzene as solvent for these experiments, since it allowed accessing higher temperatures. The ECD spectrum of **(+)‐1** in chlorobenzene at 20 °C (Figure 5 B, blue line) slightly differs to that in chloroform (Figure 5 A) in that a longer wavelength shoulder around 530 nm is present. To investigate the temperature‐dependence of the ECD‐signals, we heated the sample from 20 to 110 °C (at a rate of 2 °C per min) and recorded ECD spectra at regular intervals (Figure 5 B). All spectra in Figure 5 are UV/Vis‐corrected to rule out concentration effects. With increasing temperature, the intensities of all bands slightly decreased. The largest change happened at around 60 °C, where in particular the shoulder around 530 nm disappeared. Upon further heating, the signal intensity further decreased for all bands. We then held the temperature at 110 °C for 18 h, during which time no further change occurred (Figure 5 C). Lastly, the solution was again cooled to rt, whereby the intensities of the CD bands slightly increased again (Figure 5 D). However, the shoulder band at 530 nm did not reappear. These results show that optical activity of **(+)‐1** is not lost, even after prolonged heating to 110 °C, and complete racemization did not occur. The temperature‐dependent changes we observed in the ECD spectra of **(+)‐1** were mostly reversible, with the exception of the shoulder at 530 nm. This result is in line with the MD simulations mentioned above, which showed that in **1 a** at a simulated temperature of 400 K none of the DBP units rotated. Simulation of the ECD spectrum of **1 a** at 400 K confirmed a slightly lower intensity of the bands compared to 300 K (see SI, Figure S116). It may be that higher temperatures are required to observe a racemization of the hoop, however, the CD spectrometer we used only allowed heating to 110 °C as the highest temperature.

### Electronic structure of DBP‐based nanohoop 1

The optoelectronic properties of hoop **1** are dominated by the DBP units and reflect its ambipolar character. The UV/Vis absorption spectra of **(+)‐1** and reference compound **7** showed several bands, characteristic for substituted DBPs (Figure 6 A).[^27^, ^38^, ^49^, ^57^] Most constitute transitions involving several orbitals, as we previously assigned for small molecule DBP derivatives using TDDFT calculations.[^49^, ^57^] Electronically, the DBP units in hoop **1** are intermediate between 2,7‐^[49]^ and 5,10‐arylalkinyl‐substituted DBPs,^[57]^ since the bands between 400–500 nm are relatively large in comparison to the absorption maximum at 322 nm due to significant conjugation to the 5,10‐aryl substituents. The slight bathochromic shift of all bands in hoop **(+)‐1** compared to reference compound **7** indicates a stronger conjugation in the hoop. Most noteworthy is the lowest energy band, corresponding to the HOMO → LUMO single excitation and well visible in the inset in Figure 6 A. This transition is forbidden in planar and centrosymmetric DBP derivatives, where both orbitals are of a_u_ symmetry (Laporte's rule). Since the DBP units in hoop **1** deviate more strongly from planarity than in planar **7**, this shoulder at 500–600 nm had a significantly higher intensity for the hoop. The optical band gap of **(+)‐1** amounted to 1.66 eV. This value is strongly bathochromically shifted compared to reference compound **7** with 1.87 eV, to [2]DBP[12]CPP hoop recently reported by us with 1.83 eV^[24]^ as well as to small molecule DBP derivatives^[57]^ and indicates strong conjugation around the hoop. Nanohoop **(+)‐1** showed no fluorescence, which has been observed for other DBP derivatives.[^49^, ^57^]

The cyclic voltammogram of **(+)‐1** demonstrates its ambipolar electrochemical character due to the DBP units^[20]^ (Figure 6 B). A reversible reduction occurred at a half‐wave potential of *E*_1/2_=−1.71 V, and two quasi‐reversible oxidations appeared at *E*_1/2_=0.63 and 0.80 V (all vs. Fc/Fc^+^). Compared to the [2]DBP[12]CPP hoop recently reported by our group and a 2,5,7,10‐tetramesityl‐substituted DBP^[24]^ both reduction and oxidation were facilitated (shifted to higher respective lower absolute potential) in **(+)‐1**. For reference compound **7** the first oxidation occurred at a similar potential as in the hoop (measured in CHCl_3_ for solubility reasons, see SI for CV). Based on this and the UV/Vis data, the HOMO[^58^, ^59^] and LUMO^[60]^ energies for **(+)‐1** were estimated to −5.35 eV and −3.69 eV, respectively.

The calculated frontier molecular orbitals of **2‐1** are distributed over several DBP units (Figure 6 D and SI for images of other orbitals). From the LUMO up to LUMO+5 and the HOMO down to HOMO–11, these orbitals lie very close in energy (Figure 6 C, Δ*E* ≤0.15 eV). Hence the redox events visible in Figure 6 B could be processes involving more than one electron, which could explain the higher current for the reduction and second oxidation compared to the first oxidation.

NICS (nucleus independent chemical shift) values^[61]^ provided information on the (anti)aromatic character of the DBP units in **2‐1**. NICS(1)_iso_ above/below the five‐ and six‐membered rings amounted to average values of 4.6 and −6.3, respectively (calculated with the GIAO method on the B3LYP/6‐31G* level of theory). A comparison with the NICS(1) values for the unsubstituted DBP of 5.9 for the five‐ and −6.2 for the six‐membered ring^[62]^ shows that the antiaromaticity in the central pentalene unit in hoop **2‐1** is slightly reduced while the aromaticity of the six‐membered rings remains unaffected.

### Complexation of fullerene‐C_60_ by (+)‐1

**Figure 7:**
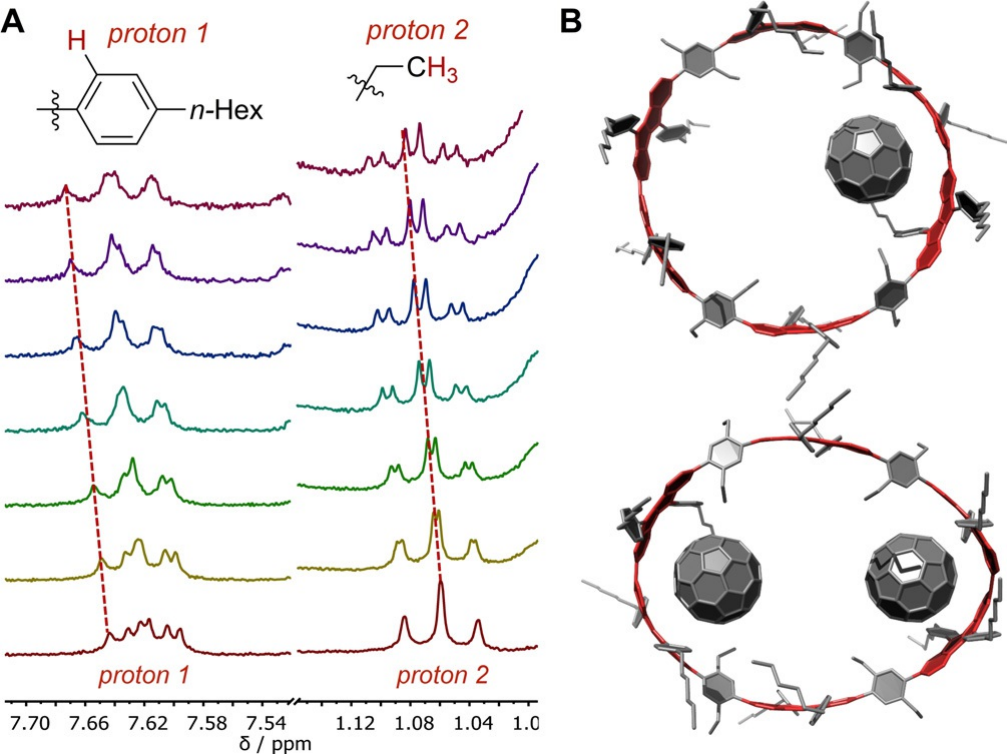
A) Selected regions of ^1^H NMR spectra during titration of **(+)‐1** with C_60_ (0 to 12 equiv. from bottom to top, [D_8_]toluene, 300 MHz, 300 K); B) Calculated equilibrium structures of **1**⊃C_60_ (GFN2‐xTB/GBSA (toluene)).

Due to their hoop shape and large π‐surface, conjugated nanohoops are well‐suited to bind guest molecules, such as fullerenes. A strong binding of C_60_ (diameter 0.71 nm^[63]^) by nanohoops has been reported on several instances[^18^, ^19^] for hoops with diameters of 1.3–1.5 nm, such as [10]CPP.^[64]^ With 2.5 nm, the diameter of hoop **1** (calculated for **2‐1**) in spherical shape significantly exceeds these values, however, our initial MD simulations indicated the hoop structure to be somewhat flexible. Indeed, NMR titration experiments^[65]^ performed in triplicate with C_60_ in [D_8_]toluene showed a shift in some of the ^1^H NMR‐resonances with increasing concentration of C_60_ (Figure 7 A). The spectra indicated a fast exchange between free and complexed species, as only one set of signals was visible. To obtain binding constants, we used hoop **(+)‐1** and a 1:2 (**(+)‐1**:C_60_) binding model,[^66^, ^67^, ^68^] which considered the formation of 1:1 and 1:2 complexes. This model provided a better fit of the experimental data using nonlinear regression and an online tool^[69]^ than a 1:1 model. The resulting binding constants were *K*_11_=(5.4±0.7)×10^3^ m ^−1^ and *K*_12_=(1.1±1.0)×10^2^ m ^−1^ for the complex formation between hoop **(+)‐1** and one respective two C_60_ molecules. These values lie three to four orders of magnitude below association constants with C_60_ reported for smaller nanohoops, such as [10]CPP, due to the large size of **(+)‐1**. In the statistical case a ratio of 4:1 between *K*_11_ and *K*_12_ would be expected;^[66]^ hence our results indicate an anti‐cooperative situation for the binding of the second fullerene molecule. This is somewhat surprising, since both C_60_ molecules are well accommodated within the hoop, and only a small distortion of the latter is required (see Figure 7 B).

DFT calculations provided insight into the molecular structures of the 1:1‐ and 1:2‐complexes (Figure 7 B). Structures were optimized at the GFN2‐xTB level of theory, as described above. The nearest distances between C_60_ and the DBP and phenylene units amount to 3.24 Å on average in the 1:1 complex, which is close to double the van‐der‐Waals radius of carbon (1.7 Å).

The calculations also furnished association free energies consisting of the following contributions [Eq. 1]:

(1) $$\Delta G=\Delta G{}_{\mathrm{RRHO}}+\Delta E+\Delta \delta G{}_{\mathrm{solv}},$$

where Δ*G*_RRHO_ is the thermostatistical contribution in the rigid‐rotor‐harmonic‐oscillator approximation calculated with GFN‐FF,^[70]^ Δ*E* is the gas phase association energy and δ*G*_solv_ the solvation free energy of each species, the sum of the latter two terms calculated at the GFN2‐xTB level of theory. The association free energy for the complexation of the first C_60_ molecule by **(+)‐1** of −5.3 kcal mol^−1^ matches very well with the experimental value of −5.1 kcal mol^−1^, while binding of the second C_60_ molecule is slightly stronger in the calculation (−6.5 kcal mol^−1^) compared to the experiment (−3.2 kcal mol^−1^).

## Conclusion

We herein reported the stereoselective synthesis of two enantiomers of the chiral conjugated nanohoop **1**. These hoops contain six dibenzo[*a*,*e*]pentalene (DBP) and four arylene units. Stereoselectivity was achieved by using bent and chiral diketone precursors to DBP units in enantiomerically pure form, thereby introducing a new bent “corner” unit for nanohoop synthesis. Electronic circular dichroism spectra and MD simulations showed that—inspite of its conformational flexibility regarding the outer shape—hoop **(+)‐1** did not racemize even when heated to 110 °C. The antiaromaticity of the DBP units was reflected in the ambipolar electrochemical properties of the hoop allowing for a reversible reduction and two quasi‐reversible oxidations. Due to its large size of 2.5 nm the [6]DBP[4]Ph‐hoop could accommodate up to two fullerene‐C_60_ molecules, shown through NMR‐based binding studies and DFT calculations.

## Conflict of interest

The authors declare no conflict of interest.

## Supporting information

As a service to our authors and readers, this journal provides supporting information supplied by the authors. Such materials are peer reviewed and may be re‐organized for online delivery, but are not copy‐edited or typeset. Technical support issues arising from supporting information (other than missing files) should be addressed to the authors.

Supplementary

Supplementary

Supplementary

Supplementary
