# Supplementary material for: Chiral Dibenzopentalene‐Based Conjugated Nanohoops through Stereoselective Synthesis
Source: Angew Chem Int Ed Engl. 2021 Mar 23;60(19):10680–9. doi: 10.1002/anie.202016968 (PMC8252646; doi:10.1002/anie.202016968)
Supplement: Supplementary file 1 — Supplementary [file ANIE-60-10680-s003.pdf]

Angewandte Chemie
Eine Zeitschrift der Gesellschaft Deutscher Chemiker

Supporting Information

Chiral Dibenzopentalene-Based Conjugated Nanohoops through Stereoselective Synthesis

*Mathias Hermann, Daniel Wassy, Julia Kohn, Philipp Seitz, Martin U. Betschart, Stefan Grimme, and Birgit Esser**

anie_202016968_sm_miscellaneous_information.pdf

Contents

1. Materials and Methods 2
2. Synthetic Manipulations 5
   2.1 Synthesis of Chiral Auxiliary **(*S*)-4** and Chiral Resolution of **3** 6
   2.2 Synthesis of Racemic Reference Compound **7** 11
   2.3 Synthesis of Enantiomerically Pure Acyclic Nanohoop Precursors 17
   2.4 Synthesis of Cyclic Compounds 22
3. NMR Spectra 28
   3.1 NMR Spectra 28
   3.2 VT NMR Spectra of **(+)-1** 77
   3.3 NMR Assignment of **(+)-1** 78
4. ATR FT-IR Spectra 80
6. GPC Elugrams 84
7. HPLC Elugrams 85
8. Cyclic Voltammograms 93
9. Absorption Spectroscopy 94
   9.1 UV/Vis Absorption Spectra 94
   9.2 Electronic Circular Dichroism Spectra 95
      9.2.1 ECD spectra of chiral precursor compounds 95
      9.2.2 Temperature-dependent ECD-spectroscopic measurements 96
      9.2.3 Calculated ECD spectra 98
10. Mass Spectra 100
11. Single-Crystal X-Ray Diffraction 105
   11.1 General Experimental Setup 105
   11.2 Solution and Refinement 105
12. NMR Titration Studies of **1** with Fullerene-C_{60} 108
13. DFT Calculations 110
   13.1 Calculation of the Ring Strain 110
   13.2 Calculation of the Nucleus Independent Chemical Shift (NICS) Values 111
   13.3 Calculated Structure of U-Shaped Acyclic precursor **(*R*,*R*)^3-12** 112
   13.4 Conformational Energies 112
   13.5 Rotational Barriers of the DBP Units in Hoop **1** 114
   13.6 Association Free Energies ΔG for the Fullerene Complexation by **1** 117
   13.7 Cartesian Coordinates of Calculated Structures 118
14. References 123

1. Materials and Methods

Chemicals were purchased from ABCR, ACROS-ORGANICS, ALFA-AESAR, CHEMPUR, FLUROCHEM, ROTH, SIGMA-ALDRICH or TCI and used directly without further purification unless otherwise noted. Moisture- or oxygen-sensitive reactions were carried out in dried glassware, heated under vacuum (10^{-3} mbar), using standard Schlenk techniques in a dry argon atmosphere (Argon 5.0 from SAUERSTOFFWERKE FRIEDRICHSHAFEN). Anhydrous solvents (CH_2Cl_2, THF) were obtained from an M. BRAUN solvent purification system (MB-SPS-800) and stored over molecular sieves (3 Å). Anhydrous acetone was purchased from ACROS-ORGANICS (extra dry, < 50 ppm H_2O, AcroSeal™) and further stored over activated molecular sieves (3 Å). Other anhydrous solvents were obtained by drying over activated molecular sieves (3 Å) for several days.[1] Cyclohexane for flash chromatography was purchased in technical grade and purified by distillation using a rotary evaporator. Other solvents were purchased and used in analytical or HPLC grade.

Analytical thin layer chromatography was carried out using silica gel-coated aluminum plates with a fluorescence indicator (MERCK 60 F_{254} or MACHERY-NAGEL ALUGRAM Xtra SIL G/UV_{254}). Detection was carried out by using short wave UV light (λ_{max} = 254 nm) and conc. aq. HCl in case of diol compounds **S4** **(*R*,*R*)^6-(+)-16** and **(*S*,*S*)^6-(+)-16**.[1]

Flash column chromatography was carried out using silica gel 60, grain size 40–63 µm (230–400 mesh) from MACHERY-NAGEL. In some cases, an automated flash chromatography system (GRACE Reveleris X2) was employed using prepacked columns (silica gel, 25 g to 80 g, 30 µm grain size) from INTERCHIM (INTERCHIM Puriflash Silica HP 30 µm flash column).

NMR spectra were recorded at 300 K, unless otherwise noted, on the following spectrometers: BRUKER *Avance III HD 300* [300.1 MHz (^{1}H)], BRUKER *Avance II 400* [400.1 MHz (^{1}H), 100.6 MHz (^{13}C)], BRUKER *Avance Neo 400* with a *Prodigy* CryoProbe [400.1 MHz (^{1}H), 100.6 MHz (^{13}C)] and BRUKER *Avance III HD 500* [500.3 MHz (^{1}H), 125.8 MHz (^{13}C)]. Chemical shifts are reported in parts per million (ppm, δ scale) relative to the signal of tetramethylsilane (δ = 0.00 ppm). ^{1}H NMR spectra are referenced to tetramethylsilane as an internal standard or the residual solvent signal of the respective solvent: $CDCl_3$: δ = 7.26 ppm; CD_2Cl_2: δ = 5.32 ppm, C_6D_6: δ = 7.16 ppm, DMSO-d_6: δ = 2.50 ppm, toluene-d_8: δ = 2.03 ppm. ^{13}C NMR spectra are referenced to the following signals: $CDCl_3$: δ = 77.16 ppm; CD_2Cl_2: δ = 53.84 ppm, C_6D_6: δ = 128.06 ppm, DMSO-d_6: δ = 39.52 ppm, toluene-d_8: δ = 20.43 ppm.[2] Analysis followed first order, and the following abbreviations for multiplets were used: singlet (s), broad singlet (br. s), doublet (d), triplet (t), quartet (q), septet (sept), multiplet (m) and combinations thereof *i.e.* doublet of doublets (dd). Coupling constants (*J*) are given in Hertz [Hz].

ATR FT-IR spectra were recorded at ambient temperature on a BRUKER Vector 22 FT-IR instrument equipped with a SPECAC Golden Gate ATR unit. Samples were measured as powders. Spectra were recorded in the range from 4000–500 cm^{-1} with 64 scans and a resolution of 2 cm^{-1}. Data processing was carried out with the software OPUS 6.5. IR wavelengths are given in wavenumbers [cm^{-1}].

---

1 Diol compounds **S4**, **(*R*,*R*)^6-(+)-16** and **(*S*,*S*)^6-(−)-16** showed a yellow/red spot after treatment with conc. aq. HCl.

High resolution mass spectra were measured on a THERMO FISHER SCIENTIFIC Exactive via electrospray ionization (ESI) or atmospheric pressure chemical ionization (APCI) with an orbitrap analyzer.

Low resolution mass spectra were measured on a THERMO FISHER SCIENTIFIC TSQ 700 using chemical ionization (CI) or electron ionization (EI).

UV/Vis absorption spectra were measured on a SHIMADZU UV-1800 using *Quartz (Suprasil)* cuvettes (10 mm path length) from HELLMA ANALYTICS.

Electronic circular dichroism (ECD) spectra were measured on a JASCO J-810 in CH_2Cl_2, $CHCl_3$ or chlorobenzene solution using 10 mm or 2 mm path length quartz cuvettes at the indicated temperatures and concentrations.

Cyclic voltammograms (CVs) and differential pulse voltammograms (DPVs) were measured inside an argon-filled glovebox using a PGSTAT128N or PGSTAT302N potentiostat by METROHM AUTOLAB. As working electrode a glassy carbon disc electrode (2 mm diameter) was used, as counter electrode a platinum rod was used, as reference electrode a Ag/$AgNO_3$ electrode containing a silver wire immersed in an inner chamber filled with 1 M $AgNO_3$ and 0.1 M *n*-Bu_4NPF_6 in anh. CH_3CN or a silver wire was used. The analyte solution contained 10 mL of solvent (anh. CH_2Cl_2 or chloroform) with 0.1 M *n*-Bu_4NPF_6 and the specified analyte concentration. The ferrocene/ferrocenium redox couple was used as internal reference. HOMO and LUMO levels were calculated using the following equations: E_{LUMO} (eV) = $-(E_{i,Fc}+x_{Red})$ (with $E_{i,Fc}$ = 4.8 eV (ionization energy of ferrocene)[3]; x_{Red} = onset of the first reduction peak, calibrated vs. Fc/Fc^+ in eV), E_{HOMO} (eV) = $-(E_{i,Fc}+x_{Ox})$ (with x_{Ox} = onset of the first oxidation peak, calibrated vs. Fc/Fc^+ in eV).

Analytical HPLC was performed using an AGILENT TECHNOLOGIES 1290 Infinity II with a G1311B-1260 Quat pump, a G7117A-1290 DAD FS detector, a G7129A-1260 Vialsampler autosampler, a G7116B-1290 MCT column oven using AGILENT Open LAB CDS (EZChrome Edition) software version A.04.07. Following chiral columns from DAICEL und PHENOMENEX were used:

PHENOMENEX Lux Cellulose LC-3 (150 mm × 4.6 mm, 3 µm)
DAICEL Chiralpak AD-3 (150 mm × 4.6 mm, 3 µm)
Chiralpak IA (250 mm × 4.6 mm, 5 µm)

Analytical Gel Permeation Chromatography (GPC) was performed by using a GPC unit from AGILENT TECHNOLOGIES composed of an IsoPump G1310A, an ALS G1329A auto sampler, a VWD G1314B UV-detector and an RID G1362A RI-detector. A set of three columns from PSS POLYMER STANDARD SERVICE GmbH was used (8 × 300 mm with a porosity of 102 Å, 103 Å and 105 Å with integrated pre-column). As eluent THF (with 250 ppm BHT as stabilizer) was used with a flow rate of 1 mL min^{-1}. Calibration was carried out with polystyrene standards by PSS POLYMER STANDARD SERVICE GmbH.

Optical rotation was measured on a PERKIN ELMER 241 polarimeter in a cuvette with 1 cm optical pathlength. The specific rotation is given as

$$[\alpha]_\lambda^T = \frac{\alpha \cdot 100}{c \cdot l}$$

with α as the rotation in °, *c* as the concentration in ${}^{g}/_{100\ mL}$, *l* as the length of the cuvette in dm, *T* as the temperature in °C and *λ* as the wavelength in nm. Therefore, the dimension of $[\alpha]_\lambda^T$ is $\frac{\circ \cdot mL}{dm \cdot g}$.

The solutions for measurement were prepared in $CHCl_3$ using a 1 mL volumetric flask and 4.20 mg of **(−)-1**, 4.444 mg of **(+)-1**, 9.76 mg of **(*S*,*S*)^6-(−)-16** or 10.348 mg of **(*R*,*R*)^6-(+)-16**.

Table S1. Measured values of the optical rotation *α* (in °) for *λ* = 589 nm (sodium D line) at the indicated concentrations in $CHCl_3$ mentioned before on the PERKIN ELMER 241 polarimeter. The integration time was set at 5 seconds and each time a value was recorded. The arithmetic mean of this value was used to calculate the specific rotation [*α*] with the formula mentioned above.

	**(−)-1**	**(+)-1**	**(*S*,*S*)^6-(−)-16**	**(*R*,*R*)^6-(+)-16**
	−0.001	+0.003	−0.075	+0.086
	−0.001	+0.002	−0.075	+0.085
	−0.001	+0.002	−0.075	+0.086
	−0.001	+0.002	−0.075	+0.085
	−0.001	+0.003	−0.075	+0.085
				+0,086
Arithmetic mean	**−0.001**	**+0.0024**	**−0.075**	**+0.0855**

2. Synthetic Manipulations

Compound **3**,[4] 2-mesityl-4,4,5,5-tetramethyl-1,3,2-dioxaborolane,[5] and (4-bromo-2,5-diethylphenyl)trimethylsilane[6] were synthesized as previously reported.

1-(4-Bromophenyl)hexan-1-one (S1)

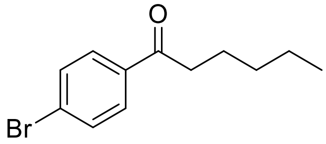

The reaction was performed following a modified procedure by NAGANO *et al*.[7]

Anh. $AlCl_3$ (5.87 g, 44.0 mmol, 1.1 eq.) was added to a dry three-necked round-bottom flask. The flask was equipped with a reflux condenser, evacuated and backfilled with argon, then cooled to 0 °C. Bromobenzene (4.20 mL, 39.9 mmol) was added in one portion, then hexanoyl chloride (6.20 mL, 44.4 mmol, 1.1 eq.) was added to the yellow suspension over 30 min at 0 °C. The mixture was warmed to rt and then to 60 °C and kept at 60 °C for 1 h. The red solution was cooled to rt and poured into ice-water (30 mL). CH_2Cl_2 (50 mL) was added, and the aq. layer was extracted with CH_2Cl_2 (3 × 50 mL). The combined org. layers were washed with aq. HCl (1 M, 50 mL) and brine (50 mL) and dried over Na_2SO_4. The solvent was removed under reduced pressure, and the resulting yellow solid was recrystallized from EtOH (~40 mL). Upon cooling white plate-shaped crystals formed, which were filtered off and washed with cold EtOH (4 × 6 mL) until the solid was completely white. The target compound was obtained as pure white plate-shaped crystals (6.13 g, 24.0 mmol, 60%).

^{1}H NMR (400 MHz, $CDCl_3$): 7.84–7.79 (m, 2H), 7.62–7.57 (m, 2H), 2.95–2.88 (m, 2H), 1.78–1.68 (m, 2H), 1.41–1.30 (m, 4H), 0.97–0.86 (m, 3H); **^{13}C NMR** (101 MHz, $CDCl_3$): δ 199.5, 136.0, 132.0, 129.7, 128.1, 38.7, 31.6, 24.1, 22.6, 14.1; **HRMS** (pos. APCI): *m/z* calcd for $C_{12}H_{16}{}^{79}BrO$ 255.0379 $[M+H]^+$, found 255.0381.

The spectroscopic data is in accordance with the literature.[8]

1-(4-Bromophenyl)hexane (15)

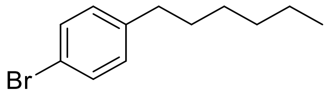

The reaction was performed following a modified procedure by JANG *et al*.[8]

1-(4-Bromophenyl)hexan-1-one (**S1**, 6.00 g, 23.5 mmol), $N_2H_4 \cdot H_2O$ (4.60 mL, 94.8 mmol, 4.0 eq.) and KOH (6.21 g, 94.1 mmol, 4.0 eq.) were refluxed in triethylene glycol (40 mL) for 20 h. After cooling to rt, the reaction mixture was poured into H_2O (100 mL) and extracted with CH_2Cl_2 (3 × 100 mL). The organic layer was washed with brine (50 mL) and dried over Na_2SO_4. The solvent was removed under reduced pressure, and the crude product was purified by filtration over a short column (SiO_2, cyclohexane). **15** was obtained as a colorless solid (5.10 g, 21.1 mmol, 90%).

^{1}H NMR (400 MHz, $CDCl_3$): δ 7.40–7.35 (m, 2H), 7.06–7.01 (m, 2H), 2.61–2.48 (m, 2H), 1.64–1.51 (m, 2H), 1.35–1.22 (m, 6H), 0.93–0.83 (m, 3H); **^{13}C NMR** (101 MHz, $CDCl_3$): δ 142.0, 131.4, 130.3, 119.4, 35.5, 31.8, 31.4, 29.0, 22.7, 14.2; **HRMS** (pos. APCI): *m/z* calcd for $C_{12}H_{16}{}^{79}BrO$ 255.0379 $[M-H_2+OH]^+$, found

255.0357; calcd for $C_{12}H_{16}{}^{79}BrO_2$ 271.0328 $[M-H_2+O+OH]^+$, found 271.0316; calcd for $C_{12}H_{16}{}^{79}BrO_3$ 287.0277 $[M-H_2+O_2+OH]^+$, found 287.0273.
The spectroscopic data is in accordance with the literature.[8]

(2,5-Diethyl-4-(4,4,5,5-tetramethyl-1,3,2-dioxaborolan-2-yl)phenyl)trimethylsilane (8)

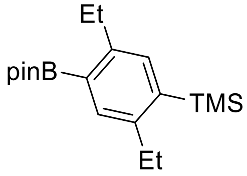

In a dry Schlenk flask, (4-bromo-2,5-diethylphenyl)trimethylsilane[6] (5.51 g, 19.3 mmol) was dissolved in anh. THF (75 mL) and cooled to −78 °C. *n*-BuLi (2.5 M in hexanes, 8.50 mL, 21.3 mmol, 1.1 eq.) was added, and the mixture was stirred at −78 °C for 1 h. *i*-PrOBpin (4.30 mL, 21.1 mmol, 1.09 eq.) was added, the mixture was stirred at −78 °C for 1 h and then warmed to rt and stirred for 3 h. The reaction was quenched with aq. HCl (1 M, 30 mL) and extracted with EtOAc (3 × 30 mL). The org. layer was dried over Na_2SO_4, and the solvent was removed under reduced pressure. The residue was recrystallized from *n*-pentane (5 mL) and obtained as a white solid (6.07 g, 18.3 mmol, 95%).
***R*f** 0.64 (cyclohexane/EtOAc: 20/1); **^{1}H NMR** (400 MHz, $CDCl_3$): δ 7.62 (s, 1H), 7.28 (s, 1H), 2.88 (q, *J* = 7.5 Hz, 2H), 2.74 (q, *J* = 7.5 Hz, 2H), 1.34 (s, 12H), 1.25 (t, *J* = 7.5 Hz, 3H), 1.20 (t, *J* = 7.5 Hz, 3H), 0.33 (s, 9H); **^{13}C NMR** (101 MHz, $CDCl_3$): δ* 147.5, 146.3, 141.1, 135.6, 134.8, 83.4, 28.7, 28.7, 25.0, 17.5, 16.9, 0.6; **HRMS** (pos. APCI): *m/z* calcd for $C_{19}H_{37}BNO_2Si$ 350.2681 $[M+NH_4]^+$, found 350.2676.
*The signal of the quaternary carbon atom connected to boron is not visible in the ^{13}C NMR spectrum due to coupling with ^{10}B and ^{11}B.

2.1 Synthesis of Chiral Auxiliary (*S*)-4 and Chiral Resolution of 3

rac Methyl phenyl sulfoximine (*rac*-S2)

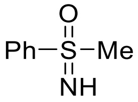

The reaction was performed following a procedure by TOTA *et al.*[9]
Thioanisole (13.0 mL, 111 mmol), (diacetoxyiodo)benzene (81.2 g, 245 mmol, 2.2 eq.) and ammonium carbamate (17.3 g, 222 mmol, 2.0 eq.) were dissolved in MeOH (220 mL) at 0 °C. After the initial foaming wore off, the clear solution was stirred at rt for 17 h. The solvent was removed under reduced pressure, the residue was filtered over a pad of silica gel (2 cm height) and eluted with EtOAc (200 mL). The solvent from the filtrate was removed under reduced pressure, and the residual yellow oil was purified by distillation (p = 5× 10^{-2} mbar, T = 117 °C) to yield a colorless oil (14.5 g, 93.4 mmol, 84%).
***R*f** 0.28 (EtOAc); **^{1}H NMR** (400 MHz, $CDCl_3$): δ 8.05–8.00 (m, 2H), 7.65–7.60 (m, 1H), 7.59–7.53 (m, 2H), 3.11 (s, 3H), 2.70 (br. s, 1H); **^{13}C NMR** (101 MHz, $CDCl_3$): δ 143.7, 133.1, 129.4, 127.8, 46.3; **HRMS** (pos. APCI): *m/z* calcd for $C_7H_{10}NOS$ 156.0478 $[M+H]^+$, found 156.0477.

(*S*)-Methyl phenyl sulfoximinium (*1S*)-(+)-10-camphorsulfonate ((*S*)-S3)

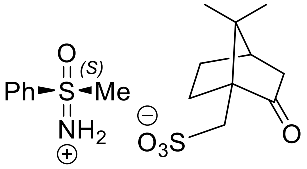

The reaction was performed following a procedure by GAIS *et al.*[10]

(*1S*)-(+)-10-Camphorsulfonic acid (10.8 g, 46.4 mmol, 0.50 eq.) was dissolved in anh. acetone (55 mL)[2] and added dropwise to a solution of ***rac*-S2** (14.4 g, 92.8 mmol, 1.0 eq.) in anh. acetone (52 mL) at rt. After 10 min a white solid started to precipitate, and the mixture was stirred for another 16 h at rt. The precipitate was filtered over a fritted glass-funnel, thoroughly washed with anh. acetone (4 × 12 mL) and *n*-pentane (100 mL) and dried under vacuum. The salt **(*S*)-S3** was obtained as a white powder (13.1 g, 33.9 mmol, 37%). The filtrate was concentrated under reduced pressure, CH_2Cl_2 (50 mL) was added, the mixture was brought to basic pH with 10% aq. NaOH (20 mL), and the aq. layer was extracted with CH_2Cl_2 (3 × 45 mL). The combined organic layers were washed with H_2O (50 mL). The H_2O washings were again extracted with CH_2Cl_2 (3 × 45 mL). All organic layers were combined, dried over Na_2SO_4 and concentrated under reduced pressure to yield a yellow oil. This oil was treated in analogy to ***rac*-S2** with (*1R*)-(−)-10-camphorsulfonic acid (10.8 g, 46.4 mmol) to yield the enantiomeric salt **(*R*)-S3** as a white powder (14.2 g, 36.5 mmol, 39%). **^{1}H-NMR** (400 MHz, DMSO-d_6): δ 8.88 (br. s, 2H), 8.15–8.10 (m, 2H), 7.96–7.90 (m, 1H), 7.85–7.78 (m, 2H), 3.88 (s, 3H, S-CH_3), 2.89 (d, J = 14.7 Hz, 1H), 2.75–2.59 (m, 1H), 2.40 (d, J = 14.7 Hz, 1H), 2.23 (dddd, J = 18.1, 4.6, 3.1, 0.9 Hz, 1H), 1.93 (dd, J = 4.6, 4.6 Hz, 1H), 1.91–1.80 (m, 1H), 1.79 (d, J = 18.1 Hz, 1H), 1.32–1.19 (m, 2 H), 1.04 (s, 3H), 0.74 (s, 3H); **^{13}C-NMR** (101 MHz, DMSO-d_6): δ 216.1, 135.8, 134.4, 130.0, 128.4, 58.2, 47.0, 46.7, 42.3, 42.2, 42.1, 26.4, 24.1, 20.1, 19.5.

[2] Gentle heating of the mixture enabled the dissolution of the camphor sulfonic acid. After some minutes the solution turned red, which was an indicator that no traces of water were left in the acetone. In cases when the solution did not turn red, the obtained enantiomeric excess of **(*S*)-S2** was < 20%. Sufficiently anh. acetone could be obtained by adding activated molecular sieves (3 Å) to the purchased “anhydrous” acetone from ACROS ORGANICS (extra dry, < 50 ppm H_2O, AcroSeal™) and allowed to stand for several days. When the acetone was used as purchased without adding extra molecular sieves, the chiral resolution did not yield a high enantiomeric excess.

(*S*)-Methyl phenyl sulfoximine ((*S*)-S2)

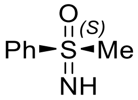

The salt **(*S*)-S3** (13.1 g, 34.0 mmol) was suspended in CH_2Cl_2 (90 mL) and treated with 10% aq. NaOH solution (18 mL), whereupon the solid dissolved. The aqueous layer was extracted with CH_2Cl_2 (3 × 45 mL). The combined organic layers were washed with H_2O (60 mL), and the H_2O washings were further extracted with CH_2Cl_2 (3 × 45 mL). All of the combined organic layers were dried over Na_2SO_4, and the solvent was removed under reduced pressure to yield **(*S*)-S2** as a colorless oil (5.28 g, 34.0 mmol, 100%) that solidified in the freezer.

The spectroscopic data were in accordance with the racemate ***rac*-S2**.

HPLC (Lux Cellulose LC-3, λ = 218 nm, *n*-heptane/*i*-PrOH = 85/15, 0.5 mL min^{-1}, 22 °C): t_R = 13.9 min (minor), 17.1 min (major), > 99% *ee.*

(S)-*N*,*S*-Dimethyl phenyl sulfoximine ((*S*)-4)

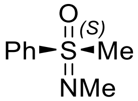

The reaction was performed following a modified procedure by Shiner *et al*.[11]

Sulfoximine **(*S*)-S2** (5.27 g, 33.9 mmol) and paraformaldehyde (2.04 g, 68.0 mmol, 2.0 eq.) were dissolved in formic acid (103 mL) and heated to 100 °C for 48 h. After the end of the reaction, excess formic acid was distilled off under reduced pressure, and aq. H_2SO_4 (2 M, 200 mL) was added. The aqueous layer was washed with CH_2Cl_2 (2 × 60 mL), brought to basic pH with aq. NaOH (4 M, 250 mL) and extracted with CH_2Cl_2 (4 × 180 mL). The organic layer was dried over Na_2SO_4, and the solvent was removed under reduced pressure to yield methylated sulfoximine **(*S*)-4** as a colorless oil (5.57 g, 32.9 mmol, 97%).

***R*f** 0.31 (EtOAc); **^{1}H-NMR** (300 MHz, $CDCl_3$): δ 7.93–7.89 (m, 2H), 7.66–7.54 (m, 3H), 3.08 (s, 3H), 2.66 (s, 3H); **^{13}C-NMR** (101 MHz, $CDCl_3$): δ 138.9, 132.9, 129.5, 128.8, 45.0, 29.6; **HRMS** (pos. ESI): *m/z* calcd for $C_8H_{12}NOS$ 170.06341 $[M+H]^+$, found 170.06328; **HPLC** (Chiralpak AD-3, λ = 248 nm, *n*-heptane/*i*-PrOH = 95/5, 0.5 mL min^{-1}, 22 °C): t_R = 24.5 min (major), 27.8 min (minor), > 99% *ee.*

(S)-*N*,*S*-Dimethyl phenyl sulfoximine adducts 5 and 6

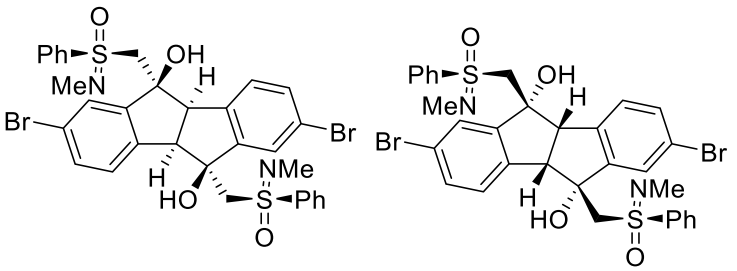

5 **6**

Anhydrous $CeCl_3$ (8.06 g, 32.7 mmol, 3.0 eq.) was placed in a dry Schlenk flask and was further dried under vacuum ($1\cdot10^{-3}$ mbar) at 135 °C for 4 h. The flask was backfilled with argon while hot and cooled to rt. Anh. THF (110 mL) was added at 0 °C, and the mixture was stirred vigorously at rt over night. In another dry Schlenk tube, a solution of sulfoximine **(*S*)-4** (0.20 M in anh. THF, stored over 3 Å molecular sieves, 164 mL, 32.8 mmol, 3.0 eq.) was cooled to 0 °C, EtMgBr (1.0 M in THF, 32.0 mL, 32.0 mmol, 2.94 eq.) was added over 12 min, and the resulting yellow solution was stirred for 1 h at 0 °C. This sulfoximine-MgBr solution was added dropwise via transfer cannula to the $CeCl_3$ slurry at 0°C within 12 min. After 1.5 h of stirring at 0 °C, diketone **3**[4] (4.27 g, 10.9 mmol) was added in one portion. The color of the mixture turned ocher, and the reaction mixture was stirred at 0 °C for 4 h. The reaction was quenched with 10% aq. HOAc (80 mL) at 0 °C, warmed to rt, and the THF was removed under reduced pressure. CH_2Cl_2 (120 mL) was added to the mixture, and the aq. layer was extracted with CH_2Cl_2 (3 × 50 mL). The combined organic layers were washed with sat. aq. $NaHCO_3$ (100 mL), dried over Na_2SO_4 and concentrated under reduced pressure to yield the crude product as a yellowish foam. Automated flash column chromatography (SiO_2, cyclohexane/EtOAc: 9/1 to 0/1) yielded **6** as a white solid (3.59 g, 4.91 mmol, 45%) and **5** as a yellowish solid (3.30 g, 4.51 mmol, 42%).[3] Excess sulfoximine **(*S*)-4** could be recovered as a colorless oil (1.06 g, 6.25 mmol, 57%).[4]

Analytical data for **6**: ***R***$_f$ 0.49 (cyclohexane/EtOAc: 2/1); **^{1}H-NMR** (500 MHz, $CDCl_3$): δ 7.96–7.94 (m, 6H), 7.68–7.58 (m, 8H), 7.39–7.35 (m, 4H), 4.99 (s, 2H), 3.53 (d, *J* = 13.9 Hz, 2H), 3.10 (d, *J* = 13.9 Hz, 2H), 2.78 (s, 6H); **^{13}C-NMR** (126 MHz, $CDCl_3$): δ 148.3, 138.6, 138.0, 133.8, 131.8, 130.8, 130.0, 129.3, 126.6, 121.9, 82.4, 64.0, 53.0, 29.2; **^{1}H-NMR** (400 MHz, C_6D_6): δ 8.04 (br. s, 2H, O*H*), 7.89 (d, *J* = 8.2 Hz, 2H), 7.62–7.60 (m, 6H), 7.31 (dd, *J* = 8.2, 2.0 Hz, 2H), 7.04–6.95 (m, 6H), 4.30 (s, 2H), 3.09 (d, *J* = 13.9 Hz, 2H), 2.61 (s, 6H), 2.40 (d, *J* = 13.9 Hz, 2H); **^{13}C-NMR** (101 MHz, C_6D_6): δ 149.6, 139.1, 139.0, 133.1, 131.7, 131.5, 129.6, 129.5, 127.0, 122.1, 82.8, 63.2, 53.5, 29.0; **HRMS** (pos. ESI): *m/z* calcd for $C_{32}H_{31}O_4N_2{}^{79}Br_2S_2$ 729.0087 $[M+H]^+$, found 729.0083.

Analytical data for **5**: $\boldsymbol{R_f}$ 0.10 (cyclohexane/EtOAc: 2/1); **^{1}H-NMR** (500 MHz, $CDCl_3$, 300 K): δ 7.86–7.83 (m, 4H), 7.63–7.54 (m, 10H), 7.35 (dd, *J* = 8.2, 2.0 Hz, 2H), 7.00 (br. s, 2H), 3.80 (s, 2H), 3.35 (d, *J* = 14.2 Hz, 2H), 3.27 (d, *J* = 14.1 Hz, 2H), 2.74 (s, 6H); **^{1}H-NMR** (500 MHz, $CDCl_3$, 253 K): δ 7.87–7.85 (m, 4H), 7.68–

[3] Both sulfoximine adducts are temperature sensitive and slowly decompose. The NMR spectrum of the crude reaction mixture only shows **6**, **5** and sulfoximine **(*S*)-4**. The given yields and masses are corrected towards residual contained solvent (CH_2Cl_2 and/or EtOAc), since rigorous drying would result in partial decomposition. The reaction was performed several times on this scale and the yields were comparable with the yield of **5** being slightly lower due to a higher temperature sensitivity.

[4] The excess sulfoximine **(*S*)-4** elutes last and quite slowly. More than 90% can be recovered.

7.58 (m, 12H), 7.39 (dd, J = 8.3, 2.0 Hz, 2H), 3.75 (s, 2H), 3.42 (d, J = 14.1 Hz, 2H), 3.26 (d, J = 14.1 Hz, 2H), 2.76 (s, 6H); **^{13}C-NMR**[5] (126 MHz, $CDCl_3$, 253 K): δ 147.1, 138.1, 136.3, 133.8, 131.9, 130.1, 130.0, 129.0, 127.7, 121.9, 81.2, 63.2, 54.1, 29.4; **HRMS** (pos. APCI): *m/z* calcd for $C_{32}H_{31}O_4N_2{}^{79}Br_2S_2$ 729.0087 $[M+H]^+$, found 729.0082.

(4b*R*,9b*R*)-2,7-Dibromo-4b,9b-dihydroindeno[2,1-*a*]indene-5,10-dione ((*R*,*R*)-3)

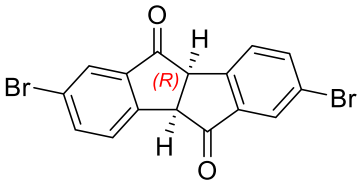

5 (93 mass% purity, containing residual EtOAc, 2.55 g, 3.24 mmol) was dissolved in toluene (330 mL) and stirred at 90 °C until complete conversion was indicated by TLC (3 h). The solvent was removed under reduced pressure, and the residue was purified by column chromatography[6] (SiO_2, CH_2Cl_2 → EtOAc/EtOH 10/1) to yield diketone **(*R*,*R*)-3** as an off-white solid (1.18 g, 3.01 mmol, 93%) and sulfoximine **(*S*)-4** as a colorless oil (1.10 g, 6.48 mmol, quant.).

The spectroscopic data is in accordance with the racemic molecule reported in the literature.[4]

m.p. 276–278 °C, **HPLC** (Chiralpak IA, λ = 250 nm, *n*-heptane/CH_2Cl_2 = 75/25, 1.0 mL min^{-1}, 22 °C): t_R = 11.4 min (major), 17.3 min (minor), > 99% *ee*.

(4b*S*,9b*S*)-2,7-Dibromo-4b,9b-dihydroindeno[2,1-*a*]indene-5,10-dione ((*S*,*S*)-3)

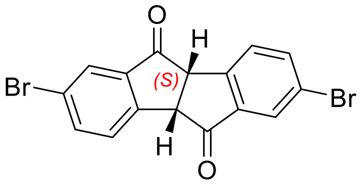

6 (87 mass% purity, containing residual CH_2Cl_2, 2.87 g, 3.43 mmol) was dissolved in toluene (350 mL) and stirred at 90 °C until complete conversion was indicated by TLC (3.5 h). The solvent was removed under reduced pressure, and the residue was purified by column chromatography (SiO_2, CH_2Cl_2 → EtOAc/EtOH 10/1) to yield diketone **(*S*,*S*)-3** as an off-white solid (1.29 g, 3.28 mmol, 96%) and sulfoximine **(*S*)-4** as a colorless oil (1.14 g, 6.74 mmol, 98%).

The spectroscopic data is in accordance with the racemic molecule reported in the literature.[4]

m.p. 278 °C, **HPLC** (Chiralpak IA, λ = 250 nm, *n*-heptane/CH_2Cl_2 = 75/25, 1.0 mL min^{-1}, 22 °C): t_R = 11.5 min (minor), 16.9 min (major), > 99% *ee*.

---

[5] The material is temperature-sensitive and already slightly decomposed at 300 K during the measurement of the carbon NMR spectrum. Therefore, the temperature was kept at 253 K during measurement.

[6] According to Johnson *et. al*[50] the separation of the sulfoximine from the ketone can be performed by washing with aq. 6 N H_2SO_4 or aq. $Cu(NO_3)_2$ solution, which is more suited for larger scales.

2.2 Synthesis of Racemic Reference Compound 7

2,7-Bis(2,5-diethyl-4-(trimethylsilyl)phenyl)-4b,9b-dihydroindeno[2,1-*a*]indene-5,10-dione (*rac*-9)

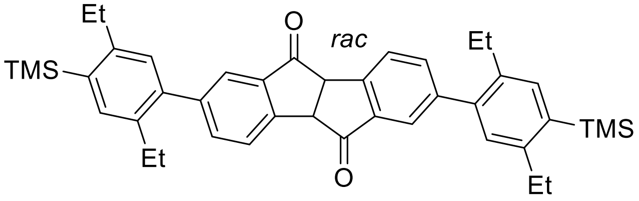

Diketone **3** (400 mg, 1.02 mmol), boronate **8** (749 mg, 2.25 mmol, 2.2 eq.), $Pd(PPh_3)_3$ (47.3 mg, 40.9 µmol, 4.0 mol%) and Na_2CO_3 (4.32 g, 40.8 mmol, 40 eq.) were dissolved in a mixture of THF (40 mL) and H_2O (20 mL), purged with argon for 45 min prior to use, and stirred at 85 °C for 5 h until complete conversion was indicated by TLC. The mixture was allowed to cool to rt and was extracted with CH_2Cl_2 (2 × 30 mL). The combined organic layers were washed with brine (50 mL) and dried over Na_2SO_4. The solvent was removed under reduced pressure. Column chromatography (SiO_2, cyclohexane/CH_2Cl_2 2/1, then cyclohexane/EtOAc 10/1) yielded the title compound as yellowish solid (594 mg, 0.924 mmol, 91%).

***R*f** 0.46 (cyclohexane/EtOAc: 10/1); **m.p.** 153–156 °C,**^{1}H NMR** (400 MHz, $CDCl_3$): δ 7.98 (dd, *J* = 7.8, 0.6 Hz, 2H), 7.71 (dd, *J* = 1.8, 0.6 Hz, 2H), 7.68 (dd, *J* = 7.8, 1.8 Hz, 2H), 7.39 (s, 2H), 7.00 (s, 2H), 4.51 (s, 2H), 2.75 (q, *J* = 7.5 Hz, 4H), 2.55 (q, *J* = 7.5 Hz, 4H), 1.23 (t, *J* = 7.5 Hz, 6H), 1.08 (t, *J* = 7.5 Hz, 6H), 0.37 (s, 18H); **^{13}C NMR** (101 MHz, $CDCl_3$): δ 201.7, 148.5, 147.5, 143.6, 140.9, 137.9, 137.7, 137.2, 135.2, 135.1, 129.5, 126.3, 125.1, 52.9, 28.6, 25.8, 16.4, 15.9, 0.6; **HRMS** (pos. ESI): *m/z* calcd for $C_{42}H_{51}O_2Si_2$ 643.3422 $[M+H]^+$, found 643.3422; **ATR FT-IR** $\tilde{\nu}$ = 2961 (mw), 2930 (w), 2868 (w), 1717 (ms), 1474 (mw), 1418 (mw), 1248 (m), 835 (s).

2,7-Bis(4-bromo-2,5-diethylphenyl)-4b,9b-dihydroindeno[2,1-*a*]indene-5,10-dione (*rac*-10)

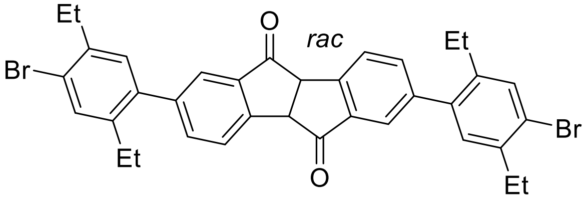

In a dry Schlenk tube, diketone ***rac*-9** (500 mg, 0.778 mmol) was dissolved in anh. CH_2Cl_2 (24 mL), the tube was protected from light with aluminum foil and the mixture cooled to −25°C. A freshly prepared solution of bromine (1 M in anh. CH_2Cl_2, 3.10 mL, 3.10 mmol, 4.0 eq.) was added dropwise over 10 min. The mixture was stirred at a temperature between –25 °C and −10 °C for 4 h (until conversion was complete as indicated by TLC), quenched with sat. aq. Na_2SO_3 (8 mL) and then warmed to rt. H_2O (15 mL) was added, the organic layer was separated, and the aq. layer was extracted with CH_2Cl_2 (3 × 40 mL). The combined organic layers were washed with brine (50 mL) and dried over Na_2SO_4. The solvent was removed under reduced pressure, and the title compound was obtained as a yellow solid (507 mg, 0.772 mmol, 99%) without further purification.

***R*f** 0.34 (cyclohexane/EtOAc: 10/1); **m.p.** 149 °C, **^{1}H NMR** (400 MHz, $CDCl_3$): δ 7.97 (dd, *J* = 7.9, 0.6 Hz, 2H), 7.65 (dd, *J* = 1.8, 0.6 Hz, 2H), 7.62 (dd, *J* = 7.9, 1.8 Hz, 2H), 7.46 (s, 2H), 6.97 (s, 2H), 4.50 (s, 2H), 2.72 (q, *J* = 7.6 Hz, 4H), 2.48 (q, *J* = 7.6 Hz, 4H), 1.20 (t, *J* = 7.6 Hz, 6H), 1.06 (t, *J* = 7.6 Hz, 6H); **^{13}C NMR** (101 MHz, $CDCl_3$): δ 201.5, 148.7, 142.6, 140.9, 140.8, 139.4, 137.1, 135.2, 132.8, 130.9, 126.4, 125.1, 124.2, 52.9, 29.0, 25.6, 15.5, 14.4; **HRMS** (pos. APCI): *m/z* calcd for $C_{36}H_{33}{}^{79}Br_2O_2$ 655.0842 $[M+H]^+$, found 655.0837; **ATR FT-IR** $\tilde{\nu}$ = 2968 (w), 1711 (m), 1702 (m), 1474 (mw), 1159 (mw), 1117 (mw).

2,7-Bis(2,5-diethyl-4-(4,4,5,5-tetramethyl-1,3,2-dioxaborolan-2-yl)phenyl)-4b,9b-dihydroindeno[2,1-*a*]indene-5,10-dione (*rac*-11)

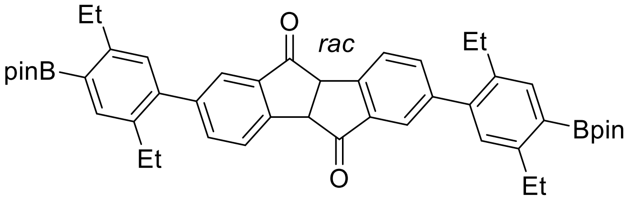

Dibromodiketone ***rac*-10** (215 mg, 328 µmol), bis(pinacolato)diboron (175 mg, 689 µmol, 2.1 eq.), KOAc (193 mg, 197 µmol, 6.0 eq.) and $PdCl_2$(dppf) (14.4 mg, 19.7 µmol, 6 mol%) were dissolved in anh. degassed 1,4-dioxane (3.4 mL) and stirred at 85 °C for 24 h. The mixture was cooled to rt, filtered over a pad of silica gel and eluted with CH_2Cl_2/EtOAc. The solvent was removed under reduced pressure, and the residue was purified by column chromatography (SiO_2, CH_2Cl_2 to CH_2Cl_2/MeOH 100/1) to yield the title compound as an off-white solid (206 mg, 274 µmol, 84%).

***R*f** 0.71 (cyclohexane/CH_2Cl_2/EtOAc: 20/5/1); **m.p.** 217 °C, ***R*f** 0.24 (CH_2Cl_2); **^{1}H NMR** (400 MHz, $CDCl_3$): δ 7.97 (dd, *J* = 7.9, 0.6 Hz, 2H), 7.70 (s, 2H), 7.68 (dd, *J* = 1.7, 0.6 Hz, 2H), 7.65 (dd, *J* = 7.9, 1.7 Hz, 2H), 6.96 (s, 2H), 4.50 (s, 2H), 2.88 (q, *J* = 7.5 Hz, 4H), 2.53 (q, *J* = 7.5 Hz, 4H), 1.35 (s, 24H), 1.17 (t, *J* = 7.5 Hz, 6H), 1.05 (t, *J* = 7.5 Hz, 6H); **^{13}C NMR** (101 MHz, $CDCl_3$): δ 201.7, 148.9, 148.4, 143.5, 142.4, 137.8, 137.1, 136.8, 135.0, 130.1, 127.7, 126.2, 125.0, 83.5, 52.8, 28.4, 25.6, 24.9, 17.1, 15.9; **HRMS** (pos. APCI): *m/z* calcd for $C_{48}H_{57}O_6B_2$ 751.4336 $[M+H]^+$, found 751.4341; **ATR FT-IR** $\tilde{\nu}$ = 2966 (w), 2930 (w), 1712 (ms), 1611 (w), 1343 (ms), 1141 (s), 1113 (m), 852 (m), 638 (mw).

7,7'-((5,10-Dioxo-4b,5,9b,10-tetrahydroindeno[2,1-*a*]indene-2,7-diyl)bis(2,5-diethyl-4,1-phenylene))bis(2-bromo-4b,9b-dihydroindeno[2,1-*a*]indene-5,10-dione) (18)

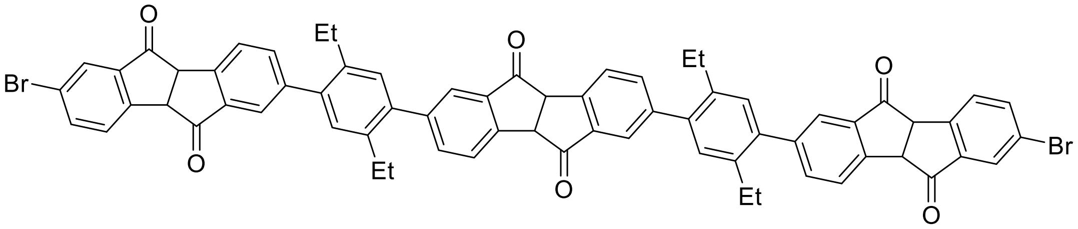

Boronate ***rac*-11** (210 mg, 280 µmol), dibromodiketone **3** (660 mg, 1.68 mmol, 6.0 eq.), $PdCl_2$(dppf) (12.5 mg, 17.1 µmol, 6 mol%) and K_3PO_4 (476 mg, 2.24 mmol, 8.0 eq.) were dissolved in a mixture of toluene (56 mL) and H_2O (5.5 mL), purged with argon for 1 h prior to use. The mixture was stirred at 85 °C for 24 h and allowed to cool to rt. H_2O (15 mL) was added, and the aq. layer was extracted with CH_2Cl_2 (3 × 40 mL). The combined organic layers were dried over Na_2SO_4, and the solvent was removed under reduced pressure. Column chromatography (SiO_2, cyclohexane/CH_2Cl_2/EtOAc: 60/15/1 to 40/10/1) yielded **18** as an off-white solid (190 mg, 170 µmol, 61%). Excess diketone **3** could be recovered as a white solid (377 mg, 962 µmol, 85%).

***R*$_f$** 0.32 (cyclohexane/CH_2Cl_2/EtOAc: 40/10/1); **m.p.** 325 °C, ***R*$_f$** 0.28 (cyclohexane/acetone: 4/1); **^{1}H NMR** (400 MHz, $CDCl_3$): δ 8.00 (d, *J* = 7.9 Hz, 2H), 7.95 (dd, *J* = 8.6, 1.0 Hz, 2H), 7.87 (d, *J* = 1.8 Hz, 2H), 7.83 (d, *J* = 8.3 Hz, 2H), 7.79 (dd, *J* = 8.3, 1.8 Hz, 2H), 7.73 (dd, *J* = 1.7, 0.7 Hz, 2H), 7.72–7.67 (m, 6H), 7.06 (s, 2H), 7.05 (s, 2H), 4.53 (s, 2H), 4.49 (dd, *J* = 5.7, 1.0 Hz, 2H), 4.40 (dd, *J* = 5.7, 0.9 Hz, 2H), 2.58–2.47 (m, 8H), 1.09–1.00 (m, 12H); **^{13}C NMR** (101 MHz, $CDCl_3$): δ 201.7, 200.9, 200.3, 148.6, 148.5, 148.1, 143.3, 143.1, 139.9, 139.7, 139.0, 139.0, 138.8, 137.4, 137.3, 136.8, 135.2, 134.9, 130.4, 130.4, 128.2, 127.9, 126.4, 126.3, 125.3, 125.2, 123.7, 52.9, 52.7, 52.7, 25.7, 15.7; **HRMS** (pos. APCI): *m/z* calcd for $C_{68}H_{49}O_6{}^{79}Br_2$ 1119.1881 $[M+H]^+$, found 1119.1890; **ATR FT-IR** $\tilde{\nu}$ = 1711 (mw), 1179 (w), 1115 (w), 655 (w), 598 (mw).

7,7'-((5,10-Dioxo-4b,5,9b,10-tetrahydroindeno[2,1-a]indene-2,7-diyl)bis(2,5-diethyl-4,1-phenylene))bis(2-mesityl-4b,9b-dihydroindeno[2,1-a]indene-5,10-dione) (19)

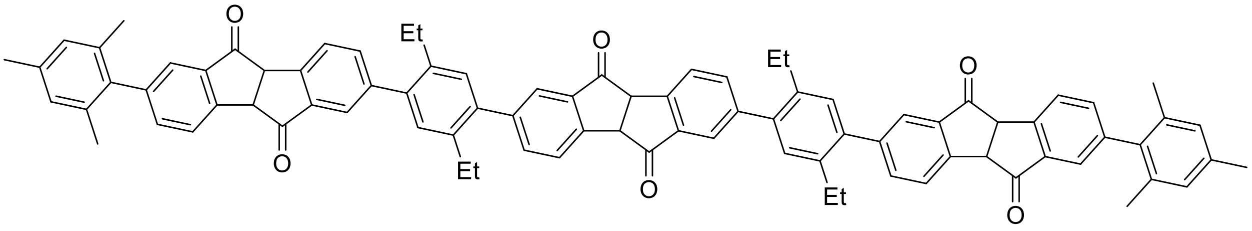

The reaction was performed following a modified procedure by Grenz *et al.*[5]

18 (115 mg, 103 µmol), 2-mesityl-4,4,5,5-tetramethyl-1,3,2-dioxaborolane[5] (106 mg, 431 µmol, 4.2 eq.) and $Pd(PPh_3)_4$ (1.8 mg, 1.6 µmol, 1.5 mol%) were dissolved in a degassed mixture of THF (3.0 mL) and aq. Na_2CO_3 (2 M, 1.0 mL). The resulting suspension was stirred at 80 °C for 18 h. A second portion of $Pd(PPh_3)_4$ (2.2 mg, 1.9 µmol, 1.8 mol%) and 2-mesityl-4,4,5,5-tetramethyl-1,3,2-dioxaborolane (102 mg, 414 µmol, 4.0 eq.) was added, and the mixture was stirred at 80 °C for another 12 h. After allowing the mixture to cool to rt the aq. layer was extracted with CH_2Cl_2 (3 × 10 mL). The combined organic layers were dried over Na_2SO_4, and the solvent was removed under reduced pressure. Column chromatography (SiO_2, cyclohexane/EtOAc: 20/1 to 5/1) yielded the title compound as an off-white solid (100 mg, 83.4 µmol, 81%). ***R*f** 0.11 (cyclohexane/EtOAc: 10/1); **m.p.** 302–305 °C, **^{1}H NMR** (500 MHz, $CDCl_3$): δ 8.00 (d, *J* = 8.0 Hz, 4H), 7.99 (d, *J* = 7.8 Hz, 2H), 7.75–7.73 (m, 4H), 7.72–7.69 (m, 4H), 7.54 (t, *J* = 1.6, 0.8 Hz, 2H), 7.49 (dd, *J* = 7.9, 1.7 Hz, 2H), 7.08 (s, 2H), 7.07 (s, 2H), 6.96–6.90 (m, 4H), 4.53 (s, 2H), 4.51 (s, 4H), 2.54 (q, *J* = 7.5 Hz, 4H), 2.53 (q, *J* = 7.5 Hz, 4H), 2.32 (s, 6H), 1.98 (s, 6H), 1.91 (s, 6H), 1.06 (t, *J* = 7.6 Hz, 6H), 1.06 (t, *J* = 7.6 Hz, 6H); **^{13}C NMR** (126 MHz, $CDCl_3$)[7]: δ 201.8, 201.72, 201.68, 148.6, 148.6, 148.3, 143.2, 143.1, 142.7, 139.84, 139.81, 139.0, 137.7, 137.4, 137.36, 137.34, 136.0, 135.8, 135.4, 135.25, 135.17, 130.42, 130.39, 128.43, 128.39, 126.7, 126.4, 125.6, 125.2, 52.94, 52.91, 25.7, 21.2, 21.0, 20.9, 15.68, 15.66; **HRMS** (pos. ESI): *m/z* calcd for $C_{86}H_{70}O_6$ 1198.5172 $[M]^+$, found 1198.5192; **ATR FT-IR** $\tilde{\nu}$ = 2968 (w), 1711 (ms), 1609 (w), 1474 (mw), 1180 (mw), 1114 (m), 852 (m).

---

[7] Some signals are missing due to overlap.

Compound S4

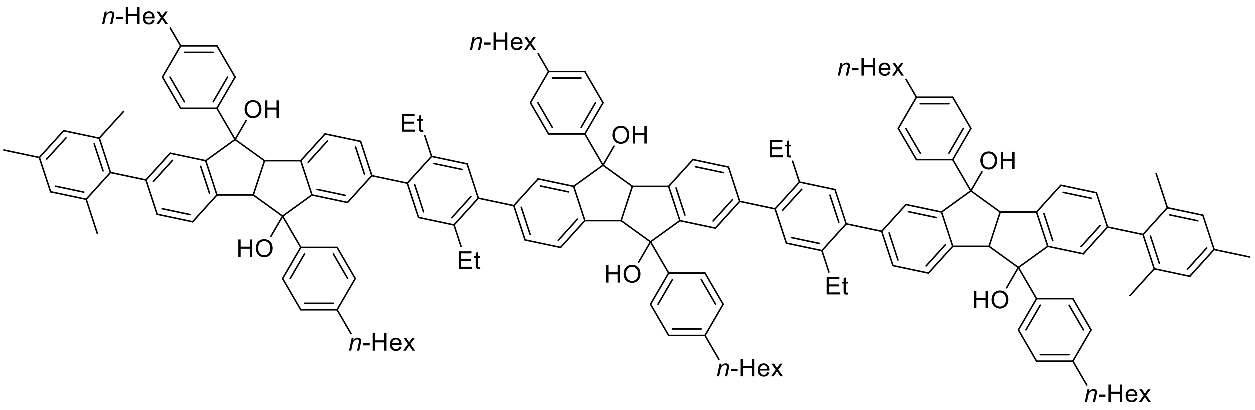

In an oven-dried Schlenk tube, anh. $CeCl_3$ (123 mg, 499 µmol, 10 eq.) was dried at 135 °C and 1×10^{-3} mbar for 3 h. The tube was backfilled with argon while hot, allowed to cool to rt, and anh. THF (1.5 mL) was added. The resulting white suspension was stirred at rt over night. For the preparation of the Grignard reagent a dry Schlenk tube was charged with Mg shavings (24.0 mg, 1.03 mmol) and heated under vacuum with a heat gun for 5 min. After cooling to rt 1-bromo-4-hexylbenzene (**15**, 0.18 mL, 880 µmol) and anh. THF (1.0 mL) were added, and the Grignard reaction was started under gentle heating. The mixture was stirred until all Mg was consumed. The $CeCl_3$ suspension was cooled to 0 °C, and half of the Grignard solution (~0.5–0.6 mL) was added. The mixture was stirred at 0 °C for 1 h. Hexaketone **19** (60.0 mg, 50.0 µmol, dried at 75 °C under vacuum over night) was added in one portion, and the resulting yellow mixture was stirred at 0 °C for 4 h. The reaction mixture was quenched with 10% aq. HOAc (5 mL) and allowed to warm to rt. CH_2Cl_2 (20 mL) was added, and the aq. layer was extracted with CH_2Cl_2 (3 × 10 mL). The combined organic layers were washed with aq. sat. $NaHCO_3$ (10 mL), dried over Na_2SO_4, and the solvent was removed under reduced pressure. Column chromatography (SiO_2, cyclohexane/EtOAc: 20/1 to 4/1) yielded the title compound as a colourless solid (68 mg, 31 µmol, 63%).

***R*f** 0.20 (cyclohexane/EtOAc: 6/1); **^{1}H NMR** (500 MHz, CD_2Cl_2): δ 7.50–7.43 (m, 12H), 7.33–7.29 (m, 4H), 7.28–7.23 (m, 12H), 7.20–7.15 (m, 6H), 7.12 (d, *J* = 2.1 Hz, 4H), 7.10 (dd, *J* = 7.7, 1.6 Hz, 2H), 7.02 (br. s, 4H), 6.90 (s, 2H), 6.88 (s, 2H), 6.83 (d, *J* = 1.6 Hz, 2H), 4.48–4.37 (m, 6H), 3.40 (s, 4H, O*H*), 3.34 (s, 2H, O*H*), 2.68–2.63 (m, 12H), 2.57–2.49 (m, 8H), 2.28 (s, 6H), 2.06 (s, 6H), 1.94 (s, 6H), 1.71–1.62 (m, 12H), 1.42–1.30 (m, 36H), 1.03 (t, *J* = 7.5 Hz, 6H), 1.02 (t, *J* = 7.5 Hz, 6H), 0.93–0.88 (m, 18H); **^{13}C NMR** (126 MHz, CD_2Cl_2):[8] δ 151.2, 150.8, 150.8, 142.9, 142.9, 142.8, 142.5, 142.4, 142.3, 142.3, 142.2, 141.8, 140.9, 140.9, 139.5, 139.5, 139.1, 139.0, 136.8, 136.1, 136.1, 130.6, 130.3, 130.2, 130.1, 128.8, 128.6, 128.6, 128.5, 128.3, 128.3, 126.8, 126.7, 126.6, 126.4, 125.8, 125.5, 84.3, 84.1, 64.5, 64.4, 64.3, 36.0, 32.1, 31.9, 29.5, 29.4, 26.1, 21.1, 21.0, 15.9, 14.3; **HRMS** (pos. ESI): *m/z* calcd for $C_{157}{}^{13}C_1H_{175}O_4$ 2137.3519 $[M-(H_2O)-OH]^+$, found 2137.3582.

[8] Not all signals can be identified and many signals overlap due to chemical similarity.

7,7'-((5,10-Bis(4-hexylphenyl)indeno[2,1-*a*]indene-2,7-diyl)bis(2,5-diethyl-4,1-phenylene))bis(5,10-bis(4-hexylphenyl)-2-mesitylindeno[2,1-*a*]indene) (7)

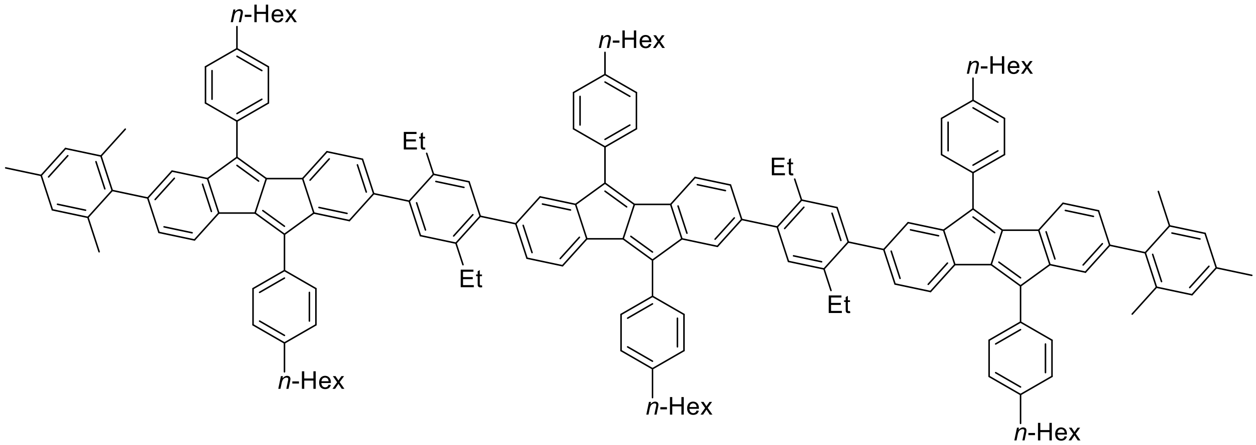

Hexaol **S4** (30.1 mg, 13.9 mmol) and trifluoroacetic anhydride (0.02 mL, 144 µmol, 10 eq.) were dissolved in $CHCl_3$ (5 mL)[9] and refluxed for 45 min, during which the solution turned deep red. After cooling to rt, $CHCl_3$ (10 mL) was added, and the mixture was washed with sat. aq. $NaHCO_3$ (10 mL) and dried over Na_2SO_4. The solvent was removed under reduced pressure to yield DBP **7** as a red solid (29 mg, 13.9 µmol, 100%).

^{1}H NMR (500 MHz, $CDCl_3$)[10]: δ 7.64–7.61 (m, 8H), 7.60–7.57 (m, 4H), 7.33–7.27 (m, 18H), 7.10 (s, 2H), 7.09 (s, 2H), 7.06 (d, *J* = 1.5 Hz, 2H), 7.06 (d, *J* = 1.5 Hz, 2H), 6.92 (s, 4H), 6.85 (d, *J* = 1.5 Hz, 2H), 6.84 (dd, *J* = 7.6, 1.5 Hz, 2H), 6.83 (dd, *J* = 7.6, 1.5 Hz, 2H), 6.63 (dd, *J* = 7.5, 1.5 Hz, 2H), 2.71–2.58 (m, 20H), 2.31 (s, 6H), 2.08 (s, 12H), 1.72–1.62 (m, 12H), 1.42–1.30 (m, 36H), 1.12 (t, *J* = 7.5 Hz, 6H), 1.11 (t, *J* = 7.5 Hz, 6H), 0.92–0.87 (m, 18H); **^{13}C NMR** (126 MHz, $CDCl_3$)[11]: δ 150.1, 149.9, 144.0, 144.0, 144.0, 143.3, 143.2, 143.0, 141.5, 141.4, 141.0, 141.0, 140.8, 140.7, 140.6, 140.4, 139.2, 139.0, 136.7, 136.2, 134.0, 133.8, 131.3, 130.0, 128.8, 128.8, 128.6, 128.2, 123.8, 123.8, 123.8, 122.0, 121.6, 121.6, 121.6, 36.1, 31.9, 31.5, 29.9, 29.2, 26.0, 22.8, 21.1, 20.9, 15.9, 15.9, 14.3; **HRMS** (pos. ESI): *m/z* calcd for $C_{158}H_{166}$ 2063.2984 $[M]^{\bullet+}$, found 2063.2998; **ATR FT-IR** $\tilde{\nu}$ = 2963 (w), 2929 (mw), 2850 (w), 1441 (mw), 829 (mw).

---

[9] $CHCl_3$ may not be replaced with CH_2Cl_2 since **7** is insoluble in CH_2Cl_2.
[10] Low solubility, ns = 960, containts some residual CH_2Cl_2.
[11] Some signals are missing due to overlap. 64000 scans were recorded, but due to extremely low solubility the signal to noise ratio is quite poor.

2.3 Synthesis of Enantiomerically Pure Acyclic Nanohoop Precursors

(4b*R*,9b*R*)-2,7-Bis(2,5-diethyl-4-(trimethylsilyl)phenyl)-4b,9b-dihydroindeno[2,1-*a*]indene-5,10-dione ((*R*,*R*)-9)

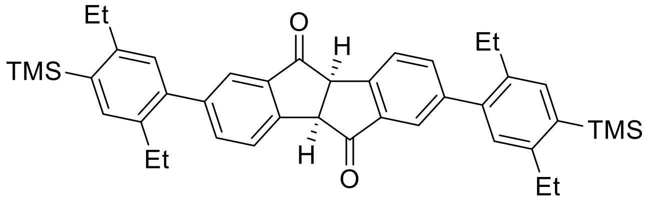

(*R*,*R*)-9 was synthesized using the same procedure described above for racemic compound ***rac*-9** using diketone **(*R*,*R*)-3** (800 mg, 2.04 mmol), **8** (1.49 g, 4.48 mmol, 2.2 eq.), $Pd(PPh_3)_4$ (94.3 mg, 81.6 µmol, 4 mol%), Na_2CO_3 (8.65 g, 81.6 mmol, 40 eq.), THF (80 mL) and H_2O (40 mL). **(*R*,*R*)-9** was obtained as a yellowish solid (1.24 g, 1.93 mmol, 95%).

The spectroscopic data is in accordance with the racemic molecule ***rac*-9**.

m.p. 172 °C, **HPLC** (Chiralpak IA, λ = 247 nm, *n*-heptane/$CHCl_3$ = 90/10, 1.0 mL min^{-1}, 22°C): t_R = 13.9 min (major), 30.1 min (minor), 99% *ee*.

(4b*S*,9b*S*)-2,7-Bis(2,5-diethyl-4-(trimethylsilyl)phenyl)-4b,9b-dihydroindeno[2,1-*a*]indene-5,10-dione ((*S*,*S*)-9)

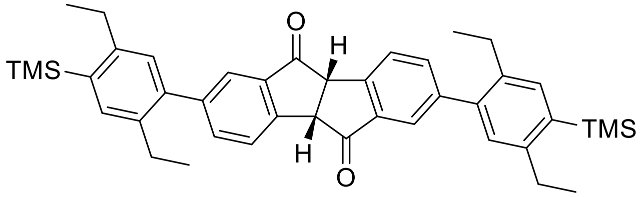

(*S*,*S*)-9 was synthesized using the same procedure described above for racemic compound ***rac*-9** using diketone **(*S*,*S*)-3** (700 mg, 1.79 mmol), **8** (1.30 g, 3.91 mmol, 2.2 eq.), $Pd(PPh_3)_4$ (83.0 mg, 71.8 µmol, 4 mol%), Na_2CO_3 (7.55 g, 71.2 mmol, 40 eq.), THF (72 mL) and H_2O (36 mL). **(*S*,*S*)-9** was obtained as a yellow solid (1.07 g, 1.66 mmol, 93%).

The spectroscopic data is in accordance with the racemic molecule ***rac*-9**.

HPLC (Chiralpak IA, λ = 240 nm, *n*-heptane/CH_2Cl_2 = 75/25, 0.5 mL min^{-1}, 22°C): t_R = 4.1 min (minor), 6.3 min (major), >99% *ee*.

(4b*R*,9b*R*)-2,7-Bis(4-bromo-2,5-diethylphenyl)-4b,9b-dihydroindeno[2,1-*a*]indene-5,10-dione ((*R*,*R*)-10)

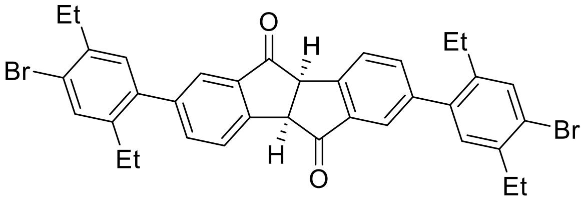

(*R*,*R*)-10 was synthesized using the same procedure described above for racemic compound ***rac*-10** using diketone **(*R*,*R*)-9** (962 mg, 1.50 mmol), Br_2 (1.0 M in CH_2Cl_2, 6.00 mL, 6.00 mmol, 4.0 eq.) and CH_2Cl_2 (45 mL). **(*R*,*R*)-10** was obtained as an off-white solid (1.00 g, 1.50 mmol, quant.).

The spectroscopic data is in accordance with the racemic molecule.

m.p. 163–166 °C, **HPLC** (Chiralpak IA, λ = 247 nm, *n*-heptane/$CHCl_3$ = 85/15, 1.0 mL min^{-1}, 22°C): t_R = 8.8 min (major), 16.0 min (minor), > 99% *ee*.

(4b*S*,9b*S*)-2,7-Bis(4-bromo-2,5-diethylphenyl)-4b,9b-dihydroindeno[2,1-*a*]indene-5,10-dione ((*S*,*S*)-10)

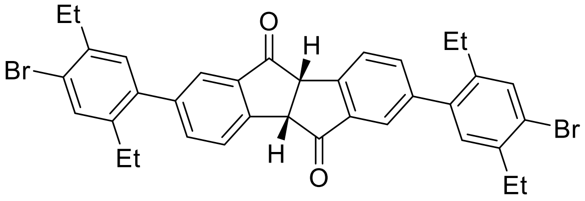

(*S*,*S*)-10 was synthesized using the same procedure described above for racemic compound ***rac*-10** using diketone **(*S*,*S*)-9** (953 mg, 1.48 mmol), Br_2 (1.0 M in CH_2Cl_2, 5.92 mL, 5.92 mmol, 4.0 eq.) and CH_2Cl_2 (44 mL). **(*S*,*S*)-10** was obtained as a white solid (782 mg, 1.19 mmol, 80%).

The spectroscopic data is in accordance with the racemic molecule.

HPLC (Chiralpak IA, λ = 248 nm, *n*-heptane/CH_2Cl_2 = 60/40, 0.5 mL min^{-1}, 22°C): t_R = 8.1 min (minor), 10.4 min (major), 98% *ee*.

(4b*R*,9b*R*)-2,7-Bis(2,5-diethyl-4-(4,4,5,5-tetramethyl-1,3,2-dioxaborolan-2-yl)phenyl)-4b,9b-dihydroindeno[2,1-*a*]indene-5,10-dione ((*R*,*R*)-11)

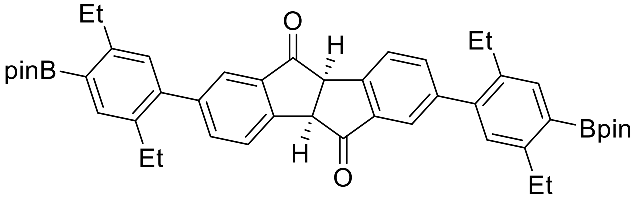

(*R*,*R*)-11 was synthesized using the same procedure described above for racemic compound ***rac*-11** using diketone **(*R*,*R*)-10** (980 mg, 1.49 mmol), bis(pinacolato)diboron (798 mg, 3.14 mmol, 2.1 eq.), KOAc (883 mg, 9.00 mmol, 6.0 eq.) $PdCl_2$(dppf) (66.0 mg, 90.2 µmol, 6 mol%) and 1,4-dioxane (15 mL). After washing the crude product with MeOH **(*R*,*R*)-11** was obtained as a yellowish solid (1.08 g, 1.43 mmol, 96%).

The spectroscopic data is in accordance with the racemic molecule.

m.p. 186 °C.

(4b*S*,9b*S*)-2,7-Bis(2,5-diethyl-4-(4,4,5,5-tetramethyl-1,3,2-dioxaborolan-2-yl)phenyl)-4b,9b-dihydroindeno[2,1-*a*]indene-5,10-dione ((*S*,*S*)-11)

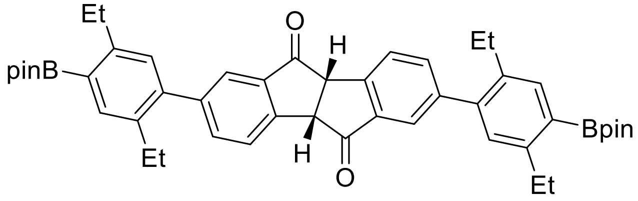

***(S,S)*-11** was synthesized using the same procedure described above for racemic compound ***rac*-11** using diketone **(*S*,*S*)-10** (712 mg, 1.08 mmol), bis(pinacolato)diboron (579 mg, 2.28 mmol, 2.1 eq.), KOAc (639 mg, 6.51 mmol, 6.0 eq.) $PdCl_2$(dppf) (48.3 mg, 66.0 µmol, 6 mol%) and 1,4-dioxane (11 mL). After washing the crude product with MeOH **(*S*,*S*)-11** was obtained as a beige solid (756 mg, 1.01 mmol, 93%). The spectroscopic data is in accordance with the racemic molecule.

(4b*R*,4b'*R*,9b*R*,9b'*R*)-7,7'-(((4b*R*,9b*R*)-5,10-Dioxo-4b,5,9b,10-tetrahydroindeno[2,1-*a*]indene-2,7-diyl)bis(2,5-diethyl-4,1-phenylene))bis(2-bromo-4b,9b-dihydroindeno[2,1-*a*]indene-5,10-dione) ((*R*,*R*)^3-12)

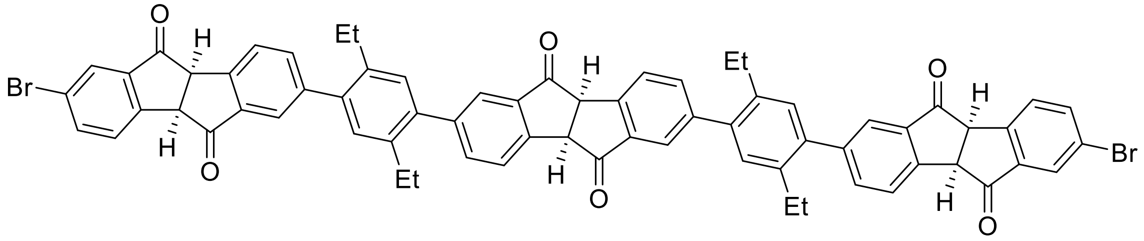

Boronate **(*R*,*R*)-11** (740 mg,0.984 mmol), dibromodiketone **(*R*,*R*)-3** (2.31 g, 5.89 mmol, 6.0 eq.), $PdCl_2$(dppf) (43.2 mg, 59.0 µmol, 6 mol%) and K_3PO_4 (1.67 g, 7.87 mmol, 8.0 eq.) were dissolved in a mixture of toluene (200 mL) and H_2O (20 mL), purged with argon for 2 h prior to use. The mixture was stirred at 85 °C for 24 h and allowed to cool to rt. The layers were separated, brine (100 mL) was added to the aq. layer, and the aq. layer was extracted with CH_2Cl_2 (2 × 100 mL). The combined organic layers were washed with brine (100 mL), dried over Na_2SO_4, and the solvent was removed under reduced pressure. Automated flash column chromatography (SiO_2, cyclohexane/CH_2Cl_2: 3/1 to CH_2Cl_2 to CH_2Cl_2/EtOAc 95/5) yielded trimer **(*R*,*R*)^3-12** as a yellowish solid (628 mg, 0.56 mmol, 57%). Excessively employed diketone **(*R*,*R*)-3** could be recovered as a white crystalline solid (1.40 g, 3.57 mmol, 91%) with no loss in optical purity.

***R*$_f$** 0.21 (CH_2Cl_2); **m.p.** >350 °C, **^{1}H NMR** (500 MjHz, $CDCl_3$): δ 8.00 (d, *J* = 7.9 Hz, 2H), 7.97–7.93 (m, 2H), 7.87 (d, *J* = 1.9 Hz, 2H), 7.83 (d, *J* = 8.2 Hz, 2H), 7.79 (dd, *J* = 8.2, 1.9 Hz, 2H), 7.74–7.72 (m, 2H), 7.71–7.68 (m, 6H), 7.06 (s, 2H), 7.05 (s, 2H), 4.53 (s, 2H), 4.49 (dd, *J* = 5.7, 1.0 Hz, 2H), 4.40 (dd, *J* = 5.7, 0.9 Hz, 2H), 2.53 (q, *J* = 7.5 Hz, 4H), 2.51 (q, *J* = 7.5 Hz, 4H), 1.05 (t, *J* = 7.5 Hz, 6H), 1.04 (t, *J* = 7.5 Hz, 6H); **^{13}C NMR** (126 MHz, $CDCl_3$): δ 201.7, 200.9, 200.3, 148.6, 148.5, 148.1, 143.3, 143.1, 139.9, 139.7, 139.0, 139.0, 138.8, 137.4, 137.3, 136.8, 135.2, 135.0, 130.4, 130.4, 128.2, 127.9, 126.4, 126.3, 125.3, 125.2, 123.7, 52.9, 52.7, 52.7, 25.7, 15.6; **HRMS** (pos. ESI): *m*/*z* calcd for $C_{68}H_{49}O_6{}^{79}Br_2$ 1119.1890 $[M+H]^+$, found 1119.1890, **ATR FT-IR** $\tilde{\nu}$ = 1701 (m), 1716 (mw), 1180 (w), 851 (w), 596 (mw).

(4b*S*,4b'*S*,9b*S*,9b'*S*)-7,7'-(((4b*S*,9b*S*)-5,10-Dioxo-4b,5,9b,10-tetrahydroindeno[2,1-*a*]indene-2,7-diyl)bis(2,5-diethyl-4,1-phenylene))bis(2-bromo-4b,9b-dihydroindeno[2,1-*a*]indene-5,10-dione) ((*S*,*S*)³-12)

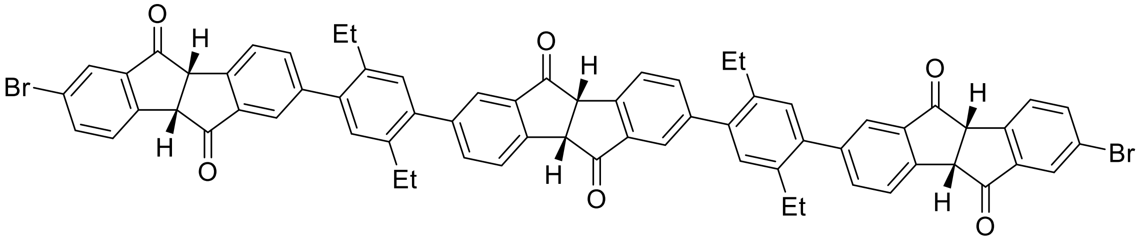

(*S*,*S*)³-12 was synthesized using the same procedure described above for **(*R*,*R*)³-12** using boronate **(*S,S*)-11** (543 mg,0.723 mmol), dibromodiketone **(*S,S*)-3** (1.70 g, 4.34 mmol, 6.0 eq.), PdCl$_2$(dppf) (31.7 mg, 43.3 µmol, 6 mol%), K_3PO_4 (1.23 g, 5.80 mmol, 8.0 eq.), toluene (150 mL) and H_2O (15 mL) to yield trimer **(*S,S*)³-12** as a yellowish solid (384 mg, 0.345 mmol, 48%).

***R*$_f$** 0.21 (CH_2Cl_2); **^{1}H NMR** (500 MHz, $CDCl_3$): δ 8.00 (d, *J* = 7.9 Hz, 2H), 7.97–7.94 (m, 2H), 7.87 (d, *J* = 1.9 Hz, 2H), 7.85–7.82 (m, 2H), 7.79 (dd, *J* = 8.2, 1.9 Hz, 2H), 7.73 (dd, *J* = 1.7, 0.6 Hz, 2H), 7.71–7.67 (m, 6H), 7.06 (s, 2H), 7.05 (s, 2H), 4.53 (s, 2H), 4.49 (dd, *J* = 5.7, 1.0 Hz, 2H), 4.40 (dd, *J* = 5.7, 0.9 Hz, 2H), 2.53 (q, *J* = 7.5 Hz, 4H), 2.51 (q, *J* = 7.5 Hz, 4H), 1.05 (t, *J* = 7.5 Hz, 6H), 1.04 (t, *J* = 7.5 Hz, 6H); **^{13}C NMR** (126 MHz, $CDCl_3$): δ 201.7, 200.9, 200.3, 148.6, 148.5, 148.1, 143.3, 143.1, 139.9, 139.7, 139.0, 139.0, 138.8, 137.4, 137.3, 136.8, 135.2, 135.0, 130.41, 130.36, 128.2, 127.9, 126.4, 126.3, 125.3, 125.2, 123.7, 52.9, 52.74, 52.67, 25.7, 15.7; **HRMS** (pos. ESI): *m*/*z* calcd for $C_{68}H_{49}O_6{}^{79}Br_2$ 1119.1890 $[M+H]^+$, found 1119.1886.

Compound (*R*,*R*)³-13

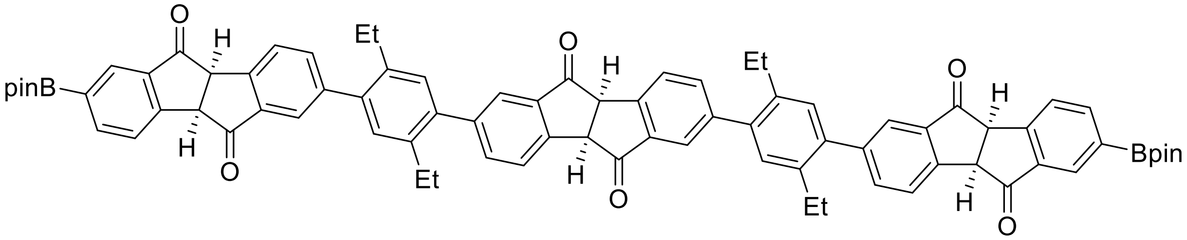

In a dry Schlenk tube trimer **(*R*,*R*)³-12** (344 mg, 307 µmol), bis(pinacolato)diboron (172 mg, 677 µmol, 2.2 eq.), KOAc (182 mg, 1.85 mmol, 6.0 eq.) and PdCl$_2$(dppf) (13.8 mg, 18.9 µmol, 6 mol%) were dissolved in anh. degassed 1,4-dioxane (3.4 mL) and stirred at 85 °C for 26 h. The mixture was cooled to rt, filtered over a pad of silica gel and eluted with CH_2Cl_2/EtOAc. The solvent was removed under reduced pressure, and the residue was washed with MeOH (2 × 4 mL) to yield the title compound as an off-white solid (372 mg, 306 µmol, quant.).

^{1}H NMR (400 MHz, $CDCl_3$): δ 8.22 (dd, *J* = 1.0 Hz, 2H), 8.11 (dd, *J* = 7.7, 1.2 Hz, 2H), 7.99 (dd, *J* = 7.9, 0.6 Hz, 2H), 7.98–7.93 (m, 4H), 7.73 (dd, *J* = 1.8, 0.7 Hz, 2H), 7.71–7.65 (m, 6H), 7.05 (s, 2H), 7.05 (s, 2H), 4.52 (s, 2H), 4.48 (dd, *J* = 5.7, 0.9 Hz, 2H), 4.45 (dd, *J* = 5.7, 0.9 Hz, 2H), 2.53 (q, *J* = 7.5 Hz, 4H), 2.50 (q, *J* = 7.5 Hz, 4H), 1.33 (s, 12H), 1.32 (s, 12H), 1.05 (t, *J* = 7.5 Hz, 6H), 1.03 (t, *J* = 7.5 Hz, 6H);

^{13}C NMR (101 MHz, $CDCl_3$)[12]: δ 201.6, 201.4, 201.3, 152.4, 148.6, 148.5, 143.1, 143.0, 142.0, 139.8, 139.7, 139.0, 138.9, 137.2, 137.1, 135.1, 135.0, 134.5, 131.7, 130.3, 126.3, 126.3, 126.0, 125.2, 125.1, 84.3, 53.3, 52.9, 52.5, 25.6, 24.9, 24.9, 15.6, 15.5; **HRMS** (pos. ESI): *m/z* calcd for $C_{80}H_{73}O_{10}B_2$ 1215.5384 M+H]$^+$, found 1215.5378.

Compound (*S,S*)^3-13

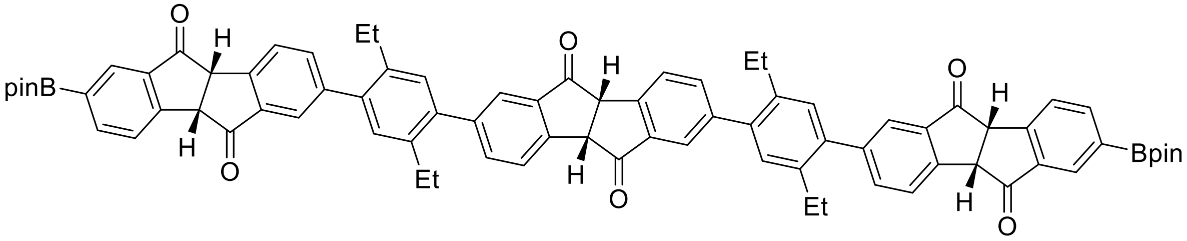

(*S*,*S*)^3-13 was synthesized using the same procedure described above for **(*R*,*R*)^3-13** using trimer **(*S,S*)^3-12** (334 mg, 298 µmol), bis(pinacolato)diboron (167 mg, 658 µmol, 2.2 eq.), KOAc (177 mg, 1.80 mmol, 6.0 eq.) and $PdCl_2$(dppf) (13.3 mg, 18.2 µmol, 6 mol%) and 1,4-dioxane (9 mL) at 85 °C for 3 d. to yield the title compound as a beige solid (382 mg, 298 µmol, quant.).

^{1}H NMR (500 MHz, $CDCl_3$): δ 8.22 (s, 2H), 8.11 (dd, *J* = 7.7, 1.1 Hz, 2H), 7.99 (d, *J* = 7.9 Hz, 2H), 7.98–7.95 (m, 2H), 7.95–7.92 (m, 2H), 7.73 (dd, *J* = 1.8, 0.7 Hz, 2H), 7.71–7.65 (m, 6H), 7.05 (s, 2H), 7.05 (s, 2H), 4.52 (s, 2H), 4.48 (dd, *J* = 5.7, 1.0 Hz, 2H), 4.46 (dd, *J* = 5.7, 0.9 Hz, 2H), 2.52 (q, *J* = 7.5 Hz, 4H), 2.50 (q, *J* = 7.5 Hz, 4H), 1.33 (s, 12H), 1.32 (s, 12H), 1.05 (t, *J* = 7.5 Hz, 6H), 1.02 (t, *J* = 7.5 Hz, 6H); **^{13}C NMR** (126 MHz, $CDCl_3$)[13]:) δ 201.6, 201.5, 201.4, 152.4, 148.6, 148.5, 143.1, 143.0, 142.0, 139.8, 139.7, 139.0, 138.9, 137.2, 137.1, 135.1, 135.0, 134.5, 131.8, 130.3, 126.30, 126.27, 126.0, 125.2, 125.1, 84.3, 53.3, 52.9, 52.5, 25.6, 25.0, 24.9, 15.6; **HRMS** (pos. ESI): *m/z* calcd for $C_{80}H_{73}O_{10}B_2$ 1215.5384 M+H]$^+$, found 1215.5396.

---

[12] Some ^{13}C signals are not resolved due to chemical similarity. The carbon atom connected to boron is not visible due to coupling with ^{10}B and ^{11}B.
[13] Some ^{13}C signals are not resolved due to chemical similarity. The carbon atom connected to boron is not visible due to coupling with ^{10}B and ^{11}B.

2.4 Synthesis of Cyclic Compounds

Compound (*R,R*)^6-14

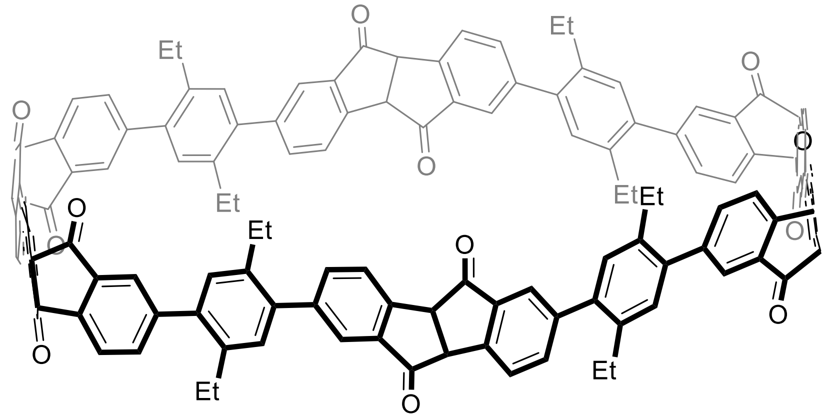

The reaction was performed following the methodology by Jasti and coworkers.[12]

Trimer **(*R*,*R*)³-13** (368 mg, 303 µmol), $Pd(PPh_3)_2Cl_2$ (43.1 mg, 61.4 µmol, 20 mol%), $B(OH)_3$ (93.5 mg, 1.51 mmol, 5 eq.) and KF (72.0 mg, 1.24 mmol, 4 eq.) were placed in a 2 L round-bottom flask, and THF (1.5 L, ACROS-ORGANICS, 99.6% ACS reagent, containing 250 ppm BHT as stabilizer) was added. The flask was heated to 40 °C, H_2O (150 mL) was added, and the reaction was stirred at 40 °C for 23 h. After cooling to rt, the THF was removed under reduced pressure. CH_2Cl_2 (150 mL) was added to the aq. residue, the layers were separated, and the aq. layer was extracted with CH_2Cl_2 (2 × 50 mL). The combined organic layers were dried over Na_2SO_4, and the solvent was removed under reduced pressure. The crude product was purified by column chromatography[14] (SiO_2, CH_2Cl_2 to CH_2Cl_2/acetone 40/1 to CH_2Cl_2/acetone 25/1) to yield **(*R*,*R*)^6-14** as a yellowish solid (92 mg, 48 µmol, 32%).

***R*f** 0.30 (CH_2Cl_2/acetone: 25/1); **m.p.** >350 °C, **^{1}H NMR** (500 MHz, $CDCl_3$): δ 8.03 (d, *J* = 8.3 Hz, 4H), 8.00–7.92 (m, 16H), 7.71–7.66 (m, 16H), 7.02 (s, 4H), 7.01 (s, 4H), 4.55–4.49 (m, 12H), 2.52–2.44 (m, 16H), 1.01 (t, *J* = 7.5 Hz, 12H), 1.00 (t, *J* = 7.5 Hz, 12H); **^{13}C NMR** (126 MHz, $CDCl_3$)[15]: δ 201.6, 201.4, 201.3, 149.5, 148.5, 148.3, 143.2, 143.1, 140.8, 139.8, 139.7, 138.9, 137.2, 137.2, 135.9, 135.0, 134.9, 134.8, 130.2, 127.3, 126.2, 126.2, 125.2, 125.1, 122.8, 52.9, 52.8, 52.8, 25.6, 15.5; **HRMS** (pos. ESI): *m*/*z* calcd for $C_{136}H_{97}O_{12}$ 1921.6975 $[M+H]^+$, found 1921.6979; **UV/Vis** (CH_2Cl_2): λ = 252 nm ($\varepsilon = 3.21 \cdot 10^5$ M^{-1} cm^{-1}); **ATR FT-IR** $\tilde{\nu}$ = 2966 (vw), 1707 (ms), 1473 (mw), 1115 (mw), 853 (mw).

---

[14] The size of the column was 3.5 cm diameter × 19 cm length of the SiO_2 layer. The crude product was applied on the column dissolved in CH_2Cl_2. The product was eluted with CH_2Cl_2/acetone: 25/1 over 50 fractions à 20 mL.

[15] Two signals in the aromatic region missing, likely due to signal overlap.

Compound (*S*,*S*)^6-14

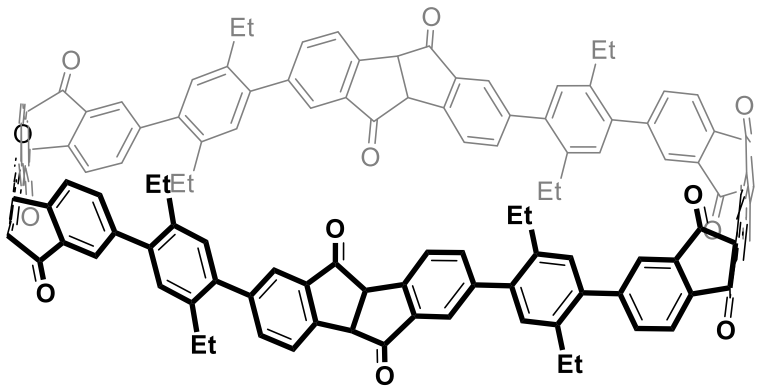

(*S*,*S*)^6-14 was synthesized using the same procedure described above for **(*R*,*R*)^6-14** using trimer **(*S,S*)^3-13** (349 mg, 287 µmol), $Pd(PPh_3)_2Cl_2$ (40.6 mg, 57.8 µmol, 20 mol%), $B(OH)_3$ (90.0 mg, 1.46 mmol, 5 eq.), KF (68.0 mg, 1.17 mmol, 4 eq.), THF (1.44 L, ACROS-ORGANICS, 99.6% ACS reagent, containing 250 ppm BHT as stabilizer) and H_2O (146 mL) to yield the title compound as a yellowish powder (71 mg, 37 µmol, 26%). ***R*$_f$** 0.30 (CH_2Cl_2/acetone: 25/1); **^{1}H NMR** (500 MHz, $CDCl_3$): δ 8.03 (d, *J* = 8.3 Hz, 4H), 7.99–7.92 (m, 16H), 7.70–7.66 (m, 16H), 7.02 (s, 4H), 7.01 (s, 4H), 4.54–4.49 (m, 12H), 2.52–2.44 (m, 16H), 1.00 (t, *J* = 7.5 Hz, 12H), 1.00 (t, *J* = 7.5 Hz, 12H); **^{13}C NMR** (126 MHz, $CDCl_3$)[16]: δ 201.7, 201.5, 201.3, 149.6, 148.5, 148.3, 143.2, 143.1, 140.8, 139.8, 139.7, 138.9, 137.24, 137.21, 135.9, 135.1, 135.0, 134.9, 130.2, 127.4, 126.3, 126.2, 125.2, 125.1, 122.8, 52.9, 52.84, 52.81, 25.6, 15.5; **HRMS** (pos. ESI): *m*/*z* calcd for $C_{136}H_{97}O_{12}$ 1921.6975 [M+H]$^+$, found 1921.6977.

Compound (*R*,*R*)^6-(+)-16

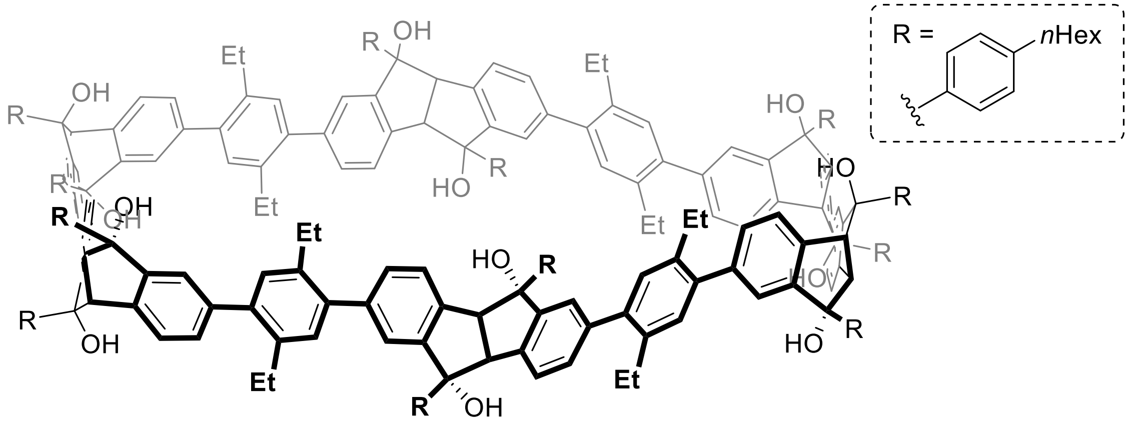

In an oven-dried Schlenk tube, anh. $CeCl_3$ (205 mg, 832 µmol, 20 eq.) was dried at 135 °C and 1 × 10^{-3} mbar for 2.5 h. The tube was backfilled with argon while hot, allowed to cool to rt, and anh. THF (2.5 mL) was added. The resulting white suspension was stirred at rt over night. For the preparation of the Grignard reagent a dry Schlenk tube was charged with Mg shavings (54.0 mg, 2.22 mmol) and heated under vacuum with a heat gun for 5 min. After cooling to rt 1-bromo-4-hexylbenzene (**15**, 0.43 mL, 2.10 mmol) and anh. THF (2.1 mL) were added, and the Grignard reaction was started under gentle heating. The mixture was stirred until all Mg was consumed, and the Grignard solution was titrated using a method by Peters *et al*.[13] to yield a concentration of 0.82 mol L^{-1}. The $CeCl_3$ suspension was cooled to 0 °C, the freshly prepared

---

[16] Two signals in the aromatic region missing, likely due to signal overlap.

Grignard reagent (0.82 M in THF, 0.96 mL, 787 µmol, 19.3 eq.) was added, and the mixture was stirred at 0 °C for 1 h. **(*R*,*R*)^6-14** (78.5 mg, 40.8 µmol, dried at 60 °C and 1×10^{-3} mbar over night) was added in one portion, and the resulting yellow mixture was stirred at 0 °C for 6 h, warmed to rt and stirred at rt for a further 19 h. The reaction mixture was quenched with 10% aq. HOAc (5 mL), CH_2Cl_2 (20 mL) was added, and the aq. layer was extracted with CH_2Cl_2 (3 × 5 mL). The combined organic layers were washed with aq. sat. $NaHCO_3$ (10 mL), dried over Na_2SO_4, and the solvent was removed under reduced pressure. Column chromatography (SiO_2, cyclohexane/CH_2Cl_2/EtOAc: 10/1/1) yielded the title compound as a yellowish solid (129 mg, 33.3 µmol, 82%).

***R*$_f$** 0.39 (cyclohexane/CH_2Cl_2/EtOAc: 10/1/1); **m.p.** 205 °C (decomposition), **^{1}H NMR** (500 MHz, CD_2Cl_2): δ 7.54 (dd, *J* = 8.2, 1.7 Hz, 4H), 7.41–7.35 (m, 26H), 7.35–7.27 (m, 20H), 7.26–7.15 (m, 28H), 7.07–7.03 (m, 14H), 4.42 (s, 4H), 4.39–4.34 (m, 8H), 2.93 (s, 4H, O*H*), 2.89 (s, 4H, O*H*), 2.75 (s, 4H, O*H*), 2.70–2.59 (m, 24H), 2.54–2.42 (m, 16H), 1.71–1.57 (m, 24H), 1.42–1.27 (m, 72H), 0.98 (t, *J* = 7.5 Hz, 12H), 0.98 (t, *J* = 7.5 Hz, 12H), 0.93–0.87 (m, 36H); **^{13}C NMR** (126 MHz, CD_2Cl_2)[17]: δ 150.7, 150.0, 149.7, 144.1, 144.0, 143.9, 142.7, 142.3, 142.28, 142.25, 141.7, 140.9, 140.8, 139.9, 139.2, 139.09, 139.07, 133.4, 130.53, 130.46, 130.0, 128.65, 128.56, 127.5, 127.0, 126.6, 126.4, 126.2, 126.1, 126.01, 125.96, 124.3, 115.3, 84.83, 84.79, 84.6, 63.1, 62.7, 62.6, 36.0, 35.93, 35.90, 32.2, 32.1, 31.91, 31.89, 31.88, 29.5, 29.45, 29.42, 26.1, 26.0, 23.04, 23.01, 15.8, 14.27, 14.25; **HRMS** (pos. ESI): *m*/*z* calcd for $C_{277}{}^{13}C_3H_{312}O_{12}Na$ 3892.3797 $[M+Na]^+$, found 3892.3787; $[\alpha]_D^{25}$ = +82.6 (*c* = 1.03, $CHCl_3$); **UV/Vis** (CH_2Cl_2): λ = 264 nm ($\varepsilon = 17.1 \cdot 10^4$ M^{-1} cm^{-1}); **ATR FT-IR** $\tilde{\nu}$ = 2957 (w), 2926 (w), 2853 (w), 1457 (w), 1104 (w), 1053 (w), 1019 (w), 802 (mw).

[17] Not all signals can be identified and many signals overlap due to chemical similarity.

Compound (*S*,*S*)^6-(−)-16

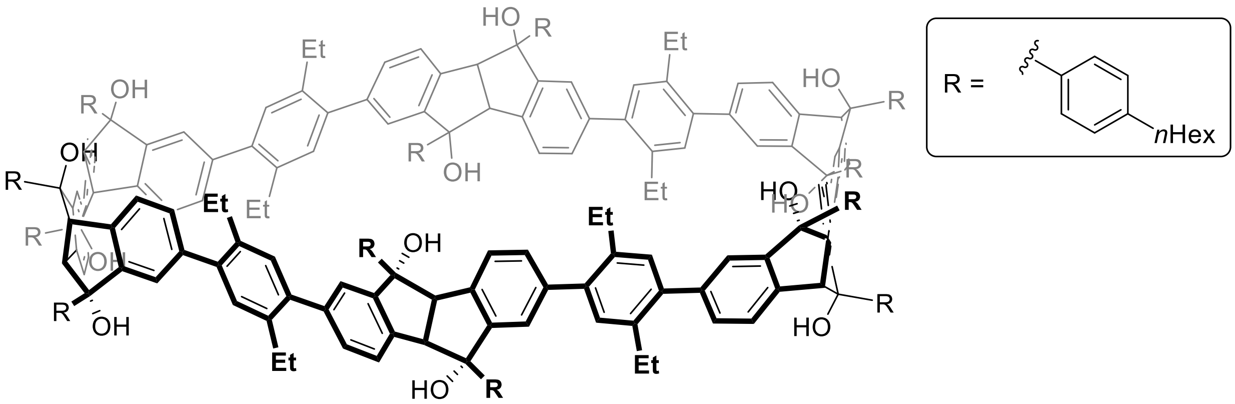

(*S*,*S*)^6-(−)16 was synthesized using the same procedure described above for **(*R*,*R*)^6-(+)-16** using $CeCl_3$ (130 mg, 527 µmol, 20 eq.), THF (2.5 mL) and freshly prepared Grignard reagent (0.82 M in THF, 0.60 mL, 492 µmol, 18.9 eq.) to yield the title compound as a yellowish powder (88 mg, 23 µmol, 87%).

***R*$_f$** 0.39 (cyclohexane/CH_2Cl_2/EtOAc: 10/1/1); **^{1}H NMR** (500 MHz, CD_2Cl_2): δ 7.53 (dd, *J* = 8.2, 1. Hz, 4H), 7.41–7.35 (m, 26H), 7.35–7.27 (m, 20H), 7.26–7.15 (m, 28H), 7.06–7.03 (m, 14H), 4.41 (s, 4H), 4.38–4.33 (m, 8H), 2.92 (s, 4H), 2.88 (s, 4H), 2.74 (s, 4H), 2.69–2.57 (m, 24H), 2.54–2.43 (m, 16H), 1.71–1.58 (m, 24H), 1.43–1.26 (m, 72H), 0.98 (t, *J* = 7.5 Hz, 12H), 0.98 (t, *J* = 7.5 Hz, 12H), 0.91–0.87 (m, 36H); **^{13}C NMR** (126 MHz, CD_2Cl_2 δ 150.76, 150.0, 149.75, 144.16, 144.0, 143.9, 142.75, 142.3, 142.27, 142.25, 141.7, 140.9, 140.8, 139.9, 139.2, 139.11, 139.09, 133.4, 130.54, 130.48, 130.0, 128.66, 128.57, 127.5, 127.0, 126.6, 126.59, 126.46, 126.2, 126.1, 126.03, 125.98, 124.3, 115.3, 84.84, 84.80, 84.7, 63.1, 62.7, 62.7, 36.0, 35.93, 35.91, 32.2, 32.1, 31.91, 31.90, 31.88, 29.50, 29.45, 29.43, 26.07, 26.05, 23.04, 23.01, 15.8, 14.27, 14.25; **HRMS** (neg. ESI): *m*/*z* calcd for $C_{279}{}^{13}CH_{312}O_{12}Cl$ 3902.3531 $[M+Cl]^-$, found 3902.3614; $[\alpha]_D^{25}$ = −76.8 (*c* = 0.98, $CHCl_3$);

Conjugated nanohoop (+)-1

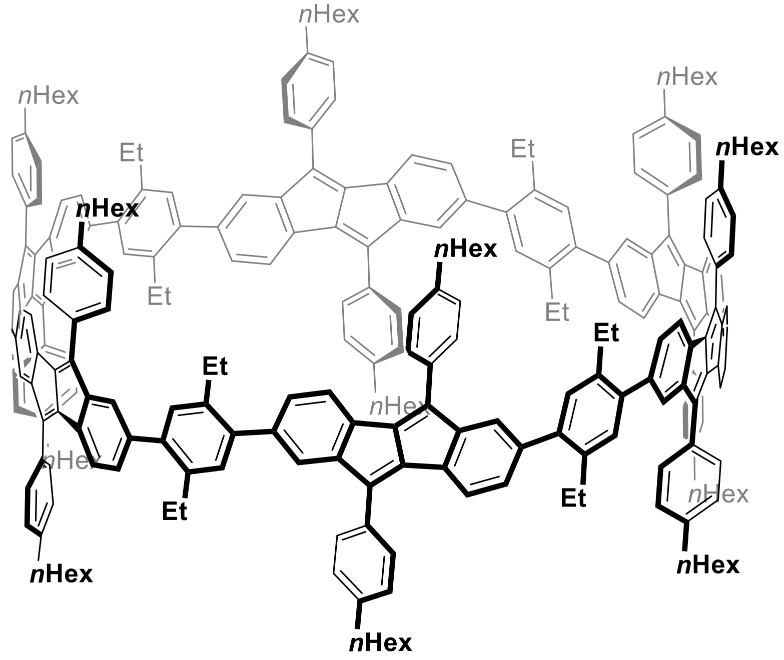

In an oven-dried Schlenk tube ***(R,R)*^{6}-(+)-16** (24.57 mg, 6.33 µmol) and Burgess's reagent (**17**, 72.4 mg, 304 µmol, 48 eq.) were dissolved in anh. degassed 1,4-dioxane (1.9 mL). The tube was placed into an oil bath, preheated to 50 °C, and the mixture was stirred at that temperature for 40 min. After 3 min the solution had already turned dark red. After cooling to rt, the reaction was quenched with sat. aq. $NaHCO_3$ (1 mL) und H_2O (3 mL). The layers were separated, and the aq. layer was extracted with $CHCl_3$ (2 × 2 mL). The combined org. layers were dried over Na_2SO_4, and the solvent was removed under reduced pressure. The crude yellow-brown solid residue was washed with MeOH (3 × 4 mL) and *n*-pentane (3 × 4 mL). **(+)-1** was obtained as a red solid (19.8 mg, 5.42 µmol, 86%).

***R*$_f$** 0.24 (cyclohexane/CH_2Cl_2: 5/1), 0.77 (cyclohexane/CH_2Cl_2: 2/1); **^{1}H NMR** (500 MHz, $CDCl_3$): δ 7.61–7.55 (m, 24H), 7.35–7.31 (m, 8H), 7.31–7.27 (m, 20H), 7.23[18] (d, J = 7.8 Hz, 4H), 7.21 (d, J = 8.0 Hz, 4H), 7.19 (d, J = 7.8 Hz, 4H), 7.08 (dd, J = 8.0, 1.6 Hz, 4H), 7.04 (d, J = 1.5 Hz, 4H), 7.02 (d, J = 1.5 Hz, 4H), 6.98 (s, 4H), 6.97 (s, 4H), 6.80 (dd, J = 7.8, 1.5 Hz, 4H), 6.76 (dd, J = 7.8, 1.5 Hz, 4H), 2.74–2.63 (m, 24H), 2.59–2.49 (m, 16H), 1.74–1.62 (m, 24H), 1.44–1.28 (m, 72H), 1.04 (t, J = 7.5 Hz, 12H), 1.01 (t, J = 7.5 Hz, 12H), 0.93–0.87 (m, 36H); **^{13}C NMR** (126 MHz, $CDCl_3$)[19]: δ 150.5, 149.7, 149.6, 144.1, 144.0, 143.3, 143.0, 142.8, 141.9, 141.8, 141.0, 140.9, 140.8, 140.6, 140.1, 139.1, 139.0, 134.1, 134.0, 131.4, 130.5, 130.3, 128.83, 128.80, 128.69, 128.66, 128.64, 128.4, 126.9, 124.8, 123.9, 123.7, 122.3, 121.7, 121.5, 121.0, 36.2, 36.1, 31.9, 31.9, 31.5, 31.4, 29.4, 29.2, 26.1, 26.1, 22.8, 22.8, 15.9, 15.9, 14.3; **HRMS** (pos. ESI): *m*/*z* calcd for $C_{277}{}^{13}C_3H_{288}$ 3653.2631 $[M]^{+\bullet}$, found 3653.2625; $[\boldsymbol{\alpha}]_{\mathbf{D}}^{\mathbf{25}}$ = +5.4 (c = 0.44, $CHCl_3$); **UV/Vis** (CH_2Cl_2): λ (ε in M^{-1} cm^{-1}) = 270 (2.03 · 10^5), 322 (2.73 · 10^5), 441 (1.31 · 10^5), 464 nm (1.34 · 10^5); **ATR FT-IR** $\tilde{\nu}$ = 2952 (w), 2923 (w), 2852 (w), 1505 (w), 1435 (w), 1180 (w), 820 (mw).

[18] Overlapping with the signal at 7.21 ppm. Spin systems were unambiguously assigned by COSY.
[19] Many signals overlap due to chemical similarity (e.g. hexylphenyl groups). Due to a relatively low solubility not all signals could be identified.

Conjugated nanohoop (−)-1

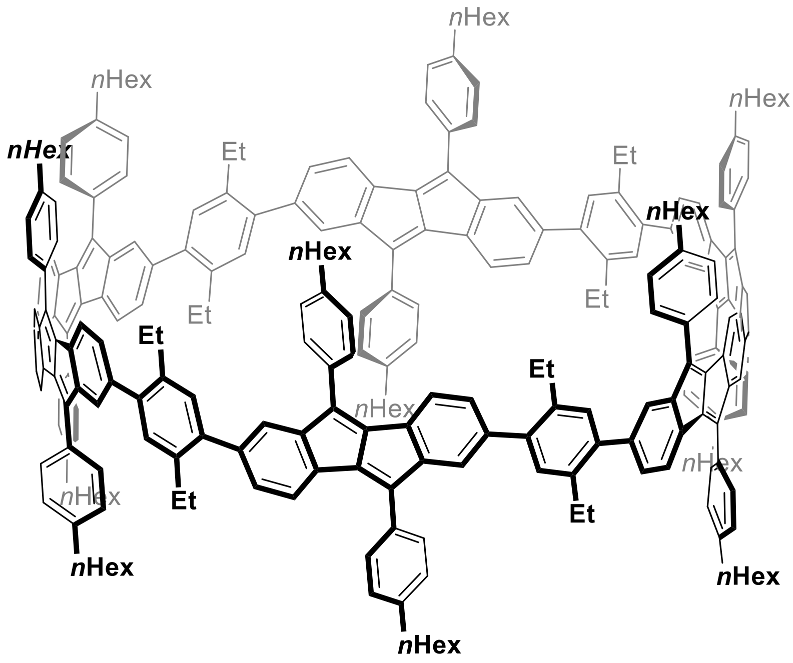

(−)-1 was synthesized using the same procedure described above for **(+)-1** using **(*S*,*S*)[6]-(−)-16** (21.0 mg, 5.43 µmol) and Burgess's reagent (**17**, 65.0 mg, 273 µmol, 50 eq.) and anh. degassed 1,4-dioxane (1.6 mL) to yield the title compound as a red solid (17.8 mg, 4.87 µmol, 90%).

***R*f** 0.24 (cyclohexane/CH_2Cl_2: 5/1), 0.77 (cyclohexane/CH_2Cl_2: 2/1); **^{1}H NMR** (400 MHz, $CDCl_3$): δ 7.61–7.56 (m, 24H), 7.34–7.31 (m, 8H), 7.31–7.26 (m, 20H), 7.23[20] (d, *J* = 7.8 Hz, 4H), 7.21 (d, *J* = 8.0 Hz, 4H), 7.19 (d, *J* = 8.1 Hz, 4H), 7.08 (dd, *J* = 8.0, 1.6 Hz, 4H), 7.04 (d, *J* = 1.5 Hz, 4H), 7.02 (d, *J* = 1.5 Hz, 4H), 6.98 (s, 4H), 6.97 (s, 4H), 6.80 (dd, *J* = 7.8, 1.5 Hz, 4H), 6.76 (dd, *J* = 7.8, 1.5 Hz, 4H), 2.75–2.62 (m, 24H), 2.61–2.48 (m, 16H), 1.76–1.61 (m, 24H), 1.47–1.25 (m, 72H), 1.04 (t, *J* = 7.5 Hz, 12H), 1.01 (t, *J* = 7.5 Hz, 12H), 0.93–0.87 (m, 36H); **^{13}C NMR** (101 MHz, $CDCl_3$)[21]: δ 150.5, 149.7, 149.6, 144.1, 144.0, 143.3, 143.0, 142.8, 141.9, 141.8, 141.0, 140.9, 140.8, 140.6, 140.1, 139.1, 139.0, 134.10, 134.0, 131.4, 130.5, 130.3, 128.83, 128.80, 128.70, 128.67, 128.64, 128.45, 124.8, 123.9, 123.7, 122.3, 121.7, 121.5, 121.1, 36.2, 36.1, 31.90, 31.87, 31.5, 31.4, 29.4, 29.2, 26.08, 26.07, 22.78, 22.77, 15.91, 15.86, 14.3; **HRMS** (pos. ESI): *m/z* calcd for $C_{279}{}^{13}CH_{289}$ 3652.2642 [M+H]$^+$, found 3652.2695 $[\alpha]_D^{25}$ = −2.4 (*c* = 0.42, $CHCl_3$).

[20] Overlapping with the signal at 7.21 ppm. Spin systems were unambiguously assigned by COSY.
[21] Many signals overlap due to chemical similarity (e.g. hexylphenyl groups). Due to a relatively low solubility not all signals could be identified.

3. NMR Spectra

3.1 NMR Spectra

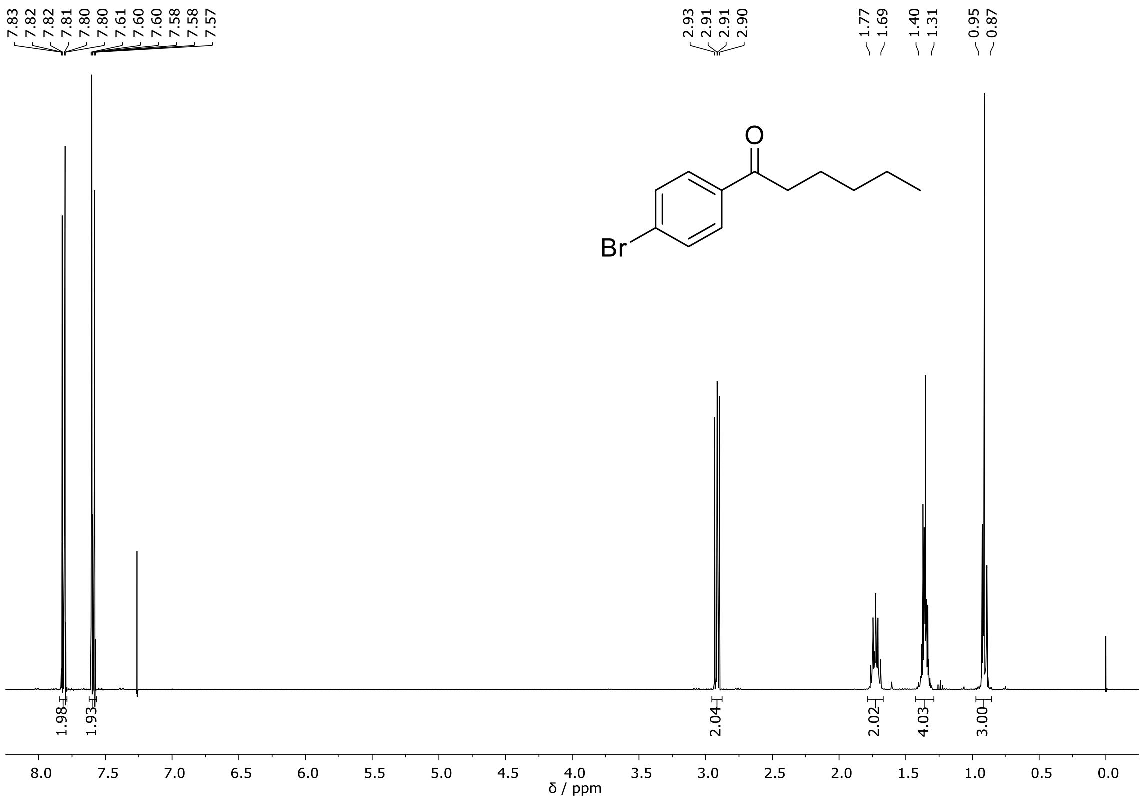

Figure S1. ^{1}H NMR spectrum of **S1** in $CDCl_3$ (400 MHz).

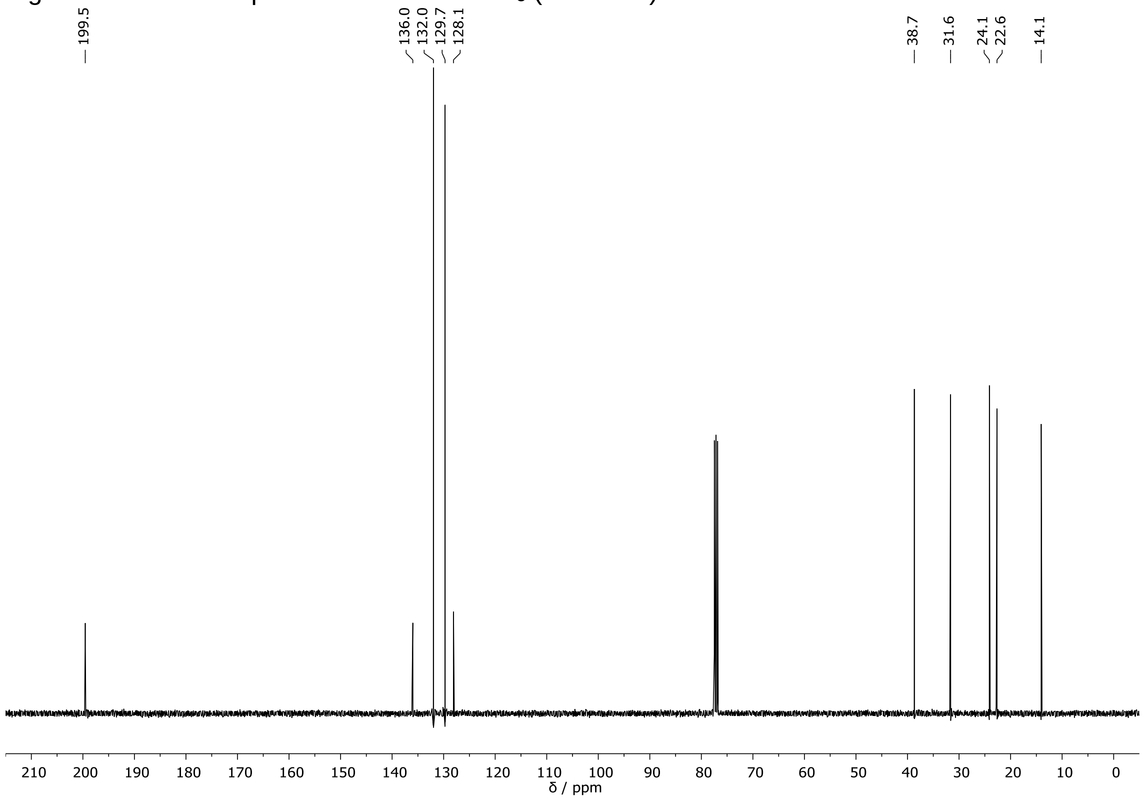

Figure S2. ^{13}C NMR spectrum of **S1** in $CDCl_3$ (101 MHz).

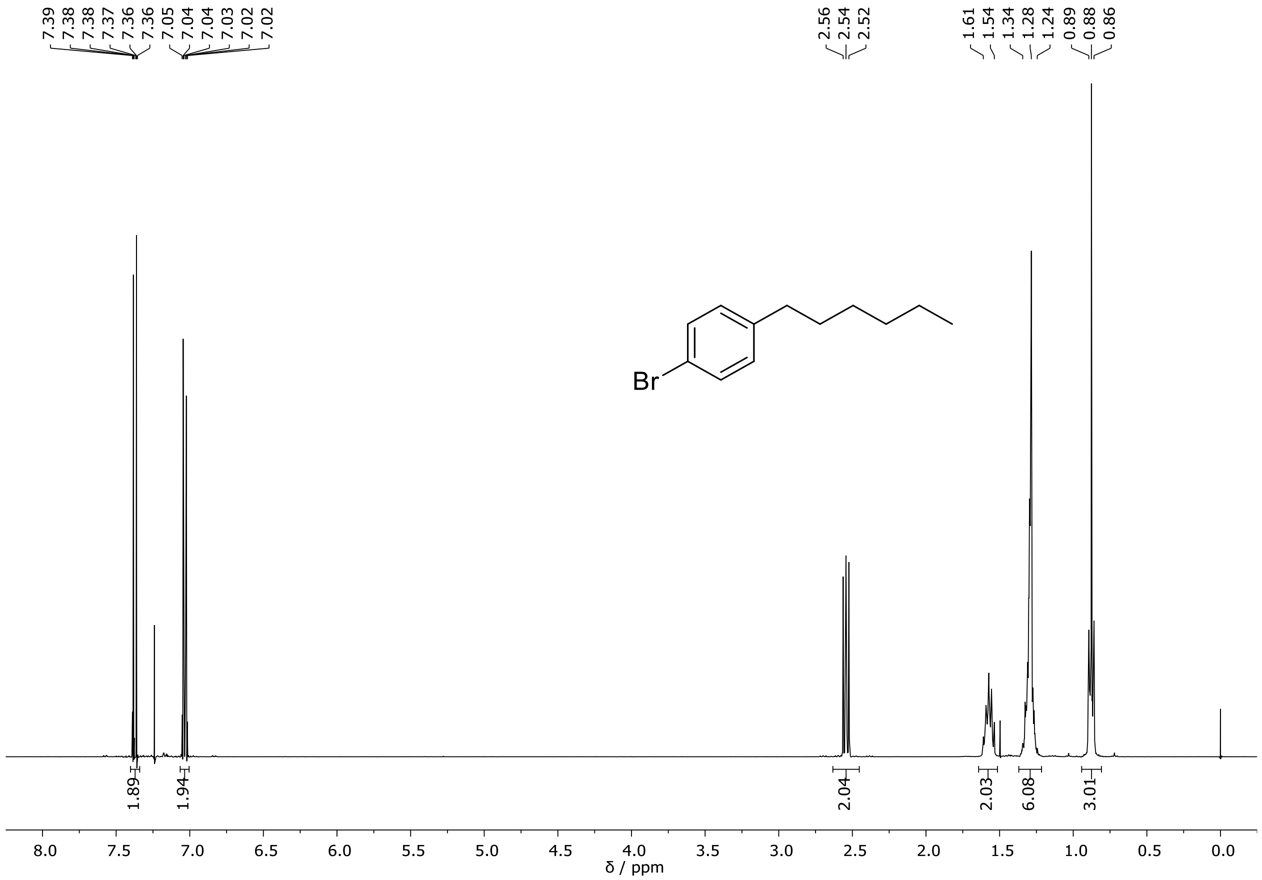

Figure S3. ^{1}H NMR spectrum of **15** in $CDCl_3$ (400 MHz).

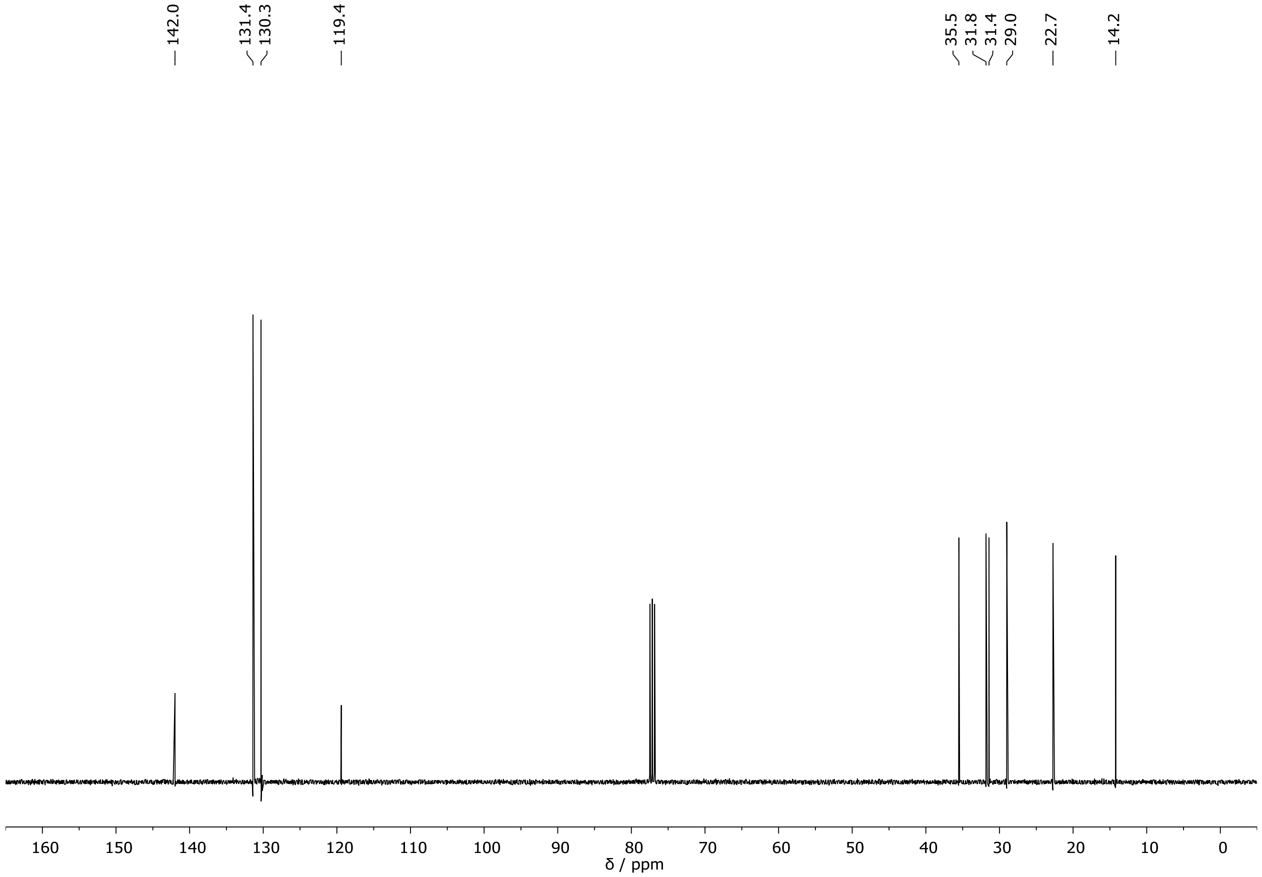

Figure S4. ^{13}C NMR spectrum of **15** in $CDCl_3$ (101 MHz).

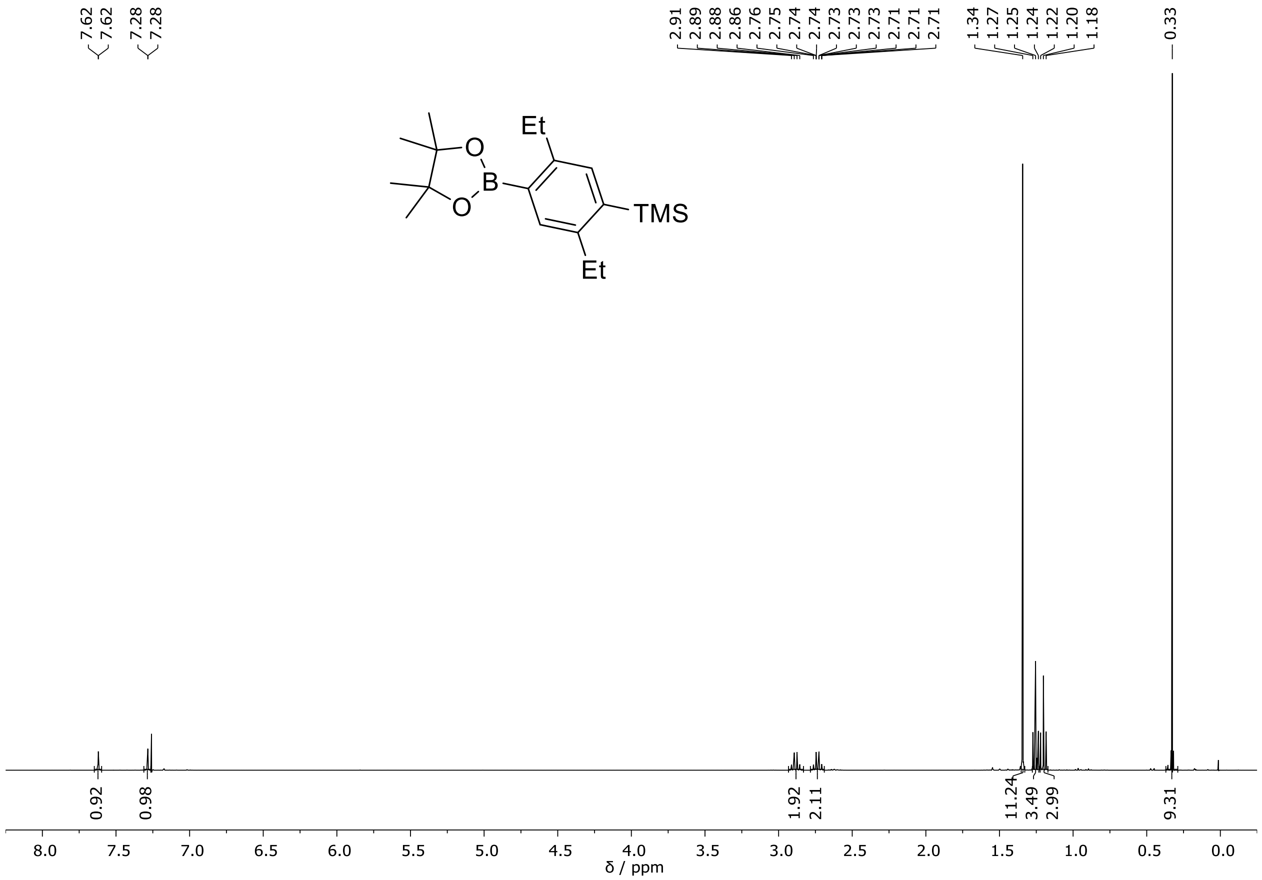

Figure S5. ^{1}H NMR spectrum of **8** in $CDCl_3$ (400 MHz).

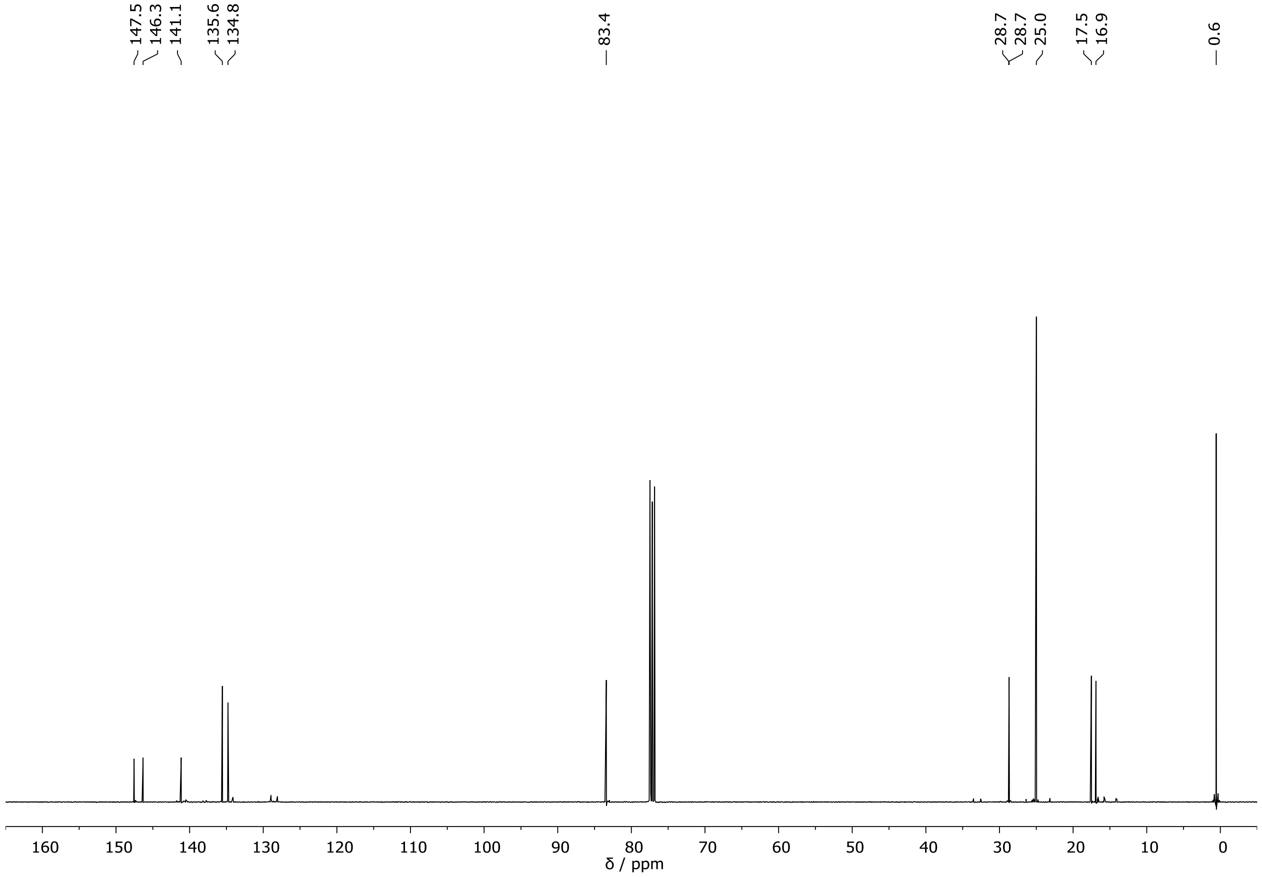

Figure S6. ^{13}C NMR spectrum of **8** in $CDCl_3$ (101 MHz).

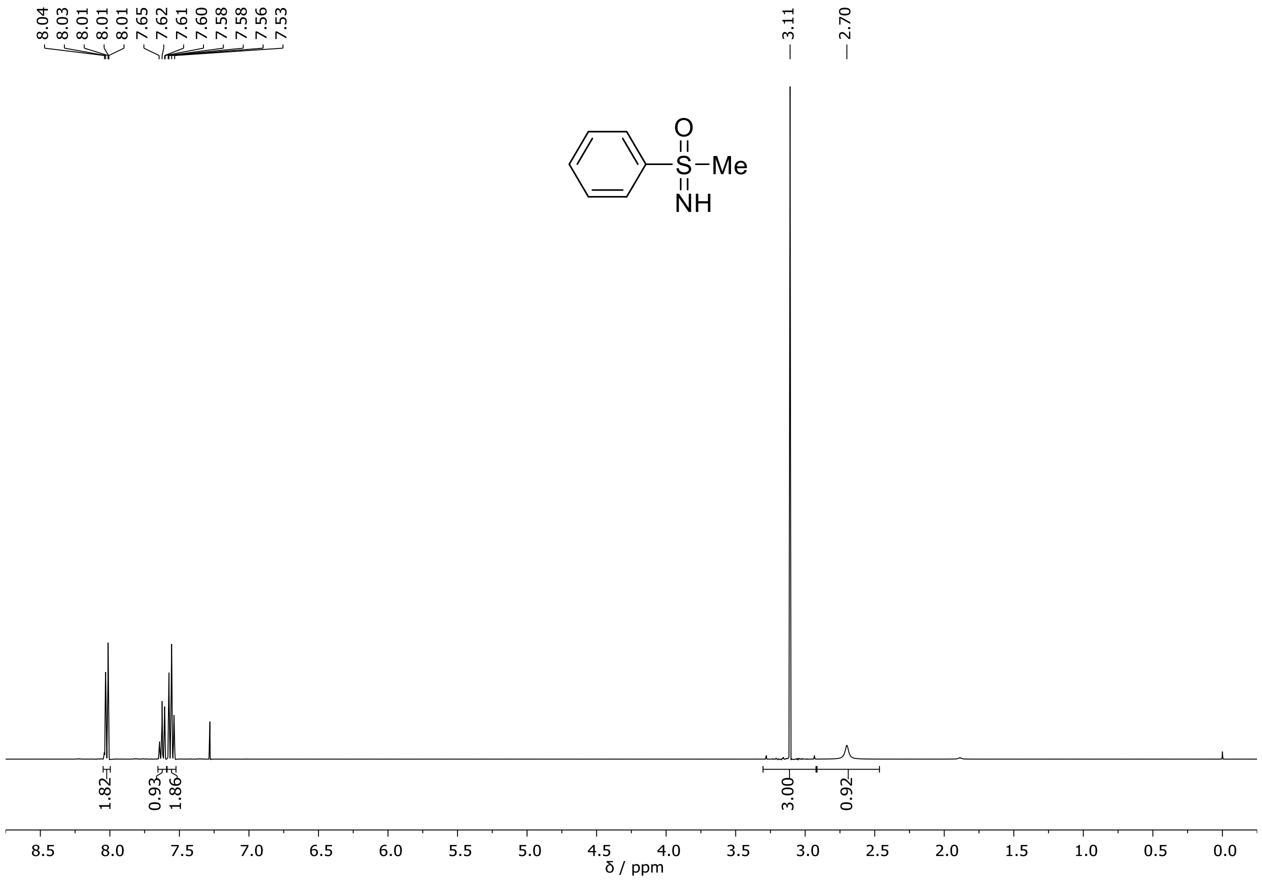

Figure S7. ^{1}H NMR spectrum of ***rac*-S2** in $CDCl_3$ (400 MHz).

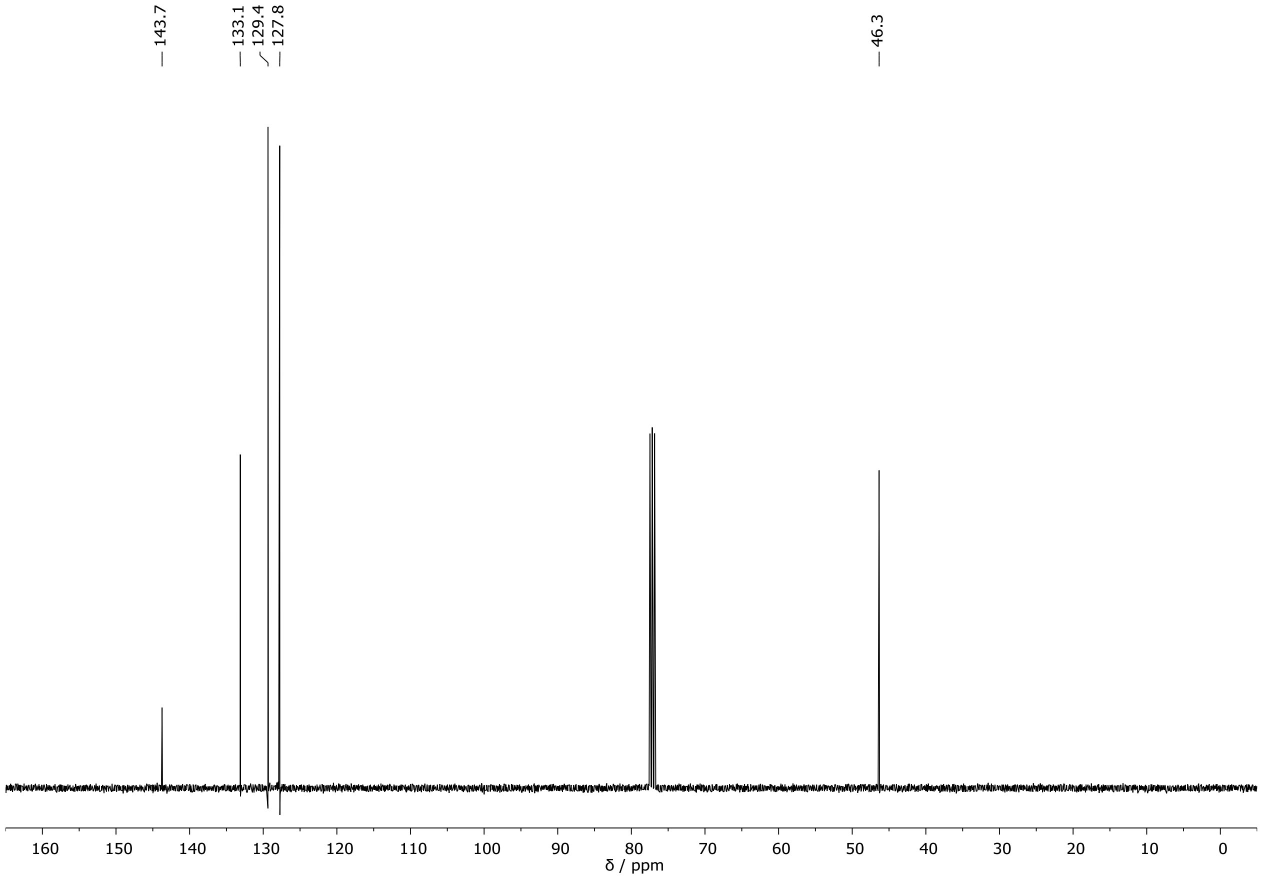

Figure S8. ^{13}C NMR spectrum of ***rac*-S2** in $CDCl_3$ (101 MHz).

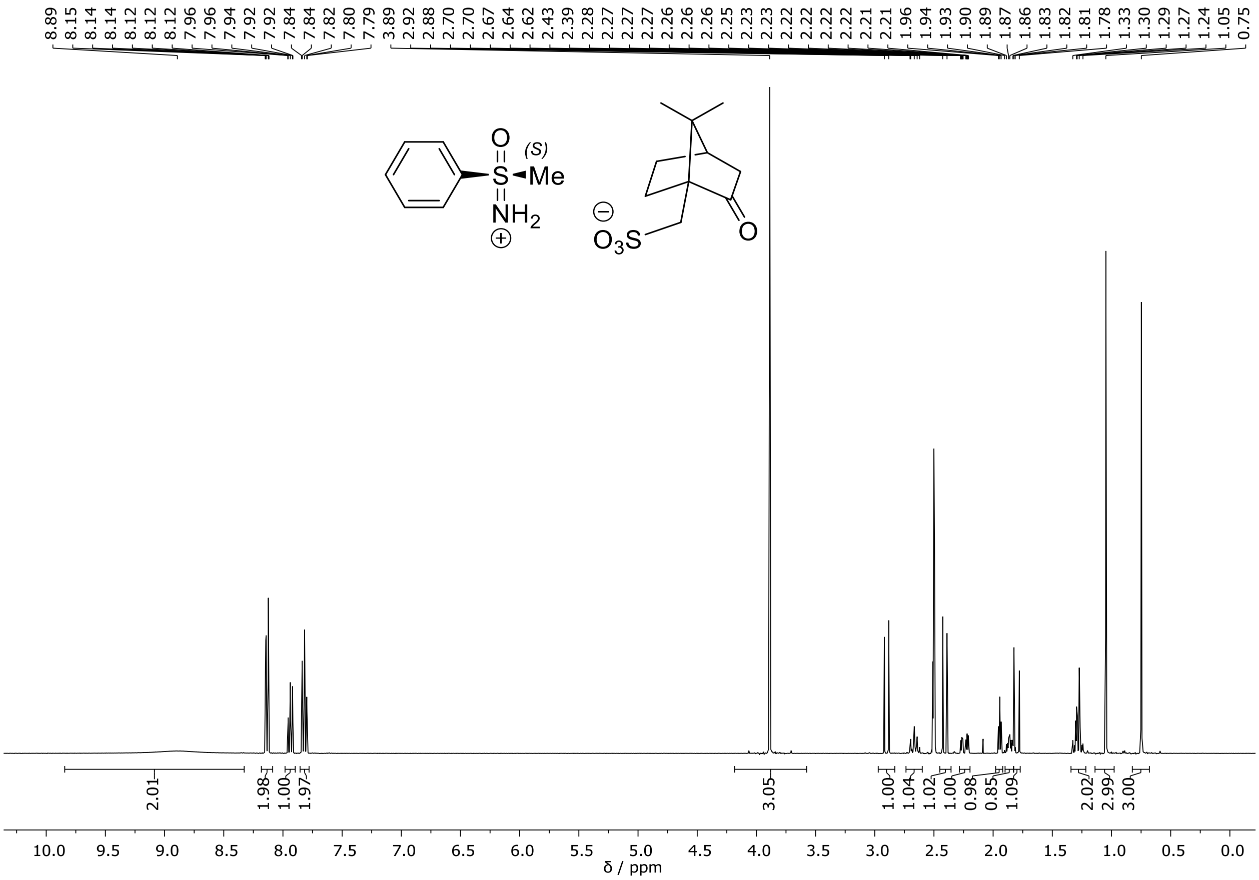

Figure S9. ^{1}H NMR spectrum of **(*S*)-S3** in DMSO-d_6 (400 MHz).

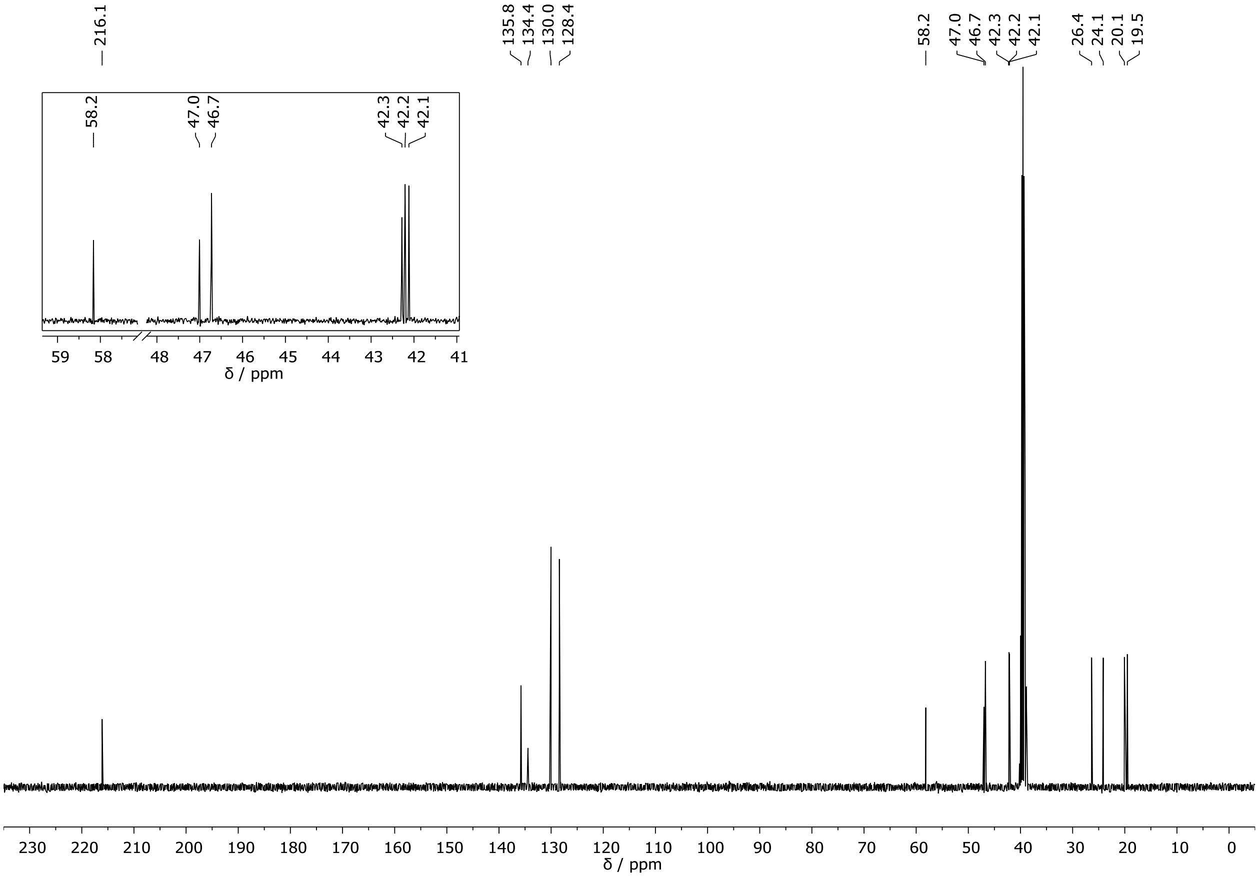

Figure S10. ^{13}C NMR spectrum of **(*S*)-S3** in DMSO-d_6 (101 MHz).

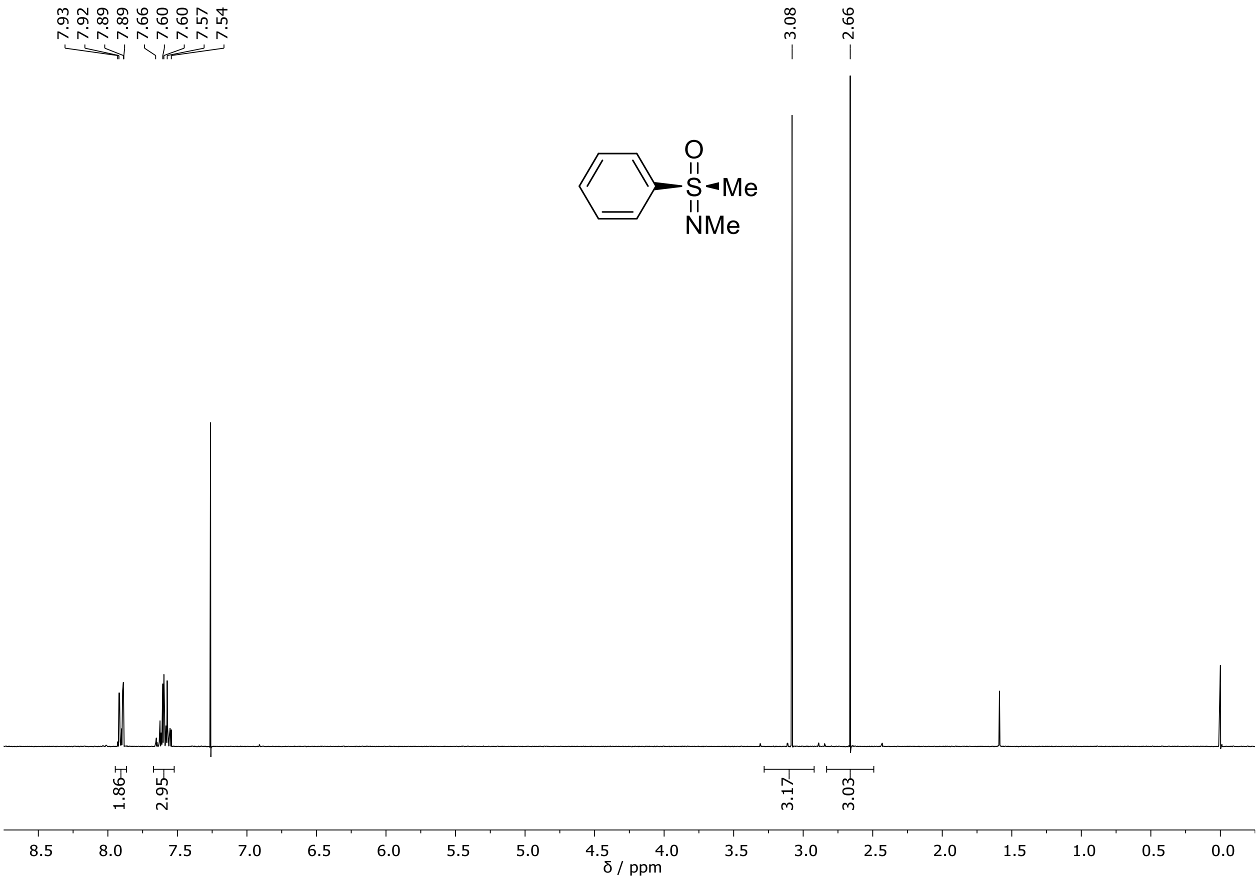

Figure S11. ^{1}H NMR spectrum of **(*S*)-4** in $CDCl_3$ (300 MHz).

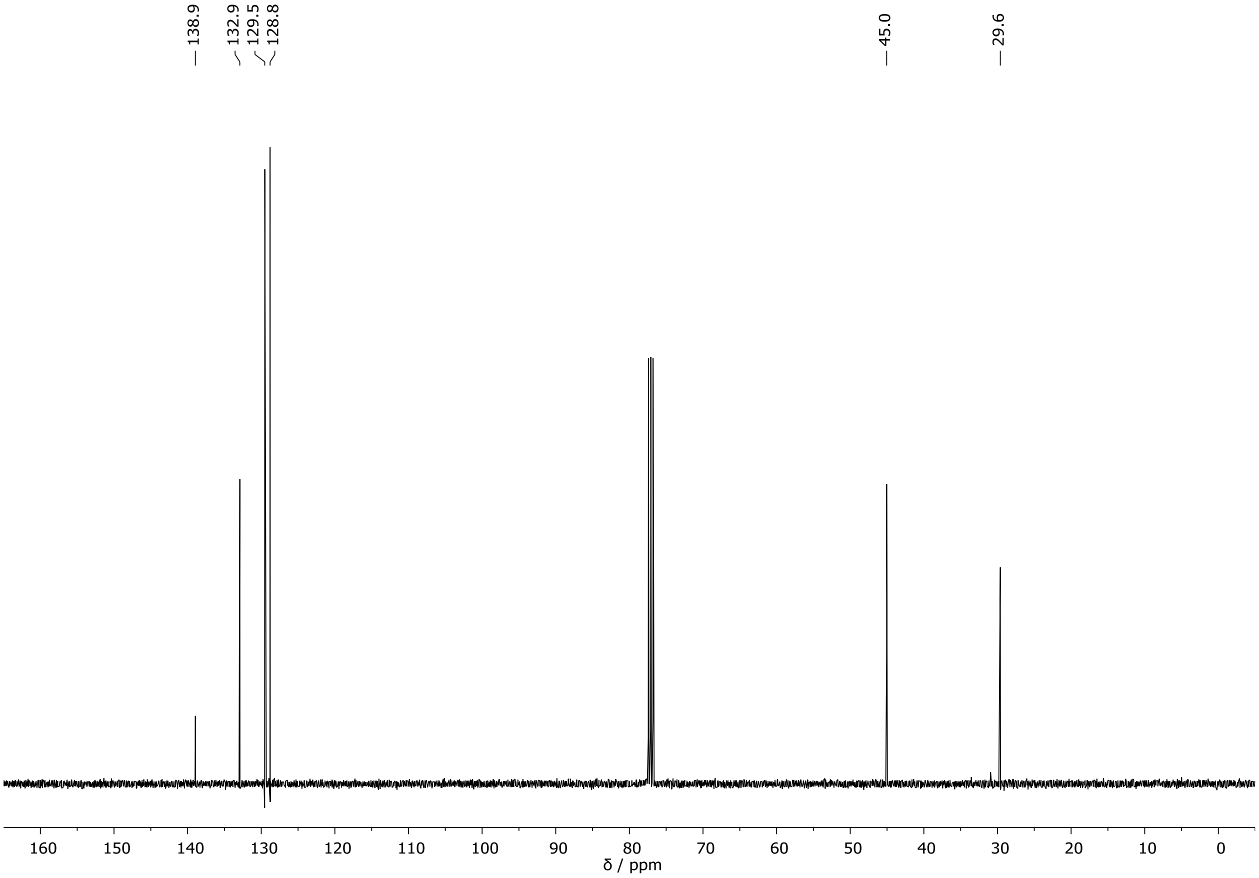

Figure S12. ^{13}C NMR spectrum of **(*S*)-4** in $CDCl_3$ (101 MHz).

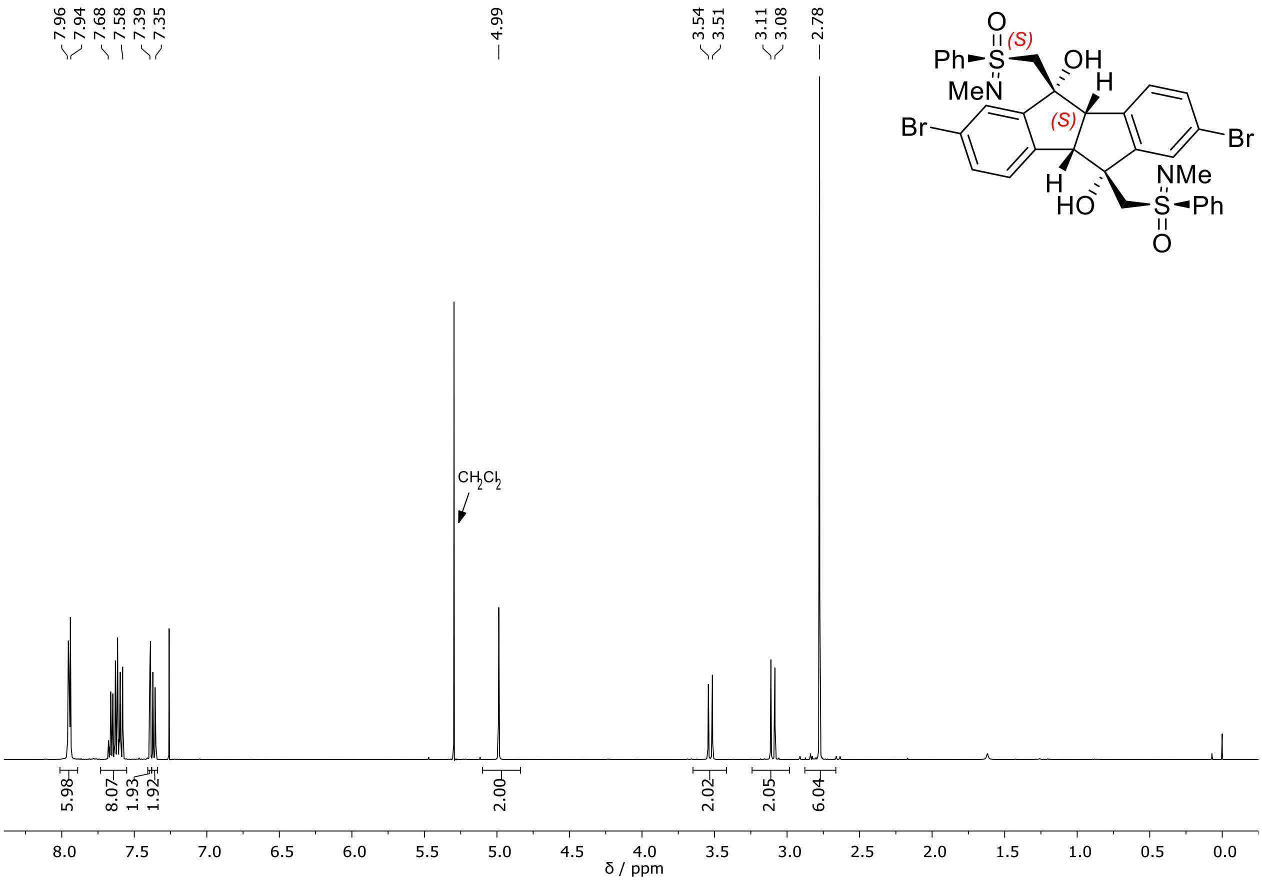

Figure S13. ^{1}H NMR spectrum of **6** in $CDCl_3$ (500 MHz). Contains residual CH_2Cl_2.

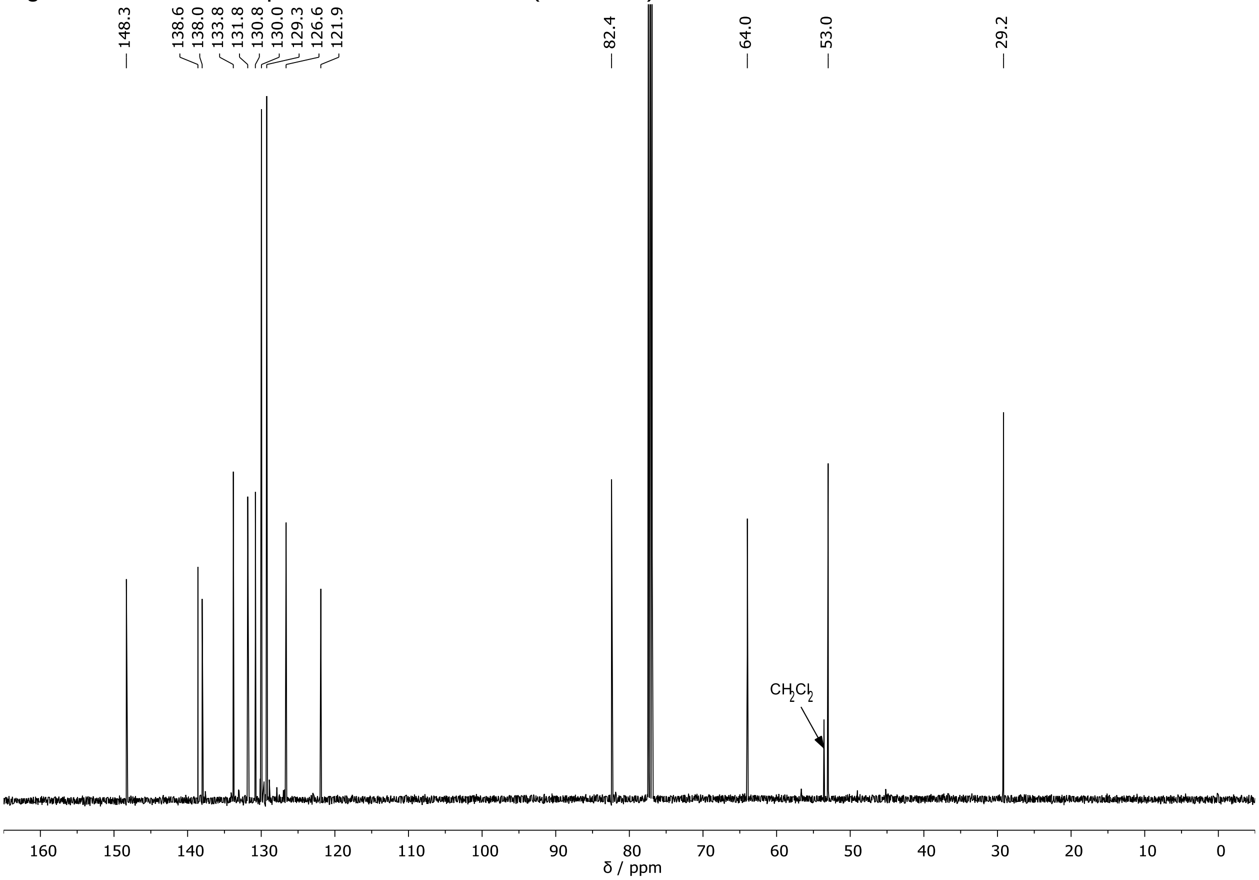

Figure S14. ^{13}C NMR spectrum of **6** in $CDCl_3$ (126 MHz). Contains residual CH_2Cl_2.

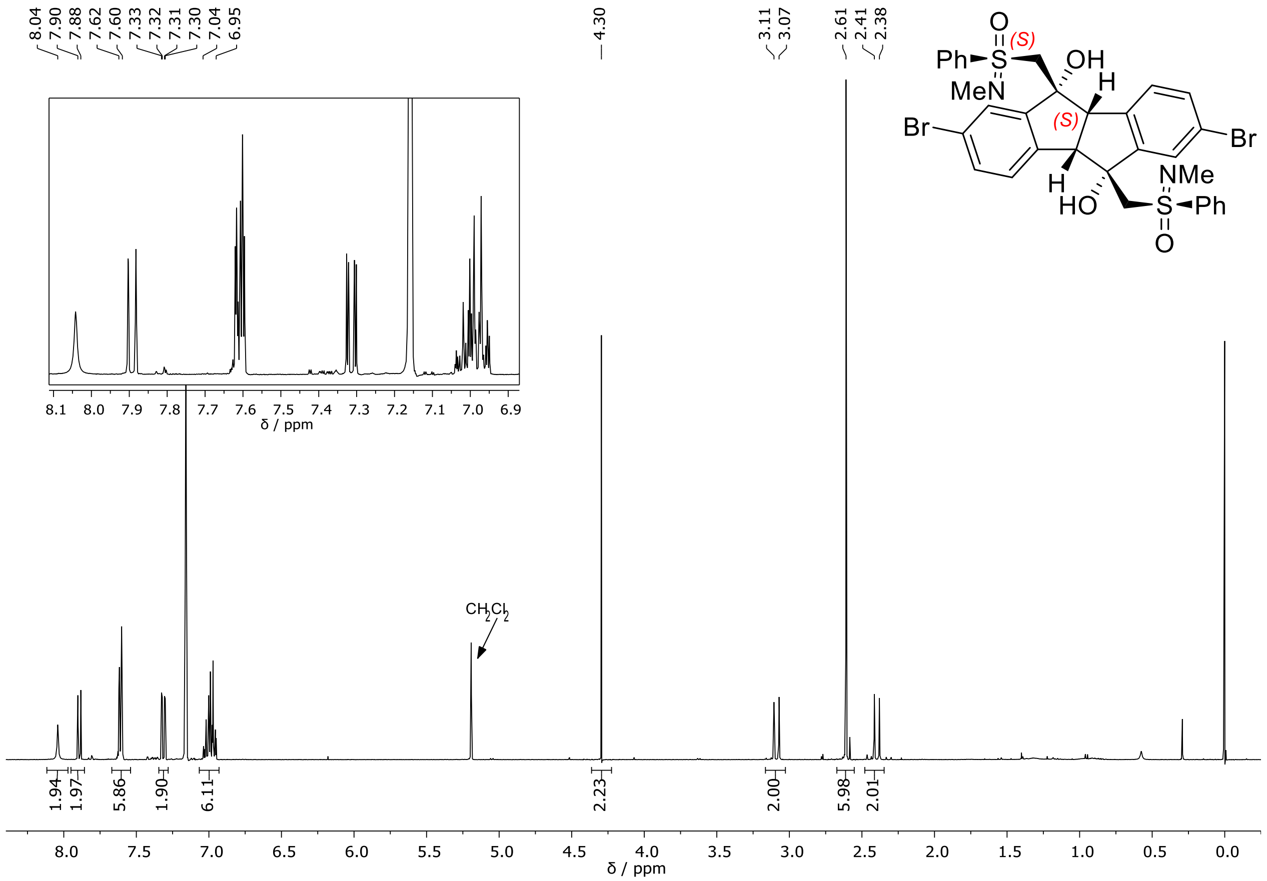

Figure S15. ^{1}H NMR spectrum of **6** in C_6D_6 (500 MHz). Contains residual CH_2Cl_2.

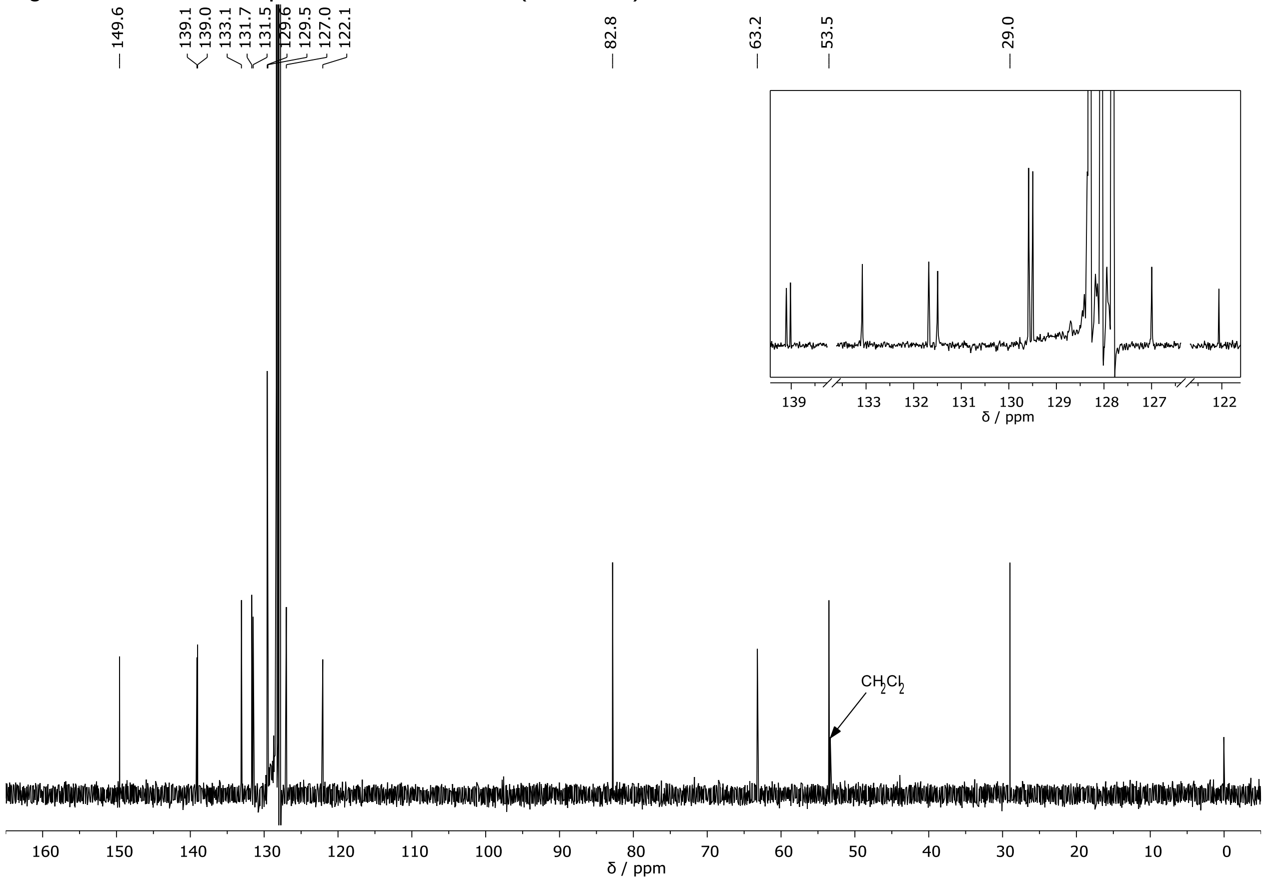

Figure S16. ^{13}C NMR spectrum of **6** in C_6D_6 (126 MHz). Contains residual CH_2Cl_2.

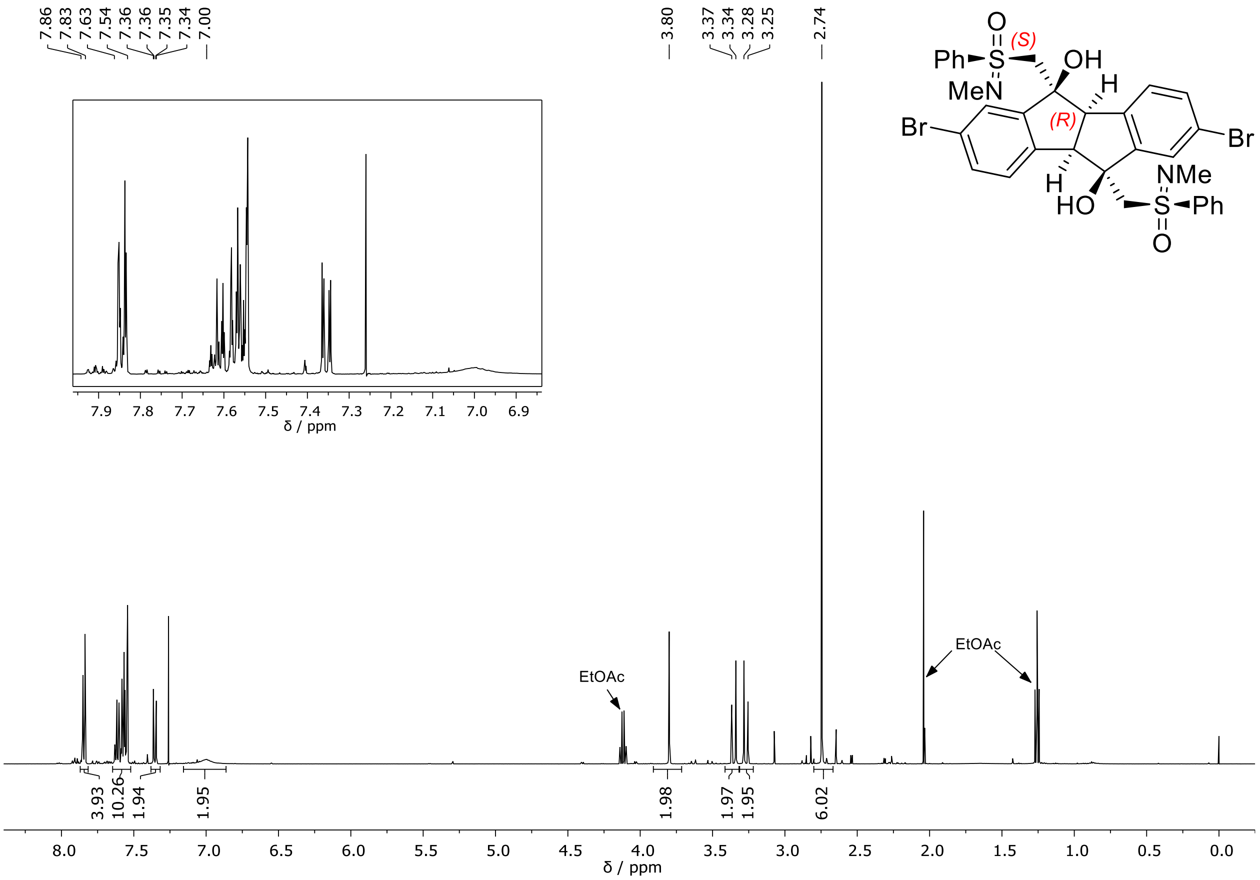

Figure S17. ^{1}H NMR spectrum of **5** in $CDCl_3$ at 300 K (500 MHz). Contains residual EtOAc.

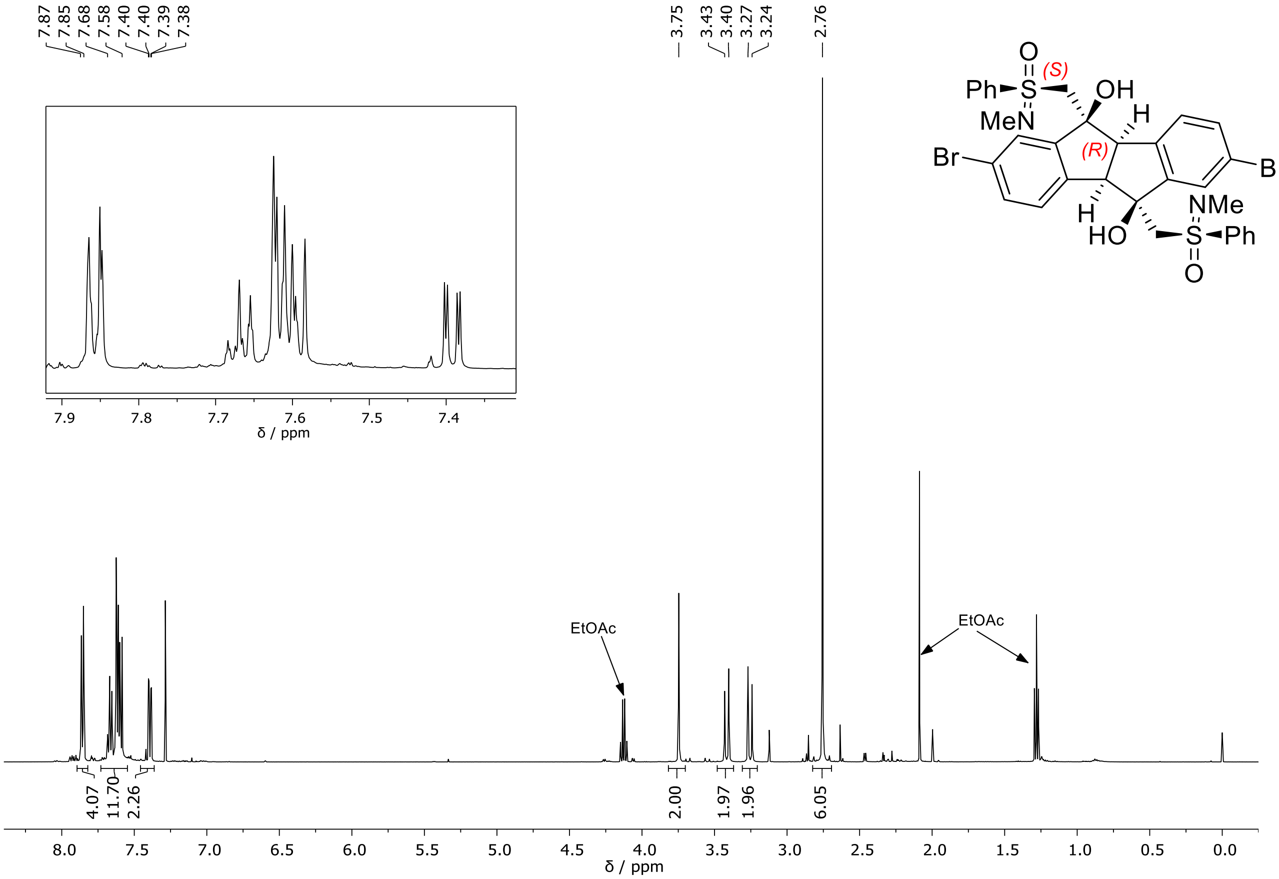

Figure S18. ^{1}H NMR spectrum of **5** in $CDCl_3$ at 253 K (500 MHz). Contains residual EtOAc.

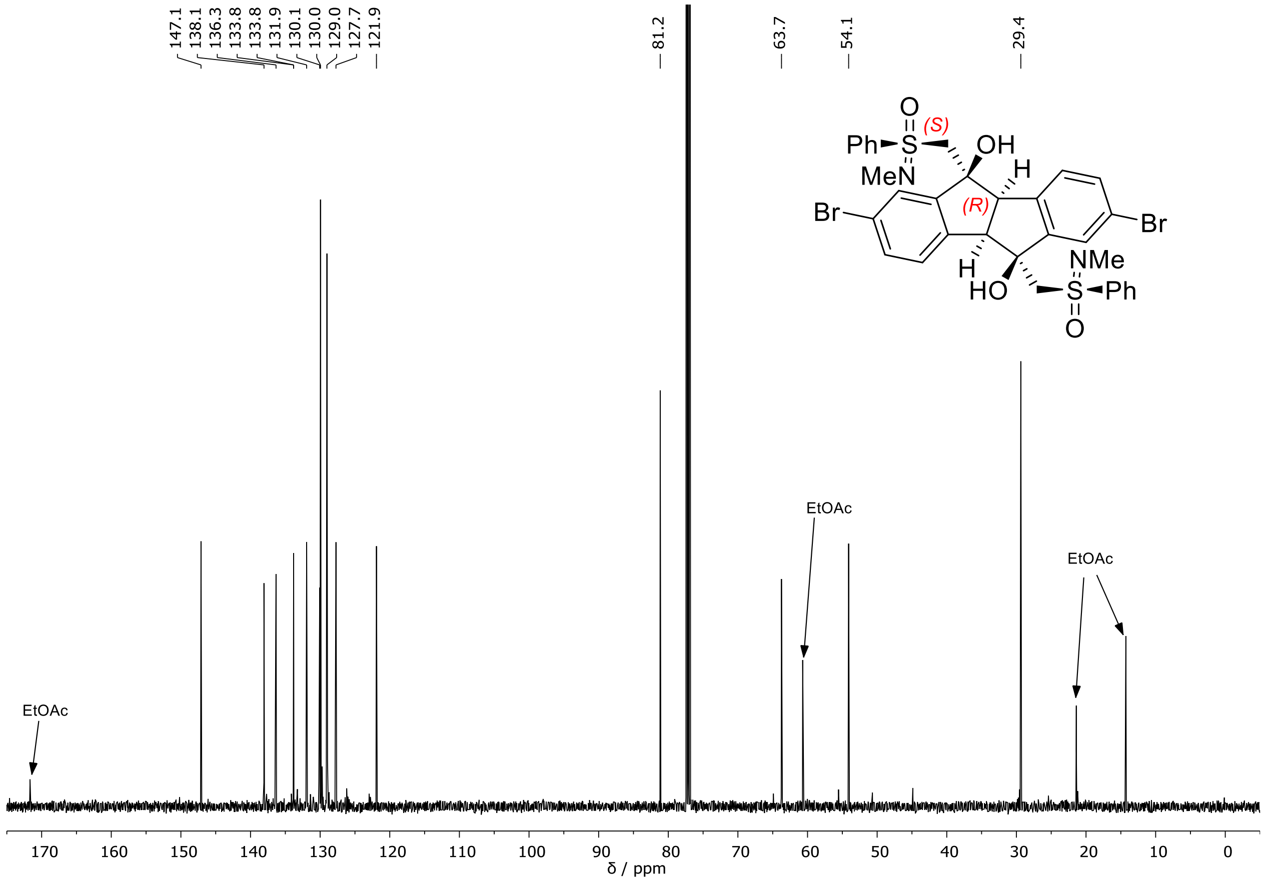

Figure S19. ^{13}C NMR spectrum of **5** in $CDCl_3$ at 253 K (126 MHz).Contains residual EtOAc.

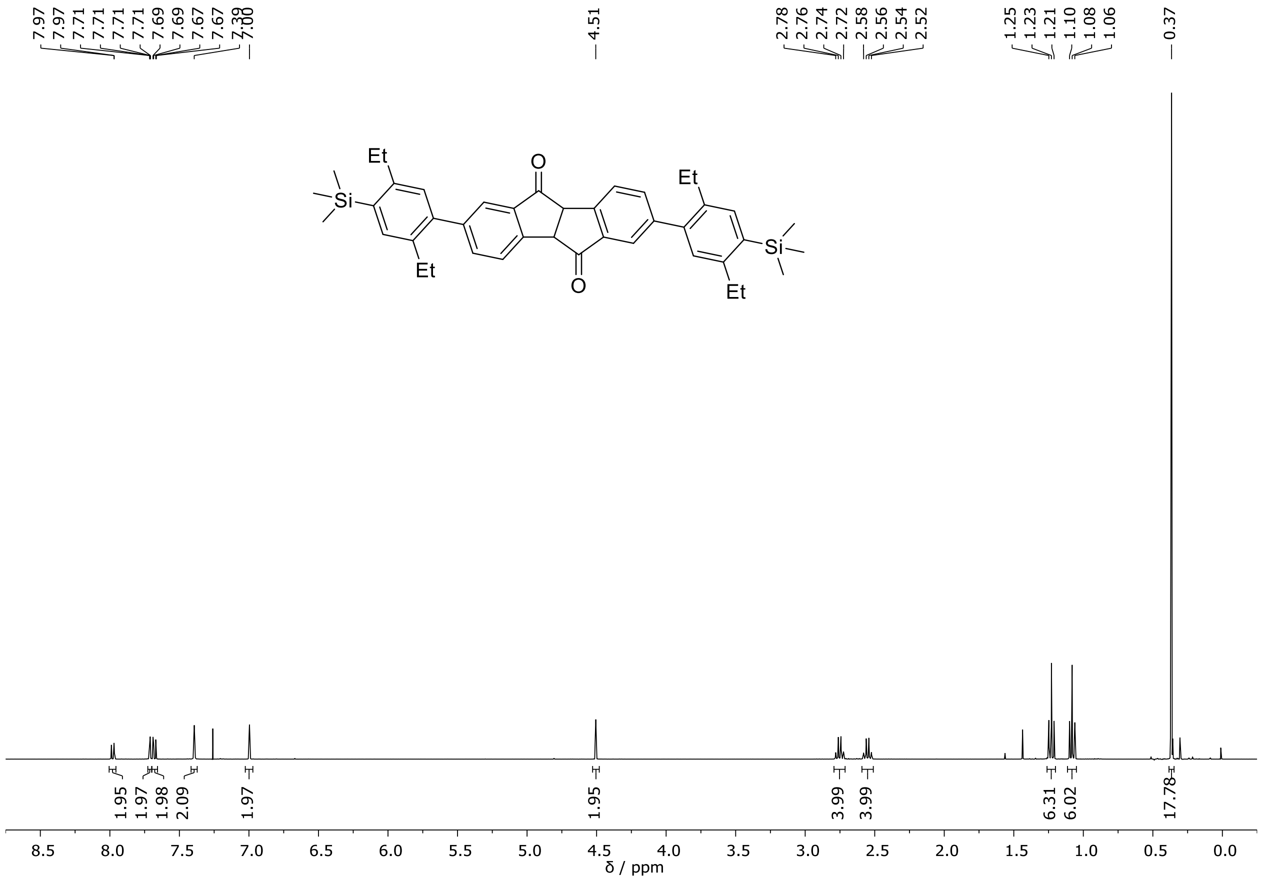

Figure S20. ^{1}H NMR spectrum of ***rac*-9** in $CDCl_3$ (400 MHz).

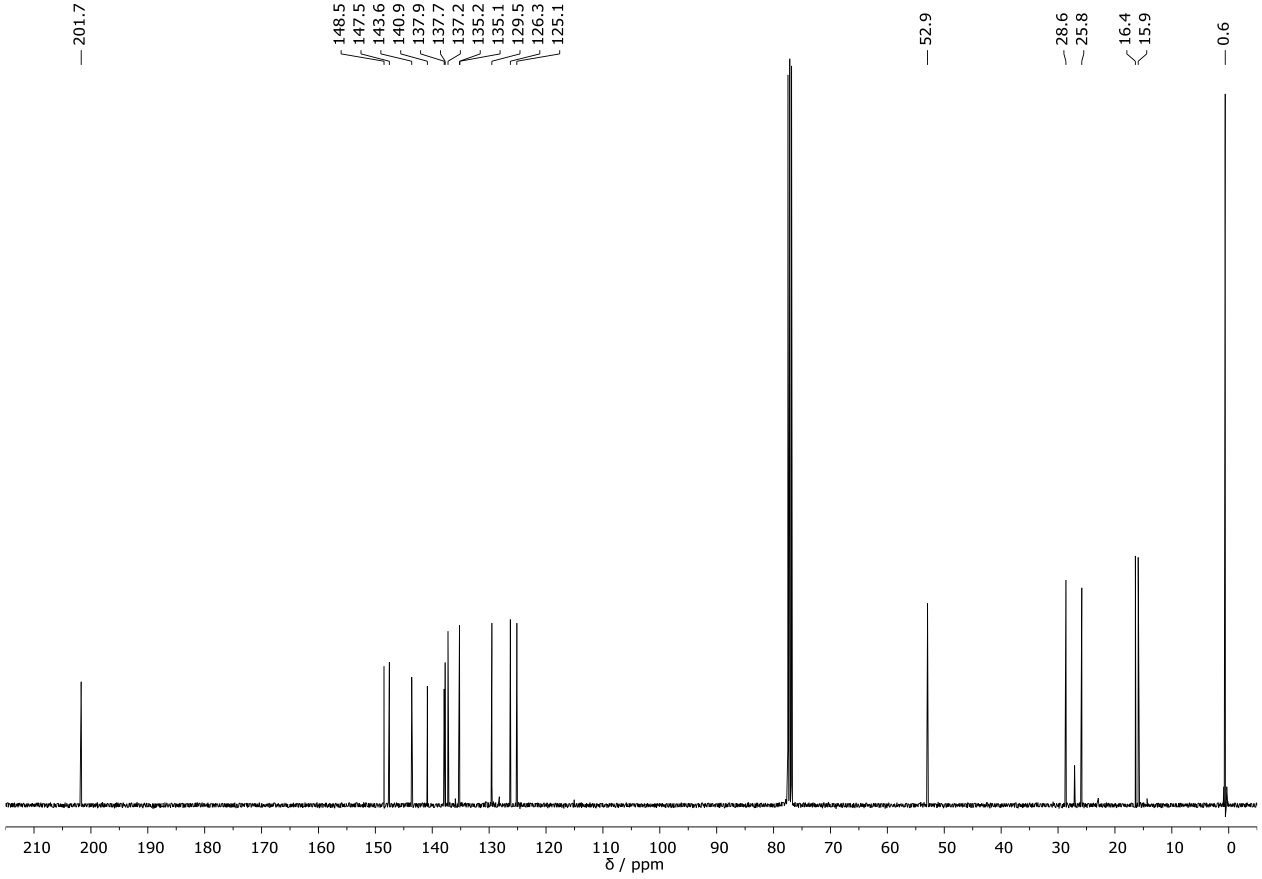

Figure S21. ^{13}C NMR spectrum of ***rac*-9** in $CDCl_3$ (101 MHz).

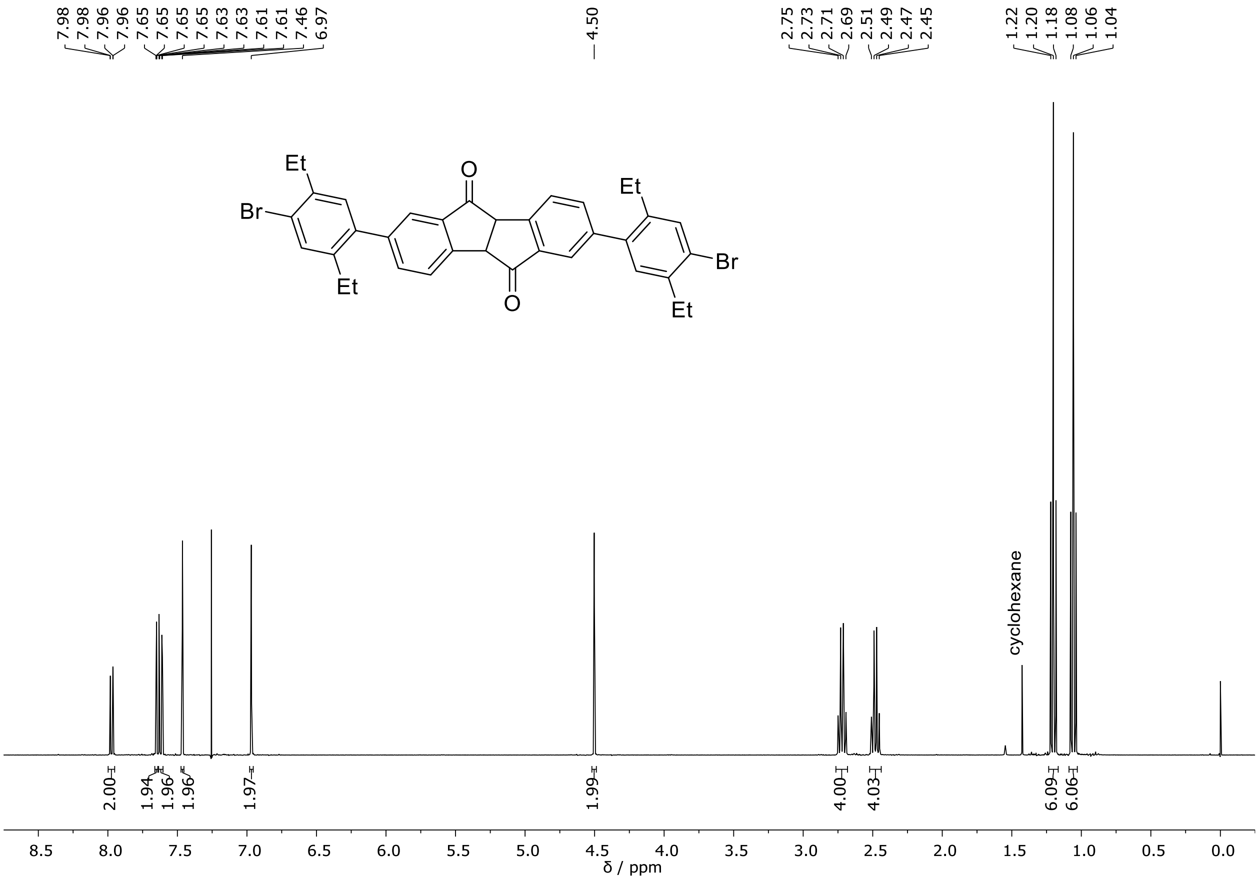

Figure S22. ^{1}H NMR spectrum of ***rac*-10** in $CDCl_3$ (400 MHz).

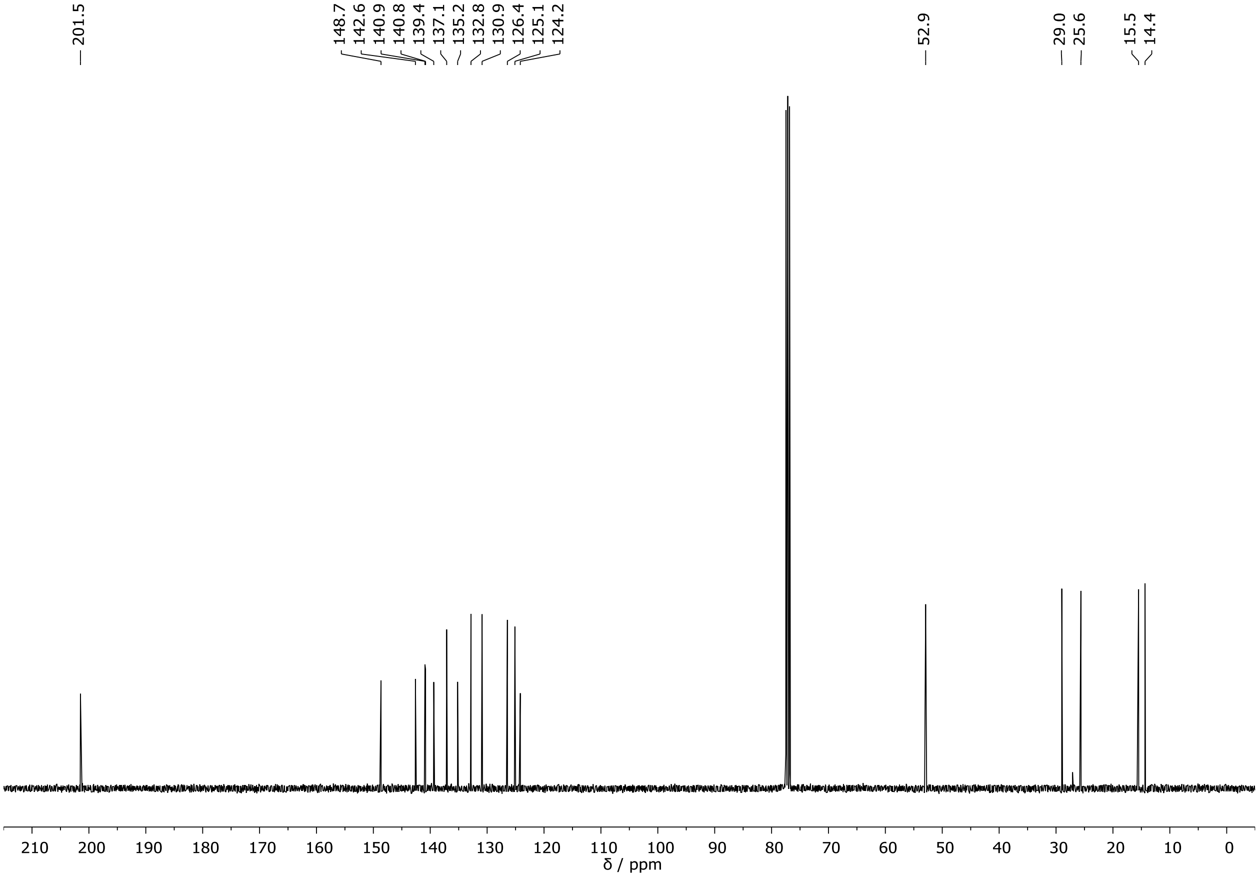

Figure S23. ^{13}C NMR spectrum of ***rac*-10** in $CDCl_3$ (101 MHz).

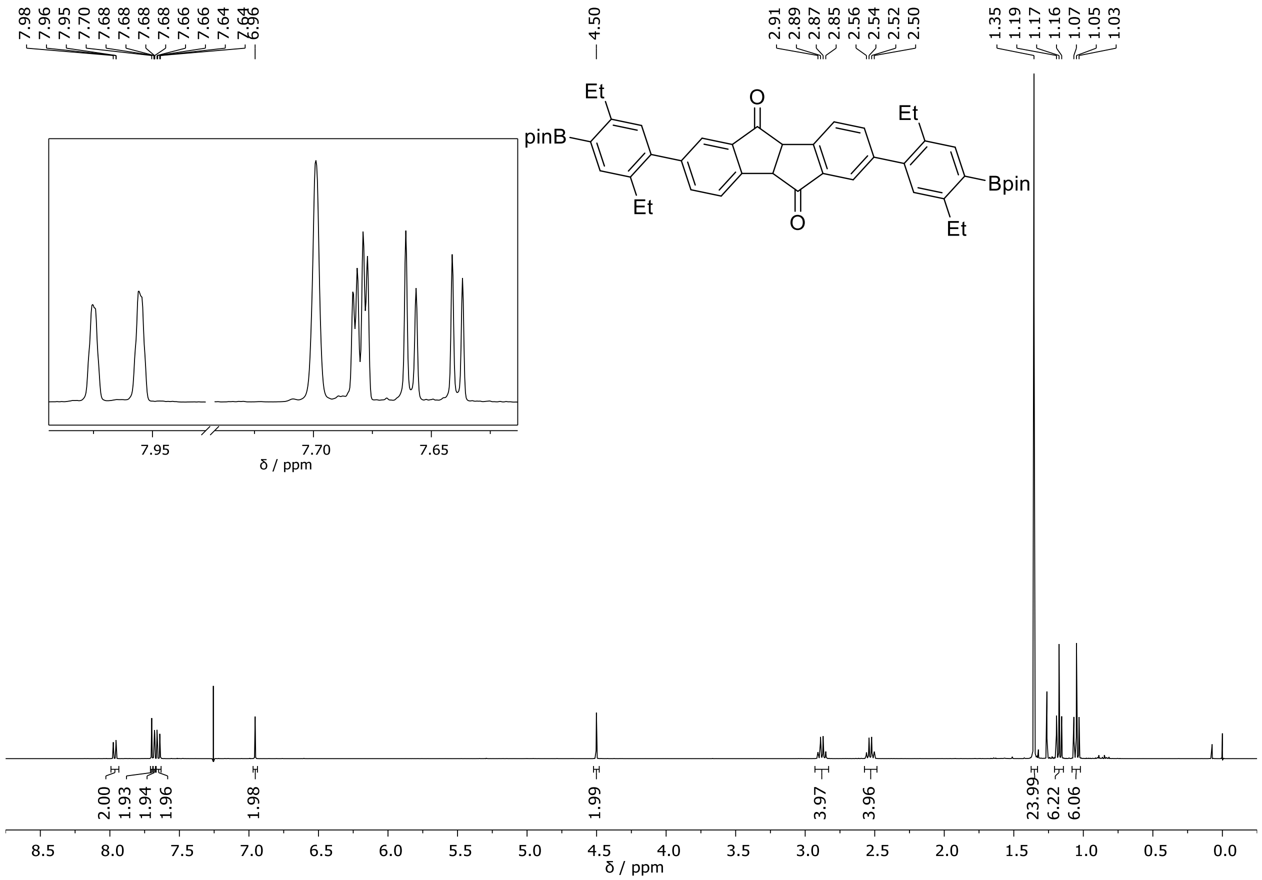

Figure S24. ^{1}H NMR spectrum of ***rac*-11** in $CDCl_3$ (400 MHz).

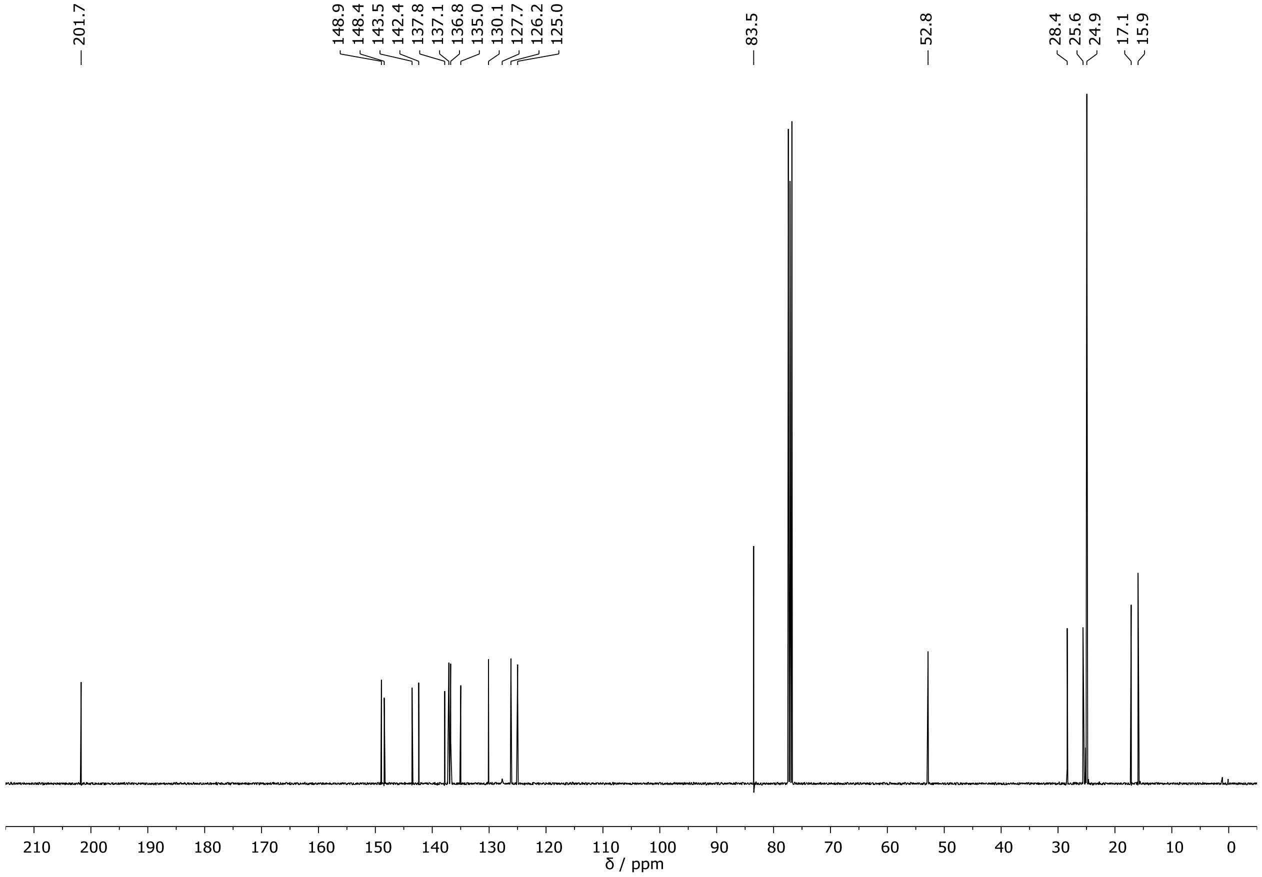

Figure S25. ^{13}C NMR spectrum of ***rac*-11** in $CDCl_3$ (101 MHz).

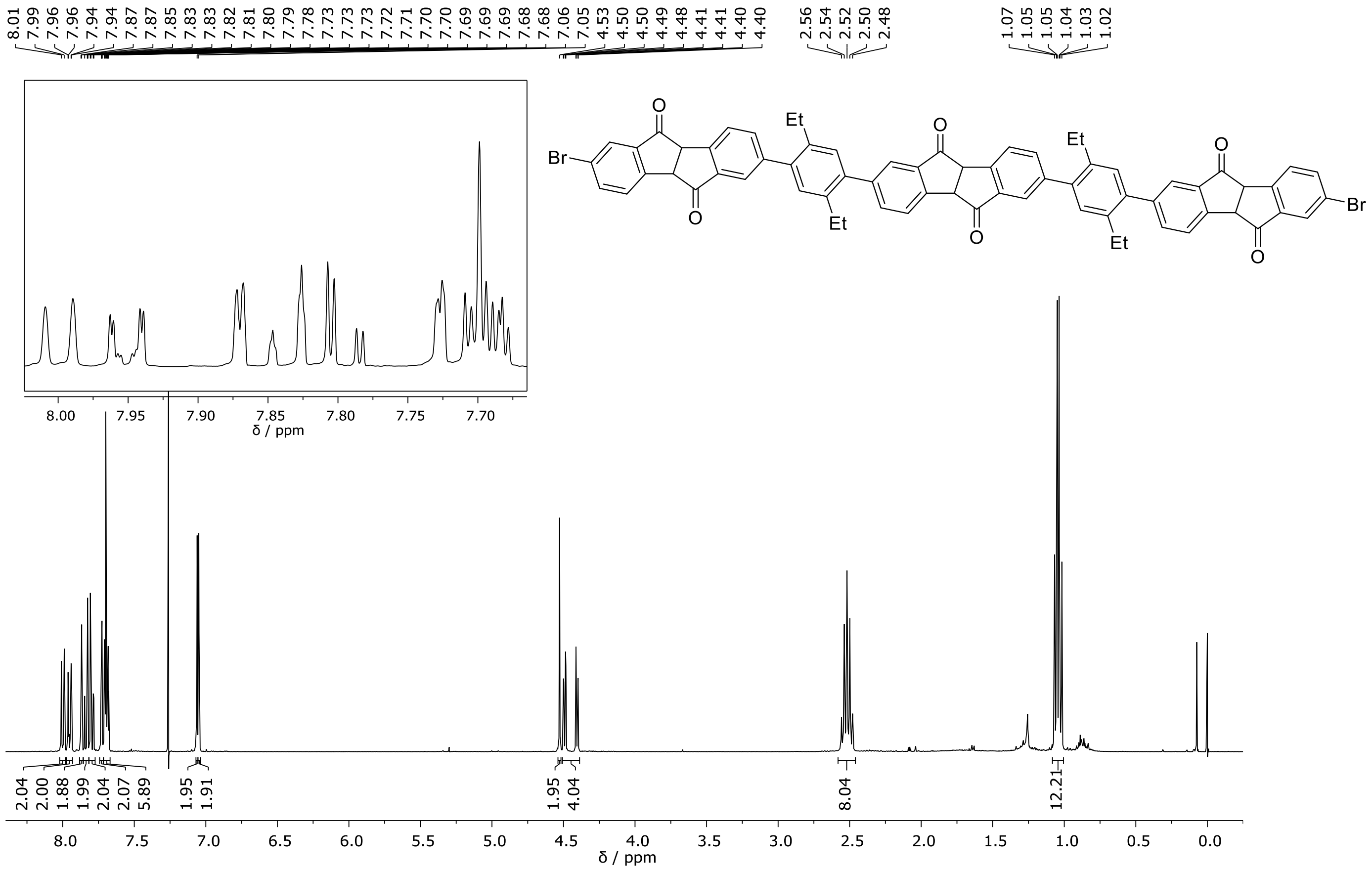

Figure S26. ^{1}H NMR spectrum of **18** in $CDCl_3$ (400 MHz).

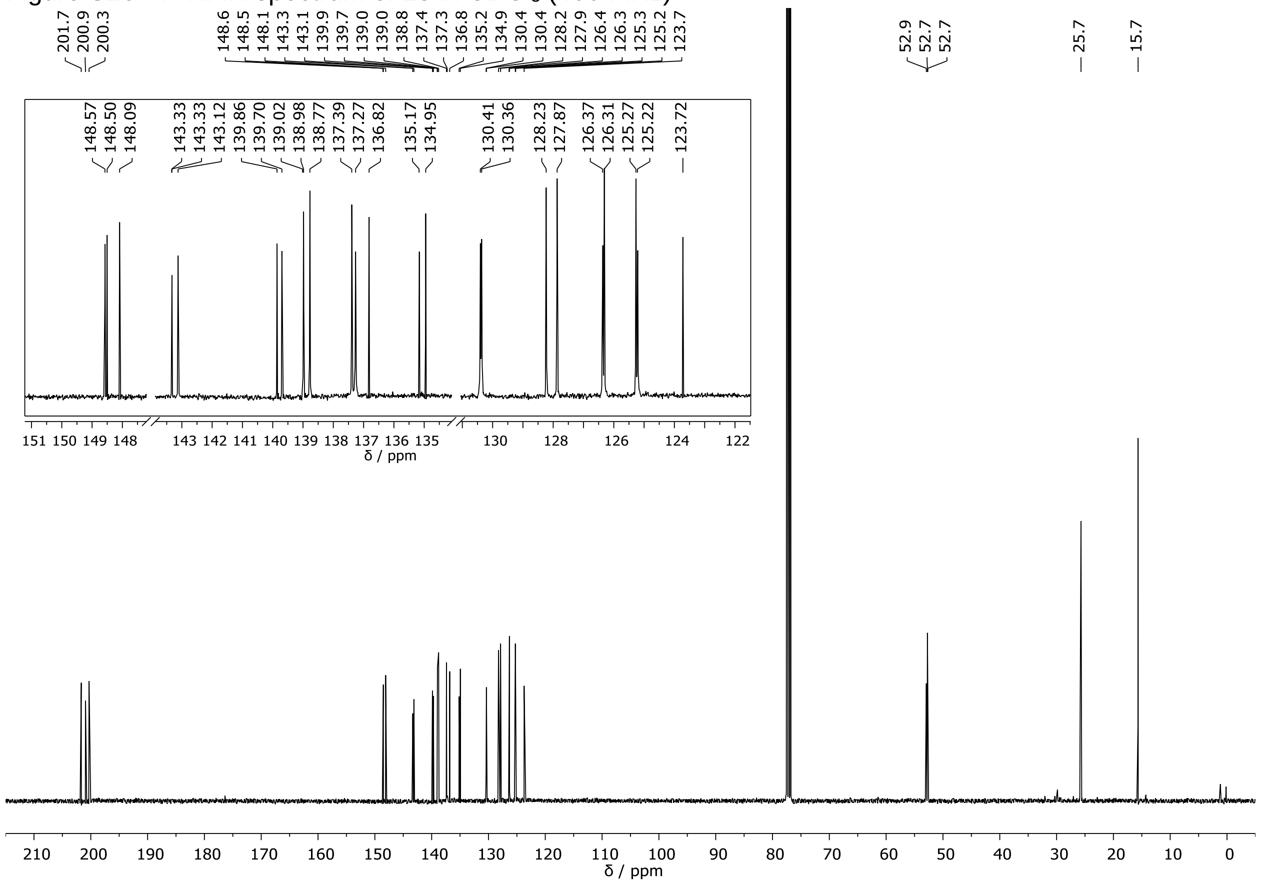

Figure S27. ^{13}C NMR spectrum of **18** in $CDCl_3$ (101 MHz).

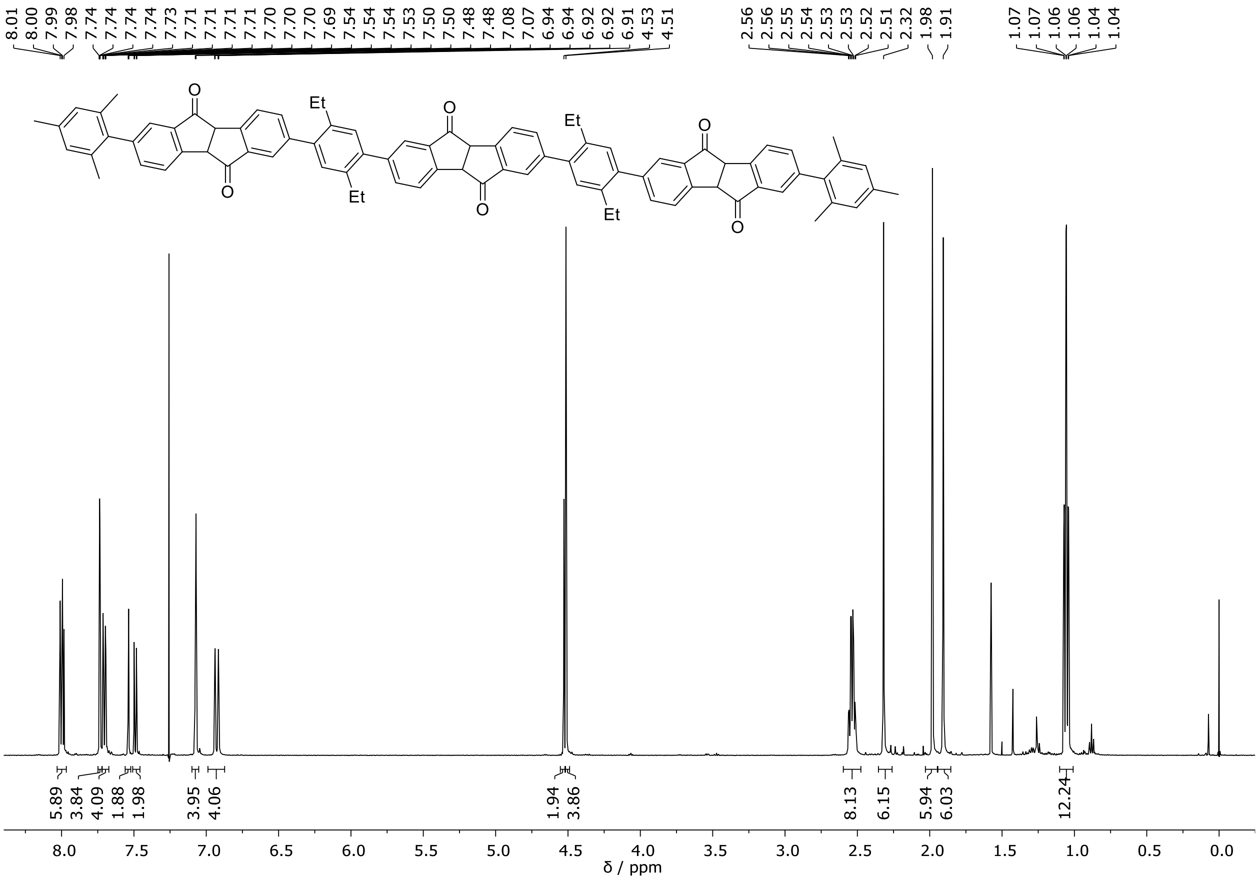

Figure 28. ^{1}H NMR spectrum of **19** in $CDCl_3$ (500 MHz).

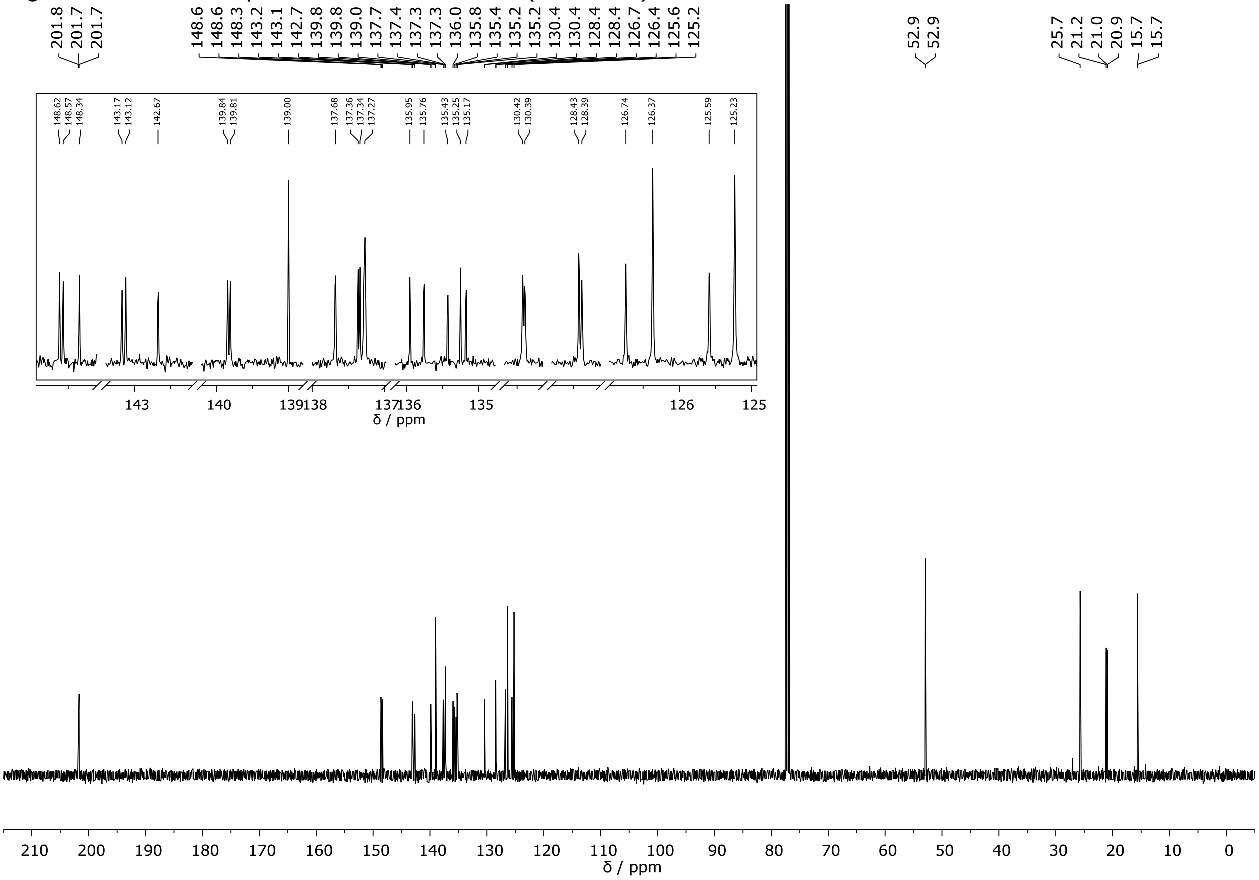

Figure 29. ^{13}C NMR spectrum of **19** in $CDCl_3$ (126 MHz).

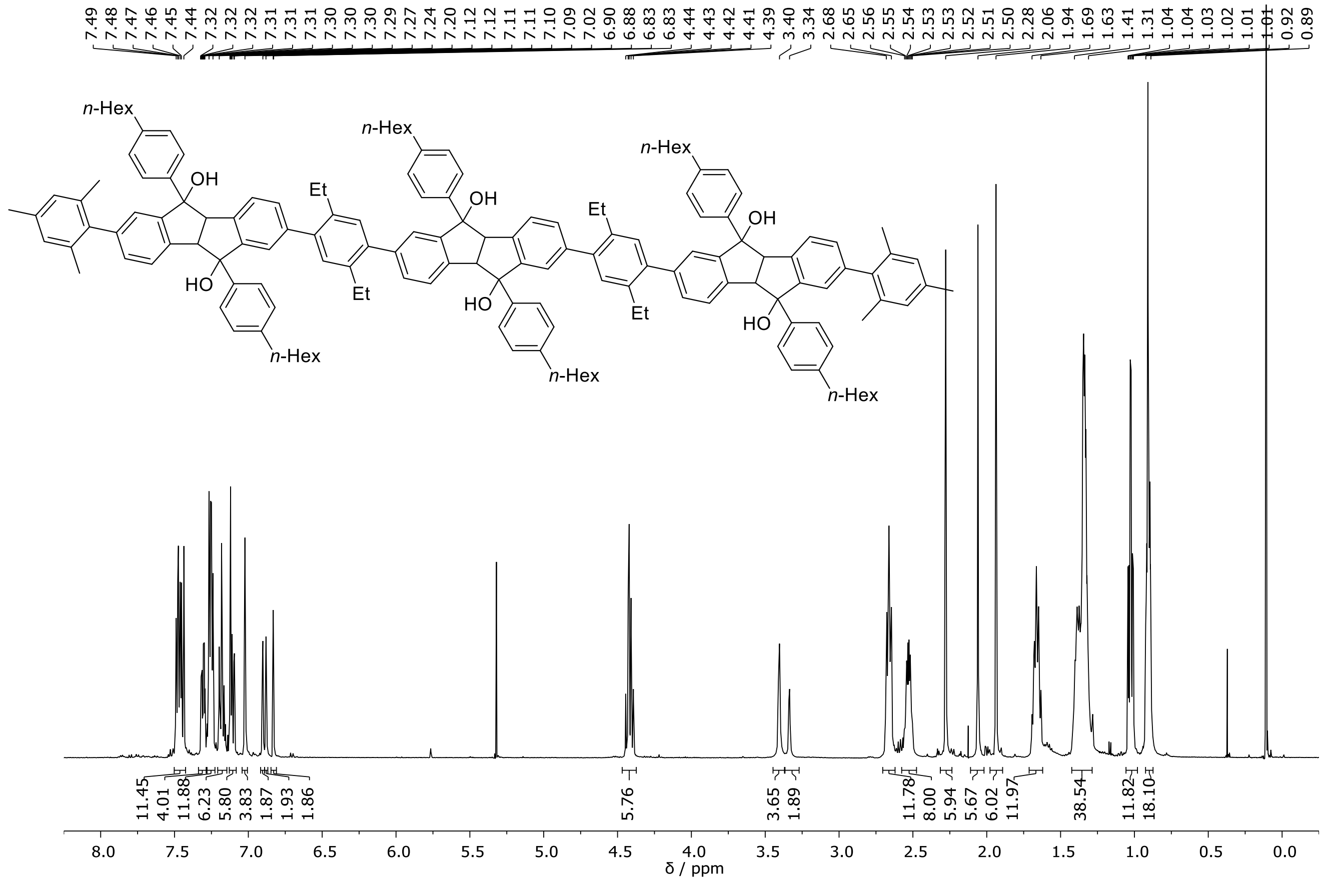

Figure S30. ^{1}H NMR spectrum of **S4** in CD_2Cl_2 (500 MHz).

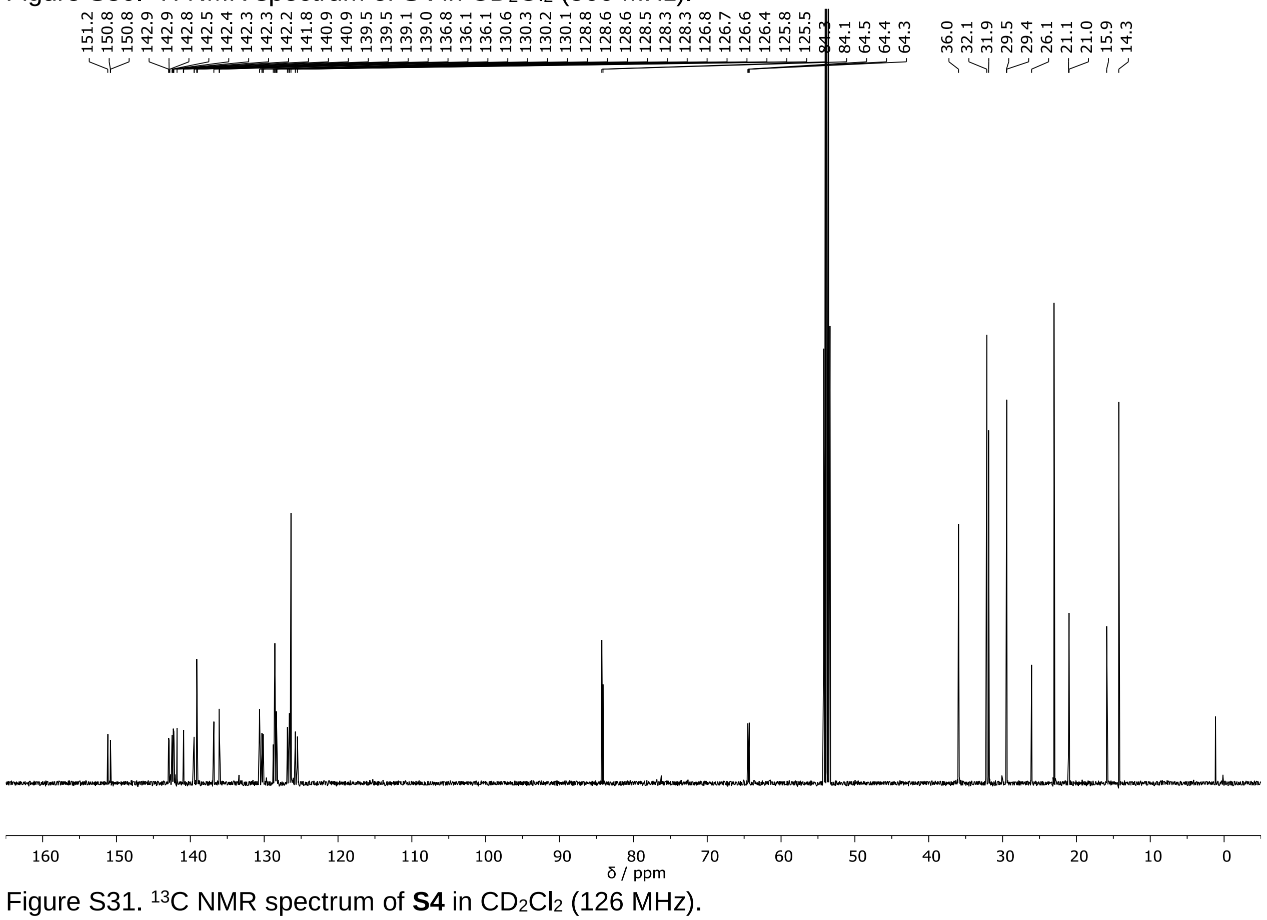

Figure S31. ^{13}C NMR spectrum of **S4** in CD_2Cl_2 (126 MHz).

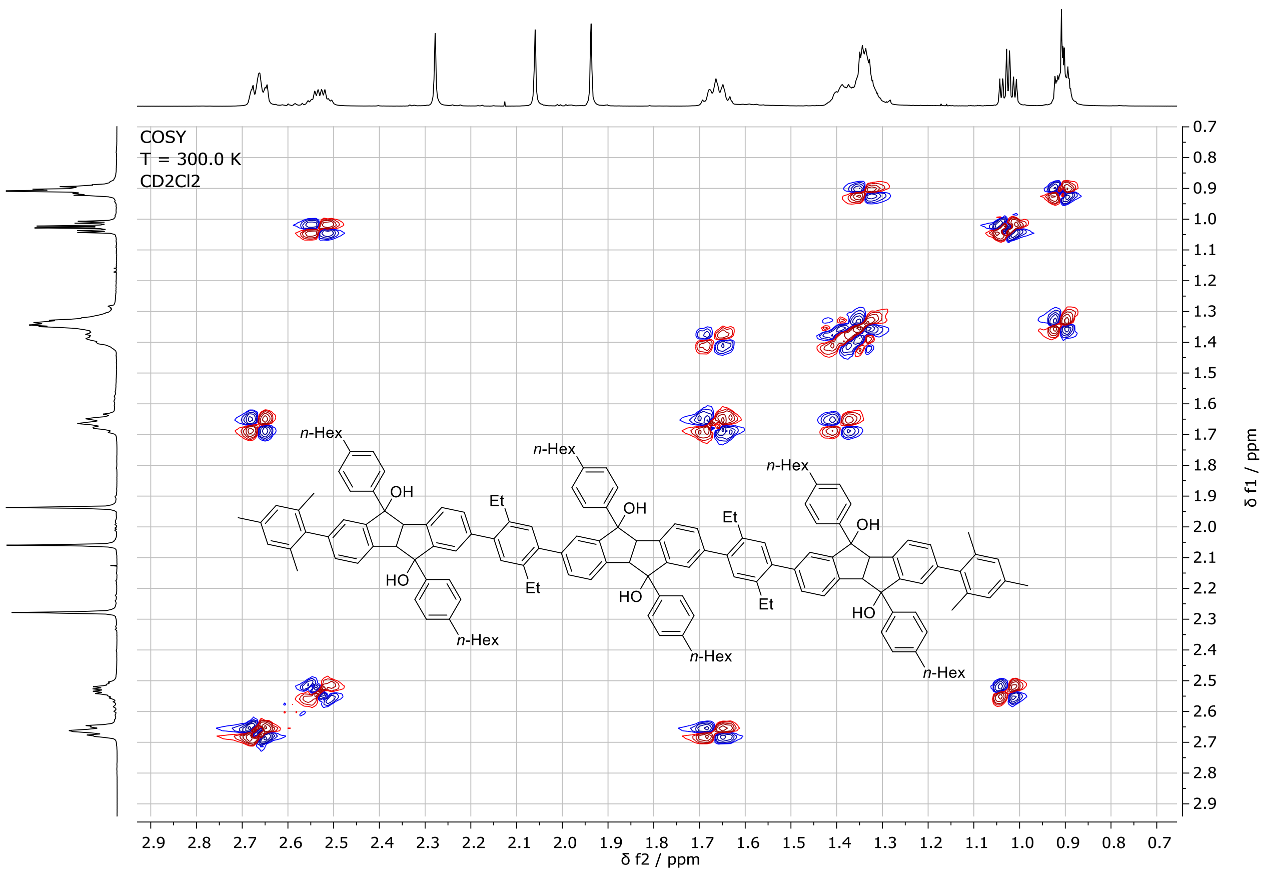

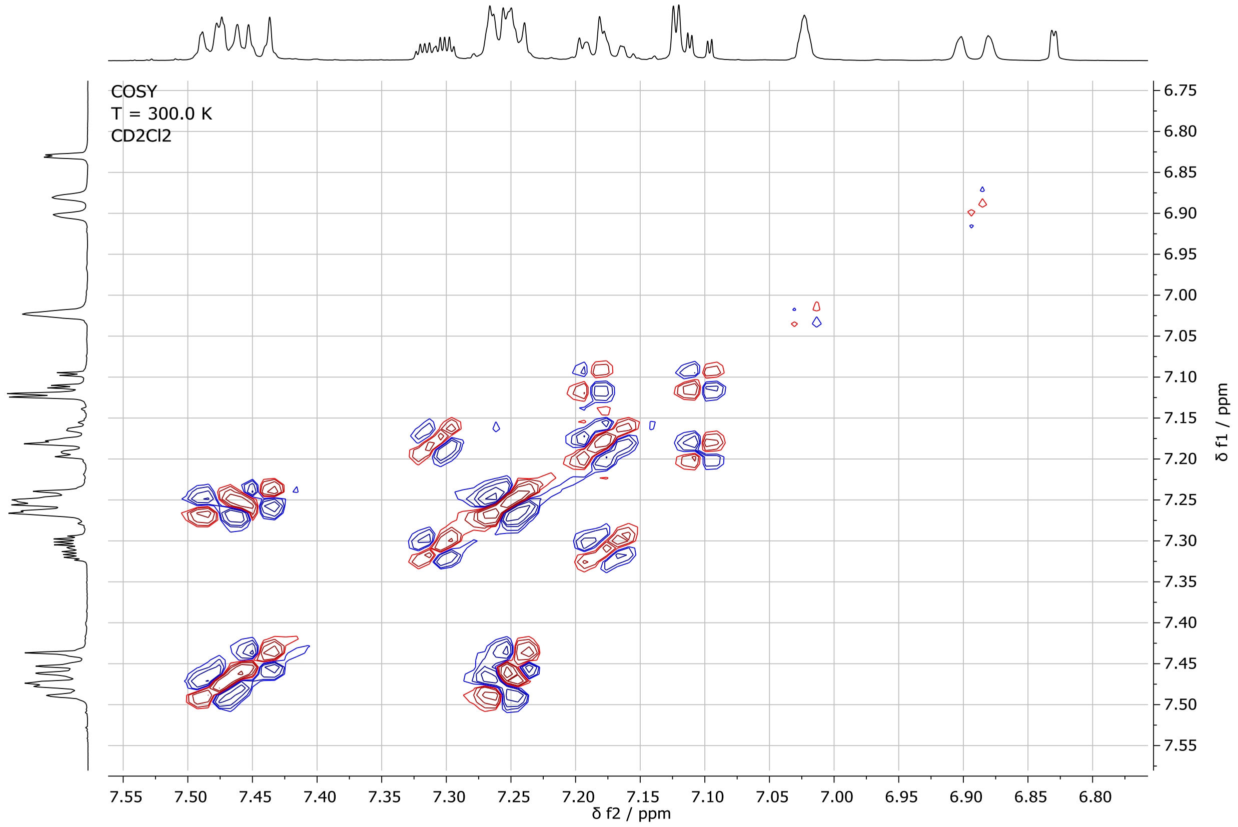

Figure S32. H,H-COSY NMR spectra of **S4** in CD_2Cl_2 (500 MHz).

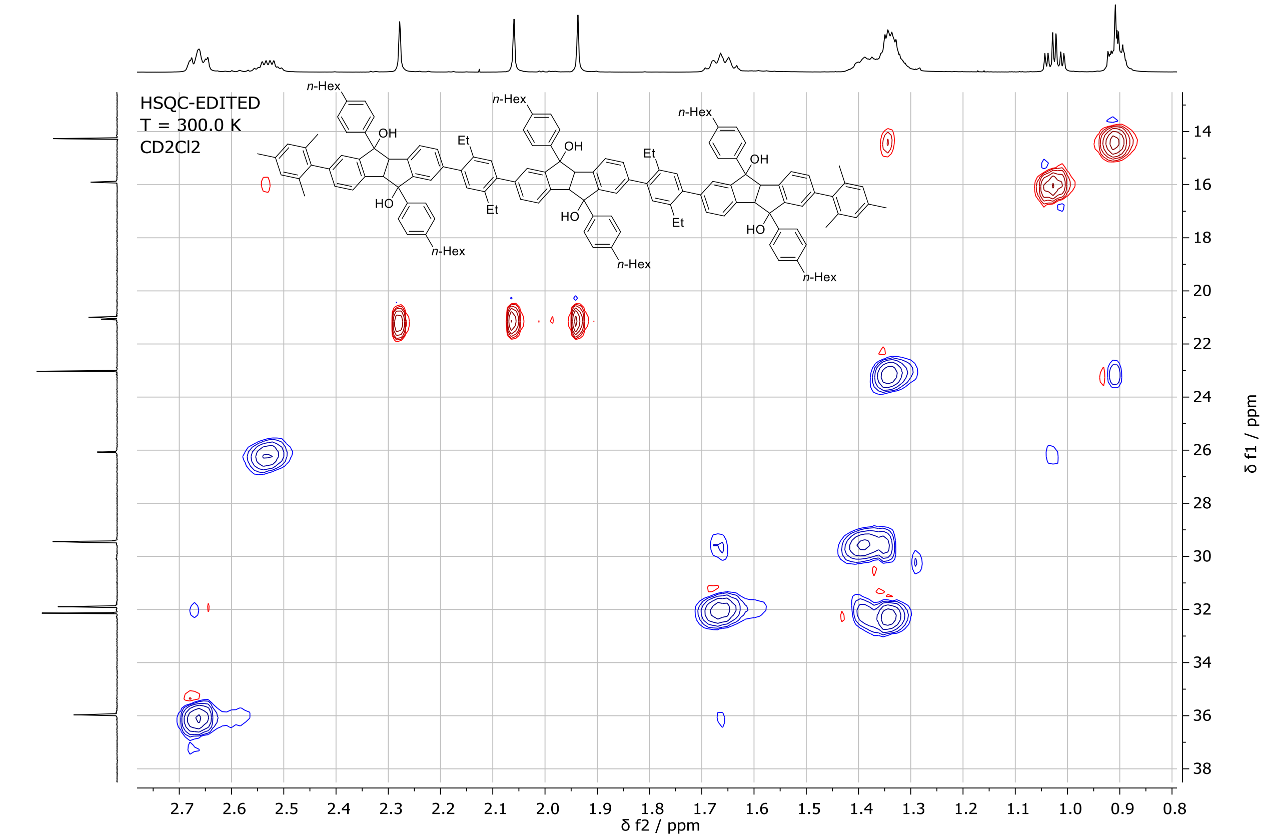

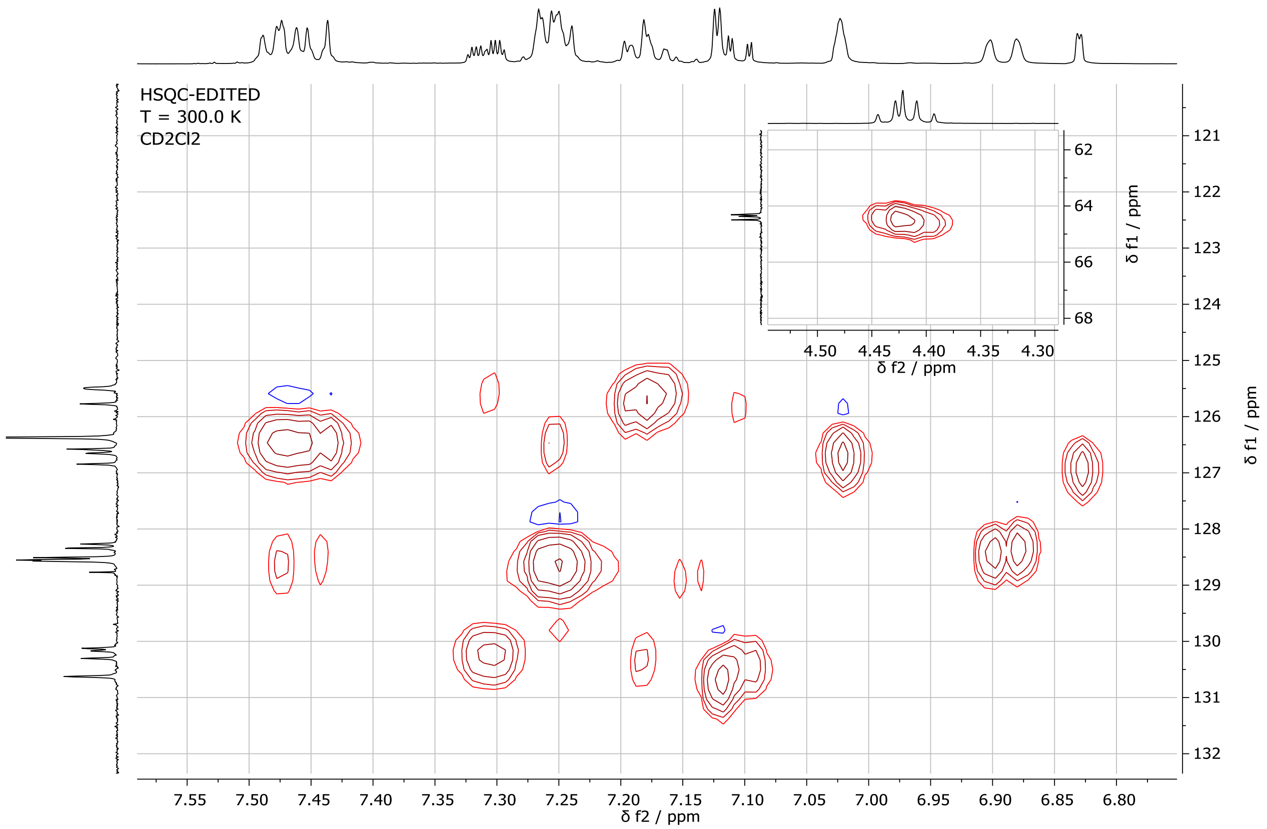

Figure S33. Edited HSQC NMR spectra of **S4** in CD_2Cl_2 (500/126 MHz). Blue: CH_2, red: CH or CH_3.

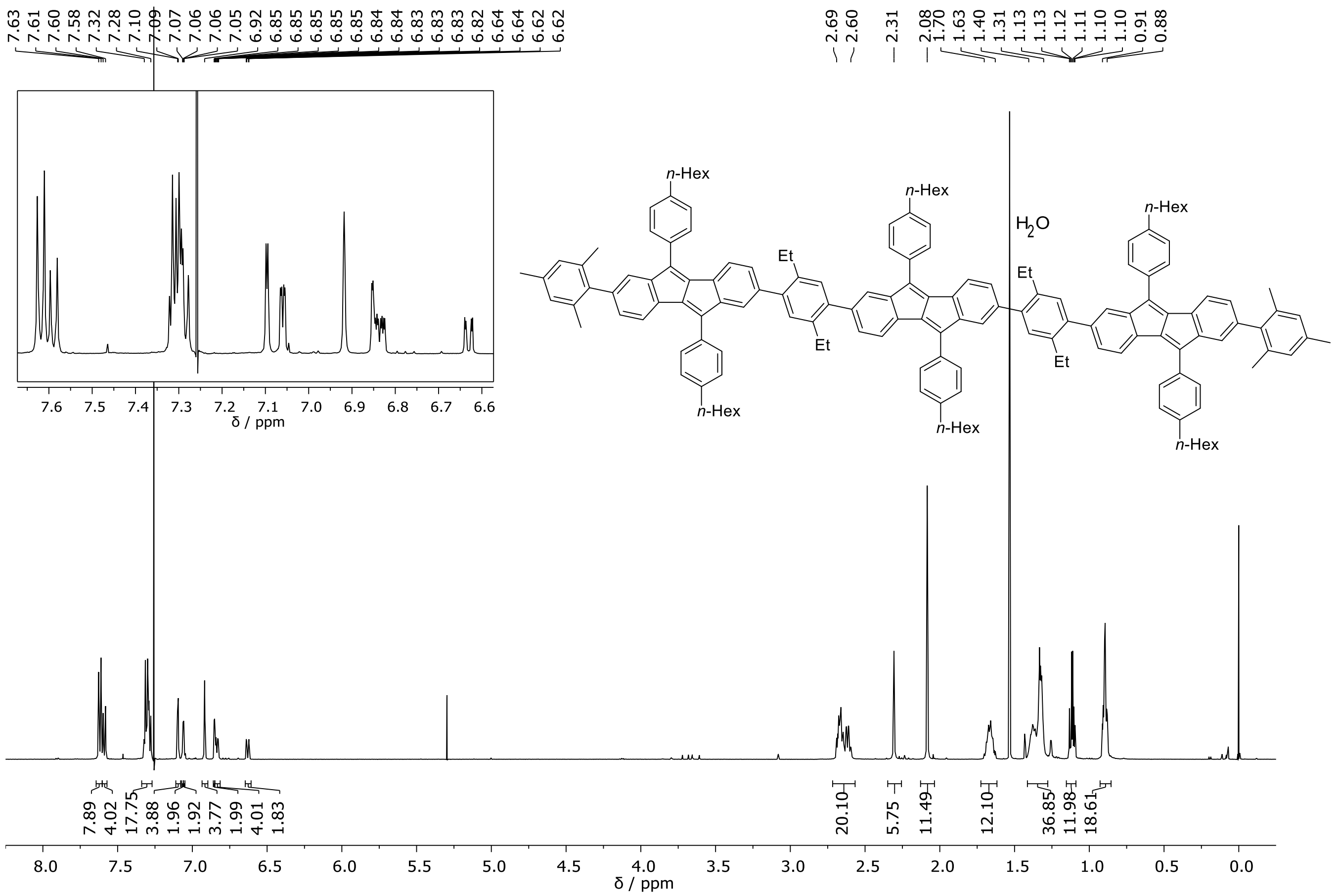

Figure S34. ^{1}H NMR spectrum of **7** in $CDCl_3$ (500 MHz).

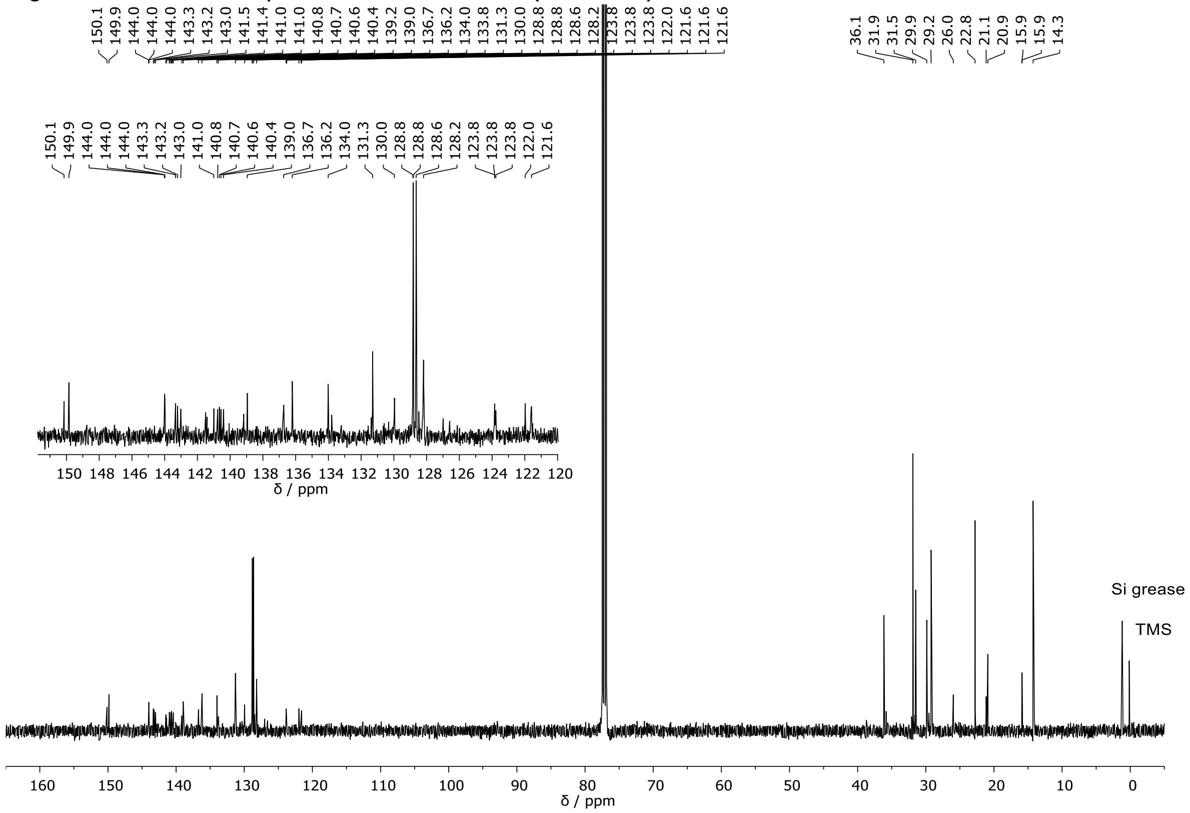

Figure S35. ^{13}C NMR spectrum of **7** in $CDCl_3$ (126 MHz).

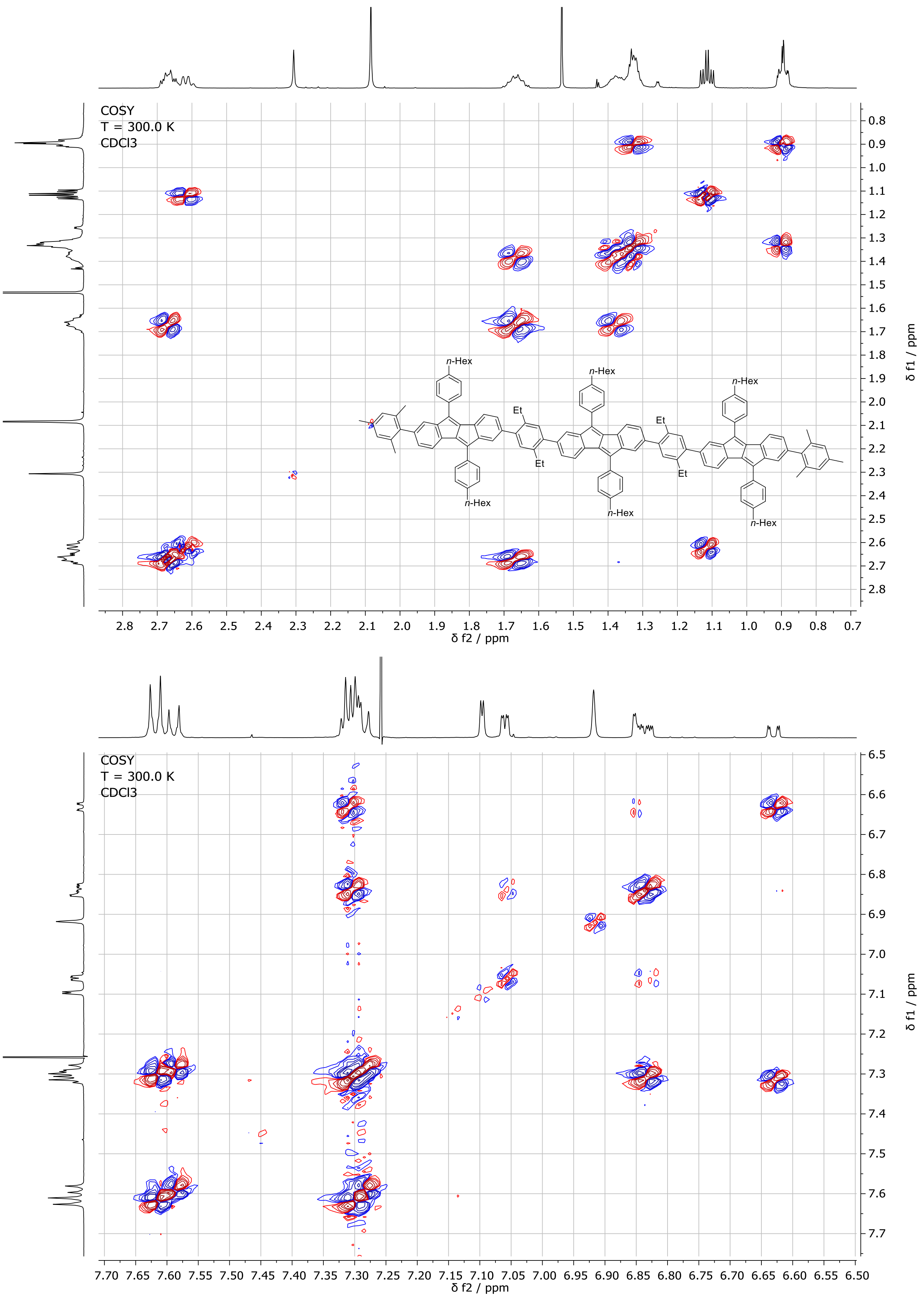

Figure S36. H,H-COSY NMR spectra of **7** in $CDCl_3$ (500 MHz).

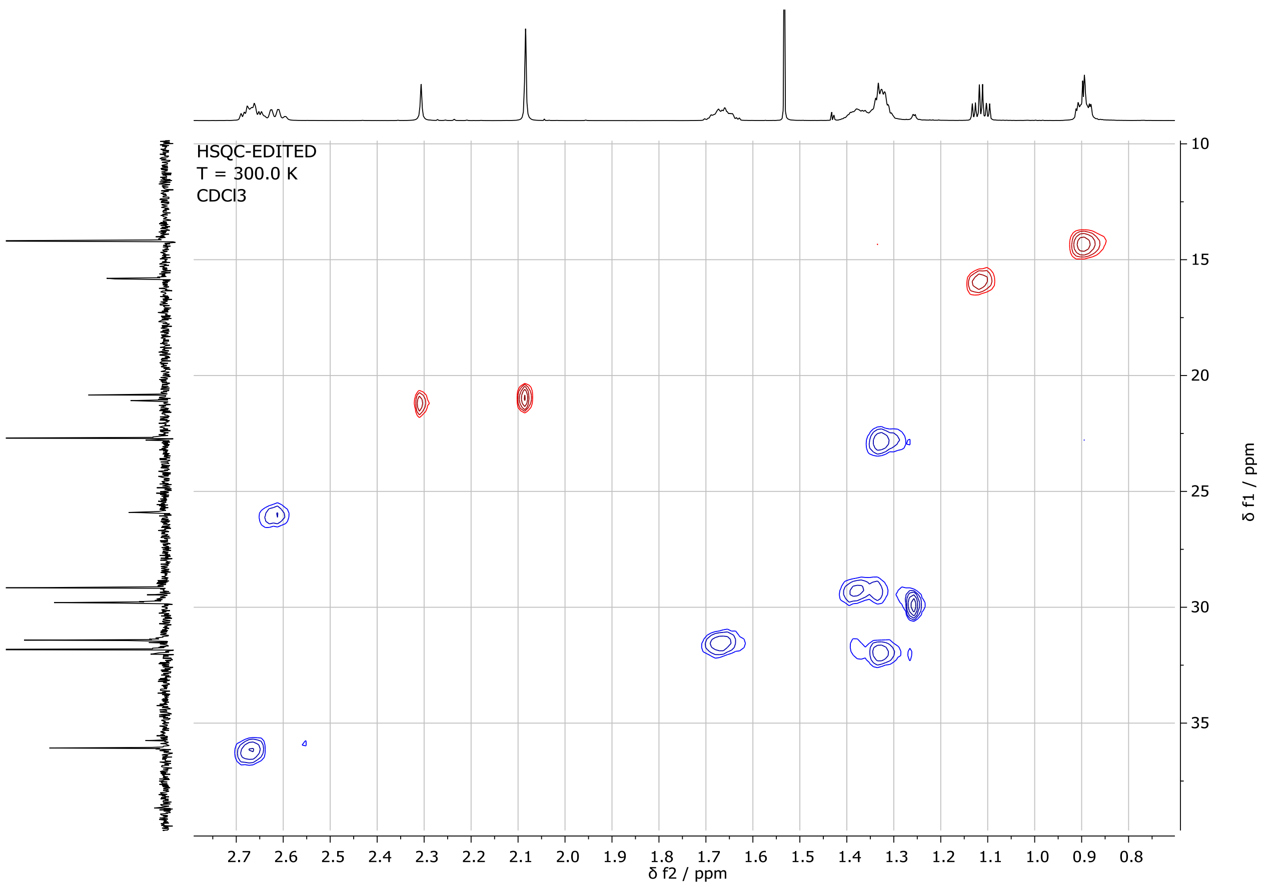

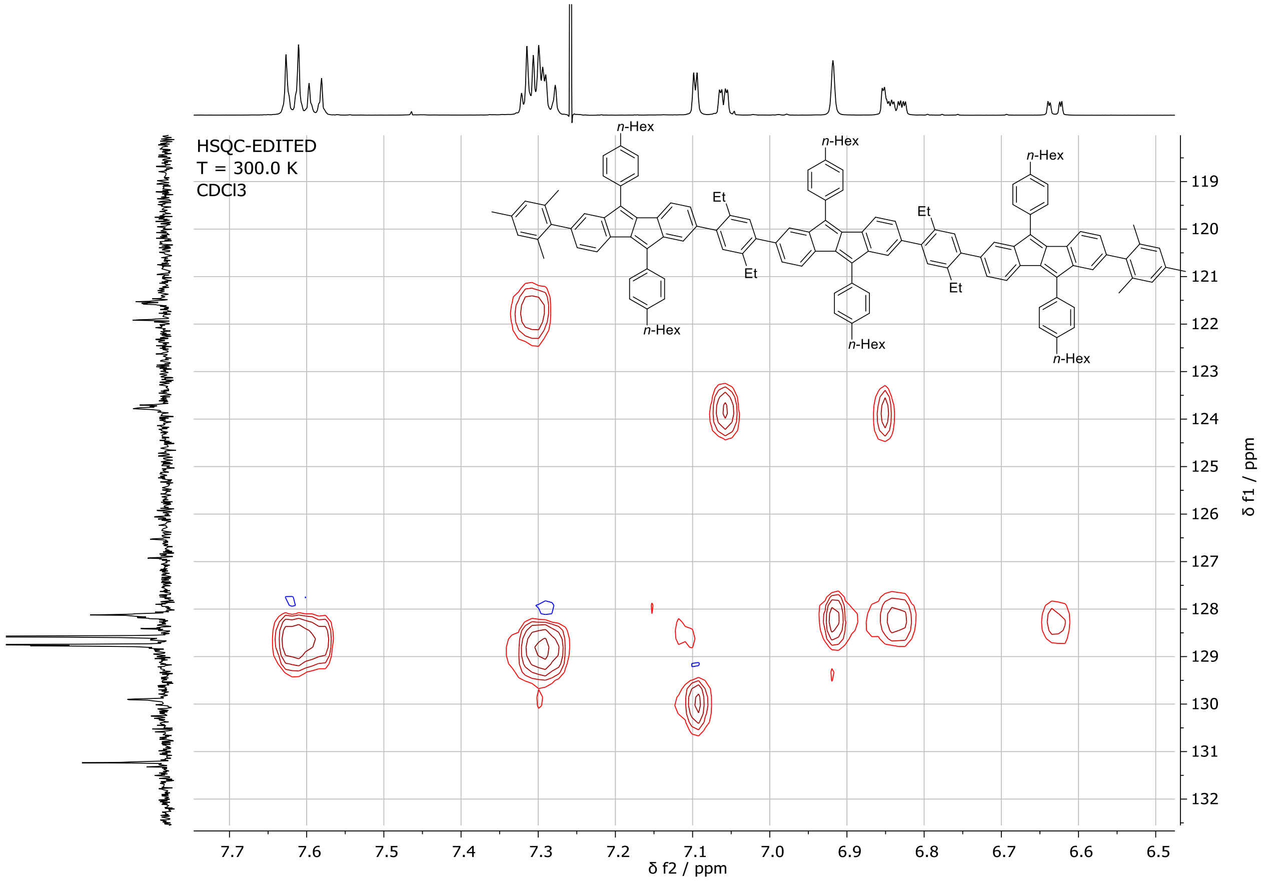

Figure S37. Edited HSQC NMR spectra of **7** in $CDCl_3$ (500 MHz). Blue: CH_2, red: CH or CH_3.

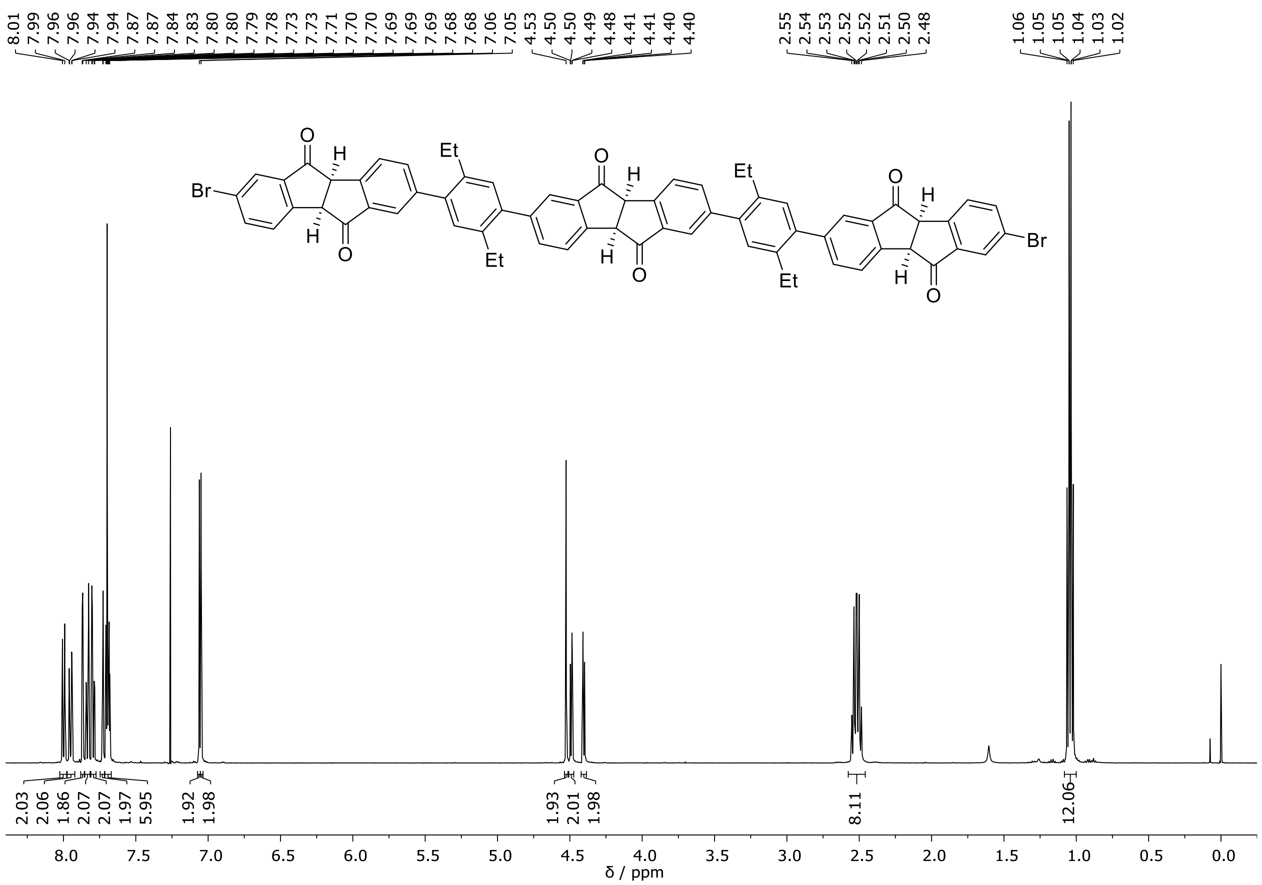

Figure S38. ^{1}H NMR spectrum of **(*R*,*R*)³-12** in $CDCl_3$ (500 MHz).

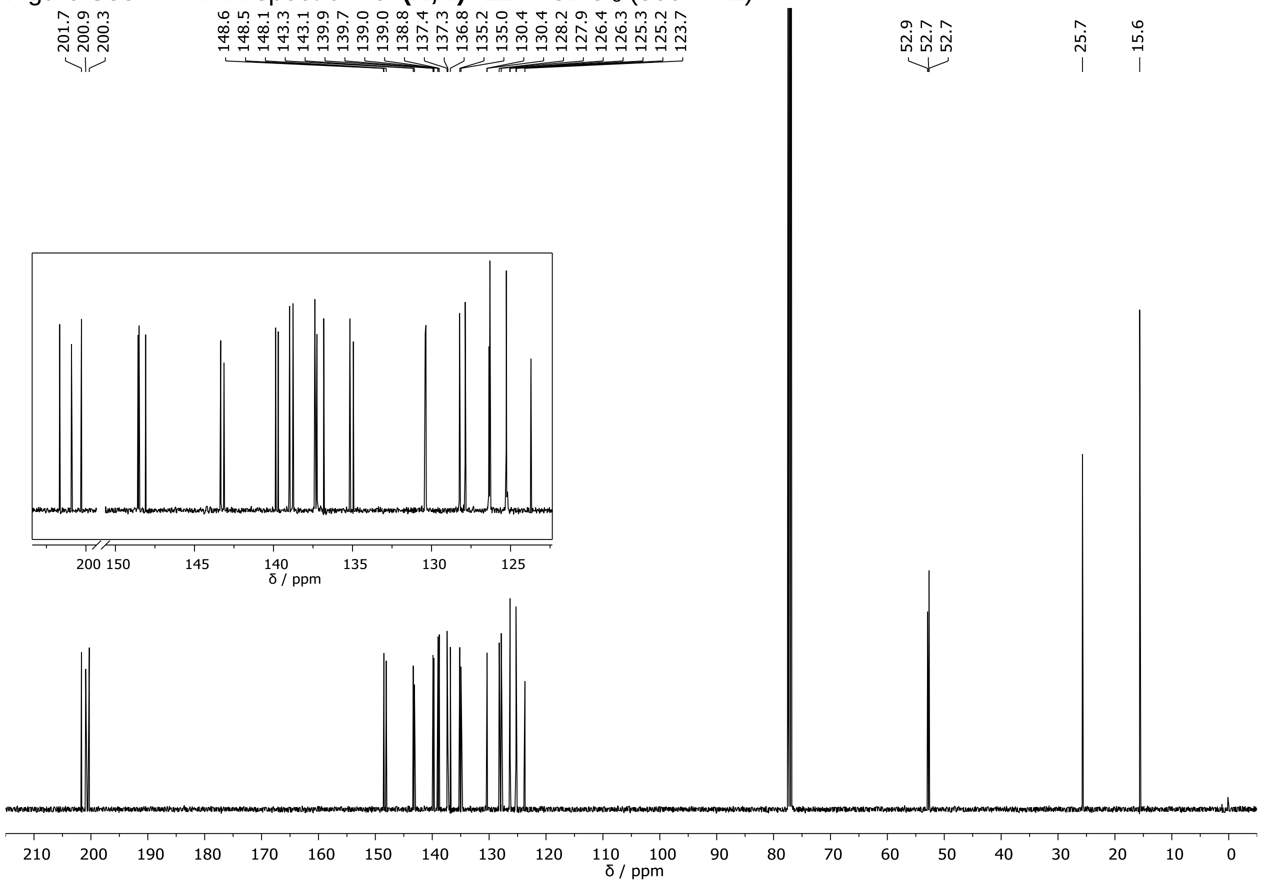

Figure S39. ^{13}C NMR spectrum of **(*R*,*R*)³-12** in $CDCl_3$ (126 MHz).

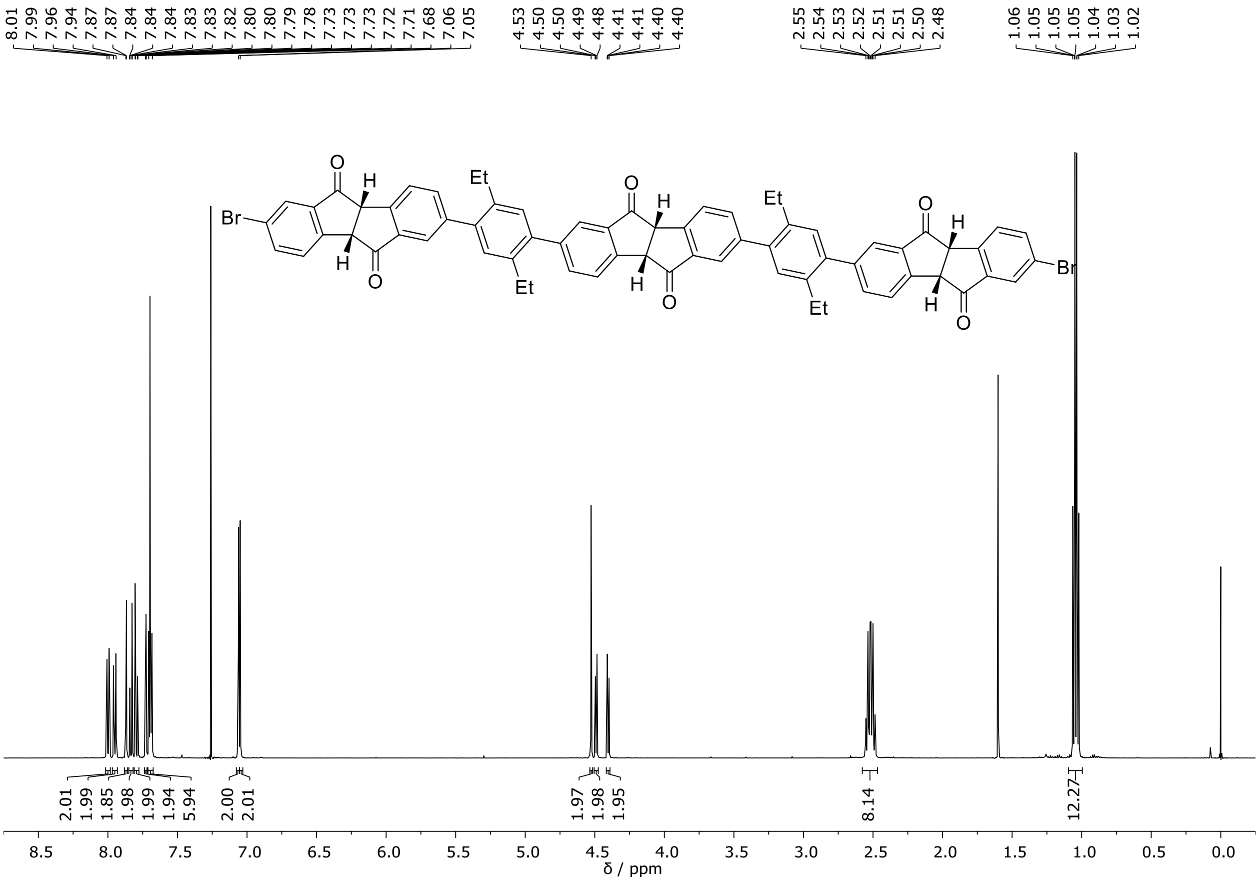

Figure S40 ^{1}H NMR spectrum of **(*S*,*S*)³-12** in $CDCl_3$ (500 MHz).

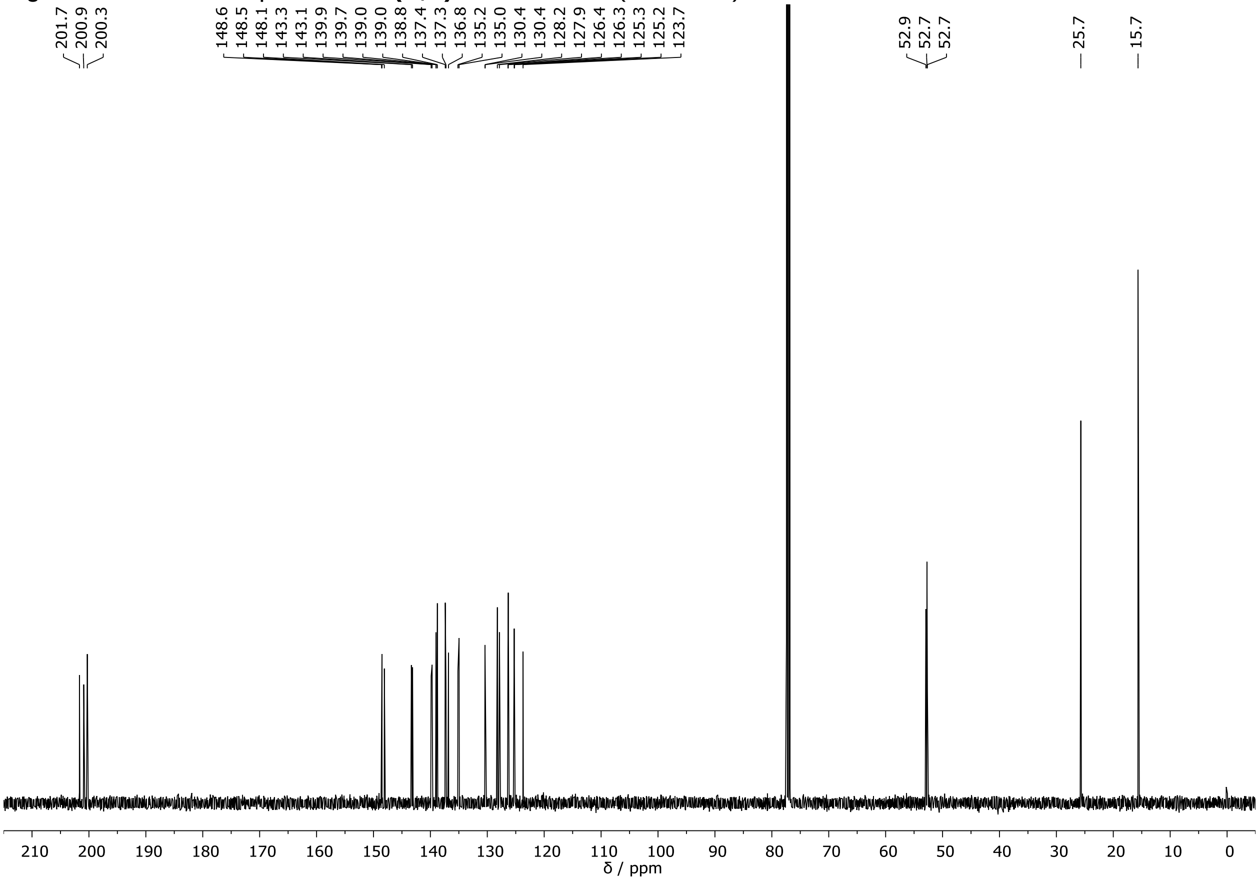

Figure S41. ^{13}C NMR spectrum of **(*S*,*S*)³-12** in $CDCl_3$ (126 MHz).

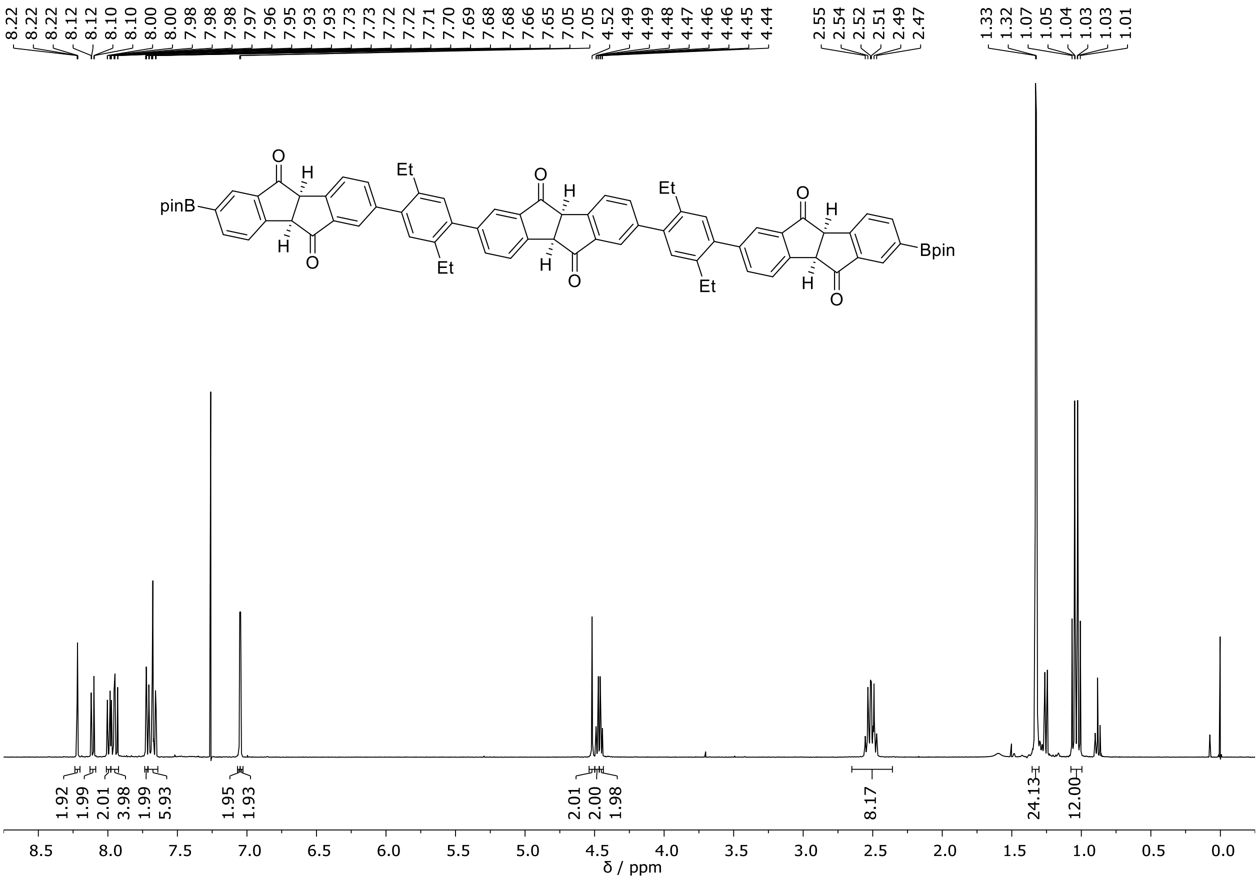

Figure S42. ^{1}H NMR spectrum of **(*R*,*R*)³-13** in $CDCl_3$ (400 MHz).

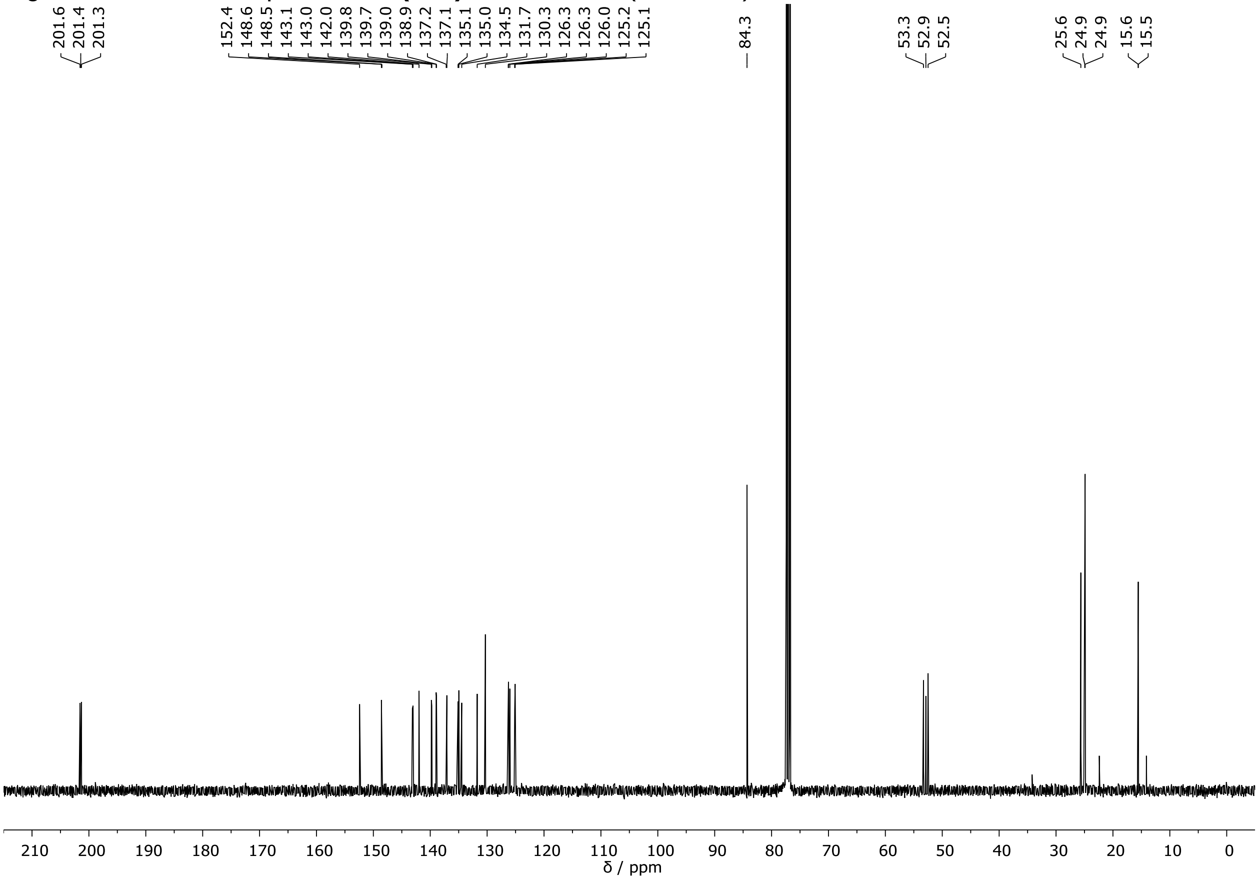

Figure S43. ^{13}C NMR spectrum of **(*R*,*R*)³-13** in $CDCl_3$ (101 MHz).

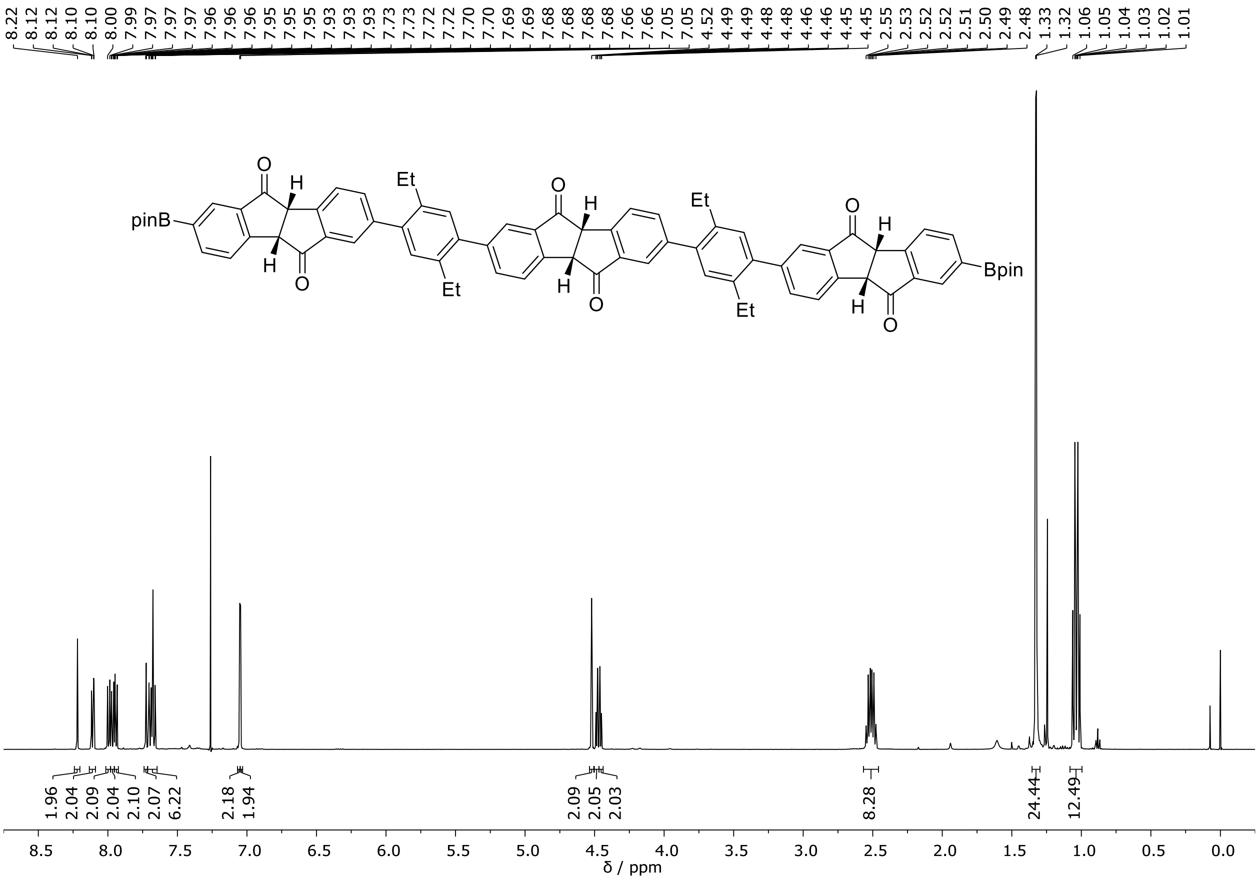

Figure S44. ^{1}H NMR spectrum of **(*S*,*S*)³-13** in $CDCl_3$ (500 MHz).

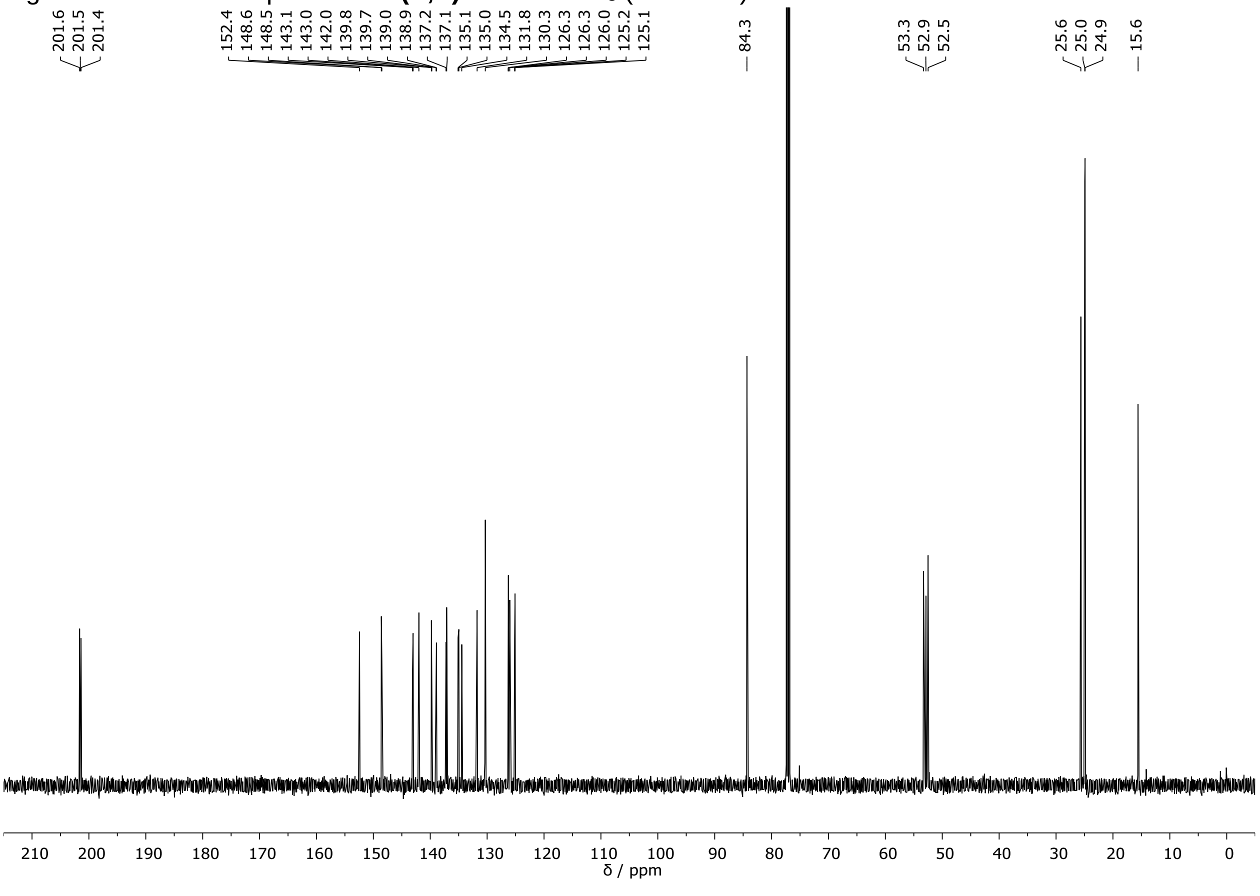

Figure S45. ^{13}C NMR spectrum of **(*S*,*S*)³-13** in $CDCl_3$ (126 MHz).

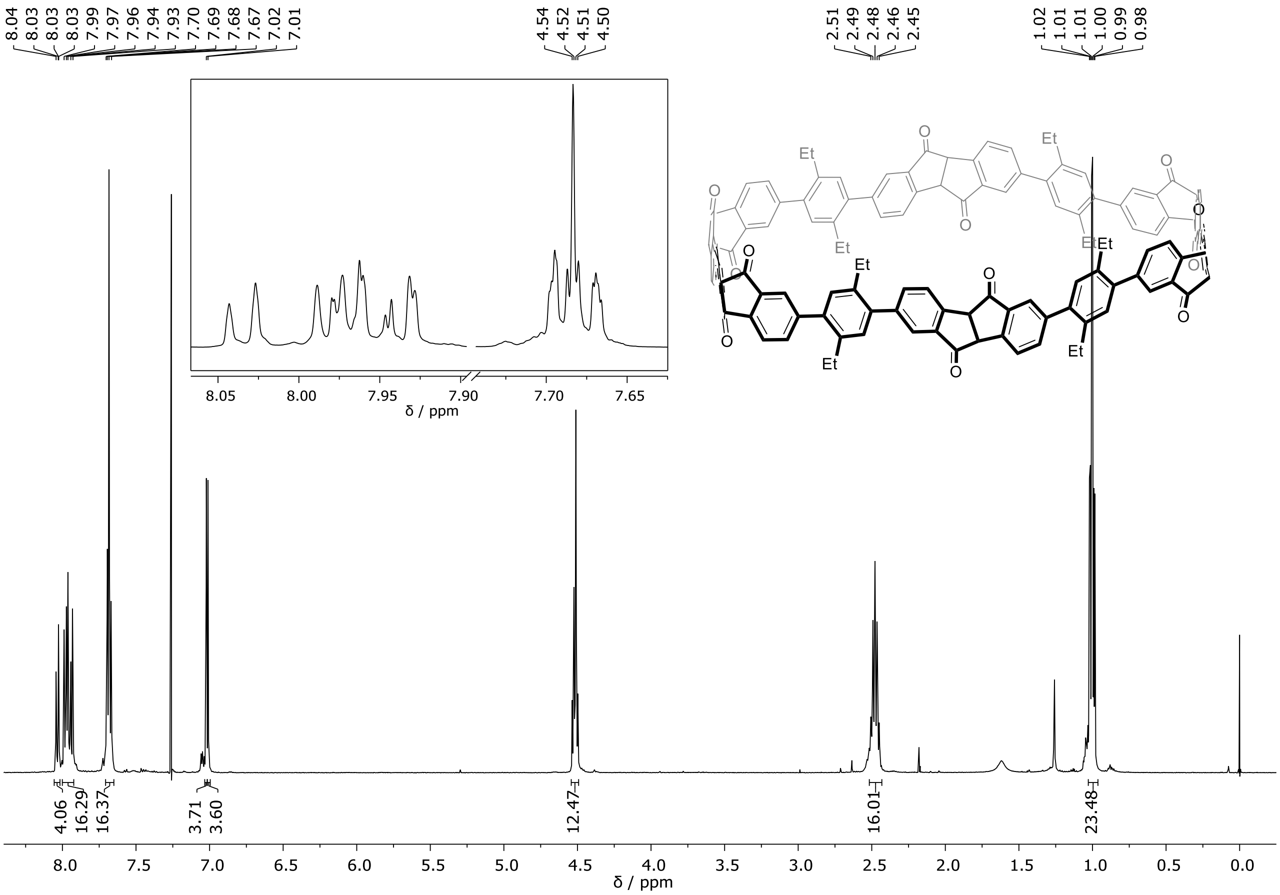

Figure S46. ^{1}H NMR spectrum of **(*R*,*R*)^{6}-14** in $CDCl_3$ (500 MHz).

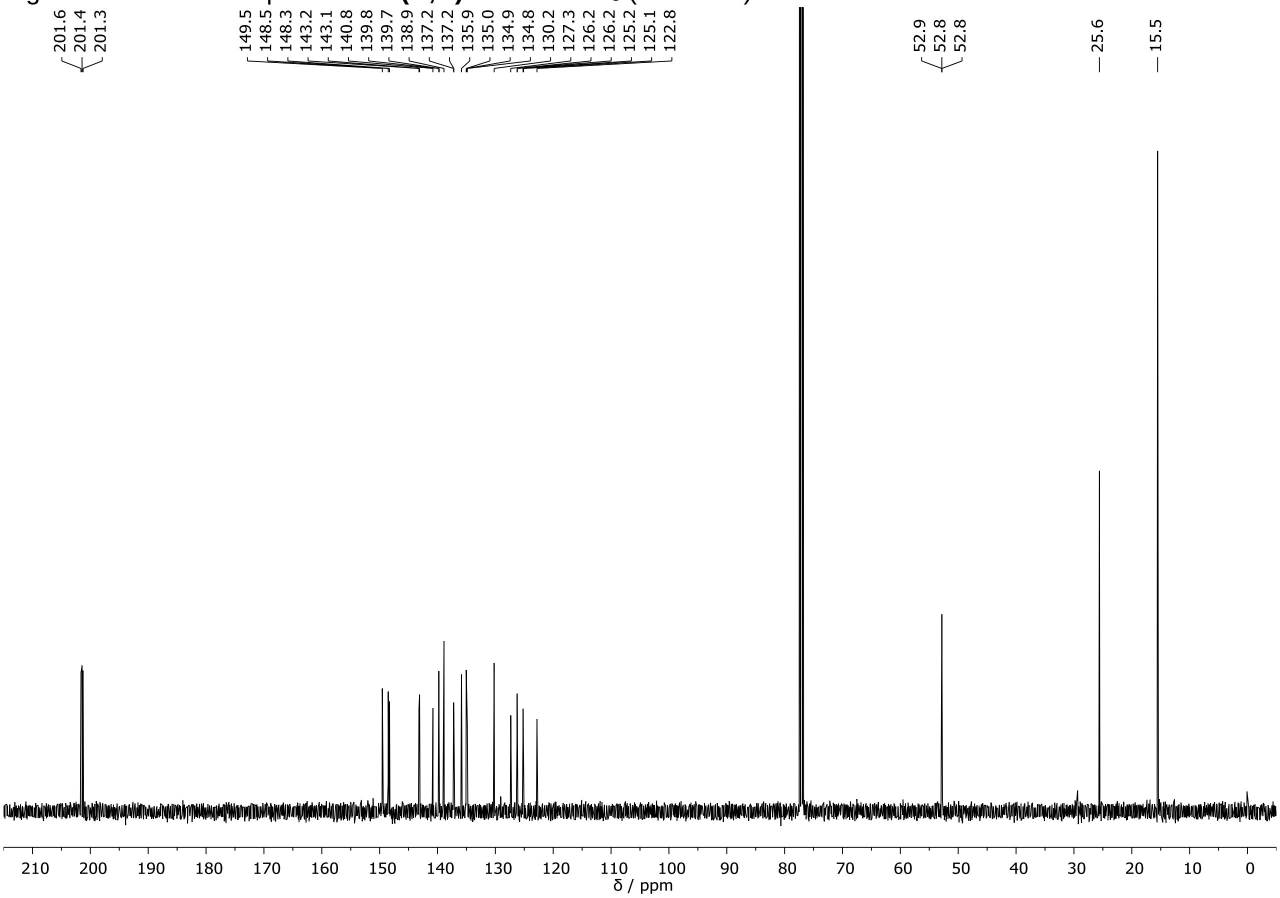

Figure S47. ^{13}C NMR spectrum of **(*R*,*R*)^{6}-14** in $CDCl_3$ (126 MHz).

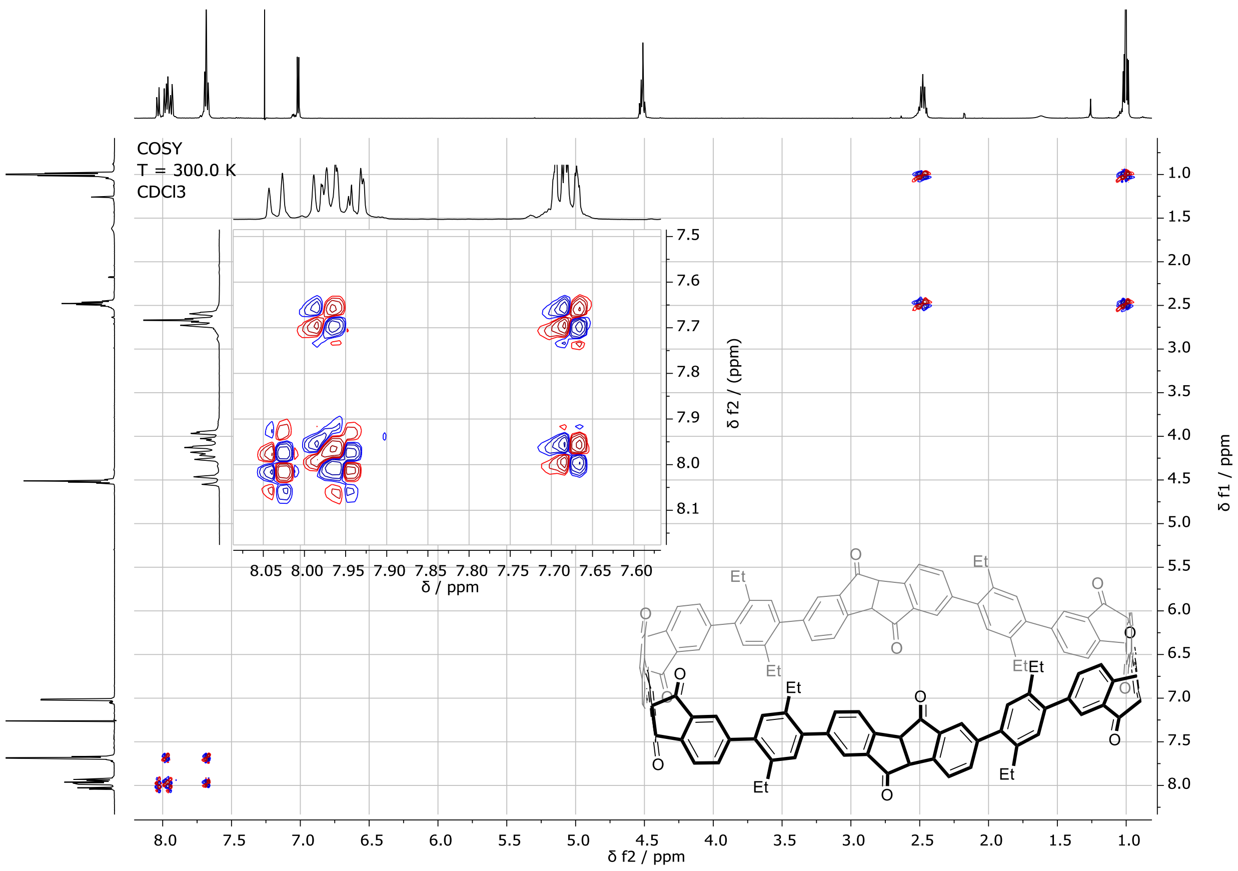

Figure S48. H,H-COSY spectra of **(*R*,*R*)[6]-14** in $CDCl_3$ (500 MHz).

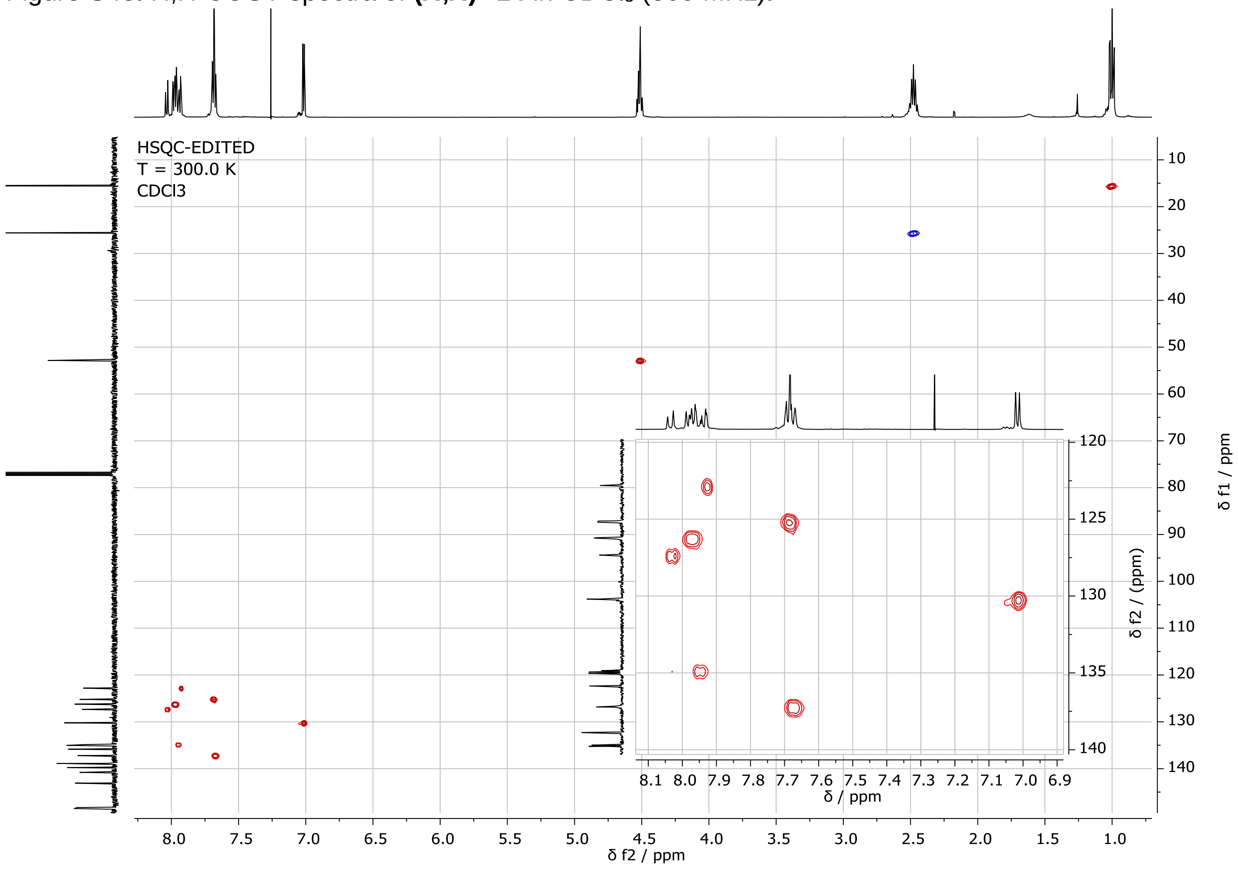

Figure S49. Edited HSQC spectra of **(*R*,*R*)[6]-14** in $CDCl_3$ (500/126 MHz). Blue: CH_2 groups, red: CH or CH_3 groups.

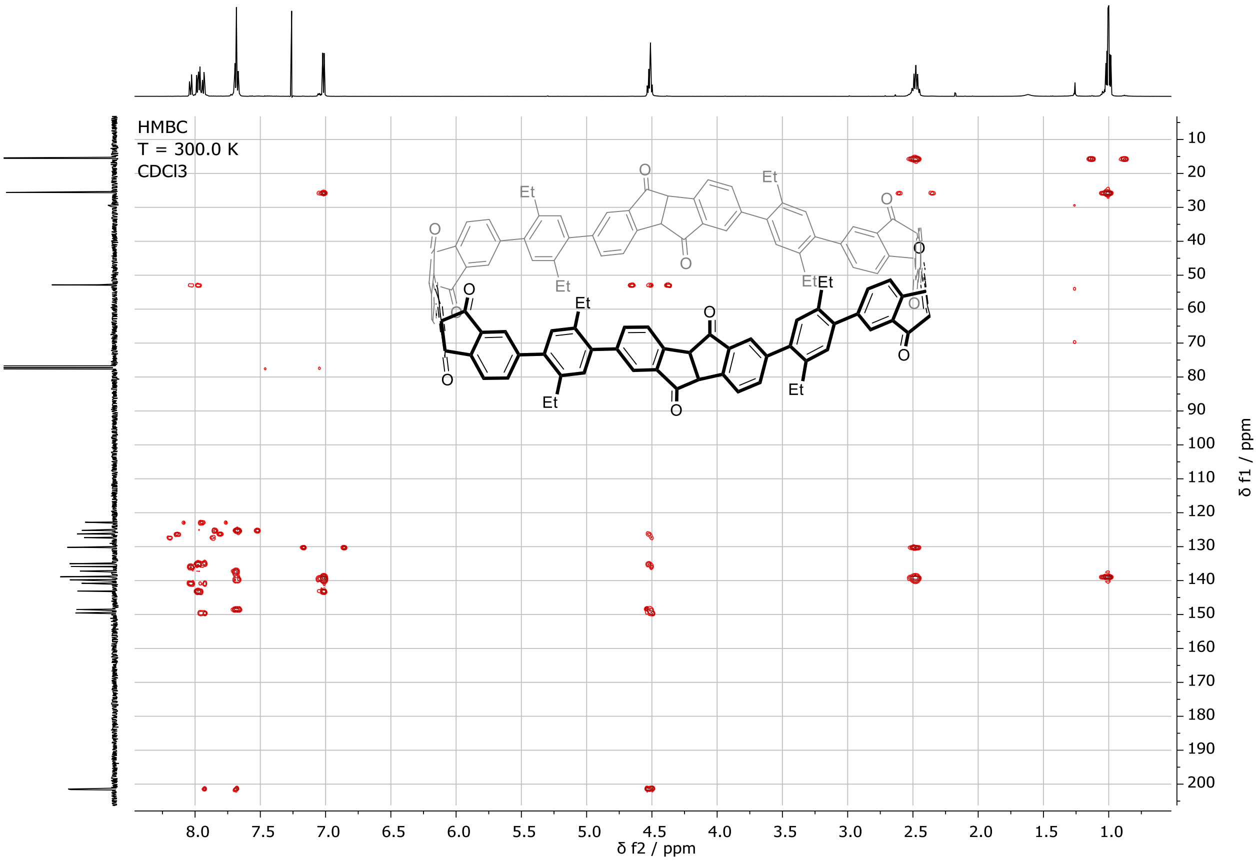

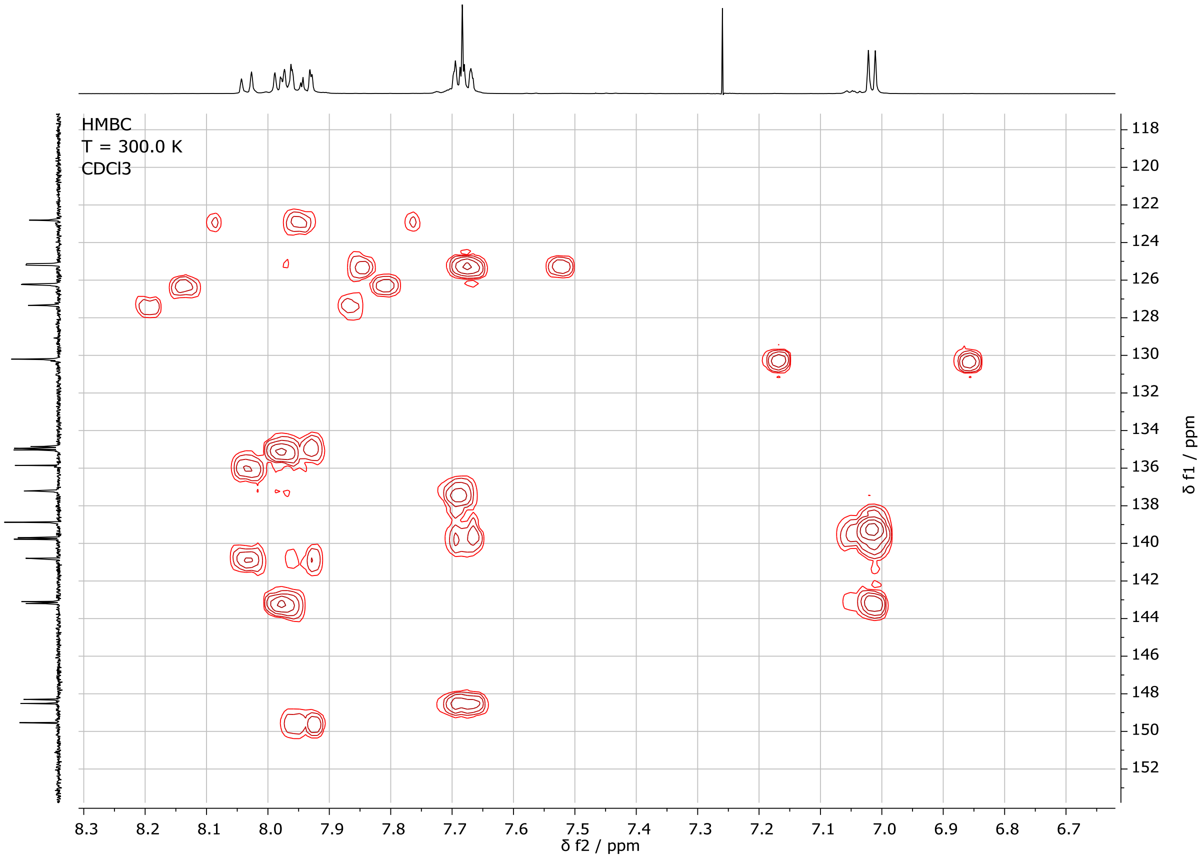

Figure S50. HMBC spectra of **(*R*,*R*)[6]-14** in $CDCl_3$ (500/126 MHz).

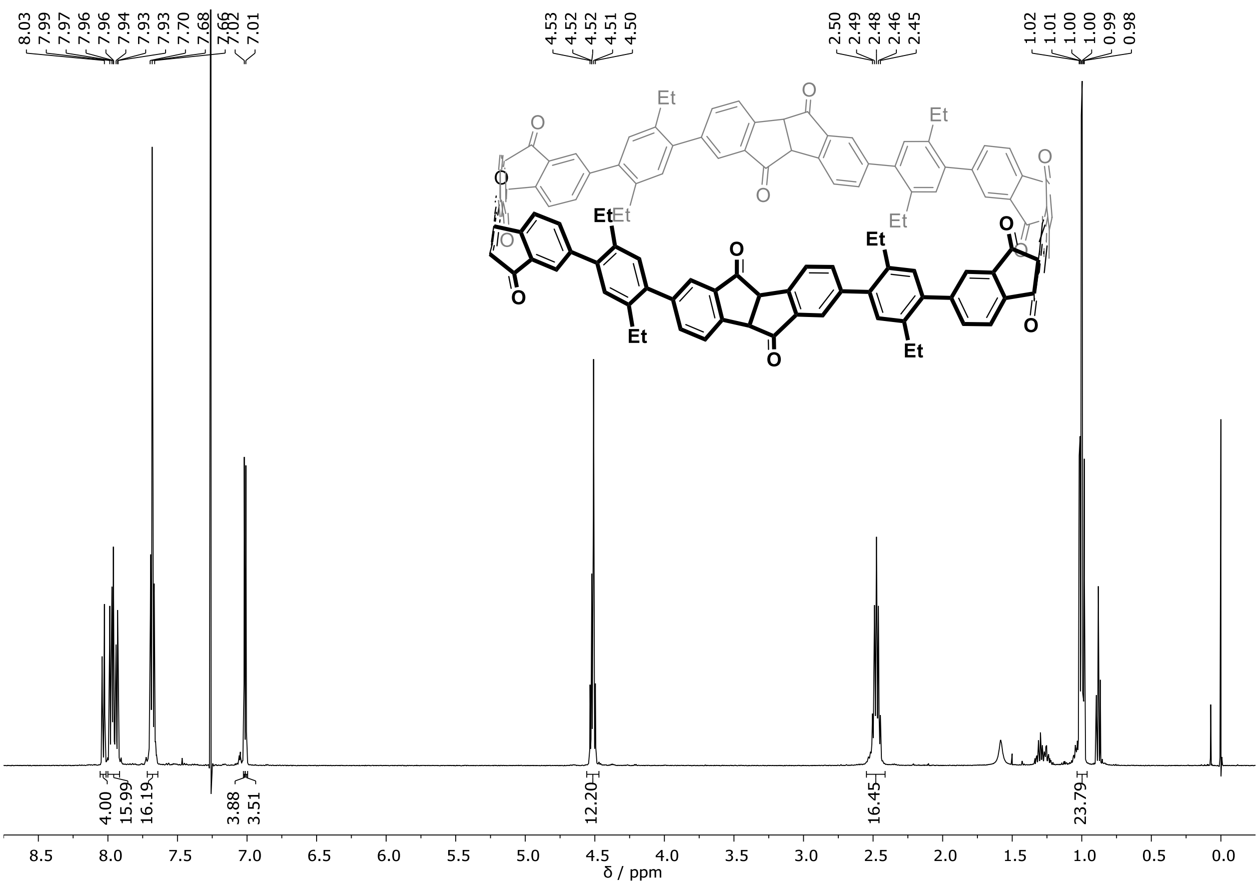

Figure S51. ^{1}H NMR spectrum of **(*S*,*S*)^{6}-14** in $CDCl_3$ (500 MHz).

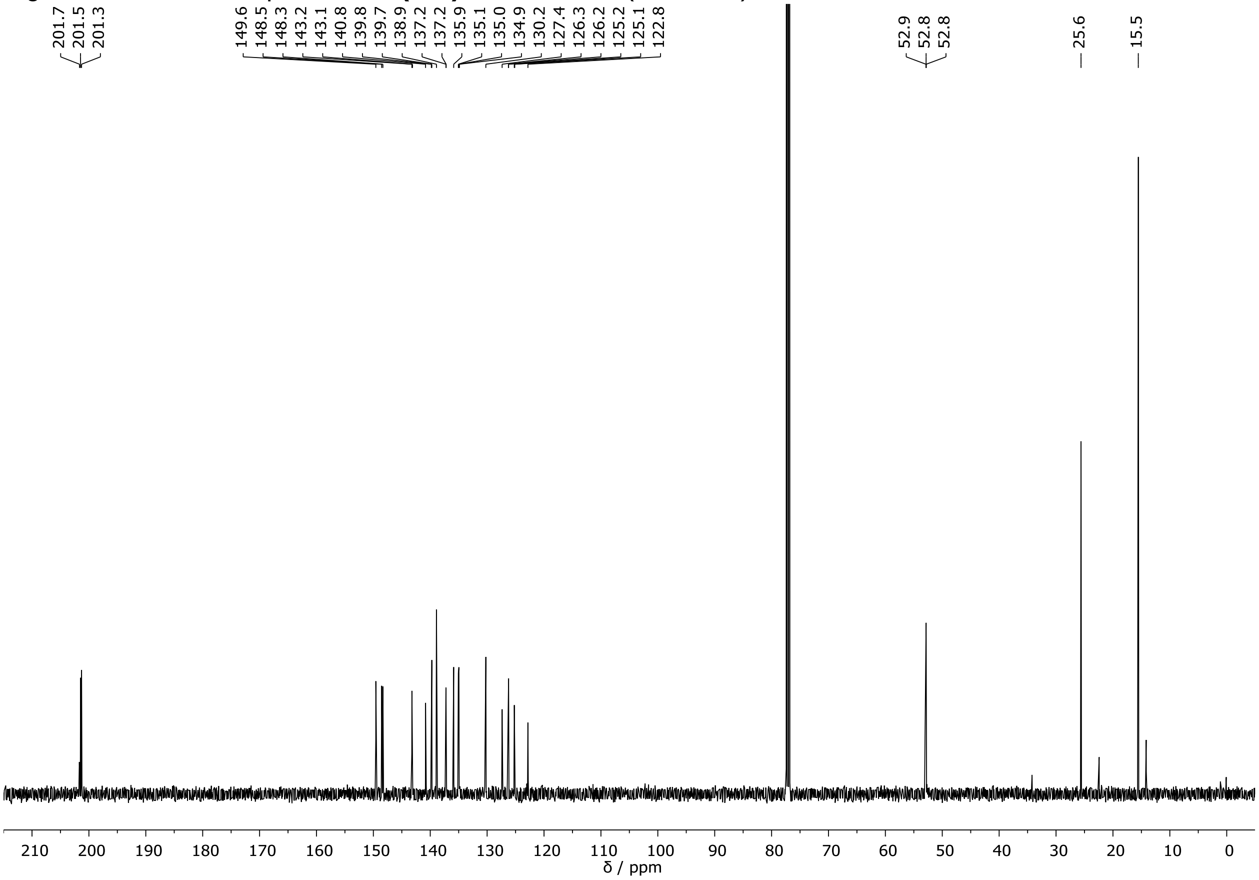

Figure S52. ^{13}C NMR spectrum of **(*S*,*S*)^{6}-14** in $CDCl_3$ (126 MHz).

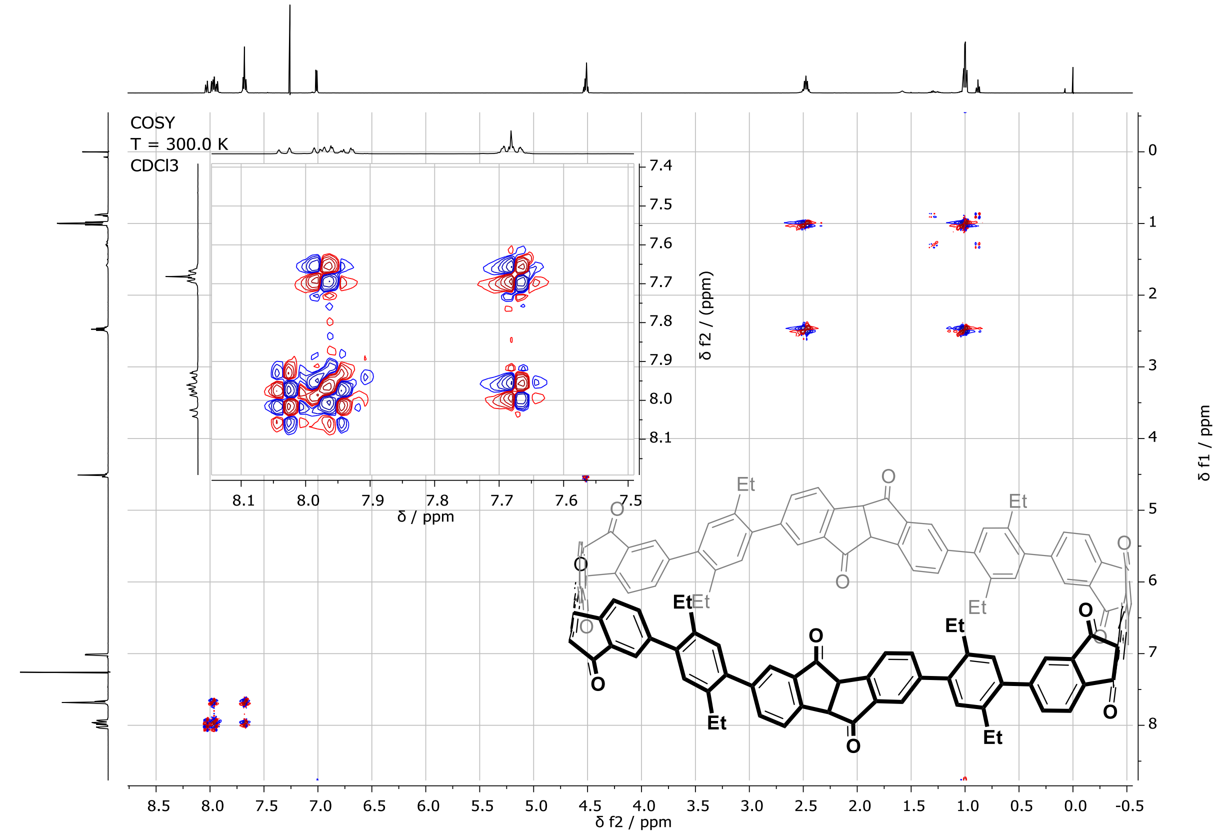

Figure S53. H,H-COSY spectra of **(*S*,*S*)[6]-14** in $CDCl_3$ (500 MHz).

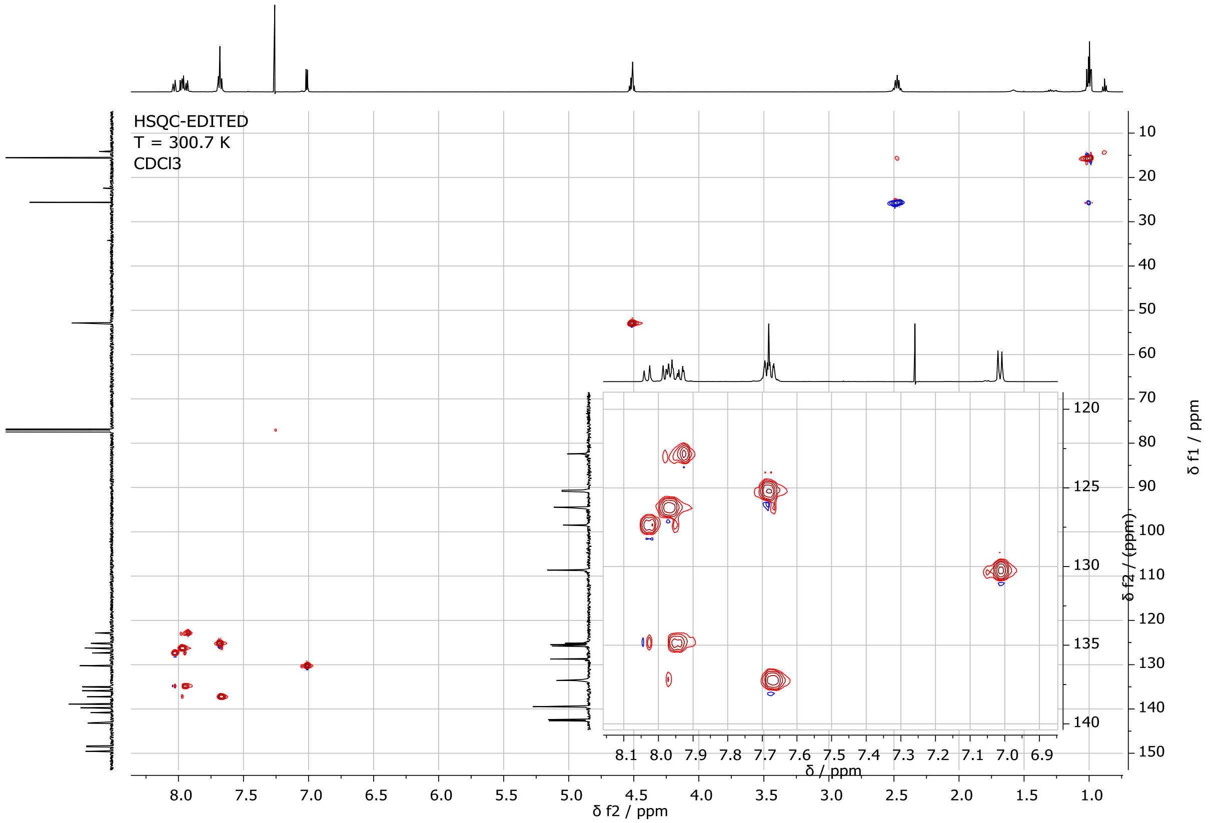

Figure S54. Edited HSQC spectra of **(*S*,*S*)[6]-14** in $CDCl_3$ (500/126 MHz). Blue: CH_2 groups, red: CH or CH_3 groups.

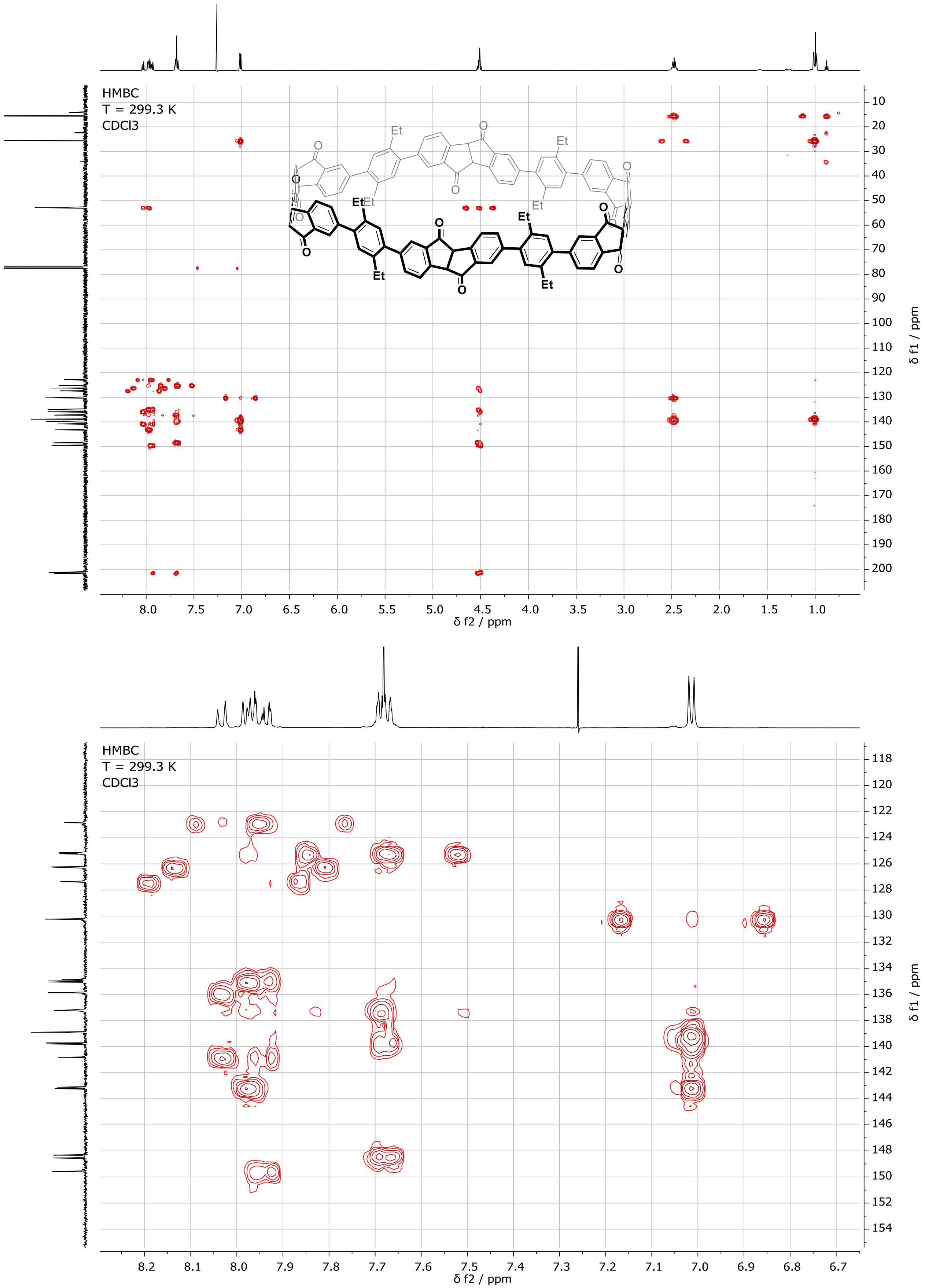

Figure S55. HMBC spectra of ***(S,S)*[6]-14** in $CDCl_3$ (500/126 MHz).

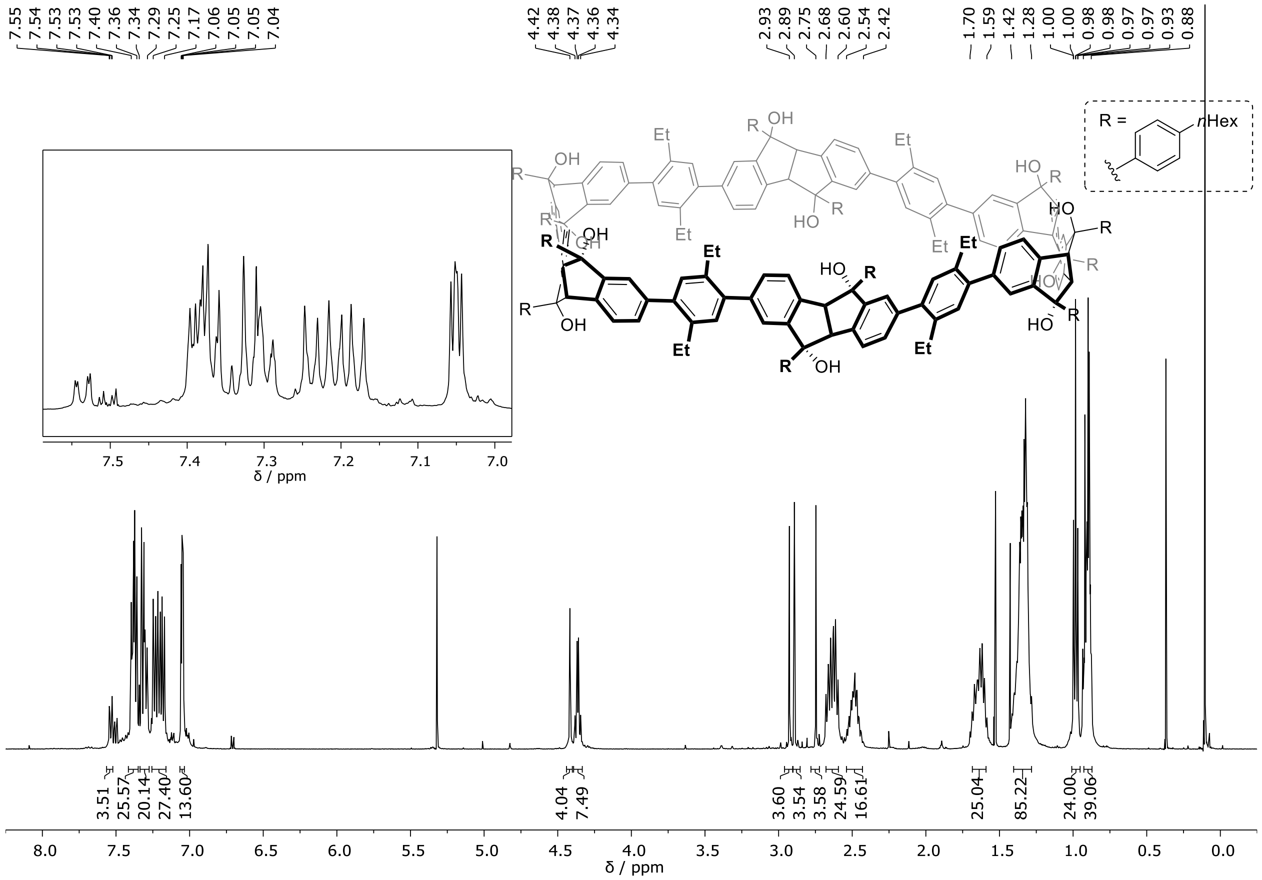

Figure S56. ^{1}H NMR spectrum of **(*R*,*R*)^{6}-(+)-16** in CD_2Cl_2 (500 MHz).

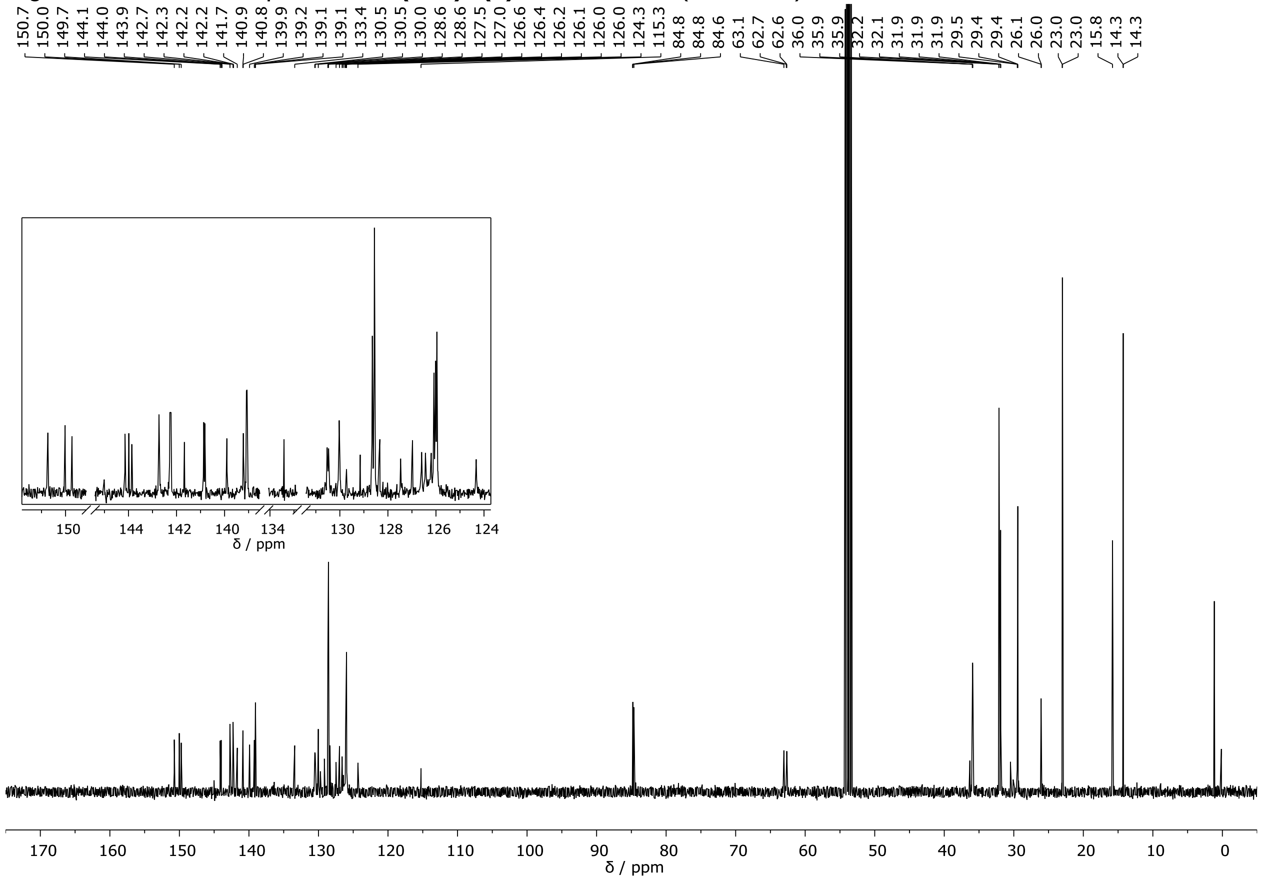

Figure S57. ^{13}C NMR spectrum of **(*R*,*R*)^{6}-(+)-16** in CD_2Cl_2 (126 MHz).

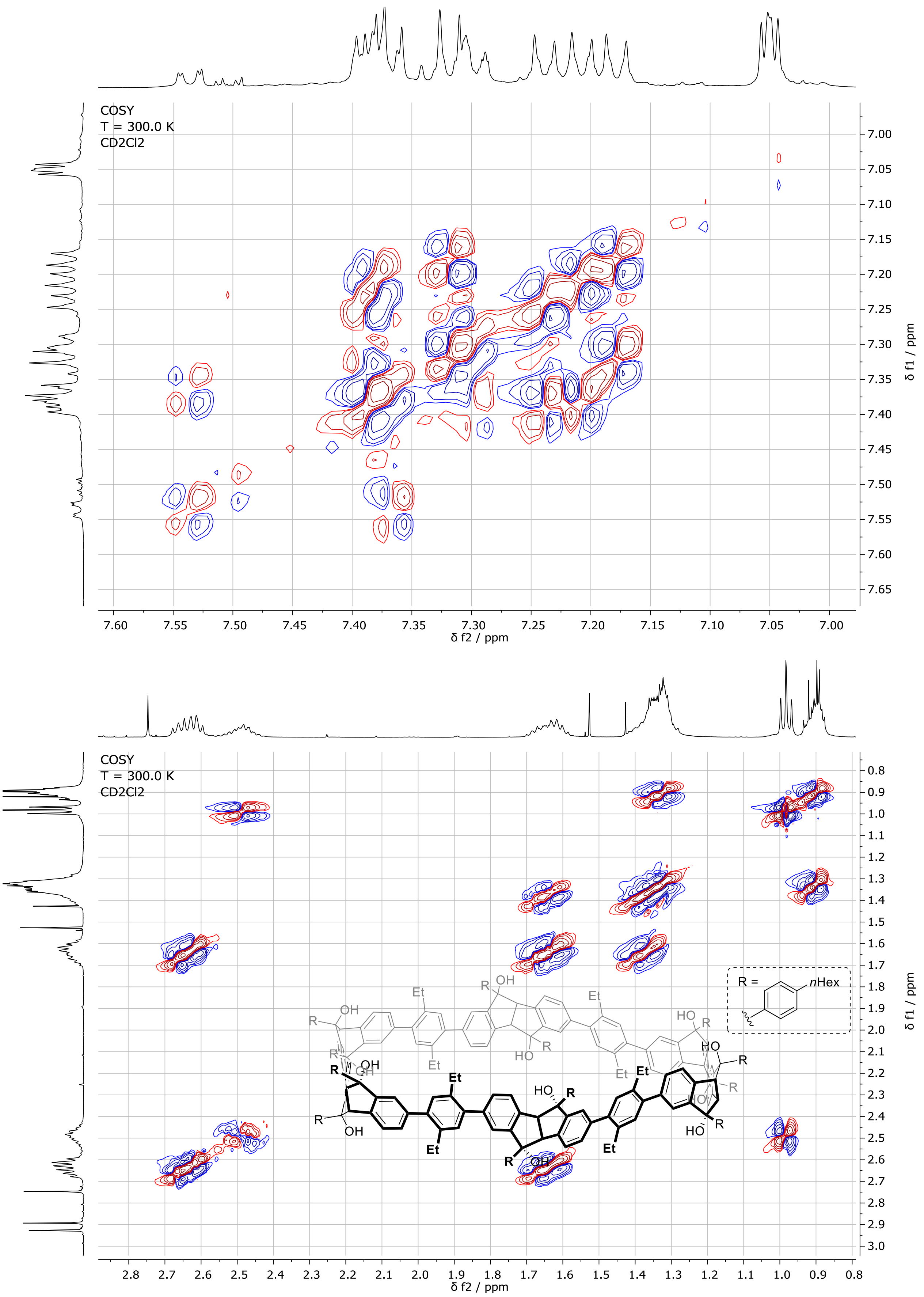

Figure S58. H,H-COSY spectra of **(*R*,*R*)^6-(+)-16** in CD_2Cl_2 (500 MHz).

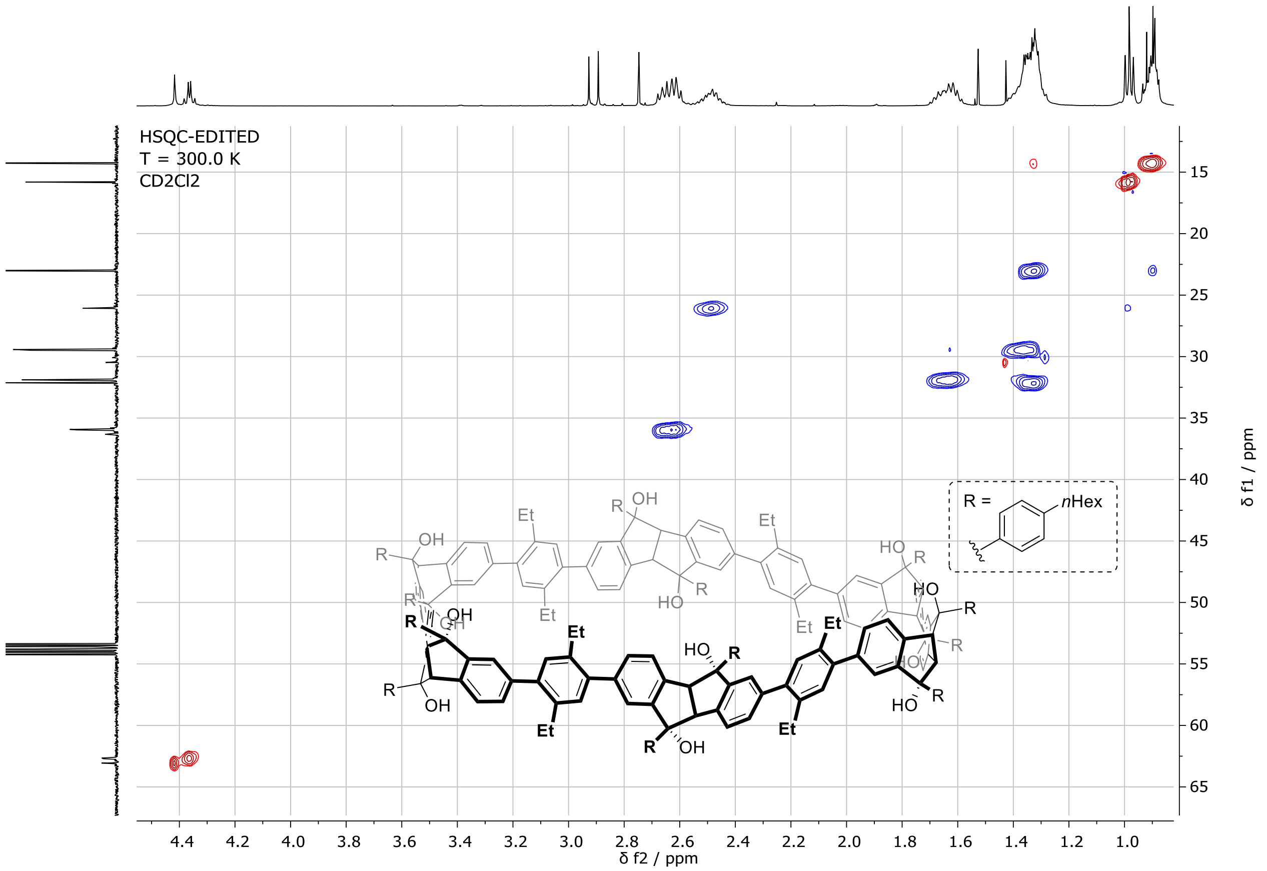

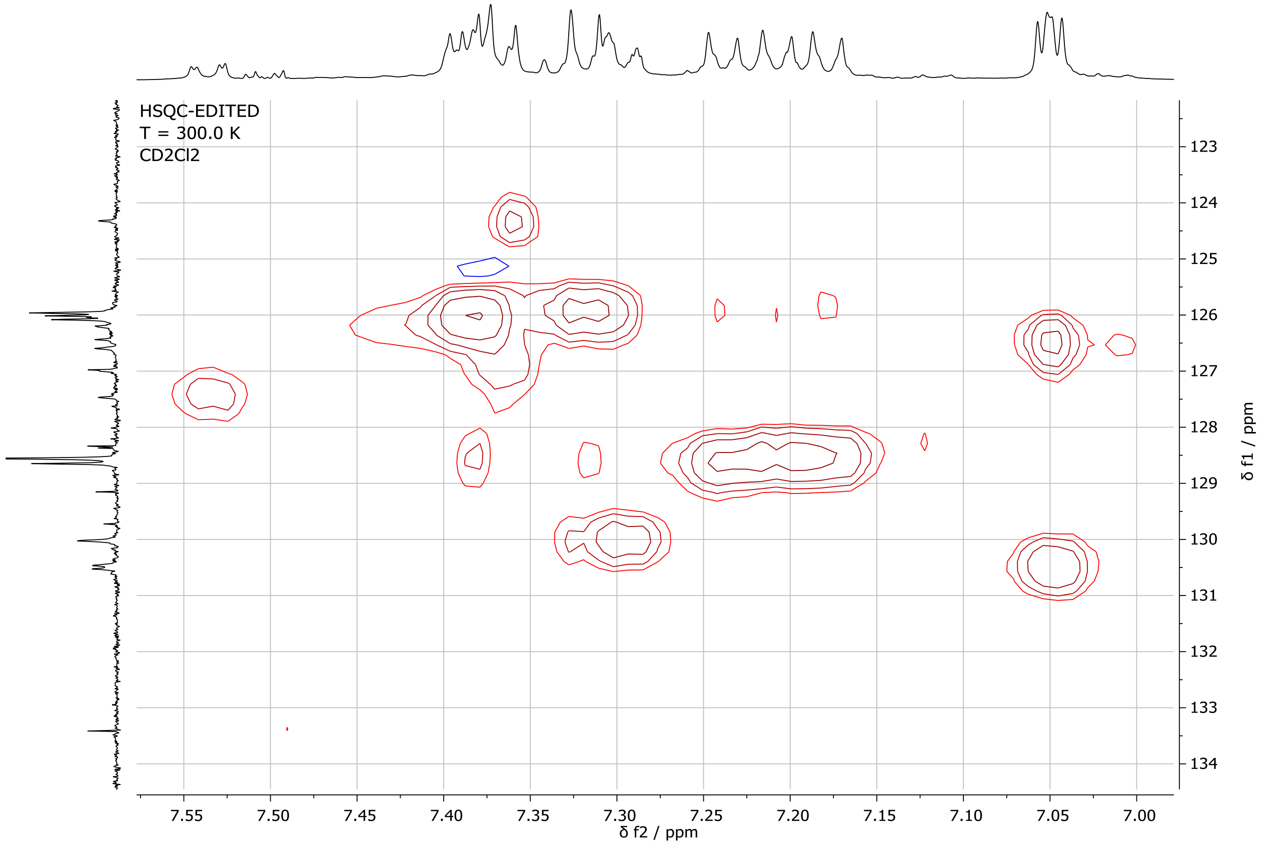

Figure S59. Edited HSQC spectra of **(*R*,*R*)^{6}-(+)-16** in CD_2Cl_2 (500/126 MHz). Blue: CH_2 groups, red: CH or CH_3 groups.

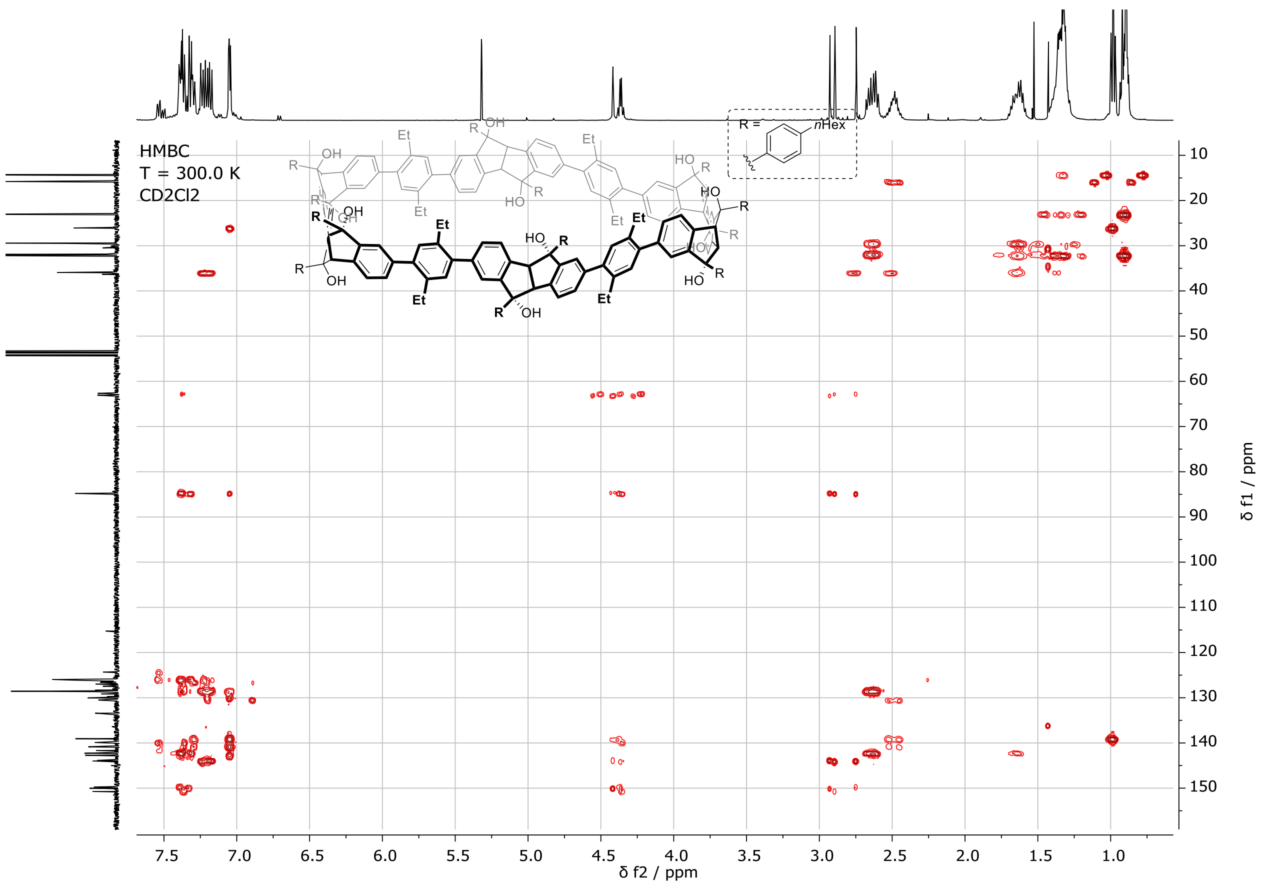

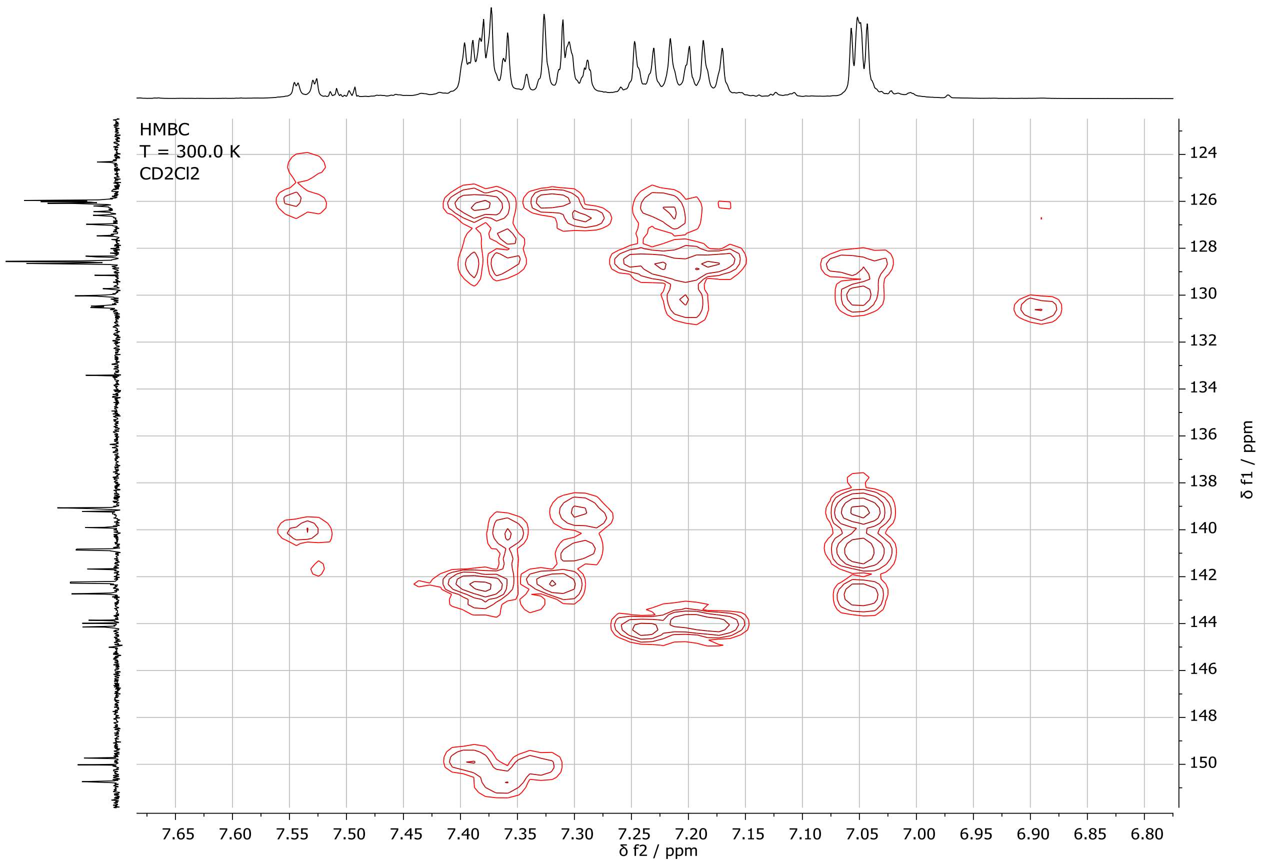

Figure S60. HMBC spectra of ***(R,R)***^{6}**-(+)-16** in CD_2Cl_2 (500/126 MHz).

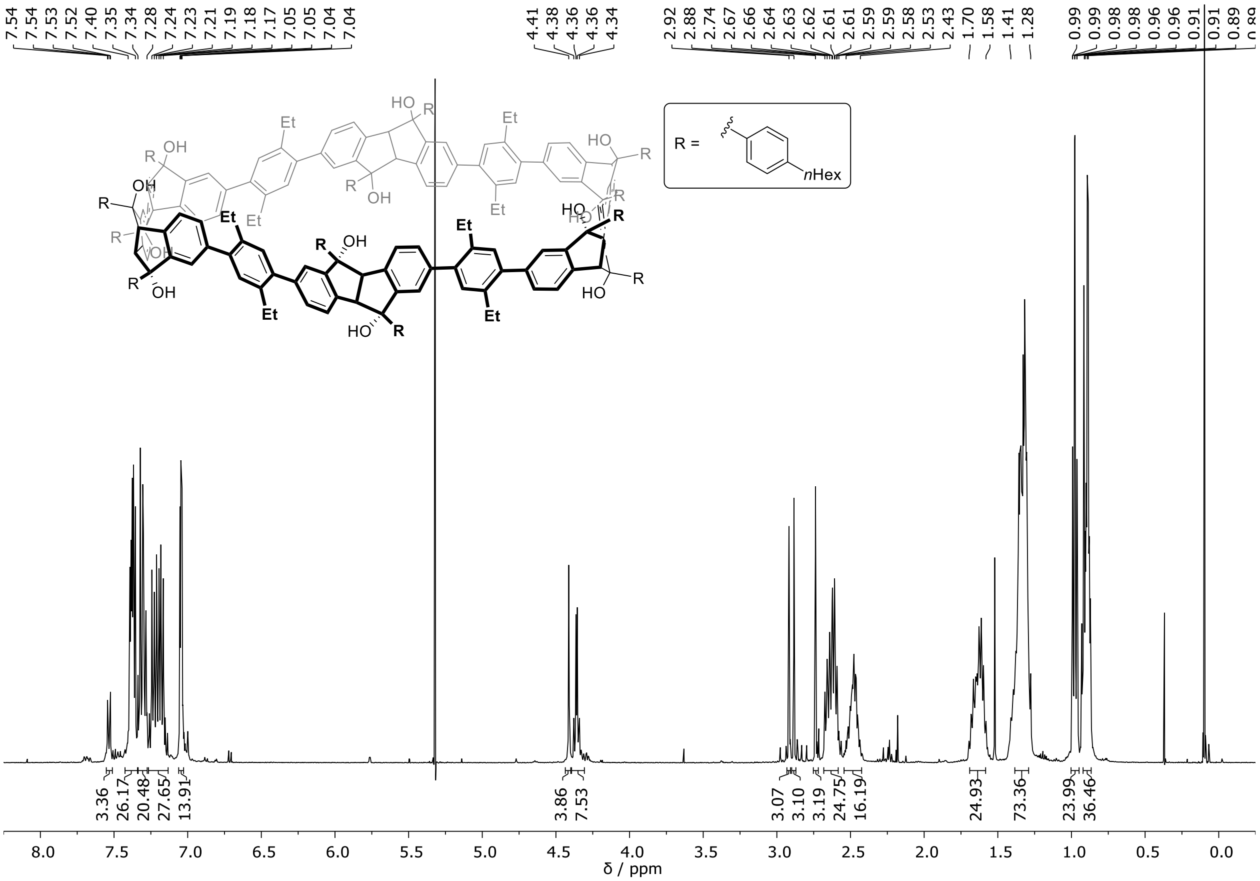

Figure S61. ^{1}H NMR spectrum of **(*S*,*S*)^{6}-(−)-16** in CD_2Cl_2 (500 MHz).

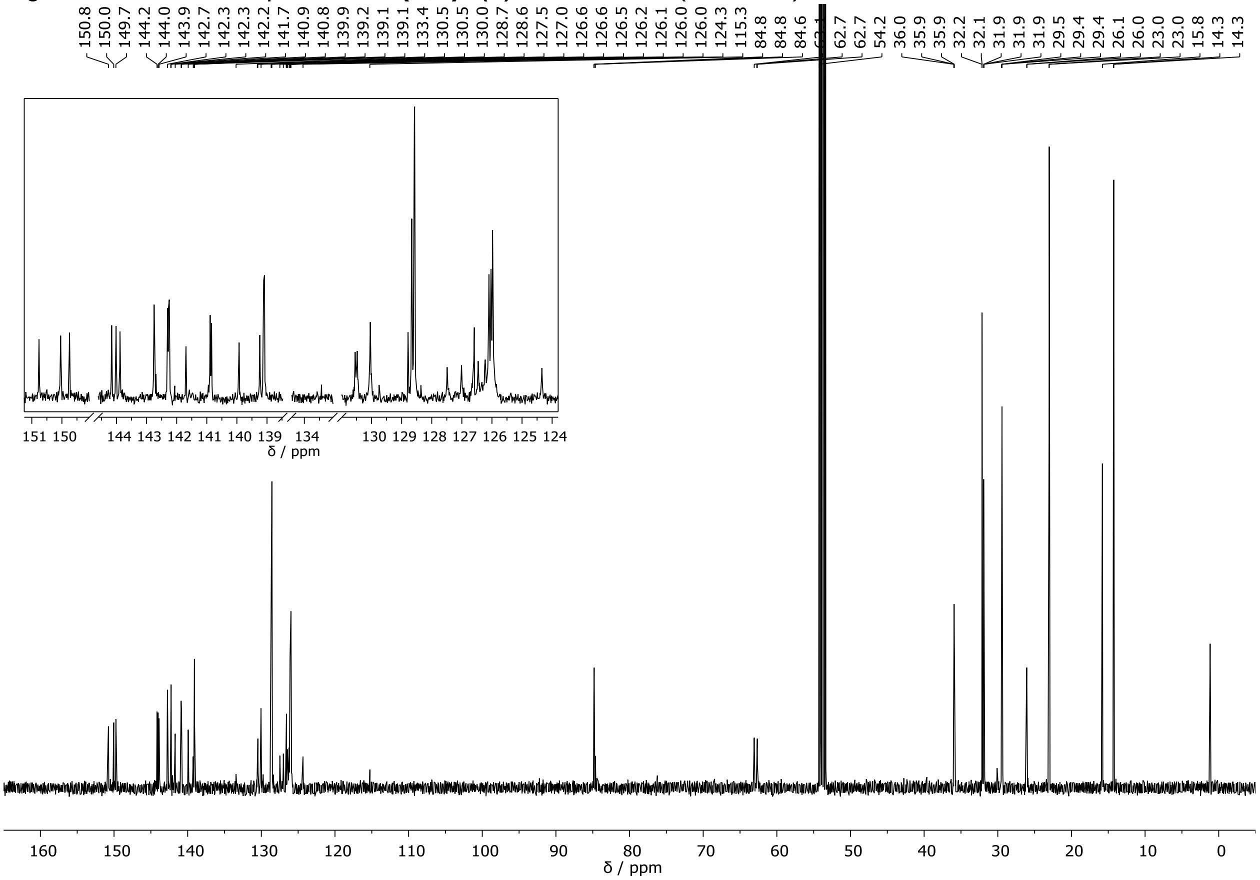

FigureS 62. ^{13}C NMR spectrum of **(*S*,*S*)^{6}-(−)-16**in CD_2Cl_2 (126 MHz).

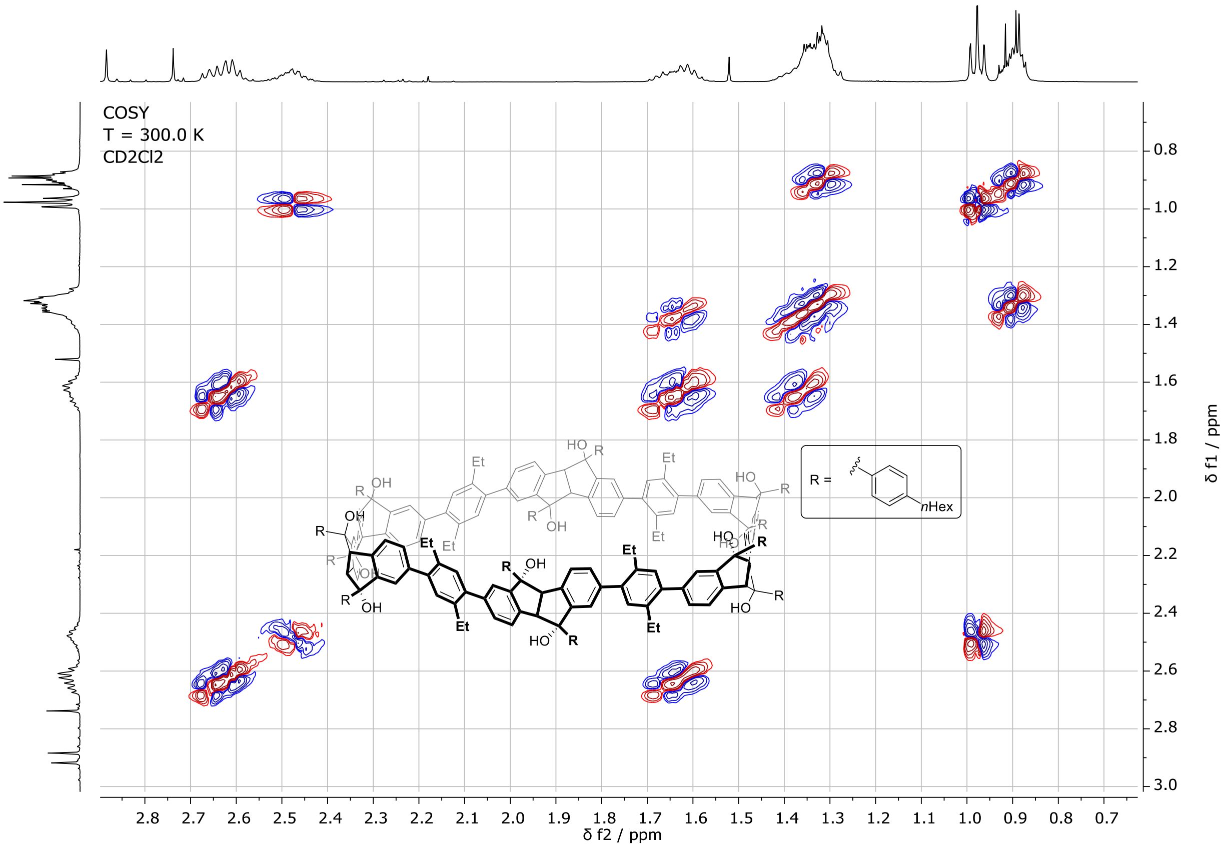

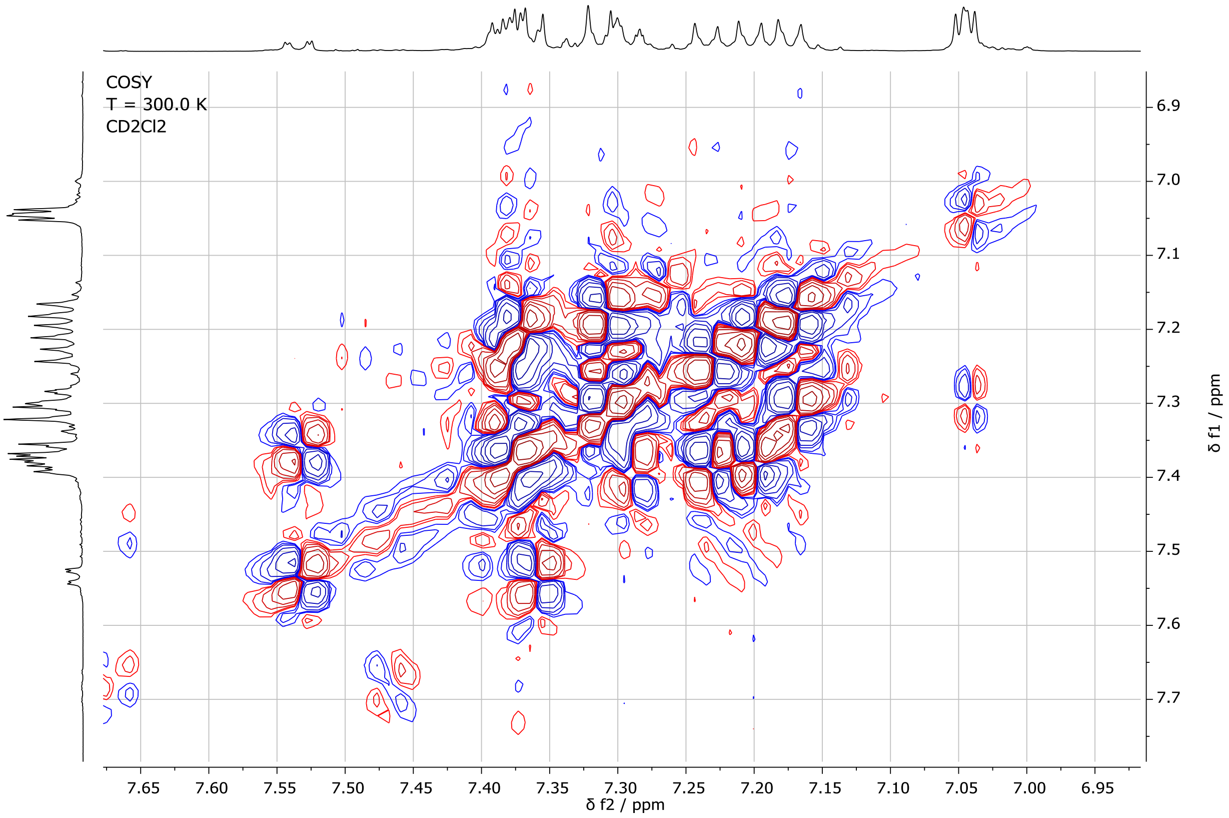

Figure S63. H,H-COSY spectra of **(*S*,*S*)^{6}-(−)-16** in CD_2Cl_2 (500 MHz).

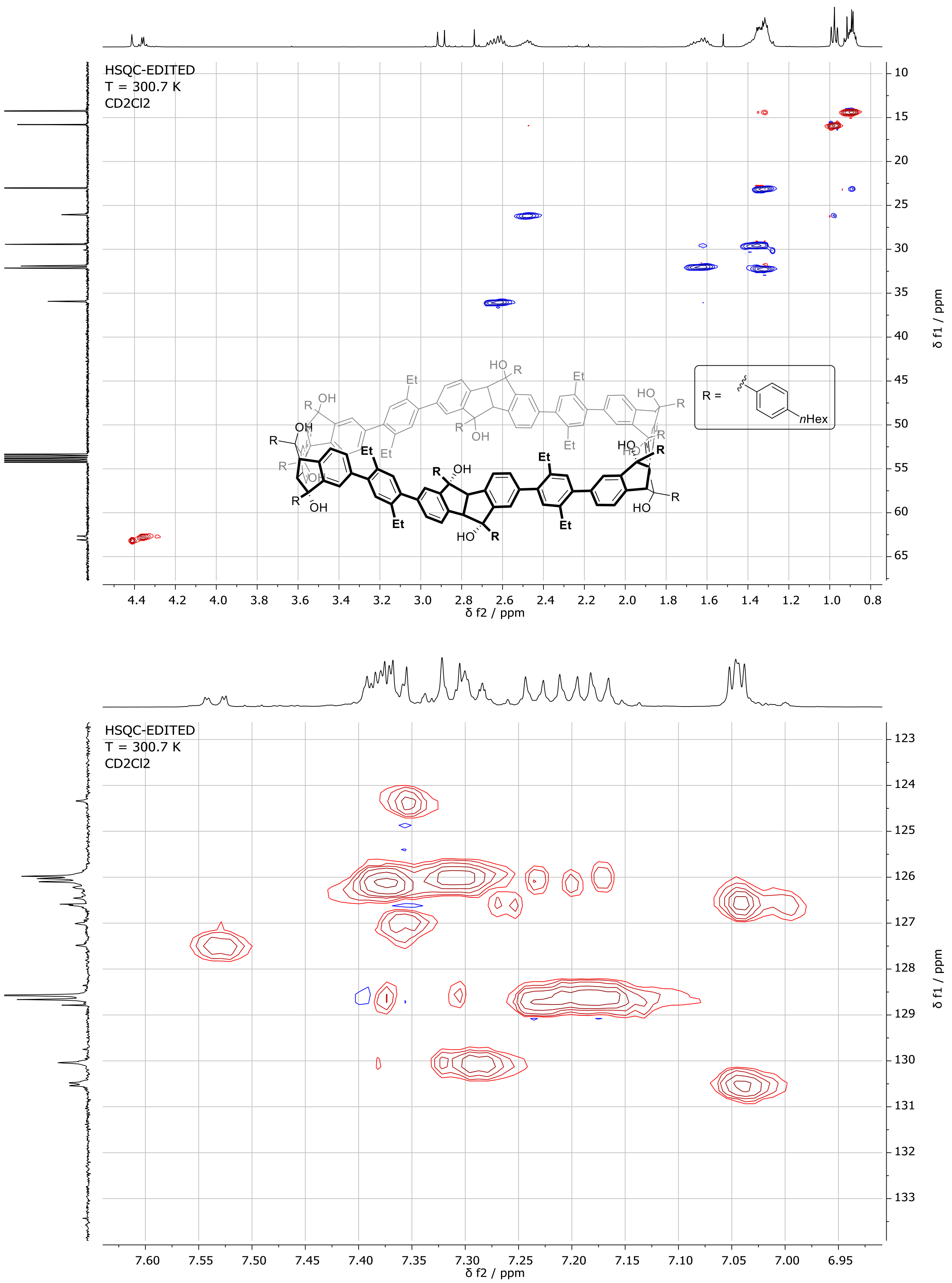

Figure S64. Edited HSQC spectra of **(*S*,*S*)⁶-(−)-16** in CD_2Cl_2 (500/126 MHz). Blue: CH_2 groups, red: CH or CH_3 groups.

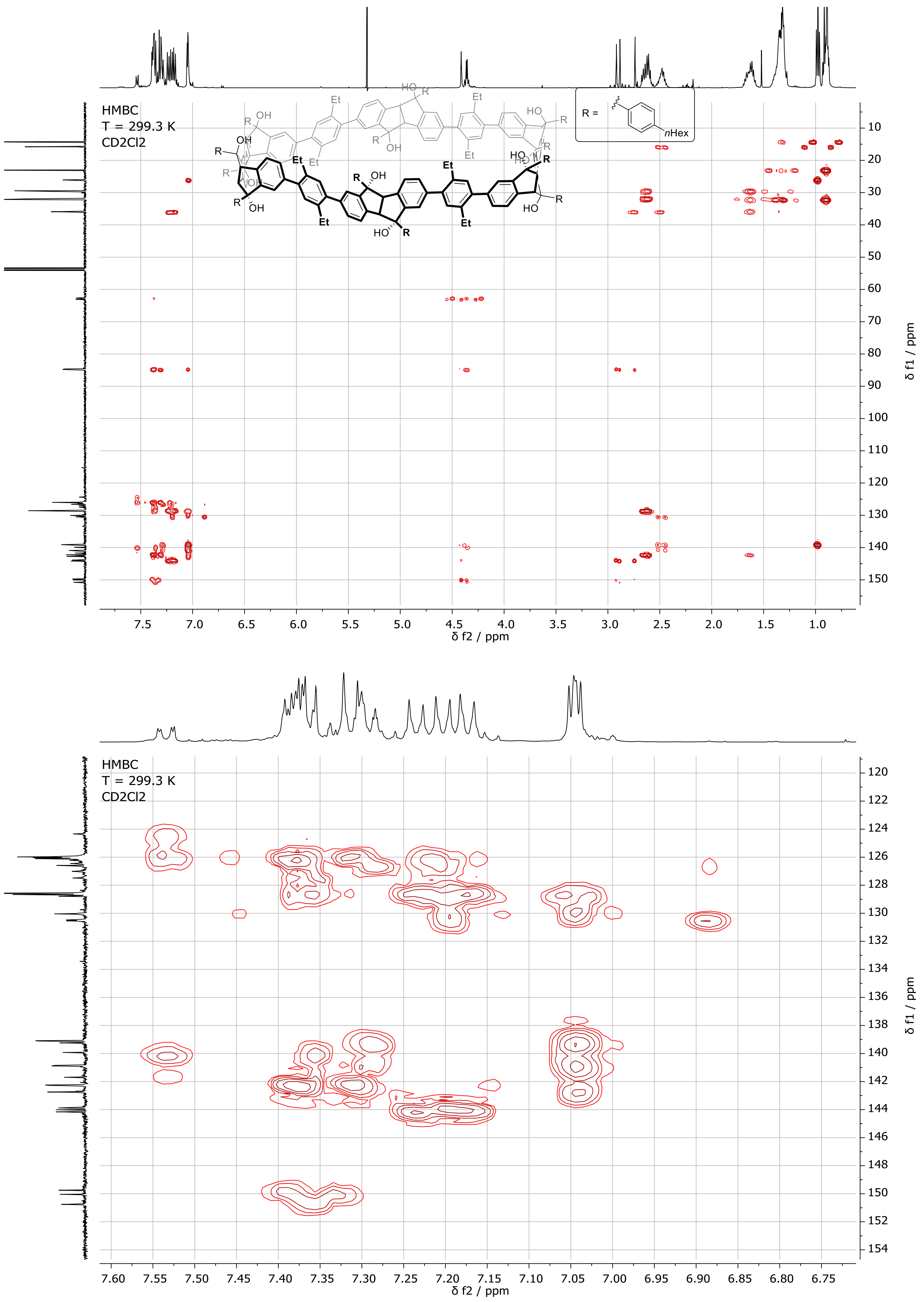

Figure S65. HMBC spectra of ***(S,S)*[6]-(−)-16** in CD_2Cl_2 (500/126 MHz).

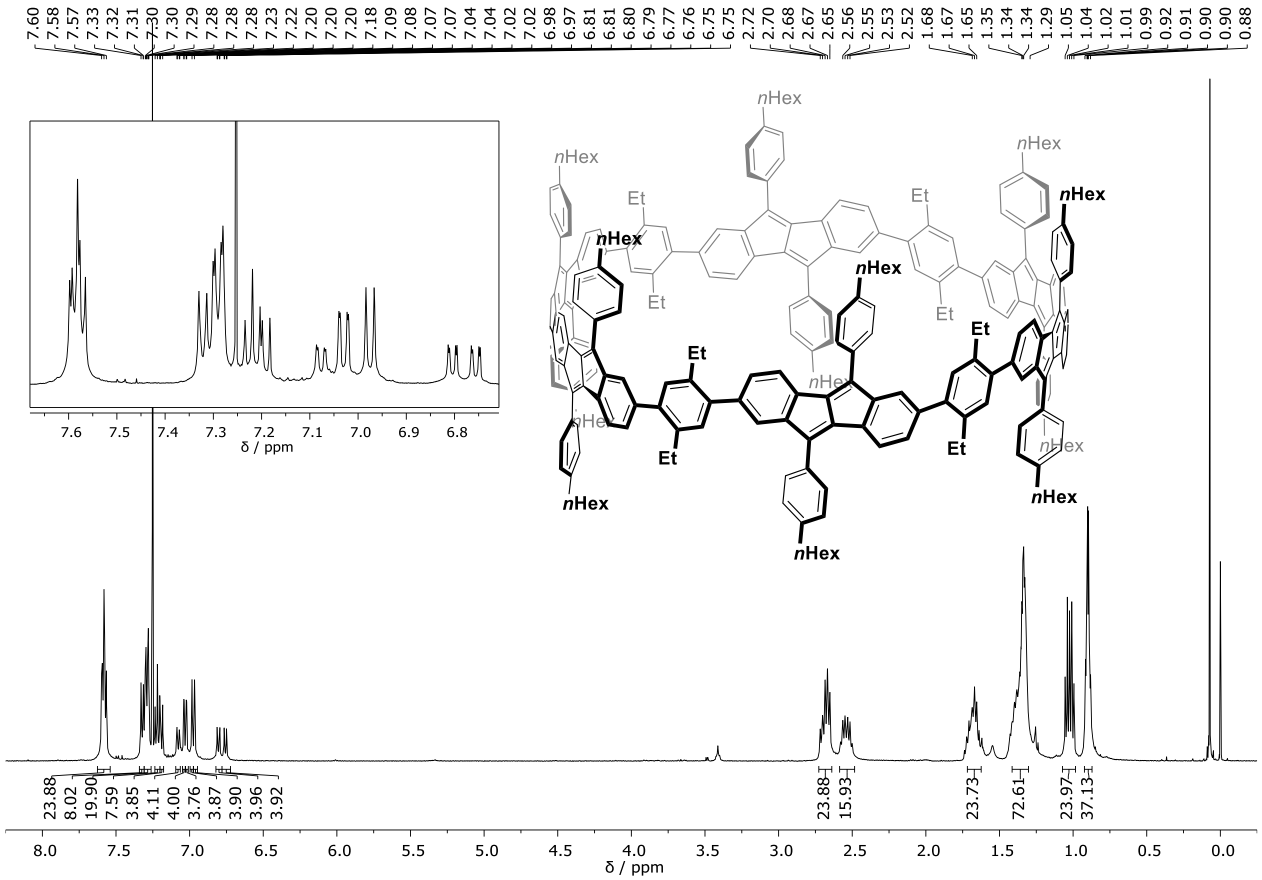

Figure S66. ^{1}H NMR spectrum of **(+)-1** in $CDCl_3$ (500 MHz).

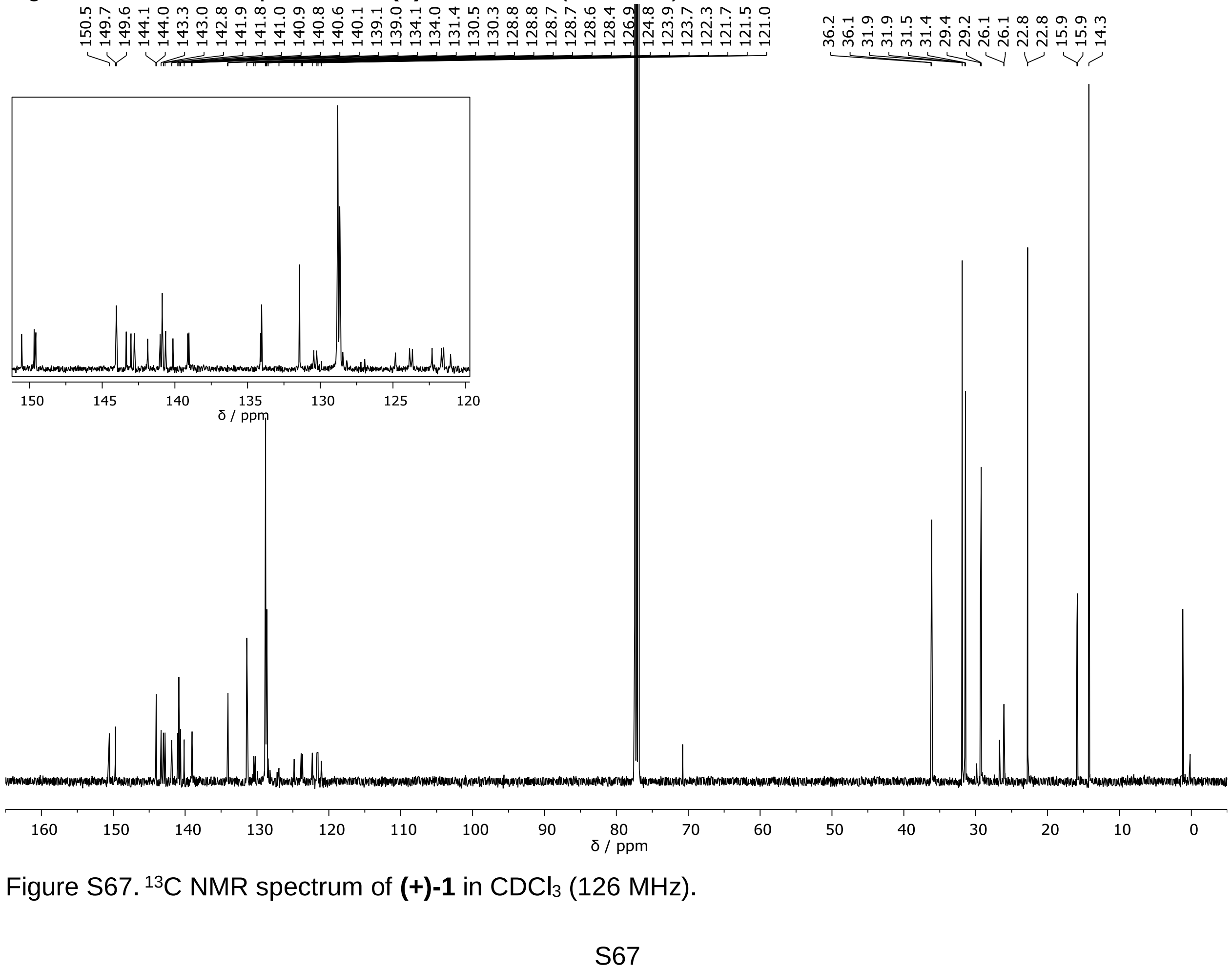

Figure S67. ^{13}C NMR spectrum of **(+)-1** in $CDCl_3$ (126 MHz).

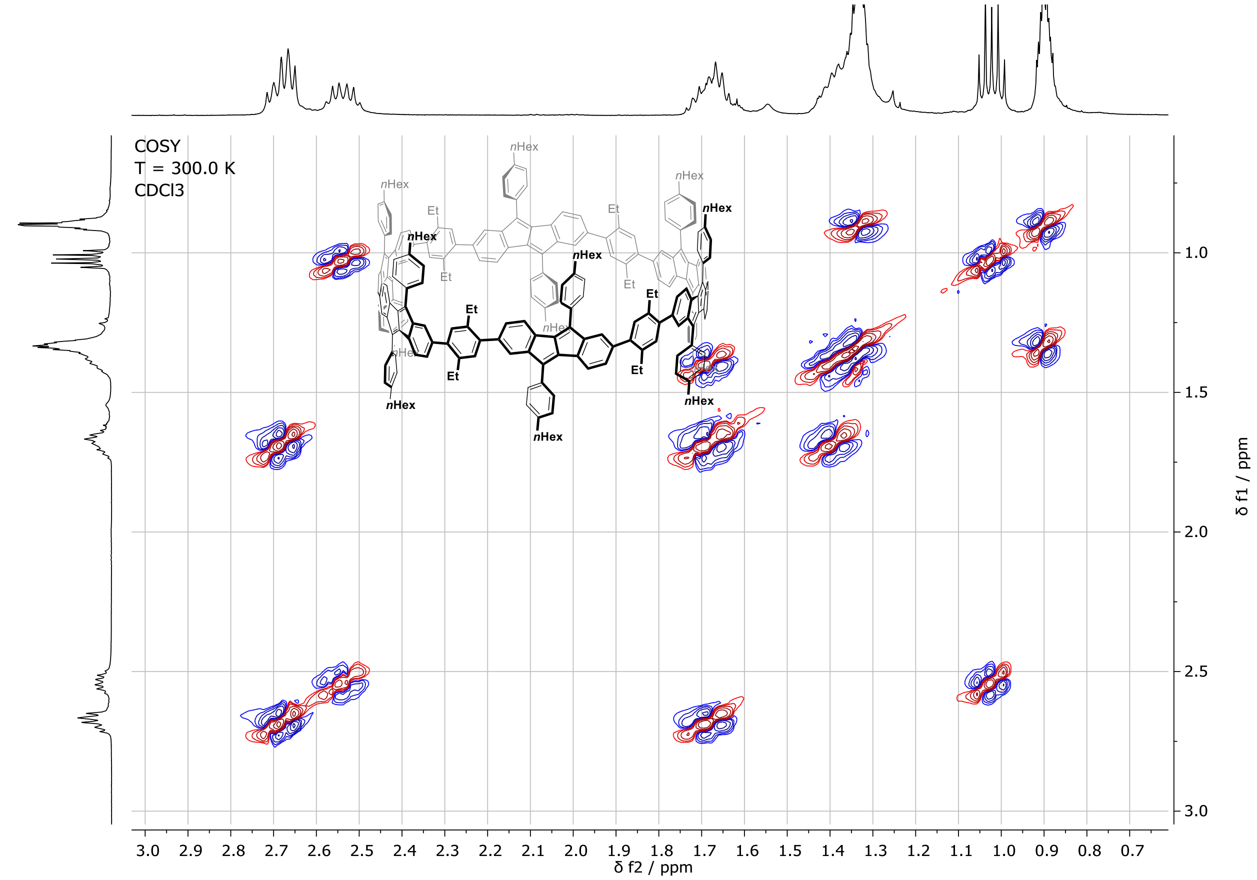

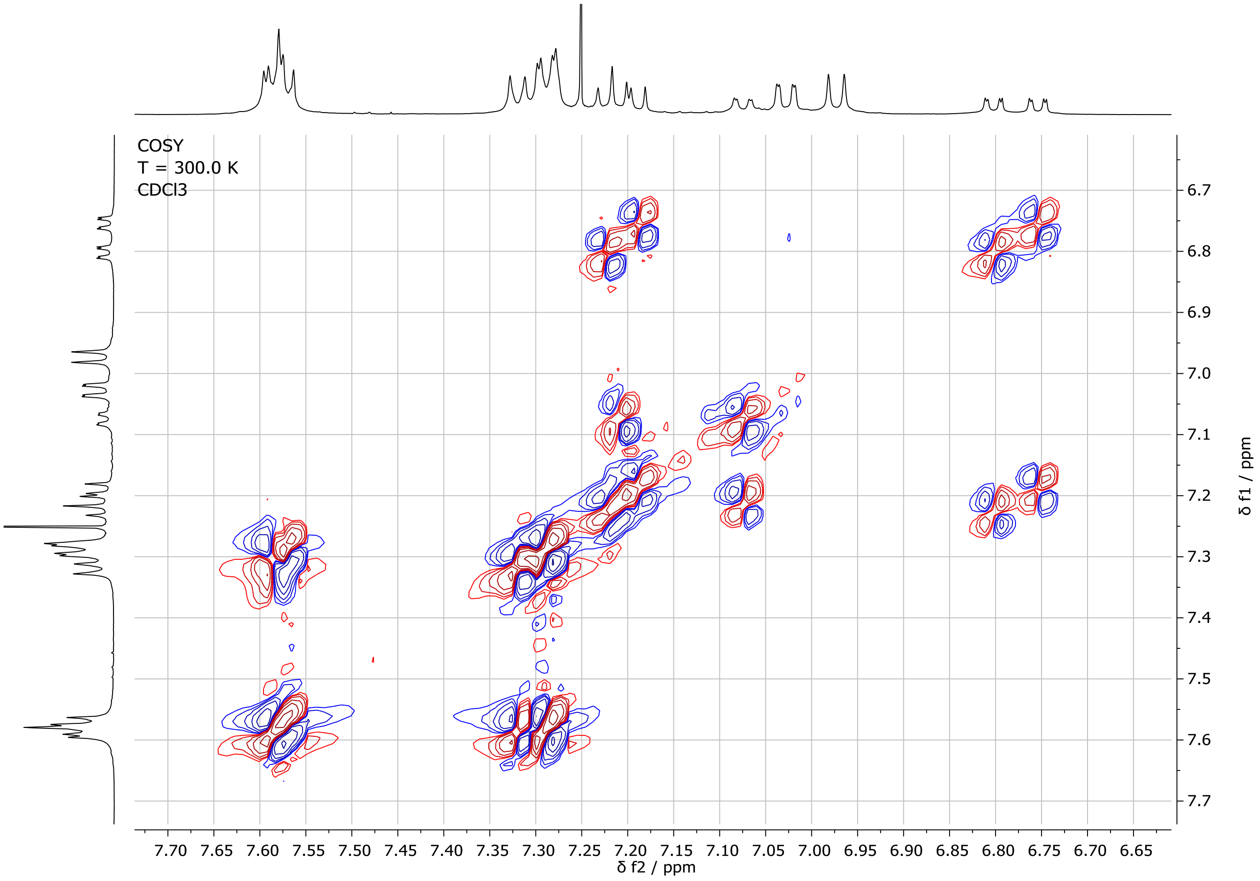

Figure S68. H,H-COSY spectra of **(+)-1** in $CDCl_3$ (500 MHz).

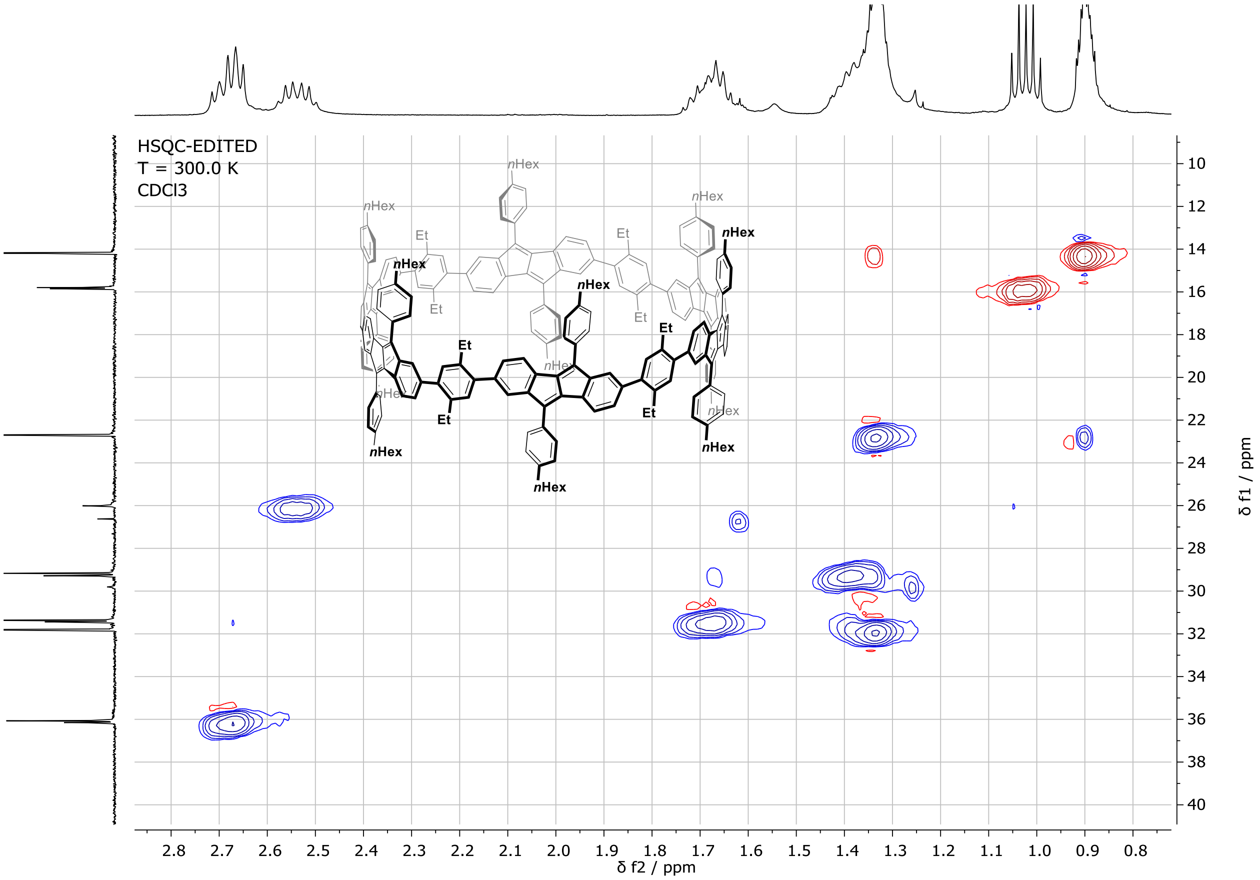

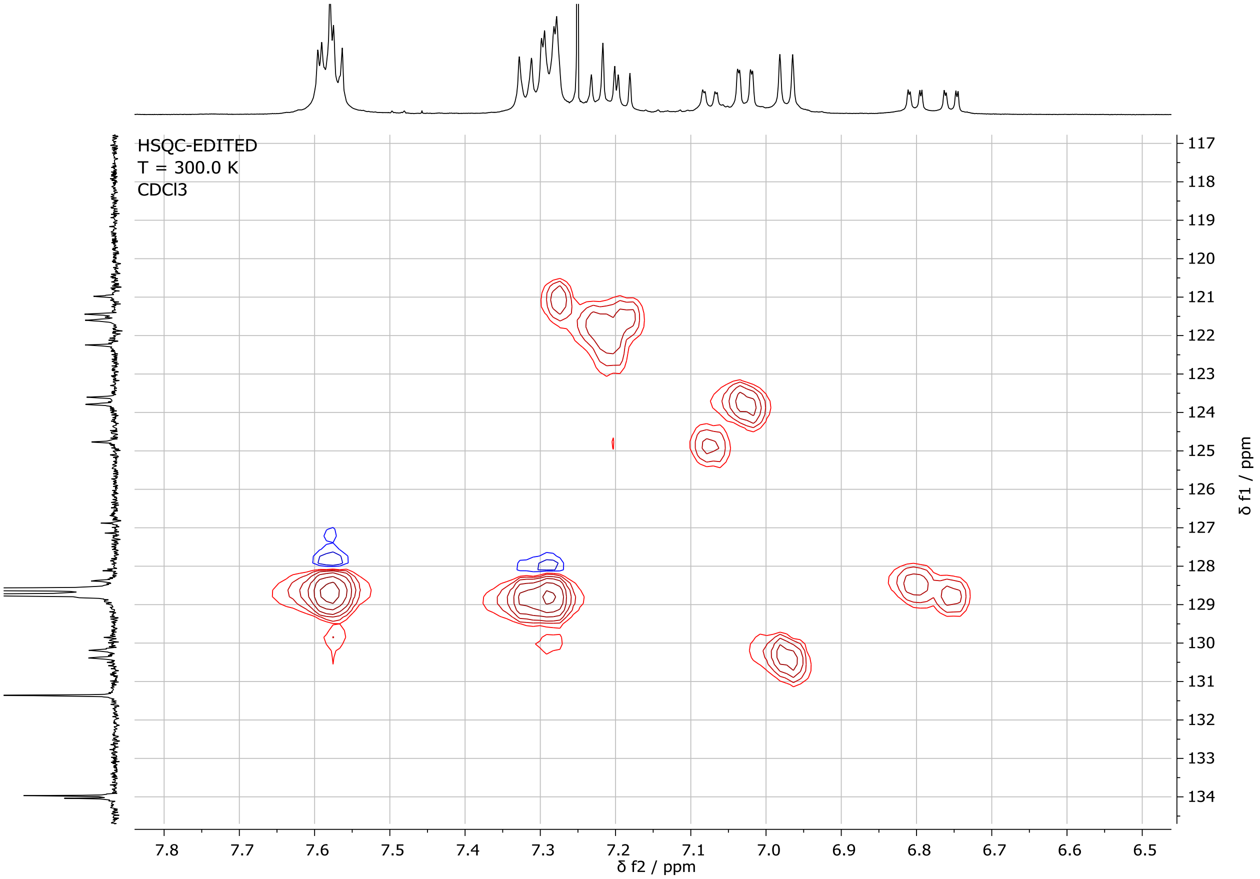

Figure S69. Edited HSQC spectra of **(+)-1** in $CDCl_3$ (500/126 MHz). Blue: CH_2 groups, red: CH or CH_3 groups.

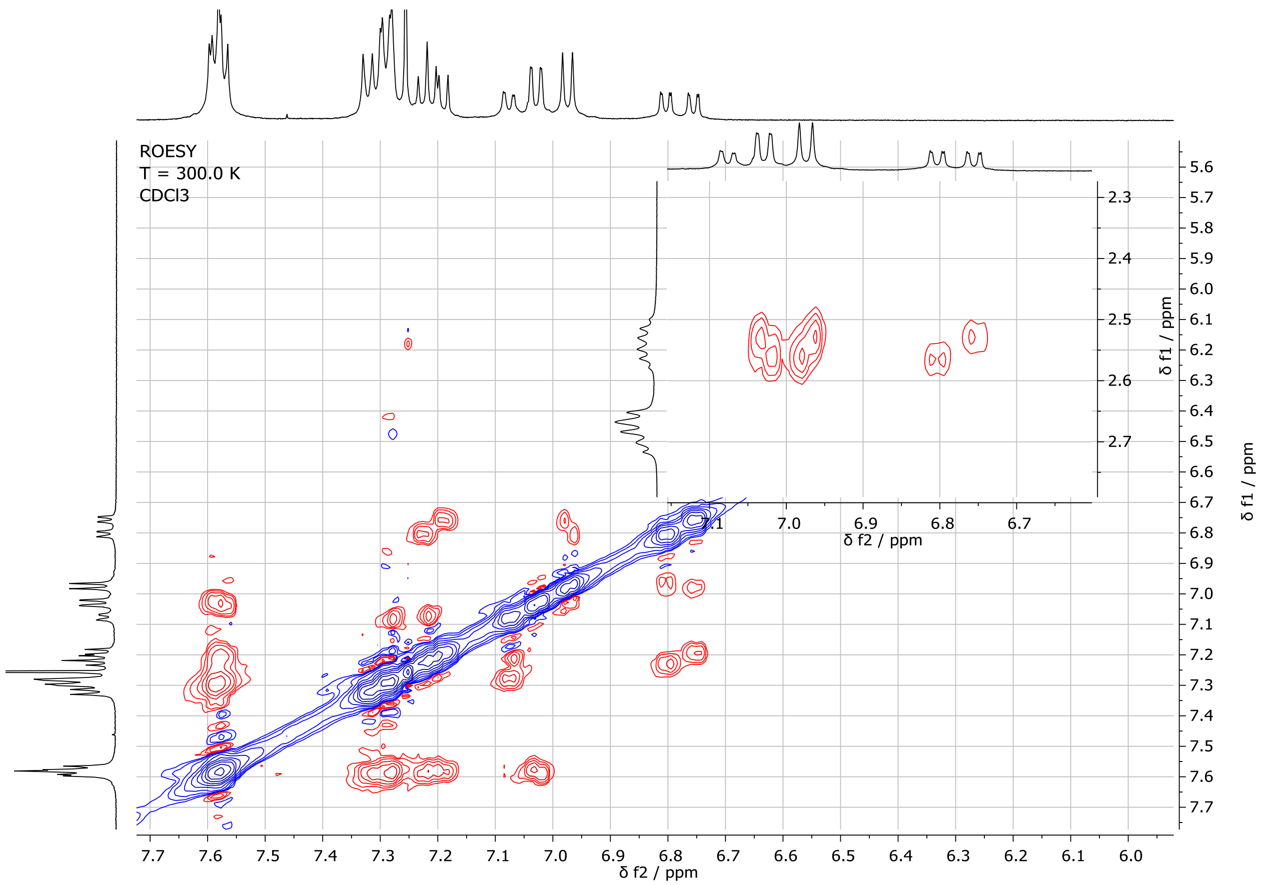

Figure S70. ROE spectrum of **(+)-1** in $CDCl_3$ (500 MHz).

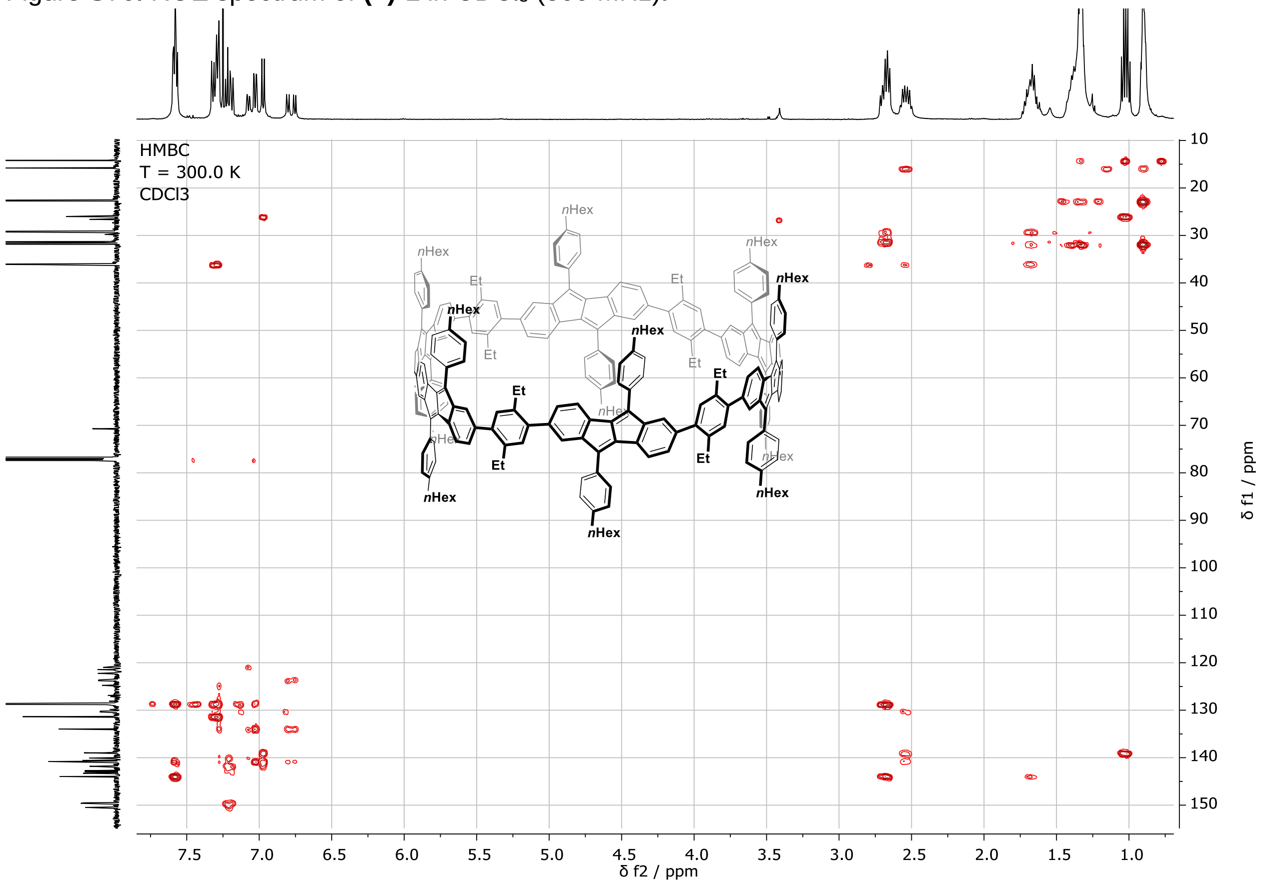

Figure S71. HMBC spectra of **(+)-1** in $CDCl_3$ (500/126 MHz

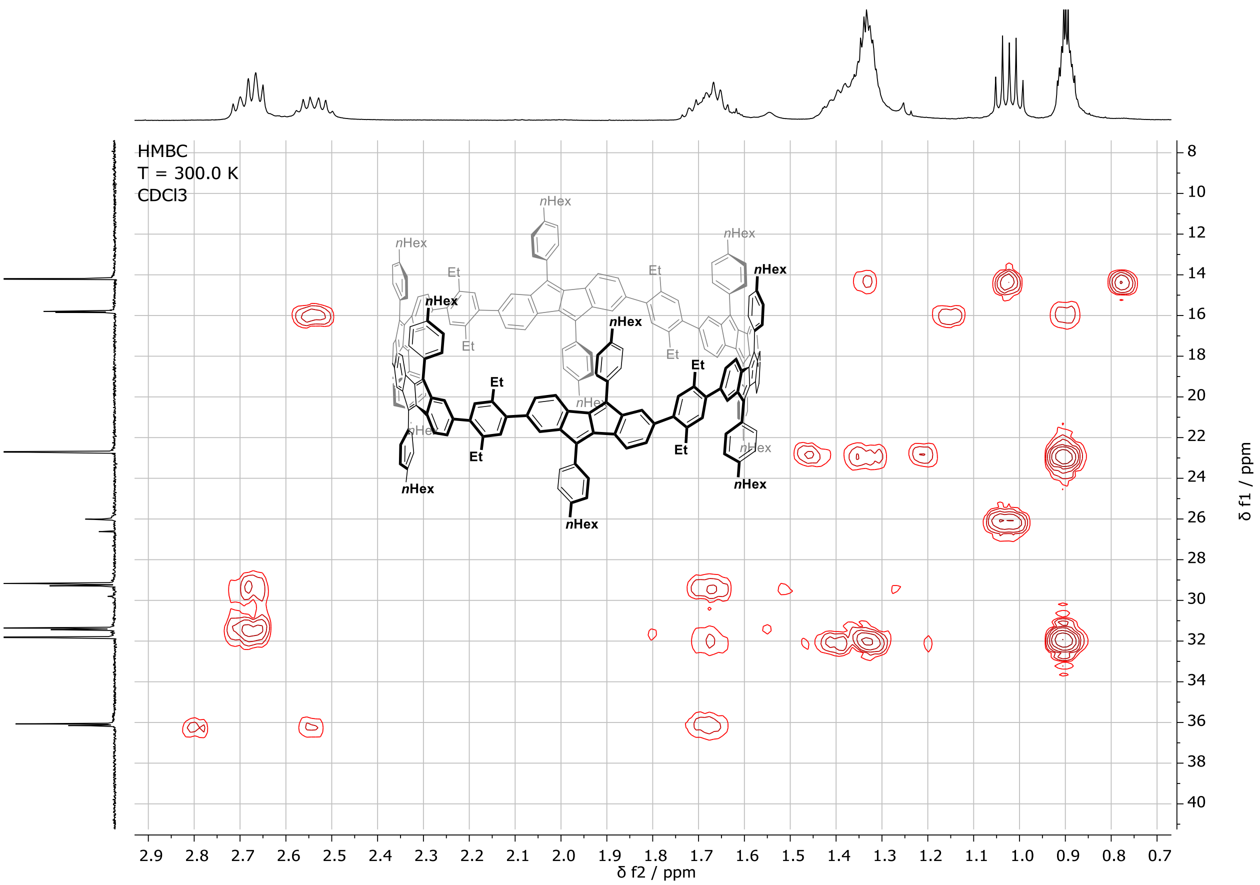

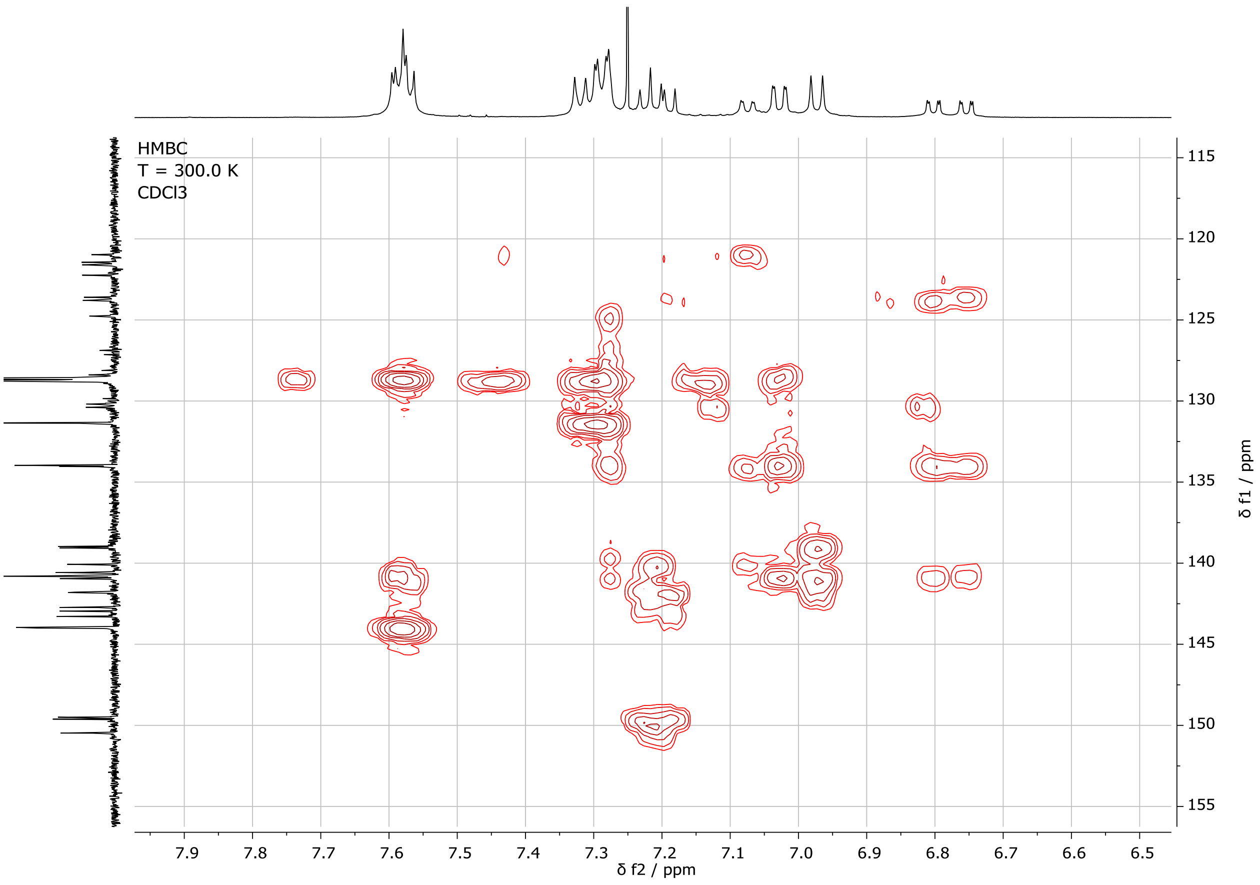

Figure S72. HMBC spectra of **(+)-1** in $CDCl_3$ (500/126 MHz

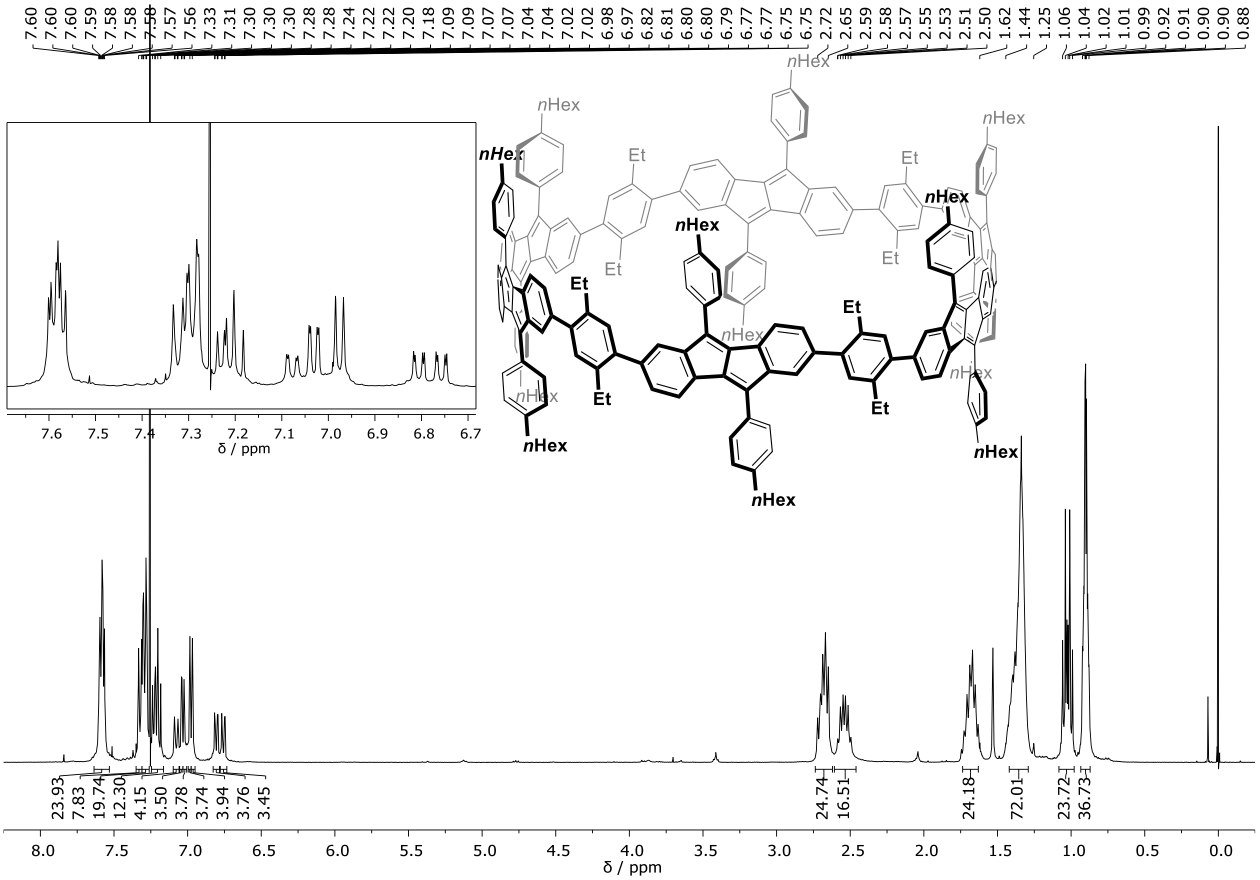

Figure S73. ^{1}H NMR spectrum of **(−)-1** in $CDCl_3$ (400 MHz).

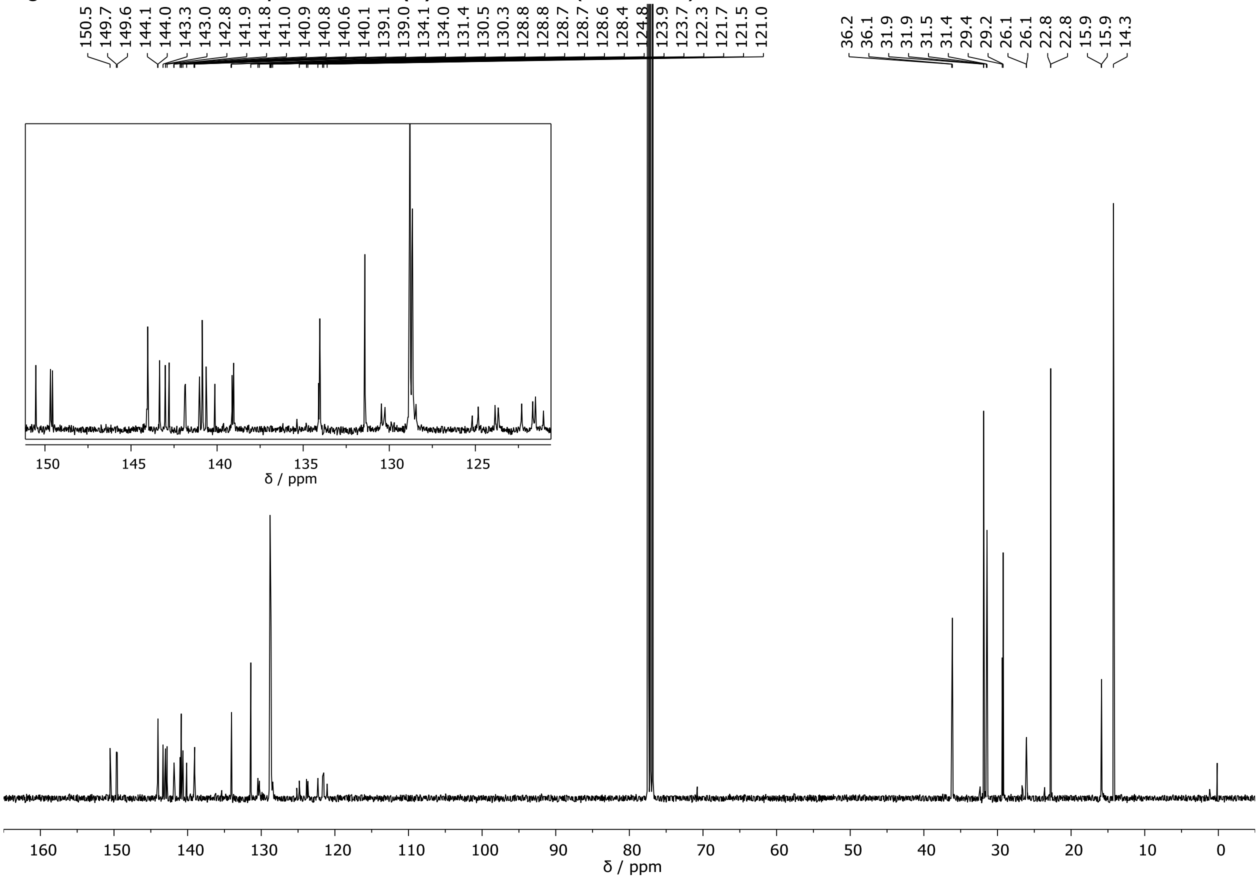

Figure S74. ^{13}C NMR spectrum of **(−)-1** in $CDCl_3$ (101 MHz).

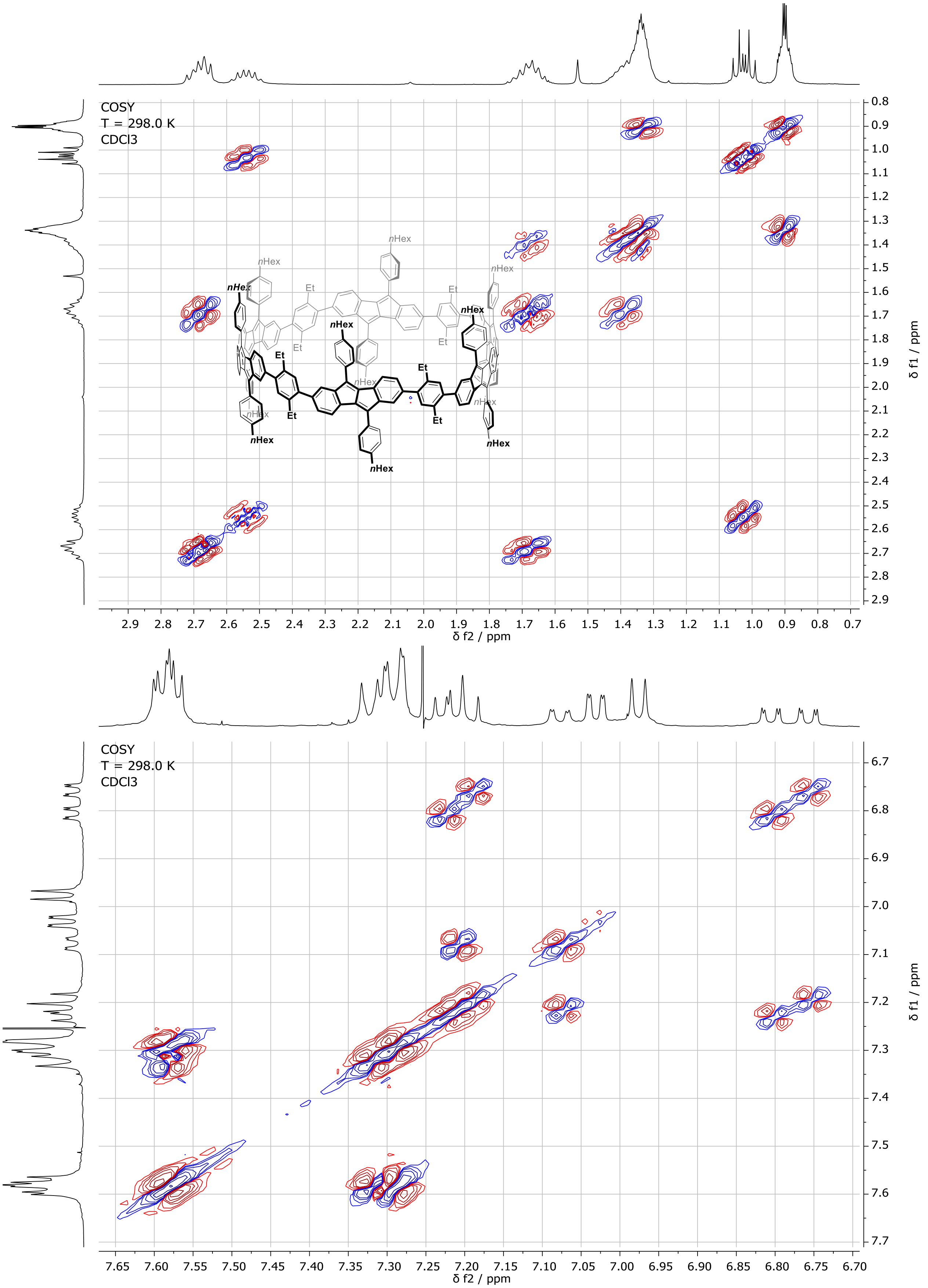

Figure S75. H,H-COSY spectra of **(−)-1** in $CDCl_3$ (400 MHz).

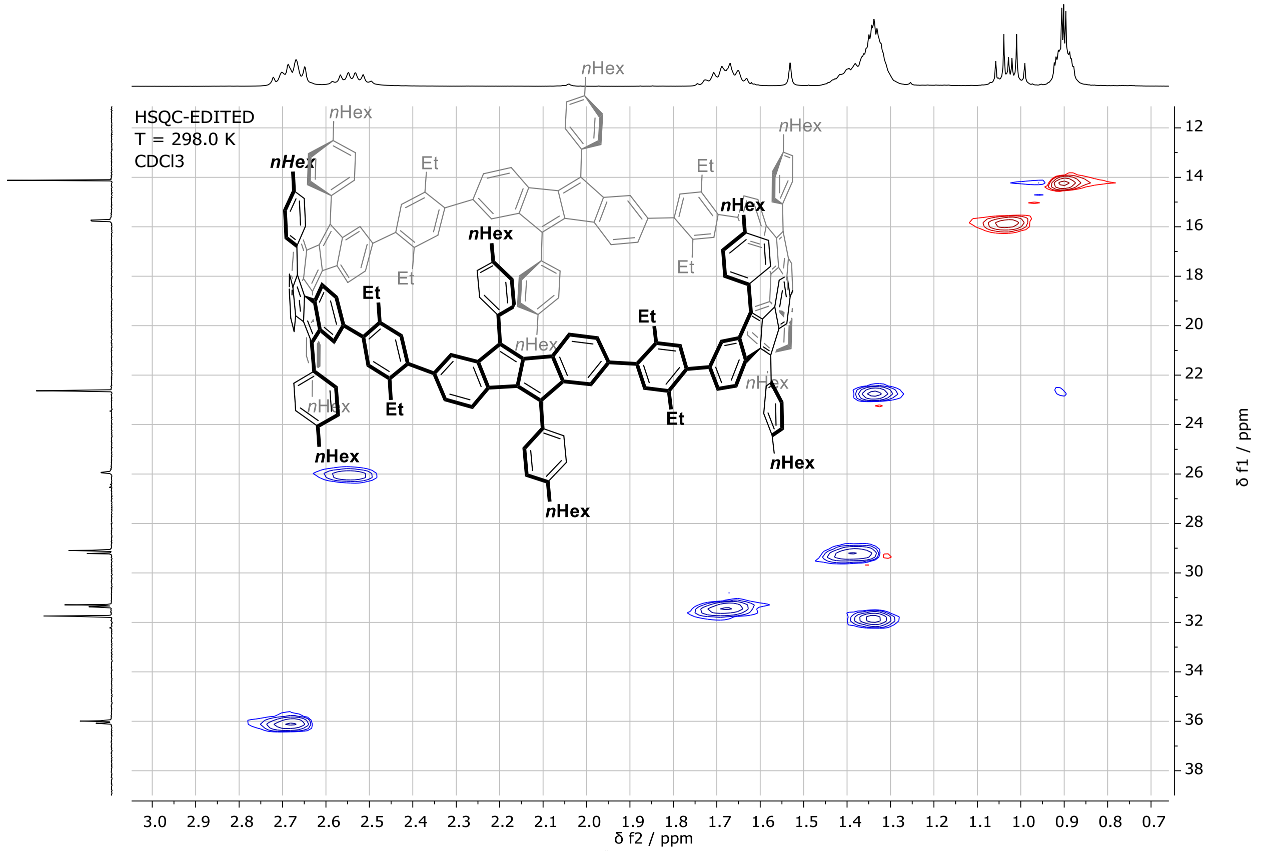

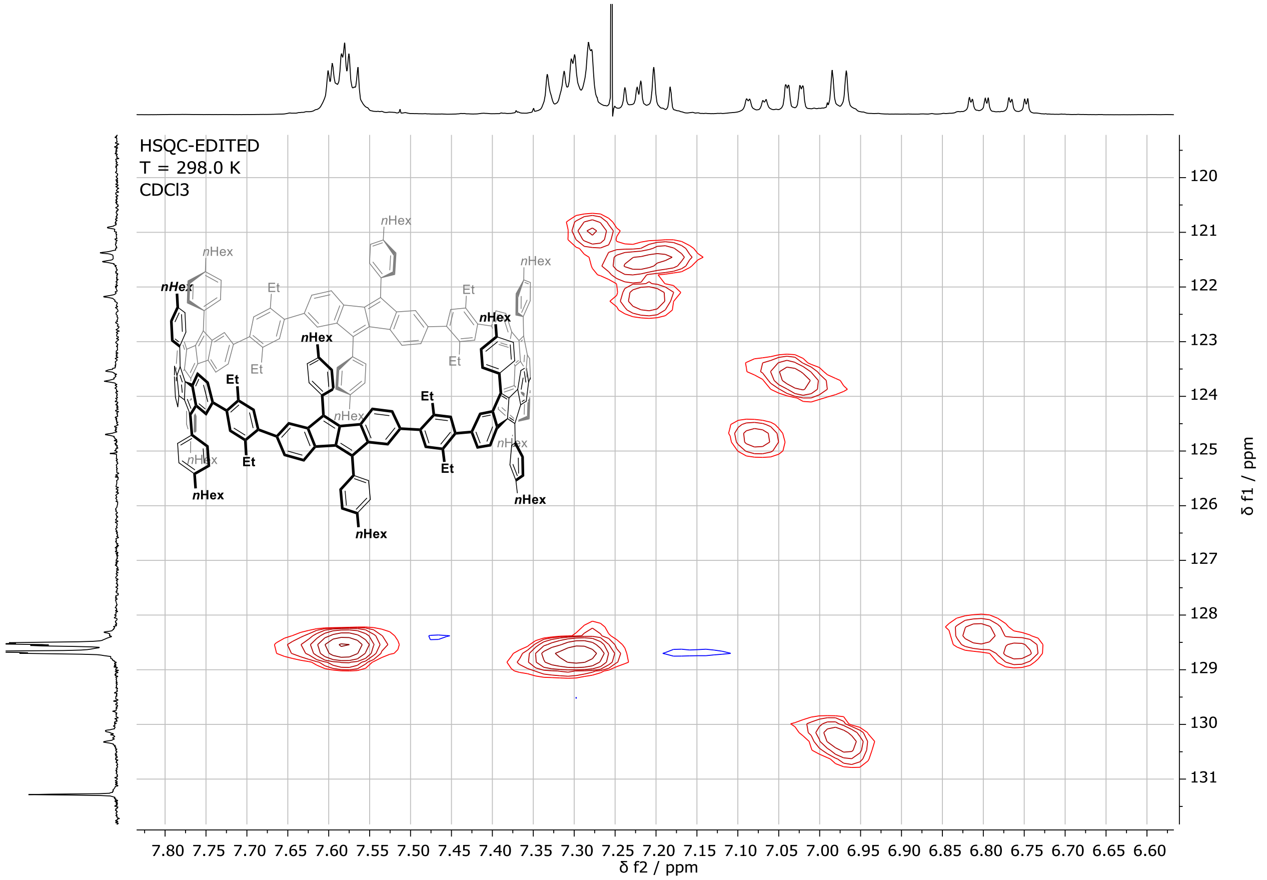

Figure S76. Edited HSQC spectra of **(−)-1** in $CDCl_3$ (400/101 MHz). Blue: CH_2 groups, red: CH or CH_3 groups.

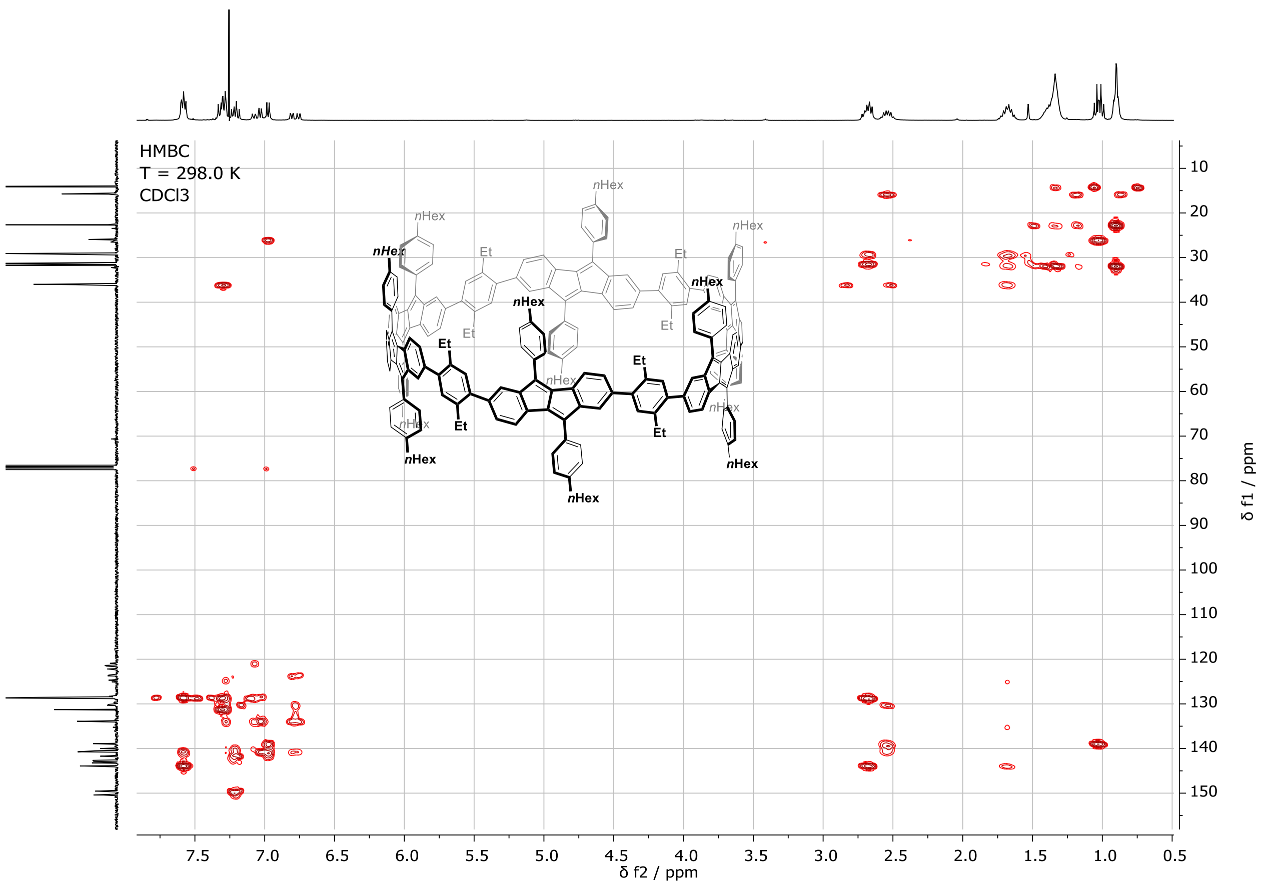

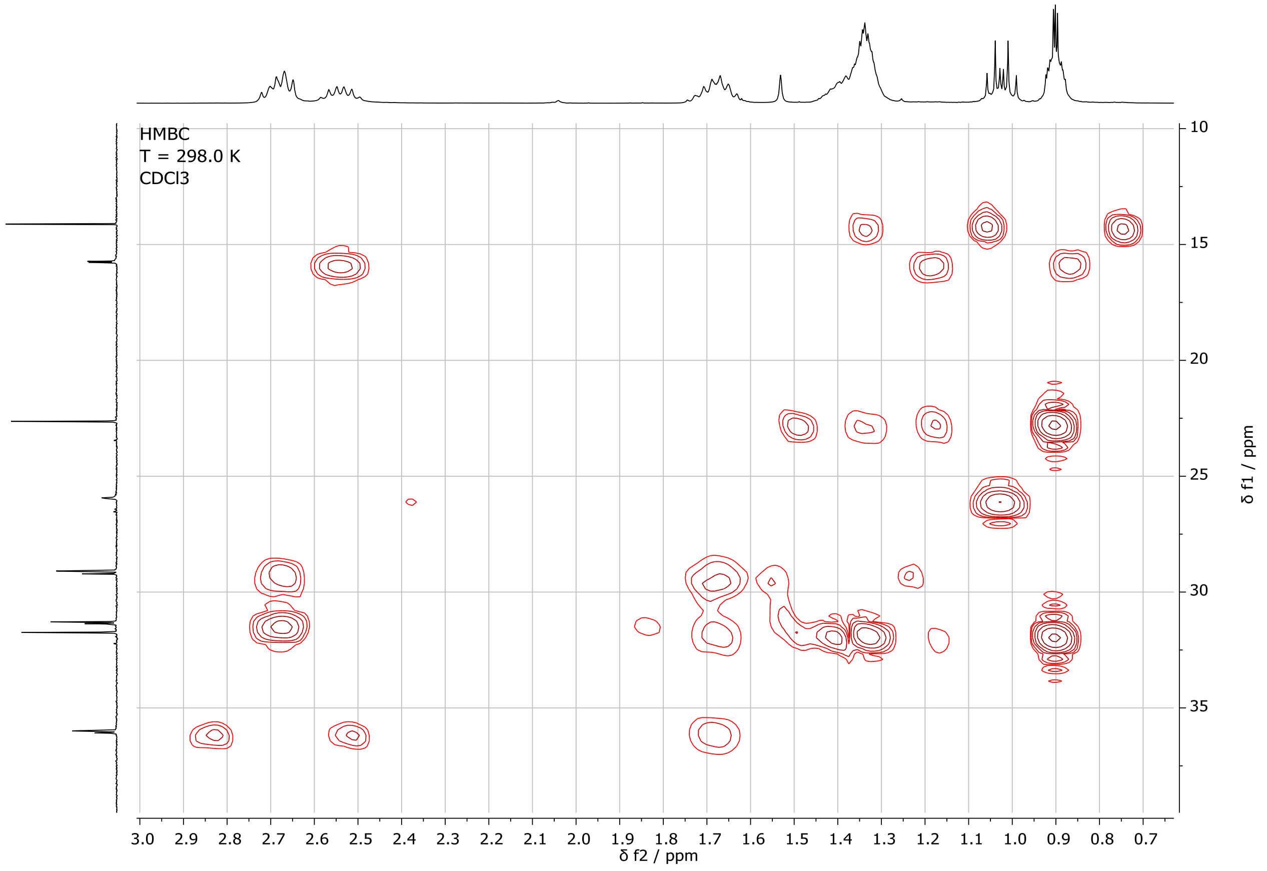

Figure S77. HMBC spectra of **(−)-1** in $CDCl_3$ (400/101 MHz

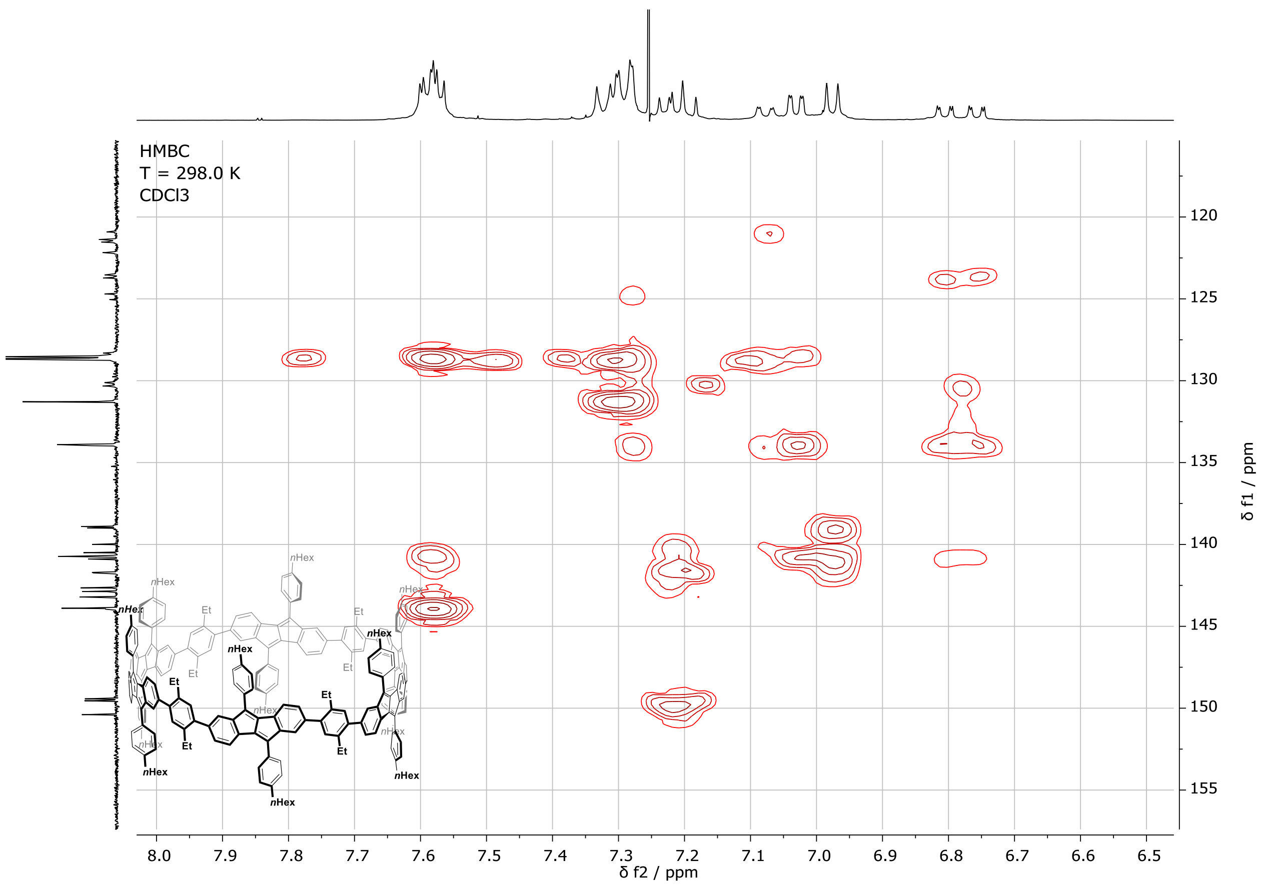

Figure S78. HMBC spectra of **(−)-1** in $CDCl_3$ (400/101 MHz

3.2 VT NMR Spectra of (+)-1

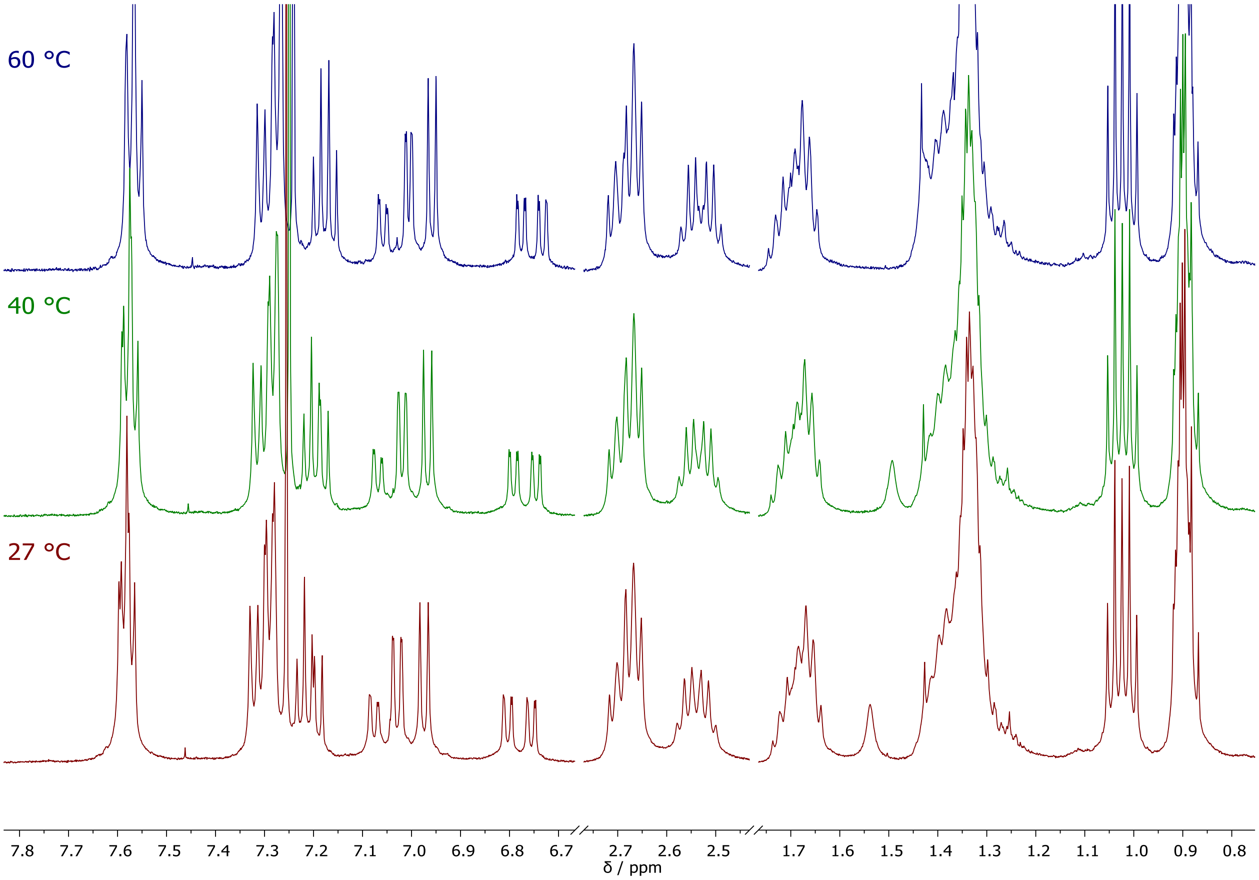

Figure S79. ^{1}H VT NMR spectra of **(+)-1** in $CDCl_3$ (500 MHz) at 27, 40 and 60 °C.

In another experiment **(−)-1** was heated to 120 °C in a chlorobenzene solution (c =5.74·10^{-4} M) for 2 days. The solvent was removed under reduced pressure at 60 °C and the solid was dried at 100 °C / 1·10^{-3} mbar for 16 h. Another NMR spectrum was recorded and the resulting spectrum is identical with the one before the heat treatment.

3.3 NMR Assignment of (+)-1

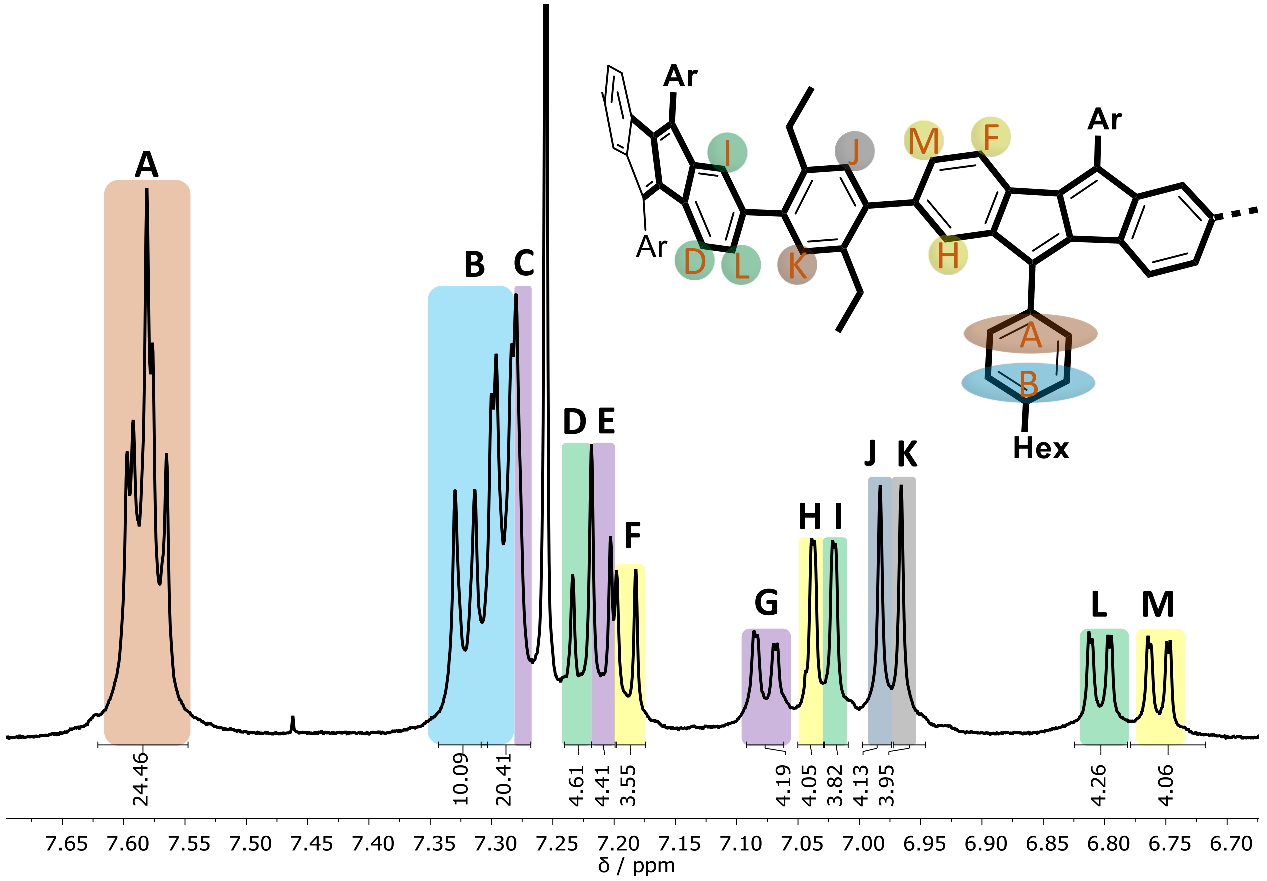

Figure S80. NMR assignment of aromatic protons of **(+)-1** (based on spectra in Figure S81 and Figure S82).

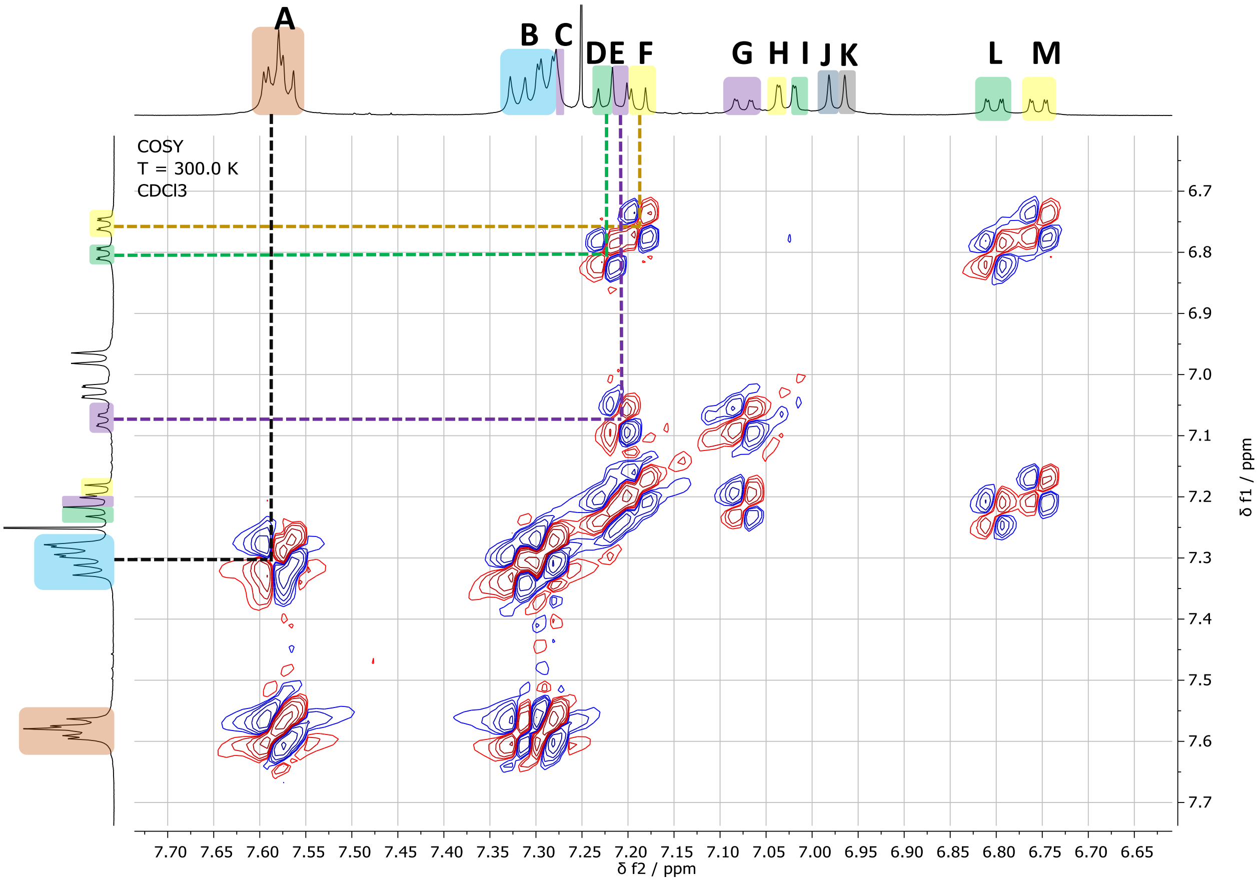

Figure S81. NMR assignment of **(+)-1** with COSY ($CDCl_3$, 500 MHz).

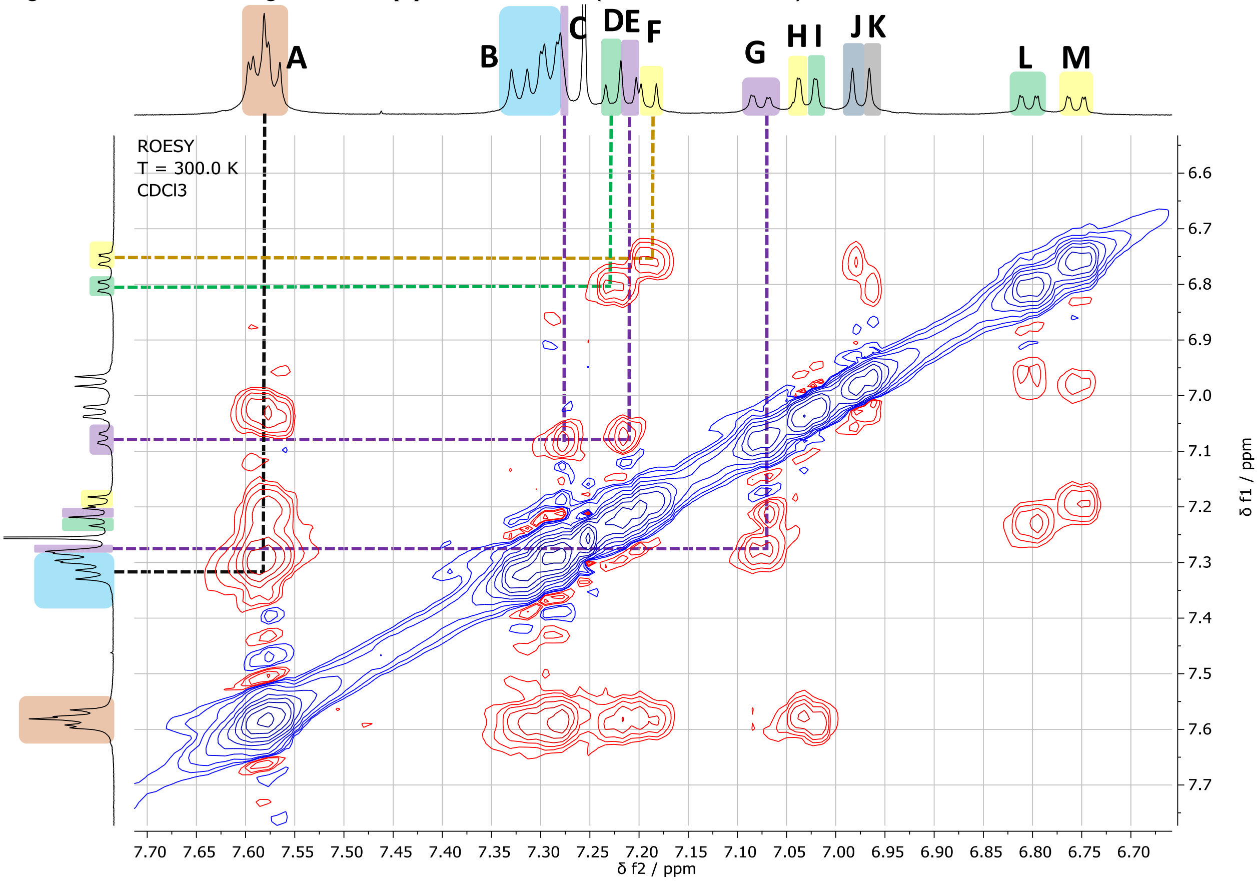

Figure S82. NMR assignment of **(+)-1** with ROESY ($CDCl_3$, 500 MHz).

4. ATR FT-IR Spectra

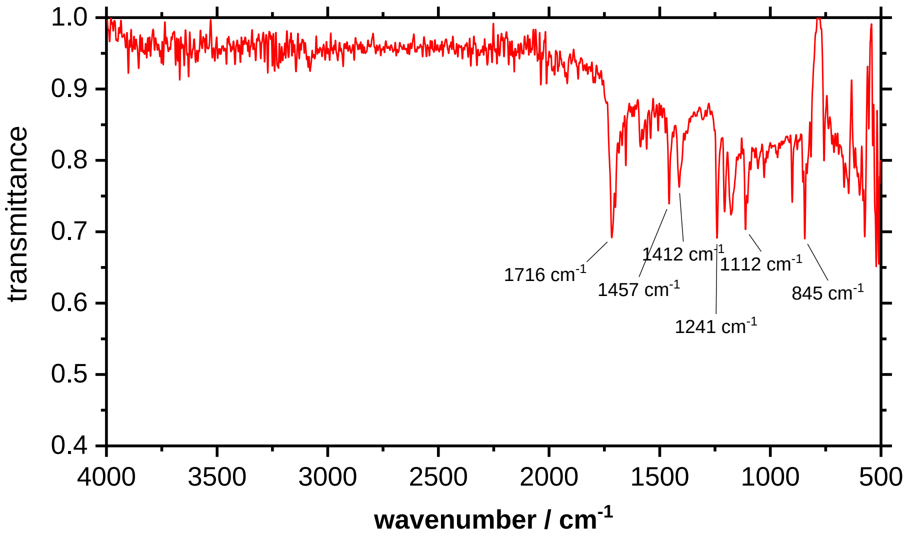

Figure S83. ATR FT-IR spectrum of **3**.

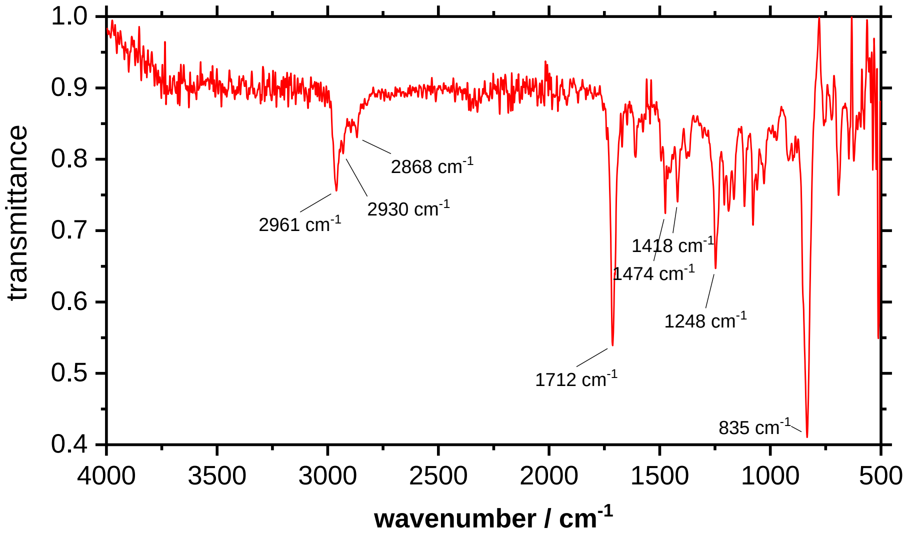

Figure S84. ATR FT-IR spectrum of **(*R*,*R*)-9**.

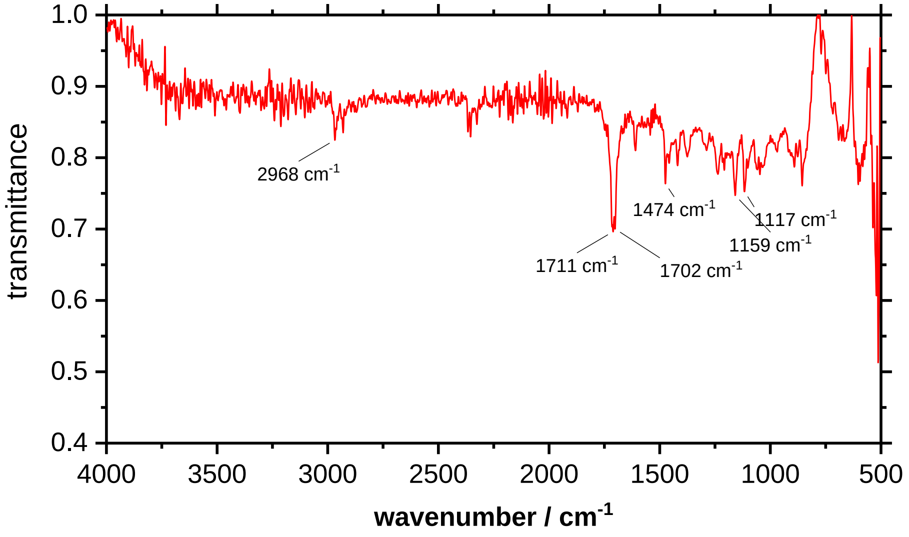

Figure S85. ATR FT-IR spectrum of **(*R*,*R*)-10**.

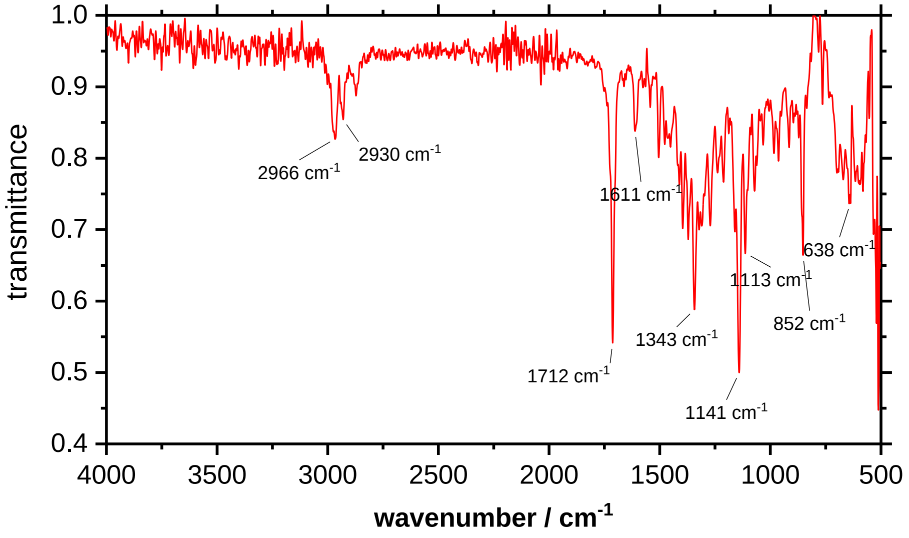

Figure S86. ATR FT-IR spectrum of **(*R*,*R*)-11**.

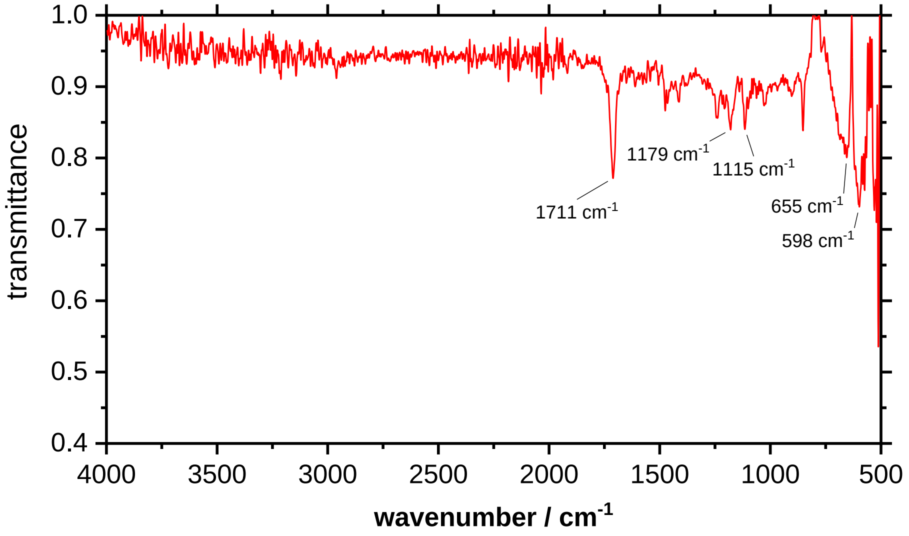

Figure S87. ATR FT-IR spectrum of **18**.

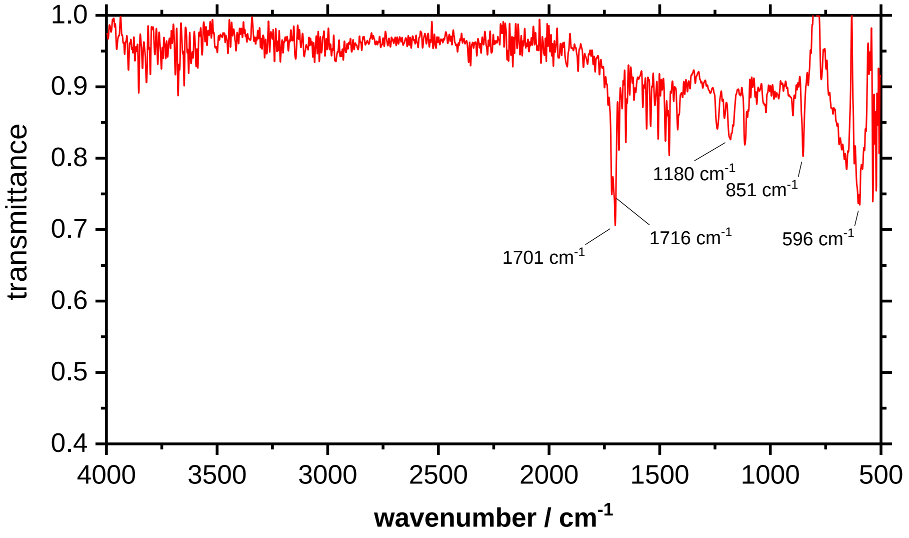

Figure S88. ATR FT-IR spectrum of **(*R*,*R*)³-12**.

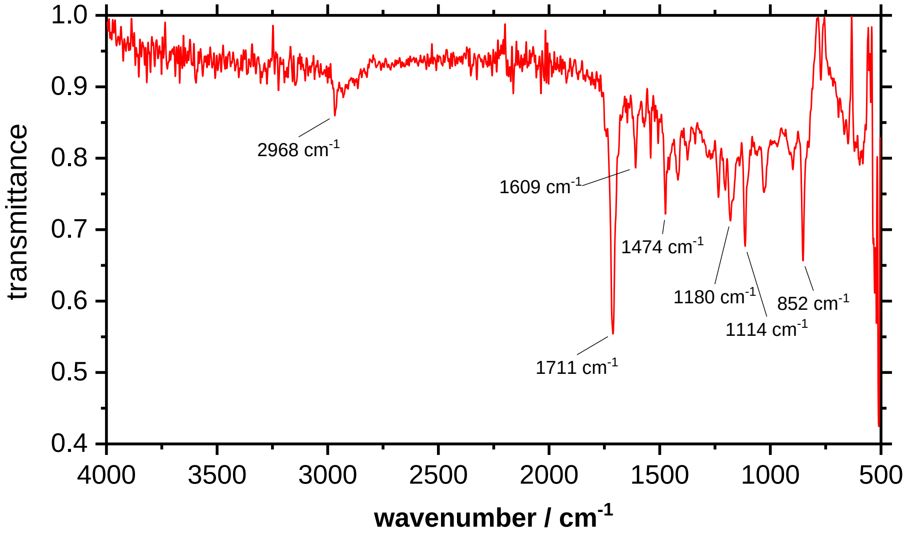

Figure S89. ATR FT-IR spectrum of **19**.

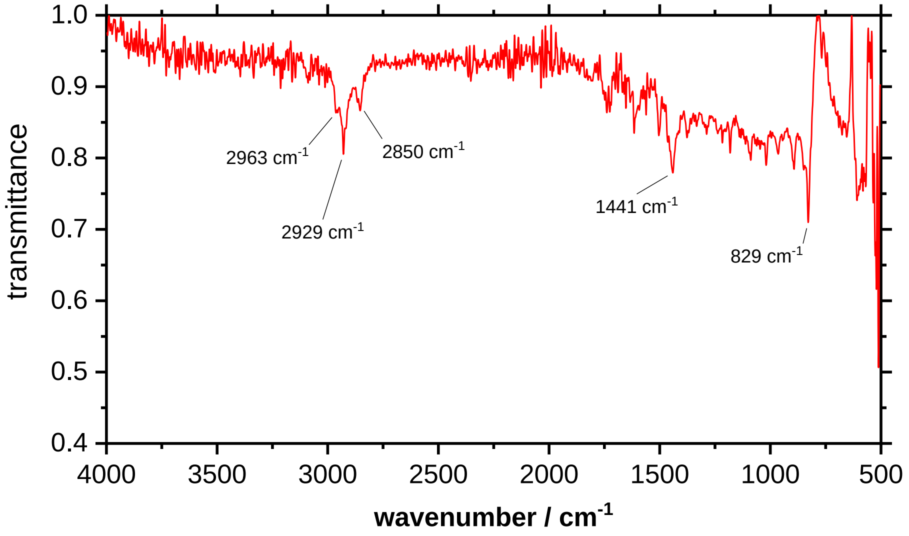

Figure S90. ATR FT-IR spectrum of **7**.

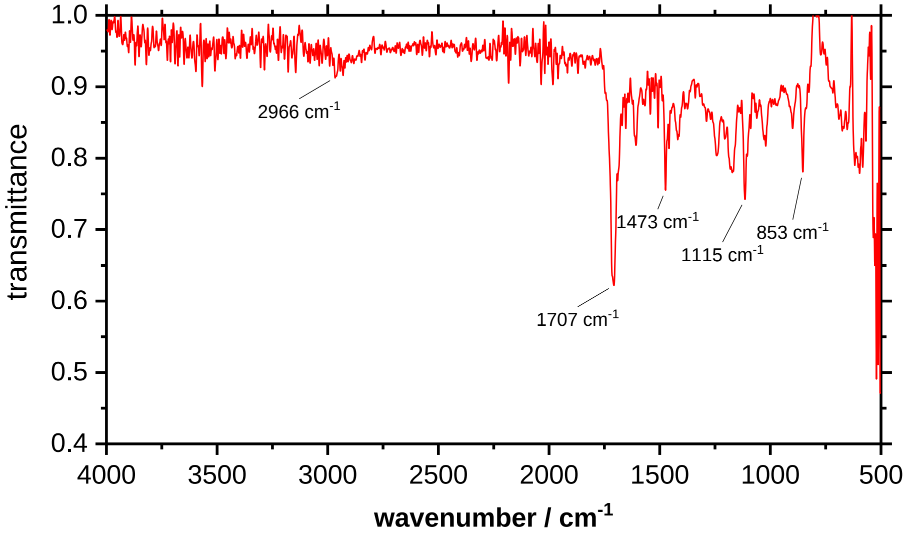

Figure S91. ATR FT-IR spectrum of **(*R*,*R*)^{6}-14**.

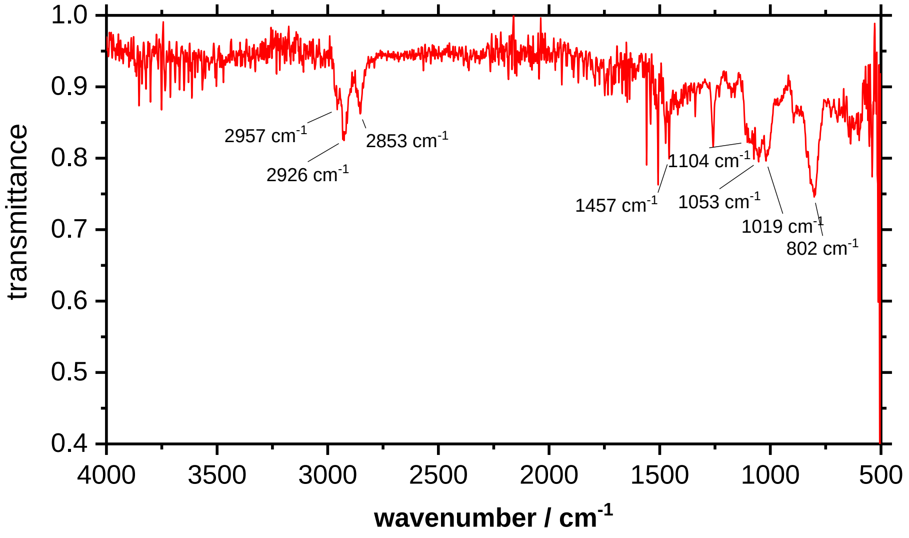

Figure S92. ATR FT-IR spectrum of **(*R*,*R*)^6-(+)-16**.

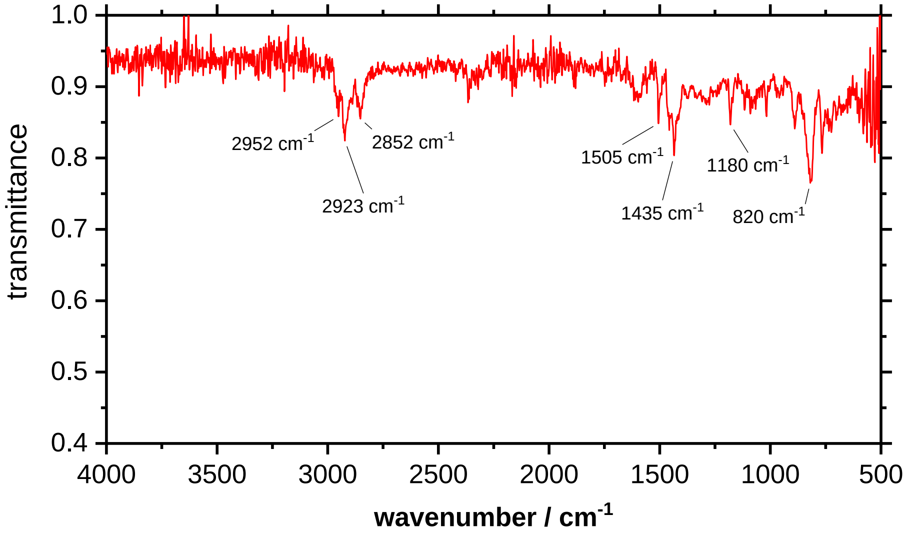

Figure S93. ATR FT-IR spectrum of **(+)-1**.

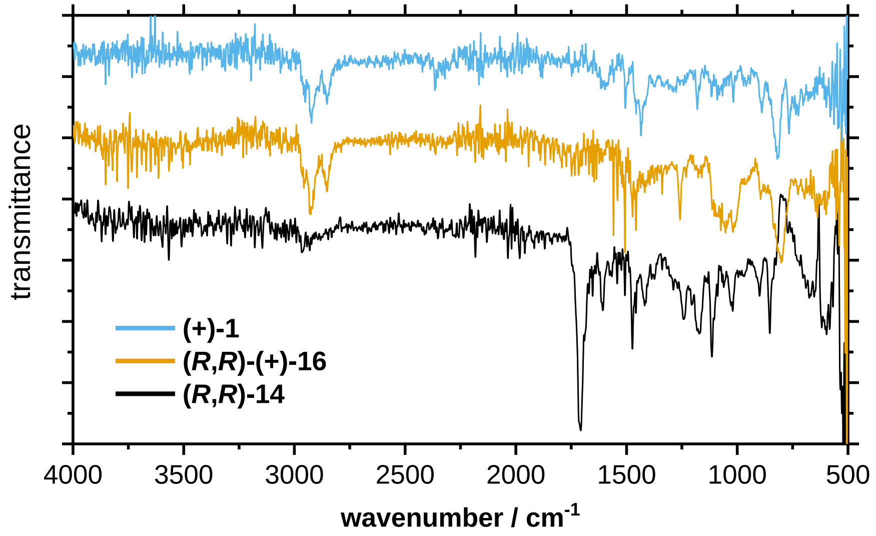

Figure 94. Comparison of ATR FT-IR spectra of **(+)-1**, **(*R*,*R*)^6-(+)-16** and **(*R*,*R*)^6-14**.

6. GPC Elugrams

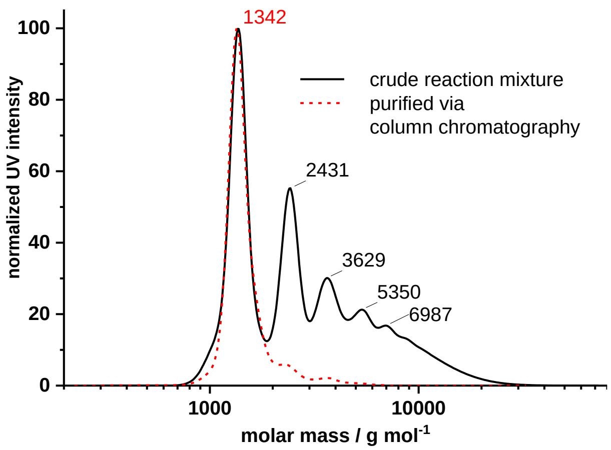

Figure S95. Analytical GPC elugram of the crude reaction mixture in the synthesis of **(*R*,*R*)⁶-14** and of purified **(*R*,*R*)⁶-14**. No signal peaks with lower molar mass than **(*R*,*R*)⁶-14** are visible. Eluent: THF, vs. polystyrene standard.

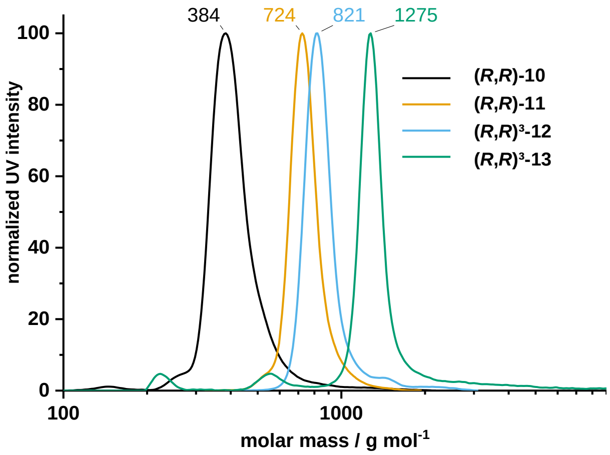

Figure S96. Analytical GPC elugrams of **(*R*,*R*)-10, (*R*,*R*)-11, (*R*,*R*)³-12 and (*R*,*R*)³-13**. Eluent: THF, vs. polystyrene standard.

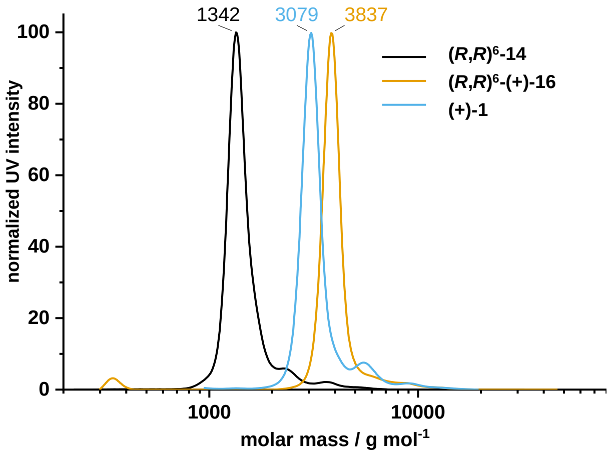

Figure S97. Analytical GPC elugrams of **(*R*,*R*)⁶-14, (*R*,*R*)⁶-(+)-16 and (+)-1**. Eluent: THF, vs. polystyrene standard.

7. HPLC Elugrams

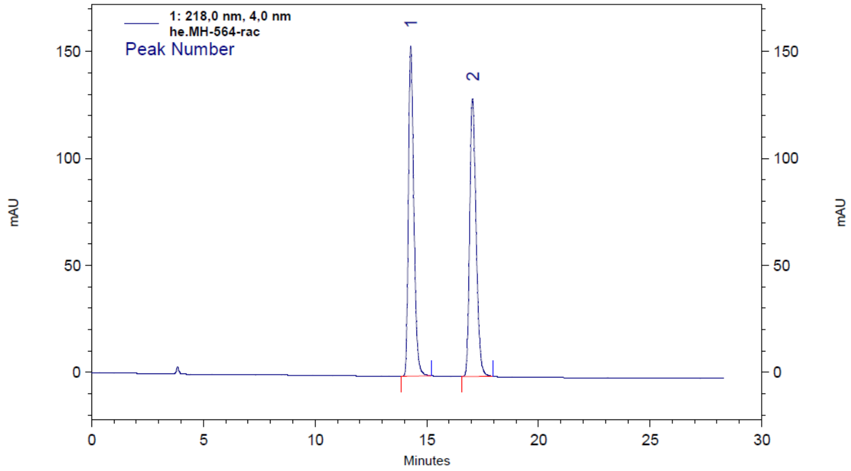

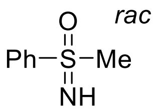

1: 218,0 nm, 4,0 nm

Results

Peak Number	Retention Time	Area Percent	Area
1	14,280	49,967	348147872
2	17,045	50,033	348609180
Totals		100,000	696757052

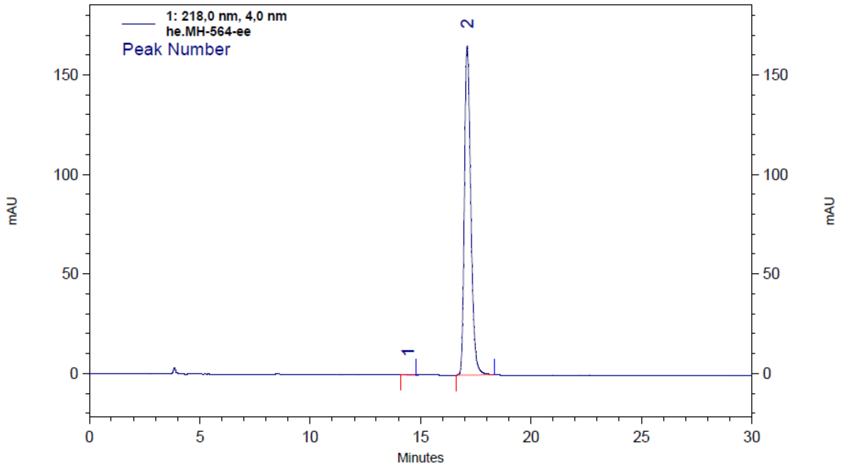

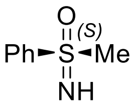

1: 218,0 nm, 4,0 nm

Results

Peak Number	Retention Time	Area Percent	Area
1	14,420	0,088	394956
2	17,113	99,912	447471462
Totals		100,000	447866418

Figure S98. HPLC elugrams of ***rac*-S2** and **(*S*)-S2** (Lux Cellulose LC-3, λ = 218 nm, *n*-heptane/*i*-PrOH = 85/15, 0.5 mL min^{-1}, 22 °C): t_R = 13.9 min (minor), 17.1 min (major), > 99% *ee.*

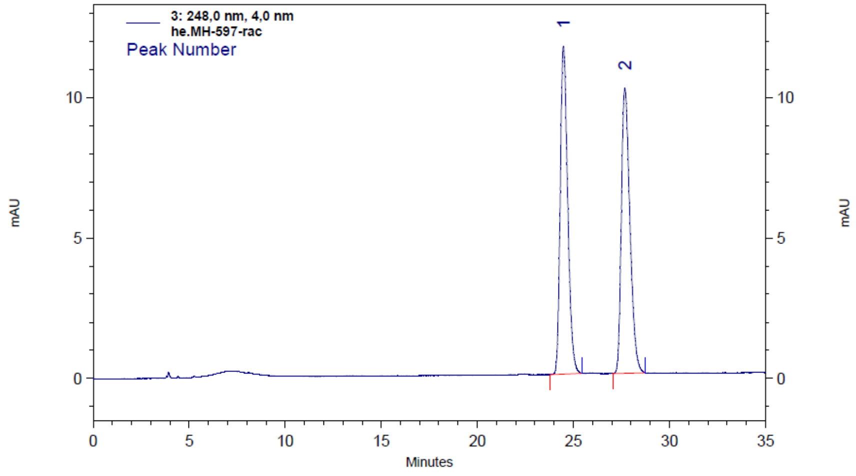

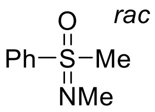

3: 248,0 nm, 4,0 nm
Results

Peak Number	Retention Time	Area Percent	Area
1	24,485	50,095	42387460
2	27,678	49,905	42227058
Totals		100,000	84614518

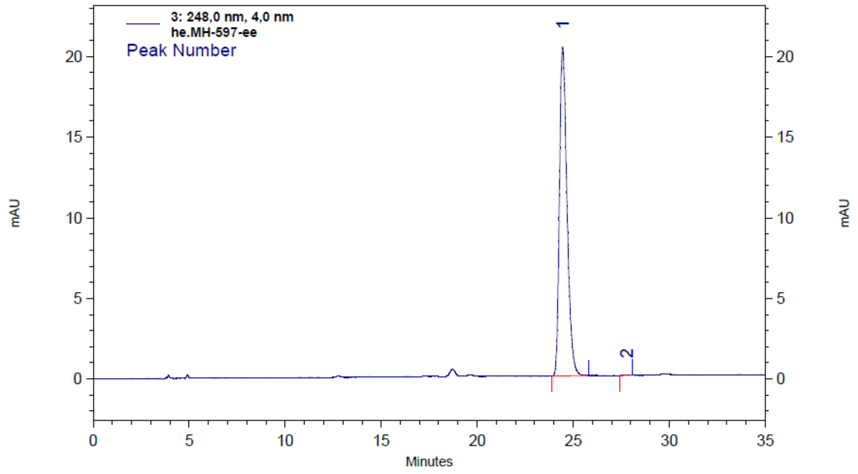

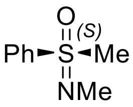

3: 248,0 nm, 4,0 nm
Results

Peak Number	Retention Time	Area Percent	Area
1	24,450	99,936	75003733
2	27,773	0,064	47737
Totals		100,000	75051470

Figure S99. HPLC elugrams of ***rac*-4** and **(*S*)-4** (Chiralpak AD-3, λ = 248 nm, *n*-heptane/*i*-PrOH = 95/5, 0.5 mL min^{-1}, 22 °C): t_R = 24.5 min (major), 27.8 min (minor), > 99% *ee.*

Sample ID: he.MH-679-rac

Method: Chiralpak-IA, nHept/DCM: 75/25, 1ml/min, 22°C

Vial: 191 Injection Volume (µl): 5
Run Time: 10.09.2019 11:35:08 Analysis Time: 10.09.2019 15:18:17

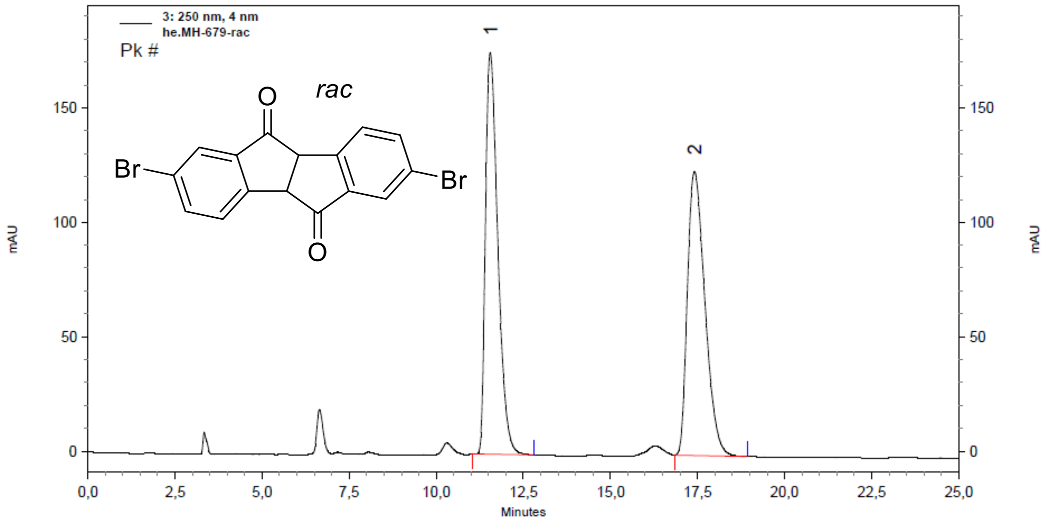

3: 250 nm, 4 nm Results

Pk #	Retention Time	Area Percent	Lambda Max
1	11,553	49,958	241
2	17,413	50,042	241

Method: Chiralpak-IA, nHept/DCM: 75/25, 1ml/min, 22°C

Vial: 194 Injection Volume (µl): 5
Run Time: 10.09.2019 12:55:22 Analysis Time: 10.09.2019 15:28:19

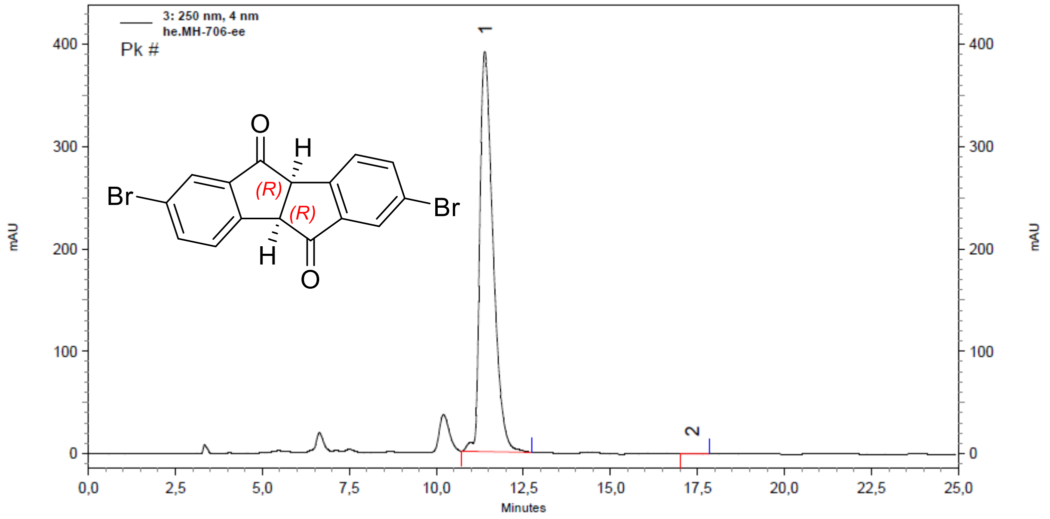

3: 250 nm, 4 nm Results

Pk #	Retention Time	Area Percent	Lambda Max
1	11,393	99,907	241
2	17,340	0,093	208

Figure S100. HPLC elugrams of ***rac*-3** and **(*R*,*R*)-3** (Chiralpak IA, λ = 250 nm, *n*-heptane/CH_2Cl_2 = 75/25, 1.0 mL min^{-1}, 22 °C): t_R = 11.4 min ((*R*)-enantiomer, > 99% *ee.*), 17.3 min ((*S*)-enantiomer).

```
Sample ID:  he.MH-707-ee

Method:     Chiralpak-IA, nHept/DCM: 75/25, 1ml/min, 22°C

Vial:  197      Injection Volume (µl):   5
Run Time:  10.09.2019 14:16:32     Analysis Time:  10.09.2019 15:39:59
```

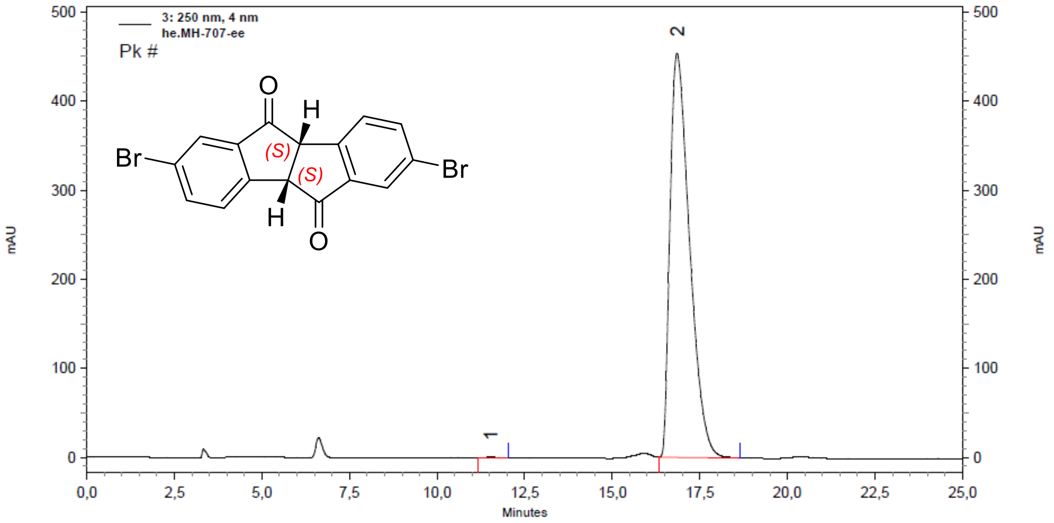

3: 250 nm, 4 nm Results

Pk #	Retention Time	Area Percent	Lambda Max
1	11,520	0,139	239
2	16,853	99,861	242

Figure S101 HPLC elugram of **(*S*,*S*)-3** (Chiralpak IA, *λ* = 250 nm, *n*-heptane/CH_2Cl_2 = 75/25, 1.0 mL min^{-1}, 22 °C): t_R = 11.5 min ((*R*)-enantiomer), 16.9 min ((*S*)-enantiomer, > 99% *ee.*).

Method: IA-CHIRALPAK, nHept_ChCl3, 90_10, 1ml, 22°C, HERMANN.met

Vial: 191 Injection Volume (µl): 2
Run Time: 12.02.2019 12:00:29 Analysis Time: 12.02.2019 13:32:26

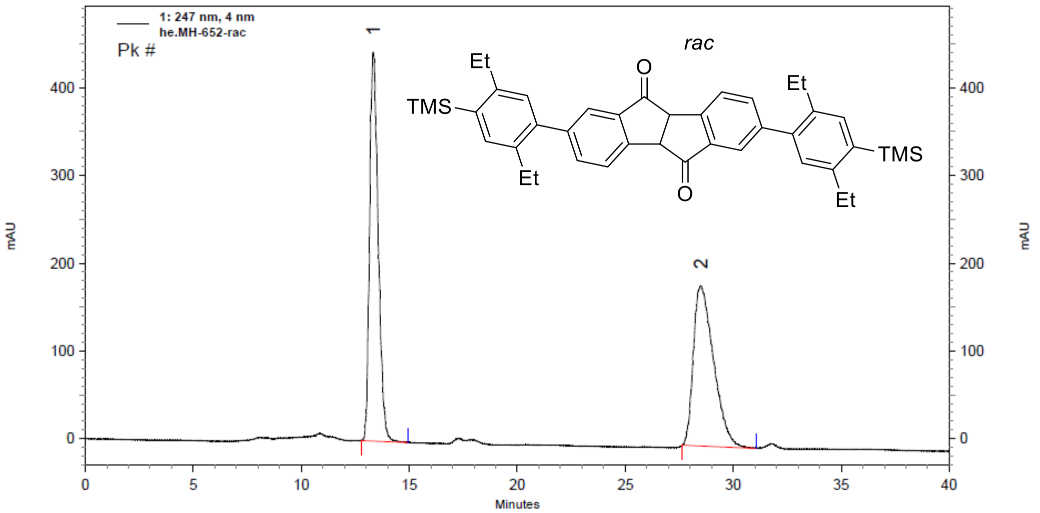

1: 247 nm, 4 nm Results

Pk #	Retention Time	Area Percent	Lambda Max
1	13,320	50,794	248
2	28,493	49,206	248

Method: IA-CHIRALPAK, nHept_ChCl3, 90_10, 1ml, 22°C, HERMANN.met

Vial: 192 Injection Volume (µl): 2
Run Time: 12.02.2019 12:42:04 Analysis Time: 12.02.2019 13:37:23

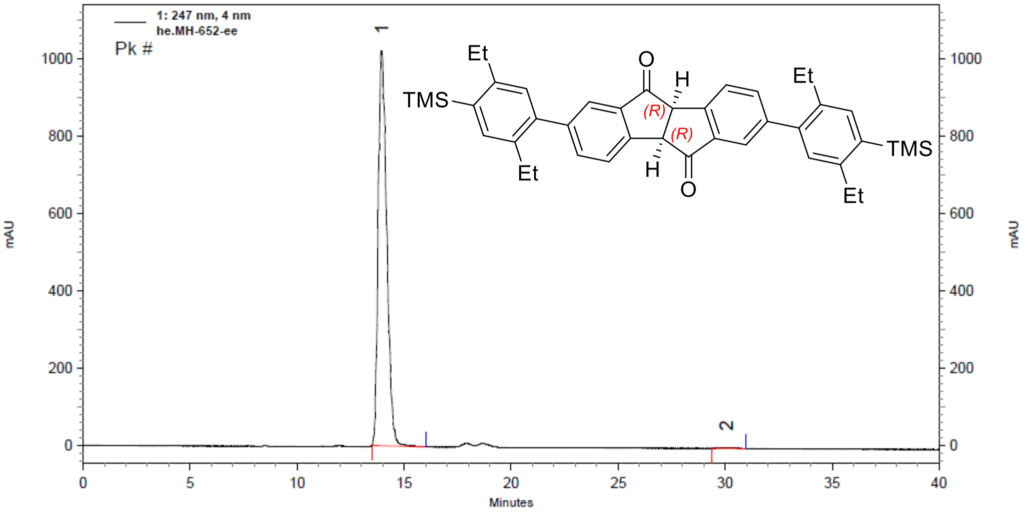

1: 247 nm, 4 nm Results

Pk #	Retention Time	Area Percent	Lambda Max
1	13,940	99,532	248
2	30,053	0,468	221

Figure S102. HPLC elugrams of ***rac*-9** and **(*R*,*R*)-9** (Chiralpak IA, λ = 247 nm, *n*-heptane/$CHCl_3$ = 90/10, 1.0 mL min^{-1}, 22 °C): t_R = 13.9 min (major), 30.1 min (minor), 99 % *ee*.

Method: CHIRALPAK IA, nHeptan/DCM 75:25, 0,5ml/min, 22°C

Vial: 195 Injection Volume (µl): 5

Run Time: 11.11.2020 15:04:18 Analysis Time: 11.11.2020 15:30:27

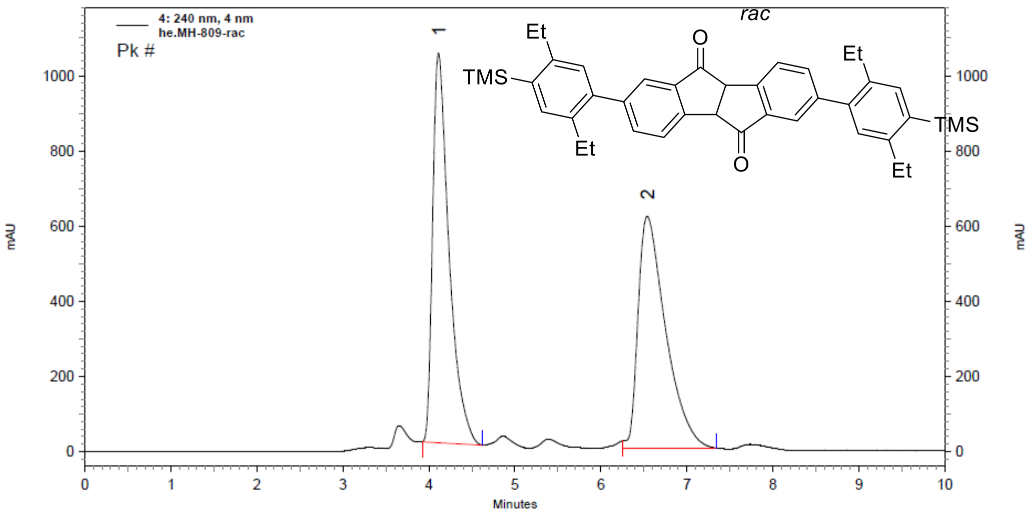

4: 240 nm, 4 nm Results

Pk #	Retention Time	Area Percent	Lambda Max
1	4,113	49,932	248
2	6,540	50,068	248

Method: CHIRALPAK IA, nHeptan/DCM 75:25, 0,5ml/min, 22°C

Vial: 181 Injection Volume (µl): 5

Run Time: 11.11.2020 15:15:41 Analysis Time: 11.11.2020 15:32:12

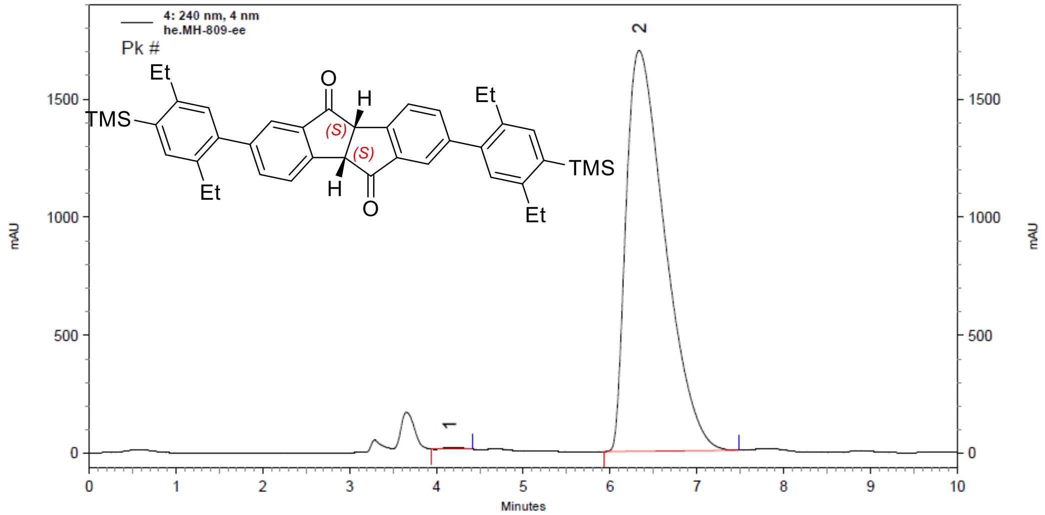

4: 240 nm, 4 nm Results

Pk #	Retention Time	Area Percent	Lambda Max
1	4,147	0,156	227
2	6,333	99,844	247

Figure S103. HPLC elugrams of ***rac*-9** and **(*S*,*S*)-9** (Chiralpak IA, λ = 240 nm, n-heptane/CH_2Cl_2 = 75/25, 0.5 mL min^{-1}, 22 °C): t_R = 4.1 min (minor), 6.3 min (major), >99% *ee.*

Method: IA-CHIRALPAK, nHept_ChCl3, 85_15, 1ml, 22°C, HERMANN.met

Vial: 193 Injection Volume (µl): 2
Run Time: 13.02.2019 10:04:44 Analysis Time: 13.02.2019 13:47:50

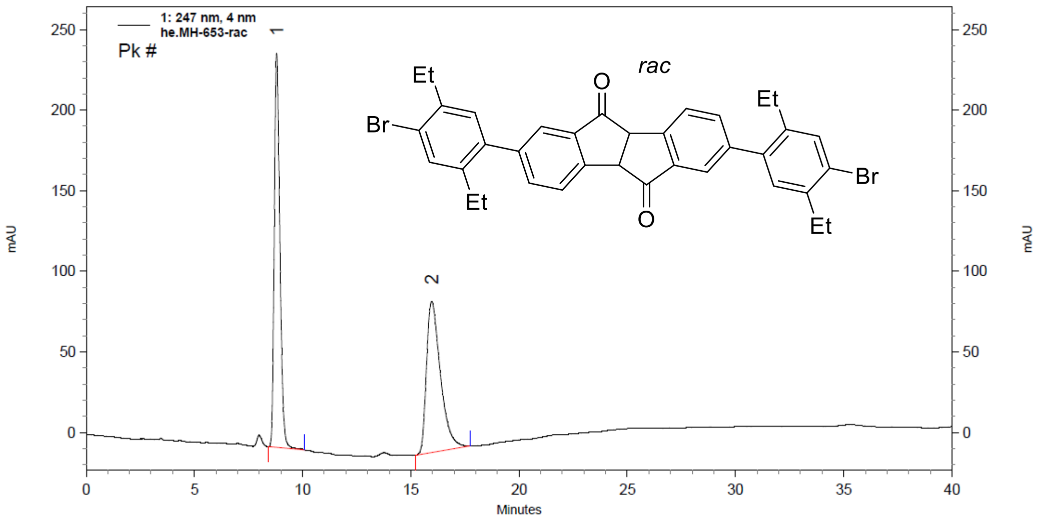

1: 247 nm, 4 nm Results

Pk #	Retention Time	Area Percent	Lambda Max
1	8,800	51,118	247
2	15,967	48,882	245

Method: IA-CHIRALPAK, nHept_ChCl3, 85_15, 1ml, 22°C, HERMANN.met

Vial: 194 Injection Volume (µl): 2
Run Time: 13.02.2019 10:48:55 Analysis Time: 13.02.2019 13:52:49

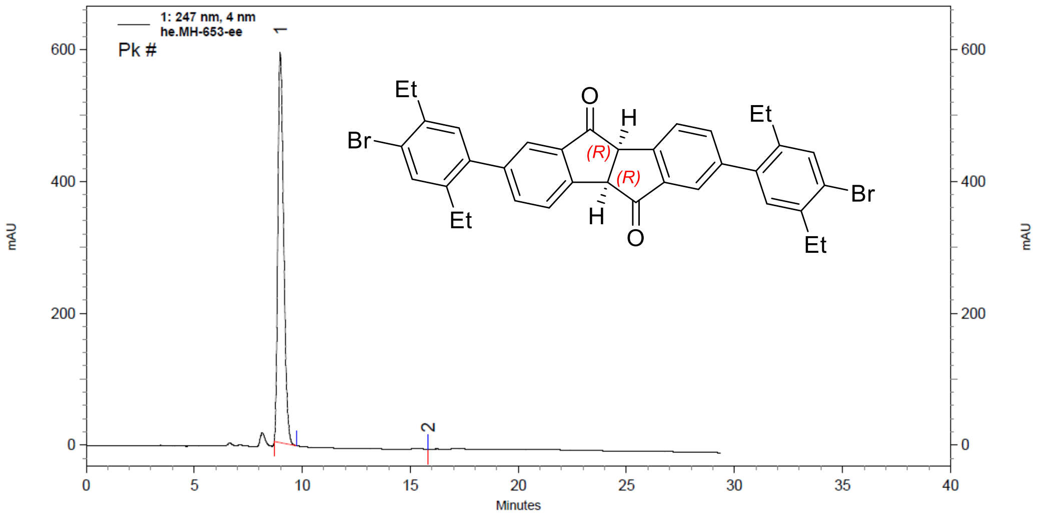

1: 247 nm, 4 nm Results

Pk #	Retention Time	Area Percent	Lambda Max
1	8,987	100,000	248
2	15,807	0,000	199

Figure S104. HPLC elugrams of ***rac*-10** and **(*R*,*R*)-10** (Chiralpak IA, λ = 247 nm, *n*-heptane/$CHCl_3$ = 85/15, 1.0 mL min^{-1}, 22 °C): t_R = 8.8 min (major), 16.0 min (minor), > 99% *ee*.

Method: CHIRALPAK IA, nHeptan/DCM 60:40, 0,5ml/min, 22°C

Vial: 197 Injection Volume (µl): 5

Run Time: 13.11.2020 07:59:27 Analysis Time: 13.11.2020 08:44:51

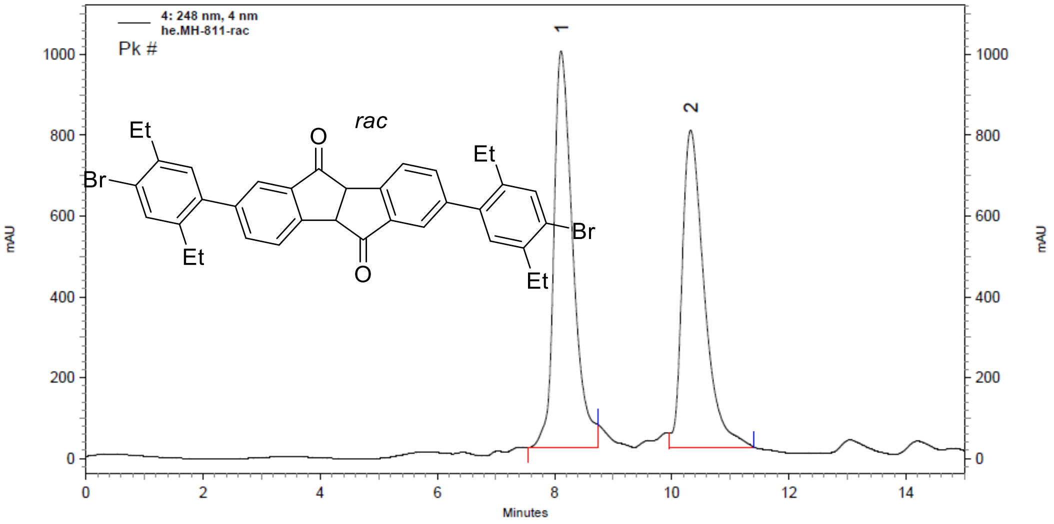

4: 248 nm, 4 nm Results

Pk #	Retention Time	Area Percent	Lambda Max
1	8,113	51,907	248
2	10,327	48,093	248

Method: CHIRALPAK IA, nHeptan/DCM 60:40, 0,5ml/min, 22°C

Vial: 181 Injection Volume (µl): 5

Run Time: 13.11.2020 08:16:05 Analysis Time: 13.11.2020 13:20:35

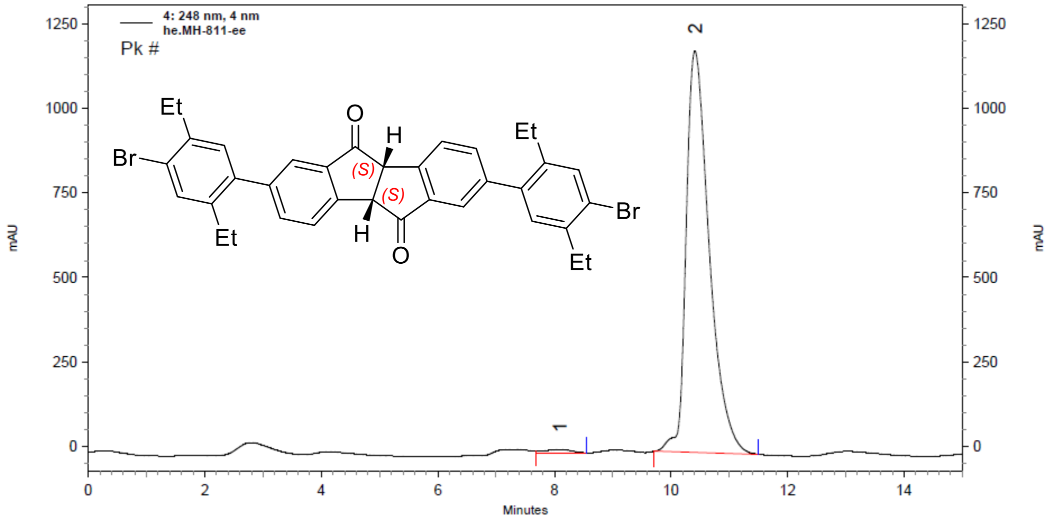

4: 248 nm, 4 nm Results

Pk #	Retention Time	Area Percent	Lambda Max
1	8,093	0,882	356
2	10,413	99,118	248

Figure S105.HPLC elugrams of of ***rac*-10** and **(*S*,*S*)-10** (Chiralpak IA, λ = 248 nm, *n*-heptane/CH_2Cl_2 = 60/40, 0.5 mL min^{-1}, 22°C): t_R = 8.1 min (minor), 10.4 min (major), 98% *ee*.

8. Cyclic Voltammograms

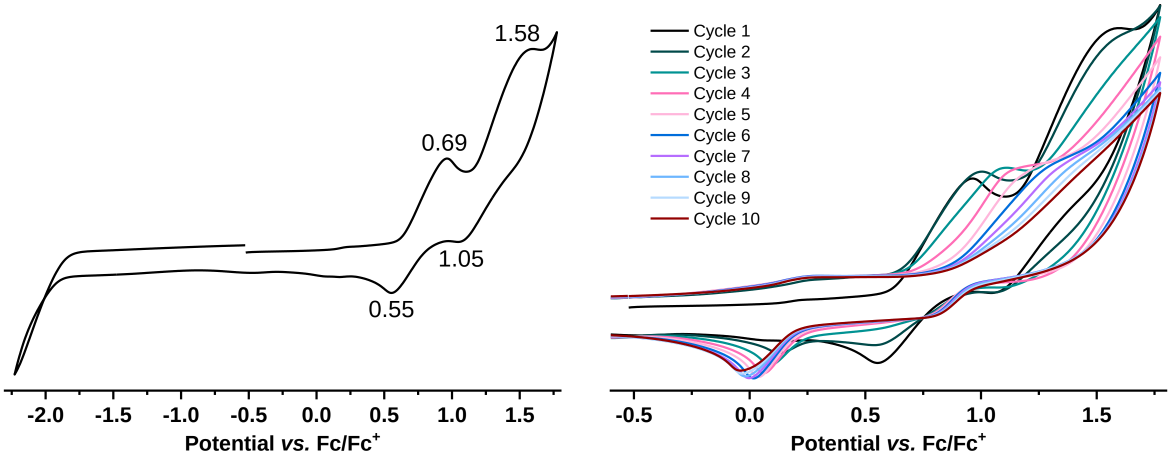

Figure S106. CV of **7** in chloroform solution, GC electrode, 0.34 mM analyte concentration, scan rate 100 mV/s. Right: the first 10 cycles are shown. The current decreases with increasing cycle number, indicating some irreversible reaction on the electrode surface.

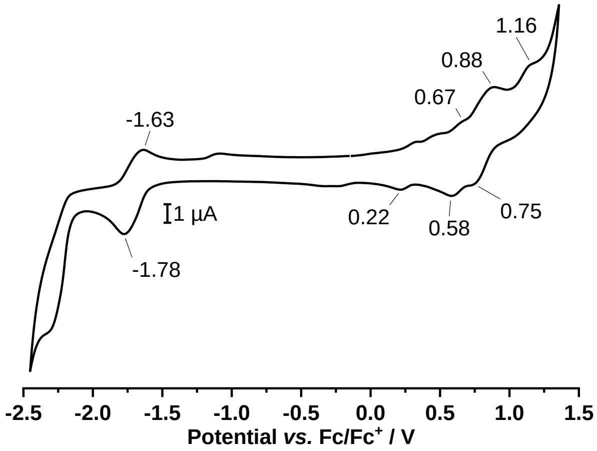

Figure S107. CV of **(+)-1** in CH_2Cl_2 solution, GC electrode, 0.16 mM analyte concentration, scan rate 100 mV/s: full range of solvent potential window, several irreversible redox waves are visible.

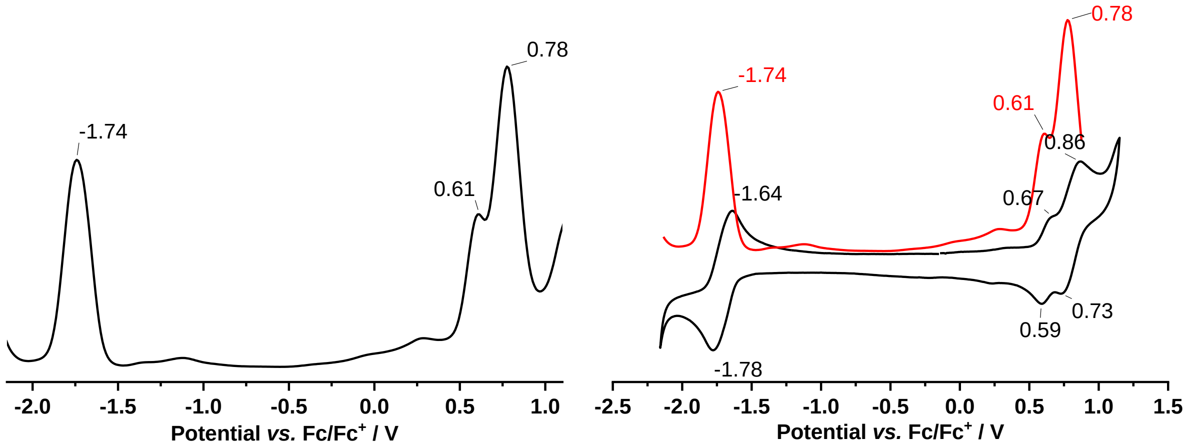

Figure S108. DPV of **(+)-1** in CH_2Cl_2 solution, GC electrode, 0.16 M analyte concentration, scan rate 100 mV/s. Right: overlay of the DPV (red) and the CV.

9. Absorption Spectroscopy

9.1 UV/Vis Absorption Spectra

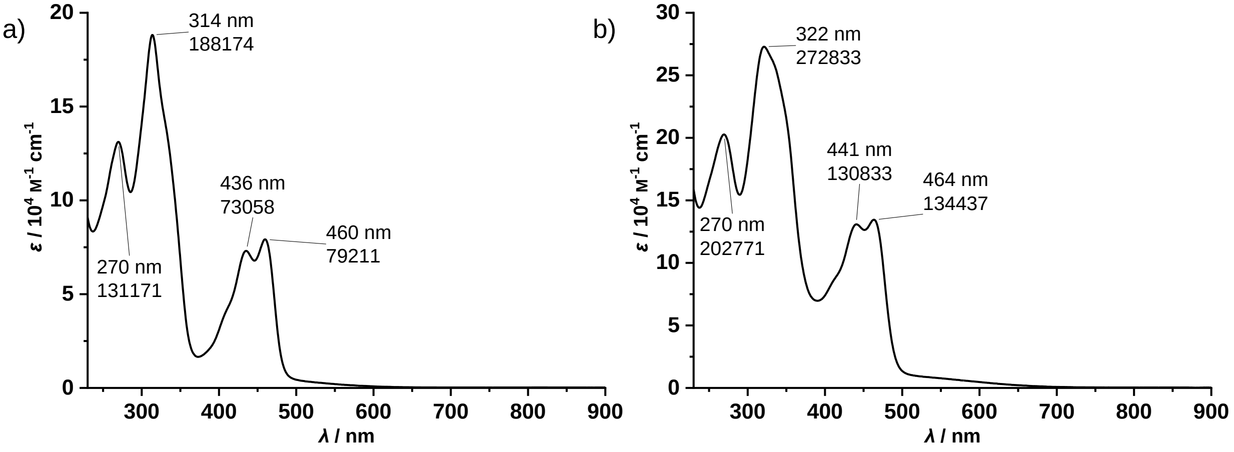

Figure S109. UV/Vis absorption spectra of **7** (a) and **(+)-1** (b) in CH_2Cl_2 solution with epsilon values.

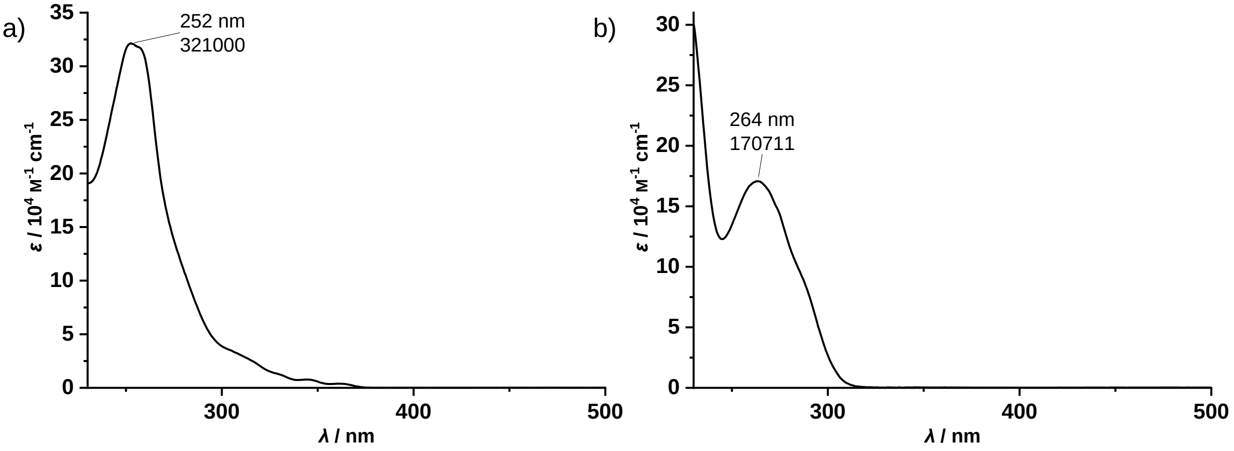

Figure S110. UV/Vis absorption spectra of **(*R*,*R*)^6-14** (a) and **(*R*,*R*)^6-(+)-16** (b) in CH_2Cl_2 solution.

9.2 Electronic Circular Dichroism Spectra

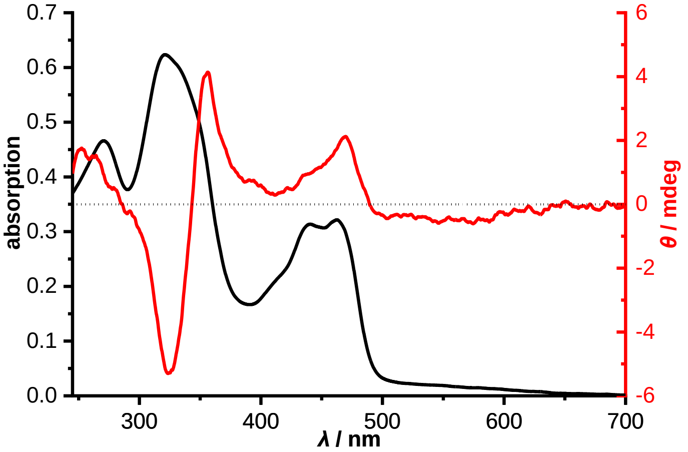

Figure S111. Overlay of ECD spectrum (red, right y-axis) and UV/Vis absorption spectrum (black, left y-axis) of **(+)-1** in $CHCl_3$ solution (c = 1.33 · 10^{-5} M) at 20 °C.

9.2.1 ECD spectra of chiral precursor compounds

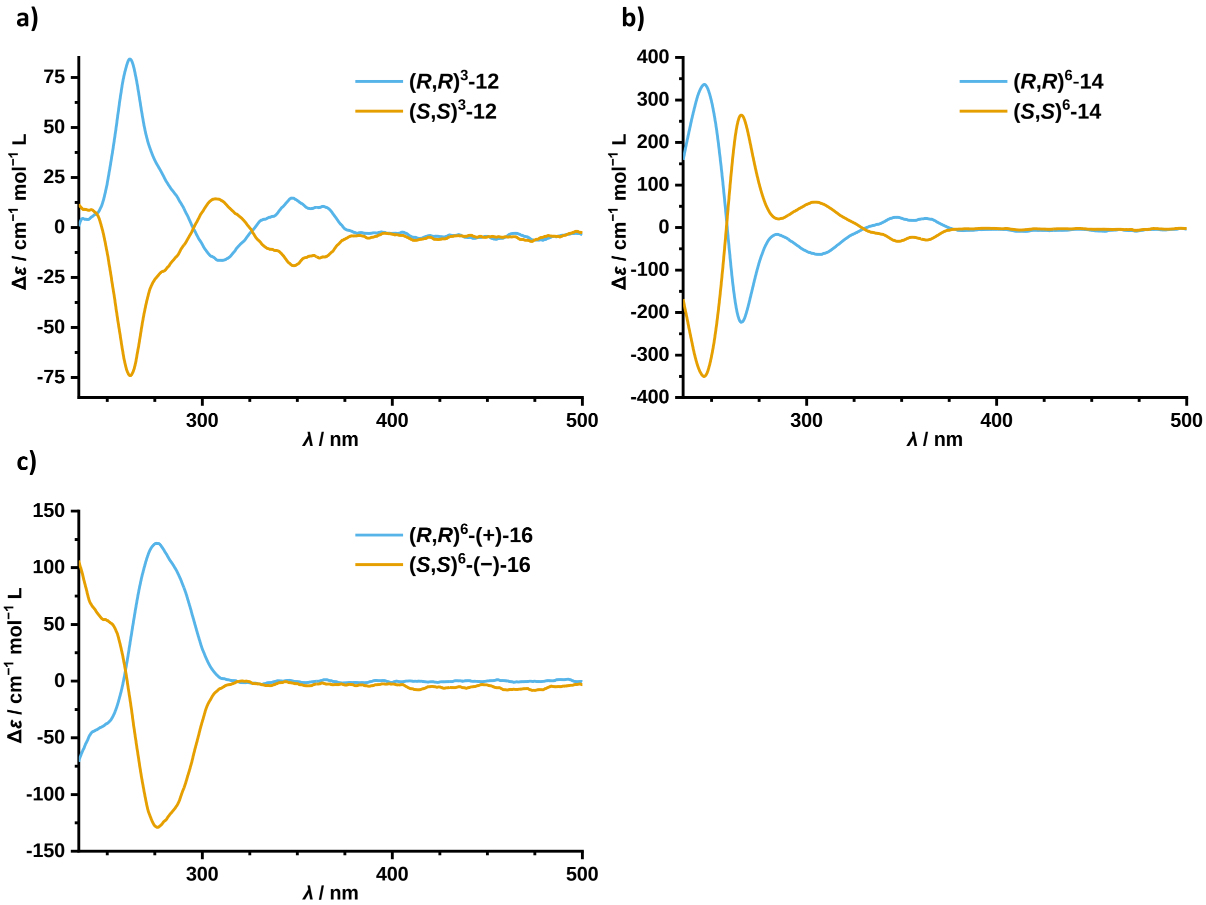

Figure S112. ECD absorption spectra of a) **(*R*,*R*)³-12** and **(*S*,*S*)³-12**, b) **(*R*,*R*)⁶-14** and **(*S*,*S*)⁶-14**, c) **(*R*,*R*)⁶-(+)-16** and **(*S*,*S*)⁶-(−)-16** in CH_2Cl_2 solution at 20 °C.

9.2.2 Temperature-dependent ECD-spectroscopic measurements

Correction of Concentration Fluctuation During ECD Measurements

UV/Vis absorption spectra in chlorobenzene of **(+)-1** were recorded during the measurement of the ECD spectra while cooling from 110 °C to 20 °C (Figure 5D in the manuscript) and used for correction of the concentration. ECD spectra were normalized according to the factor obtained from the main absorption at 324 nm in the UV/Vis spectrum (Figure S113).

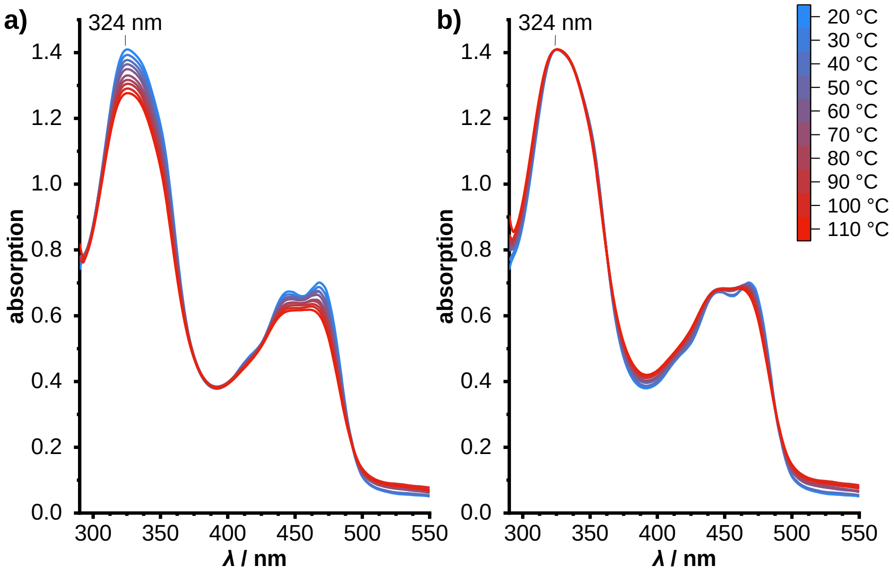

Figure S113. A) Uncorrected UV/Vis absorption spectra of **(+)-1** during the ECD measurement while cooling from 110 °C to 20 °C. b) Corrected absorption spectra based upon the normalized signal intensity at 324 nm.

The temperature range of 40–80 °C was investigated in more detail, since the largest change took place between 50–60 °C.

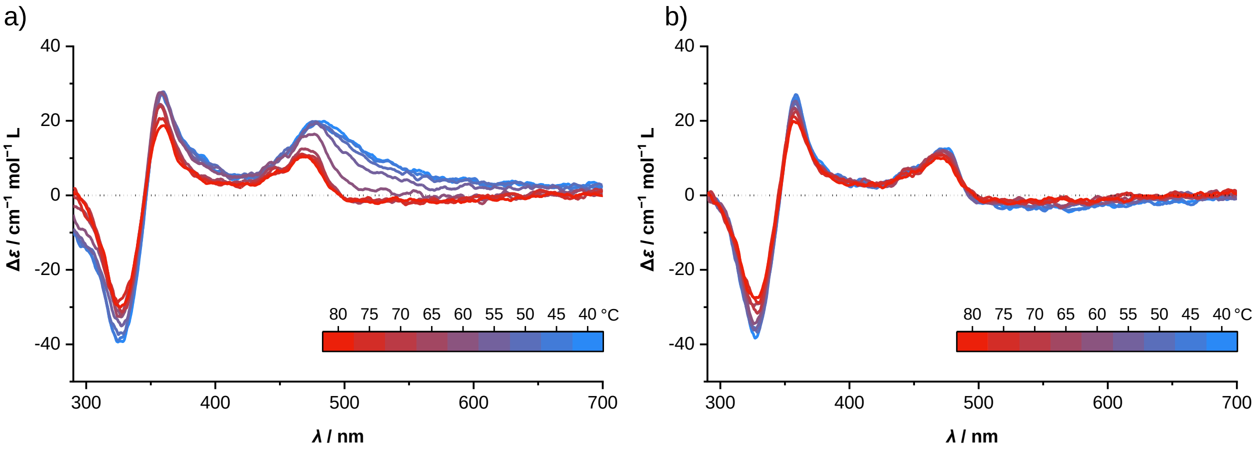

Figure S114. ECD spectra of **(+)-1** (c = 3.34 · 10^{-5} M) in chlorobenzene solution a) while heating from 40 °C with a rate of 2 °C/min to 80 °C, b) while cooling from 80 °C with a rate of 2 °C/min to 40 °C, measurement in 5 °C steps.

9.2.3 Calculated ECD spectra

The electronic circular dichroism spectra of all nanohoop stereoisomers **1a**–**1n** were simulated. For this 100 snapshots of the 100 ps lasting MD-simulation on the GFN2-xTB/GBSA(toluene) level were taken and CD spectra calculated on the sTDA-xTB level. The linewidth of the resulting spectra was adjusted to better fit the experiment but no shifting needed to be applied.

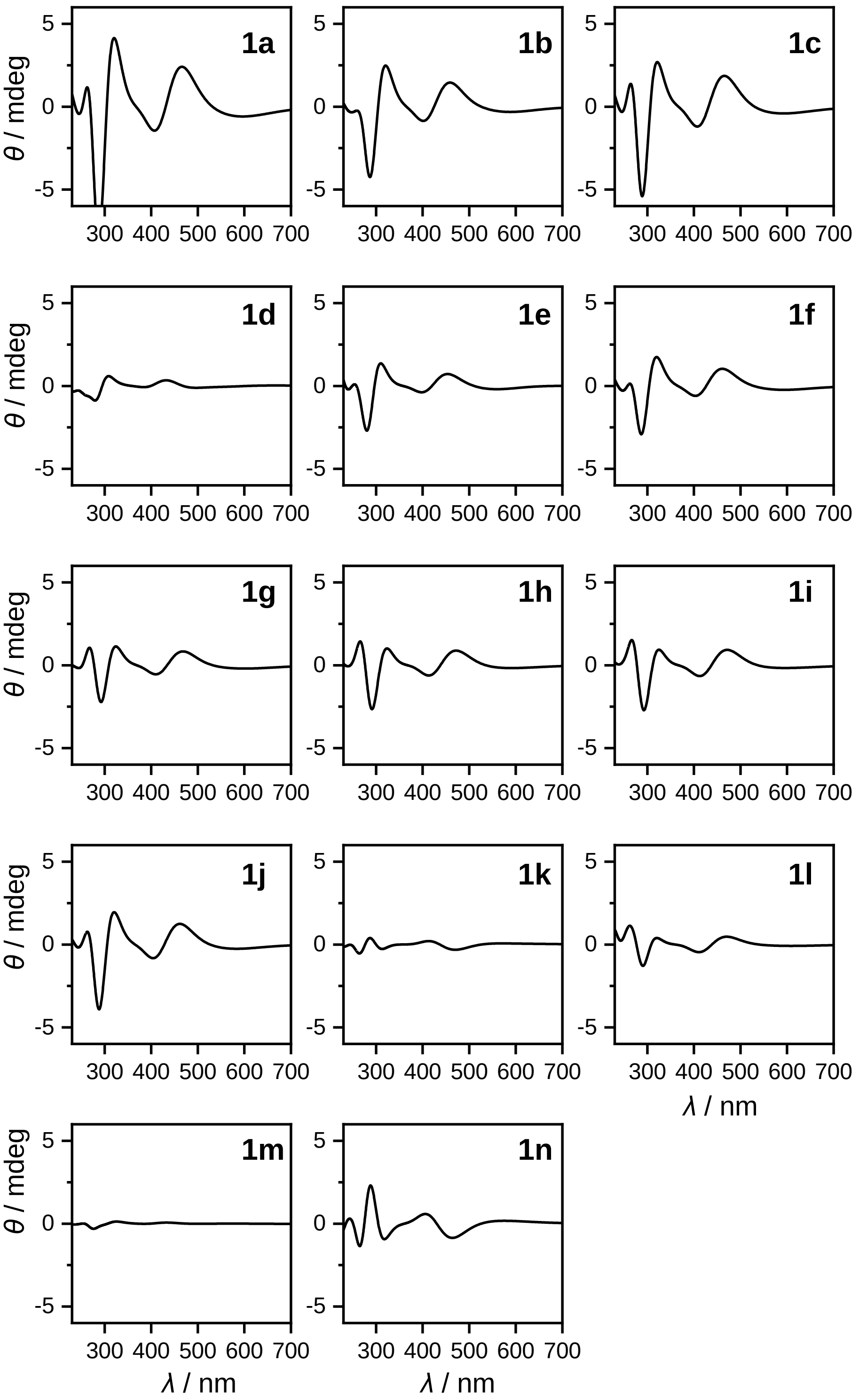

Figure S115. Calculated CD spectra (calculated on the sTDA-xTB level out of 100 snapshots of a GFN2-xTB/GBSA(toluene) MD-simulation of 100 ps length) of all possible stereoisomers **1a**–**1n** of nanohoop **1**. Numbering scheme according to Figure 4 in the manuscript.

To investigate the temperature-dependence of the ECD-signals of the stereoisomers of **1**, for two stereoisomers, **1a** and **1n** (as highest energy conformer), CD spectra were simulated both at 300 and at 400 K. Calculations were performed on GFN2-xTB/GBSA(toluene) level of theory using the xtb 6.3.2 program package. The MD length was set to 200 ps, trajectory dump step was 50 fs, Berendsen thermostat was used, all bonds were constrained using the shake algorithm and all other properties were kept at their default values. CD spectra were calculated on sTDA-xTB level of theory using the last 100 ps (2000 steps) of each trajectory in steps of 10 snapshots. The linewidth of the resulting spectrum was adjusted to better fit the experiment but no shifting needed to be applied.

As can be seen in Figure S116, the spectrum of **1a** only slightly changes with increasing temperature to a lower intensity of all signals. For **1n**, the change is more dramatic, which is due to one of the DBP units rotating at 400 K to furnish a different stereoisomer, which changes its ECD spectrum significantly.

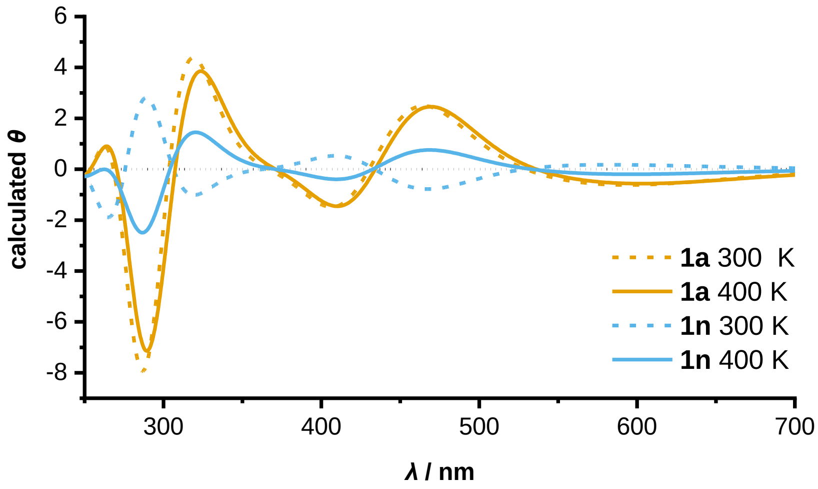

Figure S116. Calculated CD spectra of **1a** and **1n** at 300 K and 400 K.

10. Mass Spectra

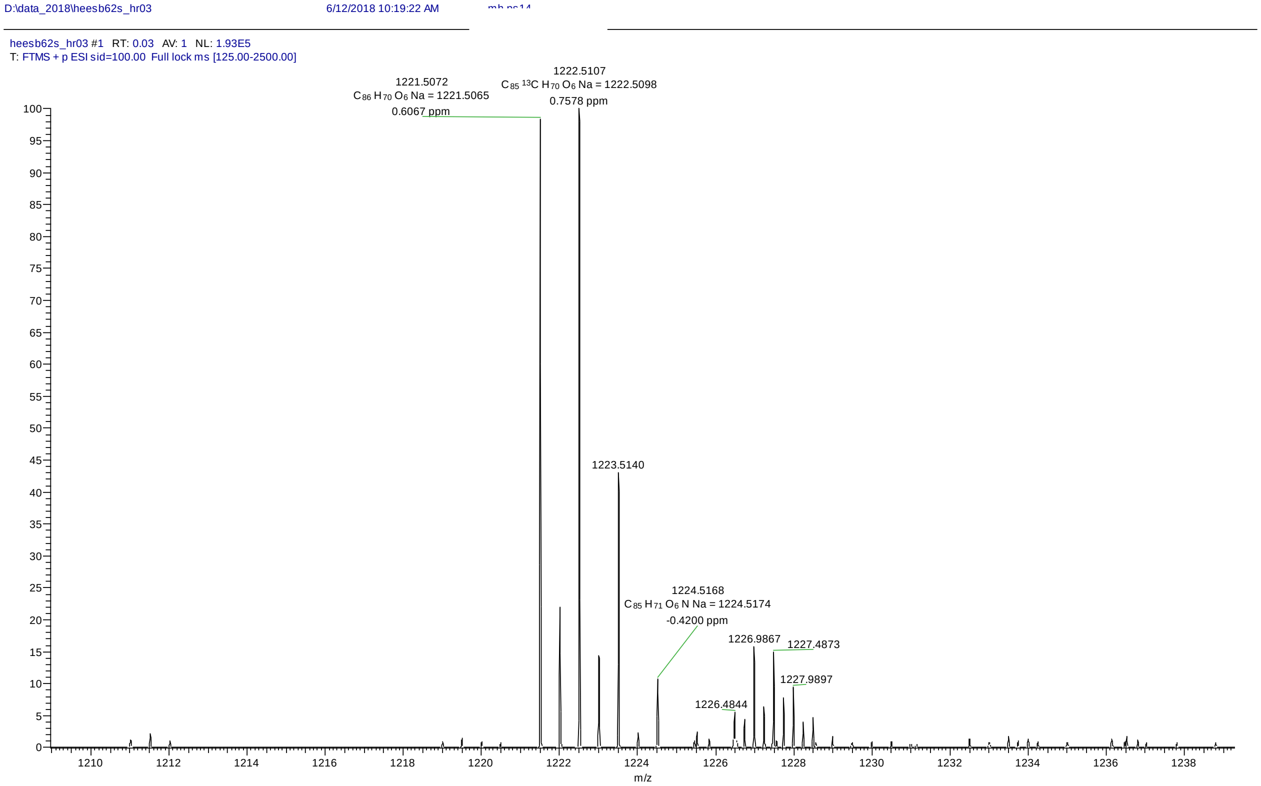

Figure S117. Mass spectrum of **19**. Positive ESI mode, ionized as $[M+Na]^+$.

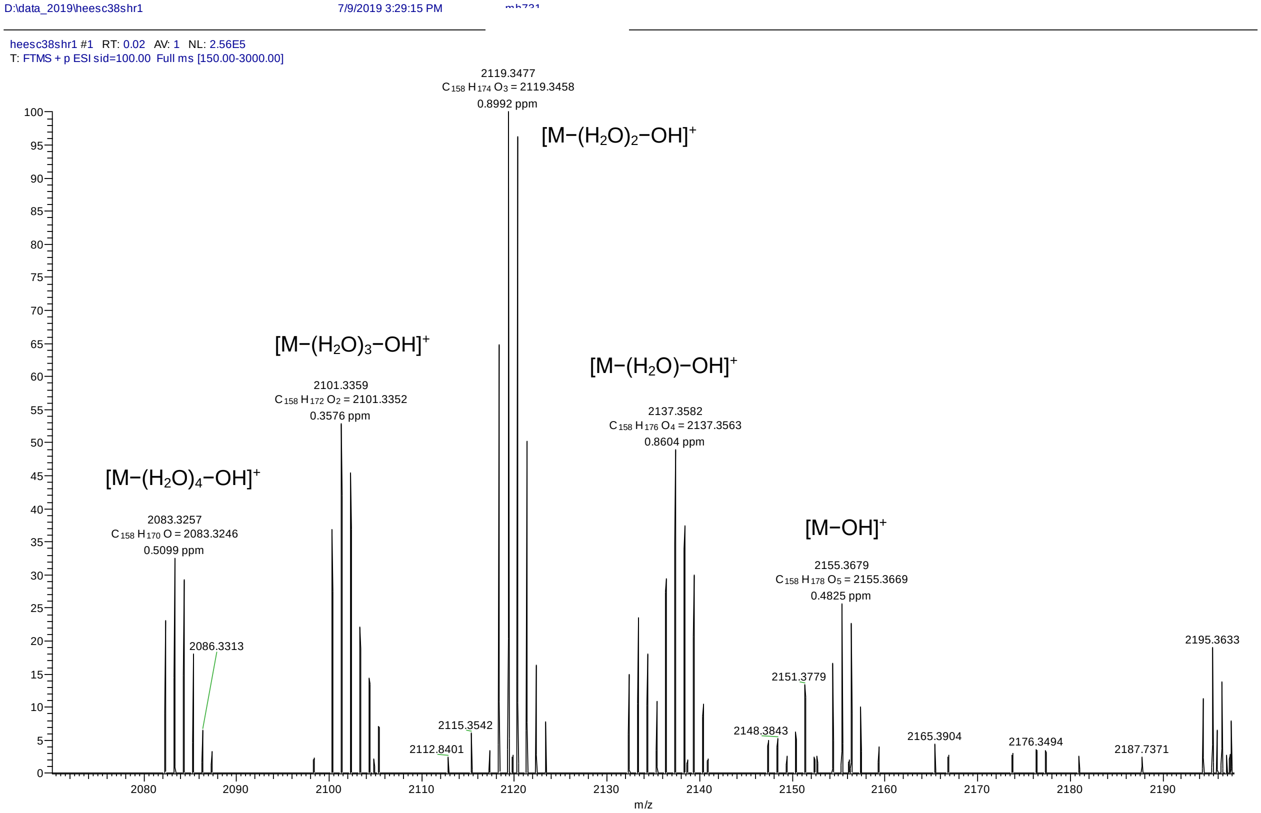

Figure S118. Mass spectrum of **S4**. Positive ESI mode.

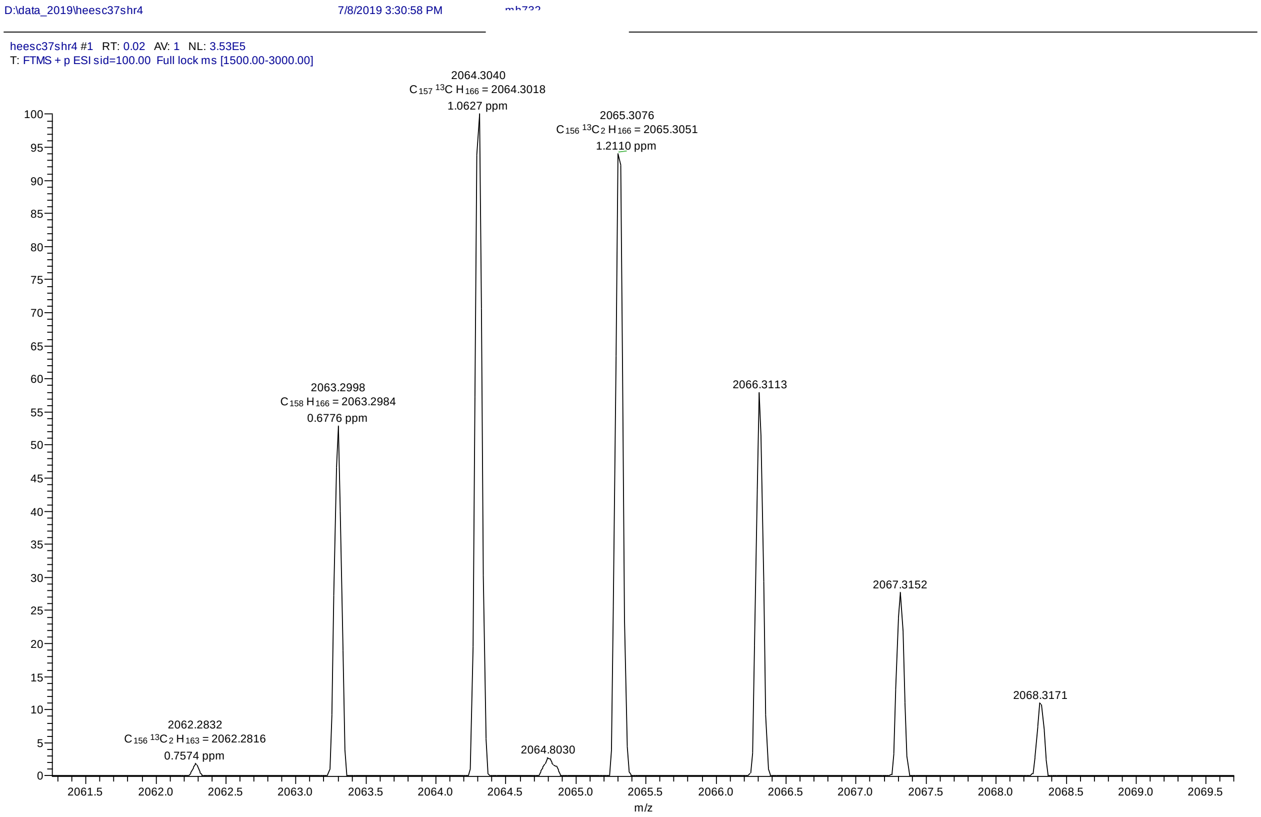

Figure S119. Mass spectrum of **7**. Positive ESI mode, ionized as $[M+H]^+$.

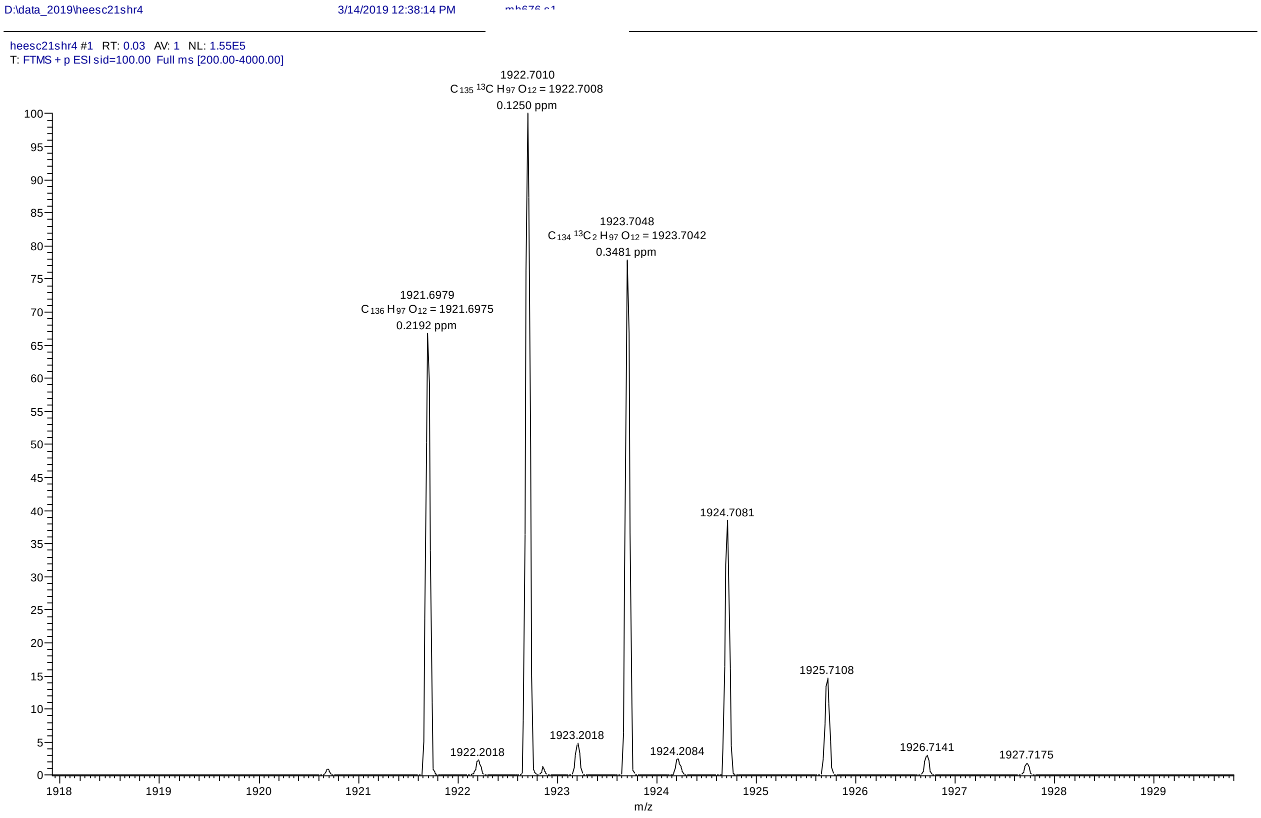

Figure S120. Mass spectrum of ***(R,R)*^{6}-14**. Positive ESI mode, ionized as $[M+H]^+$.

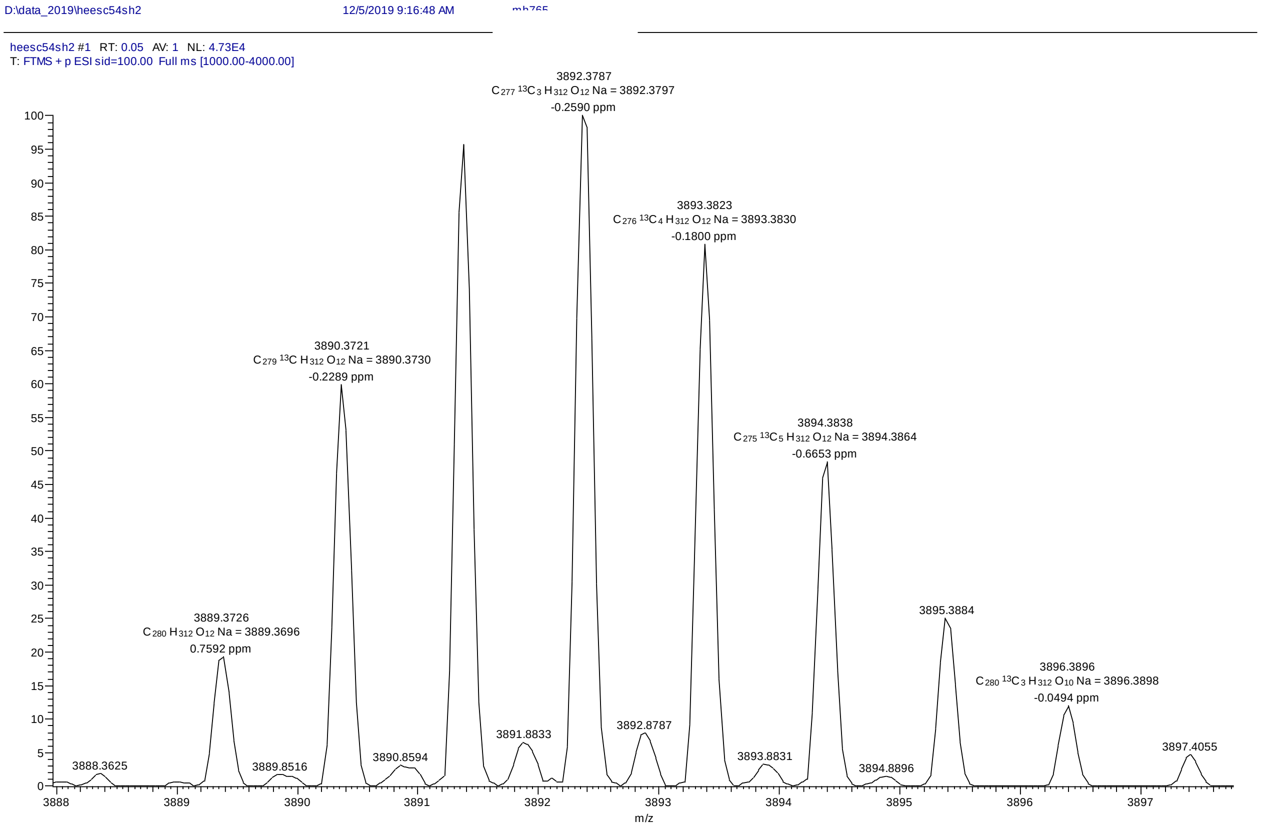

Figure S121. Mass spectrum of **(*R*,*R*)^6-(+)-16**. Positive ESI mode, ionized as [M+Na]$^+$.

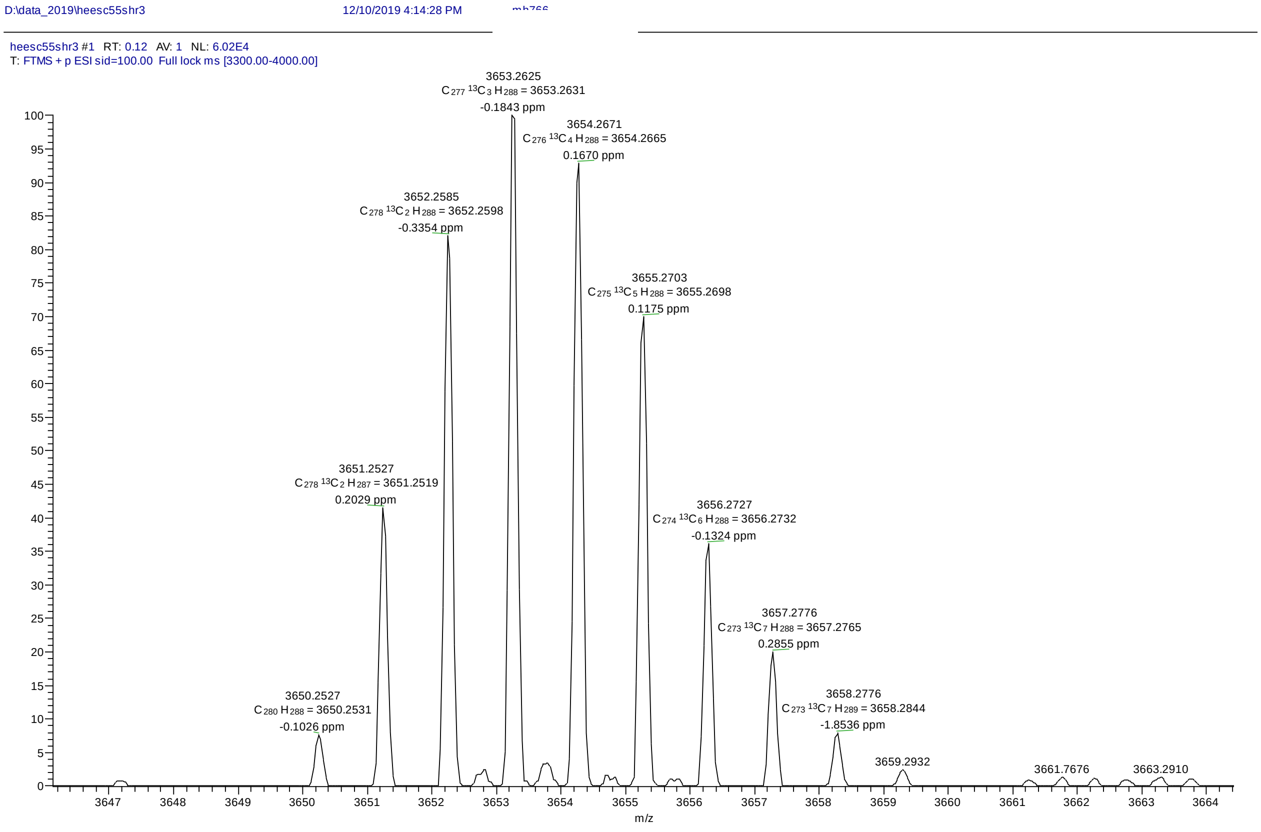

Figure S122. Mass spectrum of **(+)-1**. Positive ESI mode, ionized as [M]$^{•+}$.

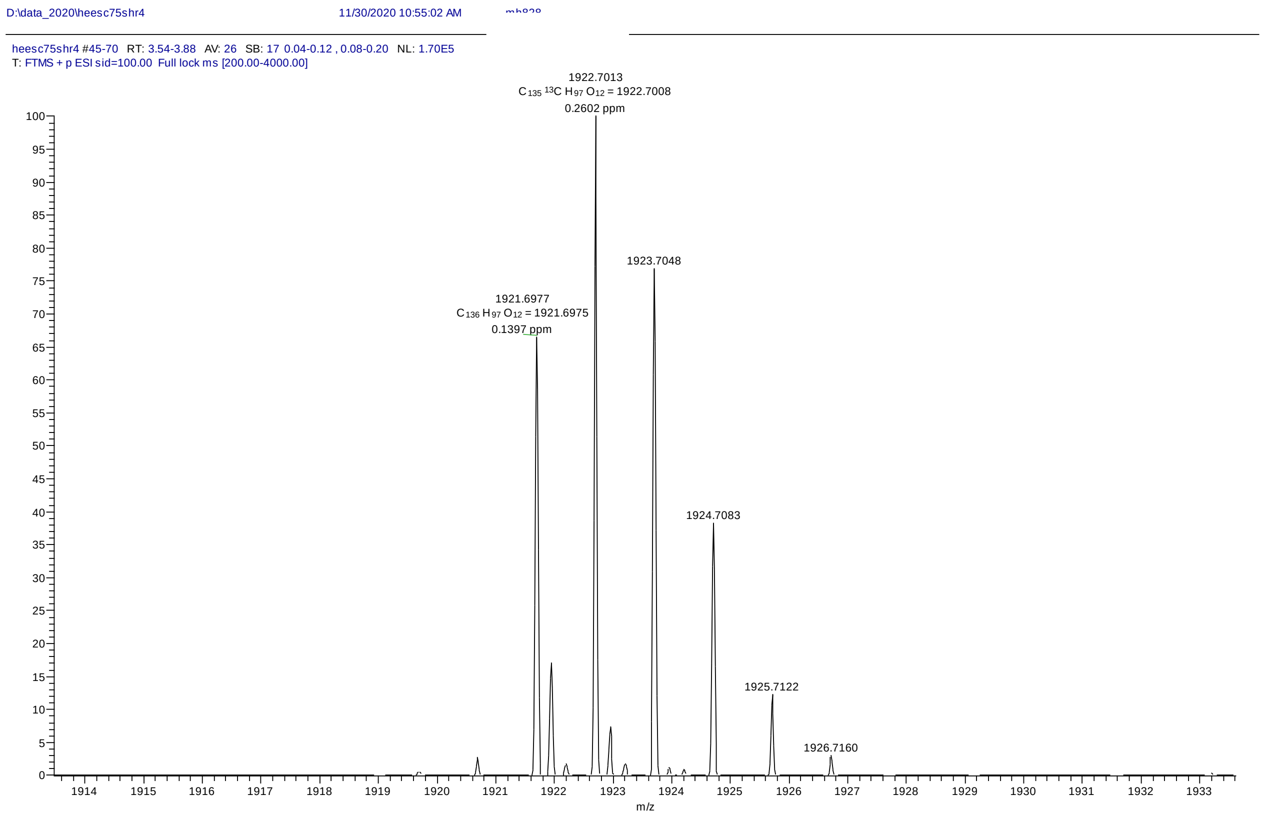

Figure S123. Mass spectrum of **(*S*,*S*)[6]-14**. Positive ESI mode, ionized as $[M+H]^+$.

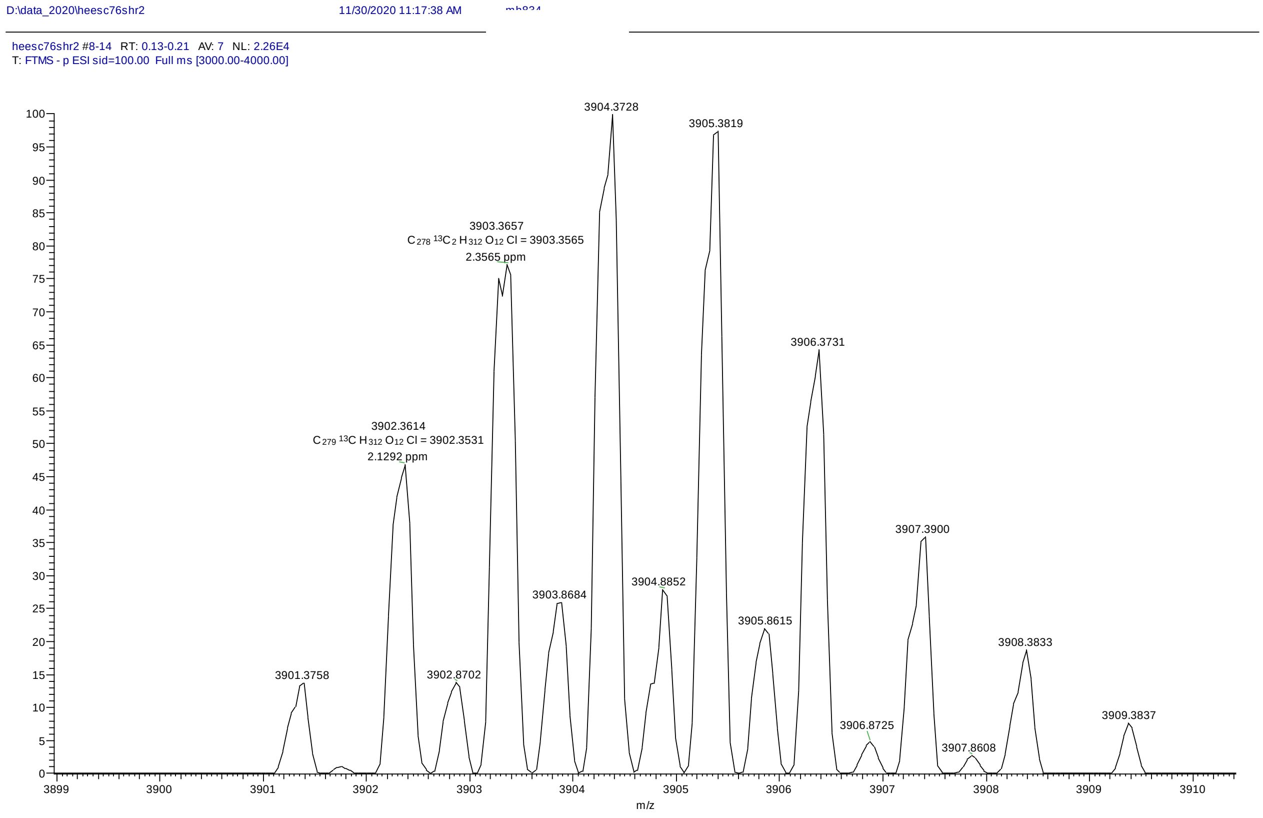

Figure S124. Mass spectrum of **(*S*,*S*)[6]-(−)-16**. Negative ESI mode, ionized as $[M+Cl]^-$.

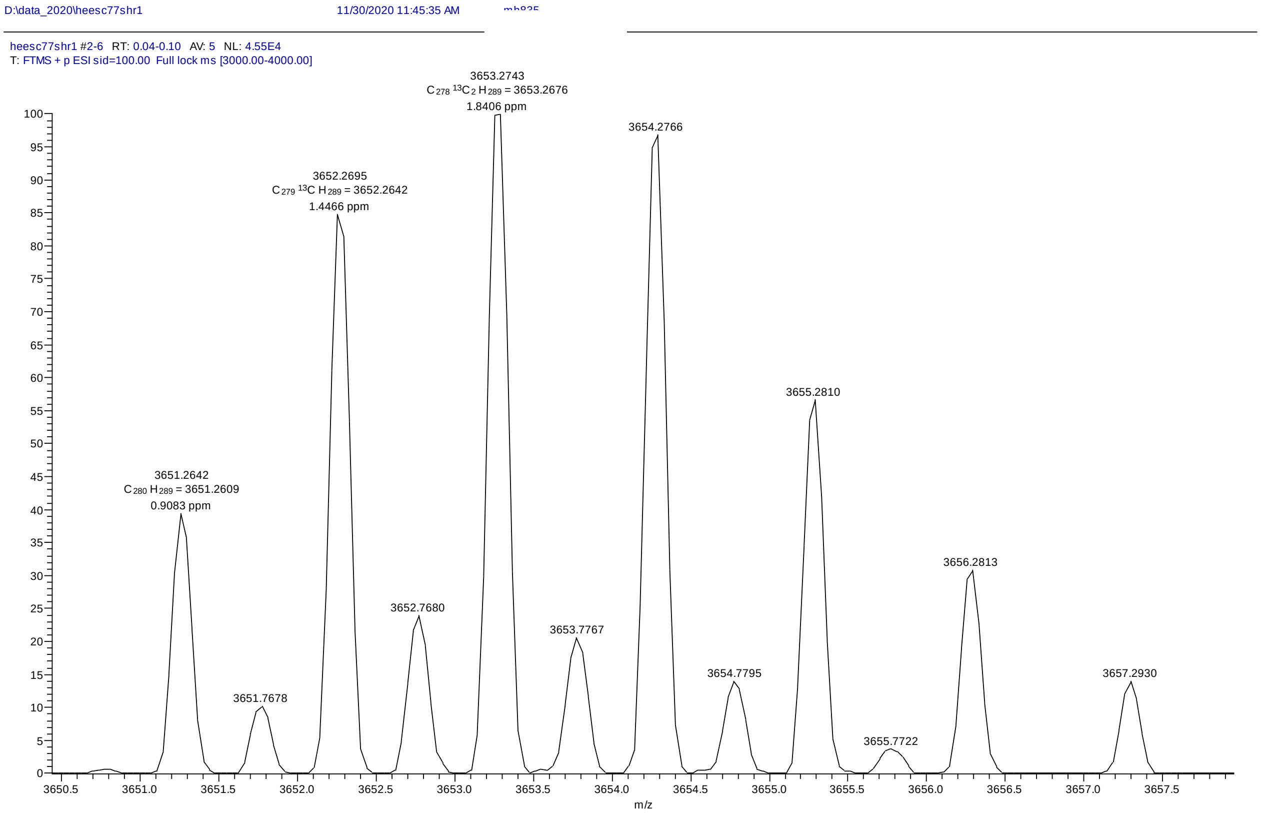

Figure S125. Mass spectrum of **(−)-1**. Positive ESI mode, ionized as $[M+H]^+$.

11. Single-Crystal X-Ray Diffraction

Single crystal X-ray diffraction was performed at the Institute for Inorganic and Analytical Chemistry at the University of Freiburg. The structure was solved by Dr. Daniel Kratzert.

11.1 General Experimental Setup

The crystals were mounted on a MITIGEN holder in perfluoroether oil. X-ray diffraction data were collected using a BRUKER SMART APEXII QUAZAR diffractometer equipped with an Oxford Cryosystems 800 low-temperature device, operating at T = 100 K. Data were measured using ω and ϕ scans scans using MoK_α radiation (microfocus sealed X-ray tube, 50 kV, 0.6 mA). The total number of runs and images was based on the strategy calculation from the program APEX2 (BRUKER). Cell parameters were retrieved using the SAINT software and refined using SAINT.[14] Data reduction was performed using the SAINT[14] software which corrects for Lorentz polarisation.
Scaling and absorption correction was performed by SADABS.[15] Severe disorder was modelled using DSR[16] or FragmentDB[16] in ShelXle[17] or Olex2.[18]

11.2 Solution and Refinement

A single crystal of **6** was obtained by layering a $CHCl_3$ solution with *n*-pentane. The data were collected from a shock-cooled single crystal at 100(2) K on Bruker APEX2 QUAZAR three-circle diffractometer with a microfocus sealed X-ray tube using mirror optics as monochromator and a Bruker APEXII detector. The diffractometer was equipped with an Oxford Cryostream 800 low temperature device and used MoK_α radiation (λ = 0.71073 Å). All data were integrated with SAINT[14] and a multi-scan absorption correction using SADABS-2014/5[15] was applied. The structure was solved by direct methods using SHELXT-2012/1,[19] and refined by full-matrix least-squares methods against F^2 by SHELXL-2014/7.[20] All non-hydrogen atoms were refined with anisotropic displacement parameters. The hydrogen atoms were refined isotropically on calculated positions using a riding model with their U_{iso} values constrained to 1.5 times the U_{eq} of their pivot atoms for terminal sp^3 carbon atoms and 1.2 times for all other carbon atoms. Disordered moieties were refined using bond lengths restraints and displacement parameter restraints. Crystallographic data (including structure factors) for the structures reported in this paper have been deposited with the Cambridge Crystallographic Data Centre. CCDC 1483358 contain the supplementary crystallographic data for this paper. Copies of the data can be obtained free of charge from The Cambridge Crystallographic Data Centre via www.ccdc.cam.ac.uk/structures.

Molecule	**6**
CCDC number	1483358
Empirical formula	$C_{33}H_{31}Br_2Cl_3N_2O_4S_2$
Formula weight	849.89
Temperature [K]	100(2)
Crystal system	orthorhombic
Space group (number)	$P2_12_12_1$ (19)
a [Å]	11.9679(3)
b [Å]	13.6936(3)
c [Å]	21.2165(5)
α [Å]	90
β [Å]	90
γ [Å]	90
Volume [Å^3]	3477.04(14)
Z	4
ρ_{calc} [g/cm^3]	1.624
μ [mm^{-1}]	2.723
F(000)	1712
Crystal size [mm^3]	0.15×0.12×0.04
Crystal colour	colourless
Crystal shape	block
Radiation	MoK_α (λ=0.71073 Å)
2Θ range [°]	3.54 to 58.38
Index ranges	−16 ≤ h ≤ 15<br>−18 ≤ k ≤ 18<br>−28 ≤ l ≤ 27
Reflections collected	50414
Independent reflections	9355<br>R_{int} = 0.0326<br>R_{sigma} = 0.0514
Completeness to θ = 25.242°	100.0%
Data / Restraints / Parameters	9355/30/454
Goodness-of-fit on F^2	0.994
Final *R* indexes [I≥2σ(I)]	R_1 = 0.0286<br>wR_2 = 0.0555
Final *R* indexes [all data]	R_1 = 0.0405<br>wR_2 = 0.0577
Largest peak/hole [eÅ^3]	0.60/−0.58
**Flack X parameter**	**−0.002(3)**

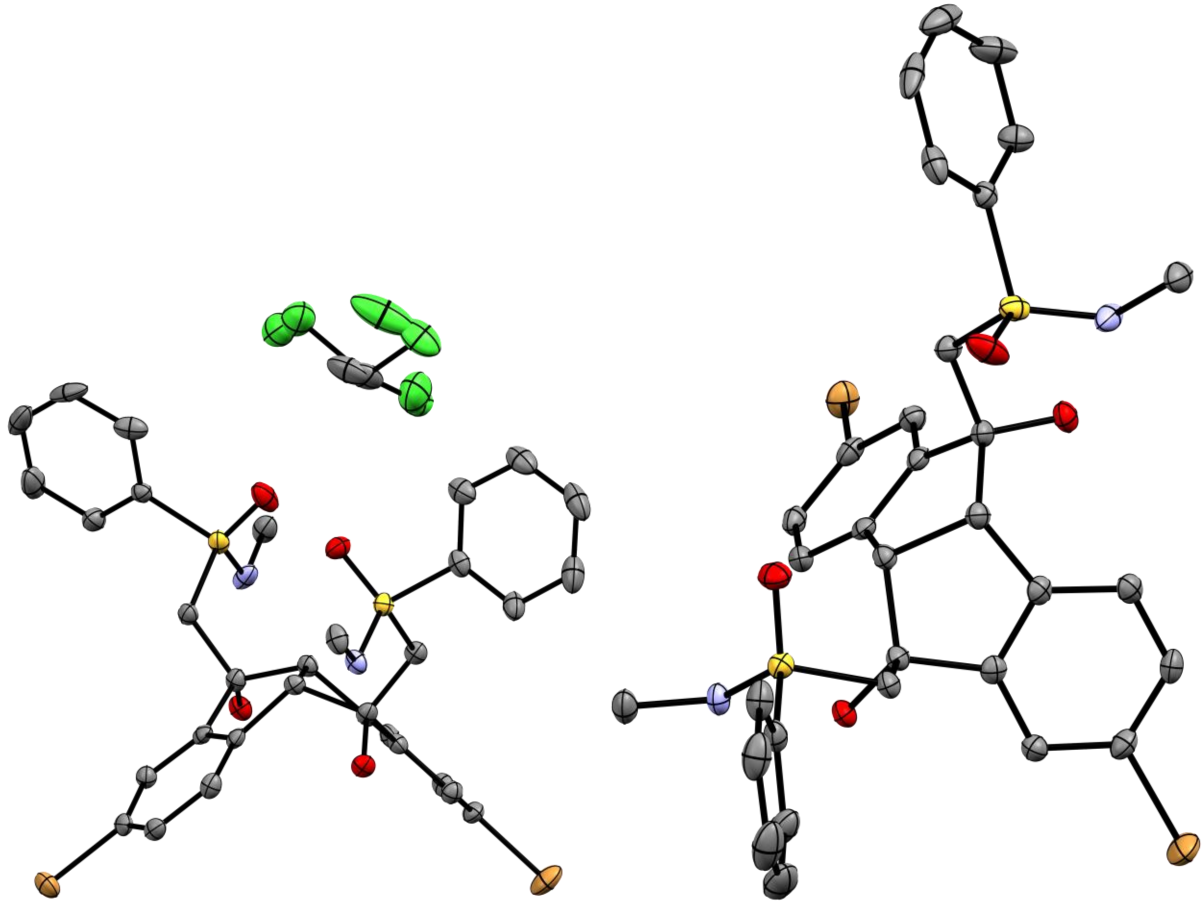

Figure S126. Molecular structure of **6** in the solid state. Thermal ellipsoids displayed with 50% probability, H-atoms omitted for clarity. Left: $CHCl_3$ solvent molecule shown, right: solvent molecule omitted for clarity. The absolute structure could be determined from this single X-ray structure.

The absolute configuration of **6** was unambiguously determined through X-ray diffraction. Therefore the absolute structure of diketone **3** is also known, since elimination of **6** yields **(*S*,*S*)-3** and elimination of **5** yields **(*R*,*R*)-3**. Furthermore diketone **(*S*,*S*)-3**, which was obtained by thermolysis of **6**, could also be investigated in the solid state (CCDC: 1479037[4]) and showed the correct absolute configuration.

12. NMR Titration Studies of 1 with Fullerene-C_{60}

The experiment was conducted by adding a solution of C_{60} toluene-d_8 to a solution of **1** toluene-d_8 in an NMR tube, and recording an NMR spectrum each time. The experiment was performed in triplicate, each time with different starting concentrations (c_0) of **1** and the data can be accessed using the following URLs:

**experiment 1** ($c_0 = 5.5 \cdot 10^{-4}$ mol L^{-1})	
1:1 fit	http://app.supramolecular.org/bindfit/view/21819049-7cd8-4639-a9fc-2af88b2c1cdf
1:2 fit	http://app.supramolecular.org/bindfit/view/dbc4da64-a0b2-4627-a5c3-c8446f89b319
**experiment 2** ($c_0 = 8.8 \cdot 10^{-4}$ mol L^{-1})	
1:1 fit	http://app.supramolecular.org/bindfit/view/2247bd3c-58e3-4bd4-9253-90bca0e393fc
1:2 fit	http://app.supramolecular.org/bindfit/view/2e1316fe-cb0e-424d-a09f-1cab97f0e686
**experiment 3** ($c_0 = 7.0 \cdot 10^{-4}$ mol L^{-1})	
1:1 fit	http://app.supramolecular.org/bindfit/view/6abaae0a-83d2-42c0-b6f6-f99535d0f57e
1:2 fit	http://app.supramolecular.org/bindfit/view/6a49ca67-c65a-4141-9ace-91cd6968a363

The data was fitted with Bindfit using supramolecular.org according to Thordarson et al.[21–23]

A fit of the data is shown exemplary for experiment 1 in Figure S127. The 1:2 fit is a better binding model than the 1:1 fit.

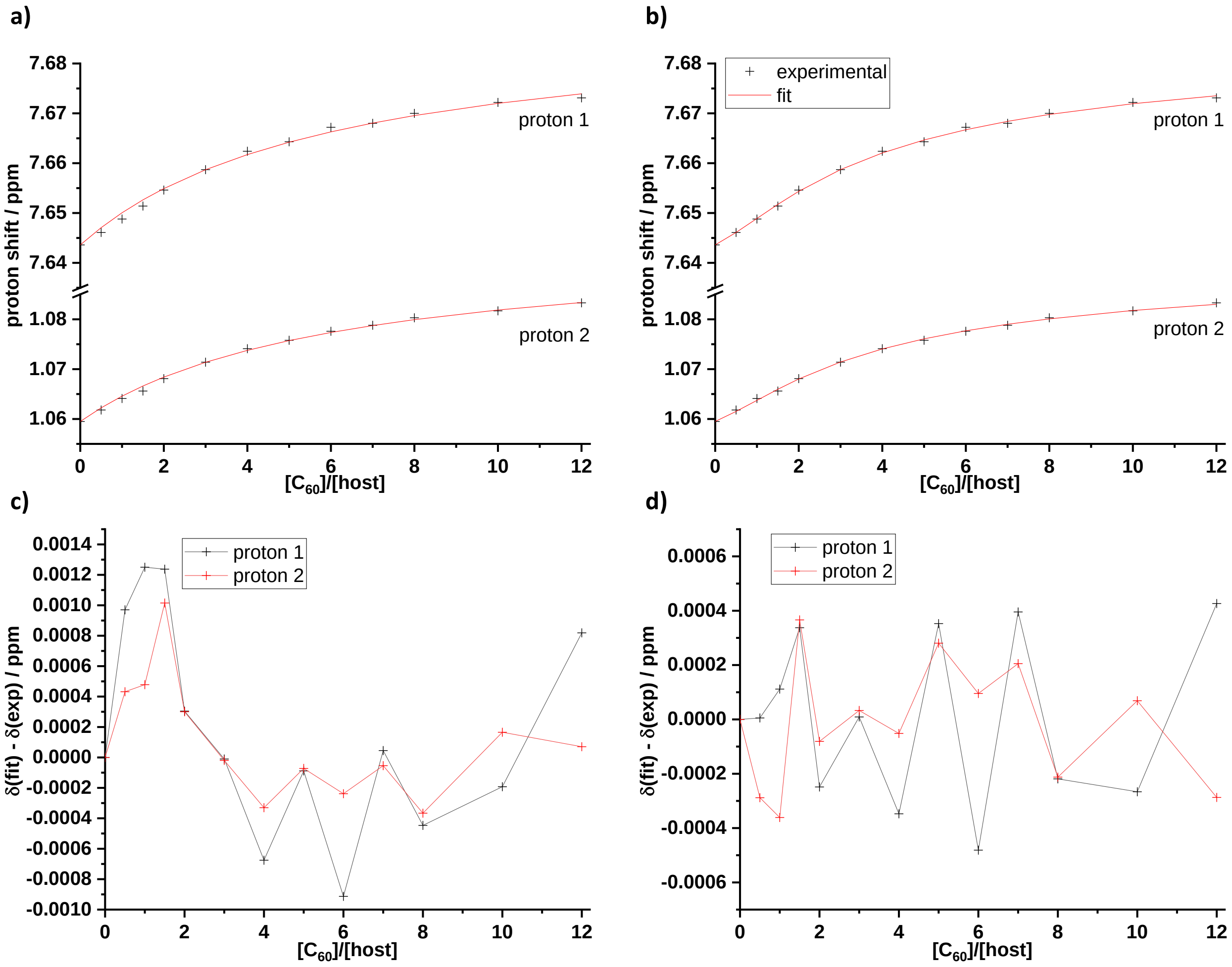

Figure S127. Binding isotherms of one titration experiment between **(+)-1** and C_{60} in toluene-d_8, fitted to a 1:1 (a) and 1:2 (b) binding model. Corresponding residuals between fitted and measured values for the 1:1 binding model (c) and the 1:2 binding model (d). Because of the more reasonable values for the 1:2 fit and the lower and better residuals, the 1:2 binding model is applicable in this case.

Table S2. Results of the titration experiments of **1** with C_{60}.

Experiment #	1	2	3	Weighted arithmetic mean*	arithmetic mean§
		**1:1 fit**			
K / M^{-1}	$6.2 \cdot 10^{-3}$	$8.6 \cdot 10^{-3}$	$4.9 \cdot 10^{-3}$		
Error	$\pm 0.031\ 10^{-3}$	$\pm 0.13\ 10^{-3}$	$\pm 0.019\ 10^{-3}$		
		**1:2 fit**			
K_{11} / M^{-1}	5862	4494	7743	**5382**	
Error	±1772	±1052	±2284	**±741**	
K_{12} / M^{-1}	226	71.3	35.4		**111**
Error	±8.8	±5.5	±1.2		**±101**

*The weighted arithmetic mean was used to calculate the mean of K_{11} because of the proximity of the individual values to the mean, which lay in the range of their errors. The weighted arithmetic mean considers the errors of each K_{11} value and weights them correspondingly.

§The arithmetic mean was used to calculate the mean of K_{12} because of the wider distribution of each value. The corresponding error is the sample standard deviation.

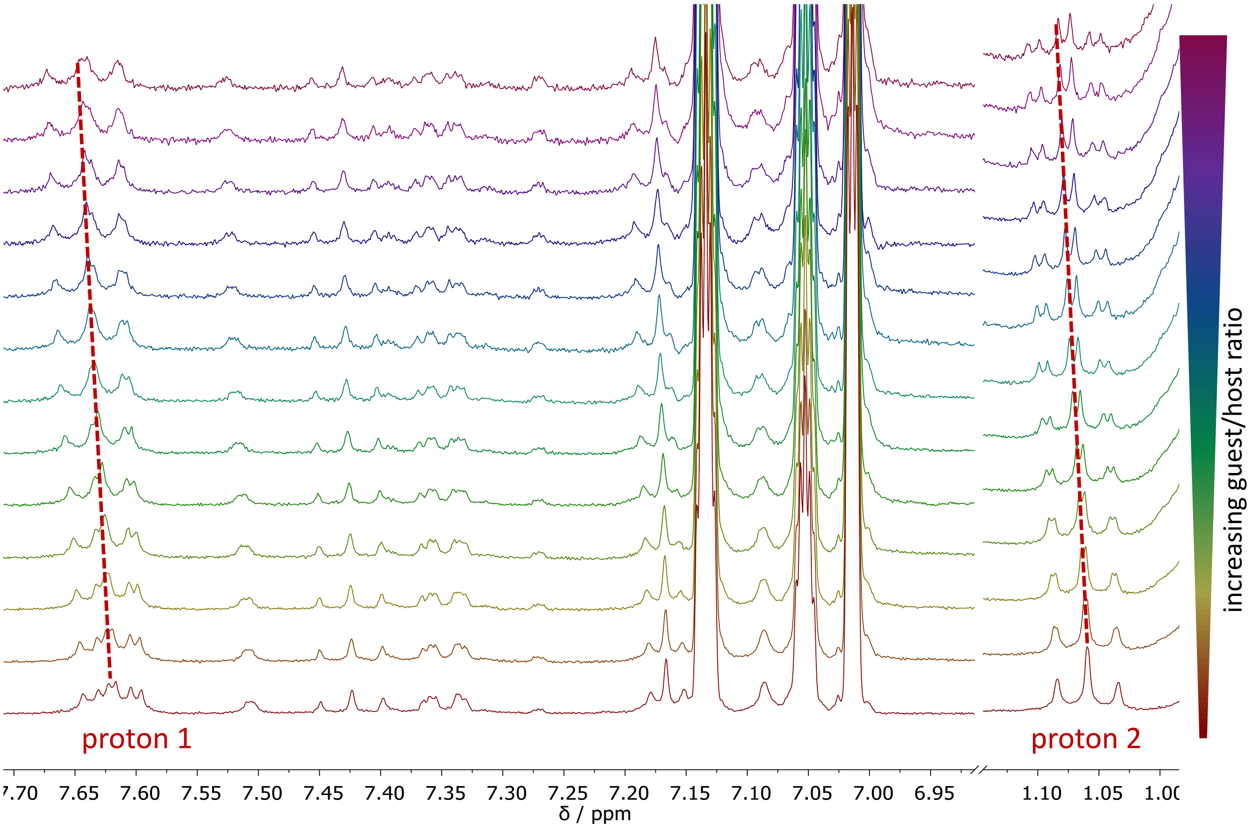

Figure S128. Selected regions of the ^{1}H NMR spectra (300 MHz, 300 K) during titration of **(+)-1** with C_{60} (toluene-d_8).

13. DFT Calculations

If not stated otherwise, all semi-empirical calculations were done with the xtb 6.3.1 program package[24,25], all DFT calculations with TURBOMOLE V.7.2,[26,27] the TURBOMOLE v7.3[28] or the Gaussian 09 program package.[29] All structural modifications were done with Avogadro[30]. Visualization and image generation of molecules were done with molden[31] and chimera[32]. The resolution-of-identity[33] (RI, RIJDX for SP) approximation for the Coulomb integrals was used in all DFT calculations employing matching auxiliary basis sets def2-XVP/J.[34] Further, the D3 dispersion correction scheme[35,36] with the Becke-Johnson damping function was applied.[37,38]

13.1 Calculation of the Ring Strain

Using TURBOMOLE, the geometries of all molecules were optimized without symmetry restrictions with the PBEh-3c[39] composite scheme. Single point calculations of were then performed on the optimized geometries at the B3LYP/def2-TZVP level of theory. The following homodesmotic equation was used to calculate the ring strain for **2-1**.

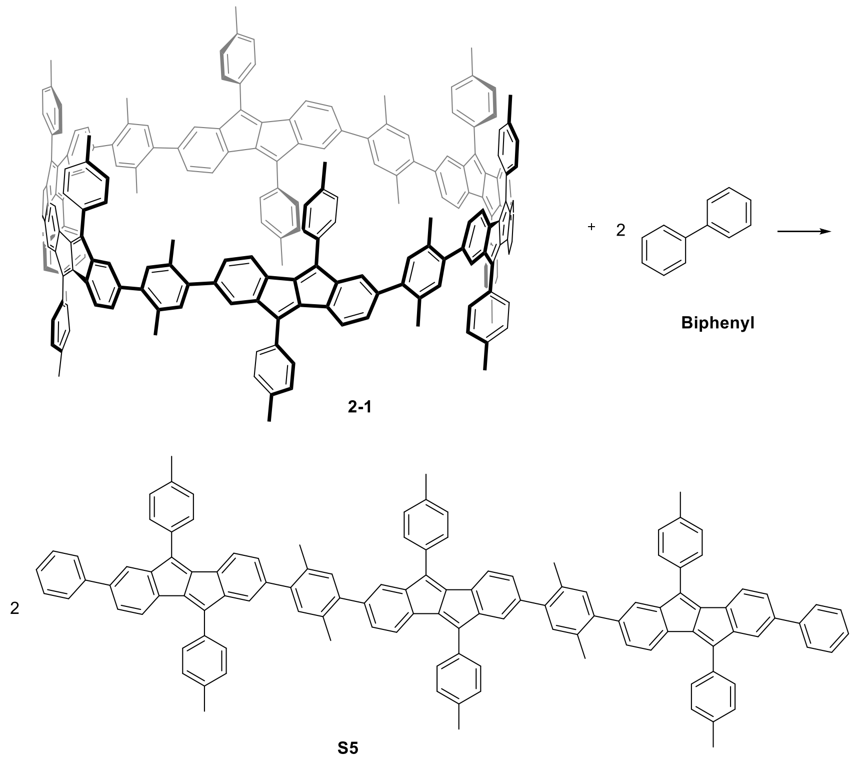

	$E_{tot,PBEh3c}$ (hartrees)	
**2-1**	−8152.35664	
**Biphenyl**	−462.26802	
**S5**	−4538.48202	
E_{Strain} (**2-1**)	0.07136	44.8 kcal mol^{-1}

Additionally, the ring strain was calculated with the recently published StrainViz scheme.[40] Figure S129 shows the visualized strain with a total value of 39.5 kcal mol^{-1} resembling the result of the homodesmotic equation above.

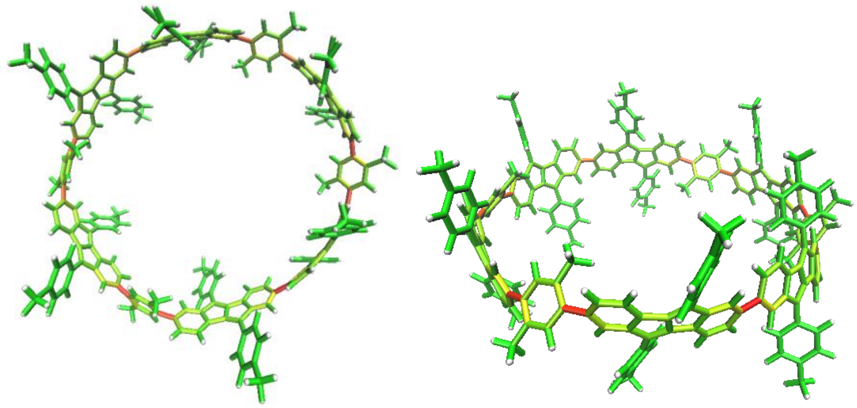

Figure S129. Outcome of the StrainViz calculation for **2-1** with a maximum value of 0.75 kcal mol^{-1} (red) strain per bond and a minimum value of 0.00 kcal mol^{-1} (green).

13.2 Calculation of the Nucleus Independent Chemical Shift (NICS) Values

NICS values were calculated using the GIAO (Gauge Including Atomic Orbitals)[41] method at the B3LYP/6-31G*[42] level of theory. In DFT calculations all alkyl chains were replaced by methyl groups. NICS(1)$_{iso}$ values were calculated by placing dummy atoms one angstrom above and below (inside and outside the cavity) the respective five and six-membered rings of the DBP units. Negative NICS values indicate a diatropic ring current (associated with an aromatic character) while positive NICS values indicate a paratropic ring current (associated with an antiaromatic character).

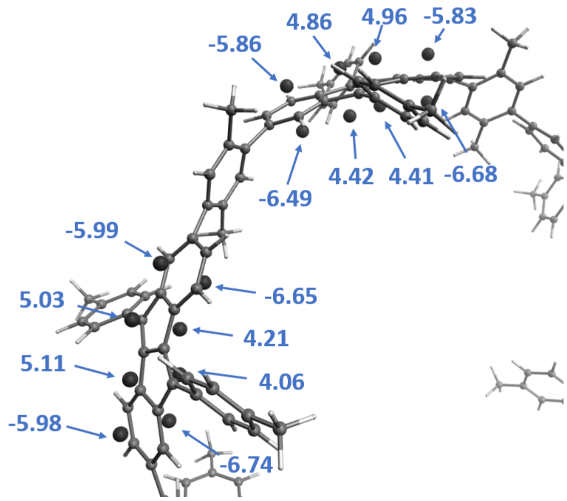

Figure S130. Representative part of the structure **2-1** to show calculated NICS(1)$_{iso}$ values.

13.3 Calculated Structure of U-Shaped Acyclic precursor (*R*,*R*)³-12

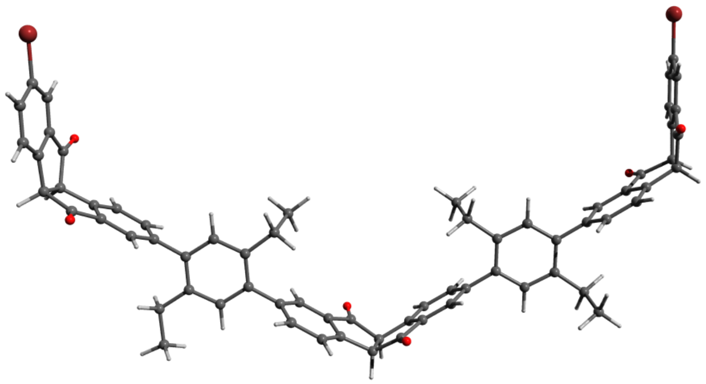

Figure S131. Force field (Avogadro) optimized geometry of **(*R*,*R*)³-12** showing the U-shaped conformation of the acyclic compound.

13.4 Conformational Energies

The relative energies of the 14 stereoisomers **1a**–**1n** were calculated. The Avogadro structures were taken as starting points with the hexyl-substituents roughly aligned by hand perpendicular to the hoop-plain. This procedure yields more similar and consistent structures for all isomers and a better comparability. The structures were then optimized on the GFN2-xTB[43] level with tight convergence criteria and applying the implicit solvation model GBSA[44] with toluene solvent. B97-3c[45] single-point energies were conducted on all optimized structures, applying the implicit solvent model D-COSMO-RS[46] with toluene solvent. All relative energies are given in kcal · mol^{-1} in Table S3 with the energetically lowest isomer set to zero of energy.

Table S3. Relative energies in kcal · mol⁻¹ at the GFN2-xTB and B97-3c//GFN2-xTB level of theory with reference to isomer **1c**.

isomer no.	GFN2-xTB	B97-3c//GFN2-xTB
**1a**	2.65	1.22
**1b**	5.82	4.20
**1c**	0.00	0.00
**1d**	5.43	2.98
**1e**	6.23	6.34
**1f**	5.68	5.35
**1g**	1.96	2.78
**1h**	3.43	3.24
**1i**	2.47	3.16
**1j**	4.98	5.25
**1k**	7.38	7.35
**1l**	7.11	6.75
**1m**	2.63	3.69
**1n**	7.04	8.98

The correlation between GFN2-xTB energies and B97-3c//GFN2-xTB energies is 92.3% and their mean absolute deviation amounts to only 0.67 kcal · mol⁻¹.

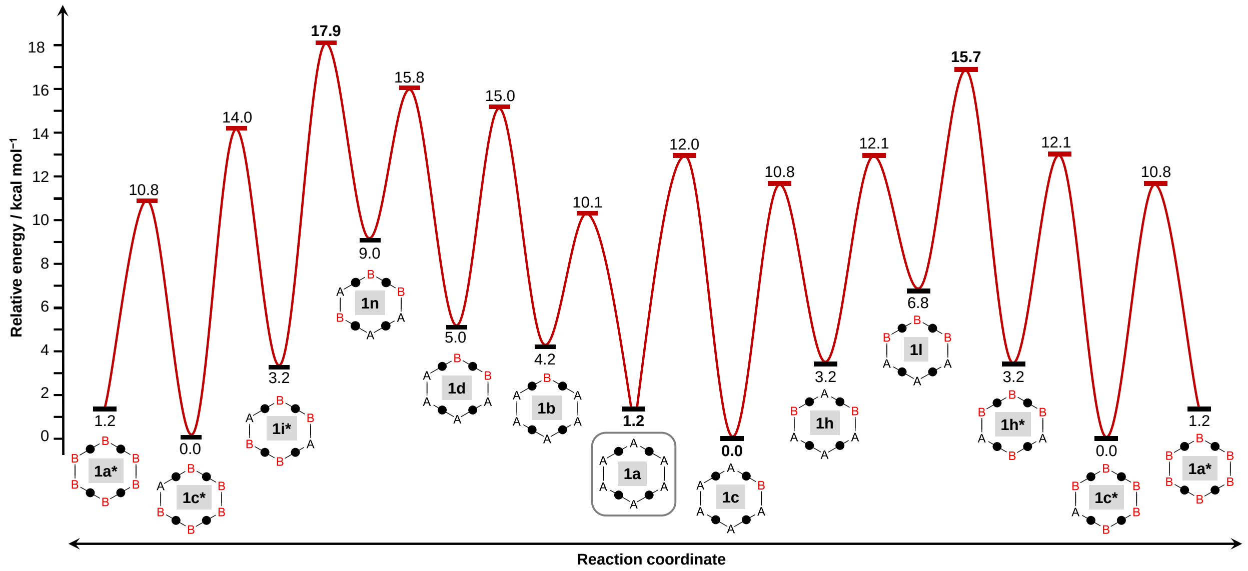

Figure S132. Possible racemization pathway of **1a** to its enantiomer **1a*** with interconversion barriers between conformational isomers derived from B97-3c(toluene)//GFN2-xTB(gas phase) transition state calculations conducted for **2-2a** to **2-2b** respective **2-2c.**

13.5 Rotational Barriers of the DBP Units in Hoop 1

A model system was generated to better examine the rotational barriers of the π-fragments in the hoop. Two cut-outs of the ring structure were made, consisting of three subunits. First, a planar H-DBP-DBP-phenyl-H strand and secondly a planar H-phenyl-DBP-phenyl-H strand. The outer units were aligned perpendicular to each other to separate both reaction barriers. These two systems are shown in Figure S133.

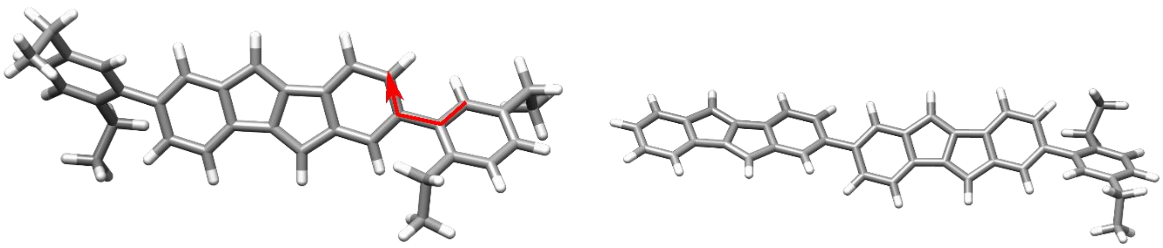

Figure S133. a) H-phenyl-DBP-phenyl-H, b) H-DBP-DBP-phenyl-H. Used model systems for investigation of the rotational barriers.

A relaxed double dihedral scan for the rotation around the two unit-interconnecting bonds in steps of 5° between −90° and 125°, respective dihedral angle denoted by the red arrow in Figure S133a), was conducted on GFN2-xTB level using ORCA 4.2.1[47,48]. As the implicit solvent model GBSA is not implemented in ORCA the calculations were done in the gas-phase. The system was allowed to relax for 25 optimization cycles applying tight convergence and otherwise default criteria except for both scanned dihedral angles that were kept fixed. Single-point energy calculations on the B97-3c level were conducted on these structures using ORCA 4.2.1, CPCM[49] for toluene and tight SCF convergence criteria. The resulting potential energy surface of rotation is depicted in Figure S134.

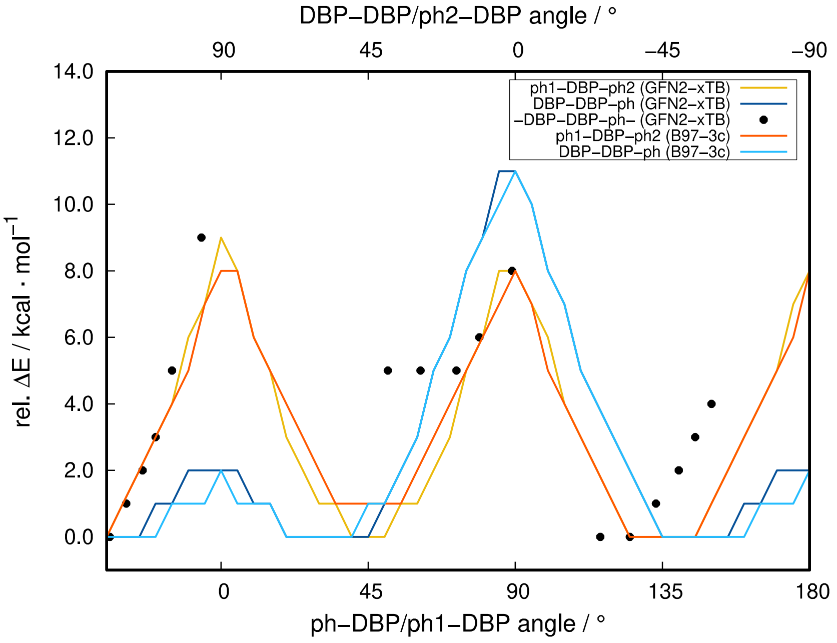

Figure S134. Relative energies in kcal mol^{-1} for rotation of the central unit of both model systems on GFN2-xTB and B97-3c//GFN2-xTB level of theory. The x1- and x2-axis are simultaneously valid and shifted against each other by 90°.

Next a hoop model compound **2-2** was calculated for hoop 1c, in which the hexyl-phenyl substituents on the DBP units were removed for simplicity. The black dots in Figure S134 arise from an examination of **2-2** for a -DBP-DBP-phenyl- unit using a relaxed dihedral scan on the GFN-FF level and subsequent reoptimization of this trajectory on the GFN2-xTB/GBSA(toluene) level keeping the scanned dihedral angles fixed. The end group angle varies between 90° and 45° so the x2-axis is not valid for these values. The jumps at 60° and 30° are due to the chosen method of trajectory generation but the missing values should well be represented by the shape of the yellow curve. Slight movements on the x1-axis are presumably due to the different end group angles and allowed relaxation for the rest of the loop. The energy maxima are always observed at dihedral angles of 0° between the central DBP unit and any outer unit. This is exemplary shown for both model systems in Figure S135.

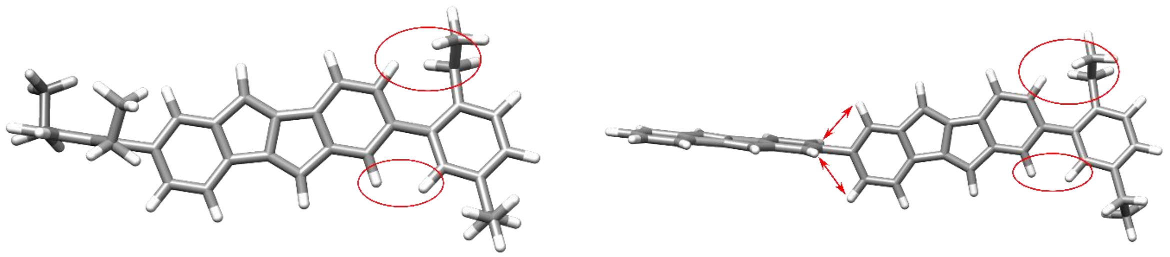

Figure S135. Steric hindrance of rotating the central DBP unit in the used model systems are indicated by red circles (repulsion of H and ethyl and H and H) and by red arrows (H-H repulsion in the second, not here depicted, other transition state).

The respective energy values are given in Table S4. The DBP-phenyl rotation will repeat all 90° due to symmetrical end-groups of the model system. The DBP-DBP rotation will only repeat all 180°. For H-DBP-DBP-phenyl-H two different barriers can be spotted because two different end-groups are present. The difference between both barriers is the hypothetically decoupled one for only DBP-phenyl rotation like in H-phenyl-DBP-phenyl-H.

Table S4. Rotational barriers in kcal · mol^{-1} for different systems on different levels of theory.

	**GFN2-xTB(gas)**	**B97-3c(toluene)//GFN2-xTB(gas)**
H-ph-DBP-ph-H	9.0	8.2
-ph-DBP-ph- (full TS opt.)	7.8	8.9
H-DBP-DBP-ph-H	12.0	11.5
-DBP-DBP-ph- (full TS opt.)	8.9	10.8

Rotational barriers for the hoop **2-2** were calculated by performing a transition state search and full optimization on GFN2-xTB gas phase level using the TURBOMOLE V.7.2 module statpt and initial structures taken from previously performed double dihedral scans on the same level. On the final transition states B97-3c single-point energies were calculated with ORCA 4.2.1, CPCM toluene solvent and tight SCF convergence criteria. The results are also given in Table S4.

All used approaches yield very similar rotational barrier heights and structures of the transition states. At the highest theoretical level, rotational barriers of 10.8 kcal · mol^{-1} for the -DBP-DBP-phenyl- unit and 8.9 kcal · mol^{-1} for the -phenyl-DBP-phenyl- unit are computed. However, the hexylphenyl substituents on all DBP units in hoop **1** were not included in the calculation, and hence real barriers are likely higher.

13.6 Association Free Energies ΔG for the Fullerene Complexation by 1

The free association energies ΔG for the stepwise association of two C_{60} fullerenes into isomer **1c** were computed. Structures were optimized with GFN2-xTB as described above. Harmonic vibrational frequencies for the thermostatistical rigid-rotor-harmonic-oscillator (RRHO) contributions were calculated with GFN-FF[7], SP energies with B97-3c/D-COSMO-RS(toluene). The final ΔG values consist of

$$\Delta G = \Delta G_{RRHO}(GFF) + \Delta E(method) + \Delta\delta G_{solv}(method) \quad (1)$$

where ΔE is the gas phase association energy and δG_{solv} is the solvation free energy for each species in the association reaction. The results are shown in Table S5.

Table S5. Association free energies ΔG in kcal · mol^{-1} for encapsulation of one and two C_{60} molecules in isomer **1c** on the GFN2-xTB and B97-3c//GFN2-xTB level of theory.

**association**	**GFN2-xTB**	**B97-3c//GFN2-xTB**	**experimental**
1st	−5.3	−8.4	−5.1
2nd	−6.5	−14.8	−2.8

From the optimized structures and the results of MD runs (see below) one could expect that the second association is slightly more favored than the first because both fullerenes fit perfectly into the hoop and do not interfere. The only structural difference is a slight deformation from the originally circle-shaped hoop to an ellipsoid-shaped hoop by the second guest. This is exemplarily shown in Figure 6b of the main part. The first association energy is very well described by the semi-empirical GFN2-xTB method while at both theoretical levels, the second one is underestimated (overbound) compared to experiment.

MD simulations of 100 ps length on the GFN2-xTB/GBSA(toluene) level at room temperature were performed on all three systems. Trajectories are available upon request. They show that the guest molecules are stable inside the hoop and make significant large amplitude motions without much interference of deformation of the host structure.

13.7 Cartesian Coordinates of Calculated Structures

The cartesian coordinates of selected calculated structures are found below. Those for all stereoisomers of hoop **1** can be found in a separate file.

Table S6. Cartesian coordinates of **S5** (PBEh-3c/def2-mSVP)

	X	Y	Z
C	-3.4592788	3.1075552	17.4646058
C	-3.3376461	1.9416085	18.2152912
C	-3.0666495	0.733176	17.5564664
C	-2.9329223	0.7181775	16.1856933
C	-3.0303557	1.9153856	15.4384396
C	-3.3014177	3.1054885	16.0804229
C	-2.8100462	1.544785	14.0505041
C	-2.6012921	0.1068811	14.0143482
C	-2.7931303	2.0531894	12.7938961
C	-2.4583771	-0.2753005	12.6189854
C	-2.6676659	-0.4091991	15.265791
C	-2.2646479	-1.4779257	11.9720155
C	-2.1676085	-1.4902742	10.5818978
C	-2.2463734	-0.3189544	9.8359447
C	-2.4428579	0.9009031	10.4981348
C	-2.56755	0.9197447	11.8708636
C	-2.6238901	0.0135136	3.2952017
C	-1.5411142	-0.2799879	4.1178695
C	-0.313417	-0.6203798	3.5335074
C	-0.1910264	-0.6308407	2.1616233
C	-1.2993702	-0.32924	1.335876
C	-2.5149618	-0.0139511	1.9053144
C	-0.8361453	-0.485464	-0.0335588
C	0.5530389	-0.9099048	0.0244821
C	-1.2468238	-0.3995121	-1.3225089
C	1.0165763	-1.0636345	-1.344742
C	0.9645561	-0.9932209	1.3132677
C	2.2333571	-1.3806923	-1.9117721
C	2.3457822	-1.4091634	-3.3003291
C	1.2647673	-1.1156055	-4.1250084
C	0.0356962	-0.7738904	-3.5438007
C	-0.0901509	-0.7603401	-2.1710992
C	1.099414	-1.9348916	-10.631426
C	1.7548193	-0.9776595	-9.8639114
C	2.5720196	-0.0339356	-10.5014588
C	2.713276	-0.0695369	-11.8713546
C	2.0148362	-1.0275669	-12.6433183
C	1.2163303	-1.9632422	-12.020607
C	2.362502	-0.7602533	-14.0295001
C	3.2862891	0.3620592	-14.0359178
C	2.1800752	-1.2253346	-15.2894121
C	3.6935385	0.5797024	-15.4139207
C	3.5120518	0.7914519	-12.7701212
C	4.544188	1.4728281	-16.0310915
C	4.704008	1.4104236	-17.4135303
C	4.0160283	0.4794302	-18.1867738
C	3.1572893	-0.4308067	-17.5531715
C	3.0119262	-0.3916378	-16.1838001
C	-2.0945438	-0.3360398	8.3644019
C	-1.1028698	0.4526517	7.7877495
C	-0.8763603	0.5043963	6.4190554
C	-1.6965462	-0.2687376	5.5885151
C	-2.7038474	-1.0378385	6.1636208
C	-2.9254322	-1.0957761	7.5335798
C	1.4031717	-1.1165354	-5.5979566
C	1.0647766	0.0365056	-6.3009123
C	1.1622254	0.1382795	-7.6822332
C	1.6153981	-0.9803469	-8.3911928
C	1.9293051	-2.1413612	-7.6909374
C	1.837595	-2.2410466	-6.308647
C	0.7588677	1.4173137	-8.3600978
C	2.1793599	-3.5419368	-5.6384059
C	-4.0477512	-1.9422681	8.0644927
C	0.2087211	1.3966185	5.8846579
C	-3.4901498	1.9697441	19.682058
C	4.3646472	1.89116	-12.3294653
C	-2.9537783	3.4444063	12.3828021
C	-2.5052514	-1.800695	15.6760228
C	2.275588	-1.3824135	1.8228322
C	5.2643893	1.7321258	-11.2734683
C	6.0802518	2.775773	-10.8771138
C	6.0227683	4.0173827	-11.5069193
C	5.120366	4.1752943	-12.553047
C	4.304571	3.1320549	-12.9598826
C	6.9037395	5.1446142	-11.0560726
C	2.9294072	-2.5072341	1.3237083
C	4.1788347	-2.8703467	1.7977522
C	4.8191404	-2.1267622	2.7838981
C	4.1641454	-1.0025955	3.2809929
C	2.9131631	-0.6379662	2.8167965
C	6.1607608	-2.5325866	3.3179096
C	-1.4415012	-2.5613373	15.1958035
C	-1.2882686	-3.8855711	15.5731387
C	-2.1891568	-4.4960572	16.4387033
C	-3.2511093	-3.7326942	16.9194776
C	-3.4066668	-2.4086894	16.5522258
C	-2.0332168	-5.9311528	16.8465422
C	-3.8120356	3.797912	11.3402877
C	-3.9714281	5.1222711	10.9741237
C	-3.2742085	6.1396533	11.6212684
C	-2.4147489	5.7839914	12.6557466
C	-2.2553869	4.4608155	13.0320483
C	-3.4304138	7.569093	11.1944671
C	4.1861079	0.4443651	-19.6512617
C	1.3356666	-2.3321066	-15.7281903
C	-2.5552891	0.0040211	-1.8280627
C	-3.2035482	-0.7286959	-2.8242925
C	-4.4506173	-0.3472643	-3.2841132
C	-5.0922508	0.7819935	-2.7797464
C	-4.4409953	1.5142309	-1.7930289
C	-3.1944574	1.1341951	-1.3231091
C	1.8178021	-3.3091142	-16.6011975
C	1.0152231	-4.3645883	-16.9940128
C	-0.2983039	-4.4796041	-16.5441436
C	-0.7780624	-3.5015927	-15.6795882
C	0.0225632	-2.4454472	-15.2766195
C	4.2854014	-0.7699076	-20.3303607
C	4.447355	-0.8049093	-21.7058116
C	4.5133054	0.3753437	-22.4314345

C	4.4145844	1.5899349	-21.7689421
C	4.2516301	1.6234132	-20.3935036
C	-4.4764255	2.7502092	20.2851628
C	-4.6181959	2.7777174	21.6630571
C	-3.7775011	2.0222958	22.4670742
C	-2.7940255	1.239782	21.8802435
C	-2.6520944	1.2143112	20.5023191
H	-5.1568795	3.321911	19.666602
H	-3.888393	2.0427903	23.5431216
H	-1.8634575	0.619862	20.0580065
H	4.2611422	-1.6973637	-19.771844
H	4.6398695	0.3487556	-23.5056016
H	4.1475633	2.5765239	-19.8903545
C	-1.1663674	-5.6156448	-16.99789
C	-6.4379686	1.1929132	-3.2997151
H	-3.6479679	4.0459791	17.9702164
H	-2.9836418	-0.1828743	18.1283328
H	-3.3880307	4.0305636	15.5262469
H	-2.1854347	-2.4030385	12.527394
H	-1.9995198	-2.4306358	10.0730563
H	-2.5029847	1.8152316	9.9203861
H	-3.5708556	0.2791848	3.7480064
H	0.5185625	-0.9065072	4.1646183
H	-3.3786855	0.2039285	1.2912232
H	3.0984296	-1.5867491	-1.2954875
H	3.3046877	-1.6376282	-3.7472653
H	-0.8025876	-0.5235144	-4.1825402
H	0.4660239	-2.6605611	-10.1369275
H	3.0961933	0.7061705	-9.9100898
H	0.68438	-2.7116954	-12.5928101
H	5.093048	2.2072353	-15.4567287
H	5.3960054	2.0901871	-17.8942645
H	2.5885305	-1.1353015	-18.1477222
H	-0.4741202	1.0464741	8.4421424
H	-3.3369219	-1.6263675	5.5087962
H	0.7173764	0.8969179	-5.7394996
H	2.2694186	-3.0037635	-8.2536658
H	1.6172715	2.0580375	-8.5705078
H	0.0809366	1.9902895	-7.7281349
H	0.2580865	1.2350069	-9.3102306
H	3.2018454	-3.5491113	-5.256254
H	2.0967458	-4.3703087	-6.3415915
H	1.523044	-3.7509269	-4.7944702
H	-3.7014807	-2.9288521	8.3782618
H	-4.8063397	-2.100441	7.2983706
H	-4.5314056	-1.4857712	8.9271109
H	0.447244	2.1830924	6.6001019
H	-0.0781325	1.8719677	4.9471855
H	1.133304	0.8466914	5.6961271
H	5.3466389	0.7724269	-10.7792057
H	6.7795046	2.6213294	-10.0636904
H	5.0475079	5.1320945	-13.0560341
H	3.5943351	3.2851542	-13.7629022
H	6.8048886	6.0156352	-11.7023019
H	7.954544	4.8520786	-11.0541346
H	6.6555617	5.4590993	-10.0409584
H	2.4415825	-3.1137394	0.5708889
H	4.6616884	-3.7533701	1.3964378
H	4.643559	-0.3984222	4.042232
H	2.4373835	0.253557	3.2063488
H	6.0640967	-3.0301767	4.2849082
H	6.8120216	-1.6705142	3.4628733
H	6.6690695	-3.2233055	2.6463554
H	-0.7149339	-2.1011139	14.5377947
H	-0.4467761	-4.4511985	15.1913931
H	-3.9720064	-4.1836912	17.5913293
H	-4.2533862	-1.8466791	16.9255733
H	-2.8799303	-6.5322011	16.5110588
H	-1.9765646	-6.0329011	17.9312082
H	-1.1304942	-6.3726518	16.4267911
H	-4.3838995	3.0312699	10.8331221
H	-4.655632	5.3706682	10.1713603
H	-1.8547293	6.553397	13.1737748
H	-1.565102	4.2061251	13.8266217
H	-4.4730622	7.8154143	10.9930232
H	-2.8692407	7.7692832	10.2795921
H	-3.0676764	8.2584131	11.9558442
H	-2.7382774	-1.6221621	-3.2209958
H	-4.937318	-0.941263	-4.0488043
H	-4.9117555	2.4020319	-1.3880586
H	-2.6976208	1.7327063	-0.5697828
H	2.8418679	-3.2577555	-16.9489458
H	1.4190222	-5.1177665	-17.6604293
H	-1.797819	-3.561208	-15.3185224
H	-0.3795784	-1.6847589	-14.6191748
H	4.53093	-1.757929	-22.2116531
H	4.4549632	2.5165749	-22.3264584
H	-5.3950061	3.3845092	22.1097148
H	-2.1276278	0.6523072	22.4982523
H	-1.444605	-5.5040271	-18.047482
H	-2.0879993	-5.6731606	-16.420452
H	-0.6541746	-6.5734048	-16.9007463
H	-6.8438434	2.0363939	-2.7430491
H	-7.1573212	0.3757327	-3.2325095
H	-6.3833596	1.4879318	-4.3489915

Table S7. Cartesian Coordinates of **2-1** (PBEh-3c)

	X	Y	Z
C	12.9177117	0.4156209	1.6079474
C	12.609402	-0.071994	0.3406451
C	12.2128377	0.8332224	-0.6555646
C	12.0412931	2.1626454	-0.3423313
C	12.3113797	2.6374885	0.9631662
C	12.7835835	1.7665823	1.9238717
C	11.8678098	4.0228911	0.9878348
C	11.3612378	4.3366716	-0.3369866
C	11.5445794	5.0136404	1.8569664
C	10.6801543	5.6163206	-0.2515944
C	11.4633489	3.257124	-1.1495695
C	9.9401913	6.3562691	-1.1485715
C	9.2268325	7.4578875	-0.6828057
C	9.2659878	7.8463943	0.6543332
C	10.096279	7.143677	1.5400869
C	10.7684641	6.0221265	1.1005634
C	4.5151771	11.8463168	3.0078985
C	4.9743364	11.4671176	1.7497815
C	4.1233506	11.6133182	0.6458901
C	2.8288524	12.0434607	0.8345085
C	2.3384531	12.3157319	2.1321377
C	3.2009894	12.2688963	3.2088299
C	0.8990966	12.4839122	1.9872806
C	0.5895845	12.362036	0.5693363

C	-0.2467387	12.5850699	2.7056223
C	-0.8561458	12.416696	0.4327278
C	1.7184782	12.1126272	-0.136952
C	-1.7207158	12.2947564	-0.6350544
C	-3.0831834	12.1452744	-0.3878776
C	-3.5925829	12.1156566	0.9080343
C	-2.7200523	12.3489878	1.9819929
C	-1.3704784	12.5040604	1.7459122
C	-9.3090139	8.9763225	2.4960336
C	-8.5310738	9.2763778	1.3832011
C	-8.7320939	8.5579128	0.1972118
C	-9.6293469	7.5119369	0.1770888
C	-10.3437003	7.15333	1.3427376
C	-10.2165722	7.9165677	2.4860291
C	-10.9639377	5.8689287	1.0496514
C	-10.6059535	5.5144765	-0.3148289
C	-11.5567733	4.8162017	1.6656722
C	-11.0596195	4.1541371	-0.5411682
C	-9.8349562	6.4843092	-0.8639732
C	-10.9926047	3.2935673	-1.6157713
C	-11.3518055	1.9611334	-1.4365945
C	-11.8046912	1.4796213	-0.2097203
C	-11.9879253	2.3923059	0.8431128
C	-11.5963716	3.7043019	0.6883415
C	8.3223578	8.8918659	1.1087441
C	8.2126953	10.0836855	0.3944636
C	7.1944406	11.0062446	0.6028831
C	6.238976	10.7179369	1.5822343
C	6.3987499	9.5707436	2.3546629
C	7.4128386	8.647121	2.1483327
C	-4.9637526	11.6148386	1.1517045
C	-5.0895413	10.5644116	2.0607017
C	-6.258682	9.841335	2.230375
C	-7.3631455	10.1865107	1.4394786
C	-7.2662026	11.2836087	0.5916362
C	-6.0948012	12.0198627	0.4352496
C	-6.2695917	8.6685204	3.1694429
C	-6.087483	13.1960628	-0.5009434
C	7.4212608	7.396161	2.9818977
C	7.1379398	12.2540756	-0.232921
C	12.8551912	-2.0593639	-1.1698538
C	12.5073032	-1.5173094	0.0655762
C	11.8812801	-2.3413216	1.0123304
C	11.5465455	-3.6342285	0.6779123
C	11.8058341	-4.1384904	-0.6170831
C	12.5079663	-3.363435	-1.5190138
C	11.111765	-5.4162924	-0.6951727
C	10.4942751	-5.6515261	0.6023214
C	10.7504983	-6.3933407	-1.5643165
C	9.7163492	-6.8741453	0.5004495
C	10.7464415	-4.6159094	1.4379692
C	8.8857404	-7.5513861	1.3681819
C	8.0938087	-8.5841448	0.872688
C	8.1213303	-8.9491478	-0.4716999
C	9.0489762	-8.3299872	-1.3213542
C	9.8377521	-7.3061192	-0.8395587
C	2.9782396	-12.2821238	-3.0237478
C	3.4572922	-12.0027195	-1.7470011
C	2.5749695	-12.0796102	-0.6608417
C	1.241112	-12.340552	-0.8824525
C	0.745005	-12.5082261	-2.1954326
C	1.6261527	-12.5332197	-3.2576631

C	-0.7063771	-12.499996	-2.0772838
C	-1.0251883	-12.3842067	-0.6607755
C	-1.8416742	-12.4338919	-2.8158174
C	-2.4688241	-12.2595924	-0.5480683
C	0.1127068	-12.3010538	0.0698531
C	-3.3320804	-12.0605275	0.5091939
C	-4.659218	-11.729721	0.2455708
C	-5.1348898	-11.5957212	-1.0565264
C	-4.2782061	-11.9077333	-2.1233541
C	-2.9643639	-12.2408711	-1.8712426
C	-10.3772773	-7.7336038	-2.6779517
C	-9.6577013	-8.1324737	-1.557084
C	-9.7764276	-7.3948675	-0.3719534
C	-10.5301981	-6.2409851	-0.3597901
C	-11.1794425	-5.7925569	-1.5324184
C	-11.1398571	-6.5651403	-2.6758065
C	-11.6304199	-4.4383063	-1.2442273
C	-12.0745207	-3.3174435	-1.8652632
C	-11.5176325	-2.7250108	0.3470953
C	-11.3503346	-1.8800424	1.4231545
C	-11.52965	-0.5120983	1.2412288
C	-11.9020446	0.0240013	0.009927
C	-12.1924581	-0.8574296	-1.0454294
C	-11.9781254	-2.2093778	-0.8875053
C	7.0723098	-9.8649946	-0.9722005
C	6.8108935	-11.056869	-0.2990136
C	5.6952379	-11.8459469	-0.5532918
C	4.7990625	-11.4159832	-1.5367741
C	5.1067509	-10.271879	-2.2683217
C	6.2179344	-9.4806546	-2.0165082
C	-6.4244091	-10.914451	-1.3084688
C	-6.393516	-9.834385	-2.190798
C	-7.4564907	-8.9637735	-2.364101
C	-8.6141867	-9.1837668	-1.6054813
C	-8.6777918	-10.302829	-0.7841007
C	-7.6136513	-11.1869426	-0.6243138
C	-7.2980965	-7.7769093	-3.2716535
C	-7.7780014	-12.3759178	0.2808371
C	6.3890162	-8.2109693	-2.8031365
C	5.4761633	-13.1037179	0.2398338
C	-9.2034266	6.5253914	-2.1804759
C	-12.029038	4.751882	3.0447708
C	-12.5221535	-3.1921309	-3.2483188
C	-10.0026997	-5.3170679	2.0026537
C	-1.9957768	-12.4569153	-4.267963
C	0.2760497	-12.1306909	1.5112926
C	11.0920834	-6.5365354	-2.9767957
C	10.2903435	-4.4072918	2.809814
C	11.8302303	5.0602245	3.2871028
C	11.0289202	3.1525402	-2.5391968
C	-0.4185051	12.6757092	4.1530599
C	1.8790454	11.8747371	-1.5691041
C	-10.6108032	-5.194948	0.6801561
C	-11.2431451	-4.1330386	0.1241316
C	-8.5022938	5.4299568	-2.6779873
C	-7.9034477	5.479556	-3.9264351
C	-7.9841757	6.6189792	-4.7194124
C	-8.6799378	7.7161333	-4.2159735
C	-9.2732272	7.6762737	-2.9675395
C	-7.3445188	6.6760657	-6.0751239
C	-11.7008942	3.6850615	3.8829766
C	-12.138498	3.6553327	5.1948225

C	-12.9239398	4.6796854	5.7178701
C	-13.2538732	5.7395681	4.879373
C	-12.814025	5.7786985	3.5668816
C	-13.3778754	4.6463519	7.1471073
C	0.2809075	11.8294763	5.0142622
C	0.1166259	11.9204066	6.3841081
C	-0.7491183	12.8564993	6.9463052
C	-1.4491666	13.694644	6.0857135
C	-1.2951485	13.6037746	4.7116834
C	1.0475918	10.9892559	-2.250041
C	1.2105505	10.7657049	-3.6076582
C	2.2039599	11.4166442	-4.3309527
C	3.0395213	12.2961733	-3.6457415
C	2.8874069	12.5182078	-2.2892608
C	2.3738278	11.1909442	-5.8042111
C	-0.9285265	12.9367453	8.433433
C	13.1120916	4.7715918	3.757276
C	13.391786	4.7881321	5.1117982
C	12.4055337	5.0912347	6.0474419
C	11.1267943	5.3720878	5.5772162
C	10.841287	5.3632763	4.2221512
C	12.7237307	5.1356131	7.5125529
C	11.3898268	4.1320601	-3.4620088
C	10.9669946	4.0578338	-4.7794253
C	10.1689522	3.0082134	-5.2203607
C	9.8063169	2.0315012	-4.2950042
C	10.2295458	2.0956667	-2.9802701
C	9.7061119	2.9208493	-6.6445815
C	11.1815686	-4.0012205	3.8042496
C	10.7509542	-3.811406	5.104913
C	9.4173124	-4.0030186	5.4585611
C	8.5295655	-4.3965045	4.4627961
C	8.9554896	-4.5958507	3.1598943
C	11.4968803	-7.7654869	-3.4940896
C	11.8271111	-7.8961302	-4.8335944
C	11.7528324	-6.8137945	-5.7030281
C	11.3334071	-5.5894675	-5.1859176
C	11.0093946	-5.4503578	-3.8491297
C	12.1134459	-6.9482473	-7.1528625
C	8.9557833	-3.7655849	6.865964
C	-12.0495082	-2.1791887	-4.0828549
C	-12.4647211	-2.096278	-5.4006853
C	-13.3715992	-3.0092446	-5.9308756
C	-13.8415211	-4.0197499	-5.0965513
C	-13.4260385	-4.1120382	-3.7798668
C	-9.1708797	-4.3181345	2.5082406
C	-8.5988349	-4.4408384	3.7615126
C	-8.8339854	-5.5612328	4.5552896
C	-9.6598152	-6.5579985	4.0465809
C	-10.2293419	-6.445321	2.7888517
C	-8.1897013	-5.6959036	5.9032406
C	-13.8505493	-2.8911469	-7.3472732
C	1.1796079	-12.9207859	2.2232837
C	1.3307783	-12.766012	3.5898686
C	0.6016701	-11.8098185	4.2922958
C	-0.2896312	-11.0157751	3.5775631
C	-0.4517256	-11.1707202	2.210983
C	-2.9711825	-13.253702	-4.870217
C	-3.1132344	-13.2789811	-6.2455931
C	-2.3005041	-12.5033319	-7.0695219
C	-1.3400917	-11.6989846	-6.4656444
C	-1.187129	-11.6746953	-5.0889376
C	-2.4593303	-12.5466306	-8.5605138
C	0.7972346	-11.6245999	5.7679705
H	-5.2592754	8.4349482	3.5032747
H	-6.6827939	7.7755066	2.7003052
H	-6.8670392	8.8624526	4.0611179
H	-7.0492903	13.7085819	-0.4788609
H	-5.9127753	12.8950309	-1.5362523
H	-5.3177044	13.9208753	-0.2412914
H	8.1795721	7.4327238	3.7667497
H	7.6151799	6.5050958	2.3849623
H	6.4599061	7.2616724	3.4765264
H	6.6672739	13.0792941	0.2996249
H	6.5732302	12.1013651	-1.1555118
H	8.1400516	12.5705057	-0.5219179
H	-7.5964001	-6.8490304	-2.7832189
H	-7.903341	-7.8671777	-4.1745038
H	-6.2609952	-7.671008	-3.587838
H	-7.5849903	-12.1252158	1.3262876
H	-7.1048386	-13.1890818	0.0143898
H	-8.7979698	-12.757133	0.2304717
H	6.6610915	-7.3676939	-2.1689235
H	5.4632603	-7.9551192	-3.317419
H	7.1636821	-8.2983734	-3.5666148
H	6.4282942	-13.5466787	0.5316157
H	4.9199125	-13.8495893	-0.3261833
H	4.9177857	-12.9139251	1.15938
H	-6.6467467	7.5111455	-6.1511223
H	-8.0910666	6.8081941	-6.8600023
H	-6.7921247	5.7642586	-6.2977449
H	-12.5733773	4.9465201	7.8215386
H	-13.6918089	3.645948	7.4451361
H	-14.2151363	5.3215036	7.3189519
H	3.4079564	10.9523639	-6.05528
H	2.1015426	12.0814169	-6.3736745
H	1.750321	10.371891	-6.1599281
H	-1.4535373	12.059248	8.8153058
H	-1.505693	13.8140537	8.7220715
H	0.0309102	12.987467	8.9493882
H	11.8231402	5.0719515	8.1220057
H	13.2299991	6.0664363	7.7758037
H	13.3816913	4.3172993	7.8053668
H	10.0520714	2.0013458	-7.11917
H	10.07412	3.7559231	-7.238953
H	8.6169101	2.9255523	-6.7085608
H	12.9935536	-6.3514494	-7.3982771
H	11.3031369	-6.6068809	-7.7984091
H	12.3338106	-7.9811497	-7.4184148
H	9.6356285	-4.2135741	7.5911249
H	7.9644319	-4.181916	7.0398209
H	8.9049621	-2.6979535	7.0880942
H	-8.6856611	-6.4502737	6.5126839
H	-8.2130016	-4.7558233	6.4547198
H	-7.1421347	-5.9890364	5.8099116
H	-14.7569977	-2.2849634	-7.4042374
H	-14.0861118	-3.8653309	-7.7748472
H	-13.1049015	-2.4188313	-7.9860664
H	-3.4973336	-12.3903224	-8.8563365
H	-2.1525485	-13.5141964	-8.9617592
H	-1.8573327	-11.7832771	-9.0514975
H	1.6805794	-11.0165381	5.9733559
H	0.9367241	-12.5781867	6.2771099
H	-0.0546901	-11.1245782	6.2271326

H	13.2500884	-0.2731354	2.3744988
H	11.9634451	0.4642542	-1.6422192
H	13.0059553	2.1102149	2.9254447
H	9.860018	6.0582611	-2.1855453
H	8.5742054	7.9888056	-1.3640333
H	10.1766954	7.4662129	2.569868
H	5.1819405	11.7675163	3.8575058
H	4.4556437	11.2964421	-0.3348686
H	2.8617011	12.5152052	4.2062712
H	-1.350756	12.2550643	-1.6508895
H	-3.7455034	11.9585808	-1.2227361
H	-3.1052773	12.3447412	2.9947576
H	-9.1711203	9.5452009	3.4066897
H	-8.1084054	8.7680886	-0.6633866
H	-10.7636362	7.6675105	3.385775
H	-10.6183164	3.626678	-2.5744943
H	-11.199294	1.2718104	-2.256616
H	-12.4130797	2.0538	1.7795682
H	8.9481315	10.2883312	-0.3761488
H	5.643747	9.3483201	3.0998855
H	-4.2070921	10.2455519	2.6030891
H	-8.1386828	11.5637928	0.0109807
H	13.3800722	-1.4441654	-1.8900875
H	11.5751031	-1.9285234	1.9658006
H	12.7599753	-3.7413787	-2.5010048
H	8.8026989	-7.2575402	2.405982
H	7.3811143	-9.0635165	1.5316165
H	9.1060746	-8.6230793	-2.3617298
H	3.664809	-12.2573058	-3.8607917
H	2.9255196	-11.8378739	0.3348582
H	1.2771949	-12.7024421	-4.2677545
H	-2.9805393	-12.0995587	1.5315774
H	-5.308363	-11.4846437	1.0756474
H	-4.6395212	-11.8242222	-3.1414868
H	-10.302397	-8.3144267	-3.5885124
H	-9.1949942	-7.6850139	0.4949203
H	-11.6393778	-6.2463127	-3.5811741
H	-11.0337734	-2.2587589	2.3857333
H	-11.2969954	0.1517127	2.0634262
H	-12.5595137	-0.4667265	-1.9861701
H	7.5020394	-11.3700228	0.4762415
H	4.3969277	-9.9384828	-3.0165042
H	-5.4655284	-9.6186152	-2.7072856
H	-9.5918694	-10.4820991	-0.2281227
H	-8.4039597	4.5398185	-2.0696811
H	-7.3554262	4.616647	-4.2853703
H	-8.7590814	8.6179212	-4.8118412
H	-9.8166394	8.5394948	-2.6039181
H	-11.0696018	2.886985	3.5135925
H	-11.8614264	2.8196732	5.8266519
H	-13.8731054	6.5454312	5.2548745
H	-13.1011351	6.6030925	2.9259751
H	0.9417646	11.0786916	4.5998267
H	0.6670414	11.2461459	7.0299553
H	-2.1265318	14.4346676	6.4945393
H	-1.8431305	14.2786949	4.066196
H	0.2831425	10.4507692	-1.7043759
H	0.556399	10.0636977	-4.1107425
H	3.8226804	12.8175366	-4.1837
H	3.5433119	13.2157818	-1.7831359
H	13.8984202	4.5509685	3.0459997
H	14.3978463	4.5664355	5.4481426
H	10.3357804	5.5957192	6.2830346
H	9.8289524	5.5554081	3.8901385
H	12.0257008	4.9492652	-3.1445849
H	11.2696682	4.8292235	-5.4773387
H	9.1750456	1.2078143	-4.6074573
H	9.9076448	1.3363302	-2.2793023
H	12.2266846	-3.8587283	3.5590379
H	11.4662085	-3.5105637	5.8614483
H	7.4841977	-4.5436778	4.7065023
H	8.2407877	-4.8800025	2.3979013
H	11.5780214	-8.6225913	-2.8373481
H	12.1506569	-8.8601065	-5.2072507
H	11.251937	-4.7315285	-5.8432347
H	10.6627597	-4.4950652	-3.4756308
H	-11.3200656	-1.4721665	-3.7087461
H	-12.0701486	-1.30861	-6.0314968
H	-14.5494758	-4.744429	-5.4809877
H	-13.8186761	-4.8953959	-3.143408
H	-8.9547918	-3.4483646	1.9009057
H	-7.9507218	-3.6530729	4.1274785
H	-9.864422	-7.4385034	4.6437431
H	-10.8779648	-7.2310436	2.4220418
H	1.7489459	-13.6815499	1.7036358
H	2.0268657	-13.4039032	4.1217625
H	-0.8639413	-10.2573997	4.0962759
H	-1.1347725	-10.5250797	1.6736231
H	-3.608875	-13.8752538	-4.2541424
H	-3.8711199	-13.9147367	-6.6881674
H	-0.7043693	-11.0730419	-7.0803729
H	-0.4487713	-11.0214605	-4.6413479

Table S8. Cartesian coordinates of Biphenyl (PBEh-3c).

	X	Y	Z
C	3.5638101	0.0000001	-0.0000902
C	2.8590657	1.1929638	0.0000336
C	1.4744534	1.1902693	0.0001124
C	0.7425493	0.0000002	0.0001289
C	1.4744533	-1.1902692	0.0000544
C	2.8590657	-1.1929636	-0.0000681
C	-3.5638101	-0.0000001	-0.0000838
C	-2.8590657	-1.1929638	0.0000272
C	-1.4744534	-1.1902693	0.0001025
C	-0.7425493	-0.0000002	0.0001202
C	-1.4744533	1.1902692	0.0000372
C	-2.8590657	1.1929636	-0.0000673
H	3.388255	2.1368607	0.0000791
H	0.9704829	2.1460181	0.000099
H	0.9704826	-2.1460179	0.000041
H	3.3882548	-2.1368606	-0.0001634
H	-3.388255	-2.1368607	0.0000612
H	-0.9704829	-2.1460181	0.0000852
H	-0.9704826	2.1460179	-0.0000057
H	-3.3882548	2.1368606	-0.0001481
H	4.6455837	0	-0.0001892
H	-4.6455837	0	-0.0001663

14. References

[1] D. B. G. Williams, M. Lawton, *J. Org. Chem.* **2010**, *75*, 8351–8354.
[2] H. E. Gottlieb, V. Kotlyar, A. Nudelman, *J. Org. Chem.* **1997**, *62*, 7512–7515.
[3] B. W. D'Andrade, S. Datta, S. R. Forrest, P. Djurovich, E. Polikarpov, M. E. Thompson, *Org. Electron.* **2005**, *6*, 11–20.
[4] M. Hermann, D. Wassy, D. Kratzert, B. Esser, *Chem. – A Eur. J.* **2018**, *24*, 7374–7387.
[5] D. C. Grenz, M. Schmidt, D. Kratzert, B. Esser, *J. Org. Chem.* **2018**, *83*, 656–663.
[6] J. Frey, C. Tock, J.-P. Collin, V. Heitz, J.-P. Sauvage, K. Rissanen, *J. Am. Chem. Soc.* **2008**, *130*, 11013–11022.
[7] T. Nagano, Z. Jia, X. Li, M. Yan, G. Lu, A. S. C. Chan, T. Hayashi, *Chem. Lett.* **2010**, *39*, 929–931.
[8] S.-H. Jang, H.-J. Kim, M.-J. Hwang, E.-B. Jeong, H.-J. Yun, D.-H. Lee, Y.-H. Kim, C.-E. Park, Y.-J. Yoon, S.-K. Kwon, et al., *Bull. Korean Chem. Soc.* **2012**, *33*, 541–548.
[9] A. Tota, M. Zenzola, S. J. Chawner, S. S. John-Campbell, C. Carlucci, G. Romanazzi, L. Degennaro, J. A. Bull, R. Luisi, *Chem. Commun.* **2017**, *53*, 348–351.
[10] J. Brandt, H.-J. Gais, *Tetrahedron: Asymmetry* **1997**, *8*, 909–912.
[11] C. S. Shiner, A. H. Berks, *J. Org. Chem.* **1988**, *53*, 5542–5545.
[12] E. R. Darzi, B. M. White, L. K. Loventhal, L. N. Zakharov, R. Jasti, *J. Am. Chem. Soc.* **2017**, *139*, 3106–3114.
[13] M. Peters, M. Trobe, R. Breinbauer, *Chem. Eur. J.* **2013**, *19*, 2450–2456.
[14] *SAINT V8.37A, Bruker AXS*, Madison, Wisconsin, USA, **2015**.
[15] L. Krause, R. Herbst-Irmer, G. M. Sheldrick, D. Stalke, *J. Appl. Crystallogr.* **2015**, *48*, 3–10.
[16] D. Kratzert, J. J. Holstein, I. Krossing, *J. Appl. Crystallogr.* **2015**, *48*, 933–938.
[17] C. B. Hübschle, G. M. Sheldrick, B. Dittrich, *J. Appl. Crystallogr.* **2011**, *44*, 1281–1284.
[18] L. J. Bourhis, O. V. Dolomanov, R. J. Gildea, J. A. K. Howard, H. Puschmann, *Acta Crystallogr. Sect. A Found. Adv.* **2015**, *71*, 59–75.
[19] G. M. Sheldrick, *Acta Crystallogr. Sect. A Found. Adv.* **2015**, *71*, 3–8.
[20] G. M. Sheldrick, *Acta Crystallogr. Sect. C Struct. Chem.* **2015**, *71*, 3–8.
[21] can be found under http://supramolecular.org, **2020**.
[22] P. Thordarson, *Chem. Soc. Rev.* **2011**, *40*, 1305–1323.
[23] D. Brynn Hibbert, P. Thordarson, *Chem. Commun.* **2016**, *52*, 12792–12805.
[24] S. Grimme, xtb 6.3.1; Mulliken Center for Theoretical Chemistry, University of Bonn; available upon request via e-mail to xtb@thch.uni-bonn.de, **2019**.
[25] C. Bannwarth, S. Ehlert, S. Grimme, *J. Chem. Theory Comput.* **2019**, *15*, 1652–1671.
[26] F. Furche, R. Ahlrichs, C. Hättig, W. Klopper, M. Sierka, F. Weigend, *Wiley Interdiscip. Rev. Comput. Mol. Sci.* **2014**, *4*, 91–100.
[27] R. Ahlrichs, M. Armbruster, M. Bär, et. al., TURBOMOLE 7.2 2017, Universität Karlsruhe. See also: http://www.turbomole.com, **2017**.
[28] TURBOMOLE V7.3, a development of the University of Karlsruhe and Forschungszentrum Karlsruhe GmbH, **2019**.
[29] Gaussian 09, Revision D.01, M. J. Frisch, G. W. Trucks, H. B. Schlegel, G. E. Scuseria, M. A. Robb, J. R. Cheeseman, G. Scalmani, V. Barone, G. A. Petersson, H. Nakatsuji, X. Li, M. Caricato, A. Marenich, J. Bloino, B. G. Janesko, R. Gomperts, B. Mennucci, H. P. Hratchian, J. V. Ortiz, A. F. Izmaylov, J. L. Sonnenberg, D. Williams-Young, F. Ding, F. Lipparini, F. Egidi, J. Goings, B. Peng, A. Petrone, T. Henderson, D. Ranasinghe, V. G. Zakrzewski, J. Gao, N. Rega, G. Zheng, W. Liang, M. Hada, M. Ehara, K. Toyota, R. Fukuda, J. Hasegawa, M. Ishida, T. Nakajima, Y. Honda, O. Kitao, H. Nakai, T. Vreven, K. Throssell, J. A. Montgomery, Jr., J. E. Peralta, F. Ogliaro, M. Bearpark, J. J. Heyd, E. Brothers, K. N. Kudin, V. N. Staroverov, T. Keith, R. Kobayashi, J. Normand, K. Raghavachari, A. Rendell, J. C. Burant, S. S. Iyengar, J. Tomasi, M. Cossi, J. M. Millam, M. Klene, C. Adamo, R. Cammi, J. W. Ochterski, R. L. Martin, K. Morokuma, O. Farkas, J. B. Foresman, and D. J. Fox, Gaussian, Inc., Wallingford CT, **2016**.
[30] M. D. Hanwell, D. E. Curtis, D. C. Lonie, T. Vandermeersch, E. Zurek, G. R. Hutchison, *J. Cheminform.* **2012**, *4*, 17.
[31] G. Schaftenaar, J. H. Noordik, *J. Comput. Aided. Mol. Des.* **2000**, *14*, 123–134.
[32] E. F. Pettersen, T. D. Goddard, C. C. Huang, G. S. Couch, D. M. Greenblatt, E. C. Meng, T. E. Ferrin, *J. Comput. Chem.* **2004**, *25*, 1605–1612.
[33] K. Eichkorn, O. Treutler, H. Öhm, M. Häser, R. Ahlrichs, *Chem. Phys. Lett.* **1995**, *240*, 283–290.
[34] F. Weigend, *Phys. Chem. Chem. Phys.* **2006**, *8*, 1057–1065.
[35] S. Grimme, J. Antony, S. Ehrlich, H. Krieg, *J. Chem. Phys.* **2010**, *132*, 154104.

[36] S. Grimme, S. Ehrlich, L. Goerigk, *J. Comput. Chem.* **2011**, *32*, 1456–1465.
[37] A. D. Becke, E. R. Johnson, *J. Chem. Phys.* **2005**, *123*, 154101.
[38] E. R. Johnson, A. D. Becke, *J. Chem. Phys.* **2006**, *124*, 174104.
[39] S. Grimme, J. G. Brandenburg, C. Bannwarth, A. Hansen, *J. Chem. Phys.* **2015**, *143*, 054107.
[40] C. E. Colwell, T. W. Price, T. Stauch, R. Jasti, *Chem. Sci.* **2020**, *11*, 3923–3930.
[41] M. Häser, R. Ahlrichs, H. P. Baron, P. Weis, H. Horn, *Theor. Chim. Acta* **1992**, *83*, 455–470.
[42] G. A. Petersson, A. Bennett, T. G. Tensfeldt, M. A. Al-Laham, W. A. Shirley, J. Mantzaris, *J. Chem. Phys.* **1988**, *89*, 2193–2218.
[43] C. Bannwarth, S. Ehlert, S. Grimme, *J. Chem. Theory Comput.* **2019**, *15*, 1652–1671.
[44] J. Zhu, Y. Shi, H. Liu, *J. Phys. Chem. B* **2002**, *106*, 4844–4853.
[45] J. G. Brandenburg, C. Bannwarth, A. Hansen, S. Grimme, *J. Chem. Phys.* **2018**, *148*, 064104.
[46] A. Klamt, *J. Phys. Chem.* **1995**, *99*, 2224–2235.
[47] F. Neese, et. al., ORCA 4.2.1 2017, Max-Planck-Institute for Chemical Energy Conversion. See also: https://cec.mpg.de/orcadownload/, **2017**.
[48] F. Neese, *Wiley Interdiscip. Rev. Comput. Mol. Sci.* **2018**, *8*, DOI 10.1002/wcms.1327.
[49] J. Tomasi, B. Mennucci, R. Cammi, *Chem. Rev.* **2005**, *105*, 2999–3094.
[50] C. R. Johnson, J. R. Zeller, *Tetrahedron* **1984**, *40*, 1225–1233.
