# Supplementary figures and images for: Chiral Dibenzopentalene‐Based Conjugated Nanohoops through Stereoselective Synthesis
Source: Angew Chem Int Ed Engl. 2021 Mar 23;60(19):10680–9. doi: 10.1002/anie.202016968 (PMC8252646; doi:10.1002/anie.202016968)

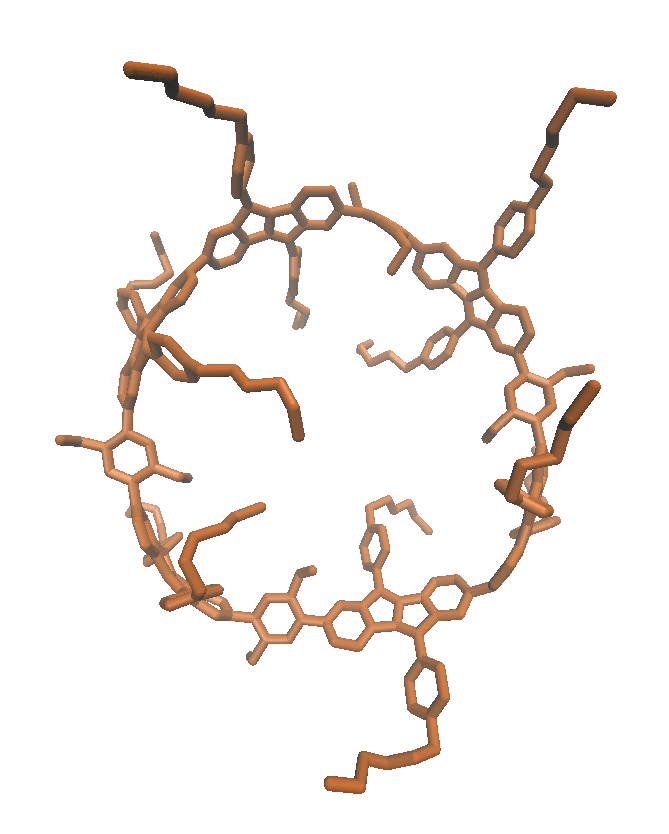

Supplement: Supplementary file 2 — Supplementary [file ANIE-60-10680-s001.gif]

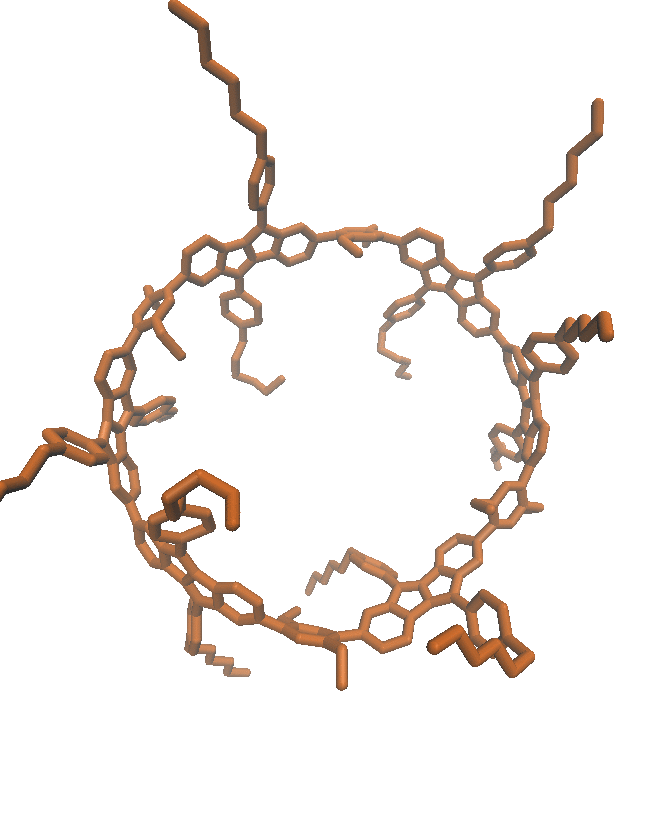

Supplement: Supplementary file 3 — Supplementary [file ANIE-60-10680-s004.gif]
