# Supplementary material for: Chiral Dibenzopentalene‐Based Conjugated Nanohoops through Stereoselective Synthesis
Source: Angew Chem Int Ed Engl. 2021 Mar 23;60(19):10680–9. doi: 10.1002/anie.202016968 (PMC8252646; doi:10.1002/anie.202016968)
Supplement: Supplementary file 4 — Supplementary [file ANIE-60-10680-s002.pdf]

Table 1 Cartesian coordinates of ***1a*** *(GFN2-xTB(GBSA(toluene)). B97-3c(COSMO(toluene) single point energy = 10838.55887 Hartree.*

	X	Y	Z
C	7.34783	0.31615	8.34607
C	8.31568	-0.66609	8.12613
C	9.61095	-0.26304	7.75705
C	9.86178	1.06419	7.48456
C	8.84851	2.04083	7.67997
C	7.60874	1.66489	8.15298
C	9.36369	3.28372	7.16005
C	10.70323	3.01684	6.65586
C	8.94644	4.52695	6.78080
C	11.10583	4.18104	5.89454
C	11.03437	1.70859	6.86928
C	12.19767	4.46136	5.09862
C	12.17273	5.59528	4.29102
C	11.08977	6.46610	4.29646
C	10.02741	6.23617	5.18203
C	10.01581	5.09308	5.94839
C	8.96585	11.10227	0.16139
C	9.91662	10.10004	0.00007
C	10.39691	9.79635	-1.27662
C	9.82654	10.41313	-2.37144
C	8.74739	11.31896	-2.20100
C	8.37459	11.72109	-0.93429
C	8.19160	11.53891	-3.51825
C	9.02901	10.81337	-4.46906
C	7.09224	12.04345	-4.14692
C	8.40187	10.93718	-5.76916
C	10.01552	10.14402	-3.80464
C	8.70037	10.45423	-7.02744
C	7.73719	10.54076	-8.02846
C	6.48603	11.09519	-7.78436
C	6.22829	11.69312	-6.54495
C	7.18472	11.63747	-5.55570
C	0.63919	10.18052	-10.60209
C	1.91134	9.79016	-11.00874
C	2.05868	8.91021	-12.08567
C	0.93343	8.33823	-12.64513
C	-0.35588	8.66837	-12.14733
C	-0.50300	9.62821	-11.16765
C	-1.28604	7.76474	-12.78475
C	-0.52543	6.96170	-13.73339
C	-2.53389	7.24210	-12.61275
C	-1.40529	5.91393	-14.20219
C	0.79903	7.29156	-13.66979
C	-1.26196	4.85955	-15.08178
C	-2.25620	3.89084	-15.14495
C	-3.39452	3.94349	-14.33785
C	-3.59071	5.08369	-13.54034
C	-2.61423	6.04886	-13.46726
C	10.89615	7.49316	3.25240
C	9.72431	7.35018	2.51046
C	9.39270	8.18938	1.46518
C	10.27437	9.22828	1.14369
C	11.43172	9.38769	1.88907
C	11.76547	8.53765	2.94066
C	5.34700	10.86978	-8.70021
C	4.33504	10.06795	-8.17644
C	3.21671	9.70607	-8.90285
C	3.09169	10.19090	-10.21186
C	4.09200	10.99938	-10.73186
C	5.22679	11.34539	-10.00372
C	2.23924	8.73682	-8.29414
C	6.27802	12.22117	-10.62975
C	13.03097	8.78589	3.71639
C	8.13113	7.92726	0.68792
C	8.56284	-2.95711	7.21173
C	7.91202	-2.07875	8.08131
C	6.75036	-2.52353	8.73453
C	6.21448	-3.75110	8.41455
C	6.84624	-4.59022	7.46016
C	8.05064	-4.20982	6.90446
C	5.90922	-5.64602	7.16018
C	4.70646	-5.39590	7.94665
C	5.68859	-6.63900	6.25069
C	3.70005	-6.32805	7.48159
C	4.88691	-4.29806	8.73717
C	2.37384	-6.53452	7.80321
C	1.60406	-7.36704	6.99585
C	2.15428	-8.02772	5.90305
C	3.52870	-7.91692	5.65317
C	4.28962	-7.05629	6.41199
C	-0.64381	-10.61362	0.53210
C	-1.15151	-9.83911	1.56843
C	-2.41789	-9.25731	1.45095
C	-3.09248	-9.35411	0.25108
C	-2.52506	-10.08307	-0.82873
C	-1.33076	-10.75443	-0.66832
C	-3.35936	-9.82554	-1.98107
C	-4.45305	-8.97288	-1.53804
C	-3.33378	-9.89840	-3.34345
C	-5.13843	-8.51068	-2.72655
C	-4.32723	-8.69757	-0.20636
C	-6.17867	-7.62827	-2.93302
C	-6.45740	-7.20852	-4.23034
C	-5.72564	-7.67749	-5.31453
C	-4.73087	-8.64225	-5.11571
C	-4.42475	-9.04423	-3.83422
C	-4.69034	-4.97809	-11.21758
C	-5.62415	-4.69706	-10.22546
C	-6.40782	-3.54210	-10.31458
C	-6.14622	-2.62806	-11.31524
C	-5.11554	-2.88050	-12.26046
C	-4.43112	-4.07812	-12.24382
C	-4.94959	-1.66251	-13.02091
C	-4.02498	-1.01947	-13.79112
C	-5.65349	0.55827	-13.14561
C	-6.23325	1.80874	-13.07455
C	-5.54099	2.90267	-13.57724
C	-4.26923	2.77924	-14.14110
C	-3.74904	1.48783	-14.33267
C	-4.42459	0.39478	-13.84129
C	1.30768	-8.64934	4.86466
C	1.46100	-8.10472	3.59049
C	0.69603	-8.49828	2.50971
C	-0.28591	-9.47527	2.71415
C	-0.42916	-10.03612	3.97386
C	0.35761	-9.65207	5.05644
C	-5.80092	-7.00730	-6.63132
C	-4.66696	-6.27797	-6.97671
C	-4.58187	-5.53417	-8.13925
C	-5.68274	-5.53157	-9.00533
C	-6.81140	-6.26652	-8.66794
C	-6.89591	-7.00586	-7.49308
C	-3.36018	-4.68457	-8.36634
C	-8.11603	-7.83648	-7.20716
C	0.21381	-10.38381	6.36289

C	0.89728	-7.81436	1.18427
C	1.88503	6.70647	-14.43919
C	-3.59579	7.73393	-11.74954
C	-2.86022	-1.60237	-14.43759
C	-7.77649	-0.66521	-10.79964
C	-2.39181	-10.63861	-4.16711
C	-5.22144	-7.89891	0.61606
C	6.63234	-7.16997	5.28146
C	3.96406	-3.65928	9.66257
C	7.67752	5.16074	7.09674
C	12.27856	1.04900	6.51062
C	5.96819	12.78197	-3.59322
C	11.04971	9.26582	-4.32739
C	-6.69453	-1.28366	-11.54853
C	-5.96669	-0.72624	-12.56138
C	3.06419	6.26959	-13.83731
C	4.07032	5.70201	-14.59551
C	3.94019	5.56466	-15.97230
C	2.76320	6.00133	-16.57204
C	1.74847	6.55765	-15.82066
C	5.01615	4.91393	-16.79127
C	-3.94189	9.08591	-11.77985
C	-4.96027	9.56965	-10.98392
C	-5.66068	8.72635	-10.12746
C	-5.30396	7.38374	-10.08545
C	-4.28857	6.89026	-10.88203
C	-6.80476	9.24860	-9.30886
C	5.33443	12.35185	-2.42779
C	4.25457	13.04564	-1.91941
C	3.77831	14.19048	-2.54970
C	4.41399	14.61995	-3.70900
C	5.48625	13.92400	-4.23222
C	10.75007	8.28755	-5.27488
C	11.73829	7.45338	-5.75908
C	13.05154	7.56794	-5.31687
C	13.34816	8.54212	-4.36982
C	12.36482	9.37502	-3.87443
C	2.63183	14.96485	-1.96824
C	7.19360	5.11888	8.40602
C	5.98722	5.70488	8.73035
C	5.21894	6.34337	7.76211
C	5.69741	6.37829	6.45773
C	6.90630	5.79796	6.12550
C	3.92386	7.00696	8.12812
C	13.49791	1.66408	6.80115
C	14.69028	1.05934	6.45992
C	14.70844	-0.17131	5.81112
C	13.49434	-0.77883	5.51417
C	12.29611	-0.18242	5.85675
C	16.01034	-0.83606	5.47235
C	2.64920	-3.38594	9.28857
C	1.78871	-2.75205	10.16338
C	2.20622	-2.38376	11.43733
C	3.51683	-2.66198	11.81051
C	4.38816	-3.28155	10.93684
C	7.93172	-7.48697	5.68216
C	8.84551	-7.98651	4.77676
C	8.49978	-8.17778	3.44287
C	7.20916	-7.85244	3.04252
C	6.28667	-7.35735	3.94368
C	9.48550	-8.75855	2.47195
C	1.28682	-1.64951	12.36883
C	-1.59197	-1.03179	-14.34025
C	-0.50860	-1.61865	-14.96650
C	-0.65308	-2.78601	-15.70650
C	-1.91880	-3.35597	-15.80083
C	-3.00474	-2.77928	-15.17510
C	-7.80322	-0.67822	-9.40543
C	-8.83077	-0.06247	-8.71831
C	-9.86456	0.57642	-9.39383
C	-9.83950	0.58489	-10.78413
C	-8.81016	-0.01854	-11.47861
C	-10.99806	1.20577	-8.63834
C	0.51380	-3.40578	-16.41820
C	-6.59873	-8.11628	0.54169
C	-7.47049	-7.38442	1.32167
C	-7.00185	-6.40671	2.19329
C	-5.63238	-6.17711	2.25231
C	-4.75199	-6.91219	1.48192
C	-2.10059	-11.96857	-3.85861
C	-1.19950	-12.68656	-4.61839
C	-0.55188	-12.10189	-5.70175
C	-0.83846	-10.77607	-6.00504
C	-1.74425	-10.05237	-5.25385
C	0.40146	-12.89739	-6.54418
C	-7.95961	-5.59039	3.01075
C	1.51289	-0.13565	12.26116
C	0.58966	0.65093	13.18835
C	0.84343	2.15471	13.10359
C	-0.08185	2.95069	14.02197
C	0.19028	4.44874	13.94363
C	-8.46201	-4.37942	2.21359
C	-9.47947	-3.56056	3.00444
C	-9.98415	-2.35414	2.21536
C	-11.02265	-1.54929	2.99463
C	-11.52242	-0.34599	2.20305
C	9.46414	-10.29175	2.52858
C	10.44283	-10.91283	1.53492
C	10.44677	-12.43829	1.61175
C	11.40916	-13.06407	0.60400
C	11.42189	-14.58570	0.69744
C	-8.09372	9.27070	-10.14114
C	-9.28505	9.79156	-9.34089
C	-10.55871	9.84303	-10.18252
C	-11.75816	10.35457	-9.38694
C	-13.01969	10.42121	-10.24055
C	4.70569	3.42749	-17.01111
C	5.77621	2.74050	-17.85547
C	5.45147	1.27007	-18.11029
C	6.52422	0.58136	-18.95196
C	6.17426	-0.87425	-19.23857
C	0.55629	-2.96488	-17.88722
C	1.69329	-3.63913	-18.65151
C	1.72018	-3.22669	-20.12191
C	2.82793	-3.93503	-20.89971
C	2.86564	-3.50120	-22.36084
C	-12.09350	0.17362	-8.34074
C	-13.24531	0.78119	-7.54378
C	-14.33527	-0.24366	-7.23679
C	-15.48735	0.36302	-6.43797
C	-16.56949	-0.66498	-6.12772
C	16.57124	-1.58964	6.68539
C	17.90303	-2.26559	6.36819
C	18.48185	-2.99226	7.58050
C	19.81815	-3.66245	7.26626
C	20.39751	-4.37695	8.48198
C	-0.34805	-13.64354	-7.65575
C	0.59675	-14.47380	-8.52114
C	-0.14611	-15.22137	-9.62657
C	0.79476	-16.06369	-10.48593

C	0.04629	-16.81343	-11.58223
C	14.13535	6.69674	-5.88069
C	14.81700	7.38746	-7.06953
C	15.93761	6.53075	-7.65466
C	16.71642	7.22986	-8.77033
C	3.12684	15.95586	-0.90654
C	1.98290	16.77795	-0.31749
C	2.46654	17.74227	0.76380
C	1.32829	18.58453	1.33702
C	1.81411	19.53192	2.42826
C	4.16833	8.42617	8.65745
C	2.86236	9.13270	9.01325
C	3.10073	10.53545	9.56845
C	1.79396	11.25339	9.90097
C	2.03728	12.64645	10.47049
C	15.90217	7.43859	-10.04620
C	16.74906	8.04493	-11.15983
H	5.97904	5.00766	-16.28496
H	5.09160	5.40775	-17.76286
H	-6.58510	10.26228	-8.96597
H	-6.95575	8.61598	-8.43172
H	1.91590	14.27965	-1.50848
H	2.11777	15.51595	-2.75863
H	3.41327	6.42378	8.89797
H	3.27354	7.05990	7.25233
H	16.73738	-0.08466	5.15538
H	15.86583	-1.53937	4.64957
H	10.49241	-8.40835	2.71022
H	9.24265	-8.43553	1.45739
H	0.24726	-1.87512	12.12221
H	1.47021	-1.96463	13.39856
H	-11.42683	2.02311	-9.22208
H	-10.63135	1.61556	-7.69449
H	0.43171	-4.49458	-16.37884
H	1.44635	-3.11598	-15.92973
H	0.92866	-13.62403	-5.92194
H	1.14182	-12.23388	-6.99629
H	-7.46878	-5.23983	3.92109
H	-8.81560	-6.20468	3.29926
H	1.34597	0.17780	11.22755
H	2.55353	0.08814	12.50808
H	-0.45064	0.44423	12.92462
H	0.74116	0.31746	14.21811
H	1.88224	2.36007	13.37455
H	0.70054	2.48721	12.07226
H	0.05362	2.61137	15.05188
H	-1.12069	2.75590	13.74431
H	1.21540	4.66586	14.23749
H	-0.47926	4.99508	14.60451
H	0.04187	4.81074	12.92809
H	-8.91832	-4.72735	1.28366
H	-7.61081	-3.74849	1.94607
H	-9.02267	-3.21714	3.93608
H	-10.32755	-4.19756	3.26863
H	-10.42571	-2.69658	1.27603
H	-9.13960	-1.70694	1.96536
H	-11.86811	-2.19613	3.24215
H	-10.58256	-1.20730	3.93459
H	-11.98479	-0.66737	1.27179
H	-10.69743	0.32168	1.96229
H	-12.25900	0.21149	2.77772
H	8.45237	-10.64374	2.31297
H	9.71629	-10.61596	3.54107
H	10.17416	-10.60333	0.52164
H	11.44994	-10.53949	1.73732
H	9.43678	-12.81198	1.42515
H	10.73081	-12.74772	2.62082
H	12.41727	-12.68192	0.78290
H	11.11745	-12.76546	-0.40603
H	11.73489	-14.90440	1.68984
H	12.10969	-15.00872	-0.03171
H	10.42889	-14.98821	0.50700
H	-7.94073	9.90235	-11.01953
H	-8.30658	8.25941	-10.49649
H	-9.05941	10.79352	-8.96705
H	-9.44980	9.14567	-8.47469
H	-10.39461	10.49498	-11.04437
H	-10.77989	8.84295	-10.56395
H	-11.53328	11.34968	-8.99514
H	-11.93247	9.69514	-8.53304
H	-12.87545	11.09547	-11.08254
H	-13.86143	10.78128	-9.65290
H	-13.26932	9.43641	-10.63059
H	4.62858	2.93073	-16.04073
H	3.73594	3.33432	-17.50585
H	6.74109	2.81484	-17.34758
H	5.86761	3.25903	-18.81338
H	5.35388	0.74991	-17.15391
H	4.48950	1.19756	-18.62420
H	7.48113	0.62885	-18.42629
H	6.64022	1.11769	-19.89722
H	6.06291	-1.43099	-18.31002
H	6.95447	-1.34702	-19.83133
H	5.23866	-0.93655	-19.78998
H	0.67573	-1.87974	-17.93301
H	-0.39660	-3.21147	-18.36174
H	2.64763	-3.38027	-18.18604
H	1.57759	-4.72401	-18.58545
H	1.86344	-2.14549	-20.19107
H	0.75517	-3.45962	-20.57950
H	3.79223	-3.72109	-20.43254
H	2.66959	-5.01513	-20.84763
H	3.04127	-2.42994	-22.43739
H	1.92050	-3.72745	-22.85078
H	3.66108	-4.01804	-22.89360
H	-11.65907	-0.65688	-7.77910
H	-12.47212	-0.22887	-9.28341
H	-12.85961	1.18781	-6.60536
H	-13.67869	1.61025	-8.10887
H	-13.90204	-1.07237	-6.67101
H	-14.72216	-0.65148	-8.17408
H	-15.10004	0.77420	-5.50237
H	-15.92431	1.18828	-7.00558
H	-16.15938	-1.48527	-5.54187
H	-16.98175	-1.07487	-7.04770
H	-17.37916	-0.20944	-5.56149
H	15.84630	-2.34118	7.00752
H	16.70618	-0.88742	7.51146
H	17.76166	-2.97967	5.55287
H	18.61729	-1.51229	6.02602
H	17.77070	-3.74845	7.92249
H	18.62083	-2.27828	8.39634
H	20.52708	-2.90666	6.91893
H	19.67910	-4.38180	6.45537
H	20.56072	-3.67343	9.29609
H	21.34915	-4.84296	8.23501
H	19.71638	-5.15074	8.83076
H	-0.87551	-12.91892	-8.28084
H	-1.09894	-14.29687	-7.20484
H	1.34685	-13.81777	-8.97022

H	1.12425	-15.19447	-7.89100
H	-0.66720	-14.50118	-10.26241
H	-0.90171	-15.87123	-9.17773
H	1.54742	-15.41414	-10.93978
H	1.31854	-16.78123	-9.84942
H	-0.46673	-16.11493	-12.24040
H	-0.69539	-17.48177	-11.14907
H	0.73515	-17.40633	-12.18019
H	14.88278	6.48978	-5.11138
H	13.71486	5.74531	-6.21332
H	15.22617	8.34520	-6.73878
H	14.06517	7.59312	-7.83327
H	15.51808	5.59721	-8.03797
H	16.63484	6.27330	-6.85289
H	17.59411	6.62460	-9.01327
H	17.07431	8.19725	-8.40867
H	3.62932	15.40419	-0.10839
H	3.86337	16.62550	-1.35754
H	1.23539	16.10418	0.10913
H	1.49762	17.34519	-1.11586
H	2.93503	17.17341	1.57097
H	3.22755	18.40444	0.34309
H	0.56113	17.92296	1.74710
H	0.86856	19.16403	0.53258
H	2.25097	18.97207	3.25292
H	0.98895	20.12593	2.81545
H	2.57074	20.20909	2.03674
H	4.70156	9.00426	7.89877
H	4.80849	8.37392	9.54131
H	2.23391	9.19986	8.12146
H	2.32053	8.53947	9.75429
H	3.65837	11.12417	8.83570
H	3.71357	10.46716	10.47084
H	1.18645	11.33303	8.99601
H	1.23084	10.65950	10.62505
H	2.58427	13.26010	9.75741
H	2.61999	12.58737	11.38770
H	1.09354	13.13928	10.69466
H	15.50167	6.47743	-10.37863
H	15.05789	8.09946	-9.84187
H	17.58758	7.39424	-11.40075
H	17.14398	9.01212	-10.85473
H	16.15443	8.18587	-12.05999
C	0.90598	-11.74758	6.29046
H	1.96909	-11.62007	6.09610
H	0.48174	-12.35161	5.49120
H	0.78777	-12.28186	7.23080
H	-0.84591	-10.53148	6.58570
H	0.66009	-9.80707	7.17271
C	-0.05493	-6.62685	1.02955
H	0.13363	-6.10970	0.09096
H	0.07759	-5.92310	1.84918
H	-1.08616	-6.97184	1.03038
H	0.72825	-8.51284	0.36352
H	1.92714	-7.45533	1.12018
C	-7.91232	-9.27196	-7.69809
H	-7.70942	-9.28374	-8.76731
H	-8.80177	-9.86828	-7.50587
H	-7.07062	-9.72979	-7.18255
H	-8.98253	-7.40209	-7.71075
H	-8.31433	-7.85573	-6.13438
C	-3.61611	-3.24709	-7.90788
H	-2.71802	-2.64485	-8.02812
H	-3.91156	-3.22886	-6.86049
H	-4.41151	-2.80041	-8.50035
H	-3.08001	-4.67280	-9.41955
H	-2.52564	-5.09859	-7.79588
C	2.54246	7.31012	-8.75736
H	3.56208	7.03352	-8.49594
H	2.42808	7.23411	-9.83643
H	1.85842	6.60592	-8.28835
H	1.21534	8.98550	-8.57254
H	2.31795	8.78464	-7.20575
C	7.05323	11.48180	-11.72217
H	7.54135	10.59935	-11.31439
H	7.81470	12.13077	-12.14966
H	6.38451	11.16174	-12.51811
H	6.97221	12.56541	-9.86070
H	5.79789	13.09886	-11.07141
C	8.39658	6.98803	-0.49066
H	7.47043	6.77574	-1.02101
H	8.82062	6.04919	-0.13976
H	9.09579	7.44332	-1.18825
H	7.71439	8.86270	0.31167
H	7.39334	7.46648	1.34928
C	14.27837	8.47044	2.88836
H	15.17627	8.65515	3.47443
H	14.31377	9.09087	1.99569
H	14.27852	7.42869	2.57495
H	13.03123	8.17864	4.62325
H	13.06488	9.83786	4.01405
H	6.34443	0.02662	8.61863
H	10.39549	-0.99670	7.65156
H	6.82959	2.39668	8.30807
H	13.03533	3.78098	5.04990
H	12.98752	5.76909	3.60416
H	9.20779	6.93864	5.21568
H	8.63909	11.35728	1.16015
H	11.15831	9.03788	-1.39596
H	7.60213	12.46221	-0.79082
H	9.64597	9.97210	-7.22742
H	7.93922	10.10147	-8.99436
H	5.26325	12.14393	-6.36040
H	0.54348	10.90839	-9.80795
H	3.05060	8.64848	-12.42516
H	-1.48182	9.91059	-10.80939
H	-0.37119	4.75802	-15.68428
H	-2.12163	3.06364	-15.82626
H	-4.47574	5.17235	-12.93145
H	9.05933	6.53164	2.75119
H	12.09517	10.20756	1.64822
H	4.44742	9.69583	-7.16646
H	3.97922	11.37838	-11.73888
H	9.45554	-2.63439	6.69852
H	6.24411	-1.88616	9.44389
H	8.55266	-4.83944	6.18450
H	1.91989	-6.02184	8.63846
H	0.54377	-7.45318	7.18768
H	3.96416	-8.47662	4.83838
H	0.32358	-11.08167	0.65300
H	-2.82002	-8.69305	2.28006
H	-0.90450	-11.32922	-1.47724
H	-6.73192	-7.22059	-2.09958
H	-7.21834	-6.45777	-4.39265
H	-4.18499	-9.02089	-5.96768
H	-4.13521	-5.90476	-11.16727
H	-7.16355	-3.35475	-9.56536
H	-3.67082	-4.29340	-12.97988
H	-7.19031	1.94940	-12.59413
H	-5.99981	3.87724	-13.49984

H	-2.78785	1.35752	-14.80319
H	2.19426	-7.32166	3.45098
H	-1.18069	-10.80087	4.11899
H	-3.83119	-6.27377	-6.28932
H	-7.65501	-6.26008	-9.34553
H	3.17583	6.34318	-12.76537
H	4.97354	5.35932	-14.10972
H	2.64441	5.90464	-17.64242
H	0.84395	6.90595	-16.29901
H	-3.41589	9.74788	-12.45313
H	-5.21845	10.61877	-11.02591
H	-5.82694	6.71445	-9.41635
H	-4.00524	5.85023	-10.80777
H	5.68072	11.45138	-1.94157
H	3.76782	12.69126	-1.02133
H	4.05923	15.50896	-4.21174
H	5.97455	14.27611	-5.12969
H	9.72899	8.17152	-5.60795
H	11.48639	6.69611	-6.48867
H	14.36388	8.64504	-4.01385
H	12.61502	10.13660	-3.14972
H	7.78598	4.63451	9.16930
H	5.63057	5.66347	9.75018
H	5.10997	6.86006	5.68830
H	7.23885	5.80265	5.09824
H	13.49681	2.61569	7.31332
H	15.62457	1.55011	6.69502
H	13.48713	-1.72934	4.99871
H	11.36328	-0.65081	5.57916
H	2.31838	-3.65000	8.29460
H	0.77535	-2.53810	9.85274
H	3.85797	-2.38698	12.79889
H	5.40065	-3.50018	11.24538
H	8.20925	-7.35254	6.71795
H	9.84608	-8.22928	5.10679
H	6.92462	-7.98241	2.00750
H	5.30127	-7.07650	3.60288
H	-1.44903	-0.14543	-13.73928
H	0.46859	-1.16536	-14.87259
H	-2.05116	-4.26590	-16.36970
H	-3.98530	-3.22479	-15.26566
H	-6.99355	-1.14055	-8.86001
H	-8.82793	-0.07152	-7.63712
H	-10.63512	1.07545	-11.32778
H	-8.80524	-0.01202	-12.55942
H	-6.97377	-8.88413	-0.12004
H	-8.53273	-7.57612	1.25893
H	-5.24905	-5.41286	2.91420
H	-3.69443	-6.69740	1.52616
H	-2.60476	-12.43681	-3.02508
H	-0.98927	-13.71667	-4.36573
H	-0.33848	-10.30082	-6.83769
H	-1.92486	-9.01318	-5.48581

*Table 2 Cartesian coordinates of **1b** (GFN2-xTB(GBSA(toluene)). B97-3c(COSMO(toluene) single point energy = 10838.55412 Hartree.*

	X	Y	Z
C	6.88729	1.21248	8.16437
C	7.80047	0.16767	8.01699
C	9.12432	0.47484	7.65985
C	9.45478	1.76694	7.31697
C	8.49291	2.80814	7.41972
C	7.22674	2.53095	7.89266
C	9.08769	3.98001	6.82134
C	10.42236	3.60352	6.37699
C	8.74770	5.20571	6.32419
C	10.90522	4.67525	5.53421
C	10.67514	2.29892	6.69030
C	12.03083	4.82197	4.75031
C	12.08656	5.87559	3.84384
C	11.05313	6.80037	3.73820
C	9.95892	6.71104	4.61080
C	9.86544	5.64349	5.47602
C	11.15178	9.17511	-2.22694
C	10.45172	9.72571	-1.15889
C	9.50596	10.72860	-1.39360
C	9.19760	11.06789	-2.69462
C	9.80255	10.37874	-3.77897
C	10.83334	9.49206	-3.54299
C	9.05957	10.75370	-4.96270
C	8.07729	11.75323	-4.55597
C	8.85067	10.39110	-6.26125
C	7.26442	12.04863	-5.71624
C	8.14501	11.95576	-3.20820
C	6.18830	12.88423	-5.93944
C	5.46714	12.74406	-7.11880
C	5.80461	11.78729	-8.07340
C	6.96525	11.02502	-7.89963
C	7.70016	11.16940	-6.74222
C	0.63359	10.16592	-11.96067
C	1.92283	9.69885	-11.73212
C	2.30076	8.44138	-12.21833
C	1.34834	7.62161	-12.78467
C	0.00426	8.06866	-12.90549
C	-0.33305	9.35871	-12.55096
C	-0.77296	6.92030	-13.31670
C	0.15943	5.81738	-13.50445
C	-2.05813	6.46163	-13.33389
C	-0.62878	4.61640	-13.66454
C	1.43254	6.21783	-13.21416
C	-0.30018	3.28386	-13.80759
C	-1.30439	2.33082	-13.69258
C	-2.62822	2.69027	-13.44941
C	-2.97763	4.04838	-13.42302
C	-1.98826	5.00278	-13.50847
C	10.96097	7.71443	2.58248
C	9.78924	7.59852	1.83526
C	9.58356	8.27613	0.64903
C	10.60136	9.11611	0.18154
C	11.74728	9.27584	0.94622
C	11.95162	8.59329	2.14173
C	4.83415	11.42856	-9.12626
C	3.56457	11.07956	-8.66927
C	2.58136	10.56801	-9.49585
C	2.87688	10.41189	-10.85643
C	4.12053	10.81406	-11.32501
C	5.11089	11.31855	-10.48959
C	1.29524	10.09432	-8.87307
C	6.43301	11.73326	-11.07829
C	13.21657	8.83853	2.92044
C	8.32600	8.00889	-0.13311
C	7.91685	-2.14136	7.15492
C	7.32148	-1.22126	8.01958
C	6.17026	-1.60658	8.72695
C	5.57573	-2.81925	8.45131
C	6.15647	-3.70390	7.50307
C	7.35784	-3.38557	6.90562

C	5.22905	-4.79500	7.33067
C	4.12694	-4.57434	8.25973
C	4.96737	-5.86559	6.52785
C	3.12261	-5.57865	7.97273
C	4.31494	-3.40409	8.93852
C	1.83828	-5.80732	8.42548
C	1.04028	-6.73136	7.75684
C	1.50314	-7.42783	6.64421
C	2.82956	-7.24993	6.23136
C	3.63166	-6.34946	6.89377
C	-0.49711	-9.34504	1.19002
C	-1.42755	-9.41961	2.22460
C	-2.77078	-9.68058	1.93552
C	-3.16713	-9.77665	0.61504
C	-2.21196	-9.64275	-0.42667
C	-0.87641	-9.45643	-0.13941
C	-2.94739	-9.66652	-1.66725
C	-4.35121	-9.87950	-1.33889
C	-2.80230	-9.36001	-2.98673
C	-5.09601	-9.73786	-2.57521
C	-4.50339	-9.94679	0.01778
C	-6.42650	-9.84690	-2.92485
C	-6.82363	-9.52441	-4.22169
C	-5.91721	-9.06862	-5.17486
C	-4.55422	-9.03659	-4.83832
C	-4.14875	-9.36894	-3.56890
C	-5.94581	-6.21674	-11.27559
C	-6.22195	-5.98179	-9.93048
C	-6.31480	-4.65268	-9.48841
C	-6.07333	-3.61958	-10.36191
C	-5.64604	-3.87836	-11.69211
C	-5.63289	-5.17953	-12.15224
C	-5.23876	-2.59898	-12.23861
C	-4.51945	-2.03925	-13.25640
C	-5.01692	-0.33409	-11.71303
C	-5.06481	0.95226	-11.21858
C	-4.34198	1.93939	-11.87028
C	-3.57977	1.66160	-13.00477
C	-3.62343	0.37863	-13.56795
C	-4.33734	-0.61463	-12.92895
C	0.59651	-8.16228	5.74282
C	-0.52638	-7.47445	5.29005
C	-1.29415	-7.90916	4.22181
C	-0.91155	-9.08546	3.56908
C	0.13017	-9.83766	4.10318
C	0.88682	-9.41244	5.18474
C	-6.26465	-8.50415	-6.49213
C	-5.41073	-8.78597	-7.56145
C	-5.39070	-8.06601	-8.74381
C	-6.31921	-7.02119	-8.88996
C	-7.26791	-6.84418	-7.88501
C	-7.26135	-7.54035	-6.68625
C	-4.34379	-8.35411	-9.79089
C	-8.28946	-7.16414	-5.65443
C	1.97259	-10.30483	5.72813
C	-2.46616	-7.07268	3.78655
C	2.64489	5.42170	-13.30778
C	-3.27513	7.23889	-13.16134
C	-4.00228	-2.70177	-14.44288
C	-6.41364	-1.56618	-8.83733
C	-1.57496	-9.08907	-3.71815
C	-5.73598	-10.12210	0.76790
C	5.76862	-6.43510	5.45633
C	3.43686	-2.81239	9.93438
C	7.50443	5.92460	6.54639
C	11.88265	1.54093	6.40765
C	9.54035	9.38720	-7.05511
C	7.32252	12.80713	-2.36331
C	-6.02046	-2.17820	-10.09603
C	-5.51683	-1.59339	-11.21949
C	3.60399	5.39452	-12.29604
C	4.74614	4.62852	-12.43141
C	4.97049	3.87011	-13.57429
C	4.00848	3.88978	-14.57901
C	2.86272	4.64711	-14.44940
C	6.23846	3.08748	-13.75127
C	-3.46434	8.40816	-13.89924
C	-4.60904	9.16311	-13.73981
C	-5.59375	8.78643	-12.83281
C	-5.40100	7.62547	-12.09296
C	-4.26311	6.85921	-12.25372
C	-6.85056	9.59286	-12.68530
C	9.82665	8.13160	-6.51837
C	10.47501	7.17689	-7.27458
C	10.86110	7.44138	-8.58491
C	10.57077	8.69065	-9.12016
C	9.91699	9.65067	-8.37172
C	5.93472	12.82146	-2.50136
C	5.15650	13.61820	-1.68574
C	5.73419	14.42771	-0.71348
C	7.11790	14.41510	-0.57955
C	7.90323	13.61217	-1.38349
C	11.53542	6.37990	-9.40373
C	6.98818	6.02234	7.84014
C	5.80370	6.68916	8.07743
C	5.08897	7.27058	7.03517
C	5.59940	7.16677	5.74660
C	6.78782	6.50633	5.50137
C	3.81860	8.02206	7.30555
C	13.13240	2.07784	6.71179
C	14.29280	1.37250	6.45189
C	14.24701	0.11285	5.86757
C	12.99671	-0.41420	5.54874
C	11.83429	0.27879	5.81459
C	15.47113	-0.71160	5.56700
C	2.92317	-3.60090	10.96504
C	2.08210	-3.05708	11.91464
C	1.71980	-1.71515	11.86474
C	2.22315	-0.93298	10.83163
C	3.07250	-1.46728	9.88238
C	6.34151	-5.62183	4.47920
C	7.09579	-6.17286	3.46241
C	7.30770	-7.54557	3.39121
C	6.73431	-8.35489	4.36542
C	5.96706	-7.81350	5.37813
C	8.17844	-8.13279	2.31949
C	0.76482	-1.14162	12.87007
C	-2.67631	-2.54430	-14.84494
C	-2.19994	-3.19949	-15.96346
C	-3.02773	-4.02182	-16.71957
C	-4.35153	-4.17304	-16.32131
C	-4.83245	-3.53115	-15.19773
C	-7.61505	-1.92154	-8.22345
C	-7.99771	-1.33021	-7.03581
C	-7.19250	-0.38049	-6.41679
C	-5.98905	-0.03741	-7.02318
C	-5.60272	-0.61747	-8.21527
C	-7.63802	0.29430	-5.15273
C	-2.51480	-4.69684	-17.95771
C	-6.64801	-11.11047	0.39642

C	-7.81943	-11.28895	1.10521	C	4.11647	9.47991	7.67985
C	-8.12135	-10.48708	2.20043	C	2.83898	10.26518	7.96711
C	-7.21726	-9.49441	2.56222	C	3.12941	11.71483	8.35057
C	-6.04062	-9.31344	1.86291	C	1.85169	12.50167	8.63676
C	-0.49032	-9.95891	-3.59840	C	2.14593	13.94803	9.01854
C	0.67276	-9.73489	-4.30729	C	2.69518	19.87926	0.49251
C	0.79669	-8.63381	-5.14780	C	2.47013	21.22968	-0.17824
C	-0.27794	-7.75830	-5.25216	H	6.04008	2.19225	-14.34491
C	-1.44902	-7.97997	-4.55362	H	6.62651	2.77487	-12.77954
C	2.04038	-8.42490	-5.96110	H	-6.63924	10.64775	-12.87396
C	-9.41342	-10.65129	2.94587	H	-7.23550	9.49820	-11.66755
C	-0.68370	-1.29528	12.38744	H	12.22398	5.81938	-8.76964
C	-1.68514	-0.75622	13.40604	H	12.10701	6.83782	-10.21366
C	-3.12732	-0.89900	12.92358	H	3.27598	7.54853	8.12697
C	-4.13245	-0.37855	13.94941	H	3.18010	8.00447	6.41973
C	-5.57017	-0.52295	13.46318	H	15.39404	-1.07259	4.53643
C	-10.48761	-9.71326	2.37954	H	15.45124	-1.59679	6.21221
C	-11.81027	-9.83808	3.13186	H	8.10193	-7.53539	1.40826
C	-12.87366	-8.89006	2.58071	H	7.85309	-9.14980	2.09014
C	-14.19836	-9.00843	3.33208	H	0.87813	-1.65773	13.82591
C	-15.25119	-8.05085	2.78509	H	0.97861	-0.08205	13.02613
C	9.64283	-8.16444	2.77679	H	-6.76861	0.61164	-4.57291
C	10.56440	-8.74856	1.70875	H	-8.21934	-0.40166	-4.54384
C	12.01876	-8.78268	2.17570	H	-3.06885	-5.62146	-18.13344
C	12.99511	-9.25435	1.09655	H	-1.45949	-4.94952	-17.83273
C	12.81524	-10.72046	0.71704	H	2.90890	-8.80550	-5.41915
C	-7.92319	9.11354	-13.67214	H	2.19133	-7.35876	-6.14394
C	-9.21562	9.91589	-13.54199	H	-9.26283	-10.42147	4.00317
C	-10.28572	9.44389	-14.52432	H	-9.76170	-11.68333	2.86702
C	-11.58335	10.23840	-14.38862	H	-0.88747	-2.35239	12.19991
C	-12.64776	9.76400	-15.37157	H	-0.80328	-0.76450	11.43981
C	7.29646	3.93726	-14.46824	H	-1.56598	-1.29539	14.34928
C	8.56326	3.14172	-14.77330	H	-1.47239	0.29814	13.60006
C	9.62153	3.99810	-15.46573	H	-3.25099	-0.34948	11.98690
C	10.88722	3.20594	-15.78754	H	-3.33624	-1.95187	12.71742
C	11.95122	4.07687	-16.44599	H	-3.92421	0.67414	14.15642
C	-2.66728	-3.78200	-19.18003	H	-4.00986	-0.92939	14.88529
C	-2.15663	-4.44782	-20.45568	H	-5.72042	0.03850	12.54309
C	-2.28775	-3.53465	-21.67297	H	-6.26645	-0.14934	14.21096
C	-1.78293	-4.20238	-22.95089	H	-5.80396	-1.56770	13.26774
C	-1.90358	-3.28282	-24.16087	H	-10.64446	-9.94492	1.32320
C	-8.50222	1.52086	-5.47400	H	-10.12956	-8.68263	2.44035
C	-8.97114	2.23754	-4.21018	H	-11.64596	-9.61864	4.18998
C	-9.84406	3.44939	-4.53007	H	-12.17196	-10.86704	3.06099
C	-10.31153	4.17323	-3.26883	H	-13.03912	-9.10846	1.52263
C	-11.18908	5.37647	-3.59502	H	-12.51079	-7.86145	2.65167
C	16.81172	-0.00260	5.74196	H	-14.56670	-10.03443	3.25447
C	17.97526	-0.90408	5.33081	H	-14.03187	-8.79660	4.39126
C	19.32365	-0.21133	5.52169	H	-15.44658	-8.25764	1.73462
C	20.51018	-1.04122	5.02786	H	-14.91217	-7.02040	2.87285
C	20.76593	-2.28863	5.86721	H	-16.18463	-8.15314	3.33456
C	1.92978	-9.15407	-7.30690	H	9.71906	-8.75963	3.69014
C	3.17270	-8.95532	-8.17093	H	9.96245	-7.14799	3.01973
C	3.06641	-9.69496	-9.50320	H	10.23052	-9.75890	1.46644
C	4.30421	-9.49472	-10.37568	H	10.49198	-8.14558	0.79989
C	4.19476	-10.24290	-11.69960	H	12.10304	-9.43649	3.04743
C	4.88527	15.32095	0.14286	H	12.30811	-7.77645	2.49113
C	4.65974	16.67697	-0.53858	H	14.01402	-9.10931	1.46516
C	3.79284	17.60222	0.31208	H	12.87560	-8.63221	0.20603
C	3.56358	18.95492	-0.35904	H	12.88446	-11.35316	1.59985
C	10.49691	5.42071	-10.00068	H	13.58931	-11.02555	0.01572
C	11.11643	4.31392	-10.85464	H	11.84927	-10.88734	0.24734
C	11.93778	3.30953	-10.04779	H	-7.53801	9.20288	-14.69080
C	12.44037	2.15507	-10.91286	H	-8.12995	8.05611	-13.49013
C	13.27418	1.16335	-10.10931	H	-9.00432	10.97339	-13.71997

H	-9.59718	9.82316	-12.52193	H	4.18118	16.51585	-1.50765
H	-9.90781	9.54290	-15.54508	H	5.62729	17.14971	-0.72411
H	-10.49254	8.38472	-14.35120	H	2.82751	17.12388	0.49677
H	-11.37724	11.29767	-14.56138	H	4.27382	17.75804	1.28117
H	-11.96226	10.13781	-13.36848	H	3.08269	18.80057	-1.32836
H	-12.29914	9.87760	-16.39622	H	4.52808	19.43503	-0.54289
H	-13.56240	10.34137	-15.25404	H	9.80480	6.00010	-10.61764
H	-12.88052	8.71410	-15.20459	H	9.91715	4.97203	-9.19033
H	6.87467	4.31850	-15.40154	H	11.74797	4.76106	-11.62614
H	7.54585	4.79867	-13.84400	H	10.30881	3.77722	-11.35889
H	8.31080	2.29274	-15.41369	H	12.79792	3.80902	-9.59790
H	8.97390	2.74102	-13.84342	H	11.32278	2.90943	-9.23732
H	9.20679	4.40465	-16.39172	H	11.58669	1.63462	-11.35440
H	9.87904	4.84290	-14.82225	H	13.04451	2.55501	-11.73095
H	10.63479	2.37665	-16.45307	H	12.68684	0.74157	-9.29587
H	11.28923	2.77856	-14.86615	H	14.14604	1.65500	-9.68192
H	11.57703	4.49903	-17.37667	H	13.61681	0.34841	-10.74347
H	12.84174	3.49251	-16.66771	H	4.66214	9.95547	6.86124
H	12.23328	4.89686	-15.78842	H	4.76264	9.49860	8.56076
H	-2.11497	-2.85564	-19.00472	H	2.19769	10.24801	7.08214
H	-3.72091	-3.51873	-19.30109	H	2.29200	9.78036	8.77982
H	-1.10711	-4.72353	-20.32430	H	3.67728	12.20025	7.53894
H	-2.71916	-5.36795	-20.63318	H	3.76916	11.73276	9.23657
H	-1.72055	-2.61685	-21.49819	H	1.21192	12.48397	7.75090
H	-3.33602	-3.25394	-21.80294	H	1.30431	12.01687	9.44902
H	-0.73703	-4.49015	-22.81810	H	2.67352	14.45556	8.21333
H	-2.35611	-5.11526	-23.13110	H	2.76595	13.98849	9.91201
H	-1.32237	-2.37520	-24.00973	H	1.22207	14.48727	9.21720
H	-2.94181	-3.00011	-24.32334	H	3.17538	20.03235	1.46222
H	-1.53827	-3.77913	-25.05755	H	1.72989	19.40022	0.67450
H	-9.37011	1.20376	-6.05737	H	1.97406	21.10119	-1.13830
H	-7.92506	2.21212	-6.09301	H	3.41926	21.73399	-0.34901
H	-9.53710	1.53983	-3.58771	H	1.84938	21.86862	0.44634
H	-8.10068	2.56144	-3.63387	C	3.06701	-10.62059	4.70717
H	-10.71657	3.12440	-5.10265	H	3.55065	-9.71015	4.35957
H	-9.28012	4.14529	-5.15640	H	2.65592	-11.13570	3.84243
H	-10.87142	3.47651	-2.64004	H	3.82252	-11.26037	5.15864
H	-9.43967	4.50417	-2.69893	H	1.51287	-11.24665	6.04490
H	-12.07451	5.06492	-4.14585	H	2.41905	-9.84187	6.60978
H	-10.64255	6.09349	-4.20454	C	-3.67523	-7.31868	4.69236
H	-11.51000	5.87512	-2.68292	H	-4.51619	-6.70446	4.37695
H	16.94257	0.28707	6.78698	H	-3.43259	-7.07025	5.72343
H	16.82767	0.90420	5.13241	H	-3.97360	-8.36430	4.65807
H	17.94315	-1.81839	5.92609	H	-2.72952	-7.29909	2.75218
H	17.86219	-1.18525	4.28047	H	-2.19345	-6.01548	3.84140
H	19.46441	0.02135	6.58027	C	-9.61730	-7.88107	-5.91236
H	19.31014	0.73599	4.97575	H	-10.01553	-7.59887	-6.88487
H	21.40530	-0.41396	5.05015	H	-10.34563	-7.61678	-5.14829
H	20.33834	-1.33233	3.98857	H	-9.48322	-8.96085	-5.90694
H	20.90883	-2.02104	6.91242	H	-8.46197	-6.08557	-5.70230
H	21.66134	-2.79972	5.51915	H	-7.92725	-7.39870	-4.65287
H	19.93287	-2.98368	5.79989	C	-3.05335	-8.98157	-9.26702
H	1.05126	-8.78516	-7.84169	H	-2.31082	-8.99659	-10.06270
H	1.77932	-10.22074	-7.12382	H	-3.20542	-10.00609	-8.93605
H	3.31419	-7.88828	-8.36085	H	-2.65497	-8.40031	-8.43830
H	4.05170	-9.31321	-7.62880	H	-4.08886	-7.41580	-10.28928
H	2.18430	-9.34096	-10.04288	H	-4.77345	-9.01977	-10.54692
H	2.92945	-10.76247	-9.31280	C	1.37180	8.59995	-8.55148
H	4.43773	-8.42803	-10.57231	H	2.21395	8.39408	-7.89349
H	5.18729	-9.84374	-9.83459	H	1.49827	8.02452	-9.46598
H	3.33135	-9.89594	-12.26392	H	0.45755	8.27089	-8.06198
H	4.08238	-11.31134	-11.52595	H	0.44959	10.26637	-9.53752
H	5.08582	-10.08520	-12.30351	H	1.12082	10.65027	-7.94906
H	5.37193	15.48350	1.10699	C	7.21871	10.54295	-11.63257
H	3.91684	14.84921	0.32431	H	7.40754	9.80795	-10.85294

H	8.17429	10.87689	-12.03159
H	6.66522	10.05314	-12.43040
H	7.02814	12.24389	-10.31924
H	6.24961	12.44059	-11.89261
C	8.53745	6.85735	-1.11854
H	7.61925	6.65333	-1.66558
H	8.83700	5.95462	-0.58958
H	9.31688	7.11293	-1.83290
H	8.01907	8.89720	-0.68625
H	7.52255	7.74409	0.55831
C	14.45805	8.33070	2.18416
H	15.34898	8.50764	2.78315
H	14.57659	8.84048	1.23089
H	14.38089	7.26339	1.98762
H	13.14707	8.35556	3.89640
H	13.32470	9.91472	3.08515
H	5.86717	0.99074	8.43928
H	9.86738	-0.30694	7.61677
H	6.48708	3.31256	7.98588
H	12.83015	4.09635	4.78970
H	12.92302	5.93893	3.16458
H	9.17932	7.45686	4.55891
H	11.91351	8.43436	-2.02546
H	8.99317	11.18909	-0.56044
H	11.35341	9.01277	-4.35925
H	5.88150	13.60587	-5.19656
H	4.59533	13.36170	-7.28392
H	7.23344	10.27777	-8.63095
H	0.37162	11.16804	-11.65021
H	3.31972	8.10995	-12.08254
H	-1.34197	9.72248	-12.67844
H	0.72650	2.97797	-13.94496
H	-1.04142	1.28329	-13.72329
H	-4.01283	4.33110	-13.30072
H	9.02616	6.91294	2.17807
H	12.51276	9.95343	0.59263
H	3.35986	11.16748	-7.61060
H	4.32623	10.71901	-12.38278
H	8.80908	-1.86385	6.61509
H	5.74522	-0.94384	9.46581
H	7.83664	-4.07053	6.22167
H	1.43423	-5.24265	9.25317
H	0.02276	-6.88613	8.08758
H	3.18868	-7.75378	5.34729
H	0.53766	-9.15109	1.43554
H	-3.48155	-9.78951	2.74094
H	-0.14607	-9.36128	-0.92914
H	-7.16597	-10.15719	-2.20097
H	-7.86342	-9.64155	-4.48417
H	-3.83209	-8.70723	-5.56916
H	-5.96119	-7.22754	-11.65384
H	-6.51659	-4.44506	-8.44778
H	-5.35296	-5.40494	-13.17097
H	-5.62660	1.18328	-10.32572
H	-4.31889	2.93513	-11.45265
H	-3.09047	0.18003	-14.48644
H	-0.76277	-6.52159	5.74413
H	0.37132	-10.78028	3.63122
H	-4.69747	-9.58422	-7.42827
H	-8.02381	-6.08316	-8.02407
H	3.43631	5.94817	-11.38398
H	5.47467	4.61587	-11.63252
H	4.16188	3.30225	-15.47368
H	2.12958	4.66522	-15.24332
H	-2.70935	8.70756	-14.61245
H	-4.74079	10.06350	-14.32380
H	-6.15091	7.32154	-11.37573
H	-4.11567	5.97976	-11.64423
H	9.51367	7.90643	-5.50929
H	10.68854	6.20877	-6.84280
H	10.86440	8.91566	-10.13615
H	9.71946	10.62476	-8.79511
H	5.47203	12.17982	-3.23714
H	4.08132	13.60875	-1.79977
H	7.58566	15.03897	0.16948
H	8.97818	13.62242	-1.27282
H	7.53851	5.58185	8.65940
H	5.42190	6.75692	9.08674
H	5.05323	7.60267	4.92145
H	7.14557	6.40475	4.48761
H	13.18620	3.05254	7.17576
H	15.23991	1.81892	6.71165
H	12.93886	-1.38761	5.08070
H	10.88044	-0.13740	5.52549
H	3.20960	-4.64132	11.02264
H	1.70063	-3.68158	12.71064
H	1.94483	0.11008	10.77071
H	3.42913	-0.85059	9.07032
H	6.16669	-4.55614	4.51138
H	7.52521	-5.52828	2.70780
H	6.88846	-9.42421	4.32597
H	5.53772	-8.45421	6.13471
H	-2.01016	-1.93413	-14.25255
H	-1.16538	-3.07679	-16.25282
H	-5.01125	-4.80685	-16.89773
H	-5.86549	-3.64894	-14.90295
H	-8.26127	-2.64347	-8.70175
H	-8.93656	-1.61001	-6.57829
H	-5.34497	0.69236	-6.55220
H	-4.65570	-0.35676	-8.66512
H	-6.41394	-11.75334	-0.44022
H	-8.50984	-12.06652	0.80846
H	-7.43979	-8.85599	3.40600
H	-5.36380	-8.52011	2.14275
H	-0.58037	-10.82647	-2.96008
H	1.49943	-10.42478	-4.20879
H	-0.19597	-6.88927	-5.89048
H	-2.26251	-7.27272	-4.62873

Table 3 Cartesian coordinates of ***1d*** *(GFN2-xTB(GBSA(toluene)). B97-3c(COSMO(toluene) single point energy = 10838.55287Hartree.*

	X	Y	Z
C	10.83422	-0.34867	7.00817
C	9.51070	-0.51666	7.41812
C	8.70497	0.62449	7.56213
C	9.18764	1.85583	7.18652
C	10.50265	1.99098	6.66701
C	11.33587	0.89085	6.63061
C	10.61002	3.34998	6.18592
C	9.36115	4.02456	6.52213
C	11.34125	4.11795	5.32616
C	9.37568	5.30571	5.85348
C	8.50004	3.15312	7.11969
C	8.46607	6.34016	5.77786
C	8.68903	7.35710	4.85819
C	9.80802	7.36158	4.02796
C	10.78016	6.36542	4.17911

C	10.56303	5.33974	5.07437
C	10.41858	9.64571	-1.93610
C	9.70749	10.26650	-0.91472
C	8.86043	11.33821	-1.21296
C	8.65018	11.67892	-2.53302
C	9.25599	10.92745	-3.57449
C	10.19860	9.96609	-3.27129
C	8.60486	11.33620	-4.80045
C	7.67565	12.40872	-4.45836
C	8.44319	10.97270	-6.10528
C	6.94265	12.73762	-5.66219
C	7.69464	12.63352	-3.11263
C	5.92984	13.63106	-5.94924
C	5.25407	13.50977	-7.15755
C	5.57748	12.51756	-8.08000
C	6.68460	11.69519	-7.84217
C	7.37165	11.81622	-6.65341
C	0.49631	10.90760	-12.09999
C	1.77984	10.44729	-11.82859
C	2.17100	9.18171	-12.28319
C	1.23331	8.34849	-12.85416
C	-0.11000	8.78710	-13.00770
C	-0.45642	10.08469	-12.69119
C	-0.87580	7.62456	-13.39988
C	0.06491	6.52316	-13.55529
C	-2.15750	7.15614	-13.40316
C	-0.71512	5.31329	-13.68952
C	1.33127	6.93676	-13.25482
C	-0.37759	3.97994	-13.80277
C	-1.37335	3.02225	-13.65344
C	-2.69540	3.37960	-13.39995
C	-3.05665	4.73419	-13.40912
C	-2.07679	5.69385	-13.53672
C	9.84477	8.26977	2.86513
C	8.70448	8.25118	2.06280
C	8.61961	8.93010	0.86228
C	9.73217	9.66670	0.43796
C	10.84881	9.73330	1.25795
C	10.93156	9.05117	2.46794
C	4.63293	12.17992	-9.16318
C	3.33581	11.88603	-8.74717
C	2.36945	11.37828	-9.59603
C	2.71153	11.17223	-10.93922
C	3.98328	11.52741	-11.37038
C	4.95706	12.02394	-10.51135
C	1.05183	10.95422	-9.00383
C	6.31487	12.37863	-11.05644
C	12.17245	9.19636	3.30873
C	7.38195	8.76771	0.02169
C	9.34154	-2.88808	6.71646
C	8.88295	-1.84062	7.51714
C	7.70731	-2.02852	8.26315
C	6.95310	-3.16448	8.07036
C	7.40242	-4.18717	7.19398
C	8.62756	-4.06907	6.57023
C	6.33404	-5.15384	7.10991
C	5.27728	-4.71267	8.01580
C	5.91507	-6.22624	6.37961
C	4.14310	-5.58833	7.80304
C	5.62188	-3.52153	8.58736
C	2.84704	-5.61332	8.27906
C	1.92366	-6.46553	7.68079
C	2.27006	-7.28583	6.61020
C	3.60258	-7.31558	6.17984
C	4.52929	-6.49426	6.77985

C	-0.10010	-8.99082	1.25670
C	-0.99284	-9.02337	2.32567
C	-2.35869	-9.20229	2.08837
C	-2.81043	-9.26232	0.78380
C	-1.89135	-9.16627	-0.29407
C	-0.53693	-9.06187	-0.05793
C	-2.67627	-9.13365	-1.50431
C	-4.07453	-9.28314	-1.12087
C	-2.57198	-8.80833	-2.82329
C	-4.86168	-9.08947	-2.32283
C	-4.17585	-9.35679	0.23993
C	-6.20865	-9.13924	-2.61790
C	-6.64412	-8.78132	-3.89261
C	-5.75876	-8.34896	-4.87605
C	-4.38293	-8.37956	-4.59703
C	-3.94033	-8.74732	-3.34967
C	-6.02990	-5.45909	-10.95924
C	-6.20255	-5.21398	-9.59896
C	-6.22598	-3.88261	-9.15563
C	-6.02463	-2.85770	-10.04860
C	-5.70759	-3.12902	-11.40677
C	-5.75781	-4.43157	-11.86080
C	-5.31421	-1.86009	-11.98759
C	-4.67089	-1.31726	-13.06430
C	-5.00057	0.39937	-11.48968
C	-4.98851	1.68758	-10.99816
C	-4.30072	2.65928	-11.70790
C	-3.62789	2.36121	-12.89245
C	-3.73548	1.07864	-13.44590
C	-4.42424	0.10211	-12.75420
C	1.25626	-7.93738	5.76163
C	0.20512	-7.13704	5.31933
C	-0.63817	-7.50799	4.28538
C	-0.40599	-8.73292	3.65197
C	0.55503	-9.58790	4.18013
C	1.38204	-9.22819	5.23419
C	-6.14143	-7.75193	-6.16891
C	-5.35144	-8.05225	-7.28104
C	-5.36602	-7.32277	-8.45803
C	-6.26741	-6.24942	-8.55194
C	-7.15387	-6.04465	-7.49674
C	-7.10877	-6.74862	-6.30354
C	-4.37915	-7.63582	-9.55507
C	-8.06401	-6.33845	-5.21587
C	2.36131	-10.23415	5.78207
C	-1.73112	-6.56501	3.86247
C	2.55513	6.15555	-13.31609
C	-3.37987	7.93052	-13.25819
C	-4.30376	-1.98730	-14.30115
C	-6.19890	-0.79913	-8.50705
C	-1.36706	-8.57369	-3.60289
C	-5.38543	-9.47169	1.03819
C	6.60801	-6.96617	5.33677
C	4.83118	-2.67414	9.46476
C	12.62977	3.79793	4.73307
C	7.14986	3.39378	7.60639
C	9.11547	9.92627	-6.85836
C	6.90613	13.56213	-2.31815
C	-5.91694	-1.41766	-9.79194
C	-5.48577	-0.84760	-10.95252
C	3.49774	6.15873	-12.28720
C	4.65938	5.42367	-12.40116
C	4.93249	4.66449	-13.53782
C	3.98443	4.64856	-14.55318
C	2.81429	5.37610	-14.44295

C	6.23700	3.91435	-13.60293	C	-8.99497	-9.82324	3.36075
C	-3.57178	9.07335	-14.03600	C	1.07997	0.06181	11.41492
C	-4.72338	9.82403	-13.91164	C	0.26075	1.09916	12.17892
C	-5.71341	9.46909	-13.00152	C	-1.12731	1.29391	11.57181
C	-5.51665	8.33683	-12.21967	C	-1.91890	2.39017	12.28238
C	-4.37104	7.57517	-12.34441	C	-3.30343	2.57994	11.67313
C	-6.98133	10.26496	-12.89909	C	-10.06634	-8.87709	2.80279
C	9.29523	8.65555	-6.31203	C	-11.37409	-8.97245	3.58489
C	9.93778	7.66757	-7.03044	C	-12.43846	-8.02117	3.04158
C	10.42508	7.91417	-8.30977	C	-13.75075	-8.12064	3.81705
C	10.23598	9.17794	-8.85697	C	-14.80749	-7.16507	3.27436
C	9.58719	10.17019	-8.14805	C	10.09784	-9.39238	2.63294
C	5.53040	13.68246	-2.51177	C	10.85922	-10.17396	1.56471
C	4.78621	14.55064	-1.73773	C	12.31938	-10.39568	1.95585
C	5.38761	15.32711	-0.75324	C	13.14690	-11.07077	0.86032
C	6.76012	15.20910	-0.56459	C	12.73271	-12.51377	0.59201
C	7.51026	14.33560	-1.32683	C	-8.01928	9.75916	-13.90961
C	11.09980	6.82577	-9.09202	C	-9.32778	10.54113	-13.82473
C	13.67215	3.34371	5.54211	C	-10.35853	10.04361	-14.83616
C	14.90293	3.03672	4.99793	C	-11.67293	10.81693	-14.74853
C	15.13137	3.16151	3.63167	C	-12.69752	10.31568	-15.76011
C	14.08792	3.60016	2.82483	C	6.44064	3.03530	-14.83404
C	12.85637	3.92002	3.36272	C	7.78049	2.30271	-14.77898
C	16.45576	2.78140	3.03740	C	8.00463	1.42246	-16.00681
C	6.89800	4.45666	8.47451	C	9.33617	0.67637	-15.94647
C	5.63531	4.65328	8.99656	C	9.55608	-0.19706	-17.17647
C	4.58128	3.81060	8.65807	C	-2.87163	-5.47708	-17.63957
C	4.82495	2.77673	7.76201	C	-2.53524	-6.26173	-18.90550
C	6.08940	2.56282	7.24782	C	-2.12968	-7.70177	-18.59691
C	3.21053	4.03259	9.22826	C	-1.80797	-8.49476	-19.86252
C	4.16937	-3.20122	10.57324	C	-1.42930	-9.93819	-19.54873
C	3.42398	-2.38395	11.40155	C	-7.96900	2.28201	-4.95508
C	3.31394	-1.02103	11.15111	C	-8.34049	2.97789	-3.64794
C	3.96361	-0.49867	10.03765	C	-9.23586	4.19311	-3.88166
C	4.71034	-1.30764	9.20653	C	-9.61599	4.88981	-2.57651
C	7.26662	-6.30213	4.30255	C	-10.51592	6.09692	-2.81607
C	7.90071	-7.01419	3.30389	C	2.46921	5.15416	8.49015
C	7.90445	-8.40495	3.30783	C	1.07460	5.38154	9.06915
C	7.25257	-9.06514	4.34314	C	0.32897	6.50706	8.35545
C	6.60448	-8.36092	5.33881	C	-1.06617	6.73091	8.93618
C	8.63010	-9.17372	2.24281	C	-1.80620	7.85752	8.22413
C	2.47765	-0.12774	12.01977	C	1.95058	-8.71690	-7.35712
C	-3.03265	-1.85977	-14.85931	C	3.15634	-8.55936	-8.28023
C	-2.71293	-2.51310	-16.03409	C	2.94721	-9.26732	-9.61750
C	-3.64920	-3.29983	-16.69454	C	4.14689	-9.10906	-10.54973
C	-4.91699	-3.42749	-16.13607	C	3.93476	-9.82797	-11.87739
C	-5.24070	-2.78788	-14.95713	C	4.58338	16.29879	0.05973
C	-7.35042	-1.14016	-7.79643	C	4.54367	17.67500	-0.61752
C	-7.62315	-0.55123	-6.57787	C	3.75344	18.69013	0.20483
C	-6.75510	0.38319	-6.02363	C	3.70849	20.06208	-0.46485
C	-5.60372	0.71547	-6.72884	C	10.06270	5.97583	-9.83867
C	-5.32672	0.13666	-7.95171	C	10.68803	4.88453	-10.70907
C	-7.08304	1.05133	-4.72058	C	11.33864	3.76388	-9.89922
C	-3.29460	-4.03565	-17.95355	C	11.96758	2.67564	-10.77221
C	-6.36113	-10.41112	0.70323	C	10.94351	1.84279	-11.53573
C	-7.51004	-10.53143	1.45908	C	16.48534	1.28660	2.69219
C	-7.72673	-9.71732	2.56561	C	17.81965	0.86966	2.07838
C	-6.76027	-8.77191	2.89015	C	17.85770	-0.62034	1.74462
C	-5.60445	-8.65062	2.14424	C	19.18863	-1.03741	1.12164
C	-0.31346	-9.48690	-3.54687	C	19.22531	-2.52678	0.79823
C	0.82351	-9.29654	-4.30644	C	2.92949	21.08236	0.36325
C	0.95150	-8.18800	-5.13642	C	2.89089	22.45065	-0.30792
C	-0.09078	-7.26907	-5.17654	H	6.31925	3.29274	-12.70540
C	-1.23522	-7.45580	-4.42587	H	7.04943	4.64722	-13.55397
C	2.16096	-8.01667	-6.00786	H	-6.77578	11.31933	-13.09703

H	-7.39388	10.18025	-11.89141
H	11.66994	6.19162	-8.41154
H	11.79038	7.26216	-9.81692
H	16.63598	3.36065	2.12926
H	17.25784	2.99877	3.74619
H	3.29119	4.29898	10.28515
H	2.62583	3.11384	9.15058
H	8.59083	-8.62649	1.29819
H	8.15217	-10.14467	2.09548
H	2.38324	-0.55391	13.02043
H	2.95957	0.84888	12.10800
H	-6.16447	1.36182	-4.21808
H	-7.61043	0.35254	-4.06733
H	-2.47642	-3.52545	-18.46622
H	-4.15700	-4.05727	-18.62400
H	3.03853	-8.44306	-5.51704
H	2.34823	-6.95477	-6.18154
H	-8.80174	-9.56623	4.40460
H	-9.37135	-10.84791	3.32538
H	0.55468	-0.89628	11.41586
H	1.18250	0.37709	10.37380
H	0.15960	0.78752	13.22145
H	0.79267	2.05389	12.17061
H	-1.02536	1.55289	10.51490
H	-1.68202	0.35380	11.62673
H	-1.36530	3.33048	12.22140
H	-2.02042	2.13425	13.33994
H	-3.22406	2.85559	10.62336
H	-3.84694	3.36538	12.19397
H	-3.88040	1.65971	11.74142
H	-10.24948	-9.12268	1.75388
H	-9.69187	-7.85123	2.83981
H	-11.18476	-8.73892	4.63571
H	-11.74943	-9.99790	3.53775
H	-12.62424	-8.25023	1.98917
H	-12.06599	-6.99497	3.09442
H	-14.12611	-9.14545	3.76032
H	-13.56509	-7.89491	4.87011
H	-15.01975	-7.38413	2.22969
H	-14.46266	-6.13520	3.34403
H	-15.73284	-7.25633	3.83922
H	10.13941	-9.93368	3.58135
H	10.57539	-8.42162	2.78718
H	10.36785	-11.13656	1.41288
H	10.81952	-9.62569	0.61987
H	12.36251	-11.00154	2.86442
H	12.77092	-9.42669	2.18567
H	14.19749	-11.05725	1.16228
H	13.06260	-10.49189	-0.06277
H	12.76880	-13.09671	1.51033
H	13.40606	-12.97079	-0.13037
H	11.72362	-12.56328	0.19097
H	-7.60741	9.84423	-14.91811
H	-8.21402	8.70034	-13.72219
H	-9.12853	11.60067	-14.00441
H	-9.73905	10.44930	-12.81621
H	-9.95006	10.14102	-15.84525
H	-10.55298	8.98245	-14.66082
H	-11.47935	11.87803	-14.92451
H	-12.08207	10.71867	-13.73993
H	-12.31739	10.42397	-16.77409
H	-13.62416	10.87993	-15.67862
H	-12.91954	9.26404	-15.58930
H	5.63519	2.29991	-14.89719
H	6.41134	3.65211	-15.73521

H	7.81724	1.68139	-13.88051
H	8.58966	3.03431	-14.70997
H	7.19009	0.69786	-16.08528
H	7.98085	2.04365	-16.90593
H	9.35846	0.05155	-15.05021
H	10.15145	1.39957	-15.86548
H	8.76965	-0.94480	-17.25941
H	10.51313	-0.71091	-17.11548
H	9.54852	0.40821	-18.08095
H	-3.68179	-5.97918	-17.10506
H	-2.00229	-5.45936	-16.97757
H	-3.40445	-6.26686	-19.56829
H	-1.72011	-5.76264	-19.43545
H	-2.94226	-8.19748	-18.05942
H	-1.25492	-7.69879	-17.94148
H	-2.67720	-8.48378	-20.52514
H	-0.98328	-8.01085	-20.39207
H	-2.24779	-10.44656	-19.04235
H	-0.55432	-9.97140	-18.90223
H	-1.20116	-10.48235	-20.46292
H	-8.87934	1.97299	-5.47456
H	-7.44127	2.98360	-5.60575
H	-8.85781	2.26942	-2.99599
H	-7.42913	3.29384	-3.13391
H	-10.14557	3.87657	-4.39833
H	-8.71833	4.90321	-4.53163
H	-10.13005	4.17870	-1.92512
H	-8.70726	5.21153	-2.06170
H	-11.43691	5.79422	-3.31061
H	-10.01402	6.82821	-3.44666
H	-10.77365	6.57545	-1.87363
H	2.38885	4.89531	7.43138
H	3.04934	6.07725	8.55995
H	0.49516	4.45862	8.98744
H	1.16011	5.62425	10.13156
H	0.24264	6.26658	7.29268
H	0.90577	7.43187	8.43753
H	-0.98007	6.96940	9.99922
H	-1.64448	5.80764	8.85093
H	-1.92033	7.63032	7.16590
H	-1.25671	8.79252	8.31500
H	-2.79588	7.99765	8.65381
H	1.06404	-8.30028	-7.84126
H	1.76102	-9.77875	-7.18241
H	3.33875	-7.49658	-8.45879
H	4.04362	-8.96832	-7.79028
H	2.05685	-8.86176	-10.10467
H	2.76881	-10.33066	-9.43845
H	4.32040	-8.04637	-10.73657
H	5.03891	-9.50805	-10.06046
H	3.05936	-9.43325	-12.38939
H	3.78348	-10.89332	-11.71455
H	4.79918	-9.69908	-12.52533
H	5.02477	16.40457	1.05324
H	3.56207	15.93003	0.17740
H	4.09205	17.57447	-1.60748
H	5.56600	18.03473	-0.75736
H	2.73319	18.32486	0.34741
H	4.20964	18.78701	1.19343
H	3.24482	19.96824	-1.45016
H	4.72888	20.42405	-0.61447
H	9.46808	6.63794	-10.47393
H	9.38391	5.51959	-9.11401
H	11.43476	5.33277	-11.36940
H	9.90218	4.45980	-11.33596

H	12.12173	4.18528	-9.26578
H	10.58962	3.31092	-9.24457
H	12.65946	3.13819	-11.48059
H	12.55109	2.01057	-10.12989
H	10.40304	2.45145	-12.25541
H	10.22474	1.39979	-10.84885
H	11.43976	1.04003	-12.07718
H	15.67557	1.06494	1.99266
H	16.30343	0.70612	3.59989
H	17.99448	1.44761	1.16734
H	18.62758	1.10296	2.77657
H	17.69021	-1.19890	2.65665
H	17.04594	-0.85618	1.05173
H	19.35309	-0.46399	0.20601
H	20.00094	-0.79691	1.81203
H	20.18102	-2.79916	0.35539
H	19.08475	-3.11703	1.70169
H	18.43508	-2.78461	0.09580
H	3.39277	21.17557	1.34870
H	1.90821	20.72267	0.51145
H	2.41191	22.38426	-1.28285
H	3.89918	22.83531	-0.44833
H	2.33433	23.16030	0.30058
C	3.37791	-10.72150	4.74812
H	3.96565	-9.89387	4.35658
H	2.88094	-11.20728	3.91200
H	4.05657	-11.43875	5.20516
H	1.79330	-11.09861	6.14138
H	2.88663	-9.80598	6.63735
C	-2.93824	-6.68330	4.79633
H	-3.72601	-5.99928	4.48701
H	-2.65168	-6.44283	5.81799
H	-3.33238	-7.69718	4.78557
H	-2.03870	-6.77792	2.83736
H	-1.35656	-5.53871	3.89681
C	-9.43565	-6.98965	-5.41095
H	-9.86940	-6.67869	-6.35917
H	-10.11048	-6.69996	-4.60795
H	-9.35288	-8.07451	-5.42156
H	-8.18805	-5.25247	-5.24467
H	-7.66213	-6.60112	-4.23671
C	-3.05787	-8.24026	-9.08232
H	-2.35644	-8.27191	-9.91393
H	-3.18296	-9.25609	-8.71552
H	-2.62371	-7.63299	-8.29099
H	-4.15687	-6.71190	-10.09346
H	-4.84597	-8.32551	-10.26633
C	1.08429	9.47005	-8.63143
H	1.90090	9.26803	-7.94080
H	1.22623	8.86169	-9.52206
H	0.14808	9.17725	-8.16076
H	0.23244	11.12189	-9.70093
H	0.85958	11.54389	-8.10458
C	7.07839	11.14981	-11.55473
H	7.20668	10.42424	-10.75424
H	8.06187	11.44071	-11.91814
H	6.54137	10.66441	-12.36637
H	6.89808	12.88393	-10.28456
H	6.18901	13.07458	-11.89123
C	7.53699	7.59259	-0.94626
H	6.63006	7.46312	-1.53327
H	7.73298	6.67284	-0.39849
H	8.36665	7.77293	-1.62618
H	7.18106	9.67397	-0.55050
H	6.52670	8.58148	0.67550

C	13.40293	8.57700	2.64251
H	14.27179	8.67852	3.28954
H	13.61779	9.06854	1.69652
H	13.24562	7.51905	2.44420
H	12.01341	8.73404	4.28426
H	12.36333	10.26159	3.47005
H	11.48934	-1.20609	6.96088
H	7.68651	0.52996	7.90389
H	12.35207	0.97719	6.27480
H	7.57261	6.33800	6.38514
H	7.96282	8.15191	4.76103
H	11.65488	6.37067	3.54840
H	11.10562	8.84947	-1.68431
H	8.34239	11.85215	-0.41497
H	10.72272	9.43438	-4.05171
H	5.63254	14.38310	-5.23316
H	4.42601	14.17134	-7.37060
H	6.94246	10.92115	-8.54904
H	0.22685	11.91736	-11.82266
H	3.18698	8.85490	-12.11784
H	-1.46276	10.44379	-12.84864
H	0.65026	3.67879	-13.94222
H	-1.10489	1.97547	-13.66054
H	-4.09255	5.00990	-13.27664
H	7.86580	7.64394	2.37568
H	11.68848	10.33518	0.93743
H	3.09424	12.01049	-7.69997
H	4.22595	11.39626	-12.41636
H	10.25005	-2.76304	6.14697
H	7.38489	-1.27382	8.96454
H	9.00714	-4.85899	5.93900
H	2.53365	-4.94604	9.06866
H	0.90119	-6.46334	8.03170
H	3.87893	-7.91611	5.32731
H	0.95329	-8.86342	1.46405
H	-3.04288	-9.27471	2.92057
H	0.16676	-8.99799	-0.87446
H	-6.92995	-9.42974	-1.86801
H	-7.69750	-8.85155	-4.11415
H	-3.67832	-8.07106	-5.35364
H	-6.09668	-6.47070	-11.33021
H	-6.34360	-3.66889	-8.10342
H	-5.56028	-4.66575	-12.89673
H	-5.47717	1.93137	-10.06657
H	-4.22973	3.65779	-11.30196
H	-3.27186	0.86928	-14.39894
H	0.09133	-6.15217	5.75148
H	0.67477	-10.56182	3.72573
H	-4.66021	-8.87555	-7.19030
H	-7.88801	-5.25611	-7.59170
H	3.30122	6.71331	-11.38149
H	5.37616	5.43687	-11.59120
H	4.15141	4.06846	-15.44723
H	2.09555	5.36667	-15.25022
H	-2.81332	9.35394	-14.75318
H	-4.85728	10.70312	-14.52675
H	-6.26996	8.05118	-11.49850
H	-4.22238	6.71881	-11.70332
H	8.90542	8.44606	-5.32658
H	10.06779	6.68780	-6.59168
H	10.60649	9.38790	-9.85079
H	9.46846	11.15380	-8.57889
H	5.04753	13.06705	-3.25690
H	3.71885	14.62371	-1.89443
H	7.24588	15.80603	0.19485

H	8.57774	14.26398	-1.17445
H	13.51024	3.25589	6.60710
H	15.70183	2.69679	5.64238
H	14.24367	3.69353	1.75888
H	12.05060	4.23426	2.71497
H	7.71248	5.10806	8.75852
H	5.46432	5.46783	9.68660
H	4.01478	2.12320	7.46841
H	6.25487	1.76300	6.54018
H	4.26463	-4.25543	10.79117
H	2.92484	-2.80974	12.26107
H	3.88755	0.55824	9.82435
H	5.17997	-0.88998	8.32835
H	7.25096	-5.22223	4.27514
H	8.39644	-6.48317	2.50296
H	7.24770	-10.14599	4.36396
H	6.10976	-8.88835	6.14148
H	-2.28364	-1.27245	-14.34861
H	-1.71949	-2.40878	-16.44802
H	-5.65953	-4.03328	-16.63684
H	-6.23425	-2.88091	-14.54239
H	-8.04527	-1.84992	-8.22188
H	-8.52379	-0.82122	-6.04398
H	-4.91260	1.43477	-6.31147
H	-4.41711	0.38742	-8.47795
H	-6.19460	-11.06323	-0.14233
H	-8.25045	-11.27253	1.19111
H	-6.91575	-8.12367	3.74149
H	-4.87656	-7.89403	2.39667
H	-0.40867	-10.36136	-2.91885
H	1.62600	-10.01947	-4.25709
H	-0.00420	-6.39358	-5.80533
H	-2.02215	-6.71572	-4.45110

*Table 4 Cartesian coordinates of **1g** (GFN2-xTB(GBSA(toluene)). B97-3c(COSMO(toluene) single point energy = 10838.55638 Hartree.*

	X	Y	Z
C	8.82130	-0.53353	6.27501
C	7.94315	-0.24092	7.31905
C	7.90402	1.06237	7.83554
C	8.66948	2.04673	7.24794
C	9.47920	1.74919	6.12022
C	9.58919	0.45029	5.66842
C	9.91934	3.01614	5.58748
C	9.37043	4.06289	6.44107
C	10.46787	3.55554	4.46109
C	9.66850	5.32730	5.79752
C	8.65061	3.50382	7.45807
C	9.41579	6.64518	6.12017
C	9.64980	7.63046	5.16446
C	10.15609	7.31300	3.91146
C	10.53109	5.99608	3.62744
C	10.28191	5.00870	4.55500
C	8.79694	11.78002	-0.75193
C	9.60273	10.64767	-0.73214
C	10.09344	10.12148	-1.93031
C	9.68523	10.67698	-3.12476
C	8.77532	11.76655	-3.13634
C	8.37962	12.35097	-1.95025
C	8.33340	11.90996	-4.50696
C	9.02222	10.89298	-5.29223
C	7.34458	12.49059	-5.24681
C	8.43536	10.91124	-6.61435
C	9.85729	10.16805	-4.49269
C	8.66741	10.18389	-7.76322
C	7.76606	10.28775	-8.81623
C	6.66023	11.12915	-8.74840
C	6.49897	11.96373	-7.63369
C	7.36543	11.84619	-6.56881
C	1.02244	10.15198	-12.22981
C	2.28720	9.59302	-12.08750
C	2.53417	8.30609	-12.57726
C	1.49172	7.57105	-13.09823
C	0.17660	8.10605	-13.12285
C	-0.04131	9.41531	-12.74351
C	-0.70176	7.00150	-13.44591
C	0.13824	5.82425	-13.63684
C	-2.01672	6.63919	-13.40411
C	-0.74634	4.69116	-13.77799
C	1.45219	6.14906	-13.46229
C	-0.53600	3.34348	-13.98497
C	-1.60837	2.47209	-13.85623
C	-2.89511	2.92290	-13.55944
C	-3.12888	4.30611	-13.49608
C	-2.06599	5.17865	-13.57618
C	10.10383	8.27498	2.78469
C	8.97629	8.16222	1.97881
C	8.79097	8.94333	0.85268
C	9.78411	9.86999	0.51696
C	10.89965	9.99845	1.33116
C	11.07774	9.21760	2.46917
C	5.55173	10.97385	-9.71042
C	4.31544	10.66893	-9.14306
C	3.21713	10.28988	-9.89228
C	3.35863	10.21994	-11.28315
C	4.56313	10.60257	-11.85727
C	5.66737	10.98135	-11.10186
C	1.96666	9.85619	-9.17620
C	6.93432	11.39549	-11.80266
C	12.32213	9.35450	3.30169
C	7.56187	8.72990	0.01093
C	5.62772	-0.86566	7.91352
C	6.95124	-1.25426	7.70474
C	7.26534	-2.62087	7.68753
C	6.25086	-3.54969	7.76599
C	4.89545	-3.13213	7.83619
C	4.59582	-1.79233	7.97872
C	4.09289	-4.31592	7.63216
C	5.01070	-5.44607	7.51621
C	2.81476	-4.71823	7.37875
C	4.21186	-6.61275	7.20256
C	6.30087	-5.01108	7.60565
C	4.50974	-7.93411	6.93369
C	3.51256	-8.75773	6.42115
C	2.22591	-8.28360	6.18517
C	1.89719	-6.97534	6.55751
C	2.87449	-6.15375	7.07372
C	-1.49032	-11.44913	1.57146
C	-1.50795	-10.32404	2.38810
C	-2.46097	-9.32418	2.15987
C	-3.29079	-9.41718	1.06504
C	-3.15156	-10.49120	0.14687
C	-2.30061	-11.53736	0.44316
C	-3.97156	-10.15066	-0.99722
C	-4.66372	-8.90659	-0.67491
C	-4.21674	-10.49988	-2.29364
C	-5.41715	-8.52088	-1.84727

C	-4.27052	-8.45176	0.54918	C	6.03804	15.63736	-3.71403
C	-6.23793	-7.44983	-2.13601	C	6.90094	14.64809	-4.14105
C	-6.63632	-7.24942	-3.45171	C	10.23369	7.98569	-5.60718
C	-6.22118	-8.09877	-4.47443	C	11.04512	6.90842	-5.90245
C	-5.47420	-9.24038	-4.16535	C	12.35364	6.84763	-5.43478
C	-5.09722	-9.46559	-2.85779	C	12.82715	7.89802	-4.65686
C	-5.82607	-5.80308	-10.75978	C	12.01865	8.97418	-4.34729
C	-6.33319	-5.43958	-9.51727	C	3.72650	16.58745	-3.42810
C	-6.71575	-4.11314	-9.28908	C	12.06858	1.87629	3.62916
C	-6.48361	-3.16667	-10.26259	C	12.69134	1.19960	2.59900
C	-5.84283	-3.52614	-11.47771	C	12.36809	1.46231	1.27246
C	-5.57079	-4.85286	-11.74457	C	11.40177	2.42712	1.00756
C	-5.49508	-2.28279	-12.13273	C	10.78121	3.11539	2.03115
C	-4.70553	-1.78536	-13.12838	C	13.01459	0.70661	0.14792
C	-5.47507	0.03377	-11.84484	C	7.10154	5.25546	8.28259
C	-5.59873	1.35957	-11.48372	C	6.41699	5.87060	9.31162
C	-4.80439	2.30257	-12.12045	C	6.53104	5.41499	10.62076
C	-3.91451	1.95380	-13.13696	C	7.34543	4.31586	10.86943
C	-3.90057	0.62734	-13.59768	C	8.02507	3.68774	9.84437
C	-4.64833	-0.32661	-12.94323	C	5.82972	6.12312	11.74256
C	1.26647	-9.03932	5.35613	C	7.72472	-6.69854	6.48015
C	1.69657	-9.36644	4.07244	C	8.91037	-7.39838	6.37594
C	0.84451	-9.85817	3.09876	C	9.94147	-7.20010	7.28797
C	-0.50060	-10.05176	3.43414	C	9.75425	-6.27197	8.30609
C	-0.90822	-9.81634	4.74178	C	8.57576	-5.55943	8.41117
C	-0.05594	-9.30852	5.71334	C	1.53769	-2.73847	6.61002
C	-6.37246	-7.65350	-5.87434	C	0.35401	-2.03491	6.50599
C	-5.18406	-7.41855	-6.56064	C	-0.81204	-2.50191	7.10300
C	-5.14205	-6.77414	-7.78470	C	-0.75631	-3.69148	7.82062
C	-6.34958	-6.34874	-8.35133	C	0.42023	-4.40761	7.91988
C	-7.54130	-6.65833	-7.70805	C	-2.09266	-1.72607	7.00276
C	-7.58108	-7.31145	-6.48258	C	11.24520	-7.92908	7.14304
C	-3.79947	-6.43535	-8.37369	C	-2.65846	-2.30725	-14.43492
C	-8.90991	-7.67775	-5.88086	C	-2.01216	-3.02696	-15.42038
C	-0.57624	-9.06941	7.10618	C	-2.68935	-3.98058	-16.17223
C	1.37076	-10.02683	1.69892	C	-4.03520	-4.20228	-15.90138
C	2.63202	5.30198	-13.52854	C	-4.68615	-3.49455	-14.91051
C	-3.16845	7.49810	-13.18123	C	-6.59005	0.06777	-8.44239
C	-4.00908	-2.52786	-14.16642	C	-7.19544	0.72109	-7.38716
C	-7.22084	-1.00869	-9.06514	C	-8.44553	0.32669	-6.92285
C	-3.68923	-11.62325	-3.05079	C	-9.07076	-0.75045	-7.54113
C	-4.63886	-7.23384	1.25355	C	-8.46800	-1.41593	-8.59013
C	1.58709	-3.94144	7.31396	C	-9.12570	1.07483	-5.81401
C	7.53908	-5.76703	7.50132	C	-1.99311	-4.71858	-17.27773
C	11.10673	2.85101	3.36150	C	-4.94790	-7.26620	2.61351
C	7.92206	4.15603	8.53410	C	-5.26726	-6.10476	3.28911
C	6.41917	13.52598	-4.81692	C	-5.27349	-4.87648	2.63814
C	10.71020	9.03842	-4.82631	C	-4.96665	-4.84613	1.28184
C	-6.59718	-1.70532	-10.17855	C	-4.65615	-6.00371	0.59645
C	-5.97416	-1.19898	-11.28194	C	-4.51970	-12.38672	-3.87120
C	2.65656	4.05295	-12.90859	C	-4.01204	-13.45796	-4.58014
C	3.78524	3.26006	-12.97188	C	-2.66470	-13.79267	-4.50526
C	4.92065	3.68311	-13.65441	C	-1.83544	-13.02124	-3.69801
C	4.89595	4.93010	-14.26917	C	-2.33639	-11.95579	-2.97751
C	3.77462	5.73330	-14.20321	C	-2.10681	-14.92725	-5.31359
C	6.12721	2.79749	-13.76352	C	-5.64212	-3.61872	3.36927
C	-3.31583	8.67959	-13.90857	C	12.20188	-7.14268	6.23703
C	-4.40508	9.50191	-13.69996	C	13.54388	-7.85066	6.06730
C	-5.37237	9.18188	-12.75332	C	14.48960	-7.06953	5.15706
C	-5.21810	8.01021	-12.02096	C	15.83652	-7.76907	4.98458
C	-4.13690	7.17711	-12.23055	C	16.77021	-6.98634	4.06803
C	-6.57516	10.05688	-12.55502	C	-7.13995	-3.32071	3.22151
C	5.04653	13.42487	-5.04146	C	-7.54359	-2.04816	3.96258
C	4.18917	14.41517	-4.60381	C	-9.02929	-1.73466	3.79708
C	4.66940	15.53802	-3.93927	C	-9.43700	-0.46530	4.54284

C	-10.91789	-0.14995	4.36436
C	-2.19738	-0.70120	8.14010
C	-3.46995	0.14560	8.07252
C	-4.73013	-0.63500	8.44392
C	-6.01397	0.18626	8.30751
C	-6.11711	1.32704	9.31426
C	-7.71582	9.62881	-13.48780
C	-8.96729	10.48008	-13.28717
C	-10.10105	10.06014	-14.22060
C	-11.36644	10.88729	-14.00099
C	-12.49133	10.46780	-14.94079
C	6.03378	1.90661	-15.00937
C	7.25441	1.00121	-15.15676
C	7.16666	0.11899	-16.40057
C	8.39204	-0.77932	-16.55923
C	8.29893	-1.65462	-17.80408
C	-2.06973	-3.92403	-18.58823
C	-1.35333	-4.63703	-19.73251
C	-1.41957	-3.83962	-21.03363
C	-0.69706	-4.54408	-22.18044
C	-0.75930	-3.73809	-23.47314
C	-9.98798	2.21027	-6.38129
C	-10.70612	2.98857	-5.28149
C	-11.56430	4.11820	-5.84771
C	-12.29054	4.89702	-4.75251
C	-13.14414	6.02301	-5.32522
C	6.72331	7.23444	12.31042
C	6.10813	7.95126	13.51283
C	4.88270	8.79250	13.15951
C	4.37347	9.59086	14.35837
C	3.14557	10.42375	14.00724
C	-1.63891	-14.43270	-6.68871
C	-1.03956	-15.55856	-7.52789
C	-0.57958	-15.06710	-8.89900
C	0.03459	-16.18590	-9.73828
C	0.48771	-15.68772	-11.10610
C	13.24488	5.69657	-5.79932
C	13.93165	5.95106	-7.14775
C	14.84813	4.79476	-7.54180
C	15.61037	5.04155	-8.84503
C	3.20334	16.20788	-2.03637
C	2.24279	17.25579	-1.47956
C	1.70266	16.86283	-0.10592
C	0.74229	17.90811	0.45817
C	0.20057	17.50473	1.82500
C	12.08698	-0.37831	-0.41976
C	11.79998	-1.48093	0.59682
C	10.80010	-2.52424	0.09518
C	11.33942	-3.39612	-1.03799
C	10.34807	-4.48637	-1.43025
C	14.71816	5.03858	-10.08546
C	15.53393	5.18563	-11.36528
H	6.20570	2.16426	-12.87704
H	7.03097	3.40729	-13.82901
H	-6.31729	11.09794	-12.76115
H	-6.91726	9.98800	-11.51998
H	2.88009	16.69195	-4.11063
H	4.23782	17.55049	-3.36754
H	13.26758	1.40294	-0.65553
H	13.93735	0.23948	0.49785
H	4.89801	6.55551	11.37571
H	5.59287	5.41536	12.53995
H	-2.93725	-2.41444	7.05151
H	-2.13277	-1.19977	6.04641
H	11.70919	-8.06137	8.12282

H	11.07393	-8.91681	6.70941
H	-8.38009	1.49645	-5.13638
H	-9.76212	0.39582	-5.24230
H	-2.45733	-5.69610	-17.42456
H	-0.94378	-4.87395	-17.01701
H	-2.86900	-15.69719	-5.45156
H	-1.25972	-15.37506	-4.78925
H	-5.07117	-2.77732	2.97008
H	-5.40141	-3.72208	4.42941
H	12.36445	-6.15049	6.66521
H	11.73514	-7.00579	5.25845
H	14.01097	-7.98086	7.04683
H	13.37773	-8.84559	5.64640
H	14.65262	-6.07333	5.57620
H	14.02263	-6.94181	4.17707
H	15.67425	-8.76697	4.56960
H	16.30767	-7.89110	5.96313
H	17.72266	-7.50103	3.96092
H	16.32813	-6.87275	3.08006
H	16.95953	-5.99433	4.47355
H	-7.71353	-4.16660	3.60845
H	-7.38118	-3.21805	2.16076
H	-6.95419	-1.20826	3.58577
H	-7.31513	-2.16089	5.02524
H	-9.61997	-2.57606	4.16849
H	-9.25691	-1.61674	2.73449
H	-9.21914	-0.58652	5.60665
H	-8.84091	0.37458	4.17707
H	-11.53045	-0.96638	4.74214
H	-11.15397	-0.00494	3.31188
H	-11.18387	0.75657	4.90393
H	-1.32757	-0.04117	8.08960
H	-2.15764	-1.22301	9.09935
H	-3.35619	0.98661	8.75903
H	-3.58010	0.55189	7.06383
H	-4.81570	-1.50869	7.79468
H	-4.64057	-0.99391	9.47259
H	-6.07667	0.59074	7.29423
H	-6.86739	-0.48213	8.44986
H	-6.01453	0.94939	10.32987
H	-5.34434	2.07232	9.14382
H	-7.08443	1.81802	9.22782
H	-7.38035	9.70985	-14.52453
H	-7.95558	8.57919	-13.30091
H	-8.72359	11.53057	-13.46495
H	-9.30241	10.39044	-12.25065
H	-9.77446	10.17006	-15.25781
H	-10.32943	9.00373	-14.05787
H	-11.13819	11.94442	-14.15824
H	-11.69751	10.77125	-12.96590
H	-12.19159	10.60082	-15.97853
H	-13.38312	11.06485	-14.76176
H	-12.74365	9.41994	-14.78992
H	5.13046	1.29510	-14.94515
H	5.93991	2.53979	-15.89506
H	7.34438	0.36774	-14.27055
H	8.15576	1.61676	-15.21463
H	6.26983	-0.50281	-16.33986
H	7.06848	0.75256	-17.28583
H	8.49034	-1.41526	-15.67581
H	9.28905	-0.15792	-16.61992
H	7.42264	-2.29802	-17.75401
H	9.18173	-2.28361	-17.89700
H	8.22012	-1.03943	-18.69837
H	-1.62175	-2.93911	-18.43539

H	-3.11874	-3.77037	-18.85289
H	-0.30667	-4.79583	-19.46037
H	-1.80635	-5.61954	-19.88752
H	-0.97051	-2.85557	-20.87638
H	-2.46575	-3.68352	-21.30884
H	0.34793	-4.70420	-21.90337
H	-1.14945	-5.52551	-22.34316
H	-0.29631	-2.76260	-23.33712
H	-1.79260	-3.58590	-23.77897
H	-0.23712	-4.25728	-24.27403
H	-10.72420	1.78937	-7.07048
H	-9.35240	2.88987	-6.95418
H	-11.34022	2.30575	-4.71030
H	-9.96767	3.40675	-4.59261
H	-12.29989	3.70018	-6.53972
H	-10.92989	4.80314	-6.41600
H	-12.92654	4.21296	-4.18517
H	-11.55589	5.31564	-4.06008
H	-13.89728	5.62458	-6.00230
H	-12.52571	6.72695	-5.87868
H	-13.65047	6.56354	-4.52827
H	7.67594	6.79150	12.61265
H	6.93482	7.96123	11.52238
H	5.83525	7.21489	14.27289
H	6.86725	8.60665	13.94846
H	5.14079	9.48293	12.35231
H	4.07948	8.14575	12.80144
H	5.16764	10.25053	14.71716
H	4.12459	8.90268	15.17012
H	2.80291	10.98580	14.87347
H	2.33284	9.78299	13.67050
H	3.37759	11.12832	13.21082
H	-0.89431	-13.64483	-6.55056
H	-2.48763	-13.99341	-7.21861
H	-0.18862	-15.99194	-6.99612
H	-1.78331	-16.34872	-7.65868
H	0.15684	-14.27010	-8.76784
H	-1.43225	-14.64190	-9.43450
H	0.88990	-16.60851	-9.20515
H	-0.70018	-16.98432	-9.86865
H	1.23491	-14.90385	-10.99826
H	-0.35472	-15.28185	-11.66277
H	0.92278	-16.49956	-11.68510
H	14.00682	5.55762	-5.02924
H	12.65695	4.77842	-5.86782
H	14.51540	6.87267	-7.08314
H	13.16419	6.09678	-7.90965
H	14.25714	3.88038	-7.63796
H	15.57321	4.63494	-6.73918
H	16.36569	4.25924	-8.95916
H	16.13504	5.99812	-8.78034
H	2.69488	15.24254	-2.09711
H	4.05038	16.09064	-1.35588
H	1.40682	17.38441	-2.17186
H	2.75874	18.21630	-1.40358
H	1.18466	15.90350	-0.18352
H	2.53803	16.73048	0.58635
H	-0.09077	18.04364	-0.23605
H	1.26096	18.86632	0.54244
H	-0.33941	16.56211	1.75864
H	-0.47897	18.26374	2.20694
H	1.01463	17.38263	2.53692
H	11.14454	0.08051	-0.72772
H	12.55921	-0.80676	-1.30548
H	12.73524	-1.97928	0.86473

H	11.39656	-1.02259	1.50327
H	9.88978	-2.01934	-0.23828
H	10.52623	-3.17250	0.93208
H	12.27759	-3.85809	-0.72033
H	11.55072	-2.77767	-1.91199
H	10.13563	-5.13393	-0.58165
H	10.74882	-5.09716	-2.23661
H	9.41085	-4.04658	-1.76602
H	14.15522	4.10238	-10.12110
H	13.99969	5.85804	-10.02624
H	16.24292	4.36577	-11.46402
H	16.09197	6.11996	-11.35634
H	14.88314	5.18342	-12.23728
C	-1.66930	-7.99974	7.14877
H	-1.29616	-7.05120	6.76885
H	-2.00957	-7.85260	8.17186
H	-2.52240	-8.29381	6.54201
H	-0.98950	-10.00694	7.49068
H	0.24782	-8.77552	7.75879
C	1.14530	-8.75021	0.88496
H	1.55641	-8.86142	-0.11627
H	1.62624	-7.90123	1.36703
H	0.08116	-8.53916	0.80058
H	0.87905	-10.85707	1.19413
H	2.44144	-10.23921	1.74188
C	-9.45534	-8.94780	-6.53956
H	-9.57268	-8.80209	-7.61139
H	-10.42251	-9.20982	-6.11560
H	-8.77030	-9.77809	-6.37992
H	-9.62120	-6.86207	-6.03197
H	-8.80666	-7.85252	-4.80969
C	-3.33962	-5.05625	-7.89446
H	-2.35619	-4.82311	-8.29720
H	-3.28736	-5.02930	-6.80773
H	-4.03820	-4.28986	-8.22413
H	-3.83930	-6.43436	-9.46222
H	-3.07036	-7.18543	-8.05876
C	2.00108	8.35243	-8.89292
H	2.87474	8.09717	-8.29629
H	2.04577	7.79317	-9.82515
H	1.10757	8.04889	-8.35130
H	1.08141	10.08250	-9.76998
H	1.89037	10.39613	-8.22952
C	7.56984	10.24983	-12.59295
H	7.80454	9.41255	-11.93913
H	8.49034	10.58622	-13.06563
H	6.89536	9.89289	-13.36764
H	7.65007	11.77606	-11.07203
H	6.69943	12.20880	-12.49609
C	7.77840	7.60383	-1.00253
H	6.86988	7.43187	-1.57605
H	8.04907	6.68115	-0.49310
H	8.57693	7.86397	-1.69343
H	7.30037	9.64509	-0.52233
H	6.72614	8.46405	0.66296
C	13.42733	8.42744	2.79031
H	14.33201	8.55475	3.38118
H	13.65704	8.64781	1.74976
H	13.11212	7.38882	2.86151
H	12.09905	9.10227	4.34052
H	12.67492	10.38777	3.26537
H	8.84890	-1.53659	5.87485
H	7.24497	1.29719	8.65886
H	10.21146	0.20949	4.81922
H	9.00371	6.91162	7.08231

H	9.38970	8.65711	5.38247
H	10.96473	5.76507	2.66532
H	8.44039	12.18565	0.18490
H	10.72627	9.24499	-1.90992
H	7.70332	13.19316	-1.94498
H	9.50682	9.50776	-7.83222
H	7.89268	9.65354	-9.68028
H	5.67535	12.66224	-7.60890
H	0.85405	11.16658	-11.89549
H	3.51981	7.87526	-12.46853
H	-1.03039	9.84754	-12.78569
H	0.45119	2.96179	-14.19954
H	-1.42230	1.41125	-13.93000
H	-4.13377	4.67734	-13.36110
H	8.22235	7.43250	2.24462
H	11.65655	10.72565	1.06857
H	4.23012	10.67265	-8.06476
H	4.64645	10.58616	-12.93560
H	5.38587	0.18674	7.94797
H	8.29203	-2.93833	7.57561
H	3.57414	-1.46046	8.09061
H	5.50917	-8.31801	7.07681
H	3.75015	-9.78035	6.16303
H	0.90487	-6.59668	6.36438
H	-0.81470	-12.26119	1.80200
H	-2.49179	-8.46069	2.80932
H	-2.23759	-12.40119	-0.20236
H	-6.53099	-6.75109	-1.36645
H	-7.22009	-6.37423	-3.70068
H	-5.16420	-9.90722	-4.95764
H	-5.59109	-6.84112	-10.95123
H	-7.11665	-3.83284	-8.32490
H	-5.11533	-5.14859	-12.67835
H	-6.26722	1.65922	-10.69033
H	-4.83797	3.32426	-11.77336
H	-3.28783	0.35759	-14.44477
H	2.72714	-9.16612	3.81093
H	-1.93928	-10.01547	5.00094
H	-4.25291	-7.69954	-6.08619
H	-8.47184	-6.35208	-8.16754
H	1.79095	3.72314	-12.35272
H	3.78859	2.29779	-12.47867
H	5.76965	5.27695	-14.80340
H	3.76764	6.69387	-14.69822
H	-2.57543	8.93474	-14.65335
H	-4.50672	10.41022	-14.27767
H	-5.95335	7.75092	-11.27177
H	-4.01531	6.28837	-11.62851
H	4.64945	12.54681	-5.52903
H	3.12696	14.31639	-4.78015
H	6.43126	16.50272	-3.19862
H	7.96423	14.74354	-3.97262
H	9.21131	8.00848	-5.95533
H	10.65600	6.09614	-6.50075
H	13.84169	7.86897	-4.28410
H	12.40563	9.78743	-3.74991
H	12.33730	1.67073	4.65570
H	13.44147	0.45538	2.82768
H	11.13375	2.64319	-0.01751
H	10.01376	3.84082	1.80340
H	6.98594	5.60508	7.26715
H	5.77960	6.71726	9.09688
H	7.44595	3.94631	11.88059
H	8.66538	2.84377	10.05755
H	6.93840	-6.84771	5.75435
H	9.04014	-8.11162	5.57360
H	10.54334	-6.10658	9.02652
H	8.43998	-4.85375	9.21834
H	2.42957	-2.37856	6.11765
H	0.33071	-1.11226	5.94259
H	-1.65050	-4.06573	8.29955
H	0.44867	-5.32621	8.48782
H	-2.10811	-1.59435	-13.83836
H	-0.96199	-2.85038	-15.60764
H	-4.57790	-4.94175	-16.47384
H	-5.73478	-3.66906	-14.71574
H	-5.60832	0.36924	-8.77730
H	-6.68817	1.54916	-6.91155
H	-10.04200	-1.07311	-7.19235
H	-8.97355	-2.24282	-9.06803
H	-4.95924	-8.21404	3.13230
H	-5.50873	-6.15023	4.34206
H	-4.96210	-3.90001	0.75848
H	-4.39413	-5.96163	-0.45086
H	-5.57344	-12.15329	-3.92510
H	-4.67257	-14.04771	-5.20061
H	-0.78335	-13.26214	-3.63284
H	-1.67909	-11.35455	-2.36629

*Table 5 Cartesian coordinates of **1j** (GFN2-xTB(GBSA(toluene)). B97-3c(COSMO(toluene) single point energy = 10838.55244 Hartree.*

	X	Y	Z
C	5.48860	1.57547	8.79513
C	6.19110	0.38003	8.64318
C	7.50203	0.42721	8.14090
C	8.00647	1.61677	7.66853
C	7.26026	2.81927	7.79121
C	6.02137	2.79672	8.39995
C	7.99807	3.83238	7.07104
C	9.19293	3.19360	6.53323
C	7.84395	5.07819	6.53333
C	9.79095	4.12734	5.60497
C	9.22606	1.87674	6.88957
C	10.86654	4.04199	4.74558
C	11.06050	5.05242	3.81026
C	10.21532	6.15501	3.74362
C	9.17668	6.28444	4.67589
C	8.94775	5.27155	5.58173
C	10.64332	8.42251	-2.24113
C	10.04245	9.09058	-1.17957
C	9.27866	10.23593	-1.42471
C	9.03817	10.61466	-2.72933
C	9.52596	9.82873	-3.80675
C	10.38917	8.78054	-3.56044
C	8.87324	10.32477	-4.99902
C	8.07306	11.47917	-4.60396
C	8.62483	10.00382	-6.30153
C	7.34083	11.91145	-5.77556
C	8.15681	11.66712	-3.25496
C	6.42322	12.91621	-6.00900
C	5.71320	12.90998	-7.20437
C	5.91484	11.92429	-8.16570
C	6.92194	10.97203	-7.97603
C	7.64079	10.97538	-6.80012
C	0.70178	10.92463	-12.22185
C	1.98404	10.41698	-12.04301
C	2.34523	9.21919	-12.67293

C	1.38263	8.48226	-13.32761	C	-5.16431	-3.12064	-12.67491
C	0.03992	8.94652	-13.37875	C	-5.14664	-4.39791	-13.19793
C	-0.27887	10.19534	-12.88602	C	-4.89677	-1.79595	-13.20015
C	-0.75028	7.85035	-13.89513	C	-4.32404	-1.15306	-14.26144
C	0.17583	6.77726	-14.23188	C	-4.71260	0.44904	-12.58290
C	-2.03416	7.38785	-13.91111	C	-4.75888	1.70564	-12.01700
C	-0.61544	5.59716	-14.49319	C	-4.13817	2.75223	-12.68013
C	1.45491	7.13993	-13.92257	C	-3.48118	2.56243	-13.89607
C	-0.28684	4.28933	-14.78561	C	-3.54742	1.31283	-14.52798
C	-1.27613	3.31774	-14.70161	C	-4.16111	0.26055	-13.87880
C	-2.58471	3.63761	-14.34526	C	0.45163	-8.64814	5.02577
C	-2.94382	4.98423	-14.18473	C	-0.67833	-7.89896	4.70496
C	-1.96741	5.95430	-14.23582	C	-1.61869	-8.31823	3.77869
C	10.25126	7.06250	2.57941	C	-1.41111	-9.54245	3.13597
C	9.06105	7.15266	1.85936	C	-0.36012	-10.35005	3.55557
C	8.95626	7.84241	0.66621	C	0.56976	-9.94034	4.50014
C	10.09845	8.48002	0.16742	C	-4.70287	-7.93873	-7.65589
C	11.27053	8.44041	0.90829	C	-4.02789	-8.14885	-8.86065
C	11.37416	7.74489	2.10876	C	-4.24699	-7.40446	-10.00736
C	4.93888	11.73542	-9.25854	C	-5.24137	-6.41165	-9.96400
C	3.63483	11.46557	-8.85145	C	-6.01981	-6.31737	-8.81199
C	2.63591	11.08758	-9.73078	C	-5.77043	-7.03401	-7.65215
C	2.95102	11.00597	-11.09398	C	-3.38210	-7.60937	-11.22593
C	4.23444	11.34132	-11.50826	C	-6.62119	-6.73051	-6.44874
C	5.24213	11.69559	-10.61906	C	1.65342	-10.89464	4.93204
C	1.31253	10.65406	-9.15840	C	-2.78997	-7.42675	3.47022
C	6.61752	12.01571	-11.14027	C	2.67102	6.37540	-14.14824
C	12.67664	7.76860	2.86387	C	-3.24499	8.13651	-13.61372
C	7.65771	7.79309	-0.09262	C	-3.92979	-1.73101	-15.53593
C	4.41819	-1.10709	9.64182	C	-5.64657	-0.99734	-9.61896
C	5.50801	-0.91131	8.79400	C	-6.13254	-10.11999	-2.60961
C	5.87429	-1.95266	7.92740	C	0.00327	-9.23491	-2.07191
C	5.10163	-3.08536	7.85126	C	0.97542	-4.93472	9.13627
C	3.93869	-3.23395	8.65414	C	6.34395	-4.45215	6.03823
C	3.63773	-2.25762	9.58437	C	6.77859	6.02790	6.80795
C	3.30003	-4.45937	8.21683	C	10.19202	0.86115	6.50284
C	4.17673	-5.05664	7.21503	C	9.11373	8.87693	-7.07904
C	2.14385	-5.18251	8.30805	C	7.47747	12.64280	-2.41705
C	3.48923	-6.19796	6.66299	C	-5.40735	-1.51977	-10.95428
C	5.24761	-4.25073	6.97274	C	-5.09295	-0.85572	-12.10233
C	3.78264	-7.04439	5.61490	C	3.64360	6.20483	-13.16467
C	2.79450	-7.90706	5.16315	C	4.80209	5.50175	-13.43780
C	1.52823	-7.94125	5.74409	C	5.03252	4.95872	-14.69584
C	1.25614	-7.12817	6.85208	C	4.05203	5.11310	-15.67109
C	2.22964	-6.26537	7.31332	C	2.88731	5.80180	-15.40318
C	-3.47942	-10.14760	1.77150	C	6.28894	4.19588	-15.00000
C	-2.12438	-9.86704	1.88112	C	-3.46520	9.37080	-14.22622
C	-1.35121	-9.73813	0.72064	C	-4.60760	10.09725	-13.95544
C	-1.95936	-9.81751	-0.51018	C	-5.55814	9.62619	-13.05644
C	-3.35781	-10.04443	-0.61637	C	-5.33156	8.40234	-12.43690
C	-4.10333	-10.24460	0.52818	C	-4.19656	7.66456	-12.71045
C	-3.68739	-9.87338	-2.01847	C	-6.81725	10.39814	-12.79106
C	-2.45464	-9.50099	-2.70126	C	9.14444	7.59802	-6.52134
C	-4.74020	-9.80968	-2.88715	C	9.57245	6.51806	-7.26525
C	-2.80107	-9.19094	-4.06671	C	9.98259	6.67458	-8.58582
C	-1.40478	-9.50903	-1.83213	C	9.95816	7.94947	-9.13815
C	-2.05973	-8.75399	-5.14370	C	9.52919	9.03670	-8.40057
C	-2.72728	-8.39202	-6.30306	C	6.11219	12.88429	-2.56776
C	-4.11260	-8.51025	-6.43098	C	5.46659	13.79444	-1.75486
C	-4.84778	-9.05875	-5.37055	C	6.15936	14.49486	-0.77319
C	-4.20640	-9.35343	-4.18277	C	7.52142	14.25684	-0.62783
C	-5.27566	-5.48991	-12.34113	C	8.17294	13.33996	-1.42928
C	-5.36512	-5.33194	-10.95970	C	10.38911	5.47826	-9.39567
C	-5.46952	-4.03052	-10.44340	C	6.40739	6.28668	8.12856
C	-5.41758	-2.94795	-11.28764	C	5.39757	7.18340	8.41312

C	4.71643	7.84048	7.39384
C	5.07897	7.57371	6.07861
C	6.09467	6.68460	5.78561
C	3.64493	8.84249	7.70960
C	11.56202	1.09476	6.62032
C	12.47494	0.12664	6.24899
C	12.05327	-1.09952	5.74720
C	10.68736	-1.32677	5.61832
C	9.76928	-0.36570	5.98945
C	13.03818	-2.13864	5.29793
C	6.72927	-3.45099	5.14737
C	7.78556	-3.65204	4.27983
C	8.49703	-4.84606	4.28226
C	8.10519	-5.84758	5.16457
C	7.04206	-5.66003	6.02499
C	0.45043	-3.64742	9.25567
C	-0.67127	-3.41589	10.02543
C	-1.30000	-4.45371	10.70621
C	-0.77207	-5.73447	10.59181
C	0.34238	-5.97760	9.81247
C	-2.49501	-4.18210	11.57236
C	9.63098	-5.06924	3.32477
C	-2.67511	-1.48776	-16.09350
C	-2.32251	-2.05481	-17.30272
C	-3.20885	-2.86906	-17.99822
C	-4.45882	-3.11405	-17.43941
C	-4.81505	-2.55979	-16.22670
C	-6.71149	-1.47253	-8.85254
C	-6.93642	-0.98103	-7.58209
C	-6.10486	-0.01192	-7.03146
C	-5.04115	0.45697	-7.79435
C	-4.81189	-0.02554	-9.06777
C	-6.37815	0.54339	-5.66442
C	-2.81201	-3.50943	-19.29600
C	0.40929	-8.10788	-2.78541
C	1.75042	-7.86100	-3.00333
C	2.72587	-8.72778	-2.52267
C	2.31948	-9.84845	-1.80701
C	0.98110	-10.09686	-1.57495
C	-6.45785	-11.27264	-1.89231
C	-7.77422	-11.58180	-1.61636
C	-8.80689	-10.74900	-2.03452
C	-8.48132	-9.59728	-2.74131
C	-7.16725	-9.28487	-3.02966
C	-10.23840	-11.10789	-1.76333
C	4.17815	-8.48906	-2.81590
C	9.14696	-5.80057	2.06631
C	10.27871	-6.02036	1.06531
C	9.80477	-6.76585	-0.18032
C	10.93091	-6.97636	-1.19069
C	10.44958	-7.72440	-2.42849
C	4.57150	-9.15133	-4.14311
C	6.04858	-8.94844	-4.47192
C	6.43329	-9.60632	-5.79572
C	7.91184	-9.41653	-6.12900
C	8.28536	-10.06932	-7.45507
C	-2.06356	-3.69361	12.96138
C	-3.26388	-3.38343	13.85260
C	-2.84182	-2.87408	15.22932
C	-4.04258	-2.55501	16.11814
C	-3.61739	-2.03681	17.48742
C	-7.94196	9.94072	-13.72926
C	-9.23865	10.70770	-13.48131
C	-10.36309	10.24690	-14.40666
C	-11.66234	11.01200	-14.16159
C	-12.78115	10.54545	-15.08622
C	6.05228	2.68212	-14.92188
C	7.29674	1.89212	-15.32054
C	7.06340	0.38369	-15.26713
C	8.30898	-0.40561	-15.66583
C	8.07606	-1.91066	-15.59852
C	-2.18553	-4.88872	-19.05164
C	-1.80230	-5.58051	-20.35766
C	-1.18004	-6.95466	-20.11777
C	-0.81031	-7.65626	-21.42335
C	-0.19157	-9.02786	-21.17797
C	-7.39066	1.69402	-5.73661
C	-7.68320	2.28129	-4.35795
C	-8.70440	3.41531	-4.42101
C	-8.99269	4.00802	-3.04293
C	-10.01871	5.13368	-3.10947
C	13.31747	-2.00042	3.79528
C	14.21801	-3.11735	3.27289
C	14.50606	-2.95866	1.78060
C	15.25135	-4.14837	1.17319
C	16.67260	-4.30554	1.70306
C	-10.78844	-11.99695	-2.88697
C	-12.27844	-12.30614	-2.73841
C	-12.60572	-13.17248	-1.52330
C	-14.07972	-13.57294	-1.48965
C	-14.41457	-14.41369	-0.26259
C	5.46138	15.51270	0.08048
C	5.49670	16.89601	-0.58239
C	4.78607	17.95000	0.26330
C	4.81832	19.32961	-0.39118
C	9.14605	4.71793	-9.87729
C	9.46887	3.50717	-10.75333
C	10.25491	2.42134	-10.01845
C	10.37283	1.12248	-10.82044
C	9.08395	0.30746	-10.83162
C	4.24740	10.24207	7.89088
C	3.18066	11.28366	8.22033
C	3.77705	12.67850	8.39913
C	2.71256	13.72241	8.73215
C	3.31251	15.11327	8.90617
C	4.10732	20.38480	0.45415
C	4.14307	21.76043	-0.20197
H	7.07436	4.47384	-14.29430
H	6.63199	4.44629	-16.00695
H	-6.63868	11.46495	-12.94182
H	-7.13404	10.24753	-11.75658
H	11.00255	4.81718	-8.78146
H	10.97743	5.79149	-10.26049
H	3.12766	8.55609	8.62803
H	2.91253	8.87175	6.89985
H	12.63526	-3.13480	5.49413
H	13.97471	-2.03336	5.84926
H	-3.09073	-5.09069	11.68189
H	-3.12154	-3.41722	11.10739
H	10.40997	-5.66640	3.80458
H	10.06362	-4.11051	3.03182
H	-5.45086	0.91151	-5.22028
H	-6.77797	-0.24134	-5.01826
H	-2.09107	-2.87707	-19.81860
H	-3.69019	-3.62623	-19.93493
H	-10.84340	-10.20079	-1.69926
H	-10.30806	-11.63786	-0.81244
H	4.79341	-8.90288	-2.01407
H	4.37506	-7.41661	-2.87906
H	8.35189	-5.21604	1.59651

H	8.72014	-6.76457	2.35320	H	-8.31859	1.32701	-6.18174
H	10.69286	-5.05323	0.76983	H	-6.99818	2.47585	-6.39140
H	11.07973	-6.59016	1.54330	H	-8.06094	1.49263	-3.70228
H	8.99751	-6.20100	-0.65361	H	-6.75444	2.65639	-3.92055
H	9.39818	-7.73705	0.11309	H	-9.63527	3.03894	-4.85275
H	11.33525	-6.00554	-1.48779	H	-8.33002	4.20238	-5.08042
H	11.73941	-7.53978	-0.71822	H	-9.36206	3.22000	-2.38195
H	9.65545	-7.16971	-2.92408	H	-8.06319	4.39001	-2.61352
H	11.26486	-7.86276	-3.13536	H	-10.95975	4.76850	-3.51613
H	10.06280	-8.70394	-2.15454	H	-9.66063	5.93945	-3.74724
H	3.95806	-8.73435	-4.94558	H	-10.20742	5.53886	-2.11759
H	4.35423	-10.22082	-4.08594	H	13.78658	-1.03183	3.60559
H	6.26424	-7.87825	-4.52443	H	12.36807	-2.01502	3.25446
H	6.65889	-9.36906	-3.66893	H	15.15446	-3.11133	3.83328
H	5.82567	-9.18179	-6.59899	H	13.73240	-4.08120	3.44435
H	6.21048	-10.67520	-5.74500	H	15.08813	-2.04786	1.61909
H	8.13723	-8.34828	-6.17555	H	13.55651	-2.83810	1.25221
H	8.51959	-9.84821	-5.33036	H	15.29291	-4.01346	0.08911
H	7.70681	-9.63793	-8.26966	H	14.68773	-5.06391	1.36908
H	9.34197	-9.92321	-7.66921	H	17.23846	-3.38817	1.55227
H	8.08589	-11.13871	-7.42384	H	17.18281	-5.11203	1.18021
H	-1.44353	-4.45913	13.43436	H	16.67042	-4.53907	2.76470
H	-1.45023	-2.79617	12.85038	H	-10.62791	-11.48859	-3.84145
H	-3.88853	-2.62943	13.36660	H	-10.22261	-12.93127	-2.91376
H	-3.86928	-4.28561	13.97153	H	-12.83757	-11.36920	-2.67505
H	-2.23348	-1.97389	15.10985	H	-12.61286	-12.82747	-3.63940
H	-2.22069	-3.62828	15.71918	H	-12.36862	-12.62949	-0.60657
H	-4.64776	-3.45628	16.24378	H	-11.98898	-14.07456	-1.54695
H	-4.66629	-1.80493	15.62534	H	-14.69775	-12.67150	-1.48988
H	-3.01063	-2.77750	18.00466	H	-14.32124	-14.13873	-2.39295
H	-4.48851	-1.81718	18.10108	H	-14.20777	-13.85720	0.64953
H	-3.03068	-1.12588	17.38545	H	-13.81961	-15.32496	-0.24937
H	-7.62471	10.08413	-14.76508	H	-15.46662	-14.69103	-0.26379
H	-8.11652	8.87191	-13.58393	H	5.94614	15.57348	1.05733
H	-9.06195	11.77566	-13.63250	H	4.42072	15.21818	0.23333
H	-9.54768	10.56875	-12.44212	H	5.02182	16.83467	-1.56461
H	-10.05474	10.38490	-15.44611	H	6.53692	17.19228	-0.73786
H	-10.54028	9.17906	-14.25515	H	3.74675	17.64881	0.41759
H	-11.48663	12.07960	-14.31565	H	5.26207	18.00617	1.24560
H	-11.97089	10.87491	-13.12214	H	4.34237	19.27457	-1.37354
H	-12.50170	10.69192	-16.12776	H	5.85725	19.63196	-0.54530
H	-13.69497	11.10381	-14.89420	H	8.51710	5.40899	-10.44518
H	-12.98641	9.48766	-14.93350	H	8.56980	4.39077	-9.00838
H	5.75713	2.41631	-13.90375	H	10.03649	3.83182	-11.62929
H	5.22544	2.41393	-15.58375	H	8.52435	3.08883	-11.10688
H	8.12205	2.15445	-14.65436	H	11.26064	2.78979	-9.80671
H	7.59159	2.17327	-16.33496	H	9.77031	2.20687	-9.06261
H	6.76397	0.09819	-14.25571	H	10.66663	1.35806	-11.84628
H	6.24151	0.12168	-15.93838	H	11.16655	0.51070	-10.38403
H	9.13395	-0.13679	-15.00192	H	8.26932	0.86621	-11.28492
H	8.60065	-0.12948	-16.68231	H	8.79313	0.04354	-9.81640
H	7.79855	-2.20940	-14.58944	H	9.22238	-0.61195	-11.39660
H	8.97712	-2.45097	-15.88117	H	4.76733	10.52903	6.97364
H	7.27414	-2.20362	-16.27342	H	4.98842	10.21136	8.69343
H	-2.89636	-5.51231	-18.50405	H	2.43972	11.30857	7.41705
H	-1.29835	-4.77329	-18.42425	H	2.66262	10.99278	9.13776
H	-2.69288	-5.69252	-20.98140	H	4.29308	12.97048	7.48098
H	-1.09362	-4.95390	-20.90513	H	4.51980	12.65362	9.20067
H	-1.88511	-7.57798	-19.56191	H	1.96856	13.74654	7.93182
H	-0.28365	-6.84306	-19.50241	H	2.19895	13.43197	9.65204
H	-1.70672	-7.76827	-22.03840	H	3.81312	15.42983	7.99317
H	-0.10435	-7.03455	-21.97961	H	4.04137	15.11690	9.71443
H	-0.88747	-9.67032	-20.64193	H	2.53596	15.83816	9.14130
H	0.71580	-8.93678	-20.58404	H	4.58310	20.43986	1.43647
H	0.06214	-9.50794	-22.12073	H	3.06802	20.08354	0.60723

H	3.65285	21.73323	-1.17320	H	-1.28383	10.58288	-12.96629
H	5.17084	22.08734	-0.34739	H	0.73292	4.01126	-15.00829
H	3.63401	22.49436	0.41922	H	-1.00833	2.28021	-14.84016
C	2.55799	-11.34602	3.78371	H	-3.97245	5.24128	-13.97916
H	3.33756	-12.00502	4.16060	H	8.19474	6.62105	2.22947
H	1.99004	-11.88424	3.02886	H	12.13700	8.96652	0.53071
H	3.03247	-10.49331	3.30263	H	3.41276	11.50048	-7.79302
H	1.17535	-11.78031	5.36297	H	4.45865	11.30543	-12.56598
H	2.25900	-10.43255	5.71321	H	4.16132	-0.34197	10.36020
C	-3.88613	-7.58549	4.52661	H	6.72931	-1.84612	7.27927
H	-4.73044	-6.93948	4.29555	H	2.79383	-2.36795	10.24998
H	-3.50563	-7.32217	5.51137	H	4.73835	-6.99622	5.11412
H	-4.23620	-8.61469	4.56115	H	2.98669	-8.51287	4.29151
H	-3.19695	-7.66466	2.48622	H	0.27428	-7.14831	7.30331
H	-2.45840	-6.38531	3.45807	H	-4.07005	-10.26561	2.66849
C	-7.94945	-7.48889	-6.50160	H	-0.29922	-9.50491	0.80643
H	-8.50109	-7.21924	-7.39992	H	-5.16716	-10.42425	0.47191
H	-8.55976	-7.24538	-5.63455	H	-0.98585	-8.66264	-5.08009
H	-7.78466	-8.56428	-6.51963	H	-2.16044	-7.96819	-7.11881
H	-6.83366	-5.65796	-6.42828	H	-5.90310	-9.25029	-5.48410
H	-6.08224	-6.97831	-5.53349	H	-5.29235	-6.48131	-12.76722
C	-2.00967	-8.22498	-10.96222	H	-5.52564	-3.87850	-9.37573
H	-1.41945	-8.19210	-11.87613	H	-5.00209	-4.56238	-14.25575
H	-2.08601	-9.26476	-10.65320	H	-5.23348	1.86724	-11.06059
H	-1.47955	-7.66649	-10.19355	H	-4.10323	3.72391	-12.21001
H	-3.23502	-6.64013	-11.70878	H	-3.10486	1.18484	-15.50508
H	-3.91867	-8.24934	-11.93431	H	-0.78426	-6.91380	5.13887
C	1.29187	9.13748	-8.95228	H	-0.25151	-11.32409	3.09854
H	2.10655	8.83056	-8.29904	H	-3.25916	-8.90533	-8.86965
H	1.40279	8.62628	-9.90625	H	-6.83099	-5.60213	-8.80006
H	0.34957	8.82912	-8.50421	H	3.47770	6.60053	-12.17329
H	0.48711	10.92932	-9.81219	H	5.54308	5.37778	-12.66032
H	1.16246	11.14747	-8.19545	H	4.20986	4.69388	-16.65511
C	7.30230	10.78804	-11.74451	H	2.14088	5.93004	-16.17427
H	7.38443	9.99260	-11.00702	H	-2.73753	9.74392	-14.93305
H	8.30189	11.04575	-12.08865	H	-4.76423	11.04823	-14.44559
H	6.73479	10.40679	-12.59033	H	-6.05300	8.02550	-11.72508
H	7.23136	12.41438	-10.33031	H	-4.02330	6.73247	-12.19317
H	6.53511	12.78719	-11.91150	H	8.80263	7.45809	-5.50629
C	7.65163	6.61763	-1.07256	H	9.58683	5.53404	-6.81723
H	6.70318	6.57187	-1.60386	H	10.27889	8.09304	-10.16077
H	7.80044	5.67948	-0.54139	H	9.53752	10.02398	-8.83923
H	8.45141	6.73180	-1.80100	H	5.55821	12.32951	-3.31107
H	7.49886	8.71808	-0.64784	H	4.40542	13.96104	-1.87849
H	6.83282	7.67399	0.61375	H	8.07736	14.79359	0.12835
C	13.79873	7.04732	2.11449	H	9.23439	13.17547	-1.31050
H	14.71902	7.08101	2.69397	H	6.93496	5.78817	8.92933
H	13.98139	7.51478	1.14968	H	5.12842	7.37562	9.44255
H	13.53960	6.00520	1.94037	H	4.55322	8.06565	5.27195
H	12.54060	7.31135	3.84538	H	6.33716	6.46587	4.75623
H	12.97363	8.81047	3.01684	H	11.90267	2.03651	7.02678
H	4.47940	1.55021	9.17908	H	13.53290	0.32337	6.35480
H	8.09674	-0.47237	8.09226	H	10.33840	-2.27143	5.22550
H	5.44161	3.70175	8.50860	H	8.71425	-0.54821	5.84894
H	11.51593	3.17903	4.75591	H	6.17461	-2.52402	5.11509
H	11.85128	4.95045	3.08250	H	8.06502	-2.86647	3.59104
H	8.54560	7.16069	4.64683	H	8.64460	-6.78452	5.17902
H	11.26855	7.56545	-2.03138	H	6.75545	-6.44053	6.71544
H	8.84938	10.78304	-0.59672	H	0.91646	-2.83713	8.71407
H	10.82527	8.21593	-4.37147	H	-1.07244	-2.41434	10.09629
H	6.22480	13.67090	-5.26210	H	-1.24422	-6.55371	11.11625
H	4.95565	13.66168	-7.37752	H	0.74644	-6.97748	9.74444
H	7.07454	10.20000	-8.71520	H	-1.96316	-0.87862	-15.55615
H	0.45613	11.89589	-11.81533	H	-1.34255	-1.86086	-17.71648
H	3.35889	8.85860	-12.57819	H	-5.16210	-3.74423	-17.96602

H	-5.79567	-2.74276	-15.81109
H	-7.37874	-2.21139	-9.27235
H	-7.76910	-1.35687	-7.00376
H	-4.37968	1.20629	-7.38180
H	-3.96630	0.32914	-9.63889
H	-0.33650	-7.41360	-3.14457
H	2.04746	-6.97834	-3.55283
H	3.06340	-10.53297	-1.42349
H	0.68122	-10.97916	-1.02766
H	-5.66449	-11.93217	-1.57066
H	-8.00718	-12.48154	-1.06373
H	-9.27077	-8.93342	-3.06511
H	-6.93455	-8.36956	-3.55230

*Table 6 Cartesian coordinates of **1c** (GFN2-xTB(GBSA(toluene)). B97-3c(COSMO(toluene) single point energy = 10838.56081 Hartree.*

	X	Y	Z
C	10.67817	-0.98402	6.02406
C	9.66770	-0.94799	6.98607
C	9.25446	0.29540	7.48902
C	9.77924	1.45258	6.95818
C	10.74221	1.39560	5.91699
C	11.22343	0.17498	5.48884
C	10.88593	2.74362	5.42099
C	9.98686	3.59135	6.19610
C	11.39730	3.43368	4.36122
C	10.00363	4.89943	5.57397
C	9.34280	2.84607	7.14141
C	9.36158	6.09098	5.84373
C	9.44479	7.12726	4.91883
C	10.18494	6.99734	3.74864
C	10.92667	5.83027	3.52859
C	10.82714	4.78569	4.42070
C	7.99942	11.40480	-0.45998
C	8.98213	10.44480	-0.68094
C	9.44724	10.22676	-1.98224
C	8.83619	10.87270	-3.03411
C	7.74011	11.74652	-2.80831
C	7.37384	12.06190	-1.51479
C	7.17376	12.03000	-4.11088
C	8.00225	11.34182	-5.09732
C	6.08608	12.58454	-4.71989
C	7.36779	11.51374	-6.38506
C	9.00593	10.65909	-4.47676
C	7.64695	11.04390	-7.65214
C	6.67894	11.16937	-8.64153
C	5.44632	11.76407	-8.38613
C	5.21396	12.34805	-7.13366
C	6.16778	12.23355	-6.14581
C	-0.16519	10.49249	-11.80222
C	1.15655	10.06758	-11.76209
C	1.52714	8.88477	-12.41209
C	0.55116	8.08918	-12.97164
C	-0.81030	8.49682	-12.94086
C	-1.15510	9.71968	-12.40282
C	-1.58372	7.37306	-13.42471
C	-0.63678	6.32241	-13.77313
C	-2.85758	6.88321	-13.43892
C	-1.40101	5.11986	-14.00573
C	0.64193	6.73519	-13.53415
C	-1.04386	3.81317	-14.26611
C	-2.01756	2.82658	-14.18820
C	-3.34792	3.12421	-13.89185
C	-3.72701	4.46929	-13.75137
C	-2.76196	5.45161	-13.76634
C	10.03351	7.95850	2.63726
C	9.50889	7.42258	1.46489
C	9.20021	8.18976	0.35516
C	9.41817	9.57150	0.42674
C	9.96428	10.10598	1.58870
C	10.28957	9.32911	2.69357
C	4.31979	11.57033	-9.31931
C	3.18352	10.98268	-8.76418
C	2.11269	10.55330	-9.52555
C	2.17968	10.71894	-10.91398
C	3.27226	11.37451	-11.46263
C	4.34611	11.81213	-10.69461
C	0.97638	9.83745	-8.84641
C	5.48011	12.54286	-11.36424
C	10.96535	9.96856	3.87531
C	8.59717	7.49286	-0.83658
C	9.50047	-3.43949	7.22776
C	8.92156	-2.17230	7.30382
C	7.53866	-2.07577	7.52050
C	6.77432	-3.21805	7.57490
C	7.36117	-4.49203	7.35969
C	8.73314	-4.59814	7.24052
C	6.26179	-5.41417	7.18213
C	5.02551	-4.65618	7.35067
C	5.98915	-6.65733	6.68876
C	3.93658	-5.54267	7.00181
C	5.31053	-3.34804	7.61430
C	2.56466	-5.39573	6.98102
C	1.79315	-6.38456	6.38209
C	2.37288	-7.52305	5.83019
C	3.75150	-7.72817	5.95835
C	4.53055	-6.74498	6.53055
C	-0.52409	-11.00397	0.91540
C	-0.85624	-9.99669	1.81354
C	-2.03388	-9.26288	1.62704
C	-2.77932	-9.46148	0.48551
C	-2.37670	-10.42963	-0.47431
C	-1.28085	-11.23145	-0.22991
C	-3.24546	-10.25559	-1.61877
C	-4.19909	-9.20964	-1.27502
C	-3.32934	-10.55115	-2.94890
C	-4.91239	-8.86815	-2.48501
C	-3.95149	-8.73381	-0.02057
C	-5.85973	-7.90879	-2.77611
C	-6.19467	-7.68258	-4.10562
C	-5.61589	-8.41753	-5.13643
C	-4.71656	-9.44697	-4.83448
C	-4.35103	-9.66085	-3.52209
C	-5.29895	-5.92080	-11.34913
C	-5.88134	-5.64711	-10.11775
C	-6.42738	-4.38062	-9.87661
C	-6.25930	-3.38346	-10.81183
C	-5.57909	-3.65001	-12.03201
C	-5.15031	-4.93044	-12.31621
C	-5.40206	-2.37146	-12.68913
C	-4.66289	-1.76147	-13.66222
C	-5.65700	-0.07596	-12.35235
C	-5.88273	1.20879	-11.90493
C	-5.17146	2.24946	-12.48360
C	-4.26529	2.03728	-13.52345
C	-4.12226	0.74204	-14.04677
C	-4.78134	-0.30935	-13.44668

C	1.58042	-8.37611	4.92152	C	3.30725	-1.98977	7.04740
C	2.01566	-8.39768	3.59877	C	2.50336	-0.88693	7.26256
C	1.28359	-8.97521	2.57671	C	2.79211	0.02435	8.27185
C	0.05333	-9.56313	2.89550	C	3.90908	-0.20368	9.06868
C	-0.35067	-9.60187	4.22416	C	4.71827	-1.30153	8.85945
C	0.39123	-9.02779	5.24944	C	7.95288	-8.04469	7.24384
C	-5.77114	-7.94582	-6.52725	C	8.87247	-9.02432	6.92634
C	-4.59955	-7.53540	-7.15816	C	8.82402	-9.68024	5.70116
C	-4.59504	-6.86289	-8.36758	C	7.82063	-9.33129	4.80414
C	-5.82627	-6.58038	-8.97169	C	6.89010	-8.36063	5.11841
C	-6.99041	-7.05922	-8.38430	C	9.80671	-10.76363	5.36605
C	-6.98999	-7.75004	-7.17871	C	1.90504	1.20643	8.53160
C	-3.28432	-6.35732	-8.90791	C	-2.54171	-2.01099	-14.93475
C	-8.27821	-8.31507	-6.64676	C	-1.79320	-2.63984	-15.91022
C	-0.07178	-9.17593	6.67264	C	-2.33164	-3.66798	-16.67564
C	1.77855	-8.82414	1.16313	C	-3.64473	-4.05568	-16.43167
C	1.87156	5.99387	-13.76327	C	-4.39662	-3.43874	-15.45196
C	-4.08551	7.60119	-13.13869	C	-6.97657	-1.49930	-8.31573
C	-3.85881	-2.39938	-14.69180	C	-7.65393	-0.82169	-7.32055
C	-7.29143	-1.28588	-9.65713	C	-8.66792	0.07893	-7.62664
C	-2.54122	-11.52246	-3.69053	C	-8.98834	0.28292	-8.96438
C	-4.68152	-7.69989	0.69610	C	-8.30843	-0.38070	-9.96555
C	6.94252	-7.69992	6.34538	C	-9.42151	0.78716	-6.53934
C	4.42908	-2.21528	7.84385	C	-1.52765	-4.31657	-17.76396
C	12.30820	2.94423	3.33949	C	-6.07465	-7.76189	0.76507
C	8.33590	3.24746	8.11085	C	-6.78874	-6.80137	1.45130
C	4.97646	13.32472	-4.14013	C	-6.14293	-5.73799	2.07518
C	10.02420	9.78902	-5.04432	C	-4.75754	-5.66994	1.99363
C	-6.57604	-1.95214	-10.73316	C	-4.03306	-6.63688	1.32207
C	-6.04085	-1.37098	-11.84583	C	-2.38578	-12.81761	-3.19487
C	2.86606	5.89499	-12.79136	C	-1.63354	-13.74898	-3.88247
C	4.02997	5.19554	-13.04708	C	-1.00155	-13.41740	-5.07598
C	4.24478	4.58528	-14.27700	C	-1.15172	-12.12483	-5.56564
C	3.24623	4.67391	-15.24166	C	-1.91093	-11.19032	-4.88932
C	2.07535	5.35945	-14.99007	C	-0.20382	-14.43669	-5.83521
C	5.50292	3.81398	-14.55039	C	-6.93691	-4.68459	2.79309
C	-4.33085	8.83871	-13.73521	C	0.90334	0.89812	9.65220
C	-5.49602	9.52910	-13.46885	C	0.02460	2.10232	9.98249
C	-6.44750	9.01566	-12.59381	C	-0.97842	1.79148	11.09179
C	-6.19230	7.79298	-11.98376	C	-1.84353	2.99998	11.44501
C	-5.03360	7.09086	-12.25277	C	-2.84686	2.67975	12.54730
C	-7.69639	9.78674	-12.28178	C	-7.71219	-3.78940	1.81683
C	4.32695	12.86267	-2.99573	C	-6.78791	-2.97633	0.91376
C	3.25436	13.55506	-2.47044	C	-7.57042	-2.12247	-0.08135
C	2.80071	14.72949	-3.06190	C	-6.65251	-1.30613	-0.98902
C	3.45523	15.19359	-4.19706	C	-7.44113	-0.47633	-1.99550
C	4.52076	14.50010	-4.73695	C	9.25068	-12.14247	5.74551
C	9.68283	8.80387	-5.97031	C	10.22690	-13.26390	5.39745
C	10.64743	7.96094	-6.48510	C	9.66703	-14.63758	5.76301
C	11.97836	8.07540	-6.09824	C	10.65435	-15.78087	5.52051
C	12.31720	9.05963	-5.17669	C	10.94407	-16.02644	4.04342
C	11.35672	9.90007	-4.64833	C	-7.46161	10.76102	-11.11986
C	1.65384	15.49200	-2.46577	C	-8.71699	11.56579	-10.79193
C	13.45633	2.24059	3.70597	C	-8.48891	12.54693	-9.64377
C	14.33146	1.77414	2.74576	C	-9.74364	13.35660	-9.32165
C	14.08695	1.98372	1.39259	C	-9.51114	14.34002	-8.18012
C	12.93481	2.67070	1.02808	C	5.28944	2.31255	-14.31776
C	12.05962	3.14987	1.98300	C	6.55634	1.50903	-14.60180
C	15.01690	1.43438	0.35078	C	6.34556	0.00986	-14.39924
C	7.26472	4.05884	7.73801	C	7.61141	-0.79361	-14.69190
C	6.30288	4.42005	8.66072	C	7.39590	-2.29003	-14.49535
C	6.37892	3.98921	9.98052	C	-1.71171	-3.57238	-19.09309
C	7.44580	3.17736	10.35028	C	-0.88857	-4.20009	-20.21540
C	8.40728	2.80452	9.43221	C	-1.07264	-3.46647	-21.54237
C	5.36468	4.42919	10.99543	C	-0.24103	-4.08678	-22.66363

C	-0.43052	-3.35377	-23.98694
C	-10.60401	-0.06030	-6.05245
C	-11.38783	0.64083	-4.94564
C	-12.54480	-0.21340	-4.43144
C	-13.32557	0.48421	-3.31920
C	-14.47464	-0.37509	-2.80386
C	5.85805	5.68403	11.72886
C	4.92538	6.13273	12.85450
C	3.57855	6.65749	12.35935
C	2.72600	7.20822	13.50132
C	1.38208	7.73242	13.00729
C	-1.07754	-15.16111	-6.86783
C	-0.27697	-16.18884	-7.66435
C	-1.13512	-16.91439	-8.69885
C	-0.32884	-17.93580	-9.49922
C	-1.18603	-18.65699	-10.53337
C	13.02955	7.18807	-6.69778
C	13.57812	7.80146	-7.99296
C	14.64128	6.91139	-8.63301
C	15.29028	7.53522	-9.86990
C	2.13650	16.42399	-1.34677
C	0.98212	17.20171	-0.71917
C	1.45172	18.11814	0.40884
C	0.29631	18.89182	1.04131
C	0.76836	19.79616	2.17429
C	14.64110	-0.01430	0.00982
C	15.44812	-0.59065	-1.15390
C	16.93415	-0.76086	-0.84275
C	17.67642	-1.46023	-1.98032
C	19.16334	-1.61502	-1.68092
C	14.34409	7.65332	-11.06388
C	15.06155	8.18464	-12.30007
H	6.30398	4.16651	-13.89738
H	5.81359	3.97050	-15.58615
H	-8.50053	9.09845	-12.01283
H	-8.01135	10.35281	-13.16128
H	0.92080	14.79429	-2.05418
H	1.16211	16.08698	-3.23830
H	14.95649	2.03989	-0.55605
H	16.04229	1.46760	0.72133
H	4.41838	4.64647	10.49873
H	5.19958	3.63413	11.72633
H	10.74242	-10.59617	5.90382
H	10.02114	-10.74974	4.29494
H	2.51455	2.06393	8.82552
H	1.35850	1.47087	7.62404
H	-9.79743	1.74284	-6.91099
H	-8.75641	0.98654	-5.69628
H	-1.84215	-5.35480	-17.89039
H	-0.46858	-4.31053	-17.49683
H	0.21329	-15.17191	-5.14365
H	0.62413	-13.94679	-6.35271
H	-6.26938	-4.06516	3.39549
H	-7.65126	-5.16776	3.46509
H	1.45133	0.59170	10.54638
H	0.27422	0.05800	9.34825
H	0.65860	2.93663	10.29334
H	-0.51524	2.41496	9.08504
H	-1.62295	0.96772	10.77483
H	-0.43898	1.46252	11.98366
H	-2.37968	3.33339	10.55290
H	-1.19989	3.82126	11.76962
H	-3.51636	1.88106	12.23390
H	-3.44590	3.55591	12.78664
H	-2.33192	2.35854	13.45065

H	-8.34797	-3.10927	2.38896
H	-8.36241	-4.41111	1.19743
H	-6.12933	-3.65528	0.36635
H	-6.15781	-2.32852	1.52860
H	-8.23315	-1.44538	0.46391
H	-8.19716	-2.77279	-0.69715
H	-6.03458	-0.64412	-0.37725
H	-5.98125	-1.98305	-1.52372
H	-8.10178	0.21947	-1.48192
H	-8.04808	-1.12356	-2.62537
H	-6.76822	0.09443	-2.63224
H	8.30633	-12.30465	5.22017
H	9.03779	-12.16009	6.81730
H	10.44229	-13.22628	4.32814
H	11.16631	-13.10439	5.93303
H	8.75580	-14.82138	5.18821
H	9.39210	-14.63274	6.82129
H	10.23871	-16.69474	5.95301
H	11.58994	-15.56573	6.04268
H	10.01863	-16.20386	3.49888
H	11.58345	-16.89896	3.92507
H	11.44976	-15.17438	3.59632
H	-7.14625	10.19809	-10.23796
H	-6.64916	11.44269	-11.38283
H	-9.52602	10.88091	-10.52539
H	-9.03307	12.11897	-11.68009
H	-8.17718	11.99518	-8.75325
H	-7.67780	13.23009	-9.90833
H	-10.55370	12.67414	-9.05254
H	-10.05695	13.90512	-10.21351
H	-9.21594	13.81227	-7.27529
H	-10.41797	14.90285	-7.96902
H	-8.72207	15.04393	-8.43756
H	4.97670	2.15051	-13.28324
H	4.48198	1.96103	-14.96439
H	7.35776	1.85280	-13.94300
H	6.87550	1.69095	-15.63132
H	6.03078	-0.17527	-13.36904
H	5.54187	-0.33344	-15.05560
H	8.41442	-0.45372	-14.03324
H	7.92712	-0.60574	-15.72123
H	7.10113	-2.50179	-13.46934
H	8.30950	-2.84009	-14.71071
H	6.61224	-2.65304	-15.15747
H	-1.41548	-2.52871	-18.96277
H	-2.76982	-3.58236	-19.36551
H	0.16894	-4.18510	-19.93937
H	-1.18224	-5.24568	-20.33801
H	-0.78589	-2.41909	-21.41874
H	-2.12868	-3.48761	-21.82323
H	0.81530	-4.06250	-22.38442
H	-0.52597	-5.13465	-22.78603
H	-0.13376	-2.31109	-23.89164
H	-1.47392	-3.38503	-24.29467
H	0.17177	-3.81216	-24.76853
H	-10.22963	-1.01868	-5.68498
H	-11.26743	-0.26800	-6.89560
H	-10.71452	0.87119	-4.11634
H	-11.77923	1.58831	-5.32457
H	-12.15325	-1.16193	-4.05525
H	-13.22158	-0.44132	-5.25886
H	-12.64742	0.71495	-2.49386
H	-13.72136	1.43063	-3.69580
H	-14.09861	-1.31439	-2.40304
H	-15.17213	-0.60167	-3.60793

H	-15.01631	0.14242	-2.01493
H	6.84434	5.47367	12.15084
H	5.97687	6.49647	11.00775
H	4.75896	5.29867	13.54103
H	5.42300	6.92679	13.41776
H	3.74876	7.44903	11.62499
H	3.02703	5.85581	11.86459
H	3.26791	8.01576	14.00003
H	2.55865	6.41892	14.23855
H	0.79102	8.11462	13.83689
H	0.81767	6.93915	12.52102
H	1.52686	8.53775	12.28963
H	-1.51010	-14.42568	-7.55044
H	-1.90329	-15.65844	-6.35323
H	0.54991	-15.68609	-8.17261
H	0.15506	-16.92118	-6.97758
H	-1.57075	-16.18284	-9.38415
H	-1.95937	-17.42301	-8.19245
H	0.49598	-17.42715	-10.00455
H	0.10527	-18.66867	-8.81450
H	-1.61226	-17.94633	-11.23879
H	-2.00257	-19.18875	-10.04879
H	-0.58968	-19.37713	-11.08970
H	13.85032	7.05220	-5.99011
H	12.60476	6.20677	-6.92105
H	14.00863	8.78080	-7.76989
H	12.74987	7.95290	-8.68714
H	14.19420	5.95207	-8.90572
H	15.42168	6.71226	-7.89348
H	16.14380	6.91782	-10.16288
H	15.67385	8.52625	-9.61437
H	2.63768	15.83100	-0.57797
H	2.87097	17.12318	-1.75403
H	0.24394	16.49755	-0.32662
H	0.48893	17.80069	-1.48898
H	1.94703	17.51944	1.17750
H	2.18744	18.82530	0.01741
H	-0.44276	18.18485	1.42659
H	-0.19429	19.49709	0.27498
H	1.23620	19.20867	2.96196
H	-0.06951	20.34104	2.60414
H	1.49614	20.51789	1.80857
H	14.78013	-0.63879	0.89549
H	13.57944	-0.04477	-0.24951
H	15.03026	-1.56776	-1.41164
H	15.33367	0.05555	-2.02798
H	17.04632	-1.34670	0.07321
H	17.39106	0.21588	-0.67286
H	17.55025	-0.88371	-2.90024
H	17.23603	-2.44670	-2.14641
H	19.62711	-0.64141	-1.53421
H	19.66969	-2.11546	-2.50361
H	19.31176	-2.20378	-0.77768
H	13.92102	6.67032	-11.28605
H	13.51922	8.32402	-10.81696
H	15.87692	7.52207	-12.58424
H	15.47599	9.17193	-12.10516
H	14.37307	8.26140	-13.13915
C	0.33094	-10.54779	7.22040
H	1.41371	-10.65575	7.20627
H	-0.10095	-11.34280	6.61597
H	-0.01506	-10.66257	8.24551
H	-1.15907	-9.07685	6.71875
H	0.37331	-8.40265	7.29912
C	1.16241	-7.58606	0.50756

H	1.55787	-7.45243	-0.49723
H	1.38509	-6.69507	1.09169
H	0.08215	-7.69498	0.44043
H	1.52833	-9.69909	0.56445
H	2.86577	-8.71842	1.17467
C	-8.59202	-9.64606	-7.33596
H	-8.67689	-9.50847	-8.41203
H	-9.52839	-10.05492	-6.96199
H	-7.79872	-10.36595	-7.14380
H	-9.09481	-7.61325	-6.83307
H	-8.20490	-8.48403	-5.57217
C	-3.01900	-4.92490	-8.43898
H	-2.05134	-4.58161	-8.79880
H	-3.02585	-4.87272	-7.35198
H	-3.78522	-4.25479	-8.82259
H	-3.27972	-6.37682	-9.99719
H	-2.47888	-7.00423	-8.55254
C	1.21274	8.32562	-8.83331
H	2.15714	8.09231	-8.34524
H	1.24292	7.94112	-9.85035
H	0.40996	7.82141	-8.29929
H	0.03402	10.04224	-9.35506
H	0.89397	10.19432	-7.81709
C	6.24443	11.66334	-12.35588
H	6.65097	10.78383	-11.86108
H	7.06785	12.22272	-12.79516
H	5.59160	11.32582	-13.15743
H	6.16832	12.92441	-10.60826
H	5.06937	13.40047	-11.90587
C	9.63338	6.65494	-1.58940
H	9.17322	6.17087	-2.44843
H	10.04992	5.88815	-0.94036
H	10.45166	7.27650	-1.94554
H	8.15021	8.21604	-1.51932
H	7.80192	6.82873	-0.48534
C	12.46046	10.15037	3.60042
H	12.94608	10.62808	4.44880
H	12.61491	10.76742	2.71770
H	12.93216	9.18456	3.43048
H	10.84433	9.34798	4.76318
H	10.51596	10.94524	4.07128
H	10.99684	-1.93660	5.62763
H	8.49383	0.34555	8.25395
H	11.96272	0.11576	4.70344
H	8.76739	6.21038	6.73775
H	8.87604	8.03129	5.08606
H	11.54081	5.75238	2.64318
H	7.67020	11.59282	0.55241
H	10.24622	9.52218	-2.15653
H	6.58214	12.76987	-1.31831
H	8.57750	10.53850	-7.86465
H	6.85878	10.72544	-9.60859
H	4.26970	12.83593	-6.93705
H	-0.43244	11.42912	-11.33236
H	2.56465	8.58319	-12.40803
H	-2.18327	10.05091	-12.39525
H	-0.01499	3.54910	-14.46267
H	-1.71791	1.79529	-14.29925
H	-4.76498	4.72266	-13.59620
H	9.29688	6.36210	1.43754
H	10.14159	11.17260	1.63047
H	3.16268	10.80917	-7.69655
H	3.29110	11.53630	-12.53192
H	10.57200	-3.52728	7.12123
H	7.06166	-1.10789	7.55798

H	9.20531	-5.56082	7.11010
H	2.09484	-4.51122	7.38499
H	0.72608	-6.23907	6.28787
H	4.19023	-8.63808	5.57523
H	0.35112	-11.61082	1.10249
H	-2.30813	-8.51126	2.35310
H	-0.98333	-11.99327	-0.93552
H	-6.29372	-7.30384	-1.99346
H	-6.87568	-6.87980	-4.35146
H	-4.29186	-10.03307	-5.63657
H	-4.93508	-6.91879	-11.55158
H	-6.91725	-4.18793	-8.93314
H	-4.66040	-5.15528	-13.25231
H	-6.55244	1.39837	-11.07912
H	-5.28197	3.24075	-12.07110
H	-3.47332	0.57292	-14.89305
H	2.94075	-7.89208	3.35533
H	-1.29142	-10.07985	4.46405
H	-3.65812	-7.70059	-6.65116
H	-7.93477	-6.86316	-8.87499
H	2.71185	6.34274	-11.82052
H	4.78722	5.12592	-12.27846
H	3.39323	4.20342	-16.20393
H	1.31315	5.43358	-15.75273
H	-3.60584	9.24052	-14.42877
H	-5.67473	10.48065	-13.95013
H	-6.91356	7.38417	-11.28962
H	-4.84053	6.15579	-11.74772
H	4.65316	11.93831	-2.54152
H	2.75370	13.17527	-1.59056
H	3.11990	16.10692	-4.66863
H	5.02371	14.87871	-5.61530
H	8.64857	8.68970	-6.26114
H	10.36378	7.19621	-7.19501
H	13.34723	9.16418	-4.86482
H	11.63823	10.66690	-3.94061
H	13.66399	2.08325	4.75480
H	15.22167	1.24053	3.04867
H	12.72140	2.83327	-0.01941
H	11.15586	3.65699	1.67934
H	7.18010	4.38038	6.71018
H	5.47616	5.04446	8.35105
H	7.52213	2.83183	11.37212
H	9.23854	2.18696	9.74119
H	3.08984	-2.67079	6.23771
H	1.64174	-0.72488	6.62956
H	4.14984	0.49380	9.85867
H	5.57215	-1.47215	9.49925
H	7.99639	-7.54649	8.20195
H	9.64513	-9.28195	7.63755
H	7.77277	-9.82361	3.84271
H	6.13881	-8.08067	4.39467
H	-2.09456	-1.23924	-14.32544
H	-0.76984	-2.33311	-16.07680
H	-4.08026	-4.85494	-17.01528
H	-5.41882	-3.74308	-15.27713
H	-6.16980	-2.16891	-8.05568
H	-7.38435	-0.98761	-6.28690
H	-9.77590	0.97701	-9.22356
H	-8.56897	-0.21654	-11.00155
H	-6.58738	-8.58746	0.29204
H	-7.86570	-6.87692	1.50841
H	-4.23604	-4.84742	2.46325
H	-2.95857	-6.54997	1.25226
H	-2.87802	-13.08983	-2.27193
H	-1.52863	-14.74902	-3.48503
H	-0.65965	-11.84489	-6.48684
H	-1.98716	-10.18225	-5.26932

*Table 7 Cartesian coordinates of **1e** (GFN2-xTB(GBSA(toluene)). B97-3c(COSMO(toluene) single point energy = 10838.550704 Hartree.*

	X	Y	Z
C	6.69974	1.77897	8.93046
C	6.92176	0.52428	8.36183
C	7.94481	0.39480	7.40940
C	8.61043	1.51147	6.96330
C	8.28412	2.80131	7.46157
C	7.37197	2.91434	8.49275
C	9.03052	3.74602	6.65744
C	9.89162	2.96478	5.77554
C	9.09810	5.06812	6.32013
C	10.52389	3.88661	4.86121
C	9.64324	1.63356	5.92709
C	11.35069	3.70798	3.77175
C	11.58291	4.78712	2.92887
C	10.99441	6.02833	3.15508
C	10.22242	6.22910	4.30567
C	10.00426	5.17243	5.16531
C	11.03138	8.30675	-2.70785
C	10.55170	9.07306	-1.64903
C	9.84531	10.25063	-1.91403
C	9.51700	10.55447	-3.21949
C	9.87705	9.67631	-4.27545
C	10.70196	8.59863	-4.02555
C	9.15528	10.12654	-5.44471
C	8.43115	11.33527	-5.06571
C	8.80729	9.74365	-6.70679
C	7.62372	11.72175	-6.20412
C	8.64231	11.61244	-3.74618
C	6.71548	12.73676	-6.42999
C	5.91566	12.68055	-7.56642
C	6.02126	11.63659	-8.47980
C	7.01599	10.66824	-8.30769
C	7.81921	10.71806	-7.18935
C	0.53840	10.48916	-12.11791
C	1.82304	9.97152	-11.99367
C	2.12965	8.73785	-12.58173
C	1.12021	7.98647	-13.14301
C	-0.21591	8.47185	-13.14313
C	-0.48907	9.74592	-12.68935
C	-1.04941	7.37109	-13.57466
C	-0.15991	6.26686	-13.90867
C	-2.34280	6.93813	-13.52860
C	-0.98547	5.09606	-14.09644
C	1.13919	6.61837	-13.68070
C	-0.69767	3.77216	-14.35768
C	-1.70946	2.82944	-14.22480
C	-3.00144	3.19282	-13.85049
C	-3.31943	4.55180	-13.70909
C	-2.31967	5.49393	-13.80848
C	10.98547	7.02315	2.06371
C	9.72714	7.35471	1.56659
C	9.54889	8.08178	0.40315
C	10.68801	8.51464	-0.28711
C	11.94098	8.26580	0.25847
C	12.11813	7.52573	1.42124
C	4.96724	11.40703	-9.48939

C	3.69360	11.16932	-8.97986
C	2.63527	10.75399	-9.76842
C	2.85608	10.59861	-11.14348
C	4.10881	10.90354	-11.66225
C	5.17616	11.29622	-10.86365
C	1.35207	10.36075	-9.08627
C	6.51421	11.58133	-11.49151
C	13.50800	7.29623	1.95385
C	8.15227	8.25105	-0.13199
C	4.66901	-0.39830	8.83221
C	6.02518	-0.61703	8.58643
C	6.46565	-1.93062	8.35437
C	5.54346	-2.94508	8.23001
C	4.16050	-2.69345	8.43670
C	3.73711	-1.42683	8.78440
C	3.46348	-3.90471	8.07142
C	4.47211	-4.87346	7.66159
C	2.21515	-4.35274	7.74723
C	3.77207	-5.97566	7.03973
C	5.71985	-4.33028	7.76770
C	4.19699	-7.10802	6.37630
C	3.26641	-7.85948	5.66689
C	1.91753	-7.51890	5.64379
C	1.47232	-6.42863	6.40470
C	2.39028	-5.64345	7.06668
C	-0.15517	-9.31341	0.02515
C	-1.03200	-9.35174	1.10581
C	-2.38684	-9.62197	0.90153
C	-2.85294	-9.75530	-0.39321
C	-1.95379	-9.65472	-1.48777
C	-0.60346	-9.47145	-1.27838
C	-2.75672	-9.68227	-2.68685
C	-4.14194	-9.87110	-2.27719
C	-2.68686	-9.36564	-4.01024
C	-4.95774	-9.70831	-3.46413
C	-4.22042	-9.91739	-0.91402
C	-6.30980	-9.78623	-3.72784
C	-6.78350	-9.41945	-4.98624
C	-5.93091	-8.95268	-5.98290
C	-4.54752	-8.96859	-5.74099
C	-4.06713	-9.34200	-4.50950
C	-6.31343	-5.77561	-11.91107
C	-6.48271	-5.61286	-10.53802
C	-6.50756	-4.30921	-10.01761
C	-6.31008	-3.23292	-10.84877
C	-5.99252	-3.42286	-12.22055
C	-6.04294	-4.69633	-12.75043
C	-5.60280	-2.12104	-12.72582
C	-4.95665	-1.51459	-13.76592
C	-5.29657	0.10706	-12.09522
C	-5.28054	1.36392	-11.52811
C	-4.59385	2.37598	-12.18053
C	-3.93260	2.15329	-13.38829
C	-4.04633	0.90525	-14.01645
C	-4.72285	-0.11323	-13.37628
C	1.00698	-8.11752	4.64840
C	0.35947	-7.20407	3.81680
C	-0.37179	-7.58827	2.70976
C	-0.46538	-8.95296	2.41700
C	0.09965	-9.86975	3.29015
C	0.83281	-9.48205	4.40777
C	-6.35517	-8.31737	-7.24394
C	-5.59184	-8.56647	-8.38729
C	-5.63605	-7.78559	-9.52971
C	-6.53638	-6.70648	-9.55105
C	-7.40337	-6.55730	-8.47066
C	-7.33173	-7.31619	-7.31287
C	-4.68371	-8.04775	-10.66976
C	-8.26650	-6.96091	-6.18864
C	1.39786	-10.53961	5.31889
C	-1.05207	-6.54100	1.87139
C	2.32907	5.82367	-13.93901
C	-3.52584	7.72354	-13.21508
C	-4.55059	-2.11358	-15.02681
C	-6.49078	-1.26647	-9.19022
C	-1.50235	-9.10907	-4.81368
C	-5.42698	-10.04709	-0.11300
C	0.94879	-3.67419	7.96700
C	6.99295	-4.94149	7.42235
C	8.37571	6.19322	6.88977
C	10.23998	0.51381	5.21580
C	9.21852	8.56965	-7.45907
C	8.09163	12.68626	-2.93415
C	-6.20684	-1.81018	-10.50813
C	-5.77970	-1.17075	-11.63359
C	3.35104	5.67627	-13.00299
C	4.48095	4.94048	-13.30799
C	4.63392	4.34314	-14.55315
C	3.60467	4.47611	-15.48015
C	2.46741	5.19615	-15.17901
C	5.85802	3.54352	-14.89282
C	-3.74041	8.94311	-13.85840
C	-4.85669	9.70316	-13.57182
C	-5.78583	9.28110	-12.62713
C	-5.56486	8.07176	-11.97803
C	-4.45579	7.30077	-12.26621
C	-7.01758	10.08988	-12.34396
C	9.25540	7.31530	-6.84882
C	9.62184	6.19613	-7.56724
C	9.96138	6.28777	-8.91368
C	9.92591	7.53754	-9.52001
C	9.55858	8.66368	-8.80816
C	6.73149	12.99144	-2.97946
C	6.21494	14.00996	-2.20402
C	7.03531	14.75916	-1.36705
C	8.39013	14.45161	-1.32083
C	8.91302	13.42516	-2.08313
C	10.30799	5.05092	-9.68974
C	7.01451	6.09198	7.17864
C	6.32814	7.16468	7.71080
C	6.97453	8.36701	7.97791
C	8.33156	8.46576	7.69217
C	9.02357	7.40144	7.14813
C	6.23310	9.51183	8.60340
C	11.62713	0.40366	5.11369
C	12.20023	-0.67159	4.46518
C	11.41238	-1.66133	3.88590
C	10.03071	-1.53860	3.96925
C	9.44909	-0.47322	4.62921
C	12.04366	-2.80947	3.15380
C	7.92383	-4.27484	6.62592
C	9.13007	-4.86714	6.30785
C	9.44960	-6.13632	6.77611
C	8.51608	-6.80549	7.56099
C	7.30501	-6.22329	7.87725
C	0.66651	-3.11550	9.21454
C	-0.53016	-2.46551	9.43996
C	-1.47737	-2.33983	8.42922
C	-1.19003	-2.88548	7.18341
C	0.00155	-3.54425	6.95188

C	-2.79460	-1.67046	8.69027
C	10.75038	-6.78787	6.40750
C	-3.26340	-1.94109	-15.53489
C	-2.89641	-2.52824	-16.72992
C	-3.79930	-3.29247	-17.46028
C	-5.08201	-3.46589	-16.95145
C	-5.45374	-2.89079	-15.75299
C	-7.64350	-1.64698	-8.50211
C	-7.92197	-1.12213	-7.25584
C	-7.05850	-0.21499	-6.65133
C	-5.90394	0.15355	-7.33295
C	-5.62132	-0.36092	-8.58294
C	-7.39618	0.39084	-5.32057
C	-3.38583	-3.95577	-18.74114
C	-6.36210	-11.03749	-0.41720
C	-7.53101	-11.14660	0.30750
C	-7.81426	-10.26454	1.34601
C	-6.87761	-9.28523	1.65390
C	-5.69778	-9.17833	0.94243
C	-0.42876	-9.99953	-4.76816
C	0.68815	-9.79546	-5.55328
C	0.77653	-8.69425	-6.39818
C	-0.28364	-7.79563	-6.42511
C	-1.40869	-7.99668	-5.64910
C	1.96236	-8.51198	-7.29918
C	-9.12342	-10.34856	2.07617
C	10.57979	-7.67010	5.16307
C	11.84610	-8.44575	4.79752
C	12.98577	-7.54710	4.31901
C	14.17216	-8.32846	3.75122
C	14.90246	-9.16202	4.79885
C	-10.28311	-9.84498	1.20449
C	-10.13343	-8.37178	0.83200
C	-11.26882	-7.88944	-0.06833
C	-11.10636	-6.42270	-0.46287
C	-12.23561	-5.94685	-1.36995
C	-3.81923	-2.68154	9.22183
C	-5.17665	-2.03349	9.48420
C	-6.19488	-3.04459	10.00874
C	-7.53899	-2.41713	10.38332
C	-8.31290	-1.88303	9.18259
C	-8.17072	9.66111	-13.26095
C	-9.44354	10.45827	-12.98612
C	-10.59480	10.03301	-13.89535
C	-11.87078	10.82609	-13.61884
C	-13.01721	10.39612	-14.52720
C	5.59903	2.04010	-14.73042
C	6.79485	1.20410	-15.18067
C	6.53441	-0.29406	-15.03767
C	7.72790	-1.13291	-15.49061
C	7.46956	-2.62627	-15.32573
C	-2.80423	-5.34898	-18.46713
C	-2.40532	-6.06332	-19.75613
C	-1.81558	-7.44632	-19.48715
C	-1.43542	-8.17129	-20.77682
C	-0.84341	-9.54924	-20.50296
C	-8.27533	1.63460	-5.50727
C	-8.66225	2.27144	-4.17475
C	-9.55541	3.49566	-4.36653
C	-9.95595	4.13419	-3.03797
C	-10.85709	5.34758	-3.23828
C	12.49300	-2.38589	1.74950
C	13.12500	-3.54457	0.98136
C	13.60979	-3.11058	-0.40096
C	14.13425	-4.26782	-1.25334
C	15.41585	-4.88946	-0.70831
C	1.73932	-9.24636	-8.62813
C	2.92301	-9.08631	-9.57880
C	2.70351	-9.83129	-10.89409
C	3.88211	-9.67111	-11.85246
C	3.66035	-10.42477	-13.15906
C	6.47634	15.90305	-0.57280
C	6.42900	17.17750	-1.42593
C	5.87785	18.36870	-0.64612
C	5.80761	19.63041	-1.50411
C	9.03375	4.27455	-10.04794
C	9.29410	3.02431	-10.88844
C	10.13474	1.97233	-10.16463
C	10.18649	0.63508	-10.90846
C	8.90080	-0.17374	-10.77250
C	6.26407	9.40326	10.13360
C	5.50863	10.54842	10.80357
C	5.53393	10.43702	12.32668
C	4.77649	11.57876	13.00201
C	4.80182	11.45839	14.52156
C	5.26441	20.82831	-0.72742
C	5.18748	22.08061	-1.59357
H	6.68780	3.83811	-14.24735
H	6.14551	3.74071	-15.92855
H	-6.81139	11.15058	-12.50269
H	-7.32065	9.95260	-11.30361
H	10.96073	4.41725	-9.08710
H	10.83671	5.31865	-10.60677
H	5.19375	9.50888	8.26744
H	6.68669	10.45877	8.30316
H	11.32755	-3.62895	3.06486
H	12.91200	-3.17083	3.71034
H	-3.17602	-1.22809	7.76739
H	-2.66739	-0.87223	9.42494
H	11.10077	-7.40865	7.23530
H	11.49880	-6.01952	6.21040
H	-6.48163	0.67371	-4.79498
H	-7.93275	-0.33572	-4.70637
H	-2.63328	-3.34861	-19.24901
H	-4.24877	-4.05437	-19.40328
H	2.86053	-8.90692	-6.81942
H	2.11995	-7.44999	-7.49931
H	-9.07675	-9.75406	2.99070
H	-9.32061	-11.38717	2.35400
H	10.27583	-7.04514	4.31972
H	9.77201	-8.38232	5.35081
H	11.59990	-9.15197	3.99973
H	12.16930	-9.02405	5.66488
H	12.60231	-6.88545	3.53825
H	13.33742	-6.92337	5.14301
H	13.82392	-8.98143	2.94688
H	14.87828	-7.61707	3.31469
H	14.26296	-9.95147	5.18522
H	15.78583	-9.62527	4.36372
H	15.21916	-8.53548	5.63053
H	-11.22143	-9.98869	1.74558
H	-10.33067	-10.44174	0.29086
H	-9.18071	-8.22600	0.31692
H	-10.11307	-7.76773	1.74267
H	-12.22335	-8.02084	0.44788
H	-11.29573	-8.50269	-0.97280
H	-11.08281	-5.80702	0.43981
H	-10.14985	-6.29298	-0.97560
H	-13.19653	-6.04946	-0.86961
H	-12.26295	-6.53476	-2.28538

H	-12.09745	-4.90134	-1.63740	H	15.78980	-5.64542	-1.39595
H	-3.93486	-3.48909	8.49466	H	15.24135	-5.36502	0.25339
H	-3.43881	-3.12361	10.14598	H	0.83514	-8.85816	-9.10317
H	-5.54218	-1.58950	8.55664	H	1.57457	-10.30745	-8.42593
H	-5.05831	-1.22961	10.21528	H	3.07911	-8.02475	-9.78680
H	-6.35692	-3.81861	9.25439	H	3.82815	-9.46454	-9.09698
H	-5.77901	-3.53180	10.89489	H	1.79581	-9.45637	-11.37368
H	-8.14718	-3.17516	10.88382	H	2.55106	-10.89330	-10.68571
H	-7.37170	-1.60612	11.09641	H	4.03051	-8.60988	-12.06697
H	-8.45606	-2.66814	8.44268	H	4.79085	-10.04081	-11.37106
H	-9.29145	-1.52387	9.49486	H	2.76810	-10.05927	-13.66360
H	-7.78466	-1.05760	8.71230	H	3.53351	-11.48878	-12.96910
H	-7.87037	9.79913	-14.30247	H	4.51003	-10.29388	-13.82578
H	-8.36771	8.59640	-13.11393	H	7.09699	16.08450	0.30714
H	-9.24257	11.52257	-13.13269	H	5.46566	15.66301	-0.23468
H	-9.73849	10.31929	-11.94285	H	5.80493	16.99645	-2.30438
H	-10.30209	10.17426	-14.93888	H	7.43674	17.40772	-1.78061
H	-10.79438	8.96814	-13.75096	H	4.87772	18.12781	-0.27671
H	-11.67231	11.89076	-13.76535	H	6.51348	18.55771	0.22266
H	-12.16345	10.68543	-12.57530	H	5.16691	19.44260	-2.36948
H	-12.75363	10.54718	-15.57220	H	6.80626	19.86783	-1.87956
H	-13.91375	10.97385	-14.31232	H	8.36688	4.94160	-10.60114
H	-13.24482	9.34192	-14.38155	H	8.51987	3.98892	-9.12693
H	5.37276	1.82538	-13.68306	H	9.79462	3.30747	-11.81799
H	4.72133	1.76260	-15.31909	H	8.32711	2.59104	-11.15145
H	7.67277	1.47728	-14.59040	H	11.15446	2.34567	-10.05142
H	7.01899	1.43078	-16.22629	H	9.72705	1.80747	-9.16406
H	6.30724	-0.52487	-13.99406	H	10.39454	0.81903	-11.96531
H	5.65778	-0.56585	-15.63141	H	11.01335	0.04193	-10.50954
H	8.60865	-0.85120	-14.90864	H	8.04974	0.37004	-11.17410
H	7.94324	-0.91632	-16.53998	H	8.70096	-0.39437	-9.72547
H	7.27212	-2.86666	-14.28282	H	8.98742	-1.11606	-11.30929
H	8.33176	-3.20319	-15.65365	H	7.30338	9.40376	10.47111
H	6.60738	-2.93200	-15.91529	H	5.82196	8.44985	10.43290
H	-3.54631	-5.94749	-17.93316	H	5.95410	11.50041	10.50389
H	-1.93131	-5.25138	-17.81719	H	4.47119	10.54740	10.45943
H	-3.28353	-6.16500	-20.39883	H	6.57103	10.43915	12.67162
H	-1.67212	-5.45697	-20.29404	H	5.08989	9.48418	12.62594
H	-2.54334	-8.04844	-18.93722	H	5.22208	12.53190	12.70652
H	-0.92888	-7.34458	-18.85627	H	3.73981	11.57789	12.65610
H	-2.32300	-8.27692	-21.40562	H	5.82575	11.48140	14.88950
H	-0.71009	-7.56850	-21.32895	H	4.34652	10.52186	14.83778
H	-1.55819	-10.17268	-19.96930	H	4.25367	12.27862	14.98025
H	0.05594	-9.46478	-19.89601	H	5.90900	21.02221	0.13350
H	-0.58300	-10.04648	-21.43492	H	4.26814	20.58962	-0.34676
H	-9.17991	1.35325	-6.05202	H	4.53100	21.91544	-2.44570
H	-7.73745	2.36370	-6.11820	H	6.17376	22.34648	-1.96894
H	-9.18737	1.53497	-3.56122	H	4.80065	22.92007	-1.01977
H	-7.75743	2.56419	-3.63602	C	2.46264	-11.39879	4.63402
H	-10.45707	3.20184	-4.91009	H	3.28946	-10.78464	4.28358
H	-9.02936	4.23406	-4.97692	H	2.04532	-11.92114	3.77640
H	-10.47649	3.39413	-2.42510	H	2.85349	-12.13748	5.33078
H	-9.05576	4.43623	-2.49685	H	0.58116	-11.19174	5.64320
H	-11.77063	5.06306	-3.75687	H	1.82323	-10.06986	6.20727
H	-10.34999	6.10615	-3.83132	C	-2.45412	-6.25251	2.41259
H	-11.12853	5.78548	-2.28009	H	-2.94821	-5.49673	1.80545
H	13.21311	-1.56860	1.83388	H	-2.39919	-5.89608	3.43928
H	11.62950	-2.00678	1.19670	H	-3.05505	-7.15855	2.39779
H	13.96341	-3.93746	1.55934	H	-1.12786	-6.87377	0.83476
H	12.39083	-4.34646	0.86843	H	-0.46455	-5.62007	1.89151
H	14.39688	-2.36057	-0.28915	C	-9.63498	-7.62037	-6.37752
H	12.77838	-2.63724	-0.93063	H	-10.09248	-7.28093	-7.30463
H	14.32628	-3.89429	-2.26262	H	-10.29539	-7.36625	-5.55087
H	13.36217	-5.03753	-1.32904	H	-9.54124	-8.70322	-6.42816
H	16.18490	-4.12941	-0.58425	H	-8.40344	-5.87628	-6.17157

H	-7.83927	-7.25684	-5.22990
C	-3.39465	-8.77498	-10.29317
H	-2.72012	-8.77761	-11.14741
H	-3.58019	-9.80938	-10.01386
H	-2.89760	-8.27146	-9.46687
H	-4.41631	-7.08896	-11.12084
H	-5.20266	-8.63513	-11.43457
C	1.34302	8.85827	-8.79434
H	2.20106	8.58352	-8.18371
H	1.38537	8.29412	-9.72367
H	0.43412	8.58102	-8.26446
H	0.48546	10.60285	-9.69873
H	1.26690	10.90807	-8.14480
C	7.15395	10.32222	-12.08009
H	7.28781	9.56459	-11.31112
H	8.12714	10.55862	-12.50559
H	6.52845	9.90138	-12.86399
H	7.18236	12.01446	-10.74458
H	6.38229	12.31549	-12.29177
C	7.80983	7.11700	-1.10118
H	6.79054	7.22529	-1.46607
H	7.90333	6.15250	-0.60576
H	8.48585	7.13344	-1.95342
H	8.04258	9.20254	-0.65113
H	7.44579	8.23504	0.70114
C	14.37449	6.46595	1.00513
H	15.36269	6.31594	1.43507
H	14.48869	6.96635	0.04646
H	13.92631	5.49101	0.82638
H	13.44926	6.79691	2.92265
H	13.98889	8.26759	2.10474
H	5.97365	1.88090	9.72388
H	8.17109	-0.56916	6.98235
H	7.15901	3.87393	8.94122
H	11.76019	2.73582	3.53987
H	12.16987	4.64151	2.03452
H	9.76366	7.19222	4.47976
H	11.61642	7.42438	-2.48812
H	9.53077	10.88758	-1.09899
H	11.04681	7.96237	-4.82715
H	6.59195	13.53991	-5.71847
H	5.16414	13.44123	-7.72610
H	7.09453	9.85082	-9.00878
H	0.32994	11.48168	-11.74324
H	3.14219	8.36555	-12.52882
H	-1.49181	10.14534	-12.72945
H	0.30844	3.46184	-14.59920
H	-1.47240	1.78225	-14.34528
H	-4.33557	4.84197	-13.48619
H	8.85525	6.97159	2.08029
H	12.81110	8.64562	-0.25976
H	3.54525	11.26058	-7.91192
H	4.25949	10.81122	-12.72950
H	4.31846	0.60777	9.00796
H	7.51896	-2.13648	8.23813
H	2.69033	-1.21964	8.95236
H	5.24354	-7.37452	6.35108
H	3.60990	-8.69056	5.07025
H	0.41897	-6.19055	6.42624
H	0.89145	-9.11641	0.21230
H	-3.05533	-9.68602	1.74815
H	0.08441	-9.40650	-2.10827
H	-7.00574	-10.10049	-2.96361
H	-7.84060	-9.50585	-5.18325
H	-3.86854	-8.63717	-6.51104

H	-6.38156	-6.76229	-12.34308
H	-6.62270	-4.15689	-8.95453
H	-5.84676	-4.86938	-13.79850
H	-5.76384	1.55201	-10.58093
H	-4.51710	3.34529	-11.71043
H	-3.59594	0.75261	-14.98645
H	0.48406	-6.14814	4.01375
H	-0.01667	-10.92401	3.07813
H	-4.89632	-9.39004	-8.34784
H	-8.14190	-5.76834	-8.51259
H	3.24521	6.11558	-12.02174
H	5.26091	4.83429	-12.56689
H	3.70253	4.01485	-16.45311
H	1.68182	5.30646	-15.91311
H	-3.02977	9.27786	-14.60078
H	-5.00938	10.64233	-14.08540
H	-6.26969	7.73283	-11.23136
H	-4.28444	6.38135	-11.72605
H	8.96742	7.22539	-5.81157
H	9.64401	5.23223	-7.07779
H	10.19067	7.63085	-10.56423
H	9.55804	9.63082	-9.28972
H	6.08070	12.40379	-3.61061
H	5.15656	14.22744	-2.24467
H	9.04360	15.02282	-0.67609
H	9.97086	13.20730	-2.04460
H	6.49625	5.17088	6.95537
H	5.27111	7.07165	7.91861
H	8.85150	9.39173	7.89526
H	10.08104	7.49141	6.94486
H	12.24959	1.15923	5.57167
H	13.27716	-0.74935	4.41013
H	9.40009	-2.28988	3.51374
H	8.37261	-0.38379	4.66358
H	7.68225	-3.30030	6.22848
H	9.83795	-4.33185	5.69072
H	8.74552	-7.79458	7.93276
H	6.59518	-6.74746	8.50143
H	1.39083	-3.21494	10.01060
H	-0.73354	-2.04418	10.41471
H	-1.90817	-2.78664	6.38108
H	0.22017	-3.92945	5.96698
H	-2.54003	-1.37199	-14.96939
H	-1.89188	-2.38997	-17.10528
H	-5.79869	-4.05622	-17.50550
H	-6.45876	-3.01725	-15.37658
H	-8.33525	-2.33524	-8.96624
H	-8.82442	-1.42048	-6.74045
H	-5.21578	0.85143	-6.87622
H	-4.70991	-0.08254	-9.09184
H	-6.14991	-11.73102	-1.21856
H	-8.23984	-11.92556	0.06291
H	-7.07860	-8.58995	2.45710
H	-4.99767	-8.38911	1.17421
H	-0.49292	-10.86793	-4.12786
H	1.50555	-10.50204	-5.51290
H	-0.22698	-6.92502	-7.06395
H	-2.20984	-7.27182	-5.66528

Table 8 Cartesian coordinates of ***1h*** *(GFN2-xTB(GBSA(toluene)). B97-3c(COSMO(toluene) single point energy = 10838.55565 Hartree.*

	X	Y	Z

C	10.72263	-0.73229	6.15801
C	9.72936	-0.75884	7.13782
C	9.26380	0.45571	7.66475
C	9.71130	1.64336	7.13111
C	10.65118	1.64843	6.06778
C	11.19299	0.45936	5.62255
C	10.69650	3.00404	5.57292
C	9.75592	3.78938	6.36552
C	11.14495	3.72922	4.50814
C	9.66511	5.09244	5.73954
C	9.19026	3.00500	7.32837
C	8.92702	6.22753	6.00694
C	8.91996	7.26377	5.07785
C	9.66918	7.19467	3.90842
C	10.50104	6.08925	3.68859
C	10.48391	5.03891	4.57874
C	7.21506	11.35726	-0.42640
C	8.29618	10.49631	-0.58672
C	8.83769	10.30698	-1.86272
C	8.20890	10.86731	-2.95224
C	7.01926	11.62547	-2.79086
C	6.57161	11.92851	-1.52002
C	6.47801	11.81537	-4.12119
C	7.41405	11.19740	-5.05644
C	5.36018	12.22945	-4.78541
C	6.81608	11.26634	-6.37036
C	8.45810	10.63938	-4.38092
C	7.19701	10.79740	-7.61078
C	6.26222	10.79018	-8.63873
C	4.96255	11.25205	-8.44805
C	4.61651	11.83890	-7.22335
C	5.53639	11.85590	-6.19719
C	1.68807	7.77304	-12.53226
C	1.11391	8.89399	-11.94222
C	-0.26372	9.11046	-12.05055
C	-1.05065	8.13906	-12.63615
C	-0.45754	6.95964	-13.16219
C	0.91248	6.80207	-13.15345
C	-1.54433	6.07551	-13.51713
C	-2.78502	6.78777	-13.24520
C	-1.79565	4.75800	-13.76747
C	-3.85921	5.82705	-13.36754
C	-2.51416	8.02522	-12.73319
C	-5.22519	5.89696	-13.18011
C	-5.96106	4.71903	-13.14677
C	-5.35907	3.47141	-13.30757
C	-3.99347	3.41528	-13.62618
C	-3.24797	4.57197	-13.63668
C	9.45051	8.13674	2.79188
C	9.00589	7.55096	1.61003
C	8.66776	8.28092	0.48418
C	8.76375	9.67678	0.54916
C	9.22325	10.26398	1.72287
C	9.58422	9.52514	2.84294
C	3.90845	10.90366	-9.41973
C	2.81909	10.21055	-8.89369
C	1.85371	9.61703	-9.68570
C	1.98024	9.72341	-11.07548
C	3.01044	10.49145	-11.59986
C	3.97914	11.09062	-10.80255
C	0.78511	8.78817	-9.02587
C	5.05038	11.92786	-11.45098
C	10.17931	10.22817	4.03178
C	8.17207	7.52535	-0.72125

C	9.66371	-3.26171	7.34890
C	9.03786	-2.01848	7.44039
C	7.65109	-1.97617	7.64836
C	6.92876	-3.14576	7.67843
C	7.56316	-4.39510	7.45243
C	8.93915	-4.44823	7.34219
C	6.49840	-5.35389	7.25308
C	5.23528	-4.64126	7.42040
C	6.27085	-6.59617	6.73452
C	4.17953	-5.55813	7.05135
C	5.47050	-3.32638	7.69600
C	2.80380	-5.45530	7.02711
C	2.06830	-6.46122	6.41226
C	2.68868	-7.57112	5.84615
C	4.07311	-7.73137	5.97404
C	4.81665	-6.73178	6.56550
C	-0.12179	-11.05432	0.88342
C	-0.48650	-10.08690	1.81234
C	-1.70191	-9.40835	1.66329
C	-2.45737	-9.61768	0.53037
C	-2.02516	-10.54006	-0.46117
C	-0.88624	-11.29107	-0.25495
C	-2.92931	-10.38835	-1.58168
C	-3.93164	-9.40706	-1.18987
C	-3.03255	-10.65831	-2.91601
C	-4.69998	-9.08580	-2.37099
C	-3.67827	-8.94392	0.06783
C	-5.71727	-8.18596	-2.61123
C	-6.11736	-7.96595	-3.92326
C	-5.53083	-8.64724	-4.98632
C	-4.55038	-9.61399	-4.73578
C	-4.12417	-9.82259	-3.44057
C	-5.71213	-5.89704	-11.11052
C	-6.28536	-5.77578	-9.85063
C	-6.99978	-4.61534	-9.52750
C	-7.01110	-3.55837	-10.40994
C	-6.34195	-3.65866	-11.66064
C	-5.73964	-4.84625	-12.02353
C	-6.39018	-2.33885	-12.25622
C	-5.80921	-1.57924	-13.23234
C	-7.00056	-0.13192	-11.80693
C	-7.41855	1.07854	-11.29483
C	-6.92752	2.24604	-11.86025
C	-6.05164	2.22683	-12.94618
C	-5.70861	0.99474	-13.52576
C	-6.15061	-0.17525	-12.94529
C	1.92208	-8.43611	4.92621
C	2.34090	-8.40955	3.59852
C	1.61903	-8.99251	2.57213
C	0.41843	-9.63834	2.89196
C	0.03302	-9.72292	4.22420
C	0.76362	-9.14130	5.25321
C	-5.78634	-8.17038	-6.36044
C	-4.68572	-7.65091	-7.03642
C	-4.79743	-6.94415	-8.22149
C	-6.07628	-6.74807	-8.75679
C	-7.16500	-7.34405	-8.13074
C	-7.04816	-8.06156	-6.94749
C	-3.56090	-6.30322	-8.79226
C	-8.26034	-8.73607	-6.36650
C	0.32423	-9.33474	6.67840
C	2.08735	-8.78603	1.15654
C	-3.47193	9.03594	-12.31603
C	-0.82630	3.72102	-14.08113
C	-4.97871	-2.05184	-14.32746

C	-8.33567	-1.72341	-9.12682	C	-9.92461	-0.80501	-7.00249
C	-2.20675	-11.56130	-3.70143	C	-10.27762	-0.53959	-8.32095
C	-4.44380	-7.96544	0.82386	C	-9.49707	-0.98740	-9.36792
C	7.26066	-7.59783	6.37272	C	-10.75732	-0.29780	-5.86174
C	4.54506	-2.22282	7.89309	C	-2.55719	-3.56694	-17.55194
C	12.06449	3.30098	3.46707	C	-5.82940	-8.10327	0.92622
C	8.19434	3.33152	8.33717	C	-6.57476	-7.19757	1.65260
C	4.15696	12.85647	-4.26125	C	-5.96924	-6.11558	2.28474
C	9.58056	9.86109	-4.88177	C	-4.59228	-5.97045	2.16782
C	-7.54390	-2.19952	-10.24886	C	-3.83589	-6.88213	1.45562
C	-7.14397	-1.49069	-11.34419	C	-1.95571	-12.85417	-3.24046
C	-3.36654	9.68336	-11.08502	C	-1.16519	-13.72012	-3.96929
C	-4.29528	10.63334	-10.71009	C	-0.58930	-13.32242	-5.17087
C	-5.35202	10.97496	-11.54761	C	-0.83461	-12.03173	-5.62597
C	-5.46100	10.32325	-12.77091	C	-1.63291	-11.16293	-4.90837
C	-4.54063	9.36608	-13.15018	C	0.24344	-14.27390	-5.97881
C	-6.32731	12.04067	-11.14060	C	-6.79574	-5.12704	3.05598
C	0.15449	3.96557	-15.04364	C	0.87268	0.83409	9.51062
C	1.09044	3.00059	-15.35757	C	-0.07723	2.00631	9.74452
C	1.08236	1.76318	-14.72321	C	-1.09737	1.70465	10.84057
C	0.11080	1.52389	-13.75726	C	-2.06186	2.86672	11.07019
C	-0.82978	2.48359	-13.43747	C	-3.06492	2.56303	12.17746
C	2.07614	0.69835	-15.08610	C	-7.64676	-4.24367	2.13381
C	3.52786	12.35512	-3.12180	C	-6.79645	-3.36628	1.21829
C	2.37992	12.94821	-2.63588	C	-7.65734	-2.47763	0.32308
C	1.82782	14.06044	-3.26297	C	-6.81731	-1.60838	-0.61061
C	2.45562	14.55835	-4.39884	C	-7.68613	-0.72048	-1.49420
C	3.59692	13.96275	-4.89970	C	9.73382	-11.94055	5.71272
C	9.37502	8.81335	-5.77874	C	10.75381	-13.01954	5.35630
C	10.43664	8.04741	-6.21728	C	10.25607	-14.41567	5.72666
C	11.73266	8.30481	-5.78314	C	11.29116	-15.51532	5.48142
C	11.93674	9.35585	-4.89637	C	11.58674	-15.74898	4.00360
C	10.87854	10.11870	-4.44206	C	1.47798	-0.33118	-16.05653
C	0.60798	14.72861	-2.69947	C	1.10773	0.28981	-17.40276
C	13.25366	2.65328	3.80423	C	0.58928	-0.73424	-18.42310
C	14.13237	2.24036	2.82250	C	1.62931	-1.17181	-19.45677
C	13.85260	2.45210	1.47684	C	2.79991	-1.94907	-18.86596
C	12.66070	3.08439	1.14133	C	-5.73528	13.44783	-11.31140
C	11.78021	3.50706	2.11732	C	-5.48931	13.79503	-12.77774
C	14.78607	1.96277	0.40854	C	-4.76046	15.12620	-12.97293
C	7.03433	4.03191	8.00974	C	-5.51511	16.36126	-12.47538
C	6.08527	4.30818	8.97477	C	-6.83120	16.60159	-13.20700
C	6.26561	3.90361	10.29271	C	-2.99738	-2.94634	-18.88380
C	7.42363	3.20522	10.61742	C	-2.18540	-3.48748	-20.05781
C	8.37106	2.91348	9.65668	C	-2.62160	-2.87674	-21.38786
C	5.26405	4.25276	11.35472	C	-1.82429	-3.43195	-22.56656
C	3.44994	-2.03924	7.04999	C	-2.25573	-2.81020	-23.88992
C	2.60949	-0.95578	7.21680	C	-10.21697	1.03963	-5.33892
C	2.83383	-0.02327	8.22305	C	-11.03679	1.56117	-4.16118
C	3.92038	-0.21290	9.07030	C	-10.47433	2.86610	-3.60131
C	4.76633	-1.29140	8.90922	C	-11.28980	3.38254	-2.41728
C	8.27867	-7.92956	7.26743	C	-10.71770	4.67722	-1.85078
C	9.23199	-8.86968	6.93073	C	5.64454	5.58006	12.02533
C	9.21052	-9.49720	5.68986	C	4.77632	5.91878	13.23732
C	8.19919	-9.16135	4.79692	C	3.31179	6.18016	12.88934
C	7.23524	-8.23033	5.13013	C	2.52130	6.67664	14.09886
C	10.23098	-10.53876	5.33573	C	1.05266	6.91185	13.76396
C	1.90879	1.14143	8.42178	C	-0.61902	-15.00733	-7.01437
C	-3.76670	-1.44419	-14.65300	C	0.20483	-15.98054	-7.85402
C	-2.98807	-1.93705	-15.68227	C	-0.64653	-16.70480	-8.89495
C	-3.38917	-3.04655	-16.41711	C	0.17726	-17.67747	-9.73681
C	-4.60157	-3.64852	-16.09615	C	-0.67536	-18.39530	-10.77700
C	-5.38368	-3.16426	-15.06790	C	12.88676	7.49117	-6.29116
C	-7.97364	-1.97830	-7.80440	C	13.42448	8.07023	-7.60640
C	-8.75687	-1.52098	-6.76296	C	14.60195	7.25642	-8.13907

C	15.22703	7.84749	-9.40395
C	0.99426	15.72611	-1.59957
C	-0.22911	16.41589	-1.00073
C	0.14958	17.39260	0.11075
C	-1.07314	18.08099	0.71450
C	-0.69066	19.04763	1.82960
C	14.40870	0.53701	-0.01677
C	15.22340	0.02471	-1.20483
C	16.70252	-0.18767	-0.88690
C	17.45083	-0.81909	-2.05979
C	18.92735	-1.03320	-1.74585
C	14.32693	7.74928	-10.63467
C	15.02903	8.26375	-11.88664
H	-6.59610	11.90121	-10.09035
H	-7.23720	11.95991	-11.73890
H	2.95485	1.15468	-15.54712
H	2.39760	0.17812	-14.18030
H	-0.06443	13.97813	-2.27752
H	0.07438	15.25877	-3.49110
H	14.72831	2.61948	-0.46234
H	15.81044	1.97369	0.78277
H	4.27091	4.33972	10.91259
H	5.23943	3.46707	12.11333
H	11.16529	-10.33814	5.86460
H	10.43282	-10.50976	4.26255
H	2.48480	2.02165	8.71612
H	1.39069	1.36930	7.48786
H	-10.74826	-1.02562	-5.04713
H	-11.79104	-0.16299	-6.18697
H	-2.65820	-4.65284	-17.61623
H	-1.50479	-3.33242	-17.37993
H	0.71271	-15.00850	-5.32093
H	1.03342	-13.72632	-6.49790
H	-6.14303	-4.49191	3.65835
H	-7.46309	-5.66736	3.73285
H	1.39244	0.59445	10.44127
H	0.29843	-0.04854	9.21838
H	0.50117	2.89086	10.02337
H	-0.60356	2.23698	8.81477
H	-1.66729	0.81302	10.56724
H	-0.57045	1.48485	11.77266
H	-2.59935	3.07841	10.14246
H	-1.49266	3.76192	11.33238
H	-3.65715	1.68533	11.92574
H	-3.74085	3.40298	12.32337
H	-2.55007	2.36923	13.11655
H	-8.28750	-3.60753	2.74944
H	-8.29428	-4.87715	1.52357
H	-6.16199	-4.00198	0.59554
H	-6.13992	-2.73786	1.82560
H	-8.28343	-1.83435	0.94704
H	-8.32285	-3.10608	-0.27455
H	-6.14601	-0.98327	-0.01646
H	-6.19733	-2.25195	-1.24013
H	-8.28705	-0.04707	-0.88611
H	-8.35788	-1.32831	-2.09687
H	-7.06878	-0.12290	-2.16176
H	8.79423	-12.13949	5.19146
H	9.52729	-11.97040	6.78548
H	10.95876	-12.97303	4.28530
H	11.68987	-12.82003	5.88430
H	9.35078	-14.64000	5.15691
H	9.98661	-14.42078	6.78635
H	10.91690	-16.44607	5.91568
H	12.21822	-15.25957	6.00058

H	10.66905	-15.97179	3.46264
H	12.26741	-16.58949	3.88397
H	12.04892	-14.87403	3.55363
H	2.20246	-1.13326	-16.20342
H	0.58506	-0.76862	-15.60337
H	1.97917	0.80203	-17.81646
H	0.34068	1.04811	-17.22840
H	-0.25342	-0.30020	-18.96609
H	0.21620	-1.61508	-17.89637
H	2.00813	-0.28875	-19.97784
H	1.13137	-1.79905	-20.20095
H	3.45690	-2.29954	-19.65996
H	2.44263	-2.81520	-18.31188
H	3.38501	-1.32370	-18.19657
H	-4.79193	13.50869	-10.76360
H	-6.42744	14.16816	-10.87220
H	-6.44110	13.81381	-13.31162
H	-4.87827	13.00723	-13.22628
H	-4.55240	15.25198	-14.03900
H	-3.79822	15.07574	-12.45594
H	-4.86916	17.23174	-12.61906
H	-5.70437	16.27243	-11.40415
H	-6.66607	16.64957	-14.28175
H	-7.27418	17.54229	-12.88598
H	-7.54390	15.80665	-13.00301
H	-2.88324	-1.86138	-18.82820
H	-4.05725	-3.15848	-19.04431
H	-1.12663	-3.27448	-19.89279
H	-2.30015	-4.57349	-20.10628
H	-2.49544	-1.79195	-21.34649
H	-3.68415	-3.07812	-21.54651
H	-0.76080	-3.23877	-22.40643
H	-1.95891	-4.51550	-22.61439
H	-2.11075	-1.73178	-23.86985
H	-3.30847	-3.00961	-24.08045
H	-1.67423	-3.21958	-24.71329
H	-10.23027	1.77346	-6.14840
H	-9.17704	0.90931	-5.03072
H	-12.06964	1.72053	-4.48134
H	-11.05020	0.80793	-3.36962
H	-10.46258	3.62335	-4.38932
H	-9.44105	2.70557	-3.28357
H	-12.32145	3.55142	-2.73596
H	-11.30703	2.62131	-1.63331
H	-10.70870	5.45593	-2.61098
H	-9.69667	4.52489	-1.50624
H	-11.31557	5.02470	-1.01081
H	6.68738	5.51918	12.34768
H	5.57555	6.38510	11.28969
H	4.83503	5.10384	13.96337
H	5.18761	6.81261	13.71434
H	3.25755	6.92763	12.09362
H	2.85003	5.26300	12.51874
H	2.96350	7.60812	14.46140
H	2.59570	5.94162	14.90436
H	0.51342	7.27227	14.63742
H	0.58462	5.98813	13.42963
H	0.95529	7.65129	12.97130
H	-1.09484	-14.27270	-7.66843
H	-1.41362	-15.55136	-6.49784
H	1.00426	-15.43350	-8.36042
H	0.67453	-16.71691	-7.19697
H	-1.11651	-15.96876	-9.55218
H	-1.44593	-17.25303	-8.38998
H	0.97756	-17.12952	-10.24049

H	0.64500	-18.41544	-9.08036
H	-1.13546	-17.67828	-11.45415
H	-1.46680	-18.96557	-10.29453
H	-0.06722	-19.08039	-11.36395
H	13.68895	7.48177	-5.54997
H	12.56736	6.46045	-6.46142
H	13.74072	9.10293	-7.43962
H	12.61675	8.08443	-8.33989
H	14.27281	6.23439	-8.34359
H	15.36999	7.20522	-7.36257
H	16.15897	7.31490	-9.61232
H	15.48038	8.89550	-9.22517
H	1.53695	15.19766	-0.81214
H	1.66952	16.47670	-2.01766
H	-0.90910	15.66011	-0.59922
H	-0.76274	16.95431	-1.78804
H	0.68413	16.85395	0.89735
H	0.82850	18.14993	-0.28933
H	-1.75411	17.32330	1.11020
H	-1.60470	18.62508	-0.07013
H	-0.17761	18.52097	2.63197
H	-1.57657	19.52457	2.24349
H	-0.02765	19.82353	1.45181
H	14.53828	-0.13676	0.83357
H	13.34913	0.52516	-0.28585
H	14.79575	-0.92754	-1.53050
H	15.12962	0.72781	-2.03627
H	16.79340	-0.83606	-0.01164
H	17.17046	0.76822	-0.64417
H	17.35677	-0.17348	-2.93663
H	16.99064	-1.77926	-2.30669
H	19.41213	-0.08481	-1.52233
H	19.43718	-1.48784	-2.59266
H	19.04313	-1.68770	-0.88403
H	14.03667	6.70641	-10.78550
H	13.41638	8.32931	-10.47434
H	15.92977	7.68607	-12.08551
H	15.31314	9.30700	-11.76256
H	14.37428	8.18733	-12.75229
C	0.79620	-10.69498	7.19902
H	1.88251	-10.75230	7.17146
H	0.39461	-11.49941	6.58624
H	0.46773	-10.84188	8.22575
H	-0.76583	-9.28705	6.73781
H	0.74011	-8.55171	7.31294
C	1.40939	-7.55993	0.54066
H	1.78352	-7.38539	-0.46595
H	1.60283	-6.67447	1.14331
H	0.33386	-7.71351	0.48611
H	1.86628	-9.65570	0.53918
H	3.16909	-8.63402	1.15680
C	-8.53466	-10.05143	-7.10047
H	-8.69701	-9.87092	-8.16109
H	-9.41801	-10.53685	-6.69079
H	-7.68743	-10.72595	-6.99321
H	-9.13027	-8.08195	-6.46530
H	-8.10567	-8.94848	-5.30834
C	-3.39531	-4.88035	-8.25328
H	-2.47930	-4.43455	-8.63521
H	-3.35337	-4.88741	-7.16572
H	-4.23436	-4.26087	-8.56271
H	-3.60610	-6.26368	-9.87953
H	-2.68845	-6.89656	-8.50883
C	1.25785	7.34474	-8.83916
H	2.16871	7.31664	-8.24447

H	1.46197	6.88763	-9.80518
H	0.49315	6.75765	-8.33462
H	-0.12405	8.78383	-9.62669
H	0.54970	9.21757	-8.04903
C	5.95896	11.11469	-12.37529
H	6.44408	10.30728	-11.83080
H	6.72973	11.75435	-12.80028
H	5.38887	10.67505	-13.19020
H	5.65283	12.41388	-10.68192
H	4.56697	12.71185	-12.04201
C	9.30595	6.77932	-1.42877
H	8.92458	6.25853	-2.30470
H	9.75962	6.05039	-0.76135
H	10.08226	7.46864	-1.75269
H	7.68789	8.20110	-1.42663
H	7.42708	6.79475	-0.39254
C	11.67489	10.46875	3.80804
H	12.10734	10.98721	4.66125
H	11.83611	11.07071	2.91602
H	12.19296	9.52034	3.67994
H	10.05157	9.62902	4.93309
H	9.67937	11.18852	4.17960
H	11.08359	-1.66353	5.74690
H	8.51962	0.45884	8.44756
H	11.91771	0.44604	4.82164
H	8.32619	6.29928	6.90171
H	8.27437	8.11507	5.24282
H	11.11848	6.05991	2.80265
H	6.82598	11.52934	0.56742
H	9.71059	9.68471	-1.98947
H	5.70537	12.55618	-1.37088
H	8.18447	10.39030	-7.77119
H	6.52785	10.34258	-9.58395
H	3.61752	12.22475	-7.07701
H	2.75792	7.63521	-12.45660
H	-0.69540	10.01422	-11.64459
H	1.37309	5.91797	-13.56867
H	-5.71306	6.84553	-13.01025
H	-7.02517	4.77402	-12.96807
H	-3.51982	2.45939	-13.78640
H	8.88589	6.47615	1.58510
H	9.30705	11.34216	1.76038
H	2.75937	10.08136	-7.82129
H	3.06848	10.60843	-12.67352
H	10.73820	-3.30732	7.24487
H	7.13852	-1.02709	7.69546
H	9.44762	-5.39091	7.20202
H	2.30474	-4.59134	7.44055
H	0.99695	-6.35220	6.31698
H	4.54345	-8.61952	5.57746
H	0.78433	-11.62291	1.04122
H	-2.00097	-8.68907	2.41198
H	-0.56365	-12.02074	-0.98307
H	-6.16039	-7.62250	-1.80324
H	-6.86144	-7.20989	-4.13155
H	-4.11667	-10.15706	-5.56297
H	-5.22237	-6.82156	-11.38336
H	-7.48319	-4.54580	-8.56406
H	-5.25487	-4.95081	-12.98299
H	-8.06799	1.12073	-10.43285
H	-7.18399	3.19143	-11.40567
H	-5.07979	0.97146	-14.40348
H	3.24160	-7.86120	3.35612
H	-0.88455	-10.24394	4.46419
H	-3.70948	-7.75072	-6.58080

H	-8.14592	-7.21604	-8.56942
H	-2.57347	9.41028	-10.40461
H	-4.20291	11.11613	-9.74706
H	-6.27860	10.56863	-13.43447
H	-4.62846	8.87711	-14.11007
H	0.16116	4.91676	-15.55671
H	1.84112	3.21053	-16.10666
H	0.09698	0.57348	-13.24140
H	-1.54991	2.29067	-12.65537
H	3.93393	11.47848	-2.63813
H	1.89961	12.54005	-1.75729
H	2.04130	15.42202	-4.90030
H	4.07773	14.36893	-5.77808
H	8.36973	8.58786	-6.10424
H	10.25762	7.22996	-6.90206
H	12.93789	9.57336	-4.55061
H	11.05480	10.93605	-3.75721
H	13.48968	2.49628	4.84713
H	15.05311	1.74785	3.10330
H	12.42043	3.24749	0.09972
H	10.84513	3.96810	1.83607
H	6.87214	4.33283	6.98490
H	5.18732	4.84440	8.70010
H	7.58148	2.88310	11.63753
H	9.27050	2.37920	9.92802
H	3.28396	-2.73670	6.24203
H	1.77140	-0.82447	6.54628
H	4.10951	0.50009	9.86045
H	5.59682	-1.43162	9.58607
H	8.30148	-7.45334	8.23738
H	10.01033	-9.11844	7.63887
H	8.17140	-9.63182	3.82380
H	6.47696	-7.95917	4.41032
H	-3.41715	-0.60665	-14.06806
H	-2.04902	-1.45551	-15.91601
H	-4.93175	-4.51147	-16.65783
H	-6.32930	-3.63310	-14.83570
H	-7.05727	-2.50982	-7.59353
H	-8.45915	-1.72647	-5.74430
H	-11.17947	0.01950	-8.52880
H	-9.79224	-0.79226	-10.38918
H	-6.30989	-8.94542	0.44846
H	-7.64425	-7.33211	1.73583
H	-4.10273	-5.13138	2.64242
H	-2.76976	-6.73645	1.35973
H	-2.40469	-13.17724	-2.31194
H	-0.98656	-14.72012	-3.59877
H	-0.38718	-11.70182	-6.55333
H	-1.78511	-10.15401	-5.26266

*Table 9 Cartesian coordinates of **1f** (GFN2-xTB(GBSA(toluene)). B97-3c(COSMO(toluene) single point energy = 10838.552286 Hartree.*

	X	Y	Z
C	6.59342	0.83559	7.42698
C	7.48691	-0.21339	7.21243
C	8.80117	0.08451	6.81801
C	9.14263	1.38434	6.51859
C	8.19929	2.43439	6.68688
C	6.94285	2.15798	7.18585
C	8.80159	3.62283	6.12810
C	10.12390	3.24629	5.64781
C	8.47853	4.87714	5.69526
C	10.61690	4.34958	4.85386
C	10.36037	1.92503	5.89568
C	11.73687	4.51405	4.06557
C	11.80445	5.61290	3.21608
C	10.79140	6.56530	3.17454
C	9.70306	6.45035	4.05139
C	9.59554	5.33843	4.85755
C	10.93187	9.08063	-2.68458
C	10.30047	9.68714	-1.60364
C	9.44774	10.77395	-1.81783
C	9.15702	11.15194	-3.11300
C	9.67911	10.41466	-4.20829
C	10.62497	9.43308	-3.99372
C	8.95966	10.86442	-5.37955
C	8.08580	11.95445	-4.95866
C	8.69331	10.51852	-6.67163
C	7.28758	12.32726	-6.10817
C	8.18802	12.13971	-3.61020
C	6.28820	13.25708	-6.31544
C	5.54332	13.19039	-7.48766
C	5.78966	12.21928	-8.45355
C	6.87311	11.34881	-8.29347
C	7.62511	11.41236	-7.14048
C	0.55237	10.85070	-12.35920
C	1.85945	10.40411	-12.19889
C	2.25881	9.20595	-12.80428
C	1.31459	8.40969	-13.41530
C	-0.04692	8.81743	-13.45213
C	-0.40910	10.06411	-12.98475
C	-0.80206	7.67628	-13.92096
C	0.15905	6.62840	-14.23599
C	-2.06907	7.16898	-13.91434
C	-0.59092	5.41190	-14.44781
C	1.42898	7.05241	-13.96872
C	-0.21742	4.10702	-14.69618
C	-1.17145	3.10465	-14.57500
C	-2.49049	3.38951	-14.22724
C	-2.89543	4.72766	-14.10850
C	-1.95345	5.72894	-14.19323
C	10.71972	7.54370	2.07184
C	9.53872	7.51416	1.33114
C	9.36042	8.24580	0.17273
C	10.41771	9.04693	-0.27369
C	11.56942	9.13216	0.49406
C	11.74481	8.39952	1.66404
C	4.79874	11.95060	-9.51553
C	3.52644	11.60133	-9.06970
C	2.53249	11.14553	-9.91697
C	2.81731	11.06315	-11.28670
C	4.06445	11.47461	-11.74021
C	5.06847	11.91001	-10.88330
C	1.25364	10.63711	-9.30637
C	6.40244	12.32096	-11.44708
C	13.01489	8.57012	2.45485
C	8.08368	8.07893	-0.60609
C	7.49345	-2.47384	6.23562
C	6.99499	-1.59771	7.20064
C	5.93190	-2.01661	8.01611
C	5.32687	-3.23142	7.77200
C	5.80283	-4.07223	6.73039
C	6.91815	-3.71370	6.00303
C	4.88838	-5.18427	6.64494
C	3.91636	-5.02815	7.72030
C	4.55403	-6.23907	5.84851
C	2.92323	-6.06912	7.54658

C	4.15466	-3.86648	8.39823
C	1.73280	-6.37417	8.17601
C	0.89377	-7.33021	7.61040
C	1.22099	-7.98136	6.42451
C	2.45886	-7.71984	5.82289
C	3.30471	-6.79105	6.38395
C	-3.50353	-10.38905	2.22319
C	-2.17578	-10.02479	2.40328
C	-1.37089	-9.78991	1.28119
C	-1.92145	-9.85964	0.02378
C	-3.29532	-10.17457	-0.15302
C	-4.06976	-10.47041	0.95112
C	-3.57315	-9.97944	-1.56358
C	-2.33750	-9.50886	-2.17786
C	-4.58616	-9.96022	-2.48082
C	-2.63806	-9.19136	-3.55222
C	-1.33040	-9.47124	-1.26047
C	-1.87483	-8.68810	-4.58405
C	-2.50488	-8.36260	-5.77460
C	-3.86931	-8.57907	-5.97679
C	-4.62048	-9.18108	-4.95801
C	-4.02168	-9.44525	-3.74093
C	-4.90155	-5.76168	-12.00442
C	-5.06441	-5.56414	-10.63490
C	-5.25703	-4.25462	-10.16743
C	-5.20713	-3.19748	-11.04409
C	-4.87974	-3.40267	-12.41118
C	-4.78335	-4.69317	-12.89126
C	-4.63787	-2.08595	-12.96682
C	-4.05015	-1.45583	-14.02704
C	-4.55719	0.18214	-12.41522
C	-4.65836	1.45284	-11.88918
C	-4.05120	2.49962	-12.56512
C	-3.35522	2.29768	-13.75708
C	-3.35926	1.02695	-14.34959
C	-3.95395	-0.02607	-13.68481
C	0.23261	-8.75749	5.65371
C	-0.99447	-8.14518	5.40896
C	-1.88360	-8.59980	4.44845
C	-1.52199	-9.71974	3.69348
C	-0.36553	-10.41309	4.03784
C	0.51718	-9.96747	5.00943
C	-4.42570	-8.05843	-7.23988
C	-3.67156	-8.24421	-8.40064
C	-3.87422	-7.53617	-9.57276
C	-4.92969	-6.60797	-9.60300
C	-5.77194	-6.53812	-8.49530
C	-5.54456	-7.22154	-7.31075
C	-2.93271	-7.70710	-10.73874
C	-6.47986	-6.96336	-6.16093
C	1.73227	-10.79535	5.33986
C	-3.16499	-7.84505	4.22552
C	2.66686	6.32768	-14.20561
C	-3.30348	7.88725	-13.64068
C	-3.58053	-2.05541	-15.26562
C	-5.60368	-1.20864	-9.45605
C	-5.97055	-10.35270	-2.27772
C	0.06320	-9.08749	-1.41651
C	5.22206	-6.72864	4.65293
C	3.39782	-3.31791	9.51131
C	7.25302	5.61156	5.96141
C	11.55677	1.16567	5.57113
C	9.22842	9.41630	-7.45440
C	7.46142	13.05918	-2.74875
C	-5.26590	-1.76006	-10.75818
C	-4.91471	-1.11992	-11.90923
C	3.67699	6.22841	-13.25058
C	4.84916	5.55346	-13.53652
C	5.05621	4.96982	-14.78033
C	4.03995	5.05496	-15.72704
C	2.86220	5.71514	-15.44570
C	6.32499	4.23417	-15.09912
C	-3.56128	9.09273	-14.29482
C	-4.72588	9.79195	-14.04932
C	-5.66275	9.32181	-13.13547
C	-5.39900	8.12743	-12.47469
C	-4.24121	7.41627	-12.72277
C	-6.94566	10.06343	-12.89950
C	9.34757	8.14769	-6.88495
C	9.81232	7.08477	-7.63095
C	10.17183	7.24843	-8.96543
C	10.06207	8.51402	-9.52866
C	9.59543	9.58438	-8.78883
C	6.07990	13.20819	-2.86701
C	5.39298	14.06901	-2.03427
C	6.05891	14.80931	-1.06355
C	7.43674	14.66180	-0.94885
C	8.13024	13.79532	-1.77069
C	10.61295	6.06434	-9.77538
C	6.74871	5.66806	7.26215
C	5.58578	6.35832	7.53704
C	4.88216	7.00585	6.52666
C	5.37814	6.93911	5.23004
C	6.54463	6.25517	4.94742
C	3.64231	7.79078	6.84005
C	12.81352	1.67947	5.89452
C	13.96038	0.96884	5.60350
C	13.89408	-0.26943	4.97352
C	12.64318	-0.77082	4.63279
C	11.49014	-0.07021	4.92891
C	15.14630	-1.01900	4.62468
C	3.04273	-4.13305	10.58677
C	2.32201	-3.62307	11.64794
C	1.92443	-2.29051	11.66798
C	2.26868	-1.48202	10.59057
C	2.99715	-1.98208	9.52928
C	5.62250	-5.84589	3.64986
C	6.26557	-6.31218	2.52104
C	6.53686	-7.66694	2.35979
C	6.13009	-8.54612	3.35648
C	5.47299	-8.08968	4.48280
C	7.29729	-8.15409	1.16136
C	1.09744	-1.75146	12.79812
C	-2.31224	-1.77770	-15.77439
C	-1.88492	-2.36117	-16.95103
C	-2.70819	-3.22625	-17.66263
C	-3.97085	-3.50713	-17.15143
C	-4.40140	-2.93675	-15.97043
C	-6.70549	-1.69380	-8.75073
C	-7.03408	-1.17096	-7.51570
C	-6.26973	-0.16299	-6.93877
C	-5.16598	0.31241	-7.63859
C	-4.83565	-0.19822	-8.87835
C	-6.65679	0.43089	-5.61606
C	-2.22800	-3.87831	-18.92600
C	0.41872	-7.91687	-2.08542
C	1.74584	-7.55627	-2.21129
C	2.75672	-8.34808	-1.67774
C	2.40062	-9.51373	-1.00871
C	1.07598	-9.87824	-0.87272

C	-6.26841	-11.53305	-1.59461	C	2.22834	13.68707	8.86338
C	-7.57844	-11.91460	-1.38755	C	3.75246	20.57313	0.30231
C	-8.63248	-11.12960	-1.84340	C	3.73117	21.96505	-0.31909
C	-8.33478	-9.95086	-2.51717	H	7.12769	4.57016	-14.43984
C	-7.02672	-9.56584	-2.73635	H	6.61942	4.44687	-16.12982
C	-10.05436	-11.56943	-1.65246	H	-6.79361	11.13195	-13.06681
C	4.20023	-7.98369	-1.86293	H	-7.27265	9.92226	-11.86696
C	-0.39876	-1.90272	12.49247	H	11.29866	5.45590	-9.18344
C	-1.27050	-1.37949	13.63149	H	11.13390	6.39603	-10.67571
C	-2.76007	-1.51894	13.32343	H	3.08204	7.29755	7.63762
C	-3.63586	-1.00162	14.46314	H	3.00279	7.84544	5.95652
C	-5.12119	-1.14174	14.14926	H	14.94567	-2.09249	4.60984
C	4.74274	-8.59026	-3.16385	H	15.91514	-0.82545	5.37603
C	6.22063	-8.27135	-3.37536	H	7.02817	-7.56020	0.28493
C	6.74912	-8.86003	-4.68203	H	7.04867	-9.19844	0.96062
C	8.22707	-8.54466	-4.90432	H	1.32939	-2.29046	13.71917
C	8.74496	-9.13587	-6.21059	H	1.32409	-0.69449	12.95417
C	8.80889	-8.03451	1.39672	H	-5.76515	0.76947	-5.08385
C	9.61338	-8.49329	0.18277	H	-7.15579	-0.32216	-5.00219
C	11.11731	-8.36347	0.41718	H	-1.50301	-3.23124	-19.42449
C	11.95809	-8.73379	-0.80630	H	-3.06833	-4.03642	-19.60558
C	11.90518	-10.21902	-1.14902	H	-10.71081	-10.69770	-1.60717
C	-8.04438	9.56022	-13.84505	H	-10.14437	-12.11890	-0.71449
C	-9.36183	10.30235	-13.63387	H	4.78659	-8.35804	-1.02164
C	-10.46036	9.79807	-14.56764	H	4.30920	-6.89759	-1.90072
C	-11.77882	10.54134	-14.36105	H	-0.62301	-2.95767	12.31669
C	-12.87199	10.03311	-15.29433	H	-0.63290	-1.35971	11.57356
C	6.14130	2.71914	-14.94192	H	-1.03868	-1.93052	14.54651
C	7.38629	1.94861	-15.37530	H	-1.03660	-0.32726	13.81237
C	7.20704	0.43848	-15.23400	H	-2.99327	-0.96550	12.41017
C	8.45055	-0.33228	-15.67321	H	-2.99345	-2.57069	13.13919
C	8.27496	-1.83783	-15.51062	H	-3.40245	0.04982	14.64850
C	-1.56768	-5.23016	-18.62452	H	-3.40470	-1.55679	15.37568
C	-1.04709	-5.90412	-19.89180	H	-5.37672	-0.57994	13.25288
C	-0.39987	-7.25629	-19.59911	H	-5.72416	-0.76608	14.97325
C	0.13612	-7.92455	-20.86388	H	-5.37934	-2.18576	13.98292
C	0.77386	-9.27723	-20.56748	H	4.16207	-8.20649	-4.00621
C	-7.60357	1.62169	-5.81684	H	4.60466	-9.67394	-3.13687
C	-8.03761	2.23876	-4.48938	H	6.35968	-7.18716	-3.38544
C	-8.97481	3.42813	-4.69065	H	6.79877	-8.67219	-2.53951
C	-9.42298	4.04173	-3.36538	H	6.16722	-8.46317	-5.51787
C	-10.35657	5.22903	-3.57382	H	6.61050	-9.94414	-4.67090
C	15.67145	-0.58789	3.24881	H	8.36688	-7.46073	-4.91662
C	16.95647	-1.32225	2.87336	H	8.81020	-8.94447	-4.07166
C	17.47707	-0.88854	1.50399	H	8.18409	-8.74326	-7.05658
C	18.71270	-1.66667	1.04742	H	9.79556	-8.89123	-6.35187
C	19.95529	-1.37142	1.88091	H	8.64221	-10.21930	-6.20634
C	-10.49551	-12.46658	-2.81703	H	9.08454	-8.63448	2.26765
C	-11.96853	-12.87007	-2.74501	H	9.05306	-6.99398	1.62427
C	-12.29094	-13.79933	-1.57602	H	9.36273	-9.53236	-0.03763
C	-13.74274	-14.27378	-1.60941	H	9.33322	-7.88870	-0.68351
C	-14.06977	-15.19258	-0.43750	H	11.40883	-8.99663	1.25886
C	5.31536	15.77477	-0.18786	H	11.33902	-7.32871	0.69229
C	5.28236	17.17142	-0.82218	H	12.99709	-8.45669	-0.60927
C	4.53074	18.17399	0.05017	H	11.61956	-8.14973	-1.66579
C	4.50176	19.56880	-0.57139	H	12.21003	-10.81789	-0.29274
C	9.39705	5.21755	-10.17527	H	12.57612	-10.43881	-1.97709
C	9.74907	4.00160	-11.03251	H	10.90175	-10.51967	-1.43911
C	10.63494	2.98617	-10.31020	H	-7.71376	9.68921	-14.87857
C	10.76413	1.66260	-11.06928	H	-8.19674	8.49092	-13.67966
C	9.52533	0.78166	-10.94629	H	-9.20635	11.37074	-13.80422
C	4.00082	9.21366	7.28876	H	-9.68495	10.17757	-12.59718
C	2.75776	10.04189	7.60482	H	-10.13700	9.92010	-15.60453
C	3.11194	11.45100	8.07602	H	-10.61819	8.73029	-14.39575
C	1.86959	12.28575	8.38168	H	-11.62177	11.60884	-14.53482

H	-12.10253	10.41977	-13.32429
H	-12.57700	10.16244	-16.33385
H	-13.79950	10.57765	-15.13039
H	-13.05973	8.97507	-15.12196
H	5.91075	2.49100	-13.89835
H	5.28825	2.39750	-15.54419
H	8.24012	2.26969	-14.77401
H	7.61128	2.18666	-16.41820
H	6.98146	0.19561	-14.19277
H	6.35370	0.11849	-15.83748
H	9.30759	-0.00153	-15.08177
H	8.66484	-0.10396	-16.72029
H	8.07781	-2.08919	-14.47025
H	9.17275	-2.36480	-15.82685
H	7.43953	-2.19198	-16.11140
H	-2.29469	-5.88279	-18.13528
H	-0.74111	-5.07705	-17.92632
H	-1.87399	-6.04447	-20.59263
H	-0.31481	-5.25091	-20.37321
H	-1.13479	-7.91408	-19.12823
H	0.42032	-7.11823	-18.88999
H	-0.68240	-8.05816	-21.57556
H	0.87613	-7.27011	-21.33128
H	0.04605	-9.95352	-20.12333
H	1.60325	-9.16395	-19.87203
H	1.15134	-9.73116	-21.48128
H	-8.48529	1.28790	-6.36949
H	-7.10167	2.37822	-6.42495
H	-8.54240	1.47996	-3.88601
H	-7.15366	2.56532	-3.93554
H	-9.85452	3.10319	-5.25219
H	-8.46702	4.19055	-5.28689
H	-9.93297	3.28034	-2.76992
H	-8.54412	4.36627	-2.80259
H	-11.24781	4.92285	-4.11824
H	-9.85909	6.01044	-4.14505
H	-10.66500	5.64622	-2.61752
H	15.85657	0.48902	3.25796
H	14.90353	-0.78273	2.49595
H	17.71249	-1.12437	3.63516
H	16.76756	-2.39877	2.86209
H	17.71284	0.17837	1.52801
H	16.68253	-1.03246	0.76665
H	18.91941	-1.40719	0.00578
H	18.49777	-2.73751	1.08336
H	20.16479	-0.30345	1.88596
H	20.81867	-1.88753	1.46587
H	19.82579	-1.70263	2.90812
H	-10.32266	-11.92870	-3.75294
H	-9.87198	-13.36367	-2.83414
H	-12.58655	-11.97143	-2.67391
H	-12.23265	-13.37703	-3.67709
H	-12.11169	-13.28277	-0.63124
H	-11.62822	-14.66782	-1.61306
H	-14.40592	-13.40537	-1.58442
H	-13.92839	-14.80370	-2.54708
H	-13.91618	-14.67593	0.50798
H	-13.43104	-16.07364	-0.45331
H	-15.10633	-15.51969	-0.48512
H	5.80035	15.83932	0.78870
H	4.29074	15.42748	-0.03807
H	4.80371	17.10845	-1.80247
H	6.30683	17.51806	-0.97799
H	3.50588	17.82516	0.20069
H	5.00807	18.22734	1.03200

H	4.02316	19.51735	-1.55264
H	5.52640	19.91773	-0.72285
H	8.70114	5.85302	-10.72991
H	8.88382	4.88325	-9.27040
H	10.25075	4.33168	-11.94580
H	8.81503	3.51730	-11.32428
H	11.63236	3.41068	-10.17970
H	10.22447	2.78756	-9.31688
H	10.96402	1.87007	-12.12337
H	11.62191	1.11190	-10.67480
H	8.64521	1.28229	-11.34153
H	9.33748	0.53595	-9.90262
H	9.66460	-0.14714	-11.49540
H	4.57577	9.70359	6.49912
H	4.63843	9.15933	8.17440
H	2.13002	10.10702	6.71240
H	2.17569	9.53857	8.38105
H	3.70209	11.95208	7.30453
H	3.73163	11.38581	8.97402
H	1.25396	12.35859	7.48160
H	1.27575	11.78085	9.14774
H	2.80546	14.21485	8.10660
H	2.82325	13.63713	9.77332
H	1.32878	14.26225	9.07253
H	4.22947	20.62241	1.28436
H	2.72646	20.22722	0.45072
H	3.23952	21.94246	-1.28971
H	4.74459	22.33645	-0.45830
H	3.19494	22.66229	0.32123
C	2.66137	-11.01145	4.14372
H	3.01716	-10.06208	3.74888
H	2.14878	-11.53790	3.34239
H	3.52284	-11.60416	4.44487
H	1.39184	-11.77367	5.69467
H	2.28680	-10.32401	6.15298
C	-4.22270	-8.25705	5.25230
H	-5.14723	-7.70853	5.08485
H	-3.87259	-8.04977	6.26142
H	-4.43263	-9.32175	5.17853
H	-3.54261	-8.03028	3.21868
H	-2.97461	-6.77335	4.32471
C	-7.74859	-7.81121	-6.27894
H	-8.26798	-7.58394	-7.20764
H	-8.42002	-7.60681	-5.44773
H	-7.50945	-8.87270	-6.27975
H	-6.76482	-5.90769	-6.16580
H	-5.97842	-7.16728	-5.21401
C	-1.54442	-8.23892	-10.38966
H	-0.90586	-8.18149	-11.26923
H	-1.57880	-9.27816	-10.07124
H	-1.09187	-7.64251	-9.60015
H	-2.81472	-6.73752	-11.22924
H	-3.39090	-8.38682	-11.46477
C	1.33521	9.12543	-9.08032
H	2.18329	8.88016	-8.44380
H	1.45542	8.60898	-10.03031
H	0.42570	8.76387	-8.60513
H	0.39721	10.84934	-9.94399
H	1.09468	11.13408	-8.34670
C	7.15683	11.14122	-12.06367
H	7.32166	10.36112	-11.32364
H	8.12305	11.46928	-12.44153
H	6.59311	10.71070	-12.88809
H	7.00929	12.77029	-10.65868
H	6.24189	13.07784	-12.22054

C	8.20509	6.92647	-1.60544
H	7.27364	6.80036	-2.15358
H	8.43468	5.99718	-1.08764
H	9.00180	7.12979	-2.31745
H	7.84113	8.99461	-1.14685
H	7.26592	7.86585	0.08654
C	14.24704	8.06853	1.69910
H	15.13825	8.19146	2.31109
H	14.38517	8.62315	0.77395
H	14.14441	7.01500	1.44769
H	12.92937	8.04176	3.40577
H	13.14989	9.63373	2.67335
H	5.58132	0.61401	7.73165
H	9.52608	-0.70945	6.71931
H	6.21810	2.94616	7.32821
H	12.52118	3.77134	4.05688
H	12.63333	5.69469	2.52973
H	8.93971	7.21452	4.05044
H	11.62516	8.27202	-2.49726
H	8.98713	11.27009	-0.97467
H	11.08403	8.91166	-4.82084
H	6.05438	13.99795	-5.56497
H	4.72528	13.88113	-7.63762
H	7.06270	10.58734	-9.03496
H	0.27254	11.81877	-11.96729
H	3.28923	8.89244	-12.72336
H	-1.43141	10.40590	-13.05289
H	0.81068	3.85753	-14.91447
H	-0.86648	2.07348	-14.67977
H	-3.93208	4.95623	-13.90984
H	8.74545	6.85292	1.65229
H	12.36376	9.78667	0.16141
H	3.32992	11.63764	-8.00630
H	4.26294	11.43720	-12.80296
H	8.31693	-2.16166	5.61148
H	5.58803	-1.38047	8.81826
H	7.31727	-4.36628	5.24079
H	1.42729	-5.84742	9.06832
H	-0.05189	-7.54866	8.08651
H	2.70776	-8.18577	4.88207
H	-4.12003	-10.59397	3.08634
H	-0.34272	-9.48722	1.41998
H	-5.11579	-10.71827	0.84335
H	-0.81464	-8.52320	-4.46377
H	-1.92811	-7.89377	-6.55825
H	-5.65371	-9.44056	-5.12698
H	-4.84969	-6.76627	-12.39517
H	-5.37701	-4.07322	-9.10946
H	-4.58150	-4.88324	-13.93530
H	-5.16681	1.62748	-10.95270
H	-4.06170	3.48476	-12.12273
H	-2.88497	0.88397	-15.30954
H	-1.22544	-7.22851	5.93458
H	-0.13537	-11.31940	3.49463
H	-2.85712	-8.94962	-8.35143
H	-6.62209	-5.87093	-8.54045
H	3.53021	6.65578	-12.26943
H	5.61909	5.48390	-12.78070
H	4.18008	4.60357	-16.69943
H	2.08689	5.78900	-16.19505
H	-2.84426	9.46448	-15.01310
H	-4.91122	10.72048	-14.57142
H	-6.10943	7.75236	-11.75100
H	-4.03994	6.50779	-12.17448
H	9.04472	8.00130	-5.85857
H	9.89506	6.10831	-7.17393
H	10.34454	8.66355	-10.56163
H	9.53466	10.56567	-9.23683
H	5.54793	12.62121	-3.60155
H	4.32054	14.16487	-2.13395
H	7.97180	15.23019	-0.20076
H	9.20260	13.70114	-1.67591
H	7.29215	5.17886	8.05807
H	5.21359	6.39454	8.55154
H	4.83926	7.42508	4.42838
H	6.89181	6.18484	3.92736
H	12.87713	2.63417	6.39698
H	14.92444	1.37747	5.87288
H	12.57184	-1.72537	4.12990
H	10.52957	-0.46267	4.62910
H	3.35894	-5.16643	10.58979
H	2.06407	-4.26758	12.47692
H	1.96044	-0.44562	10.58433
H	3.23032	-1.34622	8.68772
H	5.40312	-4.79332	3.75634
H	6.56276	-5.61498	1.74997
H	6.33066	-9.60295	3.24809
H	5.17658	-8.78403	5.25555
H	-1.64870	-1.12757	-15.22310
H	-0.89567	-2.13953	-17.32705
H	-4.62558	-4.17790	-17.69042
H	-5.39135	-3.14865	-15.59237
H	-7.31957	-2.46537	-9.19265
H	-7.89697	-1.55195	-6.98717
H	-4.55481	1.09078	-7.20293
H	-3.96232	0.16467	-9.40055
H	-0.35836	-7.27836	-2.48007
H	2.00274	-6.64137	-2.72731
H	3.17311	-10.14069	-0.58563
H	0.81671	-10.79564	-0.36391
H	-5.45767	-12.15679	-1.24603
H	-7.79004	-12.83504	-0.86102
H	-9.14171	-9.32370	-2.86985
H	-6.81739	-8.63117	-3.23422

*Table 10 Cartesian coordinates of **1i** (GFN2-xTB(GBSA(toluene)). B97-3c(COSMO(toluene) single point energy = 10838.555771 Hartree.*

	X	Y	Z
C	10.63174	-0.99624	5.76474
C	9.72164	-0.99446	6.82372
C	9.33883	0.23351	7.38662
C	9.79558	1.40872	6.82972
C	10.65563	1.38311	5.70046
C	11.10702	0.17886	5.20129
C	10.74411	2.74057	5.22051
C	9.91742	3.56588	6.09313
C	11.16087	3.45682	4.13736
C	9.87986	4.89010	5.50454
C	9.37106	2.79609	7.08040
C	9.26733	6.07620	5.85560
C	9.28330	7.13904	4.95664
C	9.93124	7.04296	3.73033
C	10.64081	5.87675	3.41785
C	10.60238	4.80644	4.28291
C	7.64758	11.53095	-0.39858
C	8.66642	10.60578	-0.59770
C	9.18240	10.41331	-1.88359

C	8.59880	11.06307	-2.94866
C	7.48506	11.92033	-2.74763
C	7.04938	12.19334	-1.46648
C	6.94969	12.20084	-4.06308
C	7.78551	11.49619	-5.02862
C	5.83015	12.68631	-4.67418
C	7.13900	11.61668	-6.31638
C	8.79281	10.83745	-4.38711
C	7.45140	11.15427	-7.57785
C	6.49619	11.25187	-8.58266
C	5.25280	11.83159	-8.35134
C	4.98628	12.41481	-7.10487
C	5.91068	12.29490	-6.08992
C	-0.26346	10.38009	-11.84004
C	1.09189	10.07377	-11.85413
C	1.55183	8.99717	-12.62157
C	0.63911	8.17292	-13.24336
C	-0.75310	8.44213	-13.14312
C	-1.19391	9.57415	-12.48891
C	-1.43219	7.30474	-13.72585
C	-0.40220	6.39947	-14.21846
C	-2.64887	6.68699	-13.74434
C	-1.05504	5.15922	-14.56752
C	0.83892	6.90295	-13.95396
C	-0.58736	3.93179	-14.98999
C	-1.46369	2.85534	-15.01797
C	-2.80807	2.98753	-14.66817
C	-3.30130	4.26103	-14.34095
C	-2.43046	5.32409	-14.25230
C	9.72779	8.04000	2.65917
C	9.13171	7.54535	1.50272
C	8.80915	8.34393	0.41986
C	9.08442	9.71396	0.50435
C	9.68717	10.21008	1.65405
C	10.02803	9.40096	2.73141
C	4.15044	11.62417	-9.31059
C	3.02861	10.97996	-8.79130
C	1.98808	10.52710	-9.58088
C	2.06855	10.73654	-10.96307
C	3.14646	11.44606	-11.47480
C	4.19244	11.89948	-10.67866
C	0.88462	9.73171	-8.93650
C	5.31748	12.67708	-11.30897
C	10.77778	9.99205	3.89347
C	8.14197	7.69705	-0.76570
C	9.63963	-3.49084	6.97420
C	9.03526	-2.24809	7.16525
C	7.67293	-2.20517	7.50197
C	6.93039	-3.36377	7.49532
C	7.54220	-4.60863	7.18414
C	8.90671	-4.67069	6.98415
C	6.47037	-5.56835	7.04449
C	5.22953	-4.86979	7.34955
C	6.20187	-6.79492	6.50697
C	4.14368	-5.75012	6.98064
C	5.48008	-3.56060	7.64462
C	2.77281	-5.59562	6.97043
C	1.99618	-6.55956	6.33967
C	2.56973	-7.67774	5.74106
C	3.95279	-7.87556	5.82173
C	4.73857	-6.91351	6.42146
C	-0.60618	-10.84673	0.76572
C	-0.86188	-9.91921	1.76871
C	-2.04198	-9.16632	1.72834
C	-2.86480	-9.25766	0.62770
C	-2.53716	-10.13329	-0.44321
C	-1.43919	-10.96284	-0.34334
C	-3.47962	-9.84879	-1.50512
C	-4.40494	-8.84544	-0.99502
C	-3.64287	-9.99329	-2.85259
C	-5.19496	-8.38247	-2.11278
C	-4.06161	-8.48314	0.27440
C	-6.15691	-7.40203	-2.23899
C	-6.57929	-7.04211	-3.51235
C	-6.07537	-7.66626	-4.65066
C	-5.15814	-8.71577	-4.51721
C	-4.70375	-9.05893	-3.26110
C	-7.32764	-3.15651	-8.84896
C	-6.66254	-4.32227	-9.22224
C	-6.03890	-4.38735	-10.47336
C	-5.98757	-3.25508	-11.25959
C	-6.54091	-2.03297	-10.79596
C	-7.27205	-2.00627	-9.62548
C	-6.07583	-1.00650	-11.70090
C	-5.99604	0.34727	-11.83949
C	-4.74632	-0.62965	-13.58770
C	-3.93037	-0.61748	-14.70261
C	-3.38473	0.58811	-15.12758
C	-3.62081	1.78452	-14.44850
C	-4.55023	1.78521	-13.39758
C	-5.12332	0.60272	-12.99215
C	1.74801	-8.49887	4.82865
C	2.12410	-8.46349	3.48868
C	1.33984	-8.98484	2.47453
C	0.11796	-9.57495	2.82053
C	-0.22172	-9.67997	4.16428
C	0.57121	-9.16135	5.18008
C	-6.32691	-7.05002	-5.96780
C	-5.19479	-6.61550	-6.65410
C	-5.25440	-5.79152	-7.76423
C	-6.52258	-5.38947	-8.21212
C	-7.65367	-5.89944	-7.58400
C	-7.58740	-6.73288	-6.47601
C	-3.98686	-5.29468	-8.41529
C	-8.85534	-7.30385	-5.90219
C	0.17325	-9.37051	6.61512
C	1.77218	-8.76482	1.04950
C	2.12009	6.29697	-14.27679
C	-3.91656	7.20876	-13.25868
C	-6.59426	1.40366	-11.03891
C	-4.56020	-4.05955	-13.24490
C	-2.87723	-10.83676	-3.75699
C	-4.71905	-7.49915	1.11935
C	7.16257	-7.79039	6.05980
C	4.51909	-2.54809	8.04789
C	11.97073	2.99580	3.02169
C	8.47985	3.19410	8.15819
C	4.74099	13.42922	-4.06176
C	9.82623	9.96660	-4.92456
C	-5.24891	-3.01266	-12.50817
C	-5.29921	-1.66911	-12.74605
C	3.14436	6.18785	-13.33719
C	4.34628	5.59622	-13.67543
C	4.56987	5.10728	-14.95704
C	3.54595	5.21338	-15.89282
C	2.33855	5.79272	-15.56031
C	5.86351	4.43621	-15.31456
C	-4.36236	8.46955	-13.65450
C	-5.55160	8.97842	-13.17048
C	-6.32246	8.25660	-12.26624

C	-5.87422	7.00152	-11.86907
C	-4.69414	6.47930	-12.35914
C	-7.62369	8.79923	-11.75167
C	3.40643	13.09185	-4.28300
C	2.39138	13.81434	-3.68636
C	2.67154	14.89858	-2.86308
C	4.00356	15.22923	-2.63605
C	5.02379	14.50577	-3.21948
C	9.51090	8.96542	-5.84268
C	10.49030	8.11674	-6.31865
C	11.81104	8.24159	-5.90092
C	12.12485	9.24476	-4.99097
C	11.14922	10.09057	-4.50064
C	1.56849	15.66468	-2.19357
C	13.14777	2.28396	3.25535
C	13.92342	1.84333	2.20146
C	13.54947	2.09151	0.88522
C	12.36908	2.78869	0.65346
C	11.59151	3.23907	1.70190
C	14.36968	1.57421	-0.25994
C	7.39083	4.03072	7.91427
C	6.56675	4.43036	8.94751
C	6.80312	4.01422	10.25313
C	7.87667	3.16297	10.49150
C	8.70030	2.75078	9.46261
C	5.96481	4.52591	11.38785
C	4.52085	-1.25783	7.51974
C	3.60605	-0.31816	7.95677
C	2.65768	-0.63434	8.92153
C	2.64291	-1.92857	9.43338
C	3.55565	-2.87031	9.00721
C	8.23002	-8.14527	6.88570
C	9.15672	-9.08219	6.47451
C	9.05887	-9.68368	5.22450
C	7.99971	-9.32330	4.39911
C	7.06200	-8.39518	4.80738
C	10.04792	-10.72615	4.79263
C	1.68950	0.39186	9.43312
C	-6.53319	1.38061	-9.64609
C	-7.10174	2.39583	-8.90244
C	-7.75206	3.45869	-9.52030
C	-7.81058	3.48047	-10.90901
C	-7.23315	2.47666	-11.66095
C	-3.24561	-3.88656	-13.67683
C	-2.58733	-4.89999	-14.34540
C	-3.21959	-6.10889	-14.61412
C	-4.53396	-6.27612	-14.19112
C	-5.19538	-5.27317	-13.51117
C	-2.48982	-7.23117	-15.29234
C	-8.41583	4.53039	-8.70640
C	-6.10604	-7.53794	1.27450
C	-6.75128	-6.61267	2.06884
C	-6.04066	-5.60932	2.72078
C	-4.66108	-5.56782	2.55990
C	-4.00517	-6.49873	1.77672
C	-2.67270	-12.18522	-3.46596
C	-1.93650	-12.98478	-4.31854
C	-1.36760	-12.46348	-5.47497
C	-1.56421	-11.11628	-5.75868
C	-2.31032	-10.31384	-4.91910
C	-0.58982	-13.33293	-6.41915
C	-6.76179	-4.58494	3.54841
C	2.10926	0.89554	10.82008
C	1.12247	1.91794	11.37897
C	1.46841	2.32416	12.81005
C	0.50705	3.37556	13.36077
C	0.83351	3.74510	14.80329
C	-7.51951	-3.57745	2.67227
C	-6.58328	-2.74212	1.80225
C	-7.34814	-1.74229	0.93748
C	-6.41732	-0.90395	0.06336
C	-7.18675	0.09100	-0.79835
C	9.55236	-12.13063	5.16170
C	10.54408	-13.21302	4.74176
C	10.03861	-14.61116	5.09378
C	11.05100	-15.71815	4.79362
C	11.31010	-15.91319	3.30341
C	-8.80407	8.25763	-12.56821
C	-10.14348	8.76228	-12.03693
C	-11.32278	8.21892	-12.84119
C	-12.66312	8.72365	-12.31005
C	-13.83697	8.18316	-13.11884
C	5.75556	2.91622	-15.13480
C	7.04845	2.20418	-15.52526
C	6.94383	0.68975	-15.35699
C	8.23057	-0.02524	-15.76505
C	8.12028	-1.53672	-15.59956
C	-9.85851	4.13557	-8.36444
C	-10.55888	5.20568	-7.53040
C	-11.99925	4.81620	-7.20249
C	-12.70722	5.81904	-6.28934
C	-12.97523	7.16246	-6.95962
C	-1.87743	-8.17191	-14.24529
C	-1.06267	-9.31502	-14.85235
C	-1.91928	-10.34124	-15.59273
C	-1.10857	-11.51369	-16.15005
C	-0.56400	-12.44299	-15.07019
C	6.62169	5.77447	11.99328
C	5.86847	6.34219	13.19671
C	4.53112	6.98600	12.83369
C	3.90002	7.69635	14.03010
C	2.55972	8.33140	13.67708
C	-1.48370	-13.83590	-7.56035
C	-0.70120	-14.67225	-8.57012
C	-1.58895	-15.18369	-9.70304
C	-0.80277	-15.99885	-10.72835
C	-1.69901	-16.53343	-11.83981
C	12.87784	7.34081	-6.45100
C	13.45257	7.91212	-7.75397
C	14.53833	7.00841	-8.33435
C	15.20723	7.58650	-9.58272
C	1.29199	15.10512	-0.79189
C	0.17832	15.87038	-0.08111
C	-0.08986	15.32272	1.31931
C	-1.20307	16.08637	2.03418
C	-1.46114	15.54006	3.43396
C	13.88136	0.18495	-0.69122
C	14.69028	-0.36601	-1.86306
C	14.19723	-1.74344	-2.30194
C	15.00350	-2.29847	-3.47475
C	14.50454	-3.67194	-3.90961
C	14.28485	7.64610	-10.79940
C	15.02382	8.13039	-12.04214
H	6.66780	4.81481	-14.68043
H	6.11759	4.65284	-16.35470
H	-7.62115	9.88975	-11.80770
H	-7.75343	8.51331	-10.70522
H	0.65594	15.60118	-2.79010
H	1.84762	16.71724	-2.10724
H	14.29375	2.25715	-1.10901

H	15.41960	1.50842	0.03334
H	4.96634	4.77527	11.02702
H	5.87551	3.76029	12.16168
H	11.01027	-10.54888	5.27775
H	10.19525	-10.67310	3.71154
H	1.63952	1.23597	8.74251
H	0.69196	-0.04908	9.50549
H	-1.69293	-6.83234	-15.92351
H	-3.18393	-7.78837	-15.92310
H	-8.42942	5.46832	-9.26517
H	-7.86075	4.69218	-7.77959
H	-0.17642	-14.19004	-5.88358
H	0.24017	-12.76379	-6.84459
H	-6.04847	-4.04773	4.17659
H	-7.47991	-5.08800	4.20121
H	3.10425	1.34276	10.75357
H	2.17555	0.04542	11.50291
H	1.11794	2.80452	10.73988
H	0.11486	1.49448	11.36143
H	1.43920	1.43971	13.45159
H	2.48744	2.71687	12.83874
H	-0.51538	2.99324	13.30776
H	0.55578	4.27026	12.73534
H	0.73706	2.87589	15.45109
H	0.15865	4.51855	15.16384
H	1.85360	4.11496	14.88069
H	-8.09898	-2.91399	3.31891
H	-8.22159	-4.11476	2.03082
H	-6.00103	-3.40561	1.15798
H	-5.88066	-2.20170	2.44177
H	-7.93126	-1.07861	1.58127
H	-8.05057	-2.28281	0.29784
H	-5.71436	-0.36252	0.70135
H	-5.83464	-1.56731	-0.58086
H	-7.75920	0.77408	-0.17379
H	-7.87789	-0.43031	-1.45784
H	-6.50344	0.67588	-1.41061
H	8.58964	-12.30929	4.67661
H	9.39049	-12.18034	6.24142
H	10.71197	-13.14149	3.66569
H	11.50072	-13.03994	5.24140
H	9.11468	-14.81020	4.54489
H	9.79894	-14.63795	6.16033
H	10.67196	-16.65531	5.20954
H	11.99318	-15.49137	5.29875
H	10.37650	-16.10051	2.77641
H	11.96989	-16.76369	3.14403
H	11.78107	-15.03470	2.86975
H	-8.68642	8.56015	-13.61164
H	-8.78806	7.16569	-12.54070
H	-10.15702	9.85464	-12.07022
H	-10.25339	8.46427	-10.99109
H	-11.21462	8.51716	-13.88714
H	-11.31085	7.12641	-12.80797
H	-12.67474	9.81601	-12.34089
H	-12.77372	8.42228	-11.26548
H	-13.75748	8.49099	-14.15969
H	-14.77905	8.55546	-12.72203
H	-13.85576	7.09551	-13.08528
H	5.51496	2.69481	-14.09206
H	4.93317	2.54093	-15.74856
H	7.86912	2.57990	-14.90903
H	7.28630	2.43453	-16.56693
H	6.71709	0.45674	-14.31350
H	6.11630	0.31565	-15.96507
H	9.05826	0.34641	-15.15599
H	8.45715	0.20881	-16.80823
H	7.91625	-1.79353	-14.56190
H	9.04657	-2.02317	-15.89789
H	7.31254	-1.92994	-16.21375
H	-9.85037	3.19022	-7.81633
H	-10.41417	3.97321	-9.29140
H	-10.54850	6.14771	-8.08116
H	-10.00647	5.35724	-6.59936
H	-12.56652	4.71266	-8.13093
H	-11.99363	3.83975	-6.71050
H	-12.10507	5.97543	-5.39082
H	-13.66118	5.38779	-5.97484
H	-13.54331	7.80886	-6.29364
H	-13.54984	7.02299	-7.87328
H	-12.04602	7.66843	-7.20906
H	-1.22747	-7.58295	-13.59246
H	-2.67665	-8.58537	-13.62531
H	-0.52823	-9.81438	-14.04195
H	-0.31717	-8.90470	-15.53820
H	-2.68640	-10.72641	-14.91600
H	-2.42697	-9.85104	-16.42600
H	-1.75253	-12.09359	-16.81654
H	-0.28006	-11.12670	-16.74837
H	-0.07062	-13.29993	-15.52442
H	0.15973	-11.93258	-14.43976
H	-1.37310	-12.80763	-14.44051
H	7.63658	5.51257	12.30371
H	6.70435	6.54356	11.22131
H	5.70348	5.54861	13.92983
H	6.50046	7.09881	13.66980
H	4.68719	7.71019	12.02984
H	3.83903	6.22571	12.46700
H	4.58235	8.47081	14.38995
H	3.75954	6.98000	14.84316
H	2.13183	8.82896	14.54480
H	1.85595	7.57600	13.33395
H	2.68317	9.06801	12.88542
H	-1.93244	-12.97861	-8.06791
H	-2.29745	-14.43313	-7.14174
H	0.10638	-14.06734	-8.99062
H	-0.24354	-15.52282	-8.05855
H	-2.06031	-14.33429	-10.20415
H	-2.38656	-15.80312	-9.28490
H	-0.02054	-15.37199	-11.16378
H	-0.31298	-16.83581	-10.22434
H	-2.19068	-15.71455	-12.36103
H	-2.46704	-17.18676	-11.43023
H	-1.11612	-17.09994	-12.56287
H	13.68388	7.23146	-5.72208
H	12.46120	6.35088	-6.65045
H	13.86967	8.90271	-7.55672
H	12.64059	8.03062	-8.47321
H	14.10722	6.03342	-8.57548
H	15.30496	6.84935	-7.57113
H	16.07165	6.96519	-9.83239
H	15.57745	8.59072	-9.36126
H	1.01606	14.05102	-0.87534
H	2.20780	15.15897	-0.19835
H	-0.73791	15.80975	-0.67393
H	0.45429	16.92550	-0.00836
H	-0.36523	14.26740	1.24772
H	0.82635	15.38402	1.91219
H	-2.12111	16.02128	1.44490
H	-0.92914	17.14214	2.10251

H	-1.75868	14.49428	3.38681
H	-2.25417	16.10104	3.92393
H	-0.56236	15.61213	4.04338
H	13.95430	-0.49946	0.15753
H	12.82732	0.24786	-0.97277
H	14.62257	0.32620	-2.70620
H	15.74274	-0.43412	-1.57607
H	13.14473	-1.67517	-2.58897
H	14.26385	-2.43611	-1.45909
H	16.05548	-2.36976	-3.18727
H	14.93766	-1.60565	-4.31735
H	14.57693	-4.38167	-3.08776
H	15.09565	-4.04858	-4.74171
H	13.46384	-3.61822	-4.22364
H	13.87365	6.65117	-10.98803
H	13.45029	8.31993	-10.59788
H	15.84904	7.46254	-12.28185
H	15.42776	9.12797	-11.88059
H	14.35232	8.16681	-12.89750
C	0.61349	-10.75765	7.09057
H	1.69566	-10.85177	7.02613
H	0.16423	-11.53354	6.47407
H	0.31298	-10.91645	8.12408
H	-0.91176	-9.28572	6.71312
H	0.63821	-8.61655	7.25083
C	1.14412	-7.48690	0.48857
H	1.49258	-7.30738	-0.52637
H	1.40856	-6.63005	1.10537
H	0.06026	-7.57774	0.46909
H	1.48418	-9.60358	0.41749
H	2.86015	-8.67188	1.01736
C	-9.36199	-8.45574	-6.77428
H	-9.54833	-8.11451	-7.79038
H	-10.28685	-8.85940	-6.36752
H	-8.62251	-9.25331	-6.81085
H	-9.62224	-6.52645	-5.85561
H	-8.68106	-7.67790	-4.89325
C	-2.79542	-5.14494	-7.47077
H	-1.99147	-4.61971	-7.98297
H	-2.41038	-6.10993	-7.15065
H	-3.07473	-4.57061	-6.58964
H	-4.18545	-4.32395	-8.87449
H	-3.71012	-5.98203	-9.22187
C	1.19604	8.23416	-8.98877
H	2.14625	8.02516	-8.50096
H	1.25583	7.89912	-10.02199
H	0.41419	7.66668	-8.48835
H	-0.06679	9.90986	-9.43670
H	0.78429	10.03992	-7.89326
C	6.12594	11.83990	-12.30216
H	6.55133	10.96509	-11.81503
H	6.93894	12.43222	-12.71684
H	5.49781	11.49678	-13.12089
H	5.97935	13.06034	-10.53050
H	4.89408	13.53476	-11.84055
C	9.11915	6.83264	-1.56586
H	8.61308	6.38422	-2.41837
H	9.52405	6.03728	-0.94451
H	9.94982	7.42843	-1.93670
H	7.70733	8.45300	-1.42046
H	7.32928	7.06044	-0.40377
C	12.27971	10.01807	3.59630
H	12.82282	10.46180	4.42816
H	12.48143	10.59985	2.69905
H	12.65025	9.00672	3.44061

H	10.60908	9.40384	4.79531
H	10.42990	11.01187	4.07467
H	10.91999	-1.93577	5.31761
H	8.65459	0.25831	8.22219
H	11.76554	0.14533	4.34578
H	8.74817	6.17464	6.79753
H	8.73378	8.03888	5.19560
H	11.18378	5.82104	2.48547
H	7.26848	11.68431	0.60211
H	9.99024	9.71356	-2.03743
H	6.21557	12.85819	-1.29446
H	8.40217	10.68148	-7.77558
H	6.70059	10.81209	-9.54690
H	4.04409	12.91887	-6.94617
H	-0.60244	11.24974	-11.29404
H	2.61201	8.79367	-12.66334
H	-2.24701	9.80704	-12.43351
H	0.45490	3.79111	-15.23613
H	-1.07501	1.87785	-15.26183
H	-4.35090	4.39742	-14.12728
H	8.88253	6.49333	1.46453
H	9.90334	11.26904	1.70707
H	2.99974	10.77581	-7.72944
H	3.17607	11.63789	-12.53893
H	10.70279	-3.54136	6.78947
H	7.19854	-1.25556	7.69215
H	9.39502	-5.61393	6.78769
H	2.31097	-4.71564	7.39332
H	0.92853	-6.41021	6.25876
H	4.38920	-8.75855	5.37762
H	0.26766	-11.47912	0.84038
H	-2.25897	-8.48102	2.53483
H	-1.19886	-11.66096	-1.13167
H	-6.53492	-6.88168	-1.37126
H	-7.27071	-6.21937	-3.62760
H	-4.78950	-9.21610	-5.40112
H	-7.83742	-3.12941	-7.89618
H	-5.56719	-5.30490	-10.79230
H	-7.75407	-1.09954	-9.29128
H	-3.68773	-1.53293	-15.22232
H	-2.73360	0.58798	-15.98976
H	-4.75942	2.69478	-12.85560
H	3.04082	-7.95190	3.22692
H	-1.15510	-10.16194	4.42428
H	-4.23288	-6.89166	-6.25135
H	-8.62561	-5.61263	-7.96335
H	2.98351	6.53685	-12.32770
H	5.12590	5.51350	-12.93086
H	3.70069	4.83892	-16.89524
H	1.55508	5.88170	-16.29952
H	-3.77300	9.03918	-14.35903
H	-5.88411	9.95568	-13.49246
H	-6.45159	6.42814	-11.15774
H	-4.34524	5.51774	-12.01128
H	3.16789	12.23734	-4.89886
H	1.36229	13.53389	-3.86421
H	4.24154	16.06757	-1.99611
H	6.05472	14.78133	-3.04775
H	8.48433	8.84180	-6.15574
H	10.22595	7.33794	-7.02066
H	13.14679	9.35887	-4.65663
H	11.41123	10.87040	-3.79970
H	13.45738	2.09929	4.27422
H	14.83665	1.30026	2.40195
H	12.05683	2.98186	-0.36357

H	10.66313	3.75365	1.50284
H	7.18706	4.34760	6.90179
H	5.72758	5.07983	8.73928
H	8.07155	2.82279	11.49919
H	9.54372	2.10759	9.66973
H	5.21927	-0.99977	6.73729
H	3.62747	0.67758	7.53573
H	1.90529	-2.19828	10.17640
H	3.54810	-3.86534	9.42883
H	8.31248	-7.68998	7.86248
H	9.97346	-9.34912	7.13090
H	7.91268	-9.77255	3.41947
H	6.26500	-8.10533	4.13863
H	-6.01231	0.57183	-9.15439
H	-7.03749	2.36680	-7.82346
H	-8.31284	4.29695	-11.40874
H	-7.29788	2.50297	-12.73940
H	-2.73667	-2.95941	-13.45676
H	-1.56424	-4.75198	-14.66279
H	-5.04419	-7.20664	-14.39806
H	-6.22165	-5.41384	-13.20338
H	-6.66796	-8.31813	0.78071
H	-7.82453	-6.66753	2.18780
H	-4.09040	-4.79271	3.05228
H	-2.93527	-6.42964	1.64415
H	-3.11404	-12.60398	-2.57257
H	-1.79501	-14.02994	-4.08013
H	-1.11974	-10.69002	-6.64741
H	-2.42658	-9.26282	-5.13916

*Table 11 Cartesian coordinates of **1k** (GFN2-xTB(GBSA(toluene)). B97-3c(COSMO(toluene) single point energy = 10838.5490907Hartree.*

	X	Y	Z
C	8.95078	-0.15874	6.40844
C	7.97120	0.15303	7.35396
C	7.84087	1.48838	7.77301
C	8.58673	2.47218	7.16018
C	9.55560	2.13156	6.17858
C	9.76017	0.81158	5.83684
C	10.04681	3.37185	5.63122
C	9.35257	4.45110	6.31827
C	10.74816	3.85365	4.56416
C	9.65192	5.66951	5.59524
C	8.49869	3.93949	7.25404
C	9.20717	6.97124	5.70745
C	9.51276	7.87603	4.69567
C	10.28425	7.50804	3.59836
C	10.81523	6.21427	3.53213
C	10.48134	5.29586	4.50218
C	10.57804	9.01496	-2.50391
C	10.11385	9.93098	-1.56368
C	9.47632	11.09875	-1.99465
C	9.19765	11.25298	-3.33727
C	9.53996	10.23465	-4.26531
C	10.29897	9.15661	-3.85754
C	8.87304	10.57063	-5.50359
C	8.19630	11.84698	-5.29735
C	8.54594	10.05371	-6.72264
C	7.43754	12.12456	-6.49958
C	8.38922	12.27645	-4.01642
C	6.57712	13.13783	-6.87244
C	5.80978	12.97841	-8.02173
C	5.90275	11.83311	-8.80541
C	6.84907	10.85370	-8.48544
C	7.61780	11.00299	-7.35185
C	0.54314	10.45645	-12.55061
C	1.80871	9.92652	-12.32386
C	2.11533	8.64614	-12.80195
C	1.11956	7.87566	-13.36177
C	-0.20353	8.38472	-13.46833
C	-0.47109	9.69213	-13.11801
C	-1.03608	7.27432	-13.87740
C	-0.15303	6.13639	-14.09502
C	-2.33857	6.86651	-13.87570
C	-0.99134	4.97198	-14.26585
C	1.13786	6.47587	-13.80922
C	-0.71730	3.63230	-14.44935
C	-1.75484	2.71604	-14.33355
C	-3.05657	3.12069	-14.04592
C	-3.35334	4.48972	-13.98006
C	-2.33062	5.40821	-14.07273
C	10.35237	8.35560	2.39146
C	9.12577	8.68767	1.82109
C	9.01312	9.26757	0.56996
C	10.18895	9.54471	-0.13867
C	11.41431	9.29916	0.46836
C	11.52505	8.70601	1.72008
C	4.88156	11.52788	-9.82944
C	3.58241	11.37008	-9.35489
C	2.54698	10.90034	-10.14403
C	2.81958	10.60481	-11.48639
C	4.10070	10.82853	-11.97702
C	5.14417	11.27426	-11.17514
C	1.22849	10.59969	-9.48251
C	6.51460	11.46318	-11.76890
C	12.88797	8.47003	2.31605
C	7.63879	9.44080	-0.01930
C	5.67738	-0.51127	8.04239
C	6.99059	-0.87373	7.73455
C	7.29073	-2.23898	7.59686
C	6.27768	-3.16988	7.63827
C	4.93435	-2.76993	7.86249
C	4.65152	-1.44380	8.12061
C	4.11199	-3.92110	7.57181
C	5.00585	-4.99946	7.16479
C	2.81458	-4.25458	7.30868
C	4.17275	-6.06771	6.65670
C	6.30354	-4.58328	7.23172
C	4.44630	-7.30210	6.10442
C	3.42273	-7.99542	5.46809
C	2.12543	-7.49504	5.41547
C	1.82053	-6.31248	6.10544
C	2.83428	-5.58959	6.69459
C	-0.16605	-9.14356	-0.15378
C	-1.01273	-9.18187	0.95127
C	-2.37315	-9.44877	0.78464
C	-2.87535	-9.57705	-0.49757
C	-2.00594	-9.47962	-1.61576
C	-0.64964	-9.30052	-1.44419
C	-2.84015	-9.50721	-2.79272
C	-4.21524	-9.68898	-2.34688
C	-2.80129	-9.20011	-4.11948
C	-5.05951	-9.53079	-3.51502
C	-4.25721	-9.73436	-0.98186
C	-6.41761	-9.60601	-3.74696
C	-6.91915	-9.25317	-4.99892
C	-6.08853	-8.80344	-6.02157

C	-4.70022	-8.81750	-5.80951	C	6.87125	15.73126	-2.04092
C	-4.19302	-9.17680	-4.58480	C	8.21179	15.37980	-1.93255
C	-6.54895	-5.73982	-12.00026	C	8.70652	14.25803	-2.56873
C	-6.70933	-5.55040	-10.62937	C	9.89435	4.96585	-9.06054
C	-6.72640	-4.23678	-10.13399	C	12.50694	2.18068	4.16653
C	-6.52447	-3.17749	-10.98585	C	13.30665	1.43583	3.32480
C	-6.20750	-3.39659	-12.35338	C	13.20545	1.56397	1.94302
C	-6.27164	-4.67875	-12.86019	C	12.27931	2.46390	1.42978
C	-5.79807	-2.11003	-12.88085	C	11.48166	3.22217	2.26605
C	-5.11894	-1.53804	-13.91918	C	14.04219	0.70631	1.03943
C	-5.46306	0.12504	-12.29037	C	8.11962	5.77496	8.85628
C	-5.44075	1.39479	-11.75288	C	7.30252	6.49111	9.70763
C	-4.72117	2.38003	-12.41145	C	5.96487	6.14756	9.87207
C	-4.02897	2.11489	-13.59277	C	5.46692	5.06752	9.15207
C	-4.15096	0.85607	-14.19673	C	6.27984	4.34037	8.30468
C	-4.86594	-0.13383	-13.55350	C	5.06828	6.95522	10.76395
C	1.14777	-8.03529	4.45126	C	7.58844	-5.94753	5.61876
C	0.61024	-7.09187	3.57462	C	8.75983	-6.55769	5.21718
C	-0.15528	-7.43857	2.47932	C	9.89505	-6.52651	6.01998
C	-0.40381	-8.79629	2.24652	C	9.82296	-5.86668	7.24176
C	0.04555	-9.73072	3.16642	C	8.65811	-5.24409	7.64651
C	0.81781	-9.38009	4.27047	C	1.47203	-2.78974	8.76677
C	-6.53641	-8.19165	-7.28629	C	0.36544	-1.99840	9.00377
C	-5.79352	-8.46499	-8.43763	C	-0.60762	-1.81824	8.02714
C	-5.85495	-7.70648	-9.59413	C	-0.43663	-2.45291	6.80122
C	-6.75331	-6.62548	-9.62171	C	0.66220	-3.25299	6.55928
C	-7.60268	-6.45457	-8.53041	C	-1.82512	-0.97832	8.28409
C	-7.51284	-7.19085	-7.35923	C	11.18325	-7.13566	5.54896
C	-4.92031	-7.99332	-10.74297	C	-3.37783	-2.05076	-15.61402
C	-8.43010	-6.81492	-6.22744	C	-2.97948	-2.68177	-16.77656
C	1.25599	-10.45512	5.22979	C	-3.87376	-3.44047	-17.52239
C	-0.71635	-6.36060	1.59279	C	-5.18183	-3.56020	-17.06407
C	2.31479	5.62958	-13.92304	C	-5.58428	-2.94444	-15.89621
C	-3.52326	7.68682	-13.67898	C	-7.81730	-1.53295	-8.64991
C	-4.68782	-2.17524	-15.15325	C	-8.06882	-0.98684	-7.40668
C	-6.67027	-1.17917	-9.36107	C	-7.18253	-0.08544	-6.82768
C	-1.63522	-8.95120	-4.95194	C	-6.03447	0.25822	-7.53339
C	-5.43815	-9.86496	-0.14326	C	-5.77958	-0.27626	-8.78065
C	1.63548	-3.43716	7.54146	C	-7.48284	0.54032	-5.49716
C	7.52083	-5.27681	6.83979	C	-3.43013	-4.15527	-18.76518
C	11.58277	3.09138	3.65030	C	-6.38101	-10.85744	-0.41534
C	7.62288	4.68107	8.14581	C	-7.51967	-10.97507	0.35481
C	8.92292	8.77964	-7.31275	C	-7.76564	-10.09916	1.40816
C	7.87124	13.46245	-3.35266	C	-6.82360	-9.11446	1.68055
C	-6.40677	-1.74926	-10.67232	C	-5.67297	-8.99978	0.92381
C	-5.97307	-1.13554	-11.80956	C	-0.56043	-9.84101	-4.92280
C	3.25657	5.51949	-12.90152	C	0.54146	-9.64003	-5.72969
C	4.36112	4.70196	-13.05070	C	0.61317	-8.54281	-6.58152
C	4.56989	3.98402	-14.22192	C	-0.44964	-7.64692	-6.59537
C	3.62707	4.09206	-15.23953	C	-1.55954	-7.84453	-5.79683
C	2.51448	4.89439	-15.09344	C	1.78557	-8.36050	-7.50016
C	5.75297	3.07395	-14.37884	C	-9.04016	-10.19914	2.19586
C	-3.67948	8.86759	-14.40624	C	12.00599	-6.09327	4.77852
C	-4.80012	9.65573	-14.23644	C	13.27323	-6.66259	4.13956
C	-5.79276	9.30158	-13.32908	C	14.32712	-7.09417	5.15793
C	-5.62951	8.13241	-12.59493	C	15.60978	-7.61941	4.50858
C	-4.51608	7.33333	-12.76606	C	16.41668	-6.53937	3.79512
C	-7.03129	10.13543	-13.17954	C	-10.24895	-9.73423	1.37097
C	8.87319	7.60877	-6.55537	C	-10.15033	-8.26327	0.97295
C	9.20386	6.39395	-7.11918	C	-11.32780	-7.82520	0.10444
C	9.59221	6.30427	-8.45246	C	-11.22495	-6.35804	-0.30828
C	9.64421	7.47122	-9.20506	C	-12.38936	-5.93192	-1.19560
C	9.31307	8.69223	-8.64863	C	-3.07325	-1.83888	8.52906
C	6.52705	13.81622	-3.46635	C	-2.95697	-2.67071	9.80448
C	6.03818	14.93183	-2.81660	C	-4.15288	-3.59572	10.03686

C	-5.44467	-2.85408	10.37808
C	-6.57825	-3.81981	10.70597
C	-8.11491	9.67004	-14.16127
C	-9.39912	10.48447	-14.02513
C	-10.47517	10.02330	-15.00628
C	-11.76811	10.82429	-14.86442
C	-12.83632	10.36059	-15.84844
C	5.36514	1.62171	-14.07135
C	6.52986	0.65876	-14.28934
C	6.13740	-0.78779	-13.99544
C	7.29890	-1.75713	-14.20659
C	6.90459	-3.19461	-13.88659
C	-3.06137	-5.61138	-18.45134
C	-2.57695	-6.35800	-19.69203
C	-2.24989	-7.81935	-19.39058
C	-1.74555	-8.56446	-20.62511
C	-1.43892	-10.02700	-20.32272
C	-8.29842	1.82724	-5.68007
C	-8.60789	2.50487	-4.34731
C	-9.43379	3.77661	-4.53139
C	-9.73379	4.46935	-3.20339
C	-10.56767	5.73126	-3.39516
C	4.41536	8.10247	9.98156
C	3.49147	8.93922	10.86317
C	2.83686	10.08088	10.08797
C	1.90979	10.91739	10.96784
C	1.26099	12.05685	10.19007
C	1.55084	-9.10478	-8.82159
C	2.72336	-8.94400	-9.78614
C	2.49201	-9.69490	-11.09605
C	3.66112	-9.53777	-12.06666
C	3.42438	-10.29367	-13.36940
C	6.34080	16.96430	-1.37005
C	6.36506	18.16166	-2.32905
C	5.83129	19.42954	-1.66675
C	5.82723	20.62074	-2.62258
C	8.58934	4.23038	-9.39399
C	8.80329	2.86340	-10.04460
C	9.51227	1.86314	-9.13138
C	9.50859	0.43721	-9.68890
C	8.15611	-0.25443	-9.55352
C	13.39891	-0.67503	0.85750
C	14.26430	-1.60133	0.00641
C	13.62511	-2.97693	-0.17400
C	14.50606	-3.91934	-0.99215
C	13.86020	-5.28780	-1.17878
C	5.29924	21.89029	-1.95699
C	5.28572	23.07449	-2.91694
H	6.55505	3.38273	-13.70555
H	6.12708	3.13038	-15.40390
H	-6.80030	11.18503	-13.37399
H	-7.41474	10.05274	-12.16018
H	10.47526	4.37096	-8.35399
H	10.48144	5.09041	-9.97265
H	14.15066	1.18348	0.06341
H	15.03805	0.57865	1.47019
H	5.64528	7.37223	11.59217
H	4.28516	6.31755	11.17974
H	-1.65703	-0.33875	9.15296
H	-2.00921	-0.33612	7.41883
H	10.97536	-7.98193	4.89056
H	11.75492	-7.49547	6.40534
H	-6.55138	0.77878	-4.97910
H	-8.05021	-0.15679	-4.87669
H	-2.56142	-3.65104	-19.19364
H	-4.23194	-4.14364	-19.50705
H	2.69224	-8.74698	-7.02935
H	1.93447	-7.29892	-7.70925
H	-8.96580	-9.59046	3.09926
H	-9.20151	-11.23756	2.49709
H	11.37532	-5.67187	3.99084
H	12.27369	-5.27788	5.45493
H	13.69805	-5.89198	3.49391
H	13.00919	-7.51598	3.50965
H	13.91517	-7.88490	5.78828
H	14.57304	-6.24724	5.80368
H	15.35602	-8.41103	3.79906
H	16.23465	-8.06440	5.28749
H	15.87033	-6.13580	2.94636
H	17.35386	-6.95183	3.42687
H	16.64624	-5.72303	4.47741
H	-11.15865	-9.89167	1.95556
H	-10.32365	-10.34451	0.46811
H	-9.21952	-8.10071	0.42380
H	-10.11544	-7.64506	1.87368
H	-12.26132	-7.98171	0.65125
H	-11.36296	-8.44796	-0.79328
H	-11.20368	-5.73206	0.58738
H	-10.28471	-6.20075	-0.84299
H	-13.33554	-6.06526	-0.67476
H	-12.41355	-6.52856	-2.10553
H	-12.29541	-4.88446	-1.47442
H	-3.22281	-2.50623	7.67728
H	-3.93945	-1.17847	8.59617
H	-2.05216	-3.28078	9.74131
H	-2.84719	-2.00350	10.66327
H	-3.91273	-4.27185	10.86189
H	-4.31226	-4.20944	9.14662
H	-5.26760	-2.20024	11.23577
H	-5.74503	-2.22678	9.53690
H	-6.78074	-4.47179	9.85843
H	-6.31621	-4.44181	11.55983
H	-7.48915	-3.27490	10.94517
H	-7.73360	9.75631	-15.18166
H	-8.33182	8.61456	-13.97950
H	-9.17899	11.54046	-14.20120
H	-9.77893	10.39253	-13.00434
H	-10.09940	10.12420	-16.02767
H	-10.68768	8.96460	-14.83701
H	-11.55654	11.88332	-15.03192
H	-12.14624	10.72033	-13.84434
H	-12.48827	10.47732	-16.87294
H	-13.74767	10.94237	-15.72702
H	-13.07468	9.31114	-15.68646
H	5.02156	1.55427	-13.03602
H	4.53003	1.33132	-14.71334
H	7.36395	0.94605	-13.64483
H	6.87266	0.73531	-15.32447
H	5.78741	-0.86516	-12.96307
H	5.30689	-1.07522	-14.64538
H	8.13598	-1.46083	-13.57013
H	7.63593	-1.69597	-15.24444
H	6.58528	-3.28070	-12.84967
H	7.74494	-3.86743	-14.04437
H	6.08322	-3.51615	-14.52410
H	-3.93492	-6.12002	-18.03672
H	-2.27943	-5.62603	-17.68802
H	-3.34817	-6.31375	-20.46531
H	-1.68609	-5.86283	-20.08669
H	-3.14511	-8.31794	-19.01023

H	-1.48954	-7.86527	-18.60670
H	-2.50029	-8.50860	-21.41360
H	-0.84231	-8.07489	-20.99783
H	-2.33197	-10.53965	-19.97045
H	-0.67393	-10.10577	-19.55265
H	-1.08109	-10.53553	-21.21545
H	-9.23289	1.58819	-6.19362
H	-7.73960	2.51622	-6.31814
H	-9.15489	1.80931	-3.70586
H	-7.67121	2.75274	-3.84146
H	-10.37517	3.52635	-5.02721
H	-8.89212	4.46719	-5.18271
H	-10.26954	3.77755	-2.54872
H	-8.79294	4.72809	-2.71115
H	-11.51982	5.49189	-3.86474
H	-10.04301	6.44260	-4.03004
H	-10.76719	6.20772	-2.43767
H	3.84619	7.68745	9.14619
H	5.19706	8.74038	9.56208
H	2.71353	8.29669	11.28336
H	4.06433	9.35200	11.69744
H	2.26508	9.66882	9.25257
H	3.61394	10.72520	9.66898
H	2.48083	11.32862	11.80398
H	1.13106	10.27387	11.38469
H	0.66985	11.66698	9.36365
H	2.02028	12.72173	9.78294
H	0.60641	12.63780	10.83635
H	0.63920	-8.72438	-9.28878
H	1.39420	-10.16552	-8.61095
H	2.87318	-7.88262	-10.00000
H	3.63531	-9.31700	-9.31303
H	1.57929	-9.32265	-11.56826
H	2.34242	-10.75621	-10.88177
H	3.80880	-8.47710	-12.28475
H	4.57445	-9.90787	-11.59425
H	2.52481	-9.93053	-13.86261
H	3.30211	-11.35776	-13.17656
H	4.26500	-10.16180	-14.04737
H	6.94578	17.19877	-0.49161
H	5.31303	16.79415	-1.04109
H	5.76194	17.92748	-3.20954
H	7.39049	18.32913	-2.66766
H	4.81320	19.25007	-1.31153
H	6.44663	19.66850	-0.79563
H	5.20662	20.38465	-3.49074
H	6.84358	20.79875	-2.98309
H	8.00285	4.85813	-10.07050
H	8.00744	4.10543	-8.47753
H	9.38106	2.98313	-10.96470
H	7.82355	2.46646	-10.31748
H	10.54854	2.17790	-8.99262
H	9.02964	1.86246	-8.15076
H	9.80526	0.46090	-10.74032
H	10.25510	-0.15243	-9.15045
H	7.38342	0.28118	-10.09889
H	7.86045	-0.30735	-8.50720
H	8.20833	-1.26828	-9.94484
H	12.41869	-0.55499	0.38945
H	13.24025	-1.12723	1.83951
H	14.42704	-1.14702	-0.97415
H	15.24174	-1.71851	0.48161
H	13.43935	-3.41914	0.80801
H	12.65833	-2.86396	-0.67114
H	14.69813	-3.47361	-1.97128

H	15.46924	-4.03883	-0.48966
H	14.50884	-5.94111	-1.75854
H	13.67284	-5.75728	-0.21514
H	12.91079	-5.19362	-1.70235
H	5.92439	22.13104	-1.09354
H	4.28548	21.71010	-1.59052
H	4.65141	22.86234	-3.77542
H	6.29046	23.28452	-3.27854
H	4.90586	23.96544	-2.42123
C	2.21567	-11.45937	4.58790
H	3.10595	-10.95978	4.21166
H	1.74015	-11.97002	3.75386
H	2.52224	-12.20486	5.31881
H	0.36952	-10.99515	5.57636
H	1.73101	-9.99982	6.10032
C	-2.14370	-6.00890	2.01782
H	-2.54512	-5.21964	1.38546
H	-2.16002	-5.67121	3.05229
H	-2.78498	-6.88272	1.93149
H	-0.72409	-6.68816	0.55161
H	-0.09046	-5.46815	1.66451
C	-9.79869	-7.48315	-6.38162
H	-10.26976	-7.16719	-7.31025
H	-10.44891	-7.21193	-5.55234
H	-9.70142	-8.56656	-6.40819
H	-8.57078	-5.73064	-6.23029
H	-7.98634	-7.08988	-5.26991
C	-3.63185	-8.72545	-10.37369
H	-2.96922	-8.74502	-11.23701
H	-3.82154	-9.75454	-10.07791
H	-3.11946	-8.21480	-9.56125
H	-4.65153	-7.04338	-11.21176
H	-5.45413	-8.58776	-11.49190
C	1.16961	9.12948	-9.06079
H	1.99838	8.89013	-8.39711
H	1.22984	8.48452	-9.93491
H	0.23577	8.92105	-8.54290
H	0.39263	10.80627	-10.14829
H	1.12184	11.23023	-8.59692
C	7.14499	10.13612	-12.19615
H	7.22842	9.46067	-11.34771
H	8.14016	10.30598	-12.60200
H	6.54082	9.64909	-12.95816
H	7.16244	11.95649	-11.04159
H	6.43564	12.11304	-12.64538
C	7.24647	8.20463	-0.83214
H	6.24431	8.32126	-1.23939
H	7.26761	7.31487	-0.20571
H	7.94072	8.06156	-1.65748
H	7.59529	10.31578	-0.66681
H	6.91786	9.58055	0.78956
C	13.72797	7.48265	1.50354
H	14.69852	7.34127	1.97469
H	13.88658	7.84951	0.49221
H	13.23572	6.51467	1.43957
H	12.78076	8.10079	3.33766
H	13.41933	9.42572	2.35918
H	9.05507	-1.17519	6.06111
H	7.13551	1.74498	8.54879
H	10.49311	0.53946	5.09157
H	8.58504	7.27365	6.53719
H	9.12085	8.88227	4.74715
H	11.41817	5.93033	2.68397
H	11.10913	8.13877	-2.15847
H	9.17467	11.84721	-1.27520

H	10.62992	8.40911	-4.56335
H	6.46506	14.02308	-6.26379
H	5.09467	13.74232	-8.29369
H	6.91524	9.95794	-9.08496
H	0.33800	11.47826	-12.26322
H	3.11506	8.25907	-12.67017
H	-1.46105	10.10683	-13.23892
H	0.29360	3.29214	-14.61947
H	-1.53506	1.66002	-14.39691
H	-4.37366	4.80683	-13.82220
H	8.22339	8.42159	2.35543
H	12.31545	9.56152	-0.06925
H	3.39264	11.57106	-8.30870
H	4.29322	10.62728	-13.02236
H	5.42495	0.53220	8.15367
H	8.30303	-2.55832	7.40090
H	3.63799	-1.11944	8.30637
H	5.44907	-7.70353	6.11230
H	3.65747	-8.91022	4.94624
H	0.79992	-5.95954	6.12781
H	0.88637	-8.95192	0.00510
H	-3.01795	-9.51221	1.64980
H	0.01556	-9.23858	-2.29258
H	-7.09710	-9.90918	-2.96356
H	-7.98087	-9.33858	-5.16998
H	-4.03769	-8.49710	-6.59837
H	-6.62759	-6.73361	-12.41370
H	-6.83675	-4.06348	-9.07365
H	-6.07894	-4.87250	-13.90530
H	-5.94409	1.61271	-10.82260
H	-4.64080	3.36016	-11.96471
H	-3.67508	0.67242	-15.14912
H	0.85296	-6.04917	3.72714
H	-0.19136	-10.77306	3.00098
H	-5.09932	-9.28941	-8.39391
H	-8.34094	-5.66548	-8.57499
H	3.10304	6.04958	-11.97284
H	5.07513	4.62199	-12.24285
H	3.77167	3.54192	-16.15903
H	1.79557	4.98005	-15.89602
H	-2.92011	9.14835	-15.12227
H	-4.90718	10.56313	-14.81460
H	-6.38437	7.84684	-11.87543
H	-4.39267	6.44601	-12.16269
H	8.54692	7.66041	-5.52689
H	9.15835	5.49674	-6.51739
H	9.94881	7.42347	-10.24150
H	9.37976	9.59253	-9.24226
H	5.86520	13.19019	-4.04716
H	4.99087	15.18537	-2.90589
H	8.87534	15.99033	-1.33588
H	9.75342	14.00417	-2.48181
H	12.60176	2.07947	5.23834
H	14.02295	0.74252	3.74335
H	12.17934	2.57205	0.35855
H	10.74725	3.89350	1.84495
H	9.16103	6.04286	8.74742
H	7.70805	7.32919	10.25734
H	4.42654	4.79239	9.25807
H	5.86680	3.52421	7.73021
H	6.72104	-5.95755	4.97486
H	8.79717	-7.06314	4.26198
H	10.69338	-5.83752	7.88259
H	8.61613	-4.74485	8.60423
H	2.21581	-2.92944	9.53837
H	0.25538	-1.51029	9.96220
H	-1.17323	-2.31095	6.02245
H	0.79240	-3.70779	5.58848
H	-2.66284	-1.48473	-15.03503
H	-1.95653	-2.58307	-17.11282
H	-5.89283	-4.14257	-17.63355
H	-6.60676	-3.03352	-15.55777
H	-8.52601	-2.21806	-9.09254
H	-8.96732	-1.26538	-6.87354
H	-5.32898	0.95171	-7.09682
H	-4.87307	-0.01676	-9.30798
H	-6.19756	-11.54697	-1.22728
H	-8.23324	-11.75729	0.13578
H	-6.99607	-8.42224	2.49306
H	-4.96821	-8.20787	1.13169
H	-0.61171	-10.70617	-4.27688
H	1.36061	-10.34524	-5.69998
H	-0.40660	-6.78032	-7.24081
H	-2.36209	-7.12093	-5.80254

*Table 12 Cartesian coordinates of **1l** (GFN2-xTB(GBSA(toluene)). B97-3c(COSMO(toluene) single point energy = 10838.55005341 Hartree.*

	X	Y	Z
C	10.83735	-0.30194	6.00085
C	9.87748	-0.18514	7.00699
C	9.50712	1.09336	7.45292
C	10.01464	2.20687	6.81788
C	10.95897	2.06284	5.76657
C	11.39546	0.81026	5.38858
C	11.17573	3.38070	5.22250
C	10.36618	4.31051	5.99973
C	11.68456	3.99580	4.11671
C	10.41295	5.58503	5.31151
C	9.67553	3.63308	6.96440
C	9.80898	6.80887	5.52027
C	9.91360	7.79187	4.53989
C	10.63149	7.57933	3.36728
C	11.31332	6.36787	3.19403
C	11.19255	5.37783	4.14101
C	10.00078	8.64399	-2.58957
C	9.77129	9.75518	-1.78128
C	9.23799	10.92244	-2.33339
C	8.87108	10.92374	-3.66660
C	8.98239	9.73588	-4.43506
C	9.59936	8.61708	-3.91689
C	8.32405	9.99553	-5.69164
C	7.91180	11.39422	-5.68848
C	7.88380	9.36767	-6.81706
C	7.23774	11.63416	-6.95023
C	8.22432	11.96375	-4.48568
C	6.67493	12.73886	-7.55884
C	5.98189	12.57705	-8.75534
C	5.80485	11.32473	-9.34111
C	6.42847	10.21543	-8.75321
C	7.17208	10.37681	-7.60815
C	1.71300	8.99527	-13.98314
C	1.59632	9.60924	-12.73708
C	0.42761	9.39674	-11.99315
C	-0.55128	8.56180	-12.47722
C	-0.38470	7.87783	-13.71147
C	0.74129	8.12640	-14.47096
C	-1.51943	6.98371	-13.83449

C	-2.38992	7.24902	-12.69554	C	-6.73846	0.22753	-12.26570
C	-1.94494	5.86213	-14.48923	C	-7.53479	1.35531	-12.27515
C	-3.43419	6.25569	-12.71039	C	-6.97408	2.56574	-12.65785
C	-1.82684	8.17308	-11.86733	C	-5.64017	2.65917	-13.05267
C	-4.50670	6.01792	-11.87747	C	-4.89241	1.48336	-13.21406
C	-5.25289	4.86639	-12.07259	C	-5.42520	0.27935	-12.80786
C	-4.95342	3.95803	-13.08898	C	1.86744	-7.63739	5.23409
C	-3.90563	4.23929	-13.97764	C	2.20819	-7.47412	3.89538
C	-3.14279	5.37319	-13.78474	C	1.48007	-8.03897	2.86287
C	10.51512	8.46698	2.19562	C	0.36725	-8.82454	3.19059
C	9.22186	8.77271	1.77588	C	0.05643	-9.02828	4.53055
C	8.94888	9.30866	0.52921	C	0.78169	-8.44687	5.56316
C	10.02064	9.54618	-0.33687	C	-6.19434	-6.99551	-5.59641
C	11.31656	9.37528	0.13286	C	-6.28258	-7.75691	-6.75967
C	11.59190	8.84797	1.38745	C	-6.37651	-7.19388	-8.02489
C	4.86457	11.09335	-10.44916	C	-6.36958	-5.80226	-8.13108
C	3.63463	11.74909	-10.41639	C	-6.32798	-5.04249	-6.96729
C	2.56555	11.40933	-11.22954	C	-6.24162	-5.59926	-5.70215
C	2.71672	10.31478	-12.09454	C	-6.54479	-8.07730	-9.23002
C	3.99482	9.77781	-12.24466	C	-6.14209	-4.65174	-4.53603
C	5.07885	10.15604	-11.47052	C	0.42894	-8.73092	6.99665
C	1.32454	12.26605	-11.18430	C	1.84217	-7.66249	1.45078
C	6.43647	9.59054	-11.80506	C	-2.35103	8.70304	-10.61847
C	13.02354	8.71882	1.84056	C	-1.31336	5.21591	-15.62827
C	7.51711	9.49504	0.10576	C	-3.52916	-1.45204	-13.26610
C	9.74466	-2.64942	7.41105	C	-8.96758	-1.46340	-10.26355
C	9.14556	-1.38952	7.42052	C	-2.15852	-10.19106	-3.50946
C	7.76447	-1.30114	7.65274	C	-4.83629	-7.77024	1.51466
C	7.00997	-2.45055	7.70994	C	7.17486	-7.00766	6.61694
C	7.61967	-3.72465	7.55323	C	4.60139	-1.54374	8.04283
C	8.99556	-3.81693	7.48110	C	12.52242	3.41436	3.08060
C	6.54031	-4.68096	7.44011	C	8.73912	4.17121	7.93713
C	5.29907	-3.95121	7.67630	C	8.04372	7.97735	-7.21214
C	6.26249	-5.94855	7.01524	C	7.96108	13.31788	-4.02848
C	4.20642	-4.85266	7.38583	C	-7.70163	-1.82498	-10.88025
C	5.55501	-2.62062	7.82601	C	-6.90661	-1.09914	-11.71942
C	2.83729	-4.68842	7.33736	C	-1.57511	8.74213	-9.46071
C	2.06727	-5.67861	6.73796	C	-2.09003	9.26258	-8.28920
C	2.65108	-6.81115	6.17877	C	-3.38293	9.77039	-8.23763
C	4.02742	-7.01973	6.30904	C	-4.15771	9.72473	-9.39170
C	4.79865	-6.05988	6.93059	C	-3.65499	9.19592	-10.56361
C	-0.07575	-10.10605	1.07874	C	-3.94866	10.30597	-6.95511
C	-0.53144	-9.30459	2.12032	C	-0.92154	5.97086	-16.73401
C	-1.84465	-8.81998	2.09430	C	-0.28696	5.37313	-17.80442
C	-2.61598	-9.01948	0.96950	C	-0.01097	4.01026	-17.80218
C	-2.08624	-9.72512	-0.14514	C	-0.40625	3.25781	-16.70152
C	-0.84393	-10.31932	-0.06054	C	-1.05202	3.84583	-15.63188
C	-3.03603	-9.55651	-1.22337	C	0.67108	3.36155	-18.97089
C	-4.17616	-8.83137	-0.67879	C	7.78112	6.94315	-6.31434
C	-3.10748	-9.59001	-2.58603	C	7.94214	5.62562	-6.69554
C	-5.01441	-8.45372	-1.79452	C	8.38005	5.29910	-7.97439
C	-3.94981	-8.50592	0.62718	C	8.63991	6.33192	-8.86814
C	-6.19734	-7.75141	-1.89391	C	8.46825	7.65160	-8.50002
C	-6.63265	-7.34993	-3.15152	C	6.72481	13.92001	-4.26432
C	-5.90694	-7.64316	-4.30215	C	6.47382	15.20434	-3.82477
C	-4.77986	-8.47084	-4.21078	C	7.44510	15.93016	-3.14358
C	-4.32695	-8.86239	-2.96943	C	8.67869	15.33103	-2.91416
C	-5.15616	-5.10440	-10.18817	C	8.93497	14.04316	-3.34148
C	-6.28839	-5.02229	-9.38348	C	8.52796	3.86642	-8.39611
C	-7.23182	-4.01654	-9.62056	C	13.64468	2.66416	3.43199
C	-6.98943	-3.07927	-10.60127	C	14.45113	2.10518	2.46004
C	-5.78302	-3.11574	-11.34974	C	14.16178	2.26701	1.10997
C	-4.89393	-4.15664	-11.17180	C	13.03775	3.00893	0.76148
C	-5.70677	-1.85476	-12.05679	C	12.23003	3.57733	1.72620
C	-4.81608	-1.05818	-12.71908	C	15.01150	1.63319	0.04707

C	9.06914	5.30955	8.67354
C	8.19490	5.81589	9.61488
C	6.96563	5.20941	9.84882
C	6.62870	4.08745	9.09966
C	7.49892	3.57252	8.15976
C	6.00324	5.77385	10.85236
C	3.65001	-1.64917	9.05803
C	2.75617	-0.62366	9.29181
C	2.77053	0.52948	8.51363
C	3.70161	0.62142	7.48597
C	4.61099	-0.39315	7.25585
C	8.27051	-6.73854	5.79663
C	9.12237	-7.75304	5.40850
C	8.91420	-9.06284	5.82759
C	7.82426	-9.32900	6.64859
C	6.96007	-8.32229	7.03185
C	9.86874	-10.15323	5.43857
C	1.77135	1.62368	8.75272
C	-2.38263	-0.66877	-13.14081
C	-1.18702	-1.07643	-13.70320
C	-1.09070	-2.27768	-14.39438
C	-2.23043	-3.07029	-14.49957
C	-3.42858	-2.66698	-13.94983
C	-10.02540	-2.37225	-10.22800
C	-11.22485	-2.02959	-9.63546
C	-11.40218	-0.78260	-9.04658
C	-10.34302	0.11831	-9.07380
C	-9.14419	-0.21188	-9.67370
C	-12.71904	-0.40160	-8.43626
C	0.19476	-2.71577	-15.03468
C	-6.17966	-8.13993	1.60601
C	-7.04383	-7.45375	2.43399
C	-6.60369	-6.36958	3.18732
C	-5.26742	-6.00042	3.09268
C	-4.39238	-6.68923	2.27401
C	-1.71291	-11.49607	-3.29535
C	-0.81803	-12.08597	-4.16544
C	-0.32886	-11.39179	-5.26668
C	-0.76325	-10.08707	-5.47146
C	-1.66576	-9.49261	-4.61129
C	0.60937	-12.04913	-6.23587
C	-7.56628	-5.60417	4.04866
C	0.43784	1.30330	8.06522
C	-0.60084	2.39772	8.29884
C	-1.93794	2.07019	7.63741
C	-2.98027	3.16182	7.87289
C	-4.31670	2.82453	7.22159
C	-8.50930	-4.73365	3.20602
C	-7.76281	-3.64701	2.43602
C	-8.70549	-2.79440	1.58962
C	-7.96034	-1.70886	0.81533
C	-8.90338	-0.86638	-0.03625
C	11.02679	-10.24389	6.44113
C	12.01233	-11.35072	6.07363
C	13.16554	-11.43733	7.07194
C	14.22541	-12.47204	6.68881
C	13.72689	-13.91053	6.77949
C	-0.35782	2.77515	-19.94699
C	0.27321	2.25764	-21.24016
C	1.22606	1.08358	-21.01840
C	1.66413	0.40882	-22.31940
C	2.49054	1.31809	-23.22260
C	-4.67857	9.19932	-6.18250
C	-5.25877	9.70941	-4.86546
C	-5.98662	8.60350	-4.10294
C	-6.66583	9.09160	-2.82186
C	-5.67983	9.51417	-1.73781
C	0.10773	-2.63803	-16.56412
C	1.38769	-3.12835	-17.23759
C	1.24148	-3.19894	-18.75643
C	2.52149	-3.67353	-19.44158
C	2.32807	-3.85375	-20.94304
C	-13.63248	0.25009	-9.48291
C	-14.98190	0.65120	-8.89190
C	-15.89426	1.29770	-9.93243
C	-17.24746	1.69529	-9.34555
C	-18.15409	2.33905	-10.38860
C	5.01478	6.73667	10.18133
C	4.01903	7.31733	11.18267
C	3.03251	8.27811	10.52187
C	2.03995	8.86383	11.52460
C	1.05969	9.82501	10.86157
C	-0.17266	-12.74389	-7.35841
C	0.75459	-13.40876	-8.37281
C	-0.02160	-14.10391	-9.48956
C	0.90325	-14.76427	-10.51028
C	0.12271	-15.45968	-11.61996
C	7.18536	17.34187	-2.70612
C	7.59077	18.33392	-3.80424
C	7.36203	19.78161	-3.37637
C	7.76724	20.77321	-4.46513
C	7.22604	3.35716	-9.02968
C	7.34002	1.94293	-9.60090
C	7.50780	0.86746	-8.52863
C	7.61272	-0.54663	-9.10457
C	6.31102	-1.05269	-9.71723
C	14.32926	0.40040	-0.56513
C	14.08661	-0.72987	0.43681
C	15.35084	-1.43537	0.93253
C	16.04984	-2.26409	-0.14410
C	17.24214	-3.02921	0.42016
C	7.55211	22.22271	-4.03351
C	7.96137	23.20845	-5.12211
H	-4.65133	11.11485	-7.16753
H	-3.14515	10.70569	-6.33275
H	1.32606	2.56560	-18.61353
H	1.27965	4.09913	-19.49859
H	8.77790	3.25464	-7.52835
H	9.33578	3.77705	-9.12598
H	15.97831	1.35003	0.46454
H	15.18960	2.35736	-0.75232
H	5.44369	4.96270	11.32400
H	6.54982	6.30971	11.63126
H	10.27405	-9.95467	4.44379
H	9.34599	-11.11173	5.40950
H	1.59836	1.73919	9.82535
H	2.15414	2.56977	8.36413
H	-12.55930	0.30135	-7.61570
H	-13.21355	-1.28892	-8.03502
H	0.40952	-3.74874	-14.74759
H	1.01833	-2.08953	-14.68595
H	1.22274	-12.78944	-5.71773
H	1.27370	-11.30158	-6.67493
H	-7.01551	-4.96680	4.74328
H	-8.16727	-6.30587	4.63291
H	0.05779	0.35249	8.44648
H	0.60807	1.18253	6.99244
H	-0.75354	2.52743	9.37341
H	-0.22468	3.34439	7.90271
H	-1.78748	1.94092	6.56252

H	-2.31338	1.12305	8.03313	H	-16.05143	0.59917	-10.75825
H	-2.60936	4.10740	7.46970	H	-15.40415	2.18519	-10.34082
H	-3.12548	3.29563	8.94779	H	-17.73795	0.80791	-8.93765
H	-4.19797	2.70478	6.14644	H	-17.09121	2.39475	-8.52049
H	-5.04097	3.61633	7.40116	H	-18.33878	1.65065	-11.21101
H	-4.71492	1.89622	7.62654	H	-17.69283	3.23802	-10.79289
H	-9.24521	-4.26817	3.86620	H	-19.11065	2.61247	-9.94830
H	-9.04927	-5.36787	2.49942	H	4.47424	6.20440	9.39492
H	-7.01750	-4.11261	1.78638	H	5.57158	7.54832	9.70654
H	-7.23091	-3.00386	3.14185	H	3.46579	6.50210	11.65599
H	-9.45211	-2.32700	2.23695	H	4.56405	7.84576	11.96905
H	-9.23698	-3.43765	0.88360	H	3.58530	9.09183	10.04545
H	-7.43233	-1.06138	1.51991	H	2.48350	7.74996	9.73812
H	-7.21018	-2.17707	0.17302	H	2.58895	9.39088	12.30892
H	-9.64283	-0.37114	0.59007	H	1.48508	8.05081	11.99946
H	-9.42876	-1.49109	-0.75600	H	1.59242	10.65229	10.39656
H	-8.35019	-0.10481	-0.58198	H	0.48472	9.31349	10.09208
H	10.62238	-10.43118	7.43897	H	0.36662	10.23186	11.59494
H	11.54820	-9.28399	6.47271	H	-0.80022	-12.00701	-7.86554
H	11.47968	-12.30269	6.04169	H	-0.83511	-13.49487	-6.92111
H	12.41454	-11.15954	5.07535	H	1.41415	-12.65380	-8.80838
H	12.76966	-11.67752	8.06188	H	1.38451	-14.14114	-7.86162
H	13.64395	-10.45636	7.13900	H	-0.65501	-13.37262	-9.99802
H	15.08153	-12.35503	7.35838	H	-0.67792	-14.86220	-9.05505
H	14.57316	-12.27207	5.67223	H	1.55709	-14.00573	-10.94785
H	13.34728	-14.11912	7.77792	H	1.53840	-15.49399	-10.00202
H	14.53877	-14.60411	6.56994	H	-0.50371	-14.74554	-12.15089
H	12.93120	-14.09552	6.06234	H	-0.51891	-16.23551	-11.20674
H	-1.08405	3.55326	-20.19711	H	0.80097	-15.92011	-12.33525
H	-0.90128	1.96611	-19.45284	H	7.75644	17.56070	-1.80113
H	0.80592	3.07744	-21.72545	H	6.12471	17.47317	-2.48095
H	-0.52886	1.93806	-21.91113	H	7.01368	18.12353	-4.70799
H	2.11514	1.42492	-20.48476	H	8.64618	18.18721	-4.04650
H	0.72480	0.34025	-20.39254	H	6.30615	19.92480	-3.13292
H	2.26322	-0.47010	-22.06740	H	7.93937	19.98564	-2.47093
H	0.78173	0.06257	-22.86371	H	7.18459	20.57609	-5.36861
H	3.34664	1.71601	-22.68112	H	8.82105	20.62469	-4.71426
H	1.89518	2.15053	-23.58884	H	6.94536	4.03956	-9.83640
H	2.85802	0.76132	-24.08235	H	6.42837	3.38645	-8.28334
H	-5.48187	8.79832	-6.80553	H	8.18707	1.89951	-10.29040
H	-3.98015	8.38291	-5.98194	H	6.43440	1.73736	-10.17468
H	-4.44877	10.10920	-4.25300	H	8.41563	1.06944	-7.95656
H	-5.95713	10.52522	-5.06972	H	6.66225	0.91192	-7.83737
H	-5.27835	7.80897	-3.85446	H	8.40222	-0.56945	-9.85992
H	-6.74788	8.17088	-4.75797	H	7.90714	-1.22646	-8.30060
H	-7.29043	8.28354	-2.43193	H	6.02617	-0.45882	-10.58204
H	-7.32415	9.93061	-3.06046	H	5.50446	-1.00822	-8.98775
H	-4.99460	8.70017	-1.50876	H	6.42411	-2.08580	-10.03980
H	-5.09816	10.37695	-2.05225	H	14.93731	0.03517	-1.39417
H	-6.21263	9.77966	-0.82688	H	13.36376	0.70669	-0.97519
H	-0.09265	-1.60580	-16.86155	H	13.54946	-0.32131	1.29650
H	-0.73284	-3.24616	-16.90540	H	13.43608	-1.47372	-0.03139
H	2.21472	-2.46192	-16.98062	H	15.07036	-2.09985	1.75449
H	1.63561	-4.12275	-16.85728	H	16.05318	-0.70342	1.33454
H	0.97056	-2.21335	-19.14236	H	15.33562	-2.97166	-0.57317
H	0.42721	-3.88438	-19.00539	H	16.39604	-1.61260	-0.94860
H	3.31848	-2.94799	-19.26109	H	16.92314	-3.70657	1.21019
H	2.83472	-4.62397	-19.00218	H	17.72562	-3.61469	-0.35917
H	1.99802	-2.92343	-21.40042	H	17.97548	-2.34106	0.83655
H	1.57610	-4.61498	-21.14165	H	8.13411	22.41951	-3.12963
H	3.25880	-4.15946	-21.41630	H	6.49816	22.37282	-3.78647
H	-13.78847	-0.45016	-10.30716	H	7.37787	23.04348	-6.02583
H	-13.13536	1.13334	-9.89132	H	9.01444	23.08856	-5.36919
H	-15.47423	-0.23417	-8.48159	H	7.80060	24.23256	-4.79183
H	-14.82221	1.35123	-8.06782	C	1.26511	-9.89614	7.53130

H	2.32376	-9.64925	7.48865
H	1.09593	-10.79057	6.93497
H	1.00029	-10.11088	8.56452
H	-0.63112	-8.98260	7.07295
H	0.61998	-7.84901	7.61044
C	0.99441	-6.47753	0.98198
H	1.28814	-6.17503	-0.02103
H	1.11986	-5.63015	1.65338
H	-0.05809	-6.75217	0.96410
H	1.68953	-8.49393	0.76505
H	2.89757	-7.38289	1.41681
C	-7.48965	-3.99252	-4.23248
H	-7.84098	-3.42823	-5.09381
H	-7.39421	-3.31243	-3.38879
H	-8.24160	-4.74046	-3.99085
H	-5.42150	-3.86770	-4.78616
H	-5.77770	-5.16869	-3.64798
C	-8.02871	-8.36133	-9.47717
H	-8.15211	-9.00637	-10.34464
H	-8.56450	-7.43169	-9.65737
H	-8.47232	-8.85129	-8.61278
H	-6.01994	-9.02204	-9.07073
H	-6.12732	-7.59448	-10.11409
C	0.64197	12.29459	-9.81483
H	1.29999	12.71931	-9.06066
H	0.36329	11.29458	-9.48979
H	-0.26141	12.89945	-9.86203
H	0.61390	11.93353	-11.94207
H	1.61927	13.28961	-11.43929
C	6.51611	8.06637	-11.69980
H	6.28958	7.72665	-10.69124
H	7.51830	7.73012	-11.95691
H	5.81234	7.59122	-12.37877
H	7.19514	10.04471	-11.16634
H	6.66671	9.87377	-12.83782
C	6.95989	8.21335	-0.51799
H	5.91858	8.35198	-0.80119
H	7.02270	7.38811	0.18862
H	7.52801	7.94961	-1.40749
H	7.43736	10.30564	-0.61943
H	6.91601	9.75777	0.97957
C	13.86818	7.81507	0.94022
H	14.88200	7.74524	1.32911
H	13.91596	8.20803	-0.07245
H	13.45269	6.81082	0.89327
H	13.05152	8.34051	2.86389
H	13.47130	9.71794	1.84410
H	11.11396	-1.28389	5.64682
H	8.80997	1.19544	8.27130
H	12.11552	0.69156	4.59253
H	9.22256	6.99192	6.40898
H	9.40737	8.73632	4.68292
H	11.86980	6.19616	2.28695
H	10.45173	7.76574	-2.14852
H	9.09609	11.79639	-1.71284
H	9.74044	7.73061	-4.51737
H	6.75665	13.71931	-7.11215
H	5.55177	13.44797	-9.22946
H	6.27946	9.23031	-9.16400
H	2.58789	9.18645	-14.58815
H	0.32095	9.84366	-11.01984
H	0.88671	7.63765	-15.42326
H	-4.73526	6.68796	-11.06200
H	-6.05272	4.63781	-11.38370
H	-3.69633	3.56210	-14.79314

H	8.39450	8.51002	2.42128
H	12.13701	9.62953	-0.52414
H	3.49729	12.53820	-9.69132
H	4.13660	8.99675	-12.97716
H	10.81904	-2.72336	7.32421
H	7.28891	-0.33472	7.71690
H	9.48565	-4.77894	7.44504
H	2.37465	-3.78090	7.69686
H	1.00148	-5.53077	6.63094
H	4.47925	-7.89642	5.86623
H	0.90642	-10.55287	1.14778
H	-2.21221	-8.24497	2.93175
H	-0.44990	-10.90069	-0.88107
H	-6.75293	-7.47105	-1.01120
H	-7.54503	-6.77928	-3.23546
H	-4.24431	-8.73697	-5.11012
H	-4.42480	-5.87569	-9.99027
H	-8.10710	-3.94570	-8.98995
H	-3.97379	-4.20256	-11.73567
H	-8.56327	1.30978	-11.94848
H	-7.57794	3.46113	-12.62153
H	-3.88171	1.54586	-13.58593
H	3.05101	-6.83899	3.65552
H	-0.79633	-9.64839	4.77401
H	-6.26771	-8.83524	-6.67010
H	-6.31377	-3.96463	-7.06105
H	-0.57557	8.33125	-9.47455
H	-1.47606	9.27898	-7.39918
H	-5.16624	10.11395	-9.37092
H	-4.26072	9.18101	-11.45870
H	-1.13622	7.02989	-16.75000
H	0.00687	5.97436	-18.65384
H	-0.19675	2.19731	-16.67913
H	-1.32058	3.25104	-14.77099
H	7.43020	7.18331	-5.32087
H	7.72895	4.83597	-5.98815
H	8.98386	6.09645	-9.86548
H	8.69383	8.44277	-9.19994
H	5.95530	13.35871	-4.77427
H	5.50660	15.65168	-4.00781
H	9.44761	15.88153	-2.39001
H	9.90539	13.60004	-3.17005
H	13.88880	2.54127	4.47764
H	15.32036	1.53441	2.75549
H	12.79339	3.14381	-0.28322
H	11.34460	4.12324	1.43350
H	10.03074	5.77717	8.51606
H	8.47265	6.69228	10.18406
H	5.67295	3.60876	9.26107
H	7.20835	2.71826	7.56672
H	3.63751	-2.53562	9.67637
H	2.03675	-0.71451	10.09383
H	3.71865	1.50322	6.86003
H	5.31520	-0.31161	6.44004
H	8.42976	-5.72982	5.44453
H	9.96044	-7.52808	4.76340
H	7.64784	-10.34074	6.98665
H	6.12386	-8.54467	7.67924
H	-2.41894	0.24618	-12.56800
H	-0.31022	-0.45278	-13.59393
H	-2.17433	-4.01649	-15.01939
H	-4.31128	-3.28049	-14.05988
H	-9.90805	-3.34147	-10.69136
H	-12.03725	-2.74290	-9.62443
H	-10.45889	1.08949	-8.61281

H	-8.32103	0.48763	-9.66649
H	-6.53068	-8.98720	1.03397
H	-8.07792	-7.76250	2.49990
H	-4.90592	-5.15751	3.66511
H	-3.36537	-6.36428	2.19309
H	-2.09492	-12.04945	-2.44919
H	-0.48995	-13.10083	-3.98773
H	-0.38424	-9.52672	-6.31496
H	-1.96650	-8.46736	-4.77129

*Table 13 Cartesian coordinates of **1m** (GFN2-xTB(GBSA(toluene)). B97-3c(COSMO(toluene) single point energy = 10838.5549284 Hartree.*

	X	Y	Z
C	9.92028	-1.27362	5.83206
C	9.03641	-1.09731	6.89860
C	8.83814	0.19565	7.40831
C	9.44207	1.26932	6.79033
C	10.26587	1.07424	5.65054
C	10.54132	-0.20259	5.20651
C	10.52229	2.38308	5.10054
C	9.83584	3.35260	5.94603
C	10.99559	2.98313	3.97070
C	9.95294	4.63895	5.28823
C	9.21546	2.71137	6.98014
C	9.51009	5.91072	5.59086
C	9.62448	6.91330	4.63186
C	10.20435	6.66770	3.39281
C	10.75535	5.40822	3.12787
C	10.61824	4.39935	4.05485
C	8.00244	10.83623	-1.06731
C	9.15751	10.06428	-1.12224
C	9.86107	9.94607	-2.32391
C	9.34106	10.52075	-3.46531
C	8.10296	11.21327	-3.41743
C	7.46471	11.41618	-2.21102
C	7.70812	11.43362	-4.79097
C	8.75815	10.88257	-5.63922
C	6.61217	11.75203	-5.53749
C	8.27535	10.94827	-7.00278
C	9.75422	10.35723	-4.86736
C	8.81762	10.60454	-8.22454
C	7.99728	10.60276	-9.34753
C	6.65581	10.96228	-9.27010
C	6.13804	11.42890	-8.05407
C	6.93494	11.41610	-6.93116
C	2.40320	8.34059	-13.75649
C	2.43145	9.09329	-12.58825
C	1.26062	9.23424	-11.83156
C	0.13485	8.52537	-12.18619
C	0.13969	7.70081	-13.34477
C	1.25915	7.65170	-14.14981
C	-1.11650	6.98106	-13.34126
C	-1.86046	7.43474	-12.17326
C	-1.73699	5.90185	-13.90181
C	-2.99786	6.55960	-12.02369
C	-1.14324	8.36715	-11.48103
C	-3.97039	6.43131	-11.05375
C	-4.81422	5.33079	-11.09389
C	-4.74857	4.37803	-12.11328
C	-3.81718	4.56822	-13.14845
C	-2.92050	5.61089	-13.07705
C	10.06733	7.61643	2.26743
C	9.31785	7.14200	1.19434
C	9.03207	7.91463	0.08389
C	9.52243	9.22379	0.04316
C	10.28616	9.69498	1.10096
C	10.57378	8.91469	2.21655
C	5.71443	10.64607	-10.36056
C	5.70251	9.32096	-10.78704
C	4.71212	8.79904	-11.60265
C	3.66700	9.64382	-11.99542
C	3.72859	10.98997	-11.64629
C	4.74055	11.51729	-10.85696
C	4.78827	7.34060	-11.96550
C	4.78690	13.00069	-10.61082
C	11.48002	9.45408	3.28857
C	8.21507	7.31657	-1.02984
C	8.60530	-3.54857	7.16333
C	8.18479	-2.22511	7.29919
C	6.84781	-1.97662	7.64754
C	5.94714	-3.01634	7.68402
C	6.36598	-4.34249	7.38944
C	7.70924	-4.60849	7.21030
C	5.16288	-5.13834	7.28773
C	4.04929	-4.27244	7.65632
C	4.69268	-6.33507	6.82681
C	2.83008	-5.00253	7.39008
C	4.49262	-3.00480	7.89966
C	1.49547	-4.65348	7.43110
C	0.56373	-5.50371	6.84815
C	0.95016	-6.68516	6.22100
C	2.29287	-7.07650	6.24798
C	3.22465	-6.25207	6.84315
C	-3.91360	-7.55685	2.24543
C	-2.83817	-8.43581	2.31520
C	-2.68035	-9.42104	1.33332
C	-3.51882	-9.41658	0.23778
C	-4.57390	-8.46858	0.14750
C	-4.79661	-7.57277	1.17215
C	-5.14702	-8.61344	-1.17145
C	-4.41965	-9.68391	-1.83897
C	-5.95974	-7.96563	-2.05568
C	-4.81776	-9.66461	-3.23007
C	-3.46470	-10.19650	-1.00846
C	-4.39221	-10.35864	-4.34430
C	-4.80813	-9.93106	-5.60171
C	-5.67340	-8.85434	-5.75042
C	-6.18529	-8.21307	-4.61433
C	-5.73808	-8.59009	-3.36816
C	-5.52778	-5.23343	-11.39266
C	-6.13168	-5.26568	-10.14017
C	-6.79333	-4.12629	-9.66669
C	-6.73551	-2.95804	-10.39410
C	-5.96253	-2.89342	-11.58378
C	-5.42520	-4.04885	-12.11522
C	-5.88048	-1.49177	-11.93502
C	-5.18004	-0.61210	-12.70775
C	-6.59061	0.64304	-11.29368
C	-7.16401	1.79778	-10.79907
C	-6.63391	3.02521	-11.17267
C	-5.50834	3.12670	-11.99522
C	-5.01912	1.95546	-12.59605
C	-5.58030	0.73827	-12.28532
C	-0.00480	-7.36729	5.32405
C	0.33642	-7.35586	3.97449
C	-0.53664	-7.75683	2.97883
C	-1.81173	-8.19819	3.35257

C	-2.12742	-8.27739	4.70335
C	-1.24802	-7.87571	5.70132
C	-5.87362	-8.18776	-7.05263
C	-4.72432	-7.67738	-7.64999
C	-4.76275	-6.79149	-8.71310
C	-6.01482	-6.40430	-9.20641
C	-7.15823	-6.98854	-8.67231
C	-7.11634	-7.87752	-7.60693
C	-3.47029	-6.17477	-9.17700
C	-8.38618	-8.51461	-7.11413
C	-1.62869	-8.05448	7.14521
C	-0.12341	-7.56354	1.54489
C	-1.54450	9.05948	-10.26803
C	-1.29746	5.09702	-15.03044
C	-4.18282	-0.86152	-13.73542
C	-8.03365	-1.32325	-8.88118
C	-6.87226	-6.86474	-1.79364
C	-2.54276	-11.28429	-1.29146
C	5.43109	-7.47813	6.31636
C	3.70319	-1.85816	8.31608
C	11.71152	2.36352	2.86781
C	8.41260	3.27613	8.05302
C	5.35786	12.32170	-5.07282
C	10.97017	9.67161	-5.27382
C	-7.23595	-1.61796	-10.05995
C	-6.72953	-0.76339	-10.99546
C	-0.69514	9.18167	-9.16920
C	-1.11677	9.83412	-8.02631
C	-2.38942	10.38579	-7.94222
C	-3.23627	10.26282	-9.03931
C	-2.82623	9.60738	-10.18180
C	-2.83708	11.12725	-6.71677
C	-1.23417	3.70634	-14.93200
C	-0.79604	2.94629	-15.99742
C	-0.41754	3.54328	-17.19535
C	-0.48390	4.92834	-17.29371
C	-0.90842	5.69738	-16.22737
C	0.01678	2.69923	-18.35793
C	4.12374	11.79771	-5.45476
C	2.94994	12.36583	-4.99799
C	2.96749	13.47422	-4.15991
C	4.19978	13.99257	-3.77509
C	5.37707	13.42480	-4.21732
C	10.94162	8.69655	-6.27093
C	12.09655	8.03755	-6.64157
C	13.31426	8.33112	-6.03679
C	13.34139	9.30355	-5.04357
C	12.18853	9.95957	-4.65848
C	1.69318	14.07145	-3.63958
C	12.78868	1.51417	3.12343
C	13.47939	0.92201	2.08487
C	13.11583	1.14952	0.76209
C	12.03292	1.98435	0.50918
C	11.34190	2.58699	1.54161
C	13.83857	0.47126	-0.36458
C	7.43713	4.23679	7.78663
C	6.70152	4.79392	8.81346
C	6.91571	4.41557	10.13444
C	7.87188	3.44046	10.39614
C	8.60544	2.87085	9.37397
C	6.18544	5.09575	11.25512
C	3.85673	-0.59641	7.74311
C	3.10109	0.47309	8.18555
C	2.16630	0.31944	9.20174
C	2.00077	-0.94429	9.76112
C	2.75382	-2.01555	9.32889
C	5.07731	-8.77673	6.68419
C	5.77605	-9.86066	6.19012
C	6.83413	-9.68765	5.30476
C	7.18132	-8.39285	4.93415
C	6.49510	-7.30303	5.43136
C	7.60899	-10.86642	4.79323
C	1.36994	1.48326	9.71499
C	-3.16904	-1.80036	-13.54515
C	-2.20946	-2.00274	-14.51702
C	-2.23235	-1.28460	-15.70820
C	-3.25012	-0.35761	-15.90074
C	-4.20658	-0.13934	-14.92924
C	-7.70913	-0.24145	-8.06193
C	-8.43203	0.01130	-6.91361
C	-9.50176	-0.79889	-6.54783
C	-9.83500	-1.86638	-7.37390
C	-9.11113	-2.13231	-8.51980
C	-10.29786	-0.50311	-5.31065
C	-1.20830	-1.53605	-16.77576
C	-3.02306	-12.46546	-1.85976
C	-2.16934	-13.51636	-2.12724
C	-0.81034	-13.42256	-1.84661
C	-0.32945	-12.23872	-1.29935
C	-1.17861	-11.18556	-1.02089
C	-7.73509	-6.92705	-0.69834
C	-8.63835	-5.91150	-0.45418
C	-8.70973	-4.80233	-1.28928
C	-7.83176	-4.72843	-2.36546
C	-6.92605	-5.73987	-2.61722
C	-9.68388	-3.69233	-1.02273
C	0.12069	-14.55347	-2.17178
C	1.91362	1.96713	11.06545
C	1.08626	3.11832	11.63256
C	1.55510	3.52966	13.02664
C	0.73816	4.69047	13.59060
C	1.20060	5.08611	14.98825
C	0.66223	-14.41831	-3.60123
C	1.61542	-15.55887	-3.95070
C	2.09050	-15.53192	-5.40505
C	2.93961	-14.31762	-5.78810
C	4.25966	-14.23877	-5.02864
C	8.77542	-11.20113	5.73224
C	9.59279	-12.38598	5.22257
C	10.74212	-12.73122	6.16810
C	11.66271	-13.82884	5.63124
C	10.98711	-15.19288	5.53787
C	-1.19774	2.11589	-19.09137
C	-0.77855	1.22922	-20.26166
C	-1.94475	0.46976	-20.89772
C	-3.02573	1.35014	-21.52830
C	-2.51831	2.18605	-22.69827
C	-2.65795	12.64061	-6.89703
C	-3.16455	13.41400	-5.68139
C	-2.91812	14.92104	-5.77164
C	-3.75914	15.61345	-6.84301
C	-3.56202	17.12535	-6.82239
C	-1.66165	-2.66446	-17.71077
C	-0.62134	-2.95982	-18.78823
C	-1.07542	-4.06503	-19.73914
C	-0.02454	-4.37699	-20.80297
C	-0.48739	-5.47169	-21.75766
C	-11.45667	0.45362	-5.61995
C	-12.24861	0.80965	-4.36401
C	-13.42781	1.73140	-4.66763

C	-14.21060	2.09657	-3.40759	H	1.21498	-15.56331	-6.05936
C	-15.40017	2.99853	-3.71634	H	2.67413	-16.43700	-5.59431
C	7.00515	6.29627	11.74904	H	3.15357	-14.37978	-6.85859
C	6.36589	7.03430	12.92558	H	2.36984	-13.40112	-5.62495
C	5.09248	7.79225	12.55281	H	4.09278	-14.06598	-3.96852
C	4.60158	8.67681	13.69779	H	4.86600	-13.42032	-5.41179
C	3.31994	9.41967	13.33752	H	4.82089	-15.16421	-5.14352
C	-9.00790	-2.52304	-0.29551	H	8.38157	-11.42864	6.72581
C	-9.98529	-1.37898	-0.03624	H	9.42094	-10.32445	5.82740
C	-9.31617	-0.19688	0.66195	H	8.93436	-13.24931	5.11190
C	-10.29548	0.94573	0.92476	H	9.99828	-12.14668	4.23608
C	-9.62075	2.12761	1.61186	H	10.33503	-13.04285	7.13326
C	14.57434	7.64681	-6.47909	H	11.33762	-11.83017	6.33856
C	15.20935	8.39402	-7.65949	H	12.52793	-13.91013	6.29450
C	16.49549	7.71781	-8.12949	H	12.03110	-13.53850	4.64410
C	17.21275	8.48494	-9.24177	H	10.57931	-15.48245	6.50444
C	1.36683	13.52572	-2.24330	H	11.70627	-15.94902	5.22934
C	0.08159	14.12904	-1.68235	H	10.17837	-15.17991	4.81171
C	-0.24450	13.59406	-0.28950	H	-1.82179	2.93787	-19.44643
C	-1.53831	14.18661	0.26577	H	-1.79159	1.53099	-18.38496
C	-1.85497	13.65862	1.66048	H	-0.27499	1.83576	-21.01624
C	13.13477	-0.84059	-0.73727	H	-0.05180	0.49517	-19.90147
C	13.73273	-1.51887	-1.97092	H	-1.54535	-0.19511	-21.66838
C	15.18263	-1.98367	-1.81953	H	-2.41209	-0.15670	-20.13362
C	15.37175	-3.06891	-0.76104	H	-3.82750	0.69741	-21.88453
C	16.80525	-3.58845	-0.73964	H	-3.45646	2.00734	-20.77062
C	16.46279	8.47710	-10.57301	H	-3.34415	2.71630	-23.16858
C	17.26348	9.16560	-11.67295	H	-2.04970	1.54956	-23.44662
H	-3.89210	10.91754	-6.52488	H	-1.79051	2.92268	-22.36755
H	-2.26108	10.79693	-5.84963	H	-3.19950	12.95429	-7.79084
H	0.64493	1.87900	-18.00115	H	-1.59919	12.85967	-7.05642
H	0.60432	3.29866	-19.05647	H	-2.66377	13.02877	-4.78932
H	0.86960	13.83601	-4.31591	H	-4.23529	13.23228	-5.55839
H	1.78892	15.15822	-3.58246	H	-1.85990	15.10457	-5.97271
H	13.85734	1.12472	-1.23984	H	-3.15196	15.37047	-4.80273
H	14.86686	0.26392	-0.06633	H	-4.81471	15.38431	-6.67629
H	5.20963	5.43679	10.90741	H	-3.48676	15.23327	-7.82921
H	6.03785	4.40002	12.08392	H	-3.85514	17.53682	-5.85838
H	8.00485	-10.64682	3.79917	H	-2.51755	17.37606	-6.99744
H	6.95337	-11.73653	4.71565	H	-4.16280	17.60054	-7.59512
H	1.40390	2.30513	8.99715	H	-2.60664	-2.38189	-18.18116
H	0.32600	1.18501	9.84201	H	-1.84547	-3.56652	-17.12203
H	-9.65215	-0.04662	-4.55701	H	-0.42774	-2.05026	-19.36140
H	-10.70325	-1.43037	-4.90015	H	0.31670	-3.25712	-18.31216
H	-0.25590	-1.81412	-16.31837	H	-2.00416	-3.76134	-20.22863
H	-1.05396	-0.63043	-17.36552	H	-1.28673	-4.97136	-19.16579
H	-10.51057	-4.06226	-0.41267	H	0.19403	-3.46970	-21.37148
H	-10.09338	-3.32960	-1.96820	H	0.90121	-4.69099	-20.31421
H	0.95969	-14.55617	-1.47235	H	-1.39830	-5.17031	-22.27124
H	-0.40548	-15.50604	-2.07729	H	-0.69089	-6.39207	-21.21366
H	2.95188	2.28506	10.94152	H	0.27631	-5.67657	-22.50499
H	1.90677	1.13402	11.77205	H	-12.12034	-0.01267	-6.35205
H	1.14986	3.97718	10.95961	H	-11.05786	1.36548	-6.07098
H	0.03665	2.81704	11.68298	H	-12.62005	-0.10696	-3.89858
H	1.47491	2.67296	13.70067	H	-11.58515	1.29975	-3.64698
H	2.60838	3.81646	12.98458	H	-14.09746	1.23916	-5.37747
H	-0.31657	4.40622	13.62541	H	-13.06028	2.64527	-5.14131
H	0.82452	5.54986	12.92127	H	-14.56555	1.18176	-2.92655
H	1.07505	4.25600	15.68075	H	-13.54532	2.60275	-2.70360
H	0.62480	5.93176	15.35819	H	-16.08630	2.50401	-4.40125
H	2.25230	5.36413	14.97799	H	-15.06691	3.92622	-4.17756
H	1.17477	-13.45913	-3.69157	H	-15.94198	3.24327	-2.80516
H	-0.17557	-14.41466	-4.30295	H	7.99254	5.93748	12.05145
H	1.10143	-16.50891	-3.77963	H	7.15057	6.99338	10.92007
H	2.47612	-15.53174	-3.28036	H	6.14561	6.32284	13.72536

H	7.09425	7.75049	13.31573
H	5.28963	8.41768	11.67821
H	4.30349	7.08658	12.28644
H	5.37961	9.40150	13.95132
H	4.42702	8.05998	14.58266
H	2.99488	10.04628	14.16535
H	2.52202	8.71724	13.10610
H	3.47890	10.05496	12.46818
H	-8.59868	-2.87830	0.65343
H	-8.17234	-2.16020	-0.89877
H	-10.40696	-1.04329	-0.98685
H	-10.81132	-1.74165	0.58085
H	-8.88997	-0.53131	1.61123
H	-8.49299	0.16911	0.04282
H	-11.11425	0.58192	1.55048
H	-10.72695	1.27616	-0.02327
H	-8.81637	2.52051	0.99292
H	-9.19832	1.82329	2.56755
H	-10.33686	2.92665	1.79190
H	15.28770	7.61056	-5.65284
H	14.35316	6.62168	-6.78484
H	15.42762	9.42102	-7.35627
H	14.48809	8.43540	-8.47731
H	16.26749	6.70726	-8.47789
H	17.17382	7.62648	-7.27682
H	18.19717	8.03452	-9.39544
H	17.37049	9.51837	-8.92296
H	1.26768	12.43871	-2.29805
H	2.19770	13.74594	-1.56887
H	-0.74769	13.90449	-2.35709
H	0.18285	15.21631	-1.63621
H	-0.33618	12.50569	-0.33243
H	0.57932	13.82799	0.38958
H	-2.36339	13.94549	-0.40910
H	-1.44918	15.27521	0.30215
H	-1.96772	12.57629	1.64263
H	-2.77924	14.09408	2.03430
H	-1.05349	13.90667	2.35368
H	13.16472	-1.52046	0.11574
H	12.08267	-0.62346	-0.94078
H	13.11297	-2.38432	-2.22070
H	13.67828	-0.82267	-2.81224
H	15.82291	-1.13160	-1.58377
H	15.51318	-2.37859	-2.78422
H	14.68904	-3.89691	-0.96851
H	15.12367	-2.67313	0.22529
H	17.07193	-4.01639	-1.70417
H	16.92327	-4.35743	0.02110
H	17.50103	-2.78036	-0.52137
H	16.26071	7.44362	-10.86578
H	15.50385	8.98601	-10.46036
H	18.21776	8.66427	-11.82315
H	17.46062	10.20274	-11.40844
H	16.71581	9.14966	-12.61293
C	-1.35732	-9.49456	7.58878
H	-0.29854	-9.72542	7.49024
H	-1.91926	-10.19523	6.97466
H	-1.64693	-9.63224	8.62839
H	-2.69041	-7.83272	7.27739
H	-1.05248	-7.37657	7.77548
C	-0.53207	-6.17217	1.05586
H	-0.20848	-6.02097	0.02805
H	-0.08322	-5.40224	1.68035
H	-1.61382	-6.06226	1.09685
H	-0.58792	-8.30917	0.90164
H	0.96119	-7.66820	1.46577
C	-8.73984	-9.72531	-7.98194
H	-8.86693	-9.42662	-9.02048
H	-9.66382	-10.18322	-7.63517
H	-7.94563	-10.46760	-7.93348
H	-9.20399	-7.79122	-7.15650
H	-8.26957	-8.84369	-6.08096
C	-3.21469	-4.85387	-8.44698
H	-2.26546	-4.42462	-8.76091
H	-3.18586	-5.01330	-7.37080
H	-4.00639	-4.14068	-8.66738
H	-3.48730	-5.98635	-10.24916
H	-2.64990	-6.86438	-8.96563
C	5.81653	7.10372	-13.07419
H	6.80540	7.42033	-12.74951
H	5.55705	7.66929	-13.96638
H	5.85678	6.04771	-13.33286
H	3.81285	6.97418	-12.28744
H	5.08761	6.76972	-11.08225
C	5.33411	13.72196	-11.84563
H	6.34608	13.38403	-12.06030
H	5.35642	14.79655	-11.67691
H	4.71350	13.51863	-12.71575
H	5.42917	13.22833	-9.76010
H	3.78186	13.37199	-10.39481
C	9.08369	6.47031	-1.96304
H	8.47068	6.01319	-2.73706
H	9.58537	5.68239	-1.40512
H	9.83971	7.08640	-2.44367
H	7.72658	8.10250	-1.60785
H	7.43794	6.68062	-0.59772
C	12.94412	9.17917	2.93524
H	13.60156	9.58556	3.70092
H	13.19799	9.63488	1.98033
H	13.11883	8.10766	2.86212
H	11.25593	8.98472	4.24665
H	11.33056	10.53175	3.38774
H	10.06924	-2.26397	5.42858
H	8.17945	0.35344	8.24993
H	11.17312	-0.36632	4.34594
H	9.04332	6.12355	6.54129
H	9.20406	7.88844	4.83478
H	11.24942	5.23565	2.18272
H	7.48099	10.93015	-0.12488
H	10.76541	9.35518	-2.35895
H	6.53203	11.95846	-2.16072
H	9.85232	10.30576	-8.30786
H	8.40233	10.28745	-10.29895
H	5.10305	11.73068	-7.99620
H	3.29266	8.27557	-14.36547
H	1.27751	9.84944	-10.94411
H	1.27075	7.04805	-15.04545
H	-4.03980	7.13960	-10.24128
H	-5.50838	5.19287	-10.27930
H	-3.78005	3.88398	-13.98217
H	8.92029	6.13721	1.24797
H	10.66696	10.70686	1.05688
H	6.46753	8.65493	-10.41113
H	2.93903	11.64621	-11.98830
H	9.65115	-3.75858	6.99222
H	6.51789	-0.96424	7.81835
H	8.06005	-5.62087	7.07318
H	1.18156	-3.70844	7.84917
H	-0.47481	-5.20567	6.81110
H	2.59369	-7.98877	5.75218

H	-4.03131	-6.81474	3.02293
H	-1.88861	-10.15016	1.43051
H	-5.60547	-6.85901	1.11887
H	-3.70082	-11.18344	-4.25141
H	-4.42884	-10.42687	-6.48439
H	-6.86436	-7.38246	-4.73699
H	-5.10373	-6.14058	-11.80021
H	-7.28653	-4.15982	-8.70559
H	-4.90449	-4.03377	-13.06159
H	-7.99437	1.75332	-10.10935
H	-7.09197	3.92134	-10.78164
H	-4.16286	1.99150	-13.25119
H	1.30531	-6.96478	3.69301
H	-3.10582	-8.64442	4.98413
H	-3.76571	-7.93446	-7.21893
H	-8.11986	-6.71072	-9.08332
H	0.28529	8.72902	-9.19870
H	-0.44781	9.90693	-7.18014
H	-4.23176	10.68226	-8.99207
H	-3.49015	9.52609	-11.03067
H	-1.49240	3.22647	-13.99899
H	-0.74131	1.87147	-15.89545
H	-0.19312	5.41152	-18.21643
H	-0.96103	6.77303	-16.31875
H	4.08795	10.92092	-6.08508
H	2.00177	11.94290	-5.29932
H	4.23386	14.85487	-3.12370
H	6.32852	13.84455	-3.92305
H	9.99838	8.44237	-6.73197
H	12.05397	7.27757	-7.40955
H	14.27876	9.54615	-4.56226
H	12.23025	10.72143	-3.89312
H	13.09102	1.34190	4.14671
H	14.31772	0.27504	2.30342
H	11.72859	2.16514	-0.51263
H	10.48601	3.20915	1.32523
H	7.25006	4.52730	6.76309
H	5.95047	5.53861	8.58831
H	8.04682	3.12734	11.41619
H	9.36028	2.13042	9.59746
H	4.54630	-0.46016	6.92295
H	3.23723	1.44341	7.72809
H	1.27106	-1.08708	10.54600
H	2.63091	-2.98732	9.78548
H	4.26572	-8.92849	7.38132
H	5.49311	-10.85988	6.49111
H	7.99618	-8.23860	4.24035
H	6.75823	-6.30381	5.11613
H	-3.12646	-2.34853	-12.61523
H	-1.42303	-2.72532	-14.34767
H	-3.29107	0.20722	-16.82168
H	-4.99794	0.57600	-15.10197
H	-6.86432	0.38034	-8.32042
H	-8.15614	0.84527	-6.28305
H	-10.66823	-2.50298	-7.11007
H	-9.39224	-2.95836	-9.15714
H	-4.07976	-12.55639	-2.06753
H	-2.56246	-14.42755	-2.55661
H	0.72609	-12.14139	-1.08618
H	-0.77808	-10.26513	-0.62262
H	-7.70665	-7.79565	-0.05579
H	-9.30631	-5.98443	0.39300
H	-7.86220	-3.86741	-3.01897
H	-6.23336	-5.64709	-3.44092

*Table 14 Cartesian coordinates of **1n** (GFN2-xTB(GBSA(toluene)). B97-3c(COSMO(toluene) single point energy = 10838.5464948 Hartree.*

	X	Y	Z
C	10.39302	-0.83717	5.39301
C	9.54409	-0.69312	6.49307
C	9.31100	0.59635	6.99807
C	9.84534	1.68964	6.35281
C	10.64656	1.52230	5.19353
C	10.95322	0.25410	4.74500
C	10.84588	2.83900	4.63921
C	10.14163	3.78503	5.49880
C	11.27141	3.45519	3.49962
C	10.20363	5.07597	4.84234
C	9.55900	3.12177	6.54037
C	9.72317	6.33305	5.15114
C	9.80480	7.34171	4.19472
C	10.39923	7.12240	2.95778
C	10.98038	5.87894	2.68397
C	10.86253	4.86063	3.60180
C	10.04640	8.96955	-2.98777
C	9.51186	9.78389	-1.99195
C	8.70344	10.86778	-2.34880
C	8.34397	11.02881	-3.67165
C	8.78344	10.10390	-4.65481
C	9.69250	9.12077	-4.32191
C	8.04314	10.40356	-5.85937
C	7.20852	11.56568	-5.57567
C	7.77368	9.91838	-7.10522
C	6.40146	11.80467	-6.75478
C	7.38368	11.96174	-4.28082
C	5.41580	12.71869	-7.06981
C	4.66788	12.52726	-8.22660
C	4.89893	11.44885	-9.07496
C	5.96684	10.58404	-8.81232
C	6.71818	10.76760	-7.67206
C	-0.32264	9.75587	-12.88054
C	0.97905	9.31692	-12.66414
C	1.38324	8.07807	-13.17755
C	0.44979	7.25086	-13.76344
C	-0.90712	7.66176	-13.86192
C	-1.27433	8.93424	-13.47512
C	-1.65013	6.50691	-14.31651
C	-0.68253	5.44779	-14.57257
C	-2.91755	6.00194	-14.33417
C	-1.43177	4.23381	-14.80656
C	0.57586	5.87133	-14.25585
C	-1.06155	2.92995	-15.06513
C	-2.03263	1.93720	-15.02031
C	-3.36139	2.23147	-14.72459
C	-3.75467	3.56793	-14.56887
C	-2.80020	4.56058	-14.60109
C	10.25627	8.06637	1.83105
C	8.95467	8.31884	1.40715
C	8.66662	8.94994	0.20881
C	9.73707	9.36552	-0.59265
C	11.03413	9.21171	-0.11575
C	11.32116	8.56507	1.07881
C	3.91177	11.08312	-10.11124
C	2.62909	10.80367	-9.64674
C	1.63536	10.27776	-10.45332
C	1.93276	10.04618	-11.80296
C	3.19242	10.38669	-12.28144
C	4.19518	10.89314	-11.46369

C	0.34139	9.85964	-9.80715
C	5.54205	11.21896	-12.05196
C	12.75116	8.42203	1.52931
C	7.23014	9.02184	-0.23552
C	9.19552	-3.15920	6.74222
C	8.76393	-1.84849	6.95587
C	7.47432	-1.64628	7.47396
C	6.61468	-2.71224	7.60673
C	7.02339	-4.01862	7.22703
C	8.33855	-4.24379	6.87186
C	5.83805	-4.84355	7.27126
C	4.76747	-4.02565	7.83270
C	5.33374	-6.02574	6.81194
C	3.54358	-4.78858	7.72362
C	5.20588	-2.74814	8.02758
C	2.22122	-4.51243	8.00837
C	1.23863	-5.36277	7.51571
C	1.55351	-6.46941	6.72862
C	2.89919	-6.80526	6.53338
C	3.88368	-5.98911	7.04631
C	-0.90883	-8.44055	1.45322
C	-1.75483	-8.46643	2.55910
C	-3.11411	-8.74262	2.39672
C	-3.61531	-8.89616	1.11728
C	-2.74892	-8.79937	-0.00357
C	-1.39386	-8.60998	0.16470
C	-3.58669	-8.83657	-1.17791
C	-4.95773	-9.03842	-0.72792
C	-3.55862	-8.51101	-2.50044
C	-5.80863	-8.88478	-1.89136
C	-4.99577	-9.07437	0.63715
C	-7.16521	-8.98575	-2.12164
C	-7.67822	-8.61877	-3.36476
C	-6.86242	-8.12038	-4.37745
C	-5.47263	-8.11625	-4.17077
C	-4.95290	-8.49885	-2.95867
C	-7.60078	-3.32992	-8.16847
C	-7.50713	-4.59374	-8.75788
C	-7.26164	-4.67733	-10.13564
C	-7.00048	-3.52390	-10.84937
C	-7.03555	-2.25757	-10.21262
C	-7.39258	-2.16134	-8.88395
C	-6.54641	-1.29689	-11.17099
C	-6.23651	0.02259	-11.30534
C	-5.62194	-1.07289	-13.30510
C	-5.03228	-1.16443	-14.55141
C	-4.35395	-0.06204	-15.06402
C	-4.27532	1.13774	-14.36305
C	-4.96344	1.26434	-13.14908
C	-5.62253	0.17615	-12.62781
C	0.52184	-7.11612	5.89672
C	-0.29377	-6.24844	5.17042
C	-1.12416	-6.67908	4.15325
C	-1.13723	-8.04192	3.83899
C	-0.41706	-8.92183	4.63335
C	0.40663	-8.49062	5.66766
C	-7.32667	-7.46323	-5.61271
C	-6.56257	-7.64163	-6.76971
C	-6.62387	-6.80831	-7.87321
C	-7.54995	-5.74991	-7.84354
C	-8.43348	-5.69004	-6.76731
C	-8.34038	-6.49726	-5.64409
C	-5.66297	-6.99468	-9.02192
C	-9.29562	-6.22769	-4.51352
C	1.12543	-9.51278	6.50927

C	-1.97337	-5.67894	3.41772
C	1.81814	5.12338	-14.36471
C	-4.15926	6.72055	-14.09499
C	-6.41032	1.11626	-10.36169
C	-6.30155	-4.45654	-13.15260
C	-2.39863	-8.23907	-3.33429
C	-6.17415	-9.20995	1.47838
C	6.01912	-7.11647	6.13893
C	4.46628	-1.61248	8.55404
C	11.95126	2.87004	2.35564
C	8.69987	3.63071	7.59758
C	8.34460	8.76334	-7.77878
C	6.73364	13.04164	-3.55582
C	-6.50153	-3.35476	-12.22489
C	-6.20888	-2.03008	-12.38730
C	2.73928	5.06489	-13.31940
C	3.91431	4.35213	-13.45883
C	4.21144	3.68222	-14.64038
C	3.28981	3.73690	-15.68033
C	2.10862	4.43845	-15.54571
C	5.51317	2.95400	-14.80537
C	-4.41258	7.91423	-14.77175
C	-5.58356	8.61203	-14.55378
C	-6.53149	8.15142	-13.64653
C	-6.27314	6.96724	-12.96581
C	-5.10916	6.25702	-13.18572
C	-7.81963	8.89279	-13.43916
C	8.49490	7.54971	-7.10722
C	9.05658	6.46137	-7.74292
C	9.48734	6.54593	-9.06324
C	9.32024	7.75013	-9.73667
C	8.75486	8.84364	-9.10914
C	5.35456	13.23046	-3.64376
C	4.73989	14.25200	-2.94765
C	5.47717	15.11673	-2.14608
C	6.85169	14.92638	-2.05796
C	7.47341	13.90143	-2.74365
C	10.08643	5.34958	-9.74286
C	13.06509	2.04924	2.53263
C	13.71642	1.50301	1.44370
C	13.27484	1.75031	0.14876
C	12.15313	2.55373	-0.02486
C	11.50098	3.11005	1.05697
C	13.95215	1.13159	-1.03874
C	8.86759	3.21406	8.91871
C	8.02184	3.66944	9.91042
C	6.97970	4.54303	9.61850
C	6.81526	4.95896	8.30211
C	7.66038	4.51333	7.30497
C	6.08541	5.05489	10.70942
C	3.71695	-1.75386	9.72329
C	3.04277	-0.67635	10.26140
C	3.08794	0.57320	9.65152
C	3.81569	0.70787	8.47505
C	4.49633	-0.36451	7.93186
C	6.94588	-6.86229	5.12774
C	7.58432	-7.90288	4.48369
C	7.32721	-9.22528	4.83015
C	6.40712	-9.47684	5.84137
C	5.75314	-8.44236	6.48146
C	8.06043	-10.34897	4.15827
C	2.32884	1.73396	10.22442
C	-5.97435	1.00430	-9.04233
C	-6.14287	2.05034	-8.15644
C	-6.75537	3.23347	-8.55427

C	-7.18811	3.34319	-9.87096
C	-7.01040	2.30810	-10.76746
C	-5.09495	-4.59359	-13.83924
C	-4.89624	-5.64970	-14.70541
C	-5.89241	-6.59619	-14.92096
C	-7.09434	-6.45853	-14.23612
C	-7.29824	-5.40978	-13.36048
C	-5.65244	-7.76234	-15.83440
C	-6.98406	4.34821	-7.57597
C	-7.09824	-10.22502	1.22731
C	-8.23463	-10.34668	2.00009
C	-8.49817	-9.45071	3.03206
C	-7.57469	-8.44306	3.28282
C	-6.42471	-8.32542	2.52553
C	-1.31956	-9.12408	-3.32616
C	-0.22436	-8.90571	-4.13753
C	-0.16343	-7.79506	-4.97240
C	-1.22879	-6.90222	-4.96254
C	-2.33258	-7.11774	-4.16016
C	0.99859	-7.59665	-5.90073
C	-9.76999	-9.55822	3.82325
C	0.86849	1.71636	9.75420
C	0.07866	2.90146	10.30455
C	-1.38079	2.87692	9.85494
C	-2.17116	4.06672	10.39633
C	-3.62819	4.03421	9.94907
C	-10.99576	-9.19012	2.97557
C	-10.95169	-7.74430	2.48630
C	-12.15469	-7.40108	1.61014
C	-12.10438	-5.96294	1.09778
C	-13.30349	-5.62873	0.21736
C	9.39099	-10.62900	4.86936
C	10.17069	-11.75676	4.19734
C	11.49531	-12.03121	4.90753
C	12.35797	-13.07982	4.20264
C	11.76028	-14.48225	4.24491
C	-8.89491	8.39668	-14.41494
C	-10.21521	9.14181	-14.23418
C	-11.29052	8.64752	-15.19975
C	-12.61029	9.39652	-15.02456
C	-13.68018	8.90063	-15.99081
C	6.60825	3.90764	-15.30173
C	7.93282	3.18494	-15.53494
C	9.03230	4.13855	-15.99836
C	10.35856	3.41847	-16.23585
C	11.45157	4.37647	-16.69691
C	-8.34941	4.18839	-6.89335
C	-8.69384	5.34747	-5.95739
C	-7.79163	5.41549	-4.72553
C	-8.27981	6.41661	-3.67669
C	-8.20505	7.86564	-4.14653
C	-4.98393	-8.91693	-15.07618
C	-4.70206	-10.10921	-15.98726
C	-4.02762	-11.25720	-15.23859
C	-3.73573	-12.44709	-16.15094
C	-3.05864	-13.58870	-15.40074
C	6.72886	6.25079	11.42334
C	5.84846	6.78284	12.55159
C	6.49129	7.96817	13.26881
C	5.61574	8.50232	14.40089
C	6.26512	9.68285	15.11419
C	0.74748	-8.31970	-7.23086
C	1.90606	-8.14246	-8.20911
C	1.65531	-8.87152	-9.52772
C	2.80596	-8.69132	-10.51607

C	2.55078	-9.42691	-11.82687
C	4.81234	16.25724	-1.43291
C	4.82082	17.51761	-2.30801
C	4.12387	18.69213	-1.62583
C	4.14555	19.95113	-2.49037
C	8.98920	4.41225	-10.26427
C	9.53939	3.18667	-10.99519
C	10.27191	2.20955	-10.07638
C	10.75653	0.95134	-10.80120
C	9.62708	0.00471	-11.19447
C	13.28205	-0.19975	-1.40554
C	13.85874	-0.83386	-2.67259
C	13.44139	-0.10923	-3.95147
C	13.96782	-0.80484	-5.20549
C	13.52890	-0.08910	-6.47828
C	3.43153	21.12360	-1.82053
C	3.46063	22.37817	-2.68627
H	5.39476	2.14147	-15.52554
H	5.82120	2.52205	-13.85060
H	-7.66233	9.96206	-13.59689
H	-8.17282	8.74695	-12.41594
H	10.71527	4.81119	-9.03206
H	10.70976	5.66999	-10.58031
H	15.00668	0.95223	-0.81927
H	13.88856	1.81527	-1.88644
H	5.12621	5.36569	10.29033
H	5.90021	4.26246	11.43831
H	8.26182	-10.08965	3.11635
H	7.44922	-11.25394	4.17321
H	2.35127	1.69076	11.31565
H	2.79322	2.67029	9.90915
H	-5.00537	-7.45861	-16.66047
H	-6.59940	-8.11019	-16.25252
H	-6.95613	5.30920	-8.09456
H	-6.19615	4.34042	-6.82180
H	1.91066	-7.99041	-5.44686
H	1.14483	-6.53181	-6.09430
H	-9.72149	-8.89799	4.69153
H	-9.88913	-10.58403	4.18241
H	0.39921	0.78378	10.07617
H	0.84439	1.73638	8.66181
H	0.11919	2.88737	11.39668
H	0.54341	3.83210	9.96932
H	-1.42229	2.88389	8.76273
H	-1.84796	1.94957	10.19596
H	-1.70729	4.99429	10.05175
H	-2.12777	4.06154	11.48832
H	-3.69486	4.05813	8.86308
H	-4.16981	4.89048	10.34539
H	-4.11533	3.12694	10.30097
H	-11.89816	-9.34397	3.57228
H	-11.04991	-9.85717	2.11243
H	-10.03357	-7.58577	1.91503
H	-10.92638	-7.07038	3.34641
H	-13.07434	-7.54814	2.18244
H	-12.18438	-8.08392	0.75705
H	-12.07764	-5.27749	1.94858
H	-11.18353	-5.81682	0.52740
H	-14.23088	-5.74399	0.77498
H	-13.33979	-6.29082	-0.64566
H	-13.24252	-4.60270	-0.13956
H	9.19153	-10.89241	5.91104
H	9.99370	-9.71740	4.87064
H	9.55802	-12.65987	4.19661
H	10.37007	-11.48751	3.15681

H	11.29633	-12.35823	5.93118
H	12.06071	-11.09704	4.96554
H	13.33887	-13.10180	4.68473
H	12.50927	-12.78130	3.16229
H	11.56768	-14.78263	5.27308
H	12.44920	-15.19891	3.80223
H	10.82577	-14.52628	3.69144
H	-8.53754	8.52957	-15.43898
H	-9.05446	7.32701	-14.25887
H	-10.05167	10.21064	-14.39387
H	-10.56625	9.01207	-13.20721
H	-10.93889	8.77335	-16.22693
H	-11.45820	7.57966	-15.03789
H	-12.44286	10.46425	-15.18687
H	-12.96309	9.27001	-13.99794
H	-13.35617	9.03500	-17.02102
H	-14.60877	9.44945	-15.84865
H	-13.87860	7.84262	-15.83067
H	6.27948	4.37506	-16.23328
H	6.74998	4.70333	-14.56633
H	7.79115	2.40447	-16.28700
H	8.24665	2.69668	-14.60956
H	8.71867	4.62882	-16.92360
H	9.17517	4.91770	-15.24514
H	10.21682	2.64067	-16.99057
H	10.67321	2.92682	-15.31198
H	11.16951	4.85590	-17.63242
H	12.38719	3.84360	-16.85338
H	11.61893	5.15292	-15.95272
H	-9.11824	4.11866	-7.66759
H	-8.36188	3.24975	-6.33389
H	-9.72878	5.22668	-5.62607
H	-8.62995	6.28372	-6.51471
H	-6.77687	5.68609	-5.02360
H	-7.75121	4.42392	-4.26630
H	-7.66521	6.30584	-2.77927
H	-9.31008	6.17771	-3.40089
H	-8.47867	8.53884	-3.33661
H	-8.88388	8.04256	-4.97687
H	-7.19428	8.10983	-4.46799
H	-4.04723	-8.56272	-14.63893
H	-5.63314	-9.22955	-14.25469
H	-5.64073	-10.46337	-16.42089
H	-4.05852	-9.78984	-16.81106
H	-4.67243	-11.58188	-14.41802
H	-3.09133	-10.90179	-14.80077
H	-4.67154	-12.80505	-16.58731
H	-3.09189	-12.12190	-16.97201
H	-2.85850	-14.42169	-16.07131
H	-2.11422	-13.25721	-14.97345
H	-3.69367	-13.94382	-14.59143
H	6.91119	7.04565	10.69583
H	7.69718	5.94767	11.82847
H	4.88200	7.08926	12.14337
H	5.66311	5.98277	13.27279
H	7.45831	7.66201	13.67593
H	6.67626	8.76903	12.54839
H	5.42995	7.70177	15.12127
H	4.64963	8.81163	13.99437
H	7.22079	9.38981	15.54455
H	6.44086	10.50019	14.41747
H	5.62483	10.04617	15.91528
H	-0.17053	-7.93292	-7.67976
H	0.59446	-9.38379	-7.03512
H	2.05248	-7.07761	-8.40744
H	2.82483	-8.52272	-7.75549
H	0.73259	-8.49585	-9.97718
H	1.51474	-9.93713	-9.32945
H	2.94329	-7.62628	-10.71897
H	3.72964	-9.06334	-10.06593
H	1.64212	-9.05916	-12.29954
H	2.43543	-10.49439	-11.64928
H	3.37982	-9.28094	-12.51606
H	5.33565	16.46844	-0.49779
H	3.77908	15.99538	-1.19485
H	4.32224	17.29794	-3.25529
H	5.85472	17.78803	-2.53585
H	3.08728	18.42099	-1.40980
H	4.61558	18.90083	-0.67230
H	3.66631	19.73860	-3.44944
H	5.18216	20.23049	-2.69527
H	8.34982	4.97591	-10.94920
H	8.36672	4.08742	-9.42700
H	10.21814	3.51369	-11.78666
H	8.70113	2.67072	-11.46668
H	11.14076	2.70744	-9.64121
H	9.61003	1.91847	-9.25657
H	11.31470	1.24306	-11.69443
H	11.44622	0.41557	-10.14375
H	8.95249	0.47012	-11.90851
H	9.05145	-0.28582	-10.31753
H	10.03212	-0.89592	-11.65153
H	12.20978	-0.03646	-1.53899
H	13.40612	-0.89148	-0.56855
H	14.94917	-0.85495	-2.60441
H	13.51306	-1.86931	-2.73170
H	13.81414	0.91677	-3.93655
H	12.35003	-0.06579	-3.99762
H	15.05944	-0.84026	-5.16721
H	13.60494	-1.83560	-5.22669
H	12.44294	-0.06354	-6.54577
H	13.89600	0.93545	-6.48699
H	13.91584	-0.59940	-7.35777
H	3.90829	21.33504	-0.86014
H	2.39366	20.84618	-1.61992
H	2.97461	22.19356	-3.64234
H	4.48670	22.68566	-2.87867
H	2.94379	23.19743	-2.19091
C	2.12074	-10.35609	5.71003
H	2.88397	-9.73016	5.25228
H	1.61659	-10.90411	4.91773
H	2.61113	-11.07360	6.36461
H	0.37848	-10.18238	6.94748
H	1.64369	-9.01295	7.32916
C	-3.29059	-5.44727	4.16173
H	-3.90758	-4.72790	3.62714
H	-3.09873	-5.06638	5.16273
H	-3.84164	-6.38049	4.25245
H	-2.18613	-6.03203	2.40715
H	-1.43549	-4.73098	3.34087
C	-10.61948	-6.96791	-4.72068
H	-11.09831	-6.63200	-5.63828
H	-11.29385	-6.77881	-3.88794
H	-10.45723	-8.04080	-4.80183
H	-9.50235	-5.15502	-4.47126
H	-8.84980	-6.51729	-3.56127
C	-4.39739	-7.78752	-8.70441
H	-3.72171	-7.73913	-9.55648
H	-4.61489	-8.83554	-8.51220
H	-3.88728	-7.36919	-7.83967

H	-5.36477	-6.00731	-9.38391
H	-6.18653	-7.49794	-9.84168
C	0.38855	8.37779	-9.42699
H	1.22987	8.18037	-8.76535
H	0.49845	7.76395	-10.31847
H	-0.52964	8.08748	-8.92068
H	-0.50552	10.02264	-10.47111
H	0.18541	10.45528	-8.90483
C	6.28036	9.96774	-12.53197
H	6.41991	9.26564	-11.71281
H	7.25791	10.23657	-12.92716
H	5.71889	9.46485	-13.31599
H	6.14924	11.73846	-11.30824
H	5.40338	11.89262	-12.90276
C	6.87570	7.81063	-1.10155
H	5.82969	7.85067	-1.39834
H	7.04789	6.88684	-0.55256
H	7.48944	7.79716	-1.99978
H	7.03811	9.92938	-0.80584
H	6.58428	9.03247	0.64553
C	13.59035	7.56190	0.58257
H	14.61166	7.48915	0.95066
H	13.61459	7.99384	-0.41513
H	13.18310	6.55600	0.50789
H	12.77601	7.98827	2.53082
H	13.20018	9.41856	1.58433
H	10.56854	-1.82031	4.98342
H	8.67829	0.73726	7.86149
H	11.56361	0.10978	3.86562
H	9.25827	6.52922	6.10635
H	9.38090	8.31295	4.40882
H	11.45996	5.71560	1.73141
H	10.70334	8.16037	-2.70056
H	8.34476	11.54862	-1.58988
H	10.09000	8.45231	-5.07110
H	5.19567	13.54745	-6.41309
H	3.86111	13.20930	-8.45639
H	6.14735	9.74075	-9.46202
H	-0.60507	10.75059	-12.56499
H	2.40977	7.76557	-13.05428
H	-2.29238	9.27612	-13.58920
H	-0.02789	2.67229	-15.24304
H	-1.74102	0.90478	-15.15059
H	-4.79498	3.80126	-14.39494
H	8.13789	7.94356	2.00961
H	11.85008	9.58522	-0.71958
H	2.42220	10.95548	-8.59558
H	3.40110	10.23462	-13.33196
H	10.21775	-3.34162	6.44528
H	7.13568	-0.65015	7.70958
H	8.69349	-5.24327	6.66659
H	1.94535	-3.62075	8.55222
H	0.19969	-5.13241	7.70524
H	3.15966	-7.64425	5.90719
H	0.14211	-8.23922	1.60840
H	-3.75927	-8.79644	3.26173
H	-0.73046	-8.54901	-0.68512
H	-7.83546	-9.31975	-1.34283
H	-8.73731	-8.73038	-3.53528
H	-4.81835	-7.76106	-4.95131
H	-7.78549	-3.25872	-7.10669
H	-7.25676	-5.63473	-10.63141
H	-7.46204	-1.20180	-8.39387
H	-5.05906	-2.08789	-15.11112
H	-3.85555	-0.14698	-16.01944
H	-4.90318	2.18687	-12.59025
H	-0.22191	-5.18726	5.36550
H	-0.47953	-9.97966	4.41725
H	-5.84872	-8.44997	-6.76956
H	-9.20306	-4.93019	-6.78027
H	2.51515	5.55258	-12.38187
H	4.61215	4.31029	-12.63400
H	3.50091	3.21978	-16.60618
H	1.40494	4.48271	-16.36489
H	-3.68707	8.27777	-15.48564
H	-5.76521	9.53187	-15.09232
H	-6.99370	6.60038	-12.24858
H	-4.91242	5.35626	-12.62303
H	8.14770	7.46498	-6.08775
H	9.16925	5.52959	-7.20593
H	9.64463	7.83536	-10.76461
H	8.65756	9.78031	-9.63861
H	4.76734	12.55066	-4.24391
H	3.66864	14.37878	-3.02195
H	7.44164	15.58910	-1.44005
H	8.54486	13.77819	-2.67630
H	13.42811	1.86341	3.53352
H	14.58510	0.87843	1.59995
H	11.78786	2.74716	-1.02403
H	10.61617	3.71046	0.90190
H	9.68204	2.54825	9.16631
H	8.16716	3.33786	10.92915
H	6.00890	5.63554	8.05419
H	7.50480	4.82224	6.28154
H	3.69289	-2.71417	10.21865
H	2.47762	-0.80201	11.17458
H	3.85456	1.66803	7.97921
H	5.03098	-0.24404	7.00076
H	7.13909	-5.84046	4.83517
H	8.29107	-7.68783	3.69410
H	6.19738	-10.49834	6.12643
H	5.04942	-8.65454	7.27319
H	-5.47740	0.09847	-8.72628
H	-5.79060	1.94937	-7.13911
H	-7.66941	4.25464	-10.19650
H	-7.36034	2.40591	-11.78544
H	-4.30640	-3.87573	-13.66576
H	-3.95286	-5.74359	-15.22535
H	-7.88237	-7.18197	-14.39340
H	-8.24685	-5.30599	-12.85344
H	-6.90217	-10.92832	0.43027
H	-8.93269	-11.14746	1.79935
H	-7.76094	-7.73566	4.07907
H	-5.73279	-7.51803	2.71554
H	-1.36263	-9.99931	-2.69345
H	0.59765	-9.60802	-4.12540
H	-1.19296	-6.02459	-5.59328
H	-3.13792	-6.39736	-4.14832

*Table 15 Cartesian coordinates of **1c** (GFN2-xTB(GBSA(toluene)). B97-3c(COSMO(toluene) single point energy = 10838.35974765 Hartree. (Used for the calculation of association free energies)*

	X	Y	Z
C	10.68843	-0.96208	6.11030
C	9.63169	-0.92517	7.02098
C	9.19423	0.31868	7.50189
C	9.74331	1.47530	6.99524

C	10.75647	1.41770	6.00271
C	11.25945	0.19661	5.60146
C	10.92023	2.76472	5.50975
C	9.98155	3.61185	6.23710
C	11.47689	3.45217	4.47125
C	10.01986	4.91627	5.60866
C	9.29412	2.86770	7.15218
C	9.35565	6.10386	5.83911
C	9.47307	7.13379	4.91112
C	10.27060	7.00328	3.77905
C	11.03227	5.84149	3.60330
C	10.89747	4.80138	4.49619
C	8.17722	11.37228	-0.47765
C	9.17608	10.42702	-0.69264
C	9.65735	10.22307	-1.99051
C	9.04473	10.86295	-3.04550
C	7.93525	11.72113	-2.82623
C	7.55361	12.02674	-1.53477
C	7.37630	12.00216	-4.13221
C	8.21864	11.32446	-5.11407
C	6.29073	12.55313	-4.74824
C	7.59253	11.49561	-6.40619
C	9.22733	10.65446	-4.48758
C	7.88361	11.03100	-7.67249
C	6.92378	11.15701	-8.66999
C	5.68745	11.74785	-8.42392
C	5.44220	12.32533	-7.17074
C	6.38700	12.20893	-6.17463
C	0.09524	10.51221	-11.88487
C	1.41361	10.07803	-11.83534
C	1.77822	8.88754	-12.47470
C	0.79845	8.09222	-13.02824
C	-0.56043	8.50923	-13.00484
C	-0.89789	9.74080	-12.48200
C	-1.34096	7.38392	-13.47376
C	-0.40080	6.32214	-13.80542
C	-2.61827	6.90215	-13.48124
C	-1.17272	5.12100	-14.01937
C	0.88077	6.72991	-13.57203
C	-0.82273	3.80855	-14.25978
C	-1.80232	2.82892	-14.17056
C	-3.13101	3.13890	-13.88028
C	-3.50227	4.48769	-13.75632
C	-2.53180	5.46470	-13.78566
C	10.16165	7.95754	2.65724
C	9.71523	7.40949	1.45842
C	9.43794	8.16949	0.33528
C	9.60592	9.55765	0.41991
C	10.08375	10.10367	1.60704
C	10.37936	9.33459	2.72513
C	4.56886	11.55902	-9.36769
C	3.42532	10.97537	-8.82318
C	2.35782	10.55435	-9.59366
C	2.43570	10.72358	-10.98122
C	3.53593	11.37459	-11.51960
C	4.60648	11.80423	-10.74217
C	1.21201	9.84455	-8.92415
C	5.74928	12.52978	-11.40243
C	10.98644	9.99009	3.93497
C	8.92168	7.45377	-0.88559
C	9.44859	-3.41664	7.26261
C	8.86973	-2.14805	7.30441
C	7.47811	-2.04782	7.45300
C	6.71036	-3.18834	7.48381
C	7.30355	-4.46629	7.31210
C	8.67917	-4.57424	7.25150
C	6.21042	-5.39108	7.10750
C	4.97081	-4.62807	7.21253
C	5.95149	-6.64751	6.63991
C	3.89281	-5.52119	6.84986
C	5.24639	-3.31575	7.46287
C	2.52291	-5.37052	6.78409
C	1.76613	-6.37820	6.19909
C	2.35845	-7.53665	5.70454
C	3.73284	-7.73855	5.87313
C	4.49845	-6.73797	6.43437
C	-0.56398	-11.09802	0.85006
C	-0.87458	-10.08023	1.74399
C	-2.04332	-9.33134	1.56021
C	-2.79740	-9.52413	0.42370
C	-2.41420	-10.50147	-0.53475
C	-1.33016	-11.31939	-0.29044
C	-3.28462	-10.31577	-1.67656
C	-4.21935	-9.25340	-1.33153
C	-3.37737	-10.61083	-3.00638
C	-4.92928	-8.90025	-2.53973
C	-3.95977	-8.77923	-0.07908
C	-5.85932	-7.92325	-2.82775
C	-6.19292	-7.68978	-4.15618
C	-5.63013	-8.43477	-5.18886
C	-4.74993	-9.48180	-4.88941
C	-4.38477	-9.70311	-3.57809
C	-5.24167	-5.89861	-11.38193
C	-5.81999	-5.62322	-10.14886
C	-6.33573	-4.34610	-9.89708
C	-6.14187	-3.34439	-10.82239
C	-5.46413	-3.61484	-12.04305
C	-5.06568	-4.90252	-12.33847
C	-5.25896	-2.33480	-12.68905
C	-4.50796	-1.73192	-13.65744
C	-5.47420	-0.03705	-12.33857
C	-5.68075	1.24927	-11.88626
C	-4.95750	2.28236	-12.46404
C	-4.05662	2.06078	-13.50629
C	-3.92891	0.76472	-14.03121
C	-4.60213	-0.27928	-13.43387
C	1.57624	-8.41997	4.81607
C	2.01839	-8.48419	3.49732
C	1.28375	-9.08108	2.48795
C	0.04505	-9.64544	2.81676
C	-0.36241	-9.64821	4.14524
C	0.38102	-9.05382	5.15731
C	-5.77634	-7.95568	-6.57788
C	-4.59355	-7.58679	-7.21420
C	-4.56962	-6.90389	-8.41729
C	-5.79224	-6.56915	-9.01238
C	-6.97039	-7.00734	-8.42108
C	-6.99037	-7.70627	-7.22009
C	-3.24356	-6.43535	-8.95344
C	-8.30074	-8.21136	-6.68078
C	-0.08748	-9.15706	6.58265
C	1.78484	-8.96932	1.07289
C	2.10381	5.97420	-13.78803
C	-3.84002	7.63615	-13.19512
C	-3.71434	-2.37626	-14.69122
C	-7.14066	-1.23794	-9.65625
C	-2.60681	-11.59870	-3.74449
C	-4.66976	-7.73392	0.64081
C	6.91401	-7.70271	6.36848
C	4.35631	-2.17882	7.62991

C	12.43305	2.95916	3.49357
C	8.23785	3.26999	8.06740
C	5.17607	13.28847	-4.17222
C	10.26069	9.79739	-5.04781
C	-6.43007	-1.90761	-10.73322
C	-5.88047	-1.32838	-11.83981
C	3.10220	5.89162	-12.81814
C	4.25833	5.17557	-13.06078
C	4.46117	4.52907	-14.27450
C	3.45906	4.60071	-15.23631
C	2.29649	5.30566	-14.99838
C	5.70930	3.73361	-14.52279
C	-4.06673	8.87108	-13.80448
C	-5.22094	9.58231	-13.54514
C	-6.17839	9.09457	-12.66215
C	-5.94520	7.87093	-12.04493
C	-4.79726	7.14856	-12.30612
C	-7.45104	9.85050	-12.41629
C	4.52389	12.82282	-3.03087
C	3.45569	13.51824	-2.50036
C	3.00857	14.69844	-3.08473
C	3.66108	15.16227	-4.22133
C	4.72282	14.46662	-4.76558
C	9.93804	8.80499	-5.97273
C	10.91628	7.97091	-6.47616
C	12.24278	8.10232	-6.07949
C	12.56336	9.09574	-5.16131
C	11.58905	9.92680	-4.64358
C	1.87740	15.47442	-2.47645
C	13.56550	2.26024	3.91387
C	14.47999	1.78608	2.99493
C	14.29157	1.98269	1.63091
C	13.15583	2.66656	1.21275
C	12.24162	3.15363	2.12620
C	15.26184	1.42129	0.63331
C	7.18263	4.07235	7.63438
C	6.17470	4.43713	8.50488
C	6.18757	4.01914	9.83096
C	7.23781	3.21495	10.26024
C	8.24497	2.83793	9.39414
C	5.12212	4.46430	10.78965
C	3.27836	-1.96585	6.77182
C	2.46920	-0.85641	6.92312
C	2.70816	0.07287	7.92909
C	3.77861	-0.14503	8.78987
C	4.59393	-1.24872	8.64369
C	7.89533	-8.00805	7.31272
C	8.82479	-8.99854	7.06682
C	8.81563	-9.70638	5.86976
C	7.84087	-9.39799	4.92762
C	6.90070	-8.41587	5.17018
C	9.80925	-10.80162	5.61520
C	1.81599	1.26506	8.11451
C	-2.39039	-2.00904	-14.92978
C	-1.65203	-2.64266	-15.90997
C	-2.20781	-3.65446	-16.68462
C	-3.52773	-4.02118	-16.44489
C	-4.26965	-3.39949	-15.46060
C	-6.84177	-1.47072	-8.31439
C	-7.51856	-0.79417	-7.31820
C	-8.51636	0.12464	-7.62377
C	-8.81924	0.34984	-8.96209
C	-8.13943	-0.31261	-9.96420
C	-9.27235	0.82800	-6.53497
C	-1.41518	-4.30787	-17.77839
C	-6.06308	-7.77520	0.71959
C	-6.75755	-6.80566	1.41337
C	-6.09079	-5.75467	2.03604
C	-4.70520	-5.70642	1.94330
C	-4.00055	-6.68166	1.26312
C	-2.47208	-12.89235	-3.23829
C	-1.73354	-13.84037	-3.91724
C	-1.09526	-13.52816	-5.11289
C	-1.22572	-12.23799	-5.61371
C	-1.97115	-11.28619	-4.94547
C	-0.31522	-14.56960	-5.86035
C	-6.86215	-4.68990	2.76152
C	0.72615	0.97409	9.15457
C	-0.15975	2.19124	9.40993
C	-1.25020	1.89992	10.43902
C	-2.13212	3.11791	10.70792
C	-3.21957	2.81836	11.73349
C	-7.61380	-3.77041	1.78934
C	-6.66814	-2.99017	0.87957
C	-7.42642	-2.10620	-0.10777
C	-6.48577	-1.33357	-1.03023
C	-7.25099	-0.47858	-2.03363
C	9.25825	-12.15664	6.07857
C	10.24846	-13.29074	5.82338
C	9.69169	-14.64082	6.27213
C	10.69023	-15.79056	6.12532
C	11.01812	-16.12507	4.67392
C	-8.53265	9.42827	-13.41948
C	-9.83789	10.19131	-13.20692
C	-10.91840	9.76478	-14.19872
C	-12.22473	10.52965	-13.99407
C	-13.30034	10.09446	-14.98303
C	5.51139	2.26753	-14.11544
C	6.77747	1.44125	-14.32947
C	6.58448	-0.01972	-13.92774
C	7.85436	-0.84525	-14.12678
C	7.65725	-2.30139	-13.72027
C	-1.59294	-3.55428	-19.10310
C	-0.78732	-4.19155	-20.23273
C	-0.96650	-3.44991	-21.55590
C	-0.15200	-4.08032	-22.68409
C	-0.33900	-3.34179	-24.00466
C	-10.45131	-0.02590	-6.05076
C	-11.23974	0.66816	-4.94279
C	-12.38914	-0.19645	-4.42889
C	-13.17553	0.49265	-3.31533
C	-14.31888	-0.37626	-2.80340
C	5.56636	5.73645	11.52457
C	4.57765	6.19078	12.59893
C	3.24479	6.68073	12.03516
C	2.33743	7.24697	13.12616
C	1.00719	7.73608	12.56401
C	-1.22048	-15.33842	-6.83169
C	-0.45024	-16.41315	-7.59528
C	-1.34475	-17.18262	-8.56510
C	-0.57482	-18.25867	-9.32869
C	-1.47128	-19.02298	-10.29640
C	13.30805	7.22106	-6.66300
C	13.85952	7.82511	-7.96128
C	14.93519	6.93720	-8.58324
C	15.58625	7.55011	-9.82442
C	2.39733	16.43601	-1.39979
C	1.27042	17.25388	-0.77362
C	1.78264	18.20285	0.30802
C	0.65849	19.03078	0.92793

C	1.17425	19.97345	2.00940
C	14.88233	-0.02224	0.27507
C	15.74459	-0.61318	-0.84071
C	17.21005	-0.78763	-0.44290
C	18.01037	-1.61872	-1.44760
C	18.16044	-0.94132	-2.80577
C	14.64566	7.64578	-11.02474
C	15.36637	8.16433	-12.26439
H	6.53663	4.15419	-13.94719
H	5.97318	3.77648	-15.58186
H	-7.27152	10.92316	-12.51662
H	-7.80962	9.65720	-11.40290
H	1.15722	14.78897	-2.02396
H	1.36298	16.04921	-3.24953
H	15.25206	2.02732	-0.27519
H	16.26932	1.43989	1.05098
H	4.19838	4.66336	10.24516
H	4.93058	3.67956	11.52513
H	10.73790	-10.59259	6.15079
H	10.03521	-10.85575	4.54782
H	2.41027	2.11742	8.45105
H	1.34472	1.53051	7.16585
H	-9.65177	1.78317	-6.90420
H	-8.60807	1.02771	-5.69126
H	-1.74415	-5.34104	-17.90955
H	-0.35544	-4.31770	-17.51412
H	0.12996	-15.27551	-5.15566
H	0.49127	-14.09600	-6.42452
H	-6.18163	-4.09031	3.36944
H	-7.58890	-5.16119	3.42849
H	1.19837	0.66467	10.09006
H	0.11141	0.14102	8.80522
H	0.45899	3.01929	9.76511
H	-0.62356	2.50551	8.47142
H	-1.87349	1.07671	10.08089
H	-0.78632	1.57812	11.37494
H	-2.59535	3.44183	9.77258
H	-1.51022	3.94013	11.07009
H	-3.86369	2.01430	11.38292
H	-3.83411	3.69886	11.90902
H	-2.77779	2.51345	12.68020
H	-8.22309	-3.06915	2.36469
H	-8.28794	-4.37100	1.17467
H	-6.04033	-3.69228	0.32522
H	-6.00865	-2.36767	1.48968
H	-8.05211	-1.39945	0.44354
H	-8.08856	-2.73061	-0.71327
H	-5.83441	-0.69398	-0.42943
H	-5.84878	-2.04085	-1.56764
H	-7.87580	0.24804	-1.51785
H	-7.89207	-1.10466	-2.65091
H	-6.56262	0.05876	-2.68274
H	8.32384	-12.36236	5.55061
H	9.02796	-12.10353	7.14556
H	10.48168	-13.32399	4.75770
H	11.17750	-13.08766	6.36233
H	8.79199	-14.87033	5.69559
H	9.39809	-14.56632	7.32277
H	10.26776	-16.67837	6.60316
H	11.61142	-15.53801	6.65630
H	10.10715	-16.33704	4.11745
H	11.66080	-17.00186	4.62538
H	11.53493	-15.30075	4.18916
H	-8.16763	9.60460	-14.43425
H	-8.71559	8.35580	-13.31780

H	-9.65204	11.26295	-13.31495
H	-10.19550	10.01999	-12.18836
H	-10.55949	9.93182	-15.21750
H	-11.10723	8.69395	-14.08799
H	-12.03779	11.60014	-14.11017
H	-12.58266	10.36577	-12.97453
H	-12.97160	10.26832	-16.00582
H	-14.22021	10.65171	-14.81869
H	-13.51640	9.03387	-14.86968
H	5.22117	2.22494	-13.06268
H	4.69285	1.84044	-14.69971
H	7.59224	1.87143	-13.74156
H	7.06867	1.49062	-15.38184
H	6.28455	-0.06851	-12.87775
H	5.77601	-0.45396	-14.52127
H	8.66288	-0.41043	-13.53392
H	8.15364	-0.79952	-15.17692
H	7.37646	-2.37016	-12.67101
H	8.57427	-2.86775	-13.86826
H	6.87007	-2.76087	-14.31489
H	-1.27752	-2.51664	-18.96961
H	-2.65252	-3.54420	-19.36979
H	0.27190	-4.19599	-19.96298
H	-1.09981	-5.23142	-20.35762
H	-0.66130	-2.40800	-21.43022
H	-2.02435	-3.45211	-21.83070
H	0.90641	-4.07275	-22.41190
H	-0.45369	-5.12345	-22.80678
H	-0.02786	-2.30337	-23.90875
H	-1.38440	-3.35841	-24.30670
H	0.25281	-3.80644	-24.79054
H	-10.07274	-0.98337	-5.68532
H	-11.11298	-0.23489	-6.89501
H	-10.56767	0.90325	-4.11377
H	-11.63924	1.61270	-5.32054
H	-11.98906	-1.14198	-4.05408
H	-13.06431	-0.42938	-5.25626
H	-12.49967	0.72581	-2.48878
H	-13.57777	1.43728	-3.68952
H	-13.93680	-1.31593	-2.40928
H	-15.01658	-0.60171	-3.60759
H	-14.86200	0.13314	-2.01021
H	6.53456	5.54527	11.99478
H	5.70840	6.53890	10.79657
H	4.39603	5.36784	13.29491
H	5.03667	7.00472	13.16658
H	3.43320	7.45552	11.28751
H	2.72657	5.85759	11.53965
H	2.84585	8.07600	13.62487
H	2.15168	6.47472	13.87692
H	0.37822	8.13308	13.35798
H	0.47377	6.92071	12.07954
H	1.16922	8.52232	11.82912
H	-1.66596	-14.63487	-7.53917
H	-2.03581	-15.80179	-6.27083
H	0.36628	-15.94596	-8.15183
H	-0.00541	-17.11312	-6.88336
H	-1.78940	-16.48369	-9.27804
H	-2.16151	-17.65031	-8.00946
H	0.24218	-17.79153	-9.88425
H	-0.13150	-18.95881	-8.61623
H	-1.90724	-18.34608	-11.02856
H	-2.28123	-19.51357	-9.76005
H	-0.90164	-19.78261	-10.82758
H	14.12523	7.10190	-5.94819

H	12.89567	6.23267	-6.87821
H	14.27940	8.81093	-7.74679
H	13.03483	7.96063	-8.66289
H	14.49894	5.97007	-8.84571
H	15.71277	6.75544	-7.83638
H	16.44580	6.93524	-10.10487
H	15.96130	8.54712	-9.57973
H	2.90734	15.86138	-0.62287
H	3.13277	17.10945	-1.84681
H	0.53086	16.57690	-0.33833
H	0.76850	17.83302	-1.55290
H	2.27753	17.62355	1.09164
H	2.52799	18.87493	-0.12488
H	-0.08834	18.35930	1.35889
H	0.16614	19.61285	0.14495
H	1.64681	19.41112	2.81241
H	0.35770	20.55514	2.43195
H	1.90889	20.66215	1.59667
H	14.96203	-0.64578	1.16896
H	13.83614	-0.03724	-0.04250
H	15.33558	-1.59109	-1.10901
H	15.67452	0.02901	-1.72048
H	17.25240	-1.28494	0.52998
H	17.68420	0.19017	-0.33858
H	17.52703	-2.59015	-1.57910
H	19.00551	-1.80006	-1.03284
H	17.19682	-0.83594	-3.29766
H	18.80746	-1.53141	-3.45180
H	18.60049	0.04749	-2.69130
H	14.22981	6.65721	-11.23542
H	13.81546	8.31472	-10.79134
H	16.18662	7.50245	-12.53574
H	15.77456	9.15637	-12.08086
H	14.68165	8.22601	-13.10771
C	0.29726	-10.51801	7.16896
H	1.37853	-10.64019	7.15852
H	-0.14464	-11.32391	6.58665
H	-0.05036	-10.59973	8.19666
H	-1.17353	-9.04341	6.62341
H	0.36573	-8.37206	7.18861
C	1.18931	-7.73680	0.38844
H	1.58714	-7.63311	-0.61892
H	1.42643	-6.83633	0.95196
H	0.10739	-7.82922	0.32316
H	1.52324	-9.85317	0.49271
H	2.87348	-8.87969	1.08546
C	-8.74746	-9.45840	-7.44840
H	-8.85910	-9.23752	-8.50780
H	-9.70071	-9.81668	-7.06533
H	-8.01049	-10.25170	-7.33951
H	-9.06462	-7.43625	-6.78217
H	-8.20342	-8.46456	-5.62482
C	-2.93303	-5.01936	-8.46245
H	-1.95456	-4.70135	-8.81627
H	-2.93989	-4.98351	-7.37466
H	-3.67751	-4.31986	-8.83638
H	-3.24038	-6.43801	-10.04277
H	-2.45907	-7.11298	-8.60868
C	1.44028	8.33154	-8.90826
H	2.37990	8.09367	-8.41319
H	1.47571	7.94610	-9.92476
H	0.63104	7.83194	-8.37974
H	0.27511	10.05418	-9.44091
H	1.12267	10.20234	-7.89571
C	6.51735	11.64568	-12.38711

H	6.91705	10.76553	-11.88789
H	7.34589	12.20106	-12.82177
H	5.86868	11.30912	-13.19242
H	6.43298	12.90866	-10.64109
H	5.34707	13.38885	-11.94807
C	10.03058	6.66966	-1.59157
H	9.63922	6.18334	-2.48274
H	10.44045	5.90862	-0.93156
H	10.84260	7.32854	-1.89056
H	8.47404	8.15987	-1.58543
H	8.14253	6.75146	-0.57512
C	12.49024	10.19157	3.72954
H	12.92977	10.67665	4.59859
H	12.67734	10.80932	2.85365
H	12.98157	9.23163	3.58349
H	10.83274	9.37261	4.81984
H	10.51424	10.96107	4.10342
H	11.02574	-1.91527	5.73101
H	8.39734	0.36992	8.22893
H	12.03708	0.13652	4.85410
H	8.71866	6.22404	6.70306
H	8.88745	8.03274	5.04358
H	11.68955	5.76313	2.74946
H	7.83431	11.54989	0.53204
H	10.47120	9.53508	-2.16121
H	6.75156	12.72404	-1.34238
H	8.81757	10.52930	-7.87886
H	7.11387	10.71708	-9.63688
H	4.49512	12.81029	-6.98063
H	-0.16745	11.45448	-11.42380
H	2.81392	8.58013	-12.46513
H	-1.92355	10.07973	-12.48110
H	0.20488	3.53572	-14.45052
H	-1.50930	1.79431	-14.26791
H	-4.53893	4.74791	-13.60372
H	9.53607	6.34338	1.41906
H	10.22881	11.17474	1.65842
H	3.39517	10.79847	-7.75635
H	3.56399	11.53956	-12.58821
H	10.52362	-3.50720	7.20462
H	7.00137	-1.07915	7.45970
H	9.15561	-5.53841	7.15092
H	2.04570	-4.47108	7.14415
H	0.70189	-6.23695	6.07213
H	4.17884	-8.66176	5.53271
H	0.30177	-11.71864	1.03575
H	-2.30285	-8.57193	2.28349
H	-1.04806	-12.09015	-0.99261
H	-6.27982	-7.31094	-2.04348
H	-6.85882	-6.87397	-4.40007
H	-4.33869	-10.07558	-5.69283
H	-4.90355	-6.90347	-11.59463
H	-6.82359	-4.15067	-8.95316
H	-4.57931	-5.13049	-13.27565
H	-6.34638	1.44537	-11.05867
H	-5.05461	3.27461	-12.05016
H	-3.28232	0.58945	-14.87808
H	2.94961	-7.99475	3.24429
H	-1.30891	-10.11007	4.39391
H	-3.65621	-7.79110	-6.71406
H	-7.90952	-6.76940	-8.90321
H	2.95647	6.36479	-11.85820
H	5.01917	5.11960	-12.29460
H	3.59554	4.10092	-16.18530
H	1.53200	5.36720	-15.75988

H	-3.33406	9.25635	-14.49938
H	-5.38234	10.53419	-14.03195
H	-6.67138	7.48177	-11.34467
H	-4.61778	6.21707	-11.78966
H	4.84595	11.89460	-2.58162
H	2.95520	13.13739	-1.62082
H	3.32838	16.07869	-4.68875
H	5.22618	14.84669	-5.64307
H	8.90739	8.67753	-6.27087
H	10.64680	7.19933	-7.18417
H	13.58981	9.21429	-4.84277
H	11.85624	10.70012	-3.93736
H	13.72931	2.11193	4.97184
H	15.35706	1.25582	3.33939
H	12.98610	2.81965	0.15585
H	11.35102	3.65744	1.78091
H	7.14768	4.38393	6.60063
H	5.36130	5.05436	8.14898
H	7.26470	2.87889	11.28769
H	9.06200	2.22598	9.74898
H	3.10120	-2.66164	5.96480
H	1.64418	-0.70272	6.24127
H	3.97902	0.56533	9.57958
H	5.41206	-1.41056	9.33081
H	7.90772	-7.47011	8.24998
H	9.57429	-9.22449	7.81271
H	7.82310	-9.93142	3.98728
H	6.17303	-8.16926	4.41109
H	-1.93042	-1.25017	-14.31383
H	-0.62336	-2.35237	-16.07327
H	-3.97659	-4.80765	-17.03576
H	-5.29728	-3.68708	-15.28936
H	-6.04791	-2.15547	-8.05415
H	-7.26202	-0.97620	-6.28401
H	-9.59329	1.05901	-9.22109
H	-8.38682	-0.13260	-11.00084
H	-6.59156	-8.59172	0.24813
H	-7.83517	-6.86460	1.47761
H	-4.16786	-4.89292	2.41075
H	-2.92559	-6.60977	1.18364
H	-2.96942	-13.14927	-2.31372
H	-1.64392	-14.83864	-3.51169
H	-0.72946	-11.97343	-6.53722
H	-2.03200	-10.28039	-5.33417

*Table 16 Cartesian coordinates of **1c@C₆₀** (GFN2-xTB(GBSA(toluene)). B97-3c(COSMO(toluene) single point energy = 13123.55851405 Hartree. (Used for the calculation of association free energies)*

	X	Y	Z
C	7.02950	0.43446	8.30272
C	8.06345	-0.48193	8.09879
C	9.33042	0.00524	7.73395
C	9.49441	1.34576	7.46154
C	8.41929	2.25504	7.65011
C	7.20166	1.79739	8.10821
C	8.86442	3.53326	7.15060
C	10.22511	3.35410	6.66330
C	8.38263	4.75733	6.78671
C	10.57294	4.55535	5.93415
C	10.63089	2.06553	6.86359
C	11.66250	4.91665	5.16871
C	11.59384	6.07578	4.40119
C	10.47016	6.89420	4.41815
C	9.40121	6.57523	5.26742
C	9.43400	5.40473	5.99115
C	8.45214	11.68354	0.32460
C	9.35477	10.63386	0.20010
C	9.84000	10.28198	-1.06250
C	9.31999	10.90354	-2.17804
C	8.29285	11.87502	-2.04768
C	7.91803	12.31550	-0.79425
C	7.77955	12.10299	-3.38245
C	8.58149	11.29335	-4.29517
C	6.74119	12.67736	-4.05501
C	7.99083	11.41895	-5.60963
C	9.51414	10.58301	-3.59828
C	8.27291	10.85641	-6.83778
C	7.33979	10.97833	-7.86080
C	6.13769	11.65533	-7.67550
C	5.90809	12.32699	-6.46783
C	6.83185	12.22351	-5.45062
C	0.57680	10.59170	-11.24258
C	1.85854	10.06972	-11.12451
C	2.17110	8.84783	-11.72999
C	1.17148	8.12588	-12.34496
C	-0.15099	8.64197	-12.41138
C	-0.43293	9.89044	-11.89619
C	-0.96915	7.59115	-12.98004
C	-0.07859	6.48311	-13.30141
C	-2.27037	7.19126	-13.07923
C	-0.90913	5.36037	-13.66138
C	1.19855	6.77529	-12.91988
C	-0.63296	4.06427	-14.04093
C	-1.67269	3.14758	-14.08508
C	-2.98558	3.50065	-13.76915
C	-3.28201	4.85538	-13.54149
C	-2.25598	5.77116	-13.46575
C	10.26508	7.96342	3.42043
C	9.10295	7.84186	2.65984
C	8.78465	8.71054	1.63413
C	9.66996	9.75711	1.35255
C	10.80929	9.90522	2.12749
C	11.12936	9.02726	3.15968
C	5.02834	11.47398	-8.63202
C	3.85418	10.95202	-8.09086
C	2.78474	10.55063	-8.86985
C	2.89345	10.67897	-10.25902
C	4.02857	11.26843	-10.79624
C	5.10222	11.67541	-10.01160
C	1.59560	9.90831	-8.20816
C	6.28729	12.33321	-10.66795
C	12.37653	9.26796	3.96736
C	7.53270	8.47172	0.83404
C	8.48772	-2.77205	7.24223
C	7.76323	-1.92075	8.07987
C	6.63282	-2.43359	8.73773
C	6.19790	-3.70945	8.45627
C	6.90266	-4.52662	7.53483
C	8.07696	-4.06999	6.97223
C	6.05743	-5.66556	7.26936
C	4.83192	-5.48744	8.04086
C	5.92871	-6.70863	6.39976
C	3.91154	-6.51931	7.60866
C	4.91663	-4.35128	8.79278
C	2.60562	-6.82768	7.93200
C	1.91789	-7.75681	7.15655
C	2.53224	-8.41365	6.09579

C	3.89366	-8.19246	5.84769
C	4.56964	-7.23713	6.57258
C	-0.10460	-11.42130	0.82660
C	-0.62253	-10.62053	1.83727
C	-1.89742	-10.06155	1.69997
C	-2.56478	-10.19927	0.50098
C	-1.98424	-10.94692	-0.55973
C	-0.78607	-11.60450	-0.37240
C	-2.80392	-10.70613	-1.72754
C	-3.89786	-9.84173	-1.30940
C	-2.76361	-10.79262	-3.08914
C	-4.55688	-9.37706	-2.50952
C	-3.79389	-9.55248	0.02033
C	-5.56992	-8.46731	-2.73057
C	-5.81873	-8.04502	-4.03148
C	-5.09257	-8.54377	-5.10881
C	-4.12411	-9.53235	-4.89261
C	-3.83789	-9.92909	-3.60395
C	-4.47393	-5.48954	-10.99759
C	-5.09464	-5.23934	-9.78022
C	-5.67059	-3.98813	-9.53717
C	-5.53564	-2.98705	-10.47583
C	-4.86672	-3.24470	-11.70295
C	-4.36457	-4.50165	-11.97043
C	-4.80515	-1.98410	-12.41026
C	-4.16088	-1.37294	-13.44681
C	-5.21929	0.29756	-12.16563
C	-5.49983	1.58887	-11.76627
C	-4.84413	2.64055	-12.39139
C	-3.93749	2.43397	-13.43192
C	-3.78303	1.13205	-13.93911
C	-4.37394	0.07377	-13.28638
C	1.75062	-9.14956	5.08191
C	1.88406	-8.66302	3.78233
C	1.15585	-9.15708	2.71763
C	0.23108	-10.17899	2.96488
C	0.11599	-10.68915	4.24879
C	0.86830	-10.20444	5.31553
C	-5.16996	-7.87056	-6.42128
C	-3.97414	-7.31154	-6.87320
C	-3.90373	-6.49875	-7.98828
C	-5.09350	-6.23030	-8.67996
C	-6.27417	-6.82737	-8.26495
C	-6.33796	-7.65866	-7.15178
C	-2.61366	-5.85829	-8.43726
C	-7.63247	-8.34873	-6.82043
C	0.76241	-10.88222	6.65431
C	1.33191	-8.52873	1.36141
C	2.36710	5.91193	-12.97756
C	-3.46367	7.98514	-12.83231
C	-3.35575	-2.00024	-14.48240
C	-6.64096	-0.92628	-9.33632
C	-1.82060	-11.54930	-3.89640
C	-4.70575	-8.74060	0.81020
C	6.91727	-7.20000	5.45407
C	3.94119	-3.75214	9.68987
C	7.07259	5.31366	7.08135
C	11.90976	1.47655	6.50288
C	5.67059	13.52496	-3.55399
C	10.49367	9.62232	-4.08145
C	-5.93388	-1.57339	-10.42699
C	-5.48475	-0.99634	-11.58295
C	3.18044	5.72790	-11.85920
C	4.28051	4.89634	-11.92131
C	4.61169	4.23168	-13.09693
C	3.79608	4.40923	-14.20858
C	2.68894	5.23324	-14.15271
C	5.79034	3.30370	-13.13956
C	-3.59855	9.23717	-13.43354
C	-4.73422	9.99499	-13.23171
C	-5.76380	9.53733	-12.41581
C	-5.61691	8.30126	-11.79741
C	-4.48903	7.53069	-12.00447
C	-6.98226	10.38063	-12.17889
C	4.96990	13.18511	-2.39714
C	3.94156	13.98453	-1.93970
C	3.58503	15.14805	-2.61345
C	4.28752	15.48753	-3.76393
C	5.30822	14.68588	-4.23665
C	10.12846	8.61868	-4.97820
C	11.06055	7.70291	-5.42348
C	12.38268	7.76076	-4.99538
C	12.74461	8.76092	-4.10040
C	11.81569	9.67399	-3.64045
C	2.49328	16.03278	-2.08687
C	6.56548	5.24154	8.37977
C	5.32514	5.76683	8.68138
C	4.54500	6.36776	7.69947
C	5.04418	6.42550	6.40320
C	6.28829	5.91109	6.09507
C	3.21764	6.97928	8.03961
C	13.09756	2.13631	6.82134
C	14.31869	1.59146	6.47823
C	14.39586	0.38170	5.79658
C	13.21163	-0.26928	5.46987
C	11.98618	0.26421	5.81760
C	15.72442	-0.22645	5.45541
C	2.60689	-3.61315	9.30950
C	1.69734	-3.00405	10.15126
C	2.08475	-2.52487	11.39786
C	3.41386	-2.67172	11.77918
C	4.33233	-3.26971	10.93917
C	8.23110	-7.41692	5.87200
C	9.18160	-7.88931	4.98928
C	8.85914	-8.14676	3.66124
C	7.55404	-7.91463	3.24182
C	6.59473	-7.45218	4.12095
C	9.88109	-8.70211	2.71323
C	1.10775	-1.80987	12.28442
C	-2.07924	-1.53609	-14.79713
C	-1.32369	-2.15918	-15.77048
C	-1.81903	-3.25339	-16.47026
C	-3.09443	-3.71322	-16.15991
C	-3.84944	-3.10546	-15.17678
C	-6.31138	-1.16368	-8.00162
C	-6.97473	-0.50471	-6.98548
C	-7.99379	0.39940	-7.26151
C	-8.33584	0.62196	-8.59135
C	-7.66920	-0.02275	-9.61339
C	-8.73489	1.08511	-6.15185
C	-1.01627	-3.89441	-17.56372
C	-6.07908	-8.98026	0.72700
C	-6.97175	-8.22117	1.45442
C	-6.52992	-7.18476	2.27173
C	-5.16364	-6.94523	2.35087
C	-4.26093	-7.71300	1.63925
C	-1.55210	-12.88218	-3.58221
C	-0.65936	-13.61706	-4.33657
C	0.00442	-13.04501	-5.41627
C	-0.25356	-11.71297	-5.72000

C	-1.15315	-10.97415	-4.97712
C	0.94407	-13.85416	-6.26107
C	-7.51726	-6.32953	3.01259
C	1.12608	-0.30324	11.99327
C	0.14304	0.46390	12.87419
C	0.16253	1.96273	12.58058
C	-0.81969	2.73649	13.45809
C	-0.79799	4.23072	13.15626
C	-8.32660	-5.44439	2.05464
C	-7.44523	-4.46009	1.28928
C	-8.24796	-3.63368	0.28700
C	-7.36748	-2.66146	-0.49565
C	-8.16789	-1.86953	-1.52334
C	9.82924	-10.23567	2.70050
C	10.84921	-10.83343	1.73420
C	10.78802	-12.35995	1.72032
C	11.84161	-13.00324	0.81653
C	11.59602	-12.75484	-0.66803
C	-6.74373	11.38334	-11.04226
C	-7.96835	12.26094	-10.79435
C	-7.73382	13.27436	-9.67583
C	-8.96190	14.14742	-9.42418
C	-8.72568	15.15635	-8.30594
C	5.40496	1.93761	-12.55991
C	6.55747	0.93807	-12.59198
C	6.14175	-0.41174	-12.01069
C	7.26300	-1.44618	-12.07376
C	6.82854	-2.78749	-11.49336
C	-1.30462	-3.22185	-18.91237
C	-0.50879	-3.85946	-20.04855
C	-0.79086	-3.19096	-21.39257
C	0.00526	-3.82727	-22.53044
C	-0.28205	-3.15929	-23.87037
C	-9.94127	0.25021	-5.70293
C	-10.71324	0.93006	-4.57491
C	-11.90269	0.09451	-4.10638
C	-12.65980	0.76471	-2.96148
C	-13.82794	-0.08533	-2.47474
C	16.20841	-1.14426	6.58580
C	17.55261	-1.78998	6.25779
C	18.03275	-2.70361	7.38420
C	19.42063	-3.29749	7.13571
C	19.45175	-4.30167	5.98830
C	0.20413	-14.46617	-7.45784
C	1.13742	-15.28042	-8.35052
C	0.40671	-15.87348	-9.55350
C	1.33689	-16.68875	-10.44982
C	0.60165	-17.27590	-11.64934
C	13.40010	6.79222	-5.52342
C	13.98105	7.28984	-6.85370
C	15.01000	6.31395	-7.42041
C	15.67258	6.81026	-8.70691
C	3.03727	16.99899	-1.02649
C	1.94582	17.91631	-0.48023
C	2.47490	18.86077	0.59717
C	1.38536	19.78354	1.14024
C	1.91526	20.71434	2.22516
C	3.38272	8.46547	8.38376
C	2.05640	9.11495	8.77108
C	2.22058	10.59882	9.09374
C	0.90148	11.25200	9.50195
C	1.07384	12.73462	9.81265
C	14.72637	6.84886	-9.90603
C	15.45338	7.25250	-11.18414
C	1.54357	-12.19903	6.64934

C	0.34808	-7.37466	1.15929
C	-7.82366	-9.57250	-7.72028
C	-1.41429	-6.08312	-7.52449
C	1.75374	8.38736	-8.15226
C	7.04139	11.38824	-11.60564
C	7.79323	7.50545	-0.32355
C	13.64556	9.01924	3.14936
H	6.04492	0.07986	8.56691
H	10.16383	-0.67349	7.63510
H	6.37470	2.47650	8.25537
H	12.53525	4.28225	5.11479
H	12.41104	6.31427	3.73738
H	8.54597	7.23348	5.31154
H	8.11629	11.97178	1.31121
H	10.56544	9.48549	-1.15457
H	7.18476	13.09977	-0.67672
H	9.17706	10.28657	-6.99360
H	7.51818	10.47000	-8.79614
H	4.98842	12.87726	-6.32707
H	0.35672	11.55345	-10.79985
H	3.17972	8.46558	-11.66123
H	-1.42988	10.30195	-11.95701
H	0.38017	3.74597	-14.23621
H	-1.43413	2.11560	-14.28951
H	-4.30509	5.16609	-13.39092
H	8.43722	7.01427	2.86396
H	11.47271	10.73433	1.92088
H	3.79890	10.81153	-7.01952
H	4.08119	11.40218	-11.86832
H	0.68012	10.14753	-8.75054
H	1.50114	10.29457	-7.19054
H	5.93156	13.18902	-11.24978
H	6.96897	12.70913	-9.90314
H	12.38038	10.30646	4.31122
H	12.37595	8.62249	4.84724
H	6.76809	8.04381	1.48713
H	7.15191	9.41304	0.43488
H	9.35835	-2.39791	6.72601
H	6.07211	-1.81475	9.42182
H	8.63280	-4.68198	6.27686
H	2.10125	-6.32337	8.74307
H	0.86728	-7.92534	7.34673
H	4.38466	-8.74520	5.06017
H	0.86915	-11.87199	0.96141
H	-2.30924	-9.47571	2.50924
H	-0.34880	-12.19665	-1.16293
H	-6.12147	-8.04265	-1.90439
H	-6.55011	-7.26790	-4.20395
H	-3.58385	-9.93795	-5.73563
H	-4.06434	-6.47152	-11.18865
H	-6.18178	-3.81835	-8.60083
H	-3.86543	-4.71006	-12.90537
H	-6.15567	1.78186	-10.93031
H	-4.98556	3.63626	-11.99950
H	-3.15893	0.96073	-14.80303
H	2.56674	-7.84235	3.60763
H	-0.58811	-11.49103	4.42754
H	-3.08713	-7.49652	-6.28810
H	-7.17998	-6.62620	-8.82190
H	-2.78501	-4.78248	-8.54263
H	-2.36569	-6.23249	-9.43475
H	-8.46621	-7.65852	-6.97139
H	-7.63649	-8.67427	-5.78028
H	-0.28696	-11.08929	6.87898
H	1.16367	-10.24068	7.43842

H	2.35157	-8.14562	1.27561
H	1.17818	-9.26751	0.57366
H	2.92834	6.21742	-10.92970
H	4.89707	4.76232	-11.04326
H	4.03485	3.89902	-15.13167
H	2.07480	5.37969	-15.02998
H	6.13137	3.17719	-14.16908
H	6.61382	3.72025	-12.55486
H	-2.81153	9.59643	-14.08142
H	-4.82895	10.95575	-13.71839
H	-6.39992	7.93565	-11.14741
H	-4.38018	6.58370	-11.49571
H	-7.23315	10.93054	-13.08885
H	-7.83077	9.74346	-11.92057
H	5.22201	12.27188	-1.87776
H	3.40045	13.69967	-1.04800
H	4.02643	16.38922	-4.30045
H	5.84924	14.96805	-5.12859
H	9.09962	8.54816	-5.30015
H	10.75776	6.92538	-6.11115
H	13.76770	8.82193	-3.75599
H	12.11597	10.45213	-2.95321
H	1.70502	15.42262	-1.63974
H	2.05578	16.60968	-2.90440
H	7.16588	4.78494	9.15372
H	4.95296	5.70822	9.69484
H	4.44647	6.87605	5.62290
H	6.64264	5.93597	5.07529
H	2.77750	6.46151	8.89451
H	2.53446	6.88296	7.19279
H	13.05068	3.07352	7.35738
H	15.22857	2.11445	6.73850
H	13.25104	-1.20435	4.92832
H	11.07615	-0.23681	5.52142
H	16.46393	0.56103	5.29446
H	15.63994	-0.80918	4.53541
H	2.29848	-3.95937	8.33381
H	0.66886	-2.89523	9.83583
H	3.73262	-2.31030	12.74704
H	5.35882	-3.38800	11.25592
H	8.49216	-7.22862	6.90370
H	10.19223	-8.05914	5.33401
H	7.28752	-8.09482	2.20955
H	5.59520	-7.24789	3.76669
H	9.69112	-8.33126	1.70346
H	10.88105	-8.38128	3.01295
H	1.36566	-1.97398	13.33290
H	0.09952	-2.19409	12.11563
H	-1.66535	-0.70643	-14.24428
H	-0.32970	-1.79304	-15.98801
H	-3.49748	-4.56274	-16.69365
H	-4.84159	-3.46909	-14.94977
H	-5.50531	-1.84012	-7.76116
H	-6.69142	-0.69075	-5.95860
H	-9.13263	1.31410	-8.82656
H	-7.95396	0.14949	-10.64159
H	-9.08451	2.06373	-6.48813
H	-8.06923	1.23450	-5.29878
H	0.04988	-3.80717	-17.34300
H	-1.26495	-4.95554	-17.63469
H	-6.43409	-9.78325	0.09648
H	-8.02985	-8.43214	1.38667
H	-4.79897	-6.14137	2.97527
H	-3.20605	-7.48755	1.69491
H	-2.06793	-13.34002	-2.75004

H	-0.46998	-14.65084	-4.08251
H	0.26349	-11.24700	-6.54748
H	-1.31490	-9.93185	-5.20808
H	1.75212	-13.21839	-6.63003
H	1.38510	-14.65622	-5.66542
H	-6.99206	-5.69676	3.73083
H	-8.20953	-6.96996	3.56579
H	0.87799	-0.14007	10.94152
H	2.13676	0.07950	12.15531
H	0.39366	0.29881	13.92515
H	-0.86634	0.07761	12.71145
H	-0.08721	2.12737	11.52928
H	1.17202	2.34893	12.74316
H	-0.56814	2.57556	14.50931
H	-1.82891	2.34930	13.29743
H	0.19545	4.64037	13.32828
H	-1.50296	4.76048	13.79343
H	-1.06826	4.41415	12.11816
H	-8.85661	-6.07872	1.34078
H	-9.07467	-4.89191	2.62818
H	-6.66734	-5.01303	0.75667
H	-6.94908	-3.79060	1.99652
H	-9.02344	-3.07334	0.81571
H	-8.74748	-4.30743	-0.41413
H	-6.57836	-3.22138	-1.00430
H	-6.88510	-1.97044	0.20023
H	-8.63438	-2.54228	-2.24040
H	-7.52114	-1.18346	-2.06637
H	-8.95058	-1.29225	-1.03509
H	10.01776	-10.60830	3.71042
H	8.82474	-10.55671	2.41417
H	10.65584	-10.44717	0.73192
H	11.85367	-10.51637	2.02618
H	9.79403	-12.67889	1.39663
H	10.93337	-12.72455	2.74084
H	12.82992	-12.62486	1.08908
H	11.84083	-14.08149	0.99647
H	11.68069	-11.69796	-0.90764
H	10.60144	-13.09436	-0.95091
H	12.32577	-13.29639	-1.26650
H	-6.49283	10.83759	-10.12934
H	-5.88779	12.01397	-11.29432
H	-8.22318	12.79340	-11.71437
H	-8.82018	11.62740	-10.53400
H	-7.47425	12.74401	-8.75601
H	-6.88604	13.91239	-9.93828
H	-9.22032	14.67962	-10.34311
H	-9.80976	13.50932	-9.16284
H	-7.89874	15.81781	-8.55689
H	-8.48434	14.64572	-7.37569
H	-9.61394	15.76339	-8.14403
H	5.07111	2.07303	-11.52911
H	4.56145	1.53557	-13.12713
H	6.89180	0.80087	-13.62350
H	7.40062	1.33504	-12.02108
H	5.83501	-0.27579	-10.97062
H	5.27559	-0.78774	-12.56131
H	7.56887	-1.58317	-13.11400
H	8.12948	-1.07514	-11.52083
H	5.96290	-3.17259	-12.02915
H	6.55911	-2.68021	-10.44442
H	7.63262	-3.51650	-11.56893
H	-2.37328	-3.29606	-19.12812
H	-1.05598	-2.16007	-18.84298
H	0.55915	-3.78703	-19.82706

H	-0.76028	-4.92107	-20.11463
H	-1.85848	-3.26376	-21.61527
H	-0.53999	-2.12913	-21.32748
H	1.07294	-3.75132	-22.30956
H	-0.24389	-4.88946	-22.59474
H	-0.02394	-2.10266	-23.83371
H	-1.33772	-3.24444	-24.12097
H	0.29784	-3.62704	-24.66323
H	-9.59276	-0.72965	-5.36842
H	-10.60465	0.09050	-6.55634
H	-11.06951	1.90515	-4.91692
H	-10.04052	1.10262	-3.73115
H	-11.54781	-0.88530	-3.77773
H	-12.58504	-0.06661	-4.94483
H	-13.03239	1.73624	-3.29553
H	-11.97155	0.94291	-2.13147
H	-14.53504	-0.26179	-3.28288
H	-14.35245	0.41367	-1.66268
H	-13.47392	-1.04907	-2.11398
H	15.46180	-1.92326	6.75874
H	16.29551	-0.56250	7.50679
H	18.29704	-1.00799	6.08722
H	17.45377	-2.36320	5.33431
H	17.31383	-3.51493	7.52327
H	18.06115	-2.12757	8.31313
H	19.75191	-3.79817	8.04931
H	20.12632	-2.48902	6.92924
H	18.72774	-5.09631	6.15829
H	20.43975	-4.75045	5.90729
H	19.22091	-3.82107	5.04100
H	-0.60227	-15.10604	-7.09109
H	-0.25293	-13.66451	-8.04298
H	1.95008	-14.63999	-8.70288
H	1.58457	-16.08822	-7.76567
H	-0.40681	-16.51340	-9.20246
H	-0.03950	-15.06531	-10.13863
H	2.15059	-16.04950	-10.80139
H	1.78186	-17.49825	-9.86597
H	0.17316	-16.48325	-12.25961
H	-0.20544	-17.92763	-11.32047
H	1.28136	-17.85703	-12.26896
H	14.21015	6.67179	-4.80092
H	12.93571	5.81612	-5.68171
H	13.16395	7.42544	-7.56403
H	14.45030	8.26424	-6.69657
H	15.78698	6.15008	-6.66882
H	14.52919	5.35109	-7.61130
H	16.08333	7.80902	-8.53907
H	16.50828	6.14687	-8.94560
H	3.83547	17.60177	-1.46665
H	3.47394	16.42274	-0.20726
H	1.13948	17.30869	-0.06146
H	1.52404	18.50382	-1.29969
H	3.28491	19.46550	0.18161
H	2.89114	18.27304	1.41931
H	0.57145	19.17890	1.54803
H	0.97562	20.37879	0.32046
H	2.30642	20.14011	3.06277
H	1.12348	21.36317	2.59357
H	2.71702	21.33814	1.83485
H	3.80701	8.98674	7.52228
H	4.09187	8.56434	9.20935
H	1.64228	8.59997	9.64161
H	1.34354	9.00064	7.95041
H	2.62243	11.11566	8.21855

H	2.94445	10.71364	9.90463
H	0.50363	10.74111	10.38225
H	0.17478	11.13295	8.69439
H	1.78320	12.87379	10.62619
H	0.12443	13.17818	10.10511
H	1.44728	13.26650	8.93975
H	14.27777	5.86147	-10.04165
H	13.91925	7.55900	-9.71768
H	16.25072	6.54720	-11.41068
H	15.89353	8.24193	-11.07573
H	14.76452	7.27392	-12.02607
H	1.16696	-12.86596	5.87674
H	1.45264	-12.69594	7.61294
H	2.59764	-12.01042	6.45515
H	0.46624	-6.63112	1.94532
H	-0.67361	-7.74623	1.18267
H	0.51724	-6.89598	0.19691
H	-7.82428	-9.28068	-8.76846
H	-8.76748	-10.06470	-7.49521
H	-7.01568	-10.28435	-7.56299
H	-1.61052	-5.71051	-6.52112
H	-0.55337	-5.54847	-7.92086
H	-1.15842	-7.13880	-7.46722
H	1.78608	7.97513	-9.15843
H	0.91452	7.93939	-7.62414
H	2.67395	8.11819	-7.63708
H	6.39504	11.04245	-12.40897
H	7.40413	10.51554	-11.06677
H	7.89478	11.89989	-12.04599
H	8.18524	6.56161	0.05039
H	8.51708	7.92925	-1.01577
H	6.87137	7.30922	-0.86735
H	13.68725	9.68708	2.29194
H	13.67206	7.99612	2.78029
H	14.52820	9.18822	3.76276
C	-2.35321	2.60090	-7.03304
C	-3.16104	1.87715	-7.98320
C	-1.44172	3.55051	-7.47560
C	-3.02458	2.12826	-9.34114
C	-1.30183	3.81424	-8.88639
C	-2.07824	3.11483	-9.80051
C	-1.99051	1.69211	-5.97434
C	-3.29554	0.52146	-7.51296
C	-2.57342	0.40710	-6.27105
C	-0.13268	3.62715	-6.87673
C	-3.02183	1.03477	-10.27960
C	-0.73023	1.76650	-5.39696
C	-3.29113	-0.53118	-8.41752
C	0.09363	4.05484	-9.15985
C	-1.48764	2.62952	-11.02084
C	0.21597	2.75211	-5.85669
C	-3.15424	-0.27124	-9.82757
C	0.81591	3.93795	-7.91723
C	-2.06942	1.34525	-11.31442
C	-1.87470	-0.75648	-5.97806
C	0.66139	3.58863	-10.33809
C	-0.14587	2.86383	-11.28578
C	-0.00416	0.55845	-5.09357
C	-2.56563	-1.73958	-8.11453
C	-0.56546	-0.67888	-5.37919
C	-1.87078	-1.85027	-6.91762
C	1.52714	2.15346	-5.83769
C	-2.34194	-1.31655	-10.39620
C	2.07896	3.36176	-7.89873
C	-1.28892	0.33868	-11.86190

C	1.39115	0.79778	-5.36622
C	-1.97890	-2.22517	-9.33881
C	2.44049	2.45235	-6.83966
C	-1.42697	-1.01797	-11.39539
C	1.97299	2.98989	-10.31959
C	0.66648	1.81699	-11.85271
C	2.66744	2.87754	-9.12278
C	0.10583	0.57891	-12.13598
C	0.24748	-1.72490	-5.94828
C	-0.55912	-2.44808	-6.89911
C	1.97490	1.89399	-11.25575
C	2.17397	-0.20964	-5.91374
C	-0.71521	-2.80013	-9.32042
C	3.25302	1.40595	-7.40821
C	-0.11637	-1.61647	-11.37891
C	1.59131	-1.49462	-6.21014
C	0.00750	-2.91368	-8.07776
C	3.39230	1.66841	-8.81904
C	0.83083	-0.63033	-11.83686
C	3.12275	0.10039	-6.95402
C	0.23365	-2.49215	-10.36013
C	2.67182	0.72954	-10.96396
C	3.39515	0.61465	-9.72259
C	2.09162	-0.55547	-11.26062
C	2.18012	-1.97912	-7.43363
C	1.40301	-2.67504	-8.34976
C	3.12673	-0.99367	-7.89306
C	1.54289	-2.41375	-9.76065
C	3.25984	-0.74089	-9.25150
C	2.45463	-1.46485	-10.20277

Table 17 Cartesian coordinates of ***1c@2C₆₀*** *(GFN2-xTB(GBSA(toluene)). B97-3c(COSMO(toluene) single point energy = 15408.77611481 Hartree. (Used for the calculation of association free energies)*

	X	Y	Z
C	8.38154	0.01640	10.02001
C	9.06791	-1.04562	9.42337
C	10.14581	-0.74119	8.57108
C	10.35708	0.55568	8.16551
C	9.50346	1.59481	8.63156
C	8.58747	1.33092	9.63062
C	9.74617	2.73771	7.78589
C	10.82064	2.37243	6.87410
C	9.13911	3.88861	7.36832
C	10.87512	3.39418	5.85140
C	11.23452	1.09056	7.11018
C	11.62374	3.53666	4.70184
C	11.30330	4.55777	3.81429
C	10.26182	5.44200	4.07027
C	9.53165	5.32786	5.25922
C	9.82110	4.30479	6.13534
C	7.24446	9.77505	0.06980
C	8.24488	8.82675	-0.11051
C	8.73681	8.57201	-1.39367
C	8.16113	9.20777	-2.47307
C	7.06452	10.09053	-2.28796
C	6.64688	10.41257	-1.01243
C	6.57458	10.41331	-3.61140
C	7.44843	9.74161	-4.56801
C	5.55204	11.03569	-4.26570
C	6.91436	9.99802	-5.88756
C	8.40578	9.02654	-3.91018
C	7.27710	9.59570	-7.15663
C	6.40295	9.83285	-8.21139
C	5.18063	10.46921	-8.01725
C	4.86353	10.97292	-6.74886
C	5.72636	10.75248	-5.69789
C	-0.19231	9.98078	-11.94308
C	1.08445	9.43611	-11.88242
C	1.40599	8.32330	-12.66729
C	0.41354	7.70234	-13.39557
C	-0.90761	8.22504	-13.38942
C	-1.19409	9.38850	-12.70566
C	-1.72845	7.26483	-14.09399
C	-0.84301	6.21202	-14.57640
C	-3.03099	6.87863	-14.21354
C	-1.68384	5.14421	-15.06381
C	0.43761	6.44889	-14.16289
C	-1.43400	3.90535	-15.61457
C	-2.47828	2.99534	-15.71823
C	-3.78036	3.30030	-15.31015
C	-4.05784	4.62789	-14.93666
C	-3.02509	5.52426	-14.78381
C	9.80596	6.40037	3.04352
C	8.49622	6.24412	2.59628
C	7.95267	7.02606	1.59290
C	8.75392	8.01881	1.01951
C	10.04922	8.19651	1.48409
C	10.59747	7.40501	2.48639
C	4.13490	10.41968	-9.05718
C	2.92917	9.84356	-8.66013
C	1.90185	9.57111	-9.54376
C	2.08805	9.89008	-10.89396
C	3.25915	10.52430	-11.28208
C	4.29015	10.80274	-10.39090
C	0.67818	8.85627	-9.03820
C	5.51671	11.52214	-10.88658
C	12.00670	7.66865	2.94488
C	6.56442	6.72773	1.09669
C	9.23673	-3.50045	8.92554
C	8.52226	-2.40920	9.43079
C	7.14895	-2.58142	9.67682
C	6.51529	-3.73248	9.27208
C	7.22681	-4.73785	8.56653
C	8.60310	-4.65153	8.47789
C	6.22686	-5.58066	7.95473
C	4.92011	-5.08351	8.37581
C	6.08175	-6.48444	6.94087
C	3.92683	-5.81689	7.62135
C	5.07095	-4.00232	9.19491
C	2.54716	-5.81249	7.61423
C	1.87741	-6.54878	6.64418
C	2.56676	-7.30938	5.70567
C	3.96300	-7.37788	5.76051
C	4.64025	-6.63319	6.70261
C	0.18367	-10.02794	0.14570
C	-0.31465	-9.14027	1.09050
C	-1.54293	-8.50711	0.87523
C	-2.19946	-8.70457	-0.32068
C	-1.66189	-9.58560	-1.29817
C	-0.49019	-10.26888	-1.04770
C	-2.50869	-9.46799	-2.46615
C	-3.56108	-8.51820	-2.13434
C	-2.54634	-9.76745	-3.79718
C	-4.27226	-8.22432	-3.35754
C	-3.40721	-8.06363	-0.85728
C	-5.28077	-7.33562	-3.66653

C	-5.61756	-7.14435	-5.00095
C	-4.98927	-7.85512	-6.02022
C	-4.01486	-8.80887	-5.69955
C	-3.63638	-8.97245	-4.38428
C	-4.83370	-5.58227	-12.31454
C	-5.44866	-5.30458	-11.09937
C	-6.14643	-4.10090	-10.93647
C	-6.11836	-3.15849	-11.94099
C	-5.43512	-3.43282	-13.15718
C	-4.83142	-4.65766	-13.35337
C	-5.45785	-2.20817	-13.92590
C	-4.81995	-1.59853	-14.96790
C	-5.99804	0.05269	-13.77118
C	-6.34321	1.33976	-13.41336
C	-5.68956	2.40101	-14.02218
C	-4.72443	2.21431	-15.01552
C	-4.51702	0.90875	-15.49848
C	-5.10380	-0.15911	-14.85629
C	1.84866	-7.91988	4.56890
C	2.20341	-7.46653	3.30119
C	1.54501	-7.87214	2.15375
C	0.47735	-8.76587	2.28418
C	0.14469	-9.24792	3.54173
C	0.81590	-8.85039	4.69193
C	-5.18797	-7.43999	-7.42160
C	-4.03559	-7.07073	-8.11423
C	-4.06426	-6.45454	-9.35206
C	-5.31992	-6.17477	-9.91291
C	-6.46544	-6.61463	-9.26062
C	-6.42952	-7.26588	-8.03490
C	-2.79112	-6.04765	-10.05098
C	-7.70514	-7.81237	-7.45431
C	0.46729	-9.49386	6.00599
C	1.94284	-7.28691	0.82590
C	1.60137	5.60201	-14.36108
C	-4.21687	7.61518	-13.80420
C	-3.96164	-2.22939	-15.95773
C	-7.38650	-1.12355	-10.91958
C	-1.66562	-10.65984	-4.53313
C	-4.24654	-7.10060	-0.16216
C	7.13470	-7.14746	6.19104
C	4.07687	-3.17309	9.85414
C	7.99628	4.55329	7.97067
C	12.33873	0.41108	6.45358
C	4.44161	11.80182	-3.72362
C	9.46572	8.19151	-4.45274
C	-6.62644	-1.77957	-11.96926
C	-6.21077	-1.23215	-13.15097
C	2.46374	5.29639	-13.30797
C	3.54413	4.46099	-13.50554
C	3.80783	3.91133	-14.75524
C	2.95469	4.22408	-15.80735
C	1.86746	5.05391	-15.61645
C	4.94671	2.95085	-14.93441
C	-4.40599	8.92848	-14.23467
C	-5.53145	9.63448	-13.86044
C	-6.49404	9.06093	-13.03610
C	-6.29376	7.75945	-12.59166
C	-5.17680	7.04096	-12.97252
C	-7.69061	9.85260	-12.59642
C	3.71353	11.33336	-2.63016
C	2.65214	12.06121	-2.12998
C	2.28723	13.27717	-2.69777
C	3.01599	13.74418	-3.78593
C	4.07202	13.01758	-4.29941

C	9.20079	7.26363	-5.45944
C	10.21043	6.46813	-5.96411
C	11.51166	6.57335	-5.48512
C	11.77420	7.49953	-4.48182
C	10.76883	8.29298	-3.96549
C	1.16155	14.08696	-2.12433
C	7.95758	4.77083	9.34858
C	6.86751	5.38283	9.93473
C	5.77620	5.78341	9.17151
C	5.81468	5.56359	7.79918
C	6.90611	4.96410	7.20344
C	4.60404	6.47671	9.80114
C	13.53085	1.11302	6.25228
C	14.60695	0.51740	5.62753
C	14.53147	-0.79350	5.16814
C	13.34653	-1.49184	5.36626
C	12.26948	-0.90646	6.00300
C	15.71288	-1.44753	4.51469
C	2.91221	-2.76951	9.20245
C	1.99626	-1.95479	9.83695
C	2.20016	-1.53074	11.14531
C	3.35546	-1.94366	11.80034
C	4.28428	-2.74467	11.16683
C	8.21095	-7.73521	6.85694
C	9.22852	-8.34282	6.14930
C	9.21483	-8.37137	4.75871
C	8.14215	-7.78540	4.09633
C	7.11330	-7.18796	4.79710
C	10.31039	-9.05364	3.99351
C	1.22564	-0.60975	11.81820
C	-2.69294	-1.73450	-16.25566
C	-1.88939	-2.37186	-17.18065
C	-2.32651	-3.51134	-17.84592
C	-3.59421	-4.00214	-17.55176
C	-4.39738	-3.37943	-16.61771
C	-7.04886	-1.24980	-9.57179
C	-7.76658	-0.58056	-8.60094
C	-8.84756	0.22725	-8.93477
C	-9.19626	0.33954	-10.27649
C	-8.47695	-0.31768	-11.25446
C	-9.63743	0.92743	-7.86854
C	-1.46960	-4.17294	-18.88473
C	-5.63404	-7.25509	-0.17512
C	-6.44963	-6.33906	0.45633
C	-5.91368	-5.22853	1.10201
C	-4.53259	-5.07710	1.11511
C	-3.70695	-6.00054	0.50157
C	-1.39286	-11.93627	-4.04050
C	-0.56109	-12.79426	-4.73229
C	0.03639	-12.40360	-5.92553
C	-0.22524	-11.12616	-6.40837
C	-1.06480	-10.26625	-5.72857
C	0.90875	-13.34887	-6.69824
C	-6.81761	-4.21156	1.73777
C	1.63826	0.85436	11.61541
C	0.66439	1.81865	12.28789
C	1.08312	3.27660	12.10929
C	0.09417	4.24187	12.76008
C	0.52659	5.69484	12.60009
C	-7.60952	-3.41828	0.68918
C	-6.70456	-2.60287	-0.23159
C	-7.50429	-1.83774	-1.28408
C	-6.60652	-1.02992	-2.21936
C	-7.41203	-0.27804	-3.27324
C	9.98390	-10.53703	3.77628

C	11.07882	-11.24809	2.98422
C	10.74994	-12.72262	2.75662
C	11.87288	-13.49594	2.06243
C	12.10289	-13.06199	0.61847
C	-7.34750	10.71580	-11.37516
C	-8.53983	11.54749	-10.90899
C	-8.18916	12.42792	-9.71114
C	-9.37833	13.26238	-9.23921
C	-9.01528	14.15178	-8.05535
C	4.51164	1.54633	-14.49804
C	5.64271	0.52507	-14.57352
C	5.18212	-0.85190	-14.09899
C	6.30623	-1.88497	-14.12456
C	5.83114	-3.24922	-13.63727
C	-1.74088	-3.57715	-20.27243
C	-0.89196	-4.24264	-21.35283
C	-1.14897	-3.64569	-22.73502
C	-0.30017	-4.31133	-23.81666
C	-0.55142	-3.70515	-25.19275
C	-10.75300	0.02298	-7.32910
C	-11.55705	0.71041	-6.22824
C	-12.66343	-0.18662	-5.67738
C	-13.46748	0.50075	-4.57549
C	-14.56516	-0.40082	-4.02272
C	16.61201	-2.11823	5.56172
C	17.83411	-2.77558	4.92454
C	18.72119	-3.45343	5.96760
C	20.01284	-4.03133	5.38591
C	19.78025	-5.21900	4.45787
C	0.09058	-14.09710	-7.75895
C	0.95395	-15.05799	-8.57270
C	0.14382	-15.79447	-9.63778
C	1.00498	-16.75434	-10.45652
C	0.19262	-17.48048	-11.52289
C	12.61636	5.74087	-6.06690
C	13.28847	6.47258	-7.23643
C	14.41473	5.64143	-7.84722
C	15.18888	6.37674	-8.94253
C	1.67403	15.02735	-1.02557
C	0.55440	15.88753	-0.44457
C	1.05810	16.82053	0.65482
C	-0.05624	17.69969	1.21952
C	0.44931	18.62352	2.32192
C	4.79008	7.99933	9.76326
C	3.58889	8.73390	10.35348
C	3.76665	10.25038	10.32101
C	2.55152	10.98259	10.88770
C	2.73241	12.49601	10.85904
C	-3.76332	0.78648	-11.97020
C	-3.49183	2.18005	-11.72377
C	-2.41945	2.80200	-12.34636
C	-1.83519	0.70892	-13.47345
C	-2.94924	0.06478	-12.82951
C	-3.77519	2.44789	-10.33636
C	-1.58481	3.71527	-11.60816
C	-0.75107	-0.23759	-13.53966
C	-2.55829	-1.28093	-12.49757
C	-4.21242	0.19221	-10.73913
C	0.55637	0.19432	-13.36358
C	0.82666	1.58867	-13.11455
C	-2.97044	3.32637	-9.62312
C	-0.22409	3.52822	-12.04603
C	-1.85532	3.97238	-10.27037
C	-1.19761	-1.46813	-12.93473
C	-2.99167	-1.85542	-11.31089
C	-3.83179	-1.10259	-10.41512
C	-4.21976	1.21853	-9.72754
C	-2.07860	-2.63642	-10.51409
C	-3.44108	-1.41987	-9.06425
C	-3.84396	0.91275	-8.42701
C	-0.31818	-2.22060	-12.16890
C	-0.77459	4.04978	-9.31931
C	-2.35656	-2.36680	-9.12483
C	-3.44743	-0.43131	-8.08882
C	-3.00777	1.82389	-7.68562
C	-2.09527	1.04287	-6.88844
C	1.44772	3.08840	-8.94326
C	-2.36702	-0.35106	-7.13781
C	-1.32127	-1.26231	-7.19654
C	0.03623	-0.81451	-7.00804
C	-1.31610	-2.28857	-8.20908
C	1.03931	-1.77143	-11.98090
C	1.01726	2.51568	-7.75411
C	-0.76683	-2.81407	-10.93461
C	0.31380	-2.73174	-9.98349
C	-0.34350	2.70237	-7.31651
C	2.29199	2.33815	-9.83932
C	2.67438	1.04402	-9.51232
C	-0.78815	1.47406	-6.70685
C	0.29796	0.52762	-6.76780
C	0.04488	-2.47518	-8.64615
C	0.88061	-1.56447	-7.90439
C	1.41380	1.17173	-7.41477
C	1.43005	-2.08840	-10.63026
C	2.67936	0.01710	-10.52430
C	2.22719	0.44939	-8.27754
C	2.23557	-1.21187	-9.91561
C	1.95552	-0.94446	-8.52688
C	0.53553	3.86931	-9.74078
C	0.81542	3.60307	-11.12942
C	1.90027	2.65600	-11.18975
C	-2.57927	3.00689	-8.27246
C	-1.22207	3.45424	-8.08479
C	-1.57532	2.05223	-13.24143
C	-0.21805	2.50012	-13.05489
C	1.90597	1.66795	-12.16380
C	1.46748	-0.58782	-12.56669
C	2.30138	0.32316	-11.82434
C	14.36802	6.63470	-10.20515
C	15.21673	7.25667	-11.30877
H	5.79763	3.26527	-14.32575
H	5.26267	2.92654	-15.97916
H	-8.51059	9.17861	-12.33976
H	-8.02412	10.50267	-13.40851
H	0.40351	13.42397	-1.70138
H	0.69395	14.68180	-2.91201
H	4.49262	6.15577	10.83884
H	3.68937	6.21710	9.26331
H	16.29644	-0.70091	3.97143
H	15.37238	-2.20118	3.80120
H	11.25186	-8.97376	4.54160
H	10.43844	-8.57164	3.02164
H	0.22562	-0.75922	11.40556
H	1.18914	-0.82418	12.88862
H	-10.08236	1.83902	-8.27325
H	-8.97777	1.20579	-7.04343
H	-1.67838	-5.24470	-18.91088
H	-0.41429	-4.03624	-18.63845
H	1.36406	-14.07431	-6.02081
H	1.70925	-12.79409	-7.19308

H	-6.22711	-3.51948	2.34145
H	-7.52563	-4.71964	2.39815
H	1.68554	1.06814	10.54465
H	2.64075	1.00358	12.02386
H	-0.33405	1.67601	11.86668
H	0.60717	1.58880	13.35494
H	2.07325	3.42401	12.54805
H	1.15972	3.50283	11.04271
H	0.00572	4.00524	13.82339
H	-0.89174	4.10515	12.30883
H	1.48843	5.86028	13.08114
H	-0.20252	6.36397	13.05191
H	0.62133	5.95010	11.54671
H	-8.30174	-2.74682	1.20305
H	-8.20257	-4.10963	0.08652
H	-5.99961	-3.27282	-0.73053
H	-6.12217	-1.89585	0.36480
H	-8.20577	-1.16313	-0.78613
H	-8.09084	-2.54646	-1.87460
H	-6.02216	-0.31671	-1.63249
H	-5.90245	-1.70436	-2.71373
H	-8.10335	0.41720	-2.80067
H	-7.98811	-0.97468	-3.87920
H	-6.75159	0.28571	-3.92918
H	9.03417	-10.61993	3.24221
H	9.85857	-11.02224	4.74747
H	11.20258	-10.74561	2.02318
H	12.02480	-11.17033	3.52630
H	9.83692	-12.80163	2.16109
H	10.55211	-13.19056	3.72491
H	11.61925	-14.55932	2.07352
H	12.79883	-13.37266	2.62962
H	11.18006	-13.13905	0.04667
H	12.85094	-13.69754	0.14879
H	12.45449	-12.03454	0.56954
H	-7.01255	10.06752	-10.56162
H	-6.51777	11.37992	-11.62887
H	-9.36317	10.88126	-10.63935
H	-8.88317	12.17967	-11.73184
H	-7.84228	11.79723	-8.88867
H	-7.36741	13.09531	-9.98309
H	-10.19651	12.59610	-8.95475
H	-9.73169	13.88524	-10.06471
H	-8.67520	13.54900	-7.21555
H	-9.87671	14.73359	-7.73442
H	-8.21735	14.84042	-8.32640
H	4.14325	1.59758	-13.47086
H	3.68052	1.21853	-15.12739
H	6.47543	0.86129	-13.95060
H	6.00622	0.45414	-15.60175
H	4.79398	-0.76829	-13.08042
H	4.36197	-1.19655	-14.73397
H	7.12737	-1.53993	-13.49141
H	6.69082	-1.97661	-15.14341
H	5.45986	-3.18082	-12.61636
H	6.64602	-3.96962	-13.65979
H	5.02580	-3.62081	-14.26799
H	-1.52896	-2.50544	-20.24925
H	-2.79979	-3.69800	-20.51350
H	0.16586	-4.12849	-21.10244
H	-1.11133	-5.31312	-21.37630
H	-0.92867	-2.57537	-22.71256
H	-2.20647	-3.75883	-22.98678
H	0.75705	-4.20396	-23.56155
H	-0.52594	-5.38024	-23.84527
H	-0.31075	-2.64382	-25.19217
H	-1.59640	-3.81813	-25.47484
H	0.06227	-4.19589	-25.94527
H	-10.31041	-0.89643	-6.93860
H	-11.41847	-0.25474	-8.15013
H	-10.88483	0.99374	-5.41444
H	-12.00072	1.62754	-6.62369
H	-12.22041	-1.10359	-5.28094
H	-13.33637	-0.47072	-6.49026
H	-12.79383	0.78888	-3.76494
H	-13.91566	1.41468	-4.97271
H	-14.13685	-1.30768	-3.60076
H	-15.25804	-0.68576	-4.81177
H	-15.12495	0.11012	-3.24231
H	16.03217	-2.87042	6.10222
H	16.93593	-1.36847	6.28780
H	17.49889	-3.51089	4.19093
H	18.41695	-2.01742	4.39509
H	18.15806	-4.25215	6.45690
H	18.98274	-2.71899	6.73434
H	20.65154	-4.35224	6.21302
H	20.54635	-3.24664	4.84377
H	19.21775	-5.99744	4.96984
H	20.73178	-5.63863	4.13764
H	19.22739	-4.92043	3.57083
H	-0.37647	-13.37049	-8.42813
H	-0.71093	-14.65308	-7.26635
H	1.75962	-14.49902	-9.05567
H	1.41506	-15.78717	-7.90174
H	-0.31807	-15.06493	-10.30790
H	-0.66150	-16.35475	-9.15598
H	1.81255	-16.19486	-10.93530
H	1.46273	-17.48780	-9.78809
H	-0.25642	-16.76772	-12.21186
H	-0.60538	-18.06187	-11.06511
H	0.82643	-18.15646	-12.09299
H	13.36499	5.52988	-5.29994
H	12.21657	4.78955	-6.42479
H	13.68990	7.42418	-6.87947
H	12.53332	6.69281	-7.99282
H	14.00075	4.71683	-8.25747
H	15.11434	5.36473	-7.05383
H	16.06178	5.77603	-9.21237
H	15.55408	7.32890	-8.54933
H	2.13028	14.43346	-0.22989
H	2.45169	15.67273	-1.44140
H	-0.22615	15.23944	-0.03783
H	0.10514	16.48254	-1.24383
H	1.49231	16.22583	1.46249
H	1.85004	17.45759	0.25278
H	-0.85144	17.06362	1.61632
H	-0.48511	18.29954	0.41293
H	0.85749	18.04442	3.14795
H	-0.35966	19.24297	2.70341
H	1.23288	19.27681	1.94299
H	4.93860	8.31528	8.72775
H	5.69228	8.26390	10.32014
H	2.69102	8.46601	9.79077
H	3.43935	8.41204	11.38684
H	3.93294	10.57292	9.29000
H	4.65371	10.52316	10.89832
H	1.66578	10.71297	10.30697
H	2.38238	10.65736	11.91719
H	2.88186	12.84328	9.83851
H	3.59857	12.78847	11.44949

H	1.85516	12.99423	11.26637
H	13.94798	5.69033	-10.56054
H	13.53733	7.30435	-9.97543
H	16.03732	6.59500	-11.57967
H	15.63740	8.20420	-10.97758
H	14.61602	7.43998	-12.19721
C	5.74494	1.68301	6.40217
C	7.10521	1.71222	6.13014
C	4.81098	2.24424	5.45816
C	7.58559	2.30629	4.90768
C	5.14813	0.49807	6.96772
C	7.92130	0.56006	6.41588
C	5.27204	2.81698	4.28024
C	6.68666	2.84926	3.99983
C	5.93471	-0.61309	7.23836
C	7.34746	-0.58035	6.95886
C	3.63754	1.40690	5.44005
C	8.69924	1.52256	4.43712
C	3.84607	0.32758	6.37270
C	8.90588	0.44366	5.36889
C	4.57803	2.57394	3.04016
C	6.86587	2.62744	2.58621
C	5.45021	-1.93377	6.92747
C	7.73356	-1.88267	6.47697
C	2.96880	1.17296	4.24591
C	8.87303	1.30916	3.07700
C	3.37978	-0.94547	6.07374
C	9.27600	-0.81024	4.90471
C	5.56268	2.45628	1.99368
C	3.44778	1.76753	3.02342
C	7.93900	1.87164	2.13383
C	4.19637	-2.09844	6.35846
C	8.68021	-1.99589	5.47022
C	6.56262	-2.71929	6.45835
C	2.48267	-0.14876	3.93792
C	9.26028	0.00633	2.59578
C	2.68428	-1.18875	4.83524
C	9.45822	-1.03387	3.49327
C	5.37952	1.53744	0.96906
C	3.25787	0.81333	1.95969
C	7.74798	0.91615	1.07084
C	4.00548	-3.05586	5.29762
C	8.49399	-2.95255	4.40900
C	6.38059	-3.64344	5.43937
C	2.66125	-0.37128	2.52474
C	8.56507	-0.23694	1.35673
C	3.07105	-2.49288	4.35540
C	8.97326	-2.35659	3.18655
C	4.20587	0.70036	0.95173
C	6.49342	0.75224	0.49897
C	5.07741	-3.81288	4.84671
C	7.36498	-3.76079	4.39389
C	3.03447	-1.62591	2.06199
C	8.09712	-1.51038	1.06070
C	3.24329	-2.70610	2.99436
C	8.30575	-2.59059	1.99210
C	4.59374	-0.60251	0.47099
C	6.00779	-0.57007	0.19131
C	5.25669	-4.03316	3.43326
C	6.67117	-4.00072	3.15312
C	4.01939	-1.74330	1.01561
C	6.79451	-1.68015	0.46709
C	4.35739	-3.49070	2.52462
C	7.13238	-3.42783	1.97546
C	4.83663	-2.89621	1.30142
C	6.19817	-2.86485	1.03252
C	1.17432	-10.84559	6.13609
H	2.25353	-10.70996	6.10314
H	0.88655	-11.50766	5.32206
H	0.91366	-11.32006	7.07983
H	-0.61300	-9.64699	6.06434
H	0.77174	-8.85784	6.83691
C	1.06066	-6.08955	0.46613
H	1.41179	-5.62137	-0.45122
H	1.07773	-5.35019	1.26430
H	0.03338	-6.41340	0.31593
H	1.85685	-8.03745	0.03832
H	2.98442	-6.96108	0.87586
C	-8.10804	-9.09648	-8.18450
H	-8.24828	-8.90663	-9.24655
H	-9.03675	-9.48903	-7.77558
H	-7.33294	-9.85203	-8.07091
H	-8.50517	-7.07490	-7.55890
H	-7.57802	-8.03736	-6.39557
C	-1.55854	-5.94326	-9.15857
H	-0.73607	-5.51695	-9.72907
H	-1.24383	-6.91994	-8.79860
H	-1.75151	-5.29728	-8.30481
H	-2.95457	-5.08235	-10.53869
H	-2.58102	-6.77329	-10.84319
C	0.83187	7.34205	-9.19023
H	1.72483	6.99593	-8.67301
H	0.91430	7.07415	-10.24147
H	-0.03220	6.82923	-8.77329
H	-0.20972	9.17716	-9.58353
H	0.53567	9.09533	-7.98174
C	6.31981	10.69018	-11.88848
H	6.63949	9.75046	-11.44314
H	7.20389	11.23834	-12.20738
H	5.72185	10.45936	-12.76703
H	6.15281	11.78962	-10.04120
H	5.20290	12.44893	-11.37649
C	6.61403	5.73017	-0.06264
H	5.60860	5.49613	-0.40675
H	7.09772	4.80749	0.25153
H	7.17593	6.14796	-0.89547
H	6.07453	7.64101	0.75736
H	5.97254	6.30278	1.91060
C	13.03508	7.30611	1.87154
H	14.04235	7.49961	2.23458
H	12.87366	7.89297	0.97018
H	12.95975	6.25363	1.60710
H	12.21199	7.09977	3.85328
H	12.10809	8.73167	3.18199
H	7.62999	-0.18119	10.76958
H	10.74424	-1.53600	8.15875
H	7.98271	2.12019	10.05170
H	12.41891	2.84335	4.46964
H	11.84764	4.64091	2.88543
H	8.73642	6.03057	5.46137
H	6.90819	9.99912	1.07292
H	9.52620	7.84555	-1.52816
H	5.85242	11.12638	-0.85160
H	8.20143	9.06397	-7.32795
H	6.64703	9.44979	-9.19051
H	3.92631	11.48963	-6.59776
H	-0.41456	10.86785	-11.36590
H	2.41501	7.93760	-12.64536
H	-2.18947	9.80777	-12.71944
H	-0.43265	3.60780	-15.88817

H	-2.24572	2.00258	-16.07026
H	-5.07006	4.93648	-14.72486
H	7.89386	5.45788	3.03216
H	10.64970	8.98431	1.04928
H	2.81232	9.55676	-7.62351
H	3.37419	10.80281	-12.32097
H	10.30960	-3.43694	8.81980
H	6.55759	-1.77209	10.07546
H	9.17918	-5.43169	8.00268
H	1.98867	-5.23421	8.33514
H	0.79874	-6.49872	6.59196
H	4.49134	-8.00303	5.05507
H	1.12416	-10.52602	0.33770
H	-1.93645	-7.84012	1.62930
H	-0.07733	-10.94805	-1.77918
H	-5.76432	-6.75672	-2.89324
H	-6.34558	-6.38820	-5.25877
H	-3.54840	-9.37904	-6.48989
H	-4.34134	-6.53361	-12.45220
H	-6.65231	-3.91083	-10.00142
H	-4.32747	-4.88297	-14.28181
H	-7.04107	1.52515	-12.61015
H	-5.88956	3.39308	-13.64900
H	-3.85267	0.73637	-16.33106
H	3.00827	-6.74854	3.21790
H	-0.67043	-9.95437	3.62698
H	-3.08868	-7.23392	-7.62461
H	-7.42523	-6.42790	-9.72436
H	2.26125	5.68614	-12.32125
H	4.19551	4.22786	-12.67459
H	3.14604	3.81224	-16.78869
H	1.22276	5.30600	-16.44652
H	-3.66956	9.38071	-14.88396
H	-5.66933	10.64677	-14.21406
H	-7.02401	7.30196	-11.93841
H	-5.02321	6.03963	-12.59605
H	3.97187	10.37856	-2.19564
H	2.09191	11.67796	-1.28826
H	2.74815	14.68844	-4.23937
H	4.63611	13.40138	-5.13743
H	8.18914	7.15509	-5.82305
H	9.98506	5.74861	-6.73921
H	12.78030	7.59584	-4.09773
H	10.99299	9.01846	-3.19659
H	8.80420	4.47399	9.95151
H	6.85931	5.54909	11.00313
H	4.97290	5.86111	7.18910
H	6.90157	4.77514	6.14079
H	13.60753	2.12929	6.61195
H	15.52051	1.07847	5.48736
H	13.26232	-2.50937	5.00999
H	11.35153	-1.46261	6.11020
H	2.74895	-3.07456	8.18004
H	1.10606	-1.64212	9.30864
H	3.52740	-1.63264	12.82149
H	5.16821	-3.06949	11.69718
H	8.22799	-7.72791	7.93759
H	10.05160	-8.79878	6.68209
H	8.11616	-7.79244	3.01530
H	6.30284	-6.71432	4.26352
H	-2.32031	-0.87069	-15.72627
H	-0.90181	-1.98175	-17.38530
H	-3.95220	-4.88805	-18.05776
H	-5.38233	-3.76834	-16.40114
H	-6.19229	-1.84161	-9.28793
H	-7.47609	-0.67829	-7.56419
H	-10.03888	0.95627	-10.55743
H	-8.76564	-0.23010	-12.29242
H	-6.06228	-8.11417	-0.67198
H	-7.52089	-6.48414	0.44720
H	-4.09455	-4.22078	1.60902
H	-2.63748	-5.84784	0.50233
H	-1.85789	-12.25469	-3.11830
H	-0.36833	-13.78290	-4.33923
H	0.24125	-10.80003	-7.32771
H	-1.23049	-9.26651	-6.10174

*Table 18 Cartesian coordinates of **C_{60}** (GFN2-xTB(GBSA(toluene)). B97-3c(COSMO(toluene) single point energy = 2285.16097392 Hartree. (Used for the calculation of association free energies)*

	X	Y	Z
C	0.20536	2.68840	-1.70688
C	-0.75947	2.06611	-2.57898
C	1.01953	3.70555	-2.18617
C	-0.87416	2.48423	-3.89779
C	0.90022	4.13995	-3.55572
C	-0.02879	3.54079	-4.39540
C	0.75582	1.66741	-0.85058
C	-0.80522	0.66046	-2.26177
C	0.13129	0.41401	-1.19362
C	2.41544	3.74113	-1.82760
C	-1.03919	1.51272	-4.95020
C	2.10007	1.70161	-0.50544
C	-0.96414	-0.27495	-3.27516
C	2.22235	4.44417	-4.04359
C	0.32877	3.22210	-5.75521
C	2.94549	2.75820	-1.00296
C	-1.08330	0.15927	-4.64479
C	3.15883	4.19753	-2.97550
C	-0.29599	1.96889	-6.09830
C	0.87411	-0.75866	-1.17847
C	2.56658	4.13734	-5.35304
C	1.60182	3.51478	-6.22497
C	2.87153	0.48371	-0.48969
C	-0.19263	-1.49282	-3.25951
C	2.27015	-0.72313	-0.81995
C	0.70910	-1.73006	-2.23104
C	4.23947	2.19328	-1.29483
C	-0.38552	-0.79023	-5.47559
C	4.40473	3.65355	-3.25650
C	0.37580	1.05468	-6.89840
C	4.19383	0.78759	-0.97749
C	0.16483	-1.81130	-4.61945
C	4.95511	2.63251	-2.40035
C	0.33016	-0.35099	-6.58108
C	3.86048	3.57235	-5.64495
C	2.29942	2.56544	-7.05605
C	4.76217	3.33507	-4.61645
C	1.69806	1.35857	-7.38625
C	2.96774	-1.67247	-1.65104
C	2.00299	-2.29504	-2.52295
C	3.69547	2.60095	-6.69752
C	4.86557	-0.12660	-1.77765
C	1.41074	-2.35527	-4.90049
C	5.65288	1.68300	-3.23117
C	1.62409	-0.91593	-6.87301
C	4.24081	-1.37981	-2.12076

C	2.34721	-2.60189	-3.83240
C	5.53368	2.11721	-4.60080
C	2.46952	0.14066	-7.37055
C	5.60878	0.32956	-2.92575
C	2.15413	-1.89889	-6.04838
C	4.43826	1.42827	-6.68236
C	5.37475	1.18182	-5.61420
C	3.81377	0.17486	-7.02540
C	4.59834	-1.69849	-3.48058
C	3.66936	-2.29768	-4.32026
C	5.44372	-0.64194	-3.97817
C	3.55005	-1.86331	-5.68981
C	5.32902	-0.22384	-5.29700
C	4.36422	-0.84614	-6.16910
